# Conformationally constrained glycosyl fluoride donors enable chemoselective glycosylation

**DOI:** 10.1039/d6qo01028h

**Published:** 2026-08-26

**Authors:** Timothy M. Emmel, Joshua L. Martin, John Montgomery

**Affiliations:** a Department of Chemistry, University of Michigan 930 N. University Ave. Ann Arbor MI 48109-1055 USA jmontg@umich.edu

## Abstract

Tuning the relative reactivity of glycosyl donors provides a key approach in the development of iterative glycosylations and convergent oligosaccharide assembly methods. While the sugar identity plays an important role in governing reactivity, strategies that activate or deactivate a donor play a key role in effective approaches. This work focuses on the use of constrained glycosides with cyclic protecting groups to alter the reactivity of glycosyl fluoride donors. Boron-catalyzed glycosylations of glycosyl fluoride donors with silyl ether acceptors can be sequenced by using the constrained glycoside effect to deactivate donors that possess this structural feature. The approach allows constrained glycosyl fluorides to be carried through synthetic steps involving similar donors that lack cyclic protecting groups. Additionally, one pot methods can be designed with sequential additions made possible by the constrained deactivation effect. NMR studies provide a simple mechanistic tool for quickly assessing the magnitude of reactivity changes imparted by a given substrate modification.

## Abstract 2

Cyclic protecting groups introduce conformational constraints that sequence the relative reactivity of glycosyl fluorides and enable iterative glycosylation.

## Graphical abstract

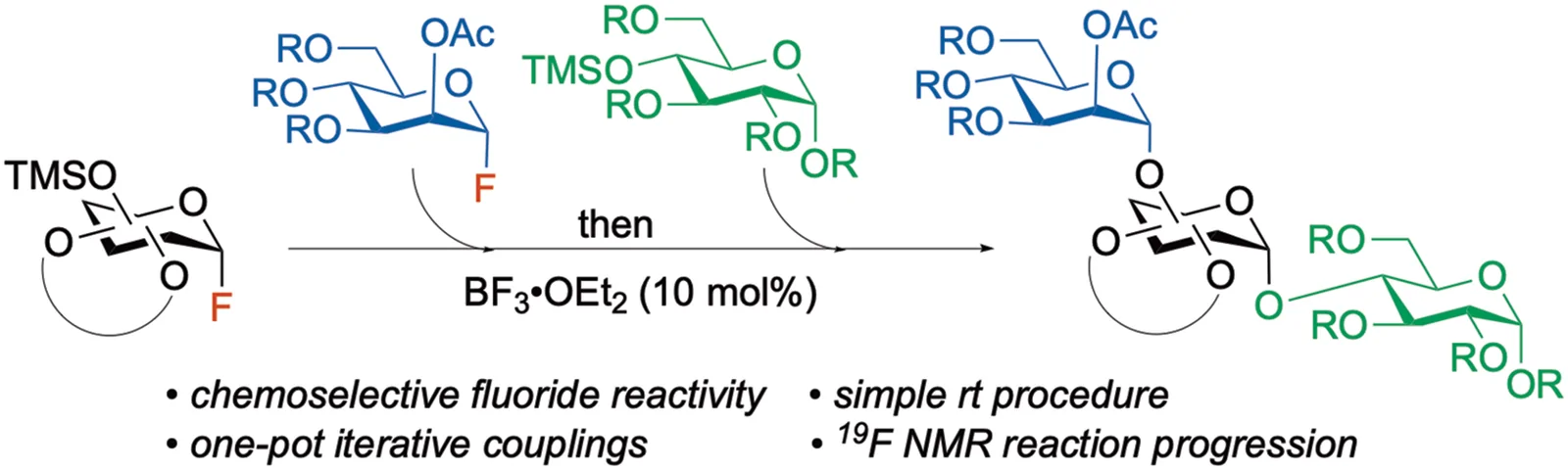

## Introduction

**Scheme 1:**
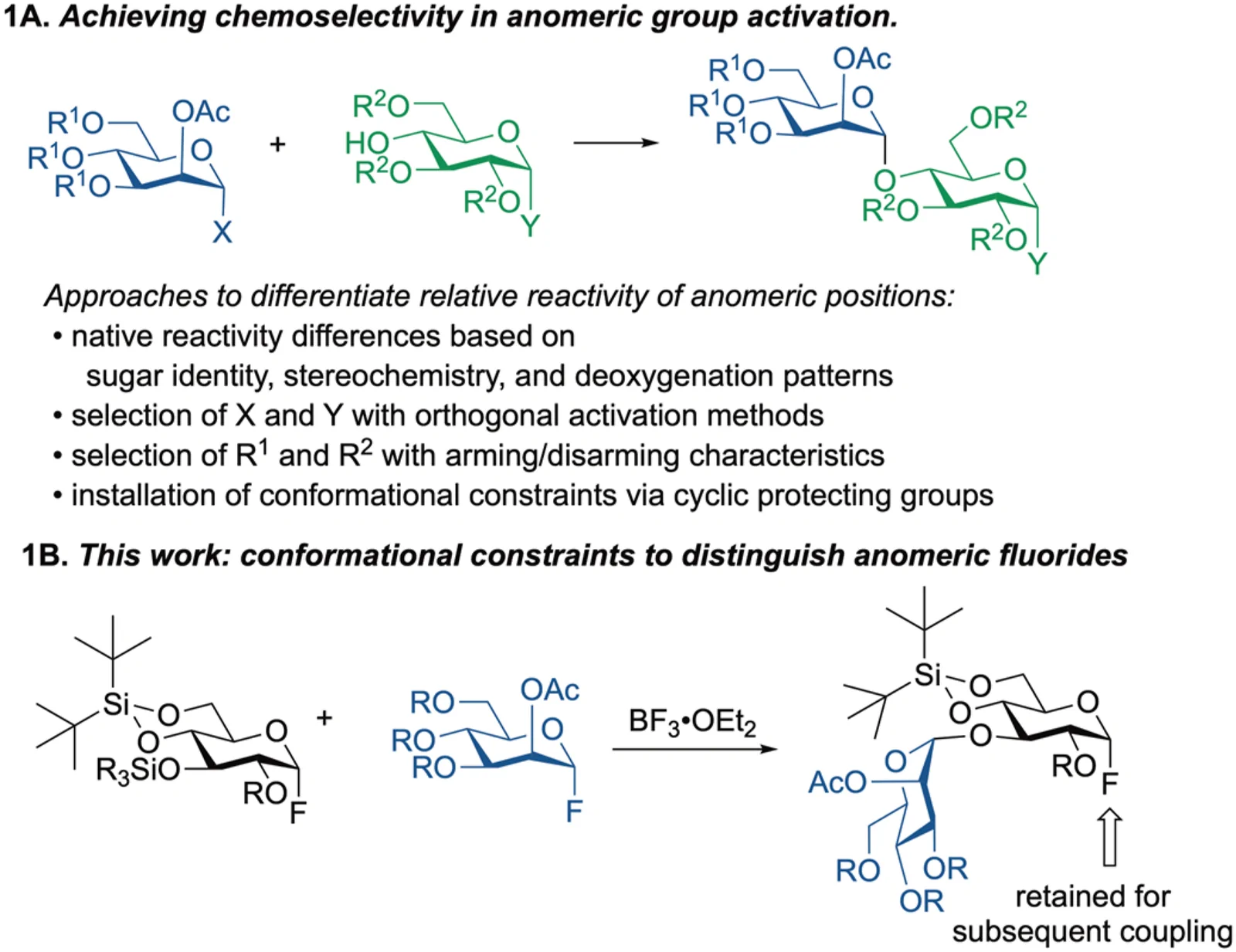
Strategies for differentiating glycosyl donors.

The development of efficient methods for oligosaccharide assembly relies on access to glycosylation strategies that provide regioselective, chemoselective, and stereoselective outcomes.^1–8^ The differentiation of two or more glycosyl donors presents challenges in iterative processes conducted in either a stepwise or one-pot manner (Scheme 1A). Different sugars offer innate differences in reactivity due to the position and stereochemistry of functionality within the sugar.^9–13^ Desired synthetic strategies, however, often require reactivity that differs from that imparted by the innate functionality. Strategies for altering and reversing the relative reactivities of glycosyl donors most commonly involve installation of orthogonal leaving groups that can be activated in a highly chemoselective manner using a specific activating agent.^14–21^ The downside to this approach is that a greater number of building blocks are required that often require installation of the anomeric functionality early in a multistep chemical synthesis. An alternative approach involves the use of armed and disarmed donors, wherein remote protecting groups have an activating or deactivating effect based typically on inductive differences in protected hydroxyls.^22–31^ An alternative approach to differentiating anomeric donor functionality involves the use of conformationally constrained protecting groups.^32–40^ In this strategy, cyclic protecting groups that result in fused bicyclic systems fundamentally alter reactivity and/or stereoselectivity of glycosylation reactions.^41^ Thioglycosides and *n*-pentenyl glycosides have most commonly been employed in this approach, but glucals, alkynylbenzoates, and halide systems have also been illustrated.^33^ This effect derives from the torsional arming or disarming of glycosyl donors that possess the same anomeric leaving group.

Recent work from our laboratory has demonstrated the iterative utility of glycosyl fluoride donors and silyl ether glycosyl acceptors in the presence of boron Lewis-acid catalysts (*e.g.*, B(C_6_F_5_)_3_ and BF_3_·OEt_2_) to allow high-yielding glycosylations.^42–45^ This protocol utilizes readily available substrates, offers ease of use in handling/storage of catalysts/intermediates, and allows stereoselectivity through implementation of intra- and intermolecular variants. While these methods allow regioselective and stereoselective outcomes to be achieved, structural variations that allow two different anomeric fluoride substrates to be distinguished in silyl ether-based glycosylations have not been developed. This restricts their utility and requires further synthetic steps to install orthogonal anomeric handles to allow for selective reactivity to produce desired oligosaccharides. The focus of this work is to explore conformationally constrained glycosyl fluorides, drawing on concepts from past demonstrations with thioglycosides and imidates, that enable effective differentiation of glycosyl fluoride donors (Scheme 1B). This outcome expands the utility of boron-catalyzed glycosylations of glycosyl fluorides with silyl ethers so that stepwise coupling processes can be developed using a single catalytic method for glycosylation using this approach.

## Results and discussion

**Scheme 2:**
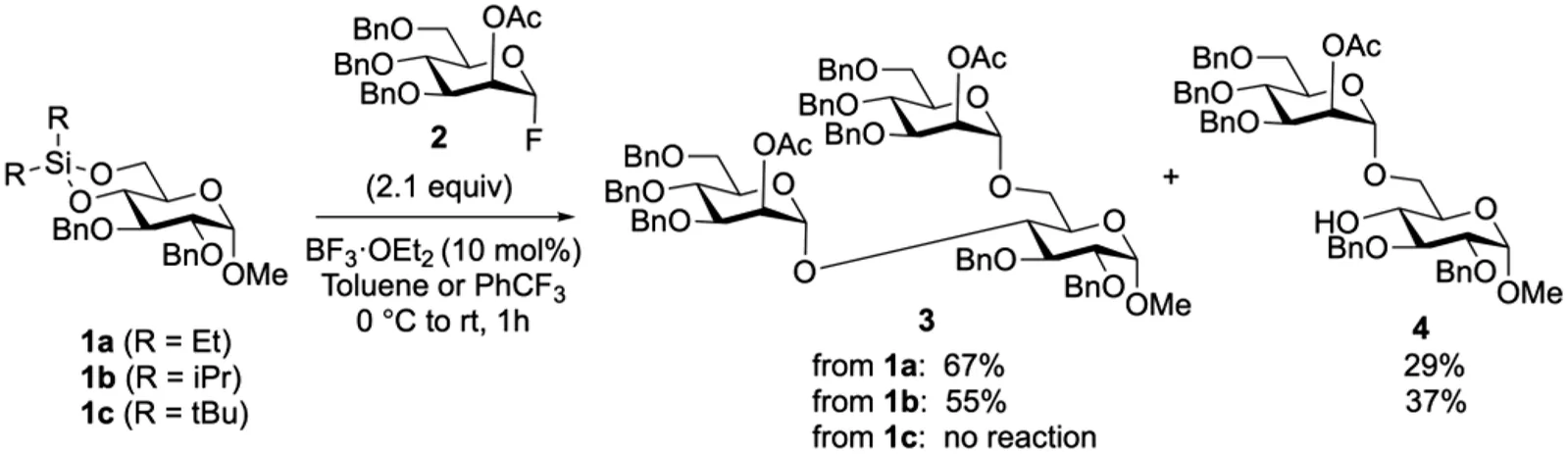
Stability evaluation of constrained glycosides.

In order to identify suitable constrained glycosides that allow differentiation of two or more glycosyl fluorides, we considered cyclic protecting groups at the C4 and C6 positions that would be synthetically relevant, stable to the Lewis-acidic conditions required for glycosylation, and removable under mild conditions to allow for subsequent chemical manipulations. Cyclic silyl ethers have been observed in other donor systems to be highly successful for stereoselective and chemoselective glycosylation reactions and appeared promising here.^46–49^ Cyclic silyl ethers that contain ethyl or isopropyl groups on silicon demonstrated reactivity under boron-catalyzed glycosylation conditions,^42^ however, cyclic di-*tert*-butyl silyl ethers were stable to these conditions (Scheme 2). Furthermore, higher catalyst loading, catalyst choice (B(C_6_F_5_)_3_*vs*. BF_3_·OEt_2_), as well as heating of the system still led to no observable reaction of cyclic di-*tert*-butyl silyl ether 1c, and the silyl ethers are readily deprotected under mild reaction conditions. Based on these observations, we further explored their utility in boron-catalyzed glycosylations and iterative couplings.

**Scheme 3:**
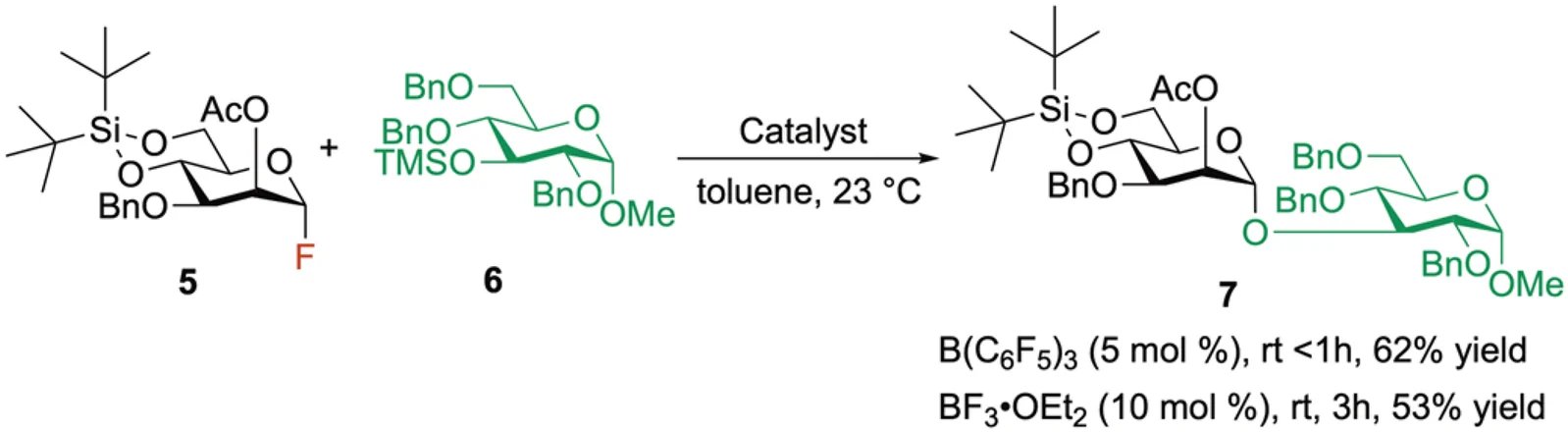
Catalyst comparison in glycosylation of constrained donors.

For the constrained glycoside approach to be successful, we needed to identify conditions that allow unconstrained glycosides to undergo much faster coupling than those possessing the constrained glycosides. Comparison of reaction of 5 and 6 using B(C_6_F_5_)_3_ (5 mol%) and BF_3_·OEt_2_ (10 mol%) illustrated that constrained glycoside 5 underwent complete conversion with B(C_6_F_5_)_3_ in <1 h, whereas BF_3_·OEt_2_ required 3 h for complete conversion (Scheme 3). This suggested that B(C_6_F_5_)_3_ will be superior in cases where constrained glycosyl fluoride consumption is desired, but BF_3_·OEt_2_ will be superior in cases where predictable selectivity is desired, allowing unconstrained glycosyl fluoride consumption while the constrained glycosyl fluoride is retained in unchanged form.

**Scheme 4:**
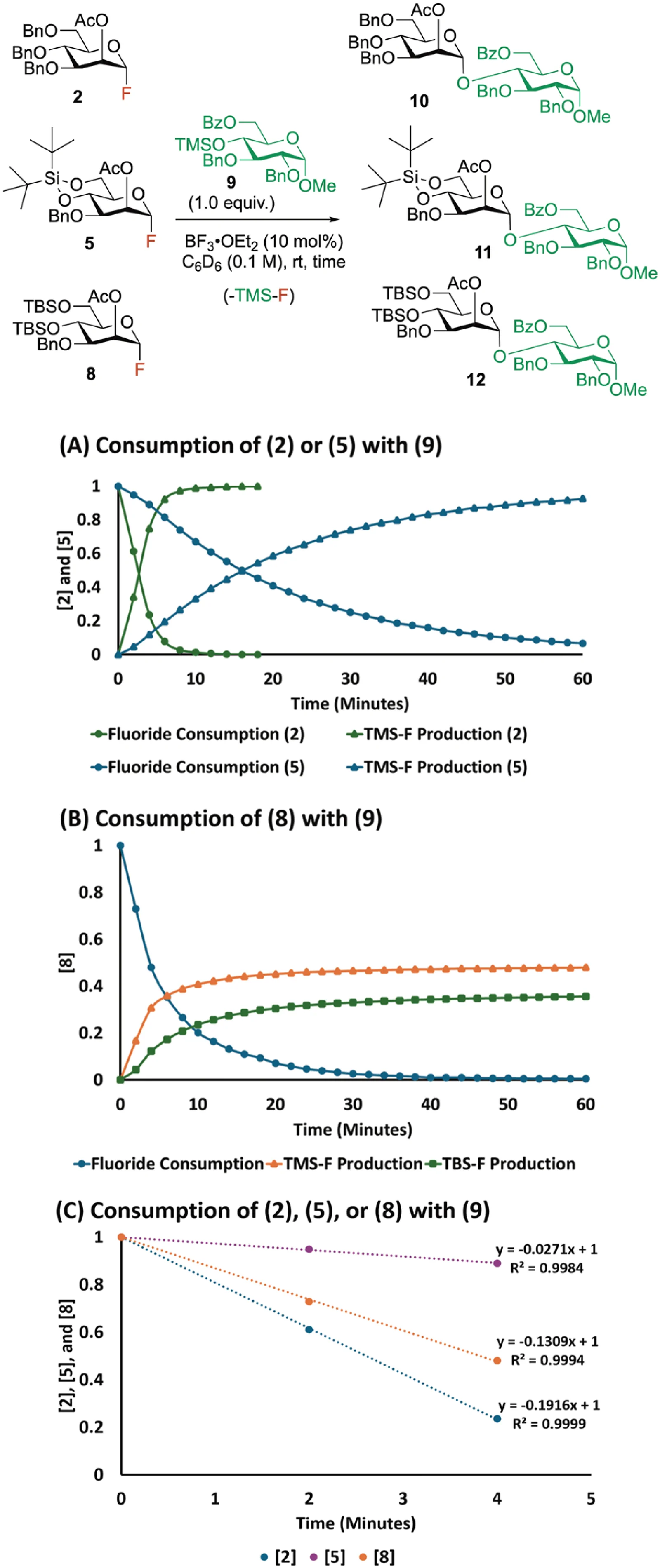
Relative reactivity of constrained and unconstrained donors.

Having identified a cyclic protecting group to explore paired with two different catalysts that display good reactivity for either slow, selective couplings (BF_3_·OEt_2_) or fast, exhaustive couplings (B(C_6_F_5_)_3_), we then examined the rate differences of BF_3_·OEt_2_-mediated couplings on three substrates using ^19^F NMR reaction progression to more quantitatively understand the relative performance of different glycosyl fluorides under these conditions (Scheme 4). The comparison of substrate 2, lacking the cyclic protecting group, with substrate 5 that possesses the cyclic silyl protecting group provides a practical approach for how the strategy will be utilized. Additionally, we added the comparison of substrate 8 to better mimic the armed/disarmed characteristics of substrate 5 so that the precise impact of the constrained protecting group can be decoupled from the inductive differences in the protecting group patterns of substrates 2 and 5.

When evaluating the reaction progression data by ^19^F NMR (Scheme 4), armed donor substrate 2 underwent >90% consumption in ∼5 min (Scheme 4a), while cyclic donor 5 required 1 h to reach the 90% consumption level and ∼3 h to reach full consumption (Scheme 4b). Initial rate comparison between these two substrates at 0.1 M total concentration (Scheme 4c) shows the armed donor 2 fluoride handle undergoes initial consumption 7 times faster than 5. Disarmed silyl donor 8 reaction progression analysis was performed and found that both the TMS and TBS silyl ethers were observed to be reactive during this experiment as production of both TBS-F and TMS-F were observed. Glycosyl fluoride 8 was observed to be fully consumed in 45 min and was found to be 4.8 times faster when comparing the initial rate to cyclic donor 5. This initial rate reflects the combined reactivity of the TMS and TBS acceptor functionalities. In the reaction of substrate 8 in the absence of 9, a mixture of di- and tri-saccharide products were observed by HRMS analysis as the major components along with low levels of a product with mass consistent with the anhydro sugar derived from intramolecular glycosylation from the C6 position, illustrating that intermolecular pathways of TMS and TBS ethers dominate over anhydrosugar formation. The greater reactivity of fully armed unconstrained substrate 2 compared with constrained substrate 5 is primarily attributed to the deactivating effect of the cyclic protecting group in 5 with minor contributions from inductive differences of the protecting group identities.

**Scheme 5:**
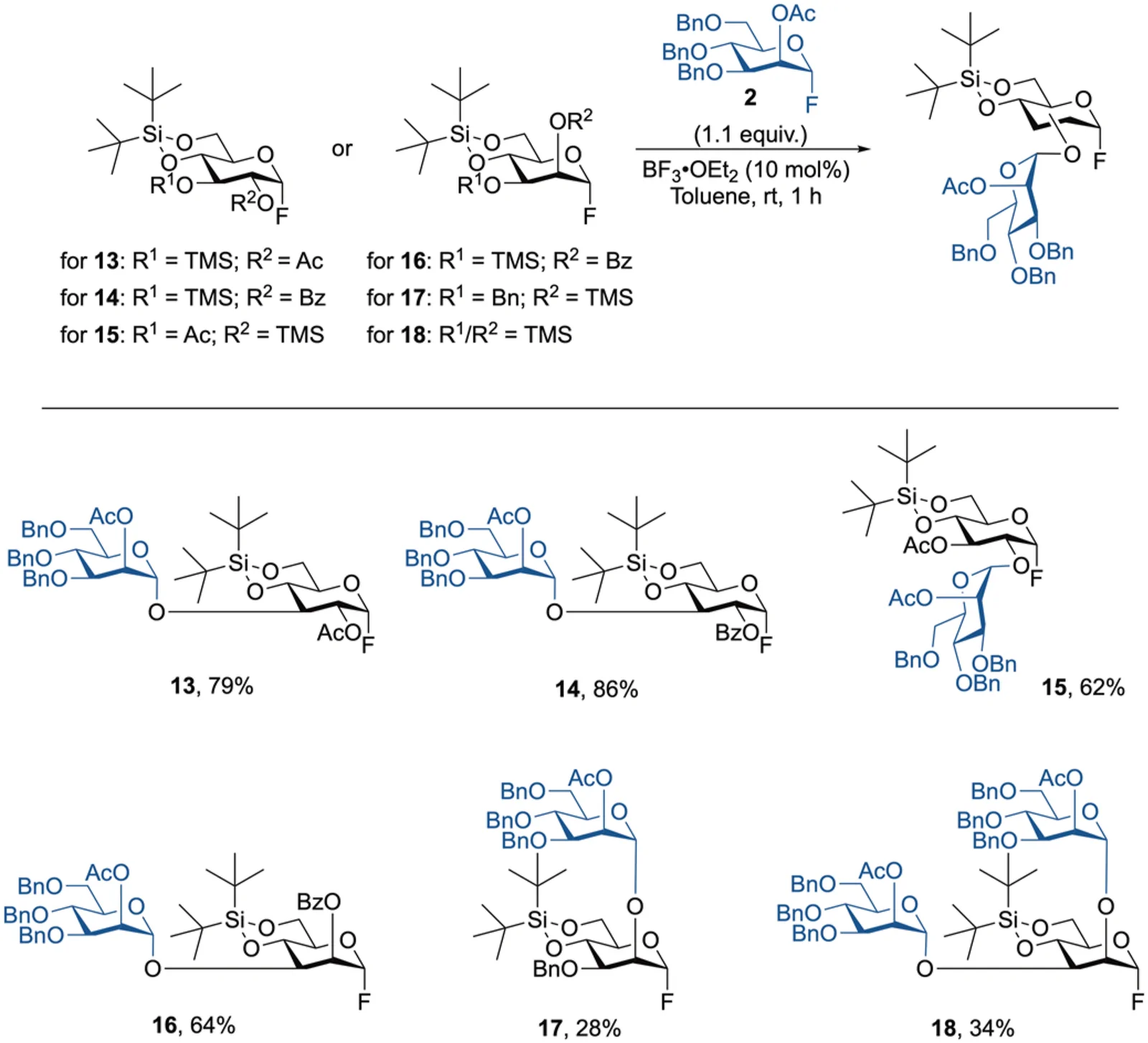
Stability of silyl ether-constrained glycosyl fluorides in glycosylation.

Following this investigation, we then looked into the synthetic utility of the approach, relying on the reactivity differences of the constrained and unconstrained glycosyl fluorides. We first began with the synthesis of substrates that utilize the C4/C6 di-*tert*-butyl cyclic silyl ether patterns that allowed for strategies to selectively install TMS silyl ethers at the C2 or C3 positions for glucoside or mannoside substrates (Scheme 5). We utilized mannosyl fluoride 2 as the donor glycosyl fluoride. With the C3-OTMS intermediates for 13 and 14 in hand, Man(α3)Glu couplings were observed in high yields for disaccharides 13 and 14. Investigations of Man–Man couplings were demonstrated by regioselective low-temperature C2-OBz protection protocols that allowed for C3-OTMS silyl ether installation and subsequent Man(α3)Man couplings in 64% isolated yield for compound 16. Although other examples produced the desired couplings in high yields, Man(α2)Man couplings consistently produced the desired disaccharide 17 in only 28–34% yield, with this potentially being attributed to the deactivating effect of the proximal axial fluoride antiperiplanar to the acceptor functionality. This reduction in yield was also observed in the direct one-pot synthesis of trisaccharide 18 with the same deactivating structural feature. When products 17 and 18 were subjected to standard glycosylation conditions with coupling partner 9, starting materials were largely recovered and no evidence for further reactivity of the remaining glycosyl fluoride was seen under the reaction conditions noted.

**Scheme 6:**
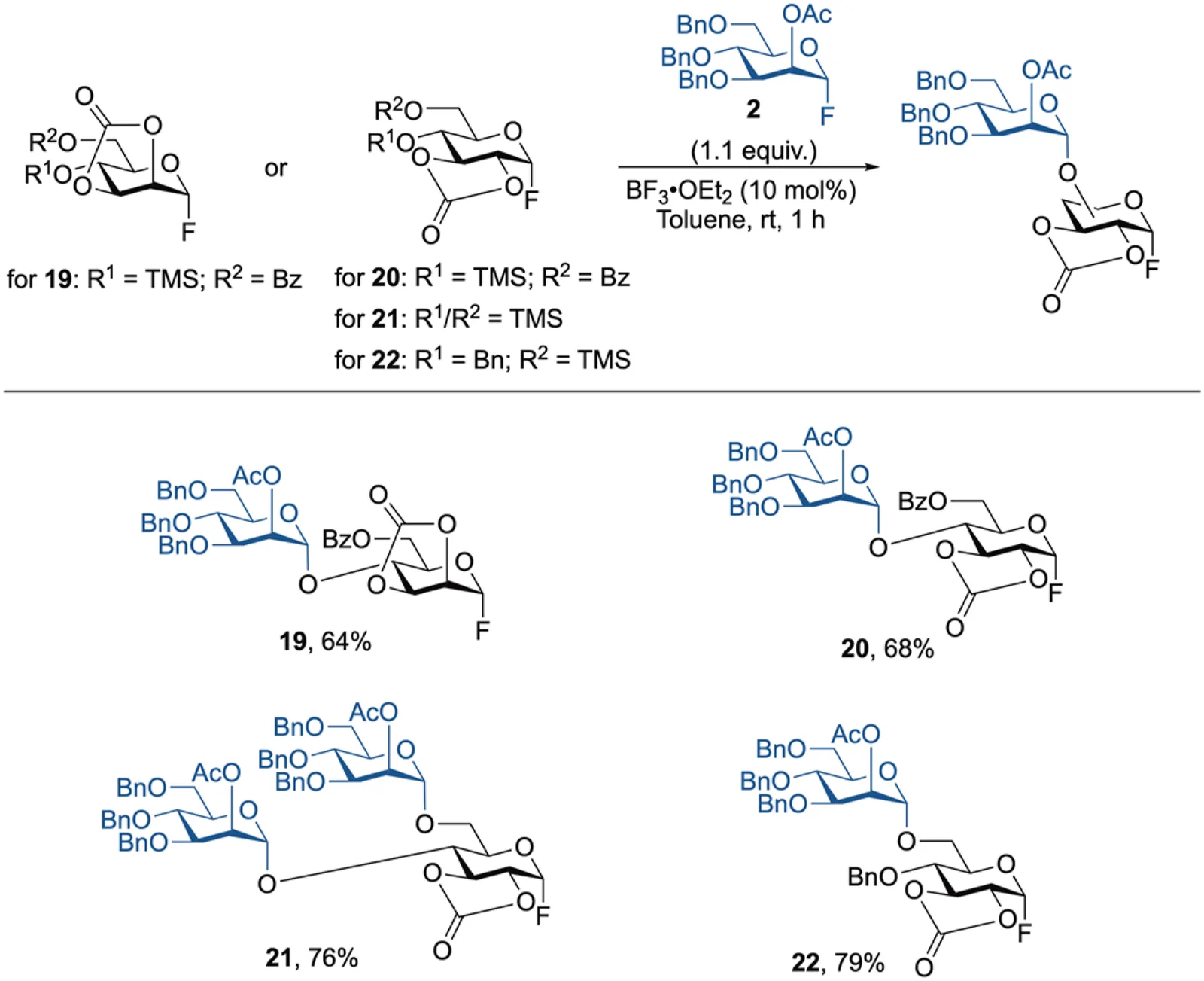
Stability of carbonate-constrained glycosyl fluorides in glycosylation.

To allow for further access to this type of chemoselective reactivity profile for glycosylation events that occur at positions other than the C2 or C3 positions, we envisioned expanding the method to functionalize the C4 and the C6 positions of glycosyl fluoride substrates. Due to the necessity of a readily available reagent to generate a 5-membered cyclic protecting group between C2/C3 positions, we hypothesized that a cyclic carbonate protecting group would provide sufficient constraint of the pyranose ring system. Carbonate protecting groups, as observed by Boons and coworkers,^50^ have also been previously explored to provide chemoselective activation of thioglycosides to generate stereochemically pure carbohydrates or influence the stereochemical outcomes as observed by Crich.^51–53^ Installation of the carbonate group proceeded under mild conditions utilizing carbonyldiimidazole (CDI) and catalytic amounts of dimethylaminopyridine (DMAP) for the mannose substrate when the alcohol substituents at C2 and C3 positions were present in a *cis* configuration. Attempts to generate the cyclic carbonate protecting group for glucose substrates where the C2/C3 alcohols are *trans* required triphosgene as the acylating reagent. After protecting group manipulation to install the desired silylation pattern, these substrates were subjected to the glycosylation conditions (Scheme 6) and were also found to be tolerated in the boron-catalyzed system in 64–79% yields for disaccharides 19, 20, and 22 as well as direct isolation of compound 21 in a one-pot synthesis in 76% isolated yield.

**Scheme 7:**
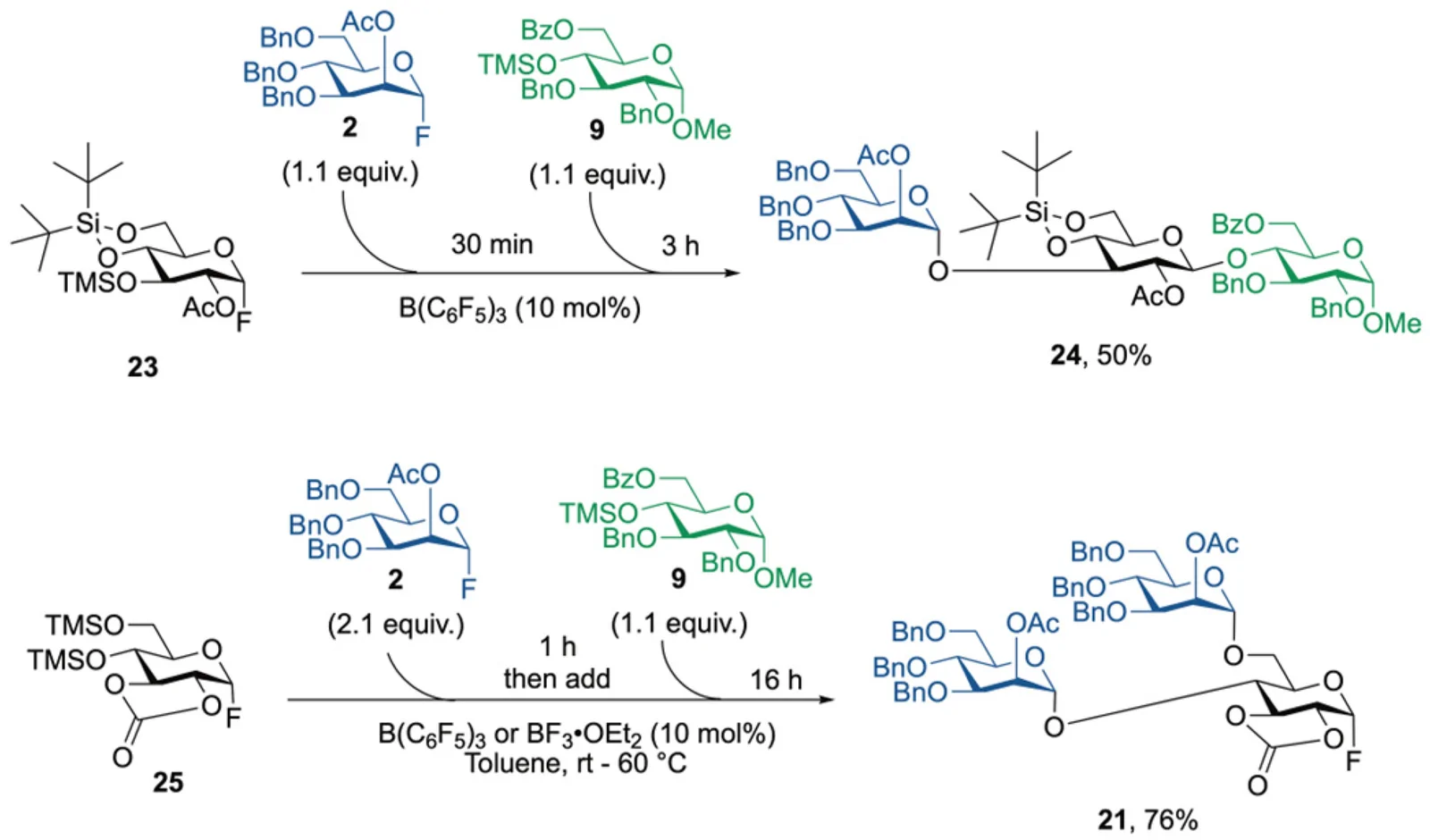
Iterative glycosylations incorporating constrained glycosyl fluorides.

With the success of employing these protecting group strategies, we turned to investigating the reactivity of the conformationally disarmed glycosyl fluoride to determine feasibility of these substrates for incorporation into a one-pot iterative approach (Scheme 7). We first began investigations with the previously described compound 23 that possesses the C2 acetate group that would demonstrate anchimeric assistance and lead to high diastereoselectivity. Treatment of compounds 23 and 2 with B(C_6_F_5_)_3_ over 30 min followed by injection of 9 with continued stirring over 3 h provided a 50% yield of trisaccharide 24. Investigations of one-pot multicomponent couplings of glycosyl fluorides that contain the C2/C3 carbonate protection pattern for the one-pot iterative method began with glucose fluoride 25. After initial trisaccharide coupling product 21 was observed, the addition of TMS silyl ether 9 was found to not lead to any observable desired tetrasaccharide product. The lower reactivity of the cyclic carbonate of 25 compared with the cyclic silyl ether of substrate 23 originates from the intrinsic electron withdrawing effect of the cyclic carbonate paired with the absence of a C2 protecting group geometrically capable of assisting in anomeric group dissociation.^50^ The deactivating effect of the cyclic carbonate plays an excellent protective role for the glycosyl fluoride but will require removal in order for that glycosyl fluoride to be utilized in a subsequent transformation. Based on these findings, the cyclic silyl protecting group possesses the best characteristics for iterative approaches where a constrained glycoside exhibits diminished reactivity but that can still be utilized in a subsequent coupling under more forcing conditions without protecting group removal. This outcome serves as a complement to prior approaches that utilize protecting group modifications as a strategy for controlling selectivity in iterative glycosylations.^23,34^

## Conclusions

In summary, this work outlines an approach for the boron-catalyzed glycosylation of glycosyl fluoride donors with silyl ether acceptors where two or more glycosyl fluorides can be differentiated. The approach relies on the presence or absence of constraints imparted by cyclic protecting groups, with the cyclic constraint deactivating the donor and allowing chemoselective glycosylation. This strategy allows a silyl ether acceptor that possesses a glycosyl fluoride to undergo intermolecular coupling while leaving the glycosyl fluoride within the acceptor substrate unchanged. Iterative couplings become possible where two or more glycosyl fluoride donors can be differentiated through the presence or absence of the cyclic constraint. Direct reaction monitoring by ^19^F NMR allows quantitative analysis of relative rates to provide insights into the magnitude of alterations in kinetic behavior. The strategy allows a versatile approach for the generation of oligosaccharides from simple building blocks through iterative catalysis while also allowing for regio-, stereo-, and chemoselective outcomes.

## Author contributions

T. M. E. was responsible for data acquisition, analysis, NMR/mechanistic studies, and writing the initial manuscript draft. J. L. M. was responsible for project conceptualization and preliminary data. J. M. was responsible for data analysis and project oversight. All co-authors contributed to edits and revisions.

## Conflicts of interest

There are no conflicts to declare.

## Supplementary Material

QO-013-D6QO01028H-s001

## Data Availability

The data supporting this article have been included as part of the supplementary information (SI). Supplementary information: experimental details and NMR spectra. See DOI: https://doi.org/10.1039/d6qo01028h.
