# Supplementary material for: Conformationally constrained glycosyl fluoride donors enable chemoselective glycosylation
Source: Org Chem Front. 2026 Aug 26;13(20):7060–6. doi: 10.1039/d6qo01028h (PMC13537277; doi:10.1039/d6qo01028h)
Supplement: Supplementary file 1 [file QO-013-D6QO01028H-s001.pdf]

Supplementary Information (SI) for Organic Chemistry Frontiers.
This journal is © the Partner Organisations 2026

Supplementary Information

Conformationally Constrained Glycosyl Fluoride Donors Enable Chemoselective Glycosylation

Timothy M. Emmel, Joshua L. Martin, John Montgomery*

Department of Chemistry, University of Michigan, Ann Arbor, MI 48109-1055, USA

I. **General Considerations**

II. **General Procedures**

III. **Synthetic Procedures for Starting Materials**

IV. **Experimental Procedures for Boron Catalyzed Glycosylations**

V. **References**

VI. **Copies of NMR Spectra**

Experimental Section

I. General considerations. The following abbreviations are used throughout this supporting information, trimethylsilyl (TMS), ethyl acetate (EtOAc), dichloromethane (DCM), tetrahydrofuran (THF), Methanol (MeOH), diethyl ether (Et_2O), and dimethylformamide (DMF).

All experiments were carried out under a dry nitrogen atmosphere unless otherwise specified. All reagents were purchased from commercial suppliers and were used without further purification unless otherwise specified. "Room temperature" for all experiments was approximately 23 °C and "high vacuum" refers to approximately 10^{-3} Torr. Boron trifluoride diethyl etherate and tris(pentafluorophenyl)borane were purchased from Sigma Aldrich and used as received. D-mannose and 2,3,4,6-tetra-*O*-acetyl-a-D-glucopyranoside were purchased from Carbosyn, and TCI respectively, and used as received. All reactions conducted above room temperature were heated employing an oil bath. All solvents were dried under nitrogen using a solvent purification system (Innovative Technology Inc. Model #SPS400-3 and PS-400-3) or distilled while stirring over CaH_2.

Chromatographic purifications were carried out over silica gel unless otherwise specified. Kieselgel 60 F254 (250µm silica gel) glass plates were utilized for TLC analysis and were visualized by UV irradiation (254 nm) and by charring with sulfuric acid in ethanol (10:90, v/v) or with ceric ammonium molybdate solution [$Ce(SO_4)_2$: 4 g, $(NH_4)Mo_7O_{24}$: 10 g, H_2SO_4: 40 ml, H_2O: 360 mL]. Flash column chromatography was performed using SiliaFlash® P60 (230-400 mesh) silica gel.

^1H and ^{13}C NMR spectra of all compounds were recorded using 500, 600, and 700 MHz instruments, and all ^{19}F NMR spectra were collected on 400 MHz instruments. ESI-HRMS were recorded using an Agilent 6230 TOF HPLC-MS. All NMR spectra presented are in $CDCl_3$ (Cambridge Isotopes) unless specifically noted.

This document contains procedures that utilize HF•Pyridine and diethylaminosulfur trifluoride (DAST). DAST is known to release one equivalent of HF during deoxy-fluorination or nucleophilic opening of orthoesters. **Buffered or unbuffered HF sources can etch glassware and are extremely hazardous, these should only be handled by experienced personnel after reading the appropriate standard operating procedures and safety data sheets (SDS).**

This document contains procedures that utilize triphosgene. Triphosgene is known to generate phosgene gas and is known to be fatal upon inhalation along with severe skin and eye irritation. **Triphosgene should only be handled by experienced personnel in a properly ventilated fume hood at all times after reading the appropriate standard operating procedures and safety data sheets (SDS).**

II. General Procedures.

General procedure A for trimethylsilyl protection of glycosyl acceptors (A)

A stirred solution of alcohol (0.62 mmol) in 3.1 mL dichloromethane was treated with triethylamine (3.09 mmol) or 2,6-lutidine (3.09 mmol) followed by chlorotrimethylsilane (1.85 mmol) at 0 °C under nitrogen. The reaction mixture was allowed to warm to rt while stirring was continued for 2 h before dilution with dichloromethane (50 mL). The dichloromethane layer was washed with saturated aq. $NaHCO_3$ (2 X 50 mL), washed with saturated brine (50 mL), dried over anhydrous $MgSO_4$, and concentrated under reduced pressure. The residue was subjected to column chromatography on silica gel to give the desired TMS ether product.

General procedure B for 1:1 couplings catalyzed by BF_3•OEt_2 for disaccharide synthesis (B)

The oven-dried reaction flask was charged with a magnetic stir bar and was allowed to cool under nitrogen. The flask was charged with the silyl ether acceptor (0.207 mmol) and the corresponding acyclic glycosyl fluoride donor (0.228 mmol). The reaction flask was placed under nitrogen with 3 vacuum/refill cycles, and 4.1 mL anhydrous toluene was injected. BF_3•OEt_2 (20.7 μmol, neat) was added to the reaction mixture via syringe under nitrogen and the reaction mixture was stirred at rt. After stirring for 1 h, the reaction mixture was quenched by the addition of triethylamine, concentrated under reduced pressure, and subjected to column chromatography on silica gel to give the desired disaccharide product.

General procedure C for 2:1 couplings catalyzed by BF_3•OEt_2 for trisaccharide synthesis (C)

The oven-dried reaction flask was charged with a magnetic stir bar and was allowed to cool under nitrogen. The flask was charged with the silyl ether acceptor (0.172 mmol) and the corresponding acyclic glycosyl fluoride donor (0.344 mmol). The reaction flask was placed under nitrogen with 3 vacuum/refill cycles, and 3.45 mL anhydrous toluene was injected. BF_3•OEt_2 (17.2 μmol, neat) was added to the reaction mixture via syringe under nitrogen and the reaction mixture was stirred at rt. After stirring for 1 h, the reaction mixture was quenched by the addition of triethylamine, concentrated under reduced pressure, and subjected to column chromatography on silica gel to give the desired trisaccharide product.

General procedure D for 1:1:1 couplings catalyzed by $B(C_6F_5)_3$ for trisaccharide synthesis (D)

The vial was charged with a magnetic stir bar and flame-dried and cooled under nitrogen. The flask was charged with the silyl ether acceptor containing cyclic anomeric fluoride (69.0 µmol) and the corresponding acyclic glycosyl fluoride donor (69.0 µmol). The reaction flask was placed under nitrogen with 3 vacuum/refill cycles before the addition of 0.69 mL anhydrous toluene. $B(C_6F_5)_3$ (6.90 µmol) was added to the reaction mixture under nitrogen, and the reaction was stirred at rt for 30 minutes. The second silyl ether acceptor (69.0 µmol) added to the reaction mixture and was stirred at rt for 3 h. The reaction mixture was quenched by the addition of triethylamine, concentrated under reduced pressure, and subjected to column chromatography on silica gel to give the desired trisaccharide product.

General procedure for NMR experiments and corresponding data

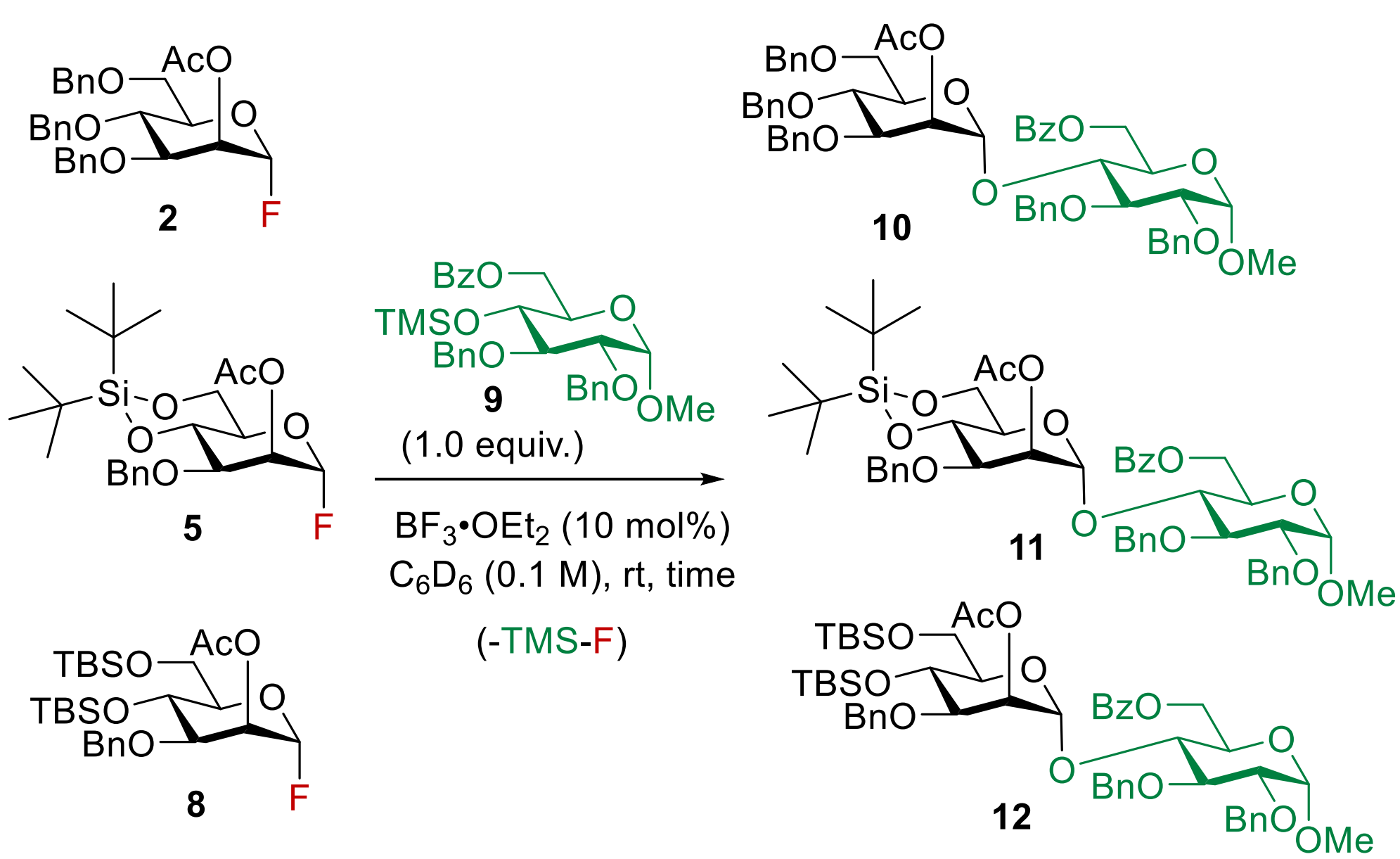

In a nitrogen-filled glovebox, a 1-dram vial was charged with silyl ether acceptor (**9**) (0.10 mmol), glycosyl fluoride donor (**2**, **5**, or **8**) (0.10 mmol), catalyst (0.01 mmol), and α,α,α-trifluorotoluene (0.10 mmol, internal standard) in benzene-d_6 (1.00 mL). The resulting solution was transferred to an NMR tube, sealed with a Teflon-lined screw cap, and removed from the glovebox. ^{19}F NMR

spectra were collected every 2 min to monitor the progression of the reaction. The disappearance of the fluoride peak and the concurrent appearance of the silyl fluoride peak indicated the desired coupling. The zero-minute data were obtained from a separately prepared NMR sample containing the same concentrations of silyl ether acceptor (0.10 mmol), mannose fluoride donor (0.10 mmol), and internal standard (0.10 mmol) in benzene-d_6, (1.00 mL) but without the catalyst. The extent of mannose fluoride consumption was quantified by integrating its signal relative to the internal standard. The disappearance of the mannose fluoride was plotted against time to evaluate the relative reaction rate in the presence of different silyl ether acceptors and catalysts. The consumption of mannose fluoride is reported as a normalized value, calculated as the percentage of mannose fluoride consumed divided by its initial percentage at time zero (i.e., before catalyst addition). Representative ^{19}F NMR spectra showing the reaction progression are provided below, using silyl ether acceptor and mannose fluoride in the presence of $BF_3 \cdot OEt_2$ as the catalyst.

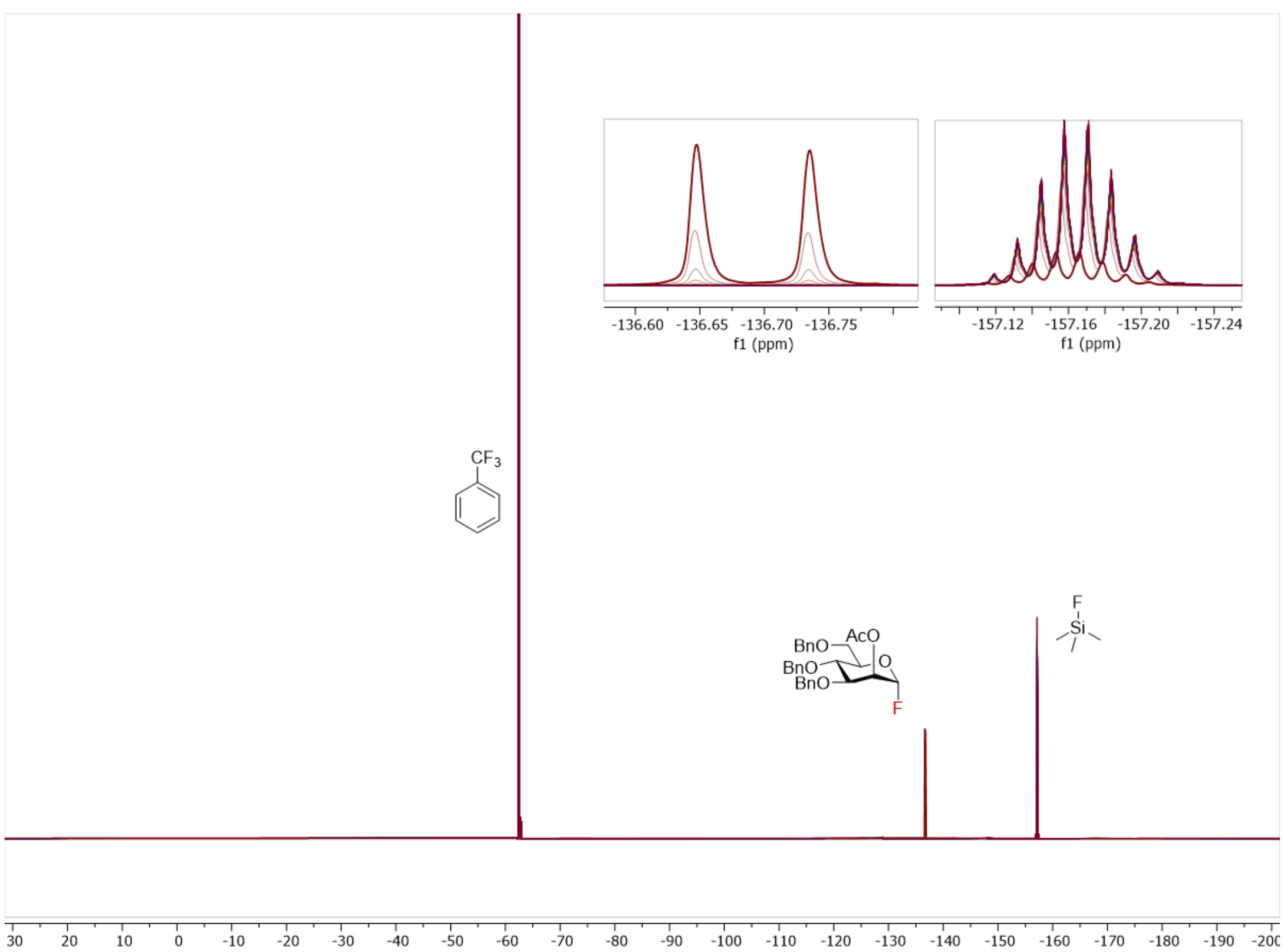

Figure S1. ^{19}F NMR spectra showing the reaction progression of compound (**2**) and (**9**) in 10 mol% $BF_3•OEt_2$

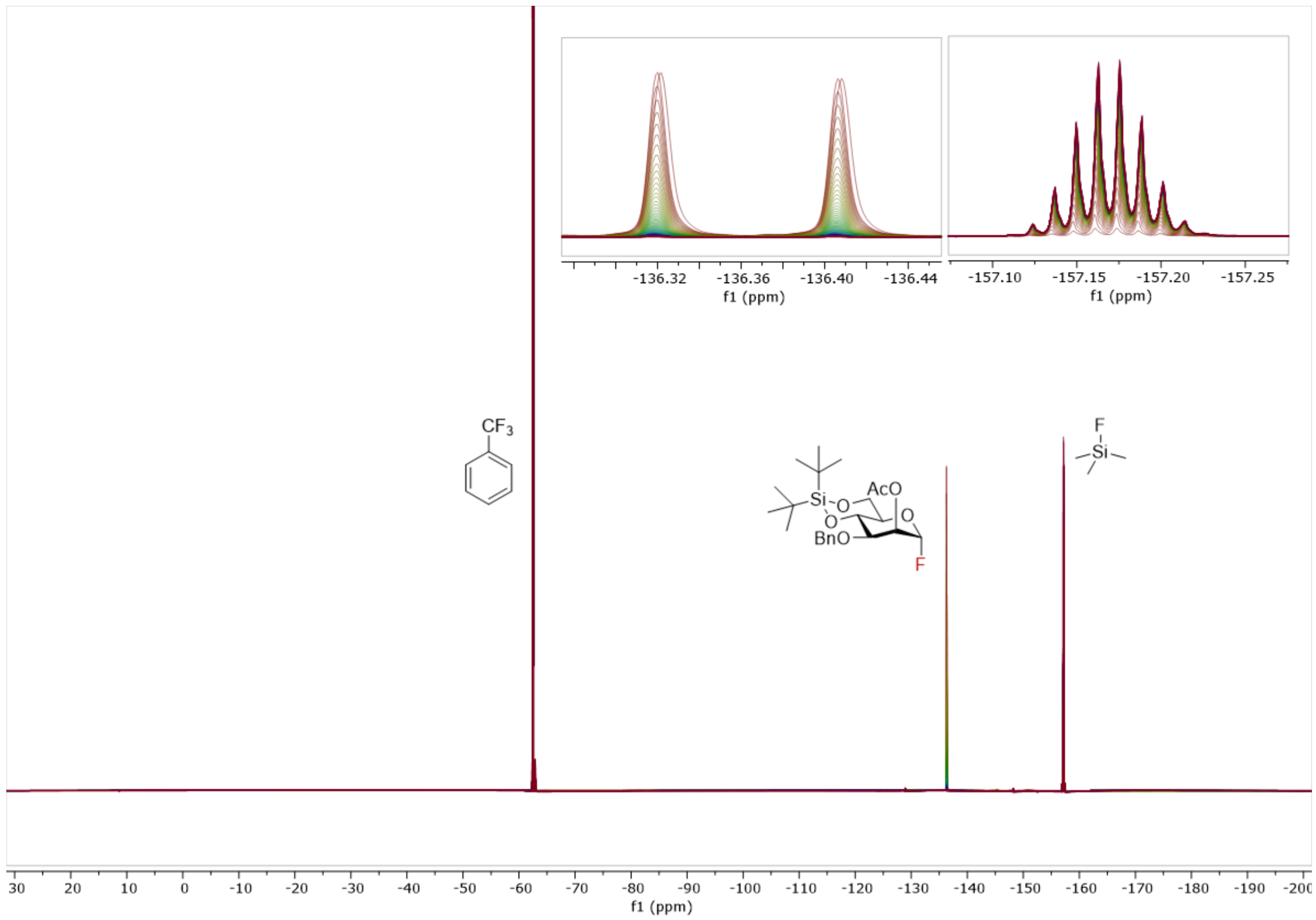

Figure S2. ^{19}F NMR spectra showing the reaction progression of compound (**5**) and (**9**) in 10 mol% $BF_3•OEt_2$

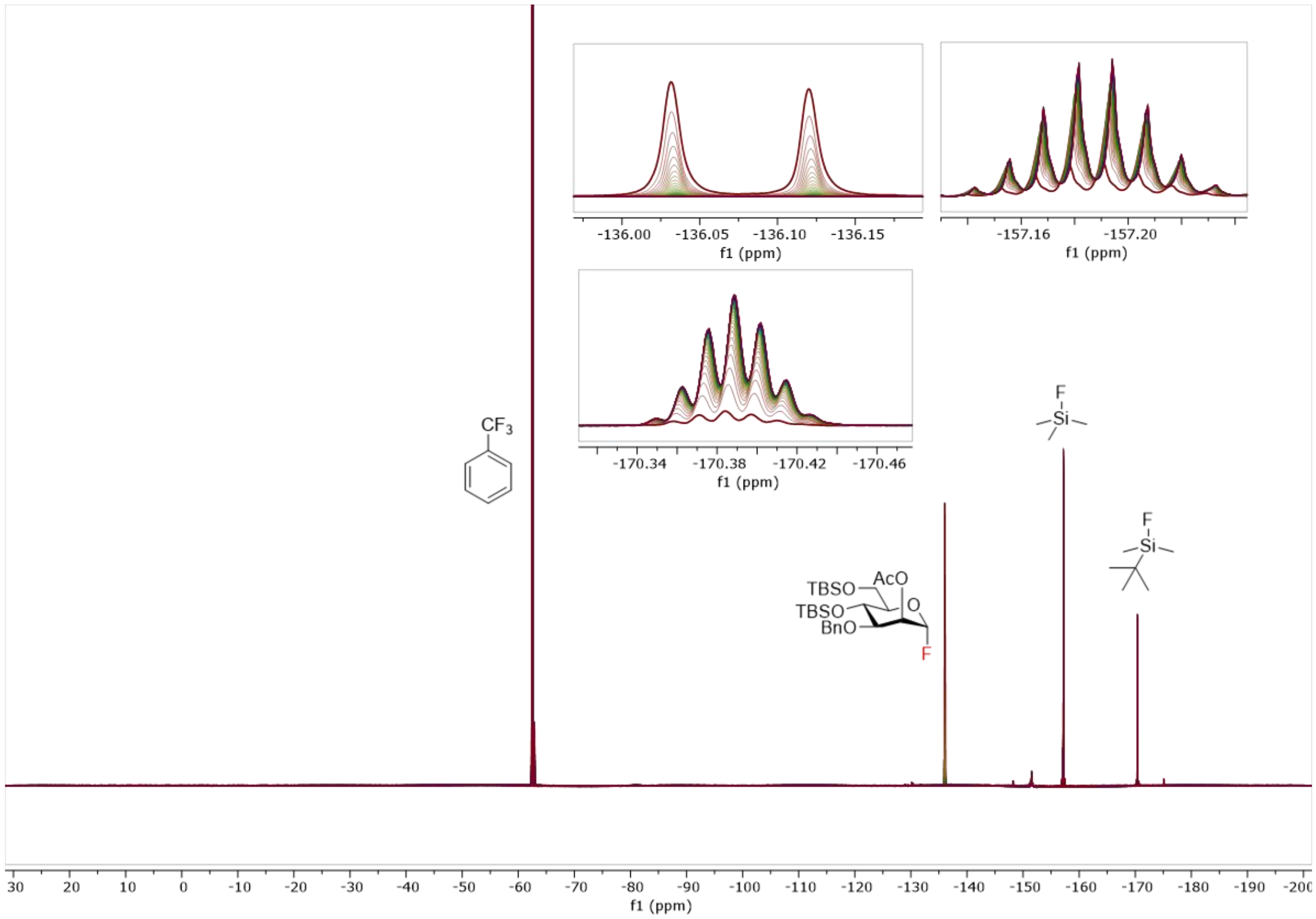

Figure S3. ^{19}F NMR spectra showing the reaction progression of compound (**8**) and (**9**) in 10 mol% BF_3•OEt_2

Methyl-2,3-di-*O*-benzyl-4-*O*-(2-*O*-acetyl-3,4,6-tri-*O*-benzyl-α-D-mannopyranosyl)-6-*O*-benzoyl-α-D-glucopyranoside (10)

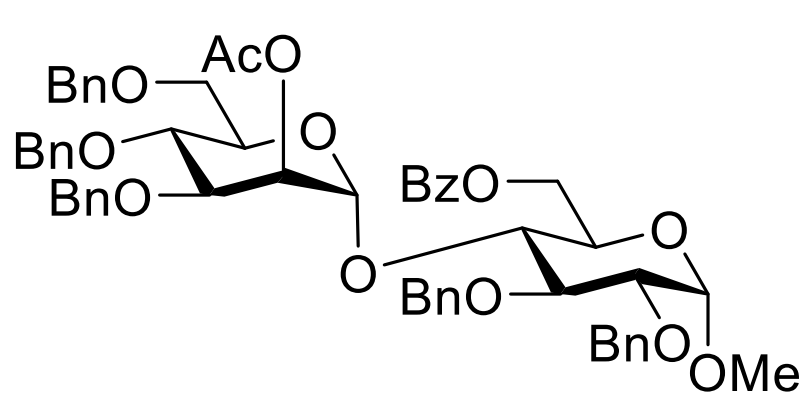

This product corresponds to the observed outcome for the ^{19}F NMR work described in Figure S1 between compounds **2** and **9**. For isolation and characterization purposes this compound was prepared by following general procedure B, with 2-*O*-acetyl-3,4,6-tri-*O*-benzyl-α-D-mannopyranosyl fluoride **2** (40.0 mg, 80.9 µmol) and methyl-2,3-di-O-benzyl-4-O-trimethylsilyl-

6-O-benzoyl-α-D-glucopyranoside (44.5 mg, 80.9 μmol) **9**, with BF_3•OEt_2 (1.15 mg, 1.02 μL, 8.09 μmol) in toluene (1.62 mL). Purification by silica gel column chromatography (5% to 20% EtOAc in hexanes) afforded the desired product **10** (58.1 mg, 61.0 μmol, 75%) as a colorless viscous residue. ^{1}H NMR (500 MHz, $CDCl_3$) δ 8.05 (d, *J* = 7.0 Hz, 2H), 7.56 (t, *J* = 6.8 Hz, 1H), 7.42 (t, *J* = 7.8 Hz, 2H), 7.36 – 7.20 (m, 23H), 7.14 – 7.07 (m, 2H), 5.52 – 5.46 (m, 2H), 5.04 (d, *J* = 11.0 Hz, 1H), 4.82 – 4.62 (m, 5H), 4.62 – 4.53 (m, 3H), 4.47 (d, *J* = 11.1 Hz, 1H), 4.42 (d, *J* = 11.1 Hz, 2H), 4.26 (d, *J* = 12.1 Hz, 1H), 3.97 (t, *J* = 8.6 Hz, 1H), 3.89 (dd, *J* = 14.1, 7.9 Hz, 4H), 3.80 (d, *J* = 8.4 Hz, 1H), 3.72 (dd, *J* = 10.8, 3.6 Hz, 1H), 3.56 (dd, *J* = 9.6, 3.6 Hz, 1H), 3.50 (d, *J* = 10.5 Hz, 1H), 3.38 (s, 3H), 1.98 (s, 3H). ^{13}C NMR (126 MHz, $CDCl_3$) δ 169.9, 166.2, 138.47, 138.46, 138.2, 138.1, 137.9, 133.2, 130.0, 129.9, 128.6, 128.5, 128.48, 128.40, 128.3, 128.25, 128.21, 128.1, 128.0, 127.9, 127.8, 127.7, 127.63, 127.61, 127.5, 99.8, 97.8, 81.7, 80.2, 78.2, 76.3, 75.5, 75.2, 73.9, 73.5, 73.4, 72.8, 71.9, 68.9, 68.4, 68.2, 63.8, 55.5, 21.0. Anomeric configurations confirmed by one bond ^{1}H-^{13}C J value analysis in the ^{1}H coupled ^{13}C NMR spectrum: ^{13}C NMR (126 MHz, $CDCl_3$) δ 99.8 (d, *J* = 173.3 Hz, α-mannose), 97.8 (d, *J* = 168.2 Hz, α-glucose). ESI-HRMS m/z: calc'd for $C_{57}H_{60}O_{13}$ $[M+NH_4]^+$: 970.4372, found: 970.4370

Methyl-2,3-di-*O*-benzyl-4-*O*-(2-*O*-acetyl-3-*O*-benzyl-4,6-*O*-di-*tert*-butylsilylene-α-D-mannopyranosyl)-6-*O*-benzoyl-α-D-glucopyranoside (11)

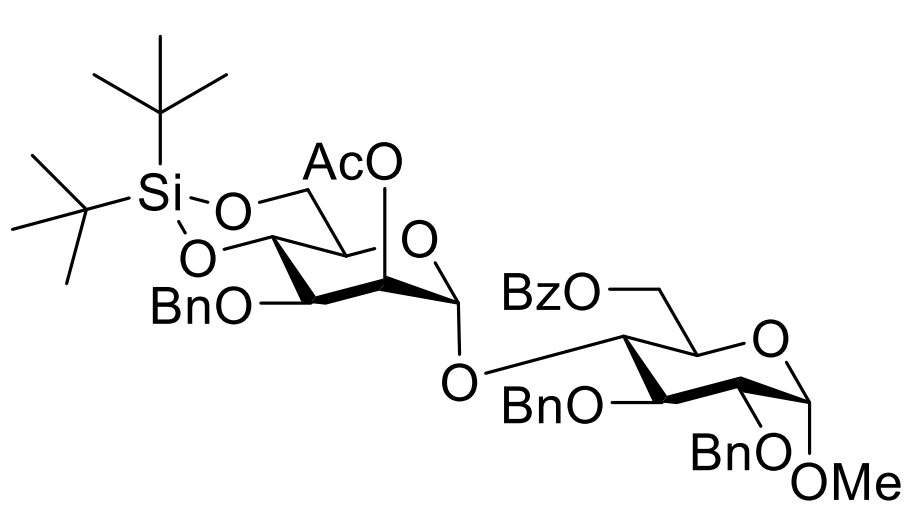

This product corresponds to the observed outcome for the ^{19}F NMR work described in Figure S2 of the main text between compounds **5** and **9**. For isolation and characterization purposes this

compound was prepared by following general procedure B, with methyl-2,3-di-O-benzyl-4-O-trimethylsilyl-6-O-benzoyl-α-D-glucopyranoside (55.1 mg, 100.0 μmol) **9** and 2-*O*-acetyl-3-*O*-benzyl-4,6-*O*-di-*tert*-butylsilylene-α-D-mannopyranosyl fluoride (45.5 mg, 100.0 μmol) **5** with BF_3•OEt_2 (1.42 mg, 1.27 μL, 10.0 μmol) in Toluene (2.00 mL). Purification by silica gel column chromatography (5% to 20% EtOAc in hexanes) afforded the desired product **11** (54 mg, 59.0 μmol, 59%) as a colorless viscous residue. ^{1}H NMR (500 MHz, $CDCl_3$) δ 8.03 (d, *J* = 6.9 Hz, 2H), 7.56 (t, *J* = 7.5 Hz, 1H), 7.44 (t, *J* = 7.8 Hz, 2H), 7.39 (d, *J* = 8.4 Hz, 2H), 7.35 – 7.22 (m, 13H), 5.45 (dd, *J* = 3.5, 1.6 Hz, 1H), 5.40 (d, *J* = 1.8 Hz, 1H), 5.03 (d, *J* = 10.9 Hz, 1H), 4.74 (d, *J* = 10.9 Hz, 2H), 4.73 – 4.69 (m, 3H), 4.58 (d, *J* = 7.1 Hz, 1H), 4.56 (d, *J* = 1.6 Hz, 1H), 4.28 (dd, *J* = 12.0, 4.6 Hz, 1H), 4.12 (t, *J* = 9.3 Hz, 1H), 4.01 – 3.94 (m, 2H), 3.92 (dd, *J* = 9.9, 4.8 Hz, 1H), 3.87 – 3.81 (m, 2H), 3.77 (td, *J* = 9.6, 4.4 Hz, 1H), 3.69 (dd, *J* = 9.4, 3.6 Hz, 1H), 3.53 (dd, *J* = 9.5, 3.5 Hz, 1H), 3.37 (s, 3H), 1.95 (s, 3H), 1.04 (s, 9H), 0.96 (s, 9H). ^{13}C NMR (126 MHz, $CDCl_3$) δ 169.8, 166.0, 138.9, 138.4, 137.9, 133.2, 129.9, 129.8, 128.6, 128.5, 128.4, 128.3, 128.2, 128.1, 127.6, 127.4, 127.3, 99.9, 97.8, 81.8, 80.3, 76.6, 75.4, 74.3, 73.4, 72.3, 69.9, 69.0, 68.2, 66.5, 63.2, 55.5, 34.8, 31.7, 27.5, 27.1, 25.4, 22.7, 21.0, 20.0, 14.2. Anomeric configurations confirmed by one bond ^{1}H-^{13}C J value analysis in the ^{1}H coupled ^{13}C NMR spectrum: ^{13}C NMR (126 MHz, $CDCl_3$) δ 99.8 (d, *J* = 172.8 Hz, α-mannose), 97.8 (d, *J* = 168.0 Hz, α-glucose). ESI-HRMS m/z: calc'd for $C_{51}H_{64}O_{13}Si$ $[M+NH_4]^+$: 930.4454, found: 930.4450

Methyl-2,3-di-*O*-benzyl-4-*O*-(2-*O*-acetyl-3-*O*-benzyl-4,6-*O*-di-*tert*-butyldimethylsilyl-α-D-mannopyranosyl)-6-*O*-benzoyl-α-D-glucopyranoside (12)

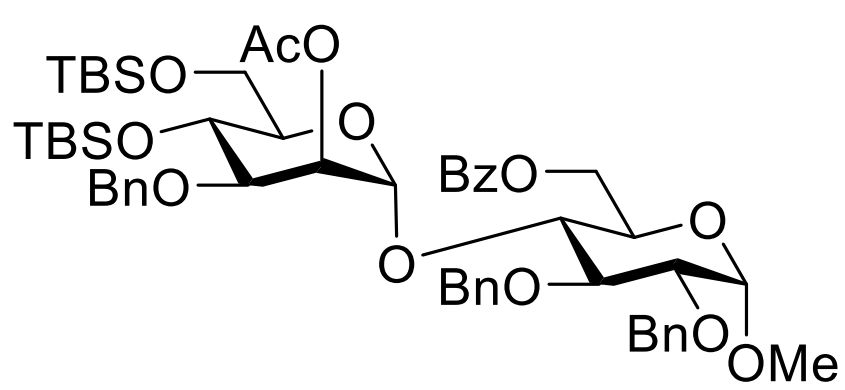

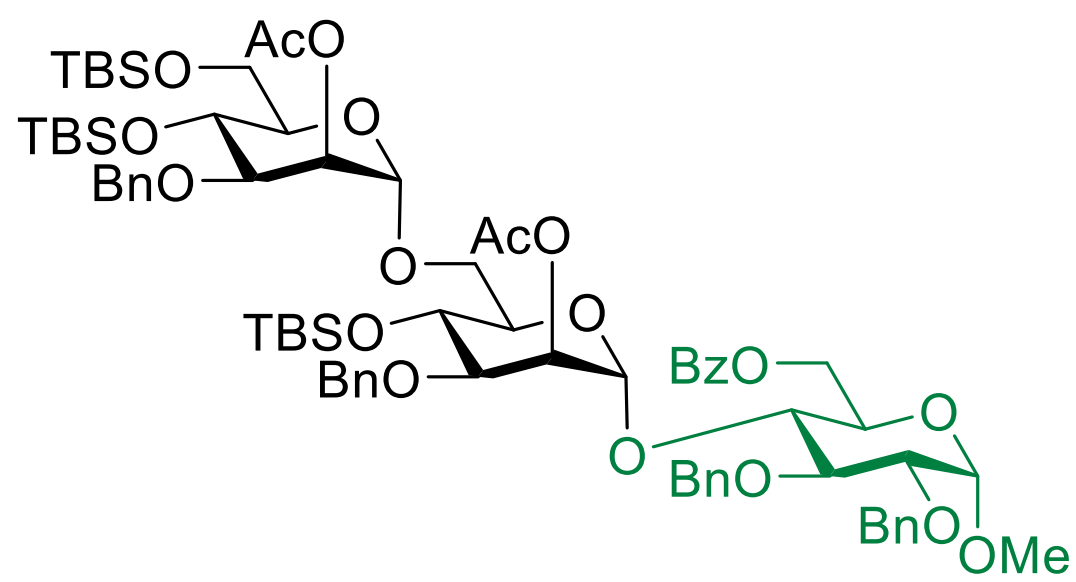

Product **12** was obtained as a mixture of di- and trisaccharides corresponding to the outcome described above in the ^{19}F NMR reaction progression experiment in Scheme 4B (manuscript) and Figure S3 (SI) between compounds **8** and **9**. The observed TMS-F and TBS-F byproducts visible in the ^{19}F NMR analysis illustrate that the mixture originates from competitive reaction of the TMS and TBS ethers.

III. Synthetic Procedures for Starting Materials.

Methyl-2,3-*O*-benzyl-4,6-*O*-di-ethylsilylene-α-D-glucopyranoside (1a)

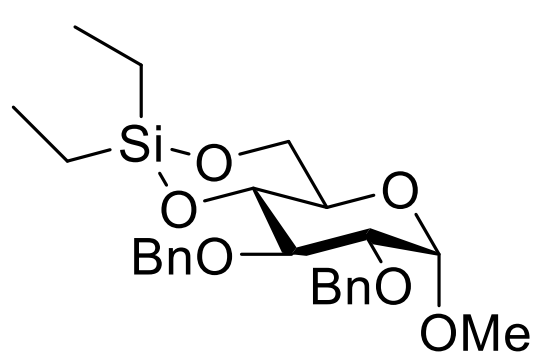

This compound was prepared as previously described.[1]

Methyl-2,3-*O*-benzyl-4,6-*O*-di-isopropylsilylene-α-D-glucopyranoside (1b)

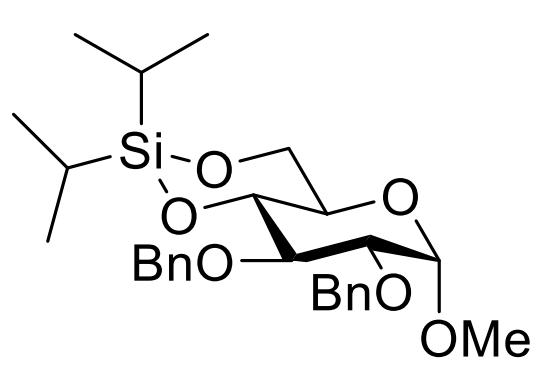

This compound was prepared as previously described.[1]

Methyl-2,3-*O*-benzyl-4,6-*O*-di-*tert*-butylsilylene-α-D-glucopyranoside (1c)

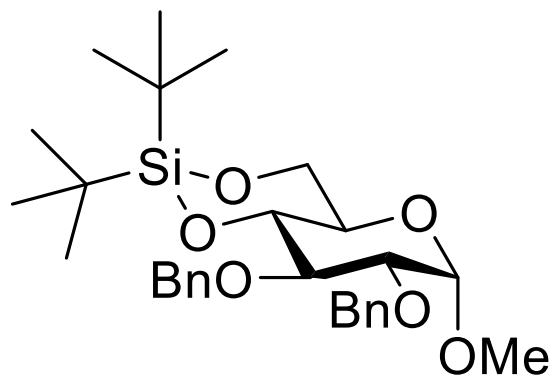

A stirred solution of methyl-4,6-*O*-di-*tert*-butylsilylene-α-D-glucopyranoside[2] (1.05 g, 3.14 mmol) in *N,N*-dimethylformamide (15.0 mL) was treated with sodium hydride (0.30 g, 7.53 mmol, 60% dispersion in mineral oil, 2.4 equiv.) at 0 °C under N_2. The mixture was stirred at 0 °C for 10 minutes then treated with benzyl bromide (1.12 mL, 1.61 g, 9.42 mmol, 3.0 equiv.) and the reaction mixture was allowed to warm to room temperature. After 16 h, the reaction mixture was diluted with ethyl acetate (100 mL) and washed with water (100 mL X 3) then saturated brine (100 mL). The organic layer was then dried over anhydrous Na_2SO_4, filtered, and concentrated under reduced pressure. The crude material was purified by silica gel column chromatography (5% to 20% EtOAc in hexanes, product elutes at 15% EtOAc in hexanes) to yield the desired product **1c** (1.35 g, 2.62 mmol, 83.5%) as a colorless oil. ^{1}H NMR (401 MHz, $CDCl_3$) δ 7.42 (d, *J* = 6.8 Hz, 2H), 7.39 – 7.27 (m, 8H), 4.97 (d, *J* = 11.1 Hz, 1H), 4.84 (d, *J* = 12.1 Hz, 1H), 4.83 (d, *J* = 11.0 Hz, 1H), 4.66 (d, *J* = 12.1 Hz, 1H), 4.54 (d, *J* = 3.8 Hz, 1H), 4.08 (dd, *J* = 10.0, 4.7 Hz, 1H), 3.88 – 3.79 (m, 3H), 3.77 – 3.71 (m, 1H), 3.46 (dd, *J* = 9.1, 3.8 Hz, 1H), 3.39 (s, 3H), 1.08 (s, 9H), 1.01 (s, 9H). ^{13}C NMR (101 MHz, $CDCl_3$) δ 139.2, 138.5, 128.5, 128.4, 128.2, 128.1, 127.9, 127.6, 99.0, 81.9, 78.8, 78.5, 75.9, 73.8, 66.9, 66.2, 55.6, 27.6, 27.2, 22.8, 20.0. ESI-HRMS m/z: Calcd. for $C_{29}H_{42}FO_6SiNH_4$ $[M+NH_4]^+$: 532.3088; found: 532.3083.

2-*O*-Acetyl-3,4,6-tri-*O*-benzyl-α-D-mannopyranosyl fluoride (2)

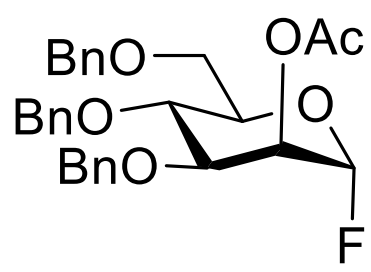

This compound was prepared as previously described.[3]

2-*O*-acetyl-3-*O*-benzyl-4,6-*O*-di-*tert*-butylsilylene-α-D-mannopyranosyl fluoride (5)

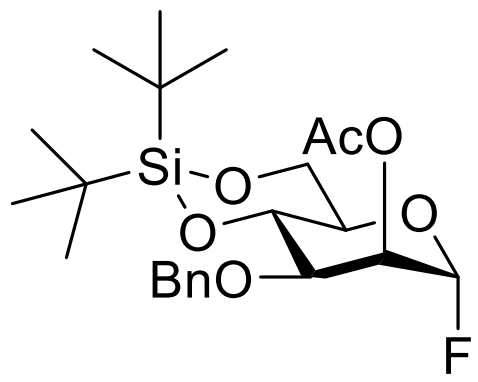

This compound was prepared as previously described.[4]

Methyl-2,4,6-tri-*O*-benzyl-3-*O*-trimethylsilyl-α-D-glucopyranoside (6)

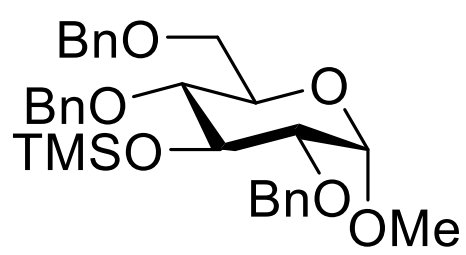

This compound was prepared as previously described.[4]

2-*O*-acetyl-3-*O*-benzyl-4,6-di-*O*-*tert*-butyldimethylsilyl-α-D-mannopyranosyl fluoride (8)

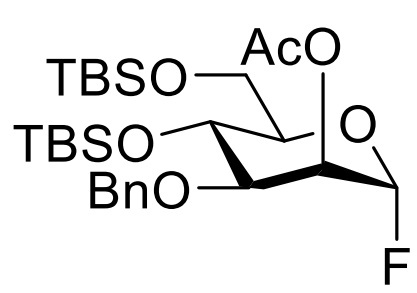

To a stirred solution of 2-*O*-acetyl-3-*O*-benzyl-α-D-mannopyranosyl fluoride[4] (118 mg, 0.375 mmol) in *N,N*-dimethylformamide (3.75 mL) was treated with imidazole (105 mg, 1.54 mmol, 4.1 equiv.) followed by *tert*-butyldimethylchlorosilane (119 mg, 0.788 mmol, 2.1 equiv.) at rt under N_2. After 16 h the reaction mixture was diluted with ethyl acetate (50 mL) and washed with water (50 mL X 2) and brine (50 mL). The organic layer was dried over anhydrous Na_2SO_4, filtered, and concentrated under reduced pressure. The crude was purified b silica gel column chromatography (5% to 20% EtOAc in hexanes) to yield the desired product **8** (152 mg, 0.280 mmol, 75%) as a colorless oil. ^{1}H NMR (500 MHz, C_6D_6) δ 7.30 (d, *J* = 7.0 Hz, 2H), 7.19 (t, *J* = 7.6 Hz, 2H), 7.09 (t, *J* = 7.4 Hz, 1H), 5.57 – 5.52 (m, 1H), 5.39 (dd, *J* = 49.8, 2.1 Hz, 1H), 4.49 (d, *J* = 10.9 Hz, 1H),

4.31 (t, J = 9.4 Hz, 1H), 4.11 (d, J = 10.9 Hz, 1H), 3.94 (dd, J = 11.8, 2.6 Hz, 1H), 3.76 (d, J = 9.5 Hz, 1H), 3.71 (dt, J = 9.5, 2.8 Hz, 1H), 3.67 (dd, J = 11.8, 1.8 Hz, 1H), 1.72 (s, 3H), 1.03 (s, 9H), 0.97 (s, 9H), 0.19 (s, 3H), 0.13 (s, 3H), 0.13 (s, 3H), 0.11 (s, 3H). ^{13}C NMR (126 MHz, C_6D_6) δ 169.2, 138.2, 128.5, 128.35, 128.33, 106.2 (d, J = 219.1 Hz), 77.8 (d, J = 2.2 Hz), 76.5 (d, J = 1.9 Hz), 71.3, 66.5 (d, J = 40.1 Hz), 66.1, 61.4, 26.2, 26.1, 20.2, 18.6, 18.5, 7.2, 5.5, -3.6, -4.8, -4.9, -5.1. ^{19}F NMR (471 MHz, C_6D_6) δ -135.95 (d, J = 49.9 Hz). ESI-HRMS m/z: Calcd. for $C_{27}H_{47}FO_6Si_2Na$ [M +Na]$^+$: 565.2787; found: 565.2784.

Methyl-2,3-di-O-benzyl-4-O-trimethylsilyl-6-O-benzoyl-α-D-glucopyranoside (9)

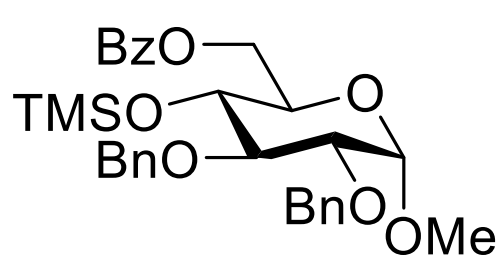

This compound was prepared by following general procedure A, using methyl-2,3-di-*O*-benzyl-6-*O*-benzoyl-α-D-glucopyranoside[5] (2.98 g, 6.23 mmol), chlorotrimethylsilane (1.01 g, 1.19 mL, 9.34 mmol), and triethylamine (2.60 mL, 1.89 g, 18.7 mmol) in DCM (31.1 mL). Purification by silica gel column chromatography (5% to 20% EtOAc in hexanes, product elutes at 8% EtOAc in hexanes) to yield the desired product **9** (3.10 g, 5.60 mmol, 90%) as a white amorphous solid. ^{1}H NMR (500 MHz, $CDCl_3$) δ 7.99 (d, J = 7.0 Hz, 2H), 7.53 – 7.48 (m, 1H), 7.38 (t, J = 7.8 Hz, 2H), 7.33 – 7.18 (m, 10H), 4.97 (d, J = 11.1 Hz, 1H), 4.72 (d, J = 11.1 Hz, 1H), 4.68 (d, J = 12.0 Hz, 1H), 4.57 – 4.51 (m, 3H), 4.33 (dd, J = 11.9, 5.1 Hz, 1H), 3.85 (ddd, J = 9.9, 5.1, 2.2 Hz, 1H), 3.76 (t, J = 9.1 Hz, 1H), 3.70 – 3.64 (m, 1H), 3.49 (dd, J = 9.5, 3.5 Hz, 1H), 3.33 (s, 3H), 0.05 (s, 9H). ^{13}C NMR (126 MHz, $CDCl_3$) δ 166.4, 139.0, 138.1, 133.1, 130.0, 129.7, 128.57, 128.53, 128.3, 128.2, 128.0, 127.5, 127.4, 98.0, 81.9, 80.4, 75.6, 73.4, 71.3, 69.9, 63.8, 55.2, 0.6. ESI-HRMS m/z: Calcd. for $C_{31}H_{38}O_7SiNa$ [M +Na]$^+$: 553.2278; found: 553.2279.

Synthesis of 2-*O*-benzoyl-3-*O*-trimethylsilyl-4,6-*O*-di-*tert*-butylsilylene-α-D-glucopyranosyl fluoride (S6), 2-*O*-acetyl-3-*O*-trimethylsilyl-4,6-*O*-di-*tert*-butylsilylene-α-D-glucopyranosyl fluoride (23), and 2-*O*-trimethylsilyl-3-*O*-acetyl-4,6-*O*-di-*tert*-butylsilylene-α-D-glucopyranosyl fluoride (S7)

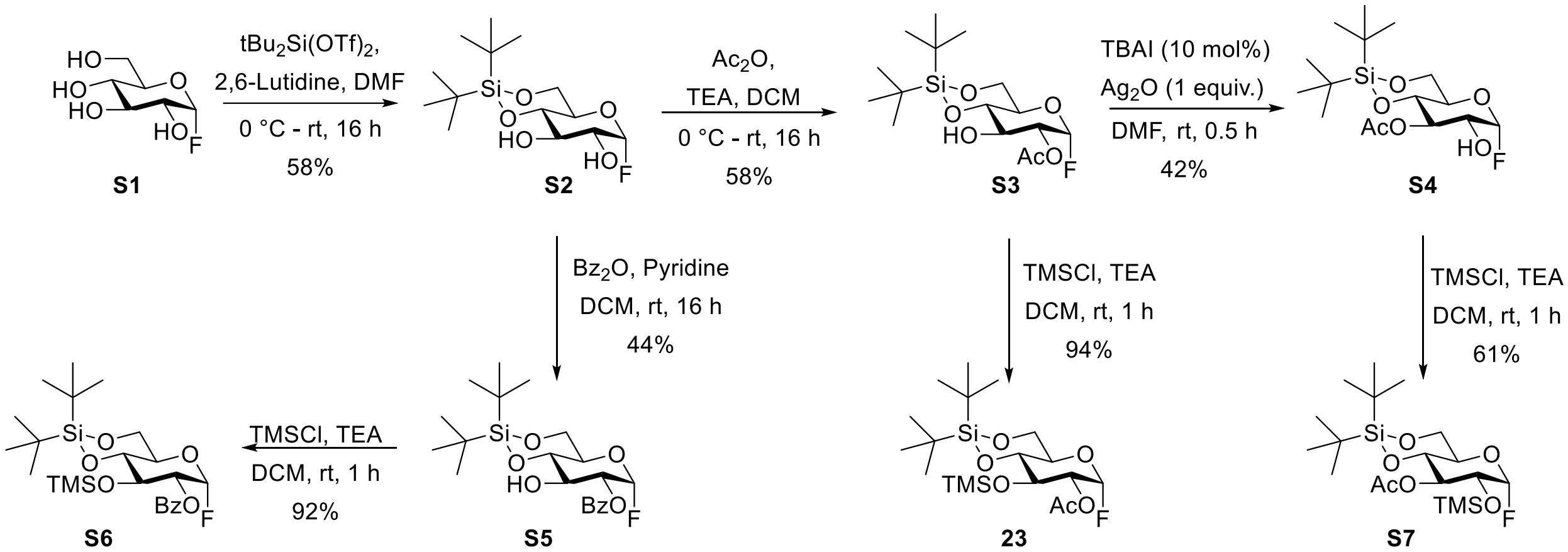

4,6-*O*-di-*tert*-butylsilylene-α-D-glucopyranosyl fluoride (S2)

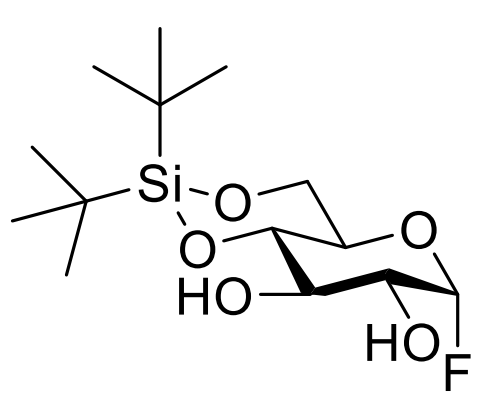

To a stirred solution of α-D-glucopyranosyl fluoride[4] (1.99 g, 10.9 mmol) in *N,N*-dimethylformamide (109 mL) was treated with 2,6-lutidine (3.51 g, 3.80 mL, 32.8 mmol) at 0 °C under N_2. Reaction mixture was then treated di-tert-butyl bis(trifluoromethanesulfonate) (5.29 g, 4.03 mL, 12.0 mmol) at 0 °C and the reaction stirred while warming to rt for 16 h. the reaction mixture was diluted with EtOAc (150 mL) and washed with sat. $NaHCO_3$ (100 mL), water (100 mL X 3) and brine (50 mL). The organic layer was dried over anhydrous Na_2SO_4, filtered, and concentrated under reduced pressure. The crude was purified by silica gel column chromatography (0% to 30% EtOAc in hexanes) to afford the product **S2** (2.04 g, 6.34 mmol, 58%) as a viscous oil. 1H NMR (400 MHz, CDCl3) δ 5.58 (dd, J = 53.1, 2.7 Hz, 1H), 4.16 (dd, J = 8.7,

3.7 Hz, 1H), 3.94 – 3.76 (m, 3H), 3.69 (t, J = 8.9 Hz, 1H), 2.83 (br s, 1H), 2.41 (br s, 1H), 1.04 (s, 9H), 0.98 (s, 9H).13C NMR (176 MHz, CDCl3) δ 106.7 (d, J = 226.6 Hz), 76.3, 74.0, 72.0 (d, J = 24.8 Hz), 68.4 (d, J = 3.1 Hz), 66.2, 27.6, 27.1, 22.8, 20.1. ^{19}F NMR (377 MHz, $CDCl_3$) δ -149.71 (dd, *J* = 53.1, 25.0 Hz). ESI-HRMS m/z: Calcd. for $C_{14}H_{27}FO_5SiNa$ $[M+Na]^+$: 345.1503; found: 345.1503.

2-*O*-acetyl-4,6-*O*-di-*tert*-butylsilylene-α-D-glucopyranosyl fluoride (S3)

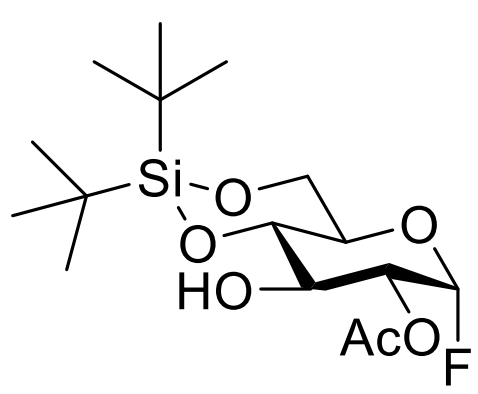

To a stirred solution of 4,6-*O*-di-*tert*-butylsilylene-α-D-glucopyranosyl fluoride **S2** (673 mg, 2.09 mmol) in DCM (10.4 mL) is treated with triethylamine (1.90 g, 1.94 mL, 18.8 mmol) at 0 °C under N_2. The reaction mixture was then treated with acetic anhydride (256 mg, 236 μL, 2.50 mmol) and allowed to stir for 16 h. Methanol (2 mL) was then added and the reaction mixture concentrated under reduced pressure. Crude material dissolved in EtOAc (30 mL) and washed with sat. $NaHCO_3$ (30 mL), water (30 mL), and brine (30 mL). Organic layer dried over anhydrous Na_2SO_4, filtered, and concentrated under reduced pressure. Crude purified by silica gel column chromatography (0 to 20% EtOAc in Hexanes) to afford the desired product **S3** (445 mg, 1.22 mmol, 58%) as a viscous residue. 1H NMR (400 MHz, CDCl3) δ 5.58 (dd, J = 53.1, 2.7 Hz, 1H), 4.16 (dd, J = 8.7, 3.7 Hz, 1H), 3.94 – 3.76 (m, 3H), 3.69 (t, J = 8.9 Hz, 1H), 3.00 – 2.77 (m, 1H), 2.40 (s, 3H), 1.04 (s, 9H), 0.98 (s, 9H). 13C NMR (126 MHz, CDCl3) δ 170.62, 104.29 (d, *J* = 228.3 Hz), 76.63, 72.33 (d, *J* = 24.2 Hz), 71.20, 68.09 (d, *J* = 3.5 Hz), 66.09, 27.54, 27.06, 22.86, 20.94, 20.10. ^{19}F NMR (377 MHz, $cdcl_3$) δ -147.51 (dd, *J* = 53.3, 24.7 Hz). ESI-HRMS m/z: Calcd. for $C_{16}H_{29}FO_6SiH$ $[M+H]^+$: 365.1790; found: 365.1795.

3-*O*-acetyl-4,6-*O*-di-*tert*-butylsilylene-α-D-glucopyranosyl fluoride (S4)

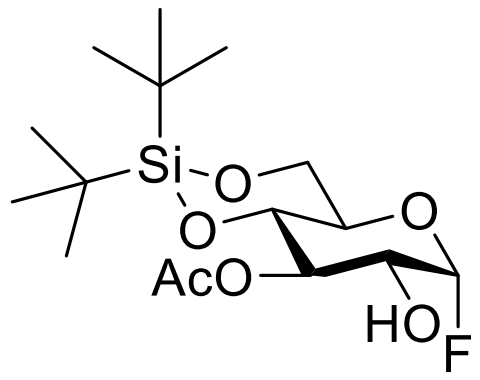

To a stirred solution of 2-*O*-acetyl-4,6-*O*-di-*tert*-butylsilylene-α-D-glucopyranosyl fluoride **S3** (373 mg, 1.02 mmol) in *N,N*-dimethylformamide (20 mL) was added tetrabutylammonium iodide (37.8 mg, 0.102 mmol) and silver (I) oxide (237 mg, 1.02 mmol) at rt under N_2. The reaction mixture was stirred at rt for 48 hours then filtered and concentrated under reduced pressure. The crude was purified by silica gel column chromatography (0% to 20% EtOAc in Hexanes) to afford the product **S4** (141 mg, 0.387 mmol, 42%) as clear residue. ^{1}H NMR (500 MHz, $CDCl_3$) δ 5.59 (dd, *J* = 52.9, 2.9 Hz, 1H), 5.15 (t, *J* = 9.6 Hz, 1H), 4.19 (dd, *J* = 10.0, 4.8 Hz, 1H), 3.96 (td, *J* = 10.0, 4.8 Hz, 1H), 3.90 – 3.81 (m, 2H), 3.64 (ddd, *J* = 25.1, 9.9, 2.9 Hz, 1H), 2.15 (s, 3H), 1.03 (s, 9H), 0.97 (s, 9H). ^{13}C NMR (126 MHz, $CDCl_3$) δ 171.7, 107.1 (d, *J* = 225.7 Hz), 74.7, 73.9, 71.3 (d, *J* = 25.9 Hz), 68.8 (d, *J* = 3.4 Hz), 66.2, 27.4, 26.9, 22.8, 20.9, 20.0. ^{19}F NMR (471 MHz, $CDCl_3$) δ -150.71 (dd, *J* = 52.8, 25.1 Hz). ESI-HRMS m/z: Calcd. for $C_{16}H_{29}FO_6SiH$ $[M+H]^+$: 365.1790; found: 365.1797.

2-*O*-benzoyl-4,6-*O*-di-*tert*-butylsilylene-α-D-glucopyranosyl fluoride (S5)

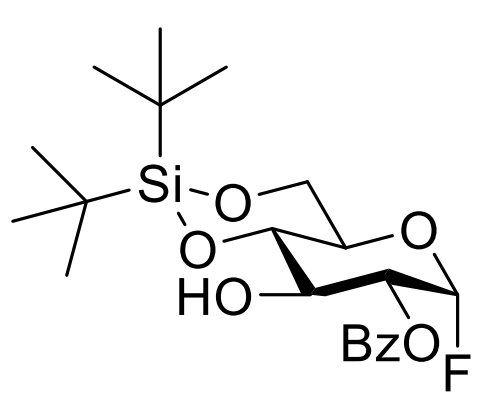

To a stirred solution of 4,6-*O*-di-*tert*-butylsilylene-α-D-glucopyranosyl fluoride **S2** (397 mg, 1.23 mmol) in DCM (6.16 mL) was added pyridine (195 mg, 199 µL, 2.46 mmol) and then benzoic anhydride (279 mg, 1.23 mmol) at rt under N_2. After stirring for 16 h and warming to rt the reaction

mixture was quenched with MeOH (1 mL) and concentrated under reduced pressure. The resulting crude was then dissolved in EtOAc (20 mL) and washed with $NaHCO_3$ (20 mL) followed by water (20 mL) and brine (20 mL). The organic layer was dried over anhydrous Na_2SO_4, filtered, and concentrated under reduced pressure. The crude was purified by silica gel column chromatography (0% to 30% EtOAc in Hexanes) to afford the product **S5** (173 mg, 0.537 mmol, 44%) as a white amorphous solid. 1H NMR (700 MHz, CDCl3) δ 8.11 (d, J = 7.7 Hz, 2H), 7.60 (t, J = 7.4 Hz, 1H), 7.46 (t, J = 7.0 Hz, 2H), 5.78 (dd, J = 53.2, 2.9 Hz, 1H), 5.08 (ddd, J = 24.6, 9.9, 3.0 Hz, 1H), 4.22 (dd, J = 9.8, 4.9 Hz, 1H), 4.16 (t, J = 9.4 Hz, 1H), 3.99 (td, J = 10.1, 4.9 Hz, 1H), 3.92 (td, J = 10.1, 1.2 Hz, 1H), 3.87 (t, J = 9.4 Hz, 1H), 2.72 (br s, 1H), 1.08 (s, 9H), 1.02 (s, 9H). 13C NMR (176 MHz, CDCl3) δ 165.9, 133.5, 130.0, 129.2, 128.4, 104.3 (d, *J* = 228.7 Hz), 76.6, 72.5 (d, *J* = 24.2 Hz), 71.2, 68.0 (d, *J* = 3.5 Hz), 66.0, 27.4, 26.9, 22.7, 20.0. ^{19}F NMR (377 MHz, $cdcl_3$) δ -147.17 (dd, *J* = 53.3, 24.7 Hz). ESI-HRMS m/z: Calcd. for $C_{21}H_{31}FO_6SiNa$ [M +Na]$^+$: 449.1772; found: 449.1765.

2-*O*-benzoyl-3-*O*-trimethylsilyl-4,6-*O*-di-*tert*-butylsilylene-α-D-glucopyranosyl fluoride (S6)

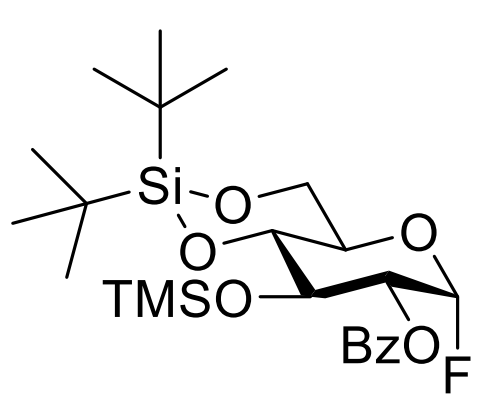

This compound was prepared by following general procedure A, using 3-*O*-acetyl-4,6-*O*-di-*tert*-butylsilylene-α-D-glucopyranosyl fluoride **S5** (105 mg, 0.246 mmol), chlorotrimethylsilane (80.2 mg, 93 µL, 0.738 mmol), and triethylamine (172 µL, 125 mg, 1.23 mmol) in DCM (1.23 mL). Purification by silica gel column chromatography (5% to 15% EtOAc in hexanes) to yield the desired product **S6** (120 mg, 226 µmol, 92%) as a colorless viscous residue. ^{1}H NMR (500 MHz, $CDCl_3$) δ 8.13 – 8.07 (m, 3H), 7.60 (tt, *J* = 7.1, 1.3 Hz, 1H), 7.51 – 7.43 (m, 2H), 5.72 (dd, *J* =

53.7, 2.9 Hz, 1H), 5.06 (ddd, *J* = 25.6, 9.5, 2.9 Hz, 1H), 4.21 (dd, *J* = 9.5, 4.5 Hz, 1H), 4.13 – 4.06 (m, 1H), 4.03 – 3.83 (m, 4H), 1.08 (s, 7H), 1.03 (s, 7H), 0.08 (s, 9H). ^{13}C NMR (126 MHz, $CDCl_3$) δ 165.2, 133.0, 129.4, 128.8, 128.0, 104.3 (d, *J* = 227.5 Hz), 72.8 (d, *J* = 24.3 Hz), 71.8, 68.1 (d, *J* = 3.7 Hz), 65.8, 27.0, 26.5, 22.3, 19.5, 0.0. ^{19}F NMR (377 MHz, $CDCl_3$) δ -147.24 (dd, *J* = 53.7, 25.7 Hz). ESI-HRMS m/z: Calcd. for $C_{24}H_{39}O_6FSi_2Na$ $[M + Na]^+$: 521.2161; found: 521.2170.

2-*O*-acetyl-3-*O*-trimethylsilyl-4,6-*O*-di-*tert*-butylsilylene-α-D-glucopyranosyl fluoride (23)

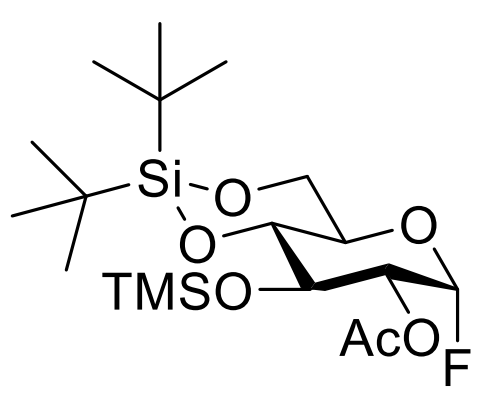

This compound was prepared by following general procedure A, using 2-*O*-acetyl-4,6-*O*-di-*tert*-butylsilylene-α-D-glucopyranosyl fluoride **S3** (225 mg, 0.617 mmol), chlorotrimethylsilane (201 mg, 235 μL, 1.85 mmol), and triethylamine (430 μL, 312 mg, 309 mmol) in DCM (3.09 mL). Purification by silica gel column chromatography (5% to 15% EtOAc in hexanes) to yield the desired product **23** (254 mg, 582 μmol, 94%) as a colorless viscous residue. ^{1}H NMR (500 MHz, $CDCl_3$) δ 5.47 (dd, *J* = 53.7, 2.8 Hz, 1H), 4.56 (ddd, *J* = 25.7, 9.5, 2.8 Hz, 1H), 4.05 – 3.98 (m, 1H), 3.80 – 3.66 (m, 3H), 3.63 (t, *J* = 8.9 Hz, 1H), 1.98 (s, 3H), 0.90 (s, 9H), 0.85 (s, 9H), 0.00 (s, 9H). ^{13}C NMR (126 MHz, $CDCl_3$) δ 169.7, 104.1 (d, *J* = 227.0 Hz), 72.6 (d, *J* = 24.4 Hz), 71.4, 68.1 (d, *J* = 3.7 Hz), 65.8, 27.0, 26.5, 22.3, 20.3, 19.5, 0.0. ^{19}F NMR (564 MHz, $CDCl_3$) δ -147.83 (dd, *J* = 53.7, 25.7 Hz). ESI-HRMS m/z: Calcd. for $C_{19}H_{37}FO_6Si_2Na$ $[M+Na]^+$: 459.2004; found: 459.2021.

2-*O*-trimethylsilyl-3-*O*-acetyl-4,6-*O*-di-*tert*-butylsilylene-α-D-glucopyranosyl fluoride (S7)

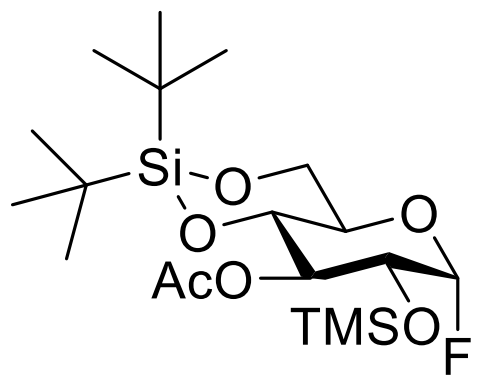

This compound was prepared by following general procedure A, using 3-*O*-acetyl-4,6-*O*-di-*tert*-butylsilylene-α-D-glucopyranosyl fluoride **S4** (141 mg, 0.387 mmol), chlorotrimethylsilane (210mg, 245 µL, 1.93 mmol), and triethylamine (377 µL, 274 mg, 2.71 mmol) in DCM (2.0 mL). Purification by silica gel column chromatography (5% to 15% EtOAc in hexanes) to yield the desired product **S7** (103 mg, 236 µmol, 61%) as a colorless viscous residue. ^{1}H NMR (500 MHz, $CDCl_3$) δ 5.40 (dd, *J* = 52.5, 2.9 Hz, 1H), 5.27 (t, *J* = 9.5 Hz, 1H), 4.17 (dd, *J* = 9.9, 4.9 Hz, 1H), 3.99 (td, *J* = 10.1, 4.9 Hz, 1H), 3.84 (td, *J* = 10.2, 1.6 Hz, 1H), 3.78 (t, *J* = 9.6 Hz, 1H), 3.70 (ddd, *J* = 24.4, 9.6, 2.9 Hz, 1H), 2.10 (s, 3H), 1.02 (s, 9H), 0.96 (s, 9H), 0.14 (s, 9H). ^{13}C NMR (126 MHz, $CDCl_3$) δ 169.7, 106.9 (d, *J* = 231.8 Hz), 74.7, 73.8, 71.0 (d, *J* = 24.7 Hz), 68.5 (d, *J* = 3.8 Hz), 66.3, 27.4, 26.9, 22.8, 21.0, 20.1, 0.1. ^{19}F NMR (471 MHz, $CDCl_3$) δ -149.47 (dd, *J* = 52.3, 24.3 Hz). ESI-HRMS m/z: Calcd. for $C_{19}H_{37}FO_6Si_2Na$ $[M+Na]^+$: 459.2004; found: 459.2017.

4,6-*O*-di-*tert*-butylsilylene-α-D-mannopyranosyl fluoride (S8)

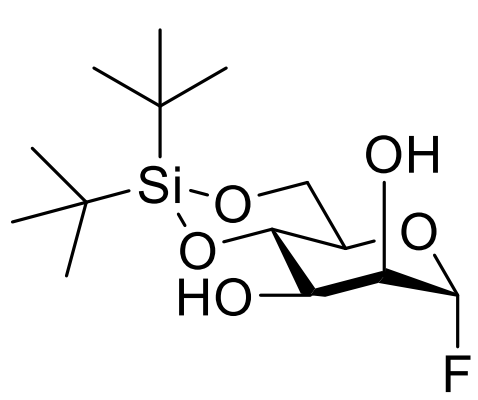

This compound was prepared as previously described.[4]

2-*O*-benzoyl-3-*O*-trimethylsilyl-4,6-*O*-di-*tert*-butylsilylene-α-D-mannopyranosyl fluoride (S9)

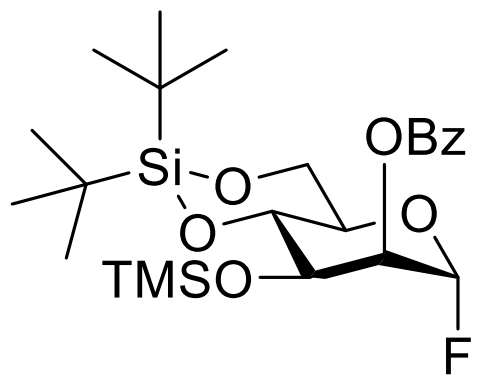

To a stirred solution of 4,6-*O*-di-*tert*-butylsilylene-α-D-mannopyranosyl fluoride **S8** (100 mg, 310 μmol) and 4 Å MS in DCM (2.0 mL) was added benzoyl cyanide (44.7 mg, 341 μmol) and N,N-dimethylaminopyridine (3.79 mg, 31.0 μmol) at -78 °C under N_2. The reaction was stirred while warming to rt for 16 h. The reaction mixture was then quenched with addition of 0.1 mL NH_4Cl and 0.1 mL methanol, diluted with 100 mL DCM, and filtered through a pad of celite. The filtrate was further washed with 100 mL NH_4Cl, dried over anhydrous Na_2SO_4, filtered, and concentrated under reduced pressure. The material was then dissolved in DCM (1 mL) and treated with hexamethyldisilazane (99.9 mg, 129 µL, 619 µmol) and iodine (7.85 mg, 30.9 µmol). The reaction stirred for 1 h and then quenched with $Na_2S_2O_3$ (3 g) with vigorous stirring. After stirring for an additional 0.5 h the reaction mixture was filtered, and the filtrate concentrated under reduced pressure. The crude was subjected to silica gel column chromatography (0% to 20% EtOAc in Hexanes) to afford the desired product **S9** (107 mg, 215 μmol, 69% over 2 steps) as a colorless residue. ^{1}H NMR (500MHz, CDCl3) δ 8.03 (d, J = 7.8 Hz, 2H), 7.59 (t, J = 7.3 Hz, 1H), 7.47 (t, J = 7.6 Hz, 2H), 5.58 (dd, J = 49.0, 1.8 Hz, 1H), 5.45 (d, J = 2.5 Hz, 1H), 4.21 (dd, J = 9.3, 4.2 Hz, 1H), 4.15 (t, J = 9.1 Hz, 1H), 4.08 – 3.91 (m, 3H), 1.08 (s, 9H), 1.02 (s, 9H), 0.12 (s, 9H). ^{13}C NMR (126 MHz, CDCl3) δ 165.7, 133.4, 130.0, 129.8, 128.6, 106.1 (d, J = 221.1 Hz), 74.3, 71.2 (d, J = 41.0 Hz), 70.1 (d, J = 2.4 Hz), 69.7 (d, J = 2.7 Hz), 66.5, 27.6, 27.2, 23.0, 20.1, 0.2. ^{19}F NMR (377 MHz, $CDCl_3$) δ -137.72 (d, J = 49.1 Hz). ESI-HRMS m/z: Calcd. for $C_{24}H_{39}FO_6Si_2$H-TMS $[M+H-TMS]^+$: 427.1946; found: 427.1948.

2-*O*-trimethylsilyl-3-*O*-benzyl-4,6-*O*-di-*tert*-butylsilylene-α-D-mannopyranosyl fluoride (S10)

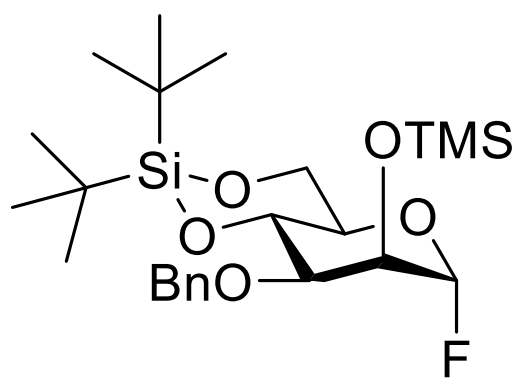

To a stirred solution of 4,6-*O*-di-*tert*-butylsilylene-α-D-mannopyranosyl fluoride **S8** (200 mg, 620 µmol) in acetonitrile (3 mL) was added tetrabutylammonium bromide (100 mg, 310 µmol), dibutyltin oxide (30.9 mg, 124 µmol), N,N-diisopropylethylamine (96.2 mg, 130 µL, 744 µmol), and benzyl bromide (212 mg, 148 µL, 1.24 mmol) at rt under N_2. The reaction mixture was heated to 60 °C for 12 h then filtered through celite and concentrated under reduced pressure. The material was then dissolved in DCM (3.10 mL) and treated with hexamethyldisilazane (150 mg, 194µL, 931 µmol) and iodine (7.87 mg, 31.0 µmol). The reaction stirred for 1 h and then quenched with $Na_2S_2O_3$ (3 g) with vigorous stirring. After stirring for an additional 0.5 h the reaction mixture was filtered, and the filtrate concentrated under reduced pressure. The crude was subjected to silica gel column chromatography (0% to 20% EtOAc in Hexanes) to afford the desired product **S10** (292 mg, 602 µmol, 97% over 2 steps) as a colorless viscous residue. ^{1}H NMR (401 MHz, $CDCl_3$) δ 7.42 – 7.27 (m, 5H), 5.34 (d, *J* = 50.6, 2.0 Hz, 1H), 4.93 (d, *J* = 11.8 Hz, 1H), 4.77 (d, *J* = 11.7 Hz, 1H), 4.34 (t, *J* = 9.5 Hz, 1H), 4.15 (dd, *J* = 9.9, 4.9 Hz, 1H), 4.07 (d, *J* = 2.4 Hz, 1H), 3.98 (t, *J* = 10.4 Hz, 1H), 3.83 (td, *J* = 10.0, 4.8 Hz, 1H), 3.60 (dd, *J* = 9.6, 2.9 Hz, 1H), 1.09 (s, 9H), 1.01 (s, 9H), 0.11 (s, 9H). ^{13}C NMR (126 MHz, $CDCl_3$) δ 138.8, 128.3, 128.0, 127.6, 108.7 (d, *J* = 222.9 Hz), 74.3, 74.0, 70.3 (d, *J* = 37.5 Hz), 70.2 (d, *J* = 2.3 Hz), 66.5, 27.6, 27.2, 22.8, 20.1, 0.4. ^{19}F NMR (377 MHz, $CDCl_3$) δ -136.12 (d, *J* = 50.4 Hz). ESI-HRMS m/z: Calcd. for $C_{24}H_{41}FO_5Si_2NH_4$ $[M+NH_4]^+$: 502.2814; found: 502.2810.

2,3-*O*-di-trimethylsilyl-4,6-*O*-di-*tert*-butylsilylene-α-D-mannopyranosyl fluoride (S11)

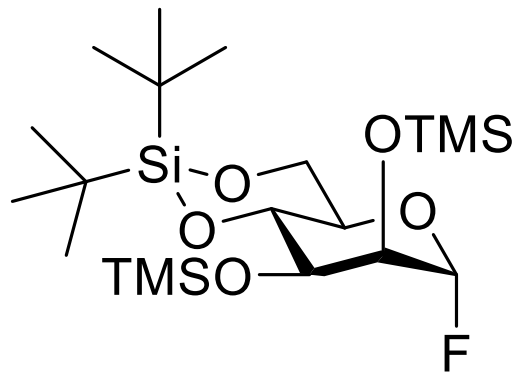

This compound was prepared by following general procedure A, using 4,6-*O*-di-*tert*-butylsilylene-α-D-mannopyranosyl fluoride **S8** (300 mg, 930 μmol), chlorotrimethylsilane (303 mg, 354 μL, 2.79 mmol), and triethylamine (389 μL, 282 mg, 2.79 mmol) in DCM (10.0 mL). Purification by silica gel column chromatography (5% to 15% EtOAc in hexanes) to yield the desired product **S11** (101 mg, 216 μmol, 23%) as a colorless residue. ^{1}H NMR (500 MHz, $CDCl_3$) δ 5.35 (dd, *J* = 50.7, 2.1 Hz, 1H), 4.14 (dd, *J* = 10.0, 5.0 Hz, 1H), 4.09 (t, *J* = 9.4 Hz, 1H), 3.94 (td, *J* = 10.2, 1.1 Hz, 2H), 3.91 (t, *J* = 2.6 Hz, 1H), 3.84 – 3.75 (m, 2H), 1.06 (s, 9H), 1.00 (s, 9H), 0.18 (s, 9H), 0.14 (s, 9H). ^{13}C NMR (126 MHz, $CDCl_3$) δ 108.8 (d, *J* = 222.4 Hz), 73.5, 72.1 (d, *J* = 37.1 Hz), 71.8 (d, *J* = 2.9 Hz), 70.4 (d, *J* = 2.2 Hz), 66.5, 27.7, 27.1, 22.8, 20.0, 0.7, 0.6. ^{19}F NMR (471 MHz, $CDCl_3$) δ -136.42 (d, *J* = 50.7 Hz). ESI-HRMS m/z: Calcd. for $C_{20}H_{43}FO_5Si_3Na$ $[M+Na]^+$: 489.2294; found: 489.2299.

Synthesis of 2,3-*O*-carbonate-4-*O*-trimethylsilyl-6-*O*-benzoyl-α-D-mannopyranosyl fluoride (S13)

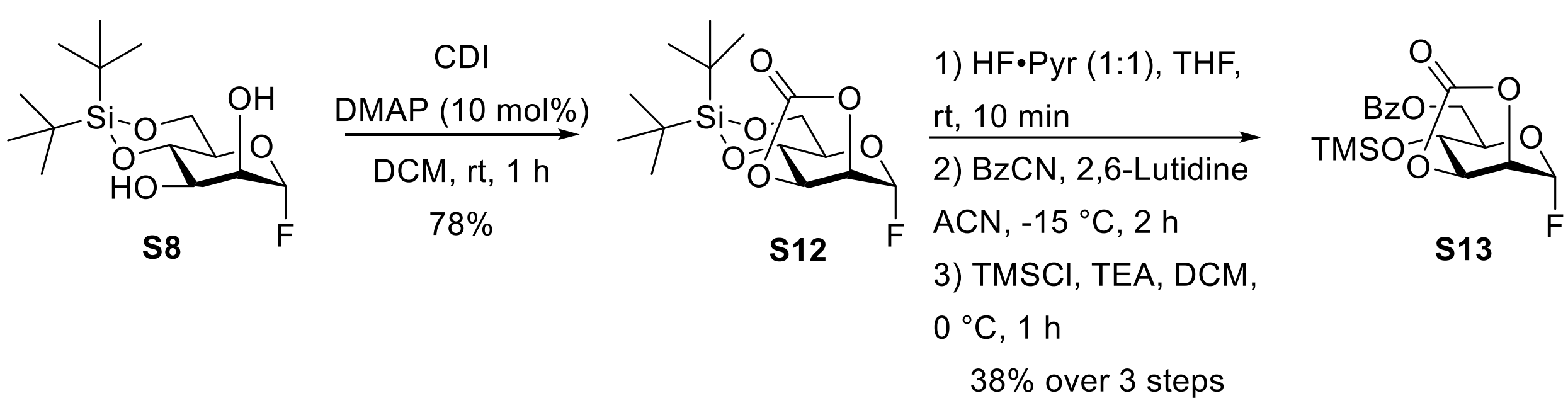

2,3-*O*-carbonate-4,6-*O*-di-*tert*-butylsilylene-α-D-mannopyranosyl fluoride (S12)

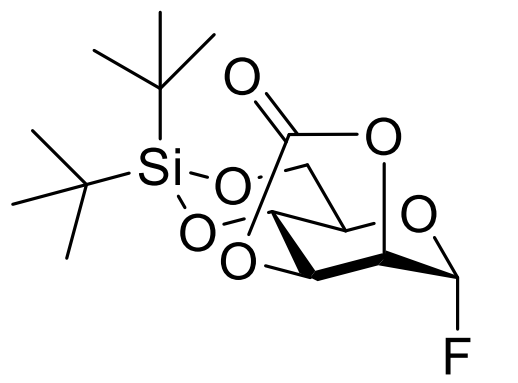

To a stirred solution of 4,6-*O*-di-*tert*-butylsilylene-α-D-mannopyranosyl fluoride **S8** (300 mg, 930 μmol) in DCM (3.00 mL) was added N,N-dimethylaminopyridine (5.68 mg, 46.5 μmol) and then carbonyldiimidazole (166 mg, 1.02 mmol) at rt under N_2. After for 2 h the reaction mixture was diluted with DCM (50 mL) and washed with water (50 mL X 2) and brine (50 mL). The organic layer was dried over anhydrous Na_2SO_4, filtered, and concentrated under reduced pressure. The crude was purified by silica gel column chromatography (0% to 30% EtOAc in Hexanes) to afford the product **S12** (253 mg, 726 μmol, 78%) as a white solid. ^{1}H NMR (500 MHz, $CDCl_3$) δ 5.82 (d, *J* = 48.1 Hz, 1H), 4.80 (dd, *J* = 7.2, 5.1 Hz, 1H), 4.74 (t, *J* = 7.3 Hz, 1H), 4.25 (dd, *J* = 10.1, 5.0 Hz, 1H), 4.04 (dd, *J* = 10.1, 7.4 Hz, 1H), 3.93 (td, *J* = 10.1, 1.1 Hz, 1H), 3.85 (td, *J* = 10.1, 5.0 Hz, 1H), 1.06 (s, 9H), 1.00 (s, 9H). ^{13}C NMR (126 MHz, $CDCl_3$) δ 152.7 (d, *J* = 1.9 Hz), 103.3 (d, *J* = 217.3 Hz), 78.5, 74.7 (d, *J* = 50.7 Hz), 74.4, 65.6, 65.5 (d, *J* = 3.1 Hz), 27.4, 26.9, 22.8, 20.0. ^{19}F NMR (471 MHz, $CDCl_3$) δ -133.68 (dd, *J* = 47.8, 5.7 Hz). ESI-HRMS m/z: Calcd. for $C_{15}H_{25}FO_6SiNa$ $[M+Na]^+$: 371.1296; found: 371.1287.

2,3-*O*-carbonate-4-*O*-trimethylsilyl-6-*O*-benzoyl-α-D-mannopyranosyl fluoride (S13)

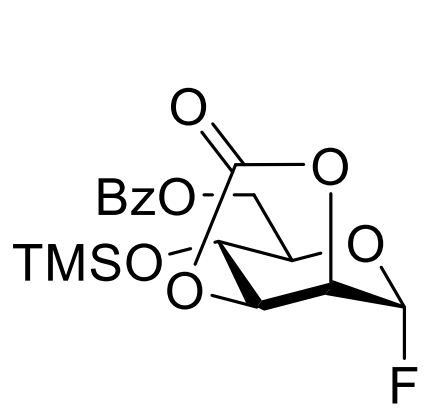

To a stirred solution of 2,3-*O*-carbonate-4,6-*O*-di-*tert*-butylsilylene-α-D-mannopyranosyl fluoride **S12** (227 mg, 651 μmol) in THF (16.3 mL) was added pyridine (3.35 g, 3.41 mL, 42.3 mmol) at rt. The solution was then treated dropwise with HF•Pyridine solution (372 mg, 340 μL, 13.0 mmol,

70% HF in Pyridine) at rt. After stirring for 10 minutes the reaction mixture was diluted with 50 mL EtOAc and washed with sat. $NaHCO_3$ (100 mL X 3), water (100 mL), and brine. The organic layer was dried over anhydrous Na_2SO_4, filtered, and concentrated under reduced pressure to afford the crude product that was taken forward with no further purification. The resulting material was dissolved in acetonitrile (4.0 mL) and was treated with 2,6-Lutidine (28.5 mg, 30.8 µL, 266 µmol) at -15 °C under N_2. The reaction mixture was then treated with benzoyl cyanide (34.9 mg, 266 µmol) and the reaction stirred for 16 h while warming to rt. The reaction mixture was then concentrated under reduced pressure. The crude was then subjected to general procedure A with trimethylchlorosilane (22 mg, 26 µL, 210 µmol) and triethylamine (42 mg, 58 µL, 410 µmol) in DCM (2.0 mL). Purification by silica gel column chromatography (5% to 25% EtOAc in hexanes) to yield the desired product **S13** (24 mg, 63 µmol, 38% over 3 steps) as a colorless residue. ^{1}H NMR (500 MHz, $CDCl_3$) δ 8.04 (dd, *J* = 8.3, 1.4 Hz, 2H), 7.60 (tt, *J* = 7.3, 1.3 Hz, 1H), 7.47 (t, *J* = 7.8 Hz, 2H), 5.90 (d, *J* = 47.9 Hz, 1H), 4.82 (dd, *J* = 7.2, 4.6 Hz, 1H), 4.75 (t, *J* = 7.0 Hz, 1H), 4.66 (dd, *J* = 12.3, 2.4 Hz, 1H), 4.49 (dd, *J* = 12.3, 4.4 Hz, 1H), 4.07 (ddd, *J* = 10.1, 4.3, 2.3 Hz, 1H), 3.95 (dd, *J* = 10.1, 6.9 Hz, 1H), 0.17 (s, 9H). ^{13}C NMR (126 MHz, $CDCl_3$) δ 166.2, 152.7 (d, *J* = 1.9 Hz), 133.5, 129.8, 128.7, 103.4 (d, *J* = 217.1 Hz), 79.6, 74.7 (d, *J* = 51.2 Hz), 69.7 (d, *J* = 3.7 Hz), 68.5, 62.1, 0.1. ^{19}F NMR (471 MHz, $CDCl_3$) δ -134.83 (dd, *J* = 47.7, 4.5 Hz). ESI-HRMS m/z: Calcd. for $C_{17}H_{21}FO_7SiNa$ $[M+Na]^+$: 402.1378; found: 402.1384.

Synthesis of 2,3-*O*-carbonate-4,6-*O*-di-trimethylsilyl-α-D-glucopyranosyl fluoride (25) and 2,3-*O*-carbonate-4-*O*-trimethylsilyl-6-*O*-benzoyl-α-D-glucopyranosyl fluoride (S16)

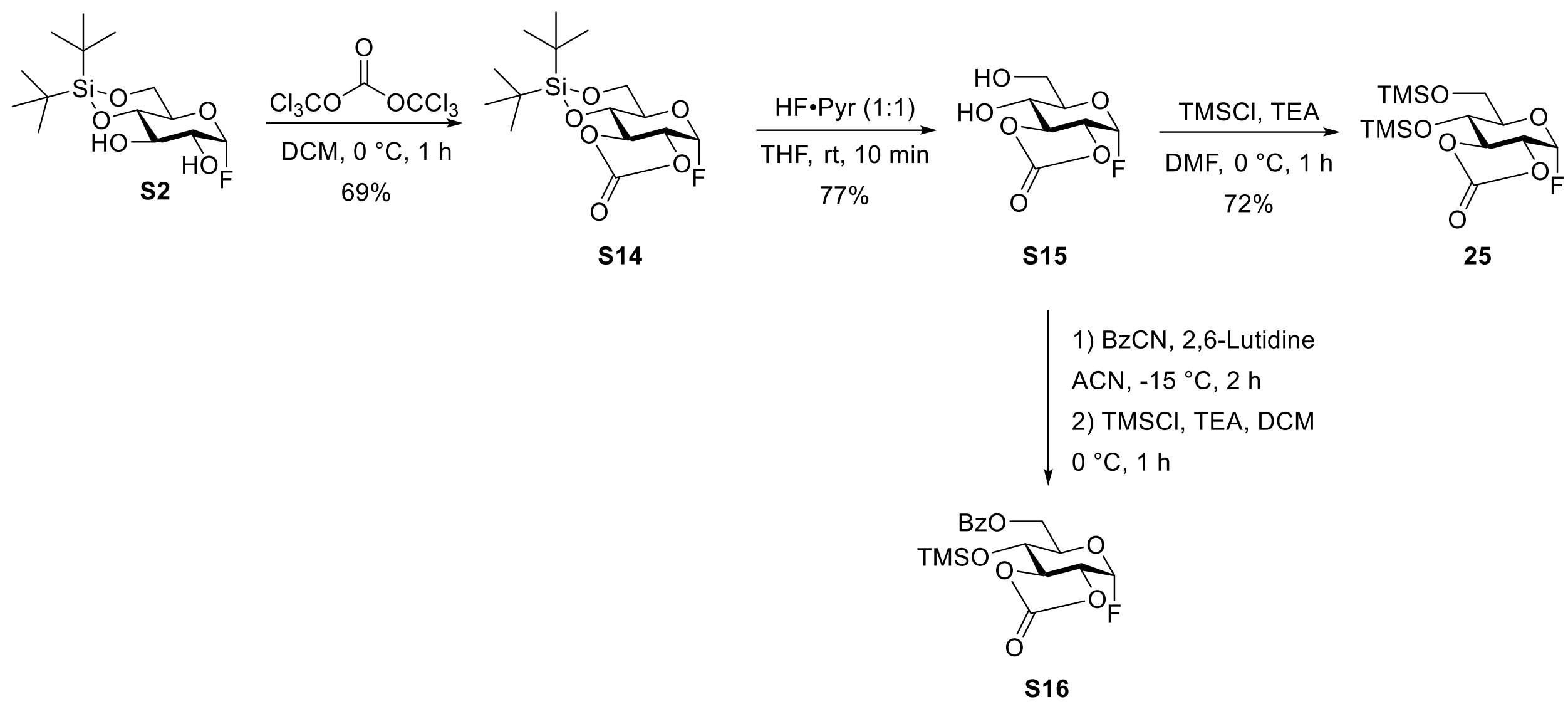

2,3-*O*-carbonate-4,6-*O*-di-tert-butylsilylene-α-D-glucopyranosyl fluoride (S14)

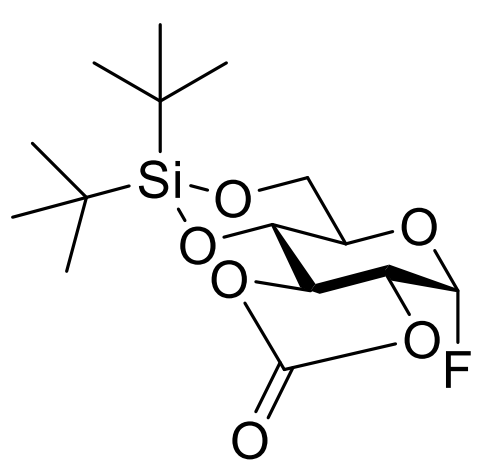

To a stirred solution of 4,6-*O*-di-*tert*-butylsilylene-α-D-glucopyranosyl fluoride **S2** (500 mg, 1.85 mmol) in DCM (18.5 mL) was treated with triethylamine (561 mg, 774 µL, 5.55 mmol) at 0 °C under N_2. Trisphosgene (603 mg, 2.03 mmol) was added slowly to the reaction mixture. After stirring for 1 h the reaction mixture was diluted with 100 mL DCM and washed with $NaHCO_3$ (100 mL X 3), water (100 mL) and brine (100 mL). The organic layer was dried over anhydrous Na_2SO_4, filtered, and concentrated under reduced pressure. The crude was purified by silica gel column chromatography (5% to 25% EtOAc in Hexanes) to afford the product **S14** (374 mg, 1.26 mmol, 68%) as a white solid. ^{1}H NMR (500 MHz, $CDCl_3$) δ 5.91 (dd, J = 55.7, 2.1 Hz, 1H), 4.67 (dd, J = 11.6, 9.6 Hz, 1H), 4.35 (ddd, J = 9.9, 8.6, 1.8 Hz, 1H), 4.22 (dd, J = 9.3, 4.2 Hz, 1H), 4.16 (ddd, J = 22.8, 11.6, 2.1 Hz, 1H), 4.01 – 3.89 (m, 2H), 1.07 (s, 9H), 1.01 (s, 9H). ^{13}C NMR (126 MHz, $CDCl_3$) δ 152.7, 101.9 (d, J = 236.3 Hz), 78.2 (d, J = 2.2 Hz), 76.9 (d, J = 26.5 Hz), 74.7, 71.8 (d,

J = 1.2 Hz), 65.7, 27.4, 26.9, 22.7, 20.1. ^{19}F NMR (471 MHz, $CDCl_3$) δ -147.97 (dd, J = 55.5, 22.6 Hz). ESI-HRMS m/z: Calcd. for $C_{15}H_{25}FO_6SiNa$ $[M+Na]^+$: 371.1296; found: 371.1291.

2,3-*O*-carbonate-α-D-glucopyranosyl fluoride (S15)

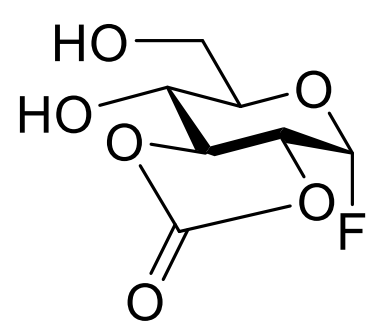

To a stirred solution of 2,3-*O*-carbonate-4,6-*O*-di-tert-butylsilylene-α-D-glucopyranosyl fluoride **S14** (200 mg, 574 µmol) in THF (14.3 mL) was added pyridine (2.95 g, 3.01 mL, 37.3 mmol) at rt. The solution was then treated dropwise with HF•Pyridine solution (328 mg, 310 µL, 11.5 mmol, 70% HF in Pyridine) at rt. After stirring for 10 minutes the reaction mixture was diluted with 50 mL EtOAc and washed with sat. $NaHCO_3$ (100 mL X 3), water (100 mL), and brine. The organic layer was dried over anhydrous Na_2SO_4, filtered, and concentrated under reduced pressure. The crude was purified by silica gel column chromatography (30% to 70% EtOAc in Hexanes) to afford the product **S15** (94 mg, 450 µmol, 79%) as a white solid. ^{1}H NMR (500 MHz, DMSO-d_6) δ 6.21 (dd, J = 57.7, 2.1 Hz, 1H), 6.10 (d, J = 6.4 Hz, 1H), 4.89 (t, J = 5.7 Hz, 1H), 4.64 (dd, J = 11.8, 9.4 Hz, 1H), 4.56 (ddd, J = 23.1, 11.9, 2.1 Hz, 1H), 4.07 – 3.99 (m, 1H), 3.70 – 3.63 (m, 1H), 3.62 – 3.53 (m, 2H). ^{13}C NMR (126 MHz, DMSO-d_6) δ 153.2, 102.3 (d, J = 229.0 Hz), 78.9 (d, J = 3.0 Hz), 78.8, 75.9 (d, J = 25.8 Hz), 66.7, 59.0. ^{19}F NMR (471 MHz, DMSO-d_6) δ -146.68 (dd, J = 57.9, 23.3 Hz). ESI-HRMS m/z: Calcd. for $C_7H_9FO_6Na$ $[M+Na]^+$: 231.0275; found: 231.0283.

2,3-*O*-carbonate-4,6-*O*-di-trimethylsilyl-α-D-glucopyranosyl fluoride (25)

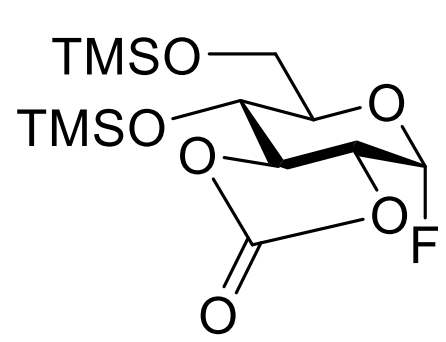

This compound was prepared by following general procedure A, using 2,3-*O*-carbonate-α-D-glucopyranosyl fluoride **S14** (50 mg, 240 μmol), chlorotrimethylsilane (55 mg, 64 μL, 0.504 mmol), and triethylamine (167 μL, 122 mg, 1.20 mmol) in *N,N*-dimethylformamide (2.40 mL). Purification by silica gel column chromatography (5% to 25% EtOAc in hexanes) to yield the desired product **25** (61 mg, 172 μmol, 72%) as a colorless residue. ^{1}H NMR (500 MHz, $CDCl_3$) δ 5.93 (dd, *J* = 56.8, 2.2 Hz, 1H), 4.62 (dd, *J* = 11.9, 9.8 Hz, 1H), 4.24 (td, *J* = 9.4, 1.6 Hz, 1H), 4.11 (ddd, *J* = 21.8, 11.8, 2.1 Hz, 1H), 3.82 (dd, *J* = 11.8, 3.0 Hz, 1H), 3.75 (dd, *J* = 11.9, 1.9 Hz, 1H), 3.71 (dd, *J* = 8.5, 1.4 Hz, 1H), 0.18 (s, 9H), 0.11 (s, 9H). ^{13}C NMR (126 MHz, $CDCl_3$) δ 153.0, 102.0 (d, *J* = 233.7 Hz), 79.3 (d, *J* = 3.1 Hz), 78.4, 76.6 (d, *J* = 26.8 Hz), 68.1, 59.8, 0.0, -0.3. ^{19}F NMR (471 MHz, $CDCl_3$) δ -147.37 (dd, *J* = 57.0, 21.7 Hz). ESI-HRMS m/z: Calcd. for $C_{13}H_{25}FO_6Si_2NH4$ $[M+ NH_4]^+$: 370.1511; found: 370.1507.

2,3-*O*-carbonate-4-*O*-trimethylsilyl-6-*O*-benzoyl-α-D-glucopyranosyl fluoride (S16)

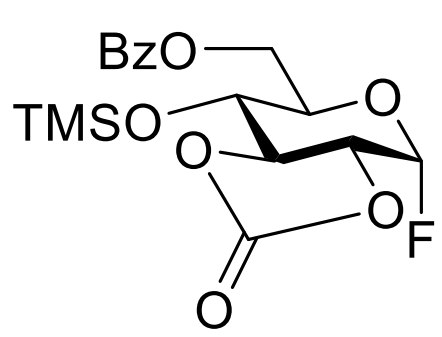

To a stirred solution of 2,3-*O*-carbonate-α-D-glucopyranosyl fluoride **S15** (90 mg, 430 μmol) in acetonitrile (10.0 mL) was treated with 2,6-lutidine (51 mg, 55 μL, 480 μmol) at -15 °C under N_2. The reaction mixture was then treated with benzoyl cyanide (62 mg, 480 μmol) and the reaction stirred for 16 h while warming to rt. The reaction mixture was then concentrated under reduced pressure. The crude was purified by silica gel column chromatography (10% to 40% EtOAc in Hexanes) to afford the crude product that was taken forward with no further purification. The resulting material was then subjected to general procedure A, using chlorotrimethylsilane (76 mg, 89 μL, 0.705 mmol), and triethylamine (137 μL, 99 mg, 0.986 mmol) in *N,N*-dimethylformamide (1.40 mL). Purification by silica gel column chromatography (5% to 25% EtOAc in hexanes) to

yield the desired product **S16** (29 mg, 75 μmol, 54% over 2 steps) as a colorless residue. ^{1}H NMR (500 MHz, $CDCl_3$) δ 8.03 (dd, *J* = 8.4, 1.4 Hz, 2H), 7.60 (d, *J* = 7.3 Hz, 1H), 7.47 (t, *J* = 7.9 Hz, 2H), 5.97 (dd, *J* = 56.2, 2.1 Hz, 1H), 4.71 – 4.62 (m, 2H), 4.50 (dd, *J* = 12.3, 3.8 Hz, 1H), 4.27 – 4.14 (m, 2H), 4.06 (dt, *J* = 8.8, 2.9 Hz, 1H), 0.18 (s, 9H). ^{13}C NMR (126 MHz, $CDCl_3$) δ 166.0, 152.6, 133.5, 129.7, 128.7, 101.7 (d, *J* = 235.4 Hz), 78.9 (d, *J* = 3.1 Hz), 76.6 (d, *J* = 26.6 Hz), 75.9 (d, *J* = 1.2 Hz), 69.2, 61.6, 0.0. ^{19}F NMR (471 MHz, $CDCl_3$) δ -147.95 (dd, *J* = 56.3, 21.8 Hz). ESI-HRMS m/z: Calcd. for $C_{17}H_{21}FO_7SiNa$ $[M+Na]^+$: 407.0932; found: 407.0929.

Synthesis of 2,3-*O*-carbonate-4-*O*-benzyl-6-*O*-trimethylsilyl-α-D-glucopyranosyl fluoride (S20)

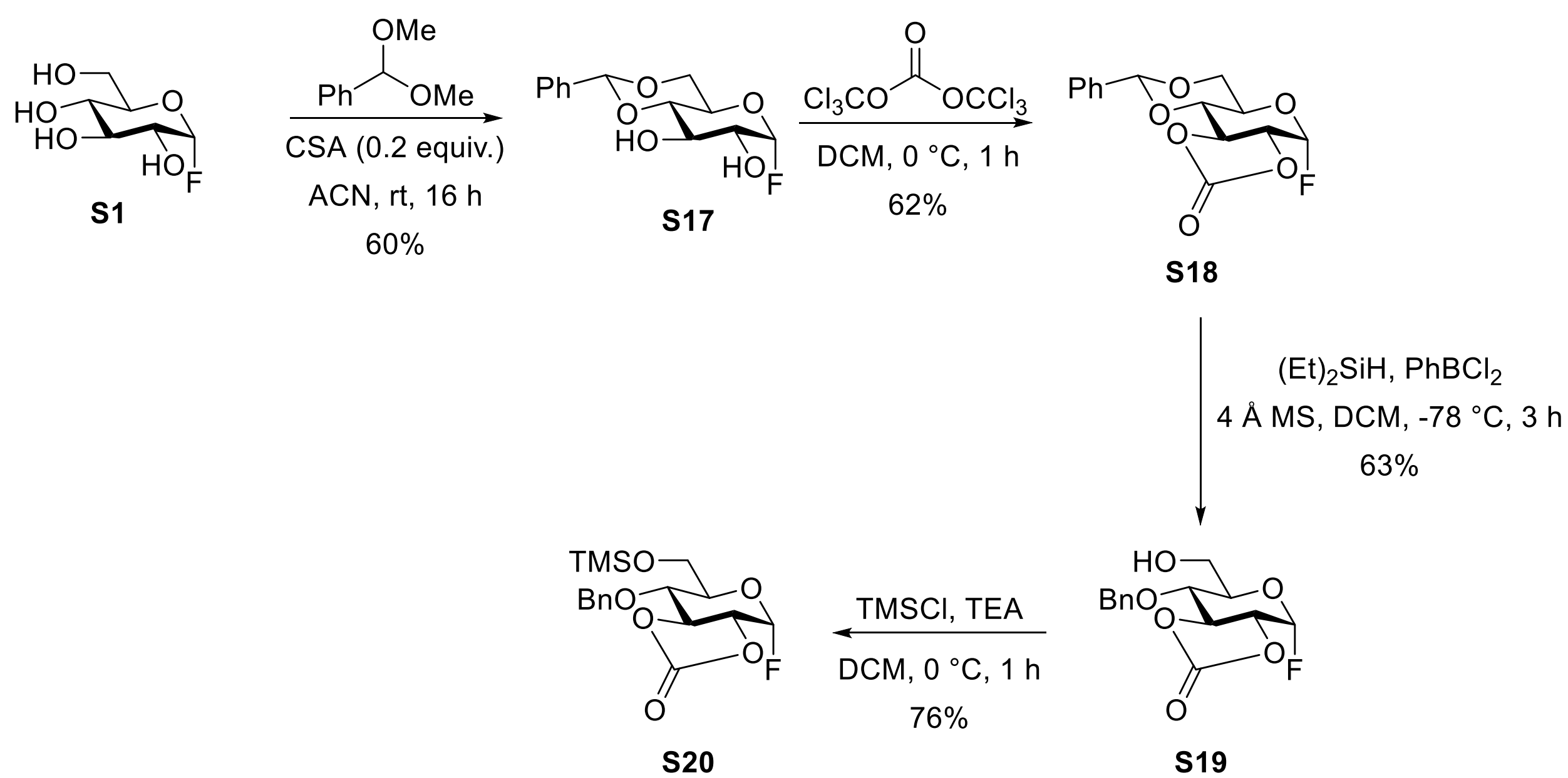

4,6-*O*-benzylidene-α-D-glucopyranosyl fluoride (S17)

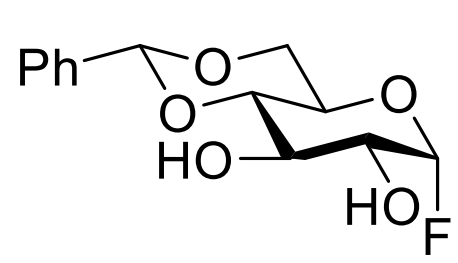

To a stirred mixture of α-D-glucopyranosyl fluoride[3] (1.54 g, 8.45 mmol) and benzaldehyde dimethyl acetal (5.15 g, 5.08 mL, 33.8 mmol) in acetonitrile (42.3 mL) was added (±)-10-

camphorsulfonic acid (196 mg, 0.845 mmol) at rt under N_2. After stirring the reaction mixture for 16 h the reaction was quenched with the dropwise addition of triethylamine (1 mL) and concentrated under reduced pressure. The crude was purified by silica gel column chromatography (0% to 65% EtOAc in Hexanes) to afford the desired product **S17** (1.37 g, 5.07 mmol, 60%) as a white solid. Spectral data matches as previously reported.[6]

2,3-*O*-carbonate-4,6-*O*-benzylidene-α-D-glucopyranosyl fluoride (S18)

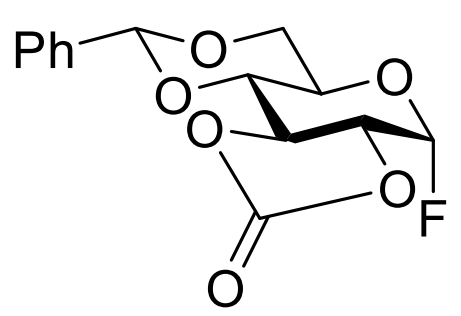

To a stirred solution of 4,6-*O*-benzylidene-α-D-glucopyranosyl fluoride **S17** (200 mg, 0.740 mmol) in DCM (14.8 mL) was treated with triethylamine (674 mg, 928 µL, 6.66 mmol) at 0 °C under N_2. Trisphosgene (329 mg, 1.11 mmol) was added slowly to the reaction mixture. After stirring for 1 h the reaction mixture was diluted with 100 mL DCM and washed with $NaHCO_3$ (100 mL X 3), water (100 mL) and brine (100 mL). The organic layer was dried over anhydrous Na_2SO_4, filtered, and concentrated under reduced pressure. The crude was purified by silica gel column chromatography (5% to 25% EtOAc in Hexanes) to afford the product **S18** (153 mg, 0.516 mmol, 62%) as a white solid. ^{1}H NMR (500 MHz, $CDCl_3$) δ 7.48 (dd, *J* = 6.9, 3.0 Hz, 2H), 7.39 (t, *J* = 3.0 Hz, 3H), 5.99 (dd, *J* = 54.9, 2.1 Hz, 1H), 5.62 (s, 1H), 4.87 (dd, *J* = 11.5, 10.0 Hz, 1H), 4.37 (dd, *J* = 10.4, 4.6 Hz, 1H), 4.25 (ddd, *J* = 23.3, 11.4, 2.1 Hz, 1H), 4.19 (dd, *J* = 8.9, 1.4 Hz, 1H), 4.05 (dd, *J* = 10.0, 4.7 Hz, 1H), 3.92 (t, *J* = 10.3 Hz, 1H). ^{13}C NMR (126 MHz, $CDCl_3$) δ 152.38, 135.97, 129.72, 128.57, 126.25, 102.32 (d, *J* = 236.8 Hz), 101.78, 79.09, 77.60, 75.44 (d, *J* = 1.9 Hz), 68.02, 67.83 (d, *J* = 1.6 Hz). ^{19}F NMR (471 MHz, $CDCl_3$) δ -148.54 (dd, *J* = 55.0, 23.2 Hz). ESI-HRMS m/z: Calcd. for $C_{14}H_{13}FO_6Na$ $[M+Na]^+$: 319.0588; found: 319.0585.

2,3-*O*-carbonate-4-*O*-benzyl-α-D-glucopyranosyl fluoride (S19)

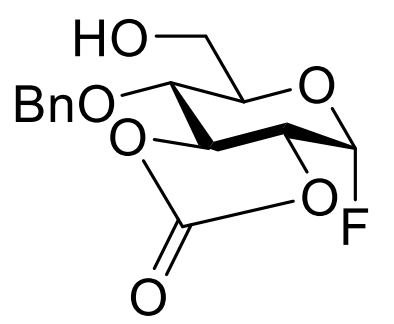

To a stirred mixture of 2,3-*O*-carbonate-4,6-*O*-benzylidene-α-D-glucopyranosyl fluoride **S18** (100 mg, 337 µmol) and 4 Å molecular sieves in DCM (5.0 mL) was added triethylsilane (196 mg, 270 µL, 1.69 mmol) and dichlorophenylborane (107 mg, 87.6 µL, 675 µmol) at -78 °C under N_2. After stirring for 3 h at -78 °C the reaction mixture was diluted with 8 mL DCM and slowly treated with a solution of triethylamine:methanol (1:1, 1.0 mL) and continued stirring. After warming to rt for 30 min, the reaction mixture was diluted with DCM (30 mL) and washed with sat. $NaHCO_3$ (30 mL X 2) and water (30 mL) and brine (30 mL). The organic layer was dried over anhydrous Na_2SO_4, filtered, and concentrated under reduced pressure. The crude was purified by silica gel column chromatography (10% to 40% EtOAc in Hexanes) to afford the desired product **S19** (63 mg, 210 µmol, 63%) as a colorless residue. ^{1}H NMR (500 MHz, $CDCl_3$) δ 7.42 – 7.30 (m, 5H), 5.96 (dd, *J* = 56.4, 2.0 Hz, 1H), 4.88 (d, *J* = 11.4 Hz, 1H), 4.81 (dd, *J* = 11.9, 9.8 Hz, 1H), 4.64 (d, *J* = 11.4 Hz, 1H), 4.18 – 4.08 (m, 2H), 3.91 – 3.86 (m, 2H), 3.83 (dd, *J* = 12.5, 3.4 Hz, 1H), 1.79 (s, 1H). ^{13}C NMR (126 MHz, $CDCl_3$) δ 152.9, 136.6, 128.7, 128.5, 128.2, 101.8 (d, *J* = 235.1 Hz), 79.2 (d, *J* = 3.2 Hz), 76.8 (d, *J* = 26.7 Hz), 76.3, 73.5, 73.1, 60.5. ^{19}F NMR (471 MHz, $CDCl_3$) δ -147.12 (dd, *J* = 56.7, 22.0 Hz). ESI-HRMS m/z: Calcd. for $C_{14}H_{15}FO_6Na$ $[M+Na]^+$: 321.0744; found: 321.0739.

2,3-*O*-carbonate-4-*O*-benzyl-6-*O*-trimethylsilyl-α-D-glucopyranosyl fluoride (S20)

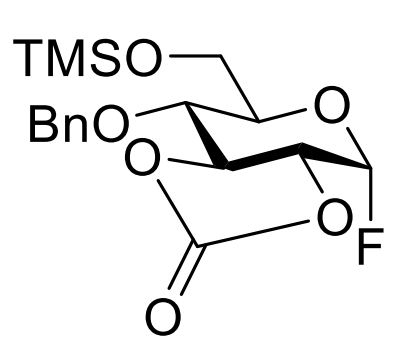

This compound was prepared by following general procedure A, with 2,3-*O*-carbonate-4-*O*-benzyl-α-D-glucopyranosyl fluoride **S19** (54 mg, 0.18 mmol), 2,6-Lutidine (58 mg, 63 µL, 0.54 mmol), and trimethylchlorosilane (30 mg, 34 µL, 0.27 mmol). Purification by silica gel column chromatography (5% to 15% EtOAc in Hexanes) to afford the product **S20** (51 mg, 0.14 mmol, 76%) as a colorless viscous residue. ^{1}H NMR (500 MHz, $CDCl_3$) δ 7.41 – 7.30 (m, 5H), 5.96 (dd, *J* = 56.8, 1.9 Hz, 1H), 4.87 (d, *J* = 11.3 Hz, 1H), 4.81 (dd, *J* = 11.8, 9.8 Hz, 1H), 4.63 (d, *J* = 11.3 Hz, 1H), 4.19 – 4.11 (m, 2H), 3.90 (dd, *J* = 11.6, 3.0 Hz, 1H), 3.86 (dd, *J* = 8.9, 1.8 Hz, 1H), 3.81 (dd, *J* = 11.6, 1.6 Hz, 1H), 0.12 (s, 9H). ^{13}C NMR (126 MHz, $CDCl_3$) δ 153.0, 136.8, 128.6, 128.3, 128.1, 101.9 (d, *J* = 234.2 Hz), 79.3 (d, *J* = 3.2 Hz), 76.9 (d, *J* = 26.6 Hz), 76.6, 73.6, 73.1, 60.2, -0.3. ^{19}F NMR (471 MHz, $CDCl_3$) δ -147.25 (dd, *J* = 56.5, 21.6 Hz). ESI-HRMS m/z: Calcd. for $C_{17}H_{23}FO_6NH_4$ $[M+NH_4]^+$: 388.1586; found: 388.1581.

IV. Experimental Procedures for Boron Catalyzed Glycosylations.

Methyl-2,3-di-*O*-benzyl-4,6-di-*O*-(2-*O*-acetyl-3,4,6-tri-*O*-benzyl-α-D-mannopyranosyl)-α-D-glucopyranoside (3)

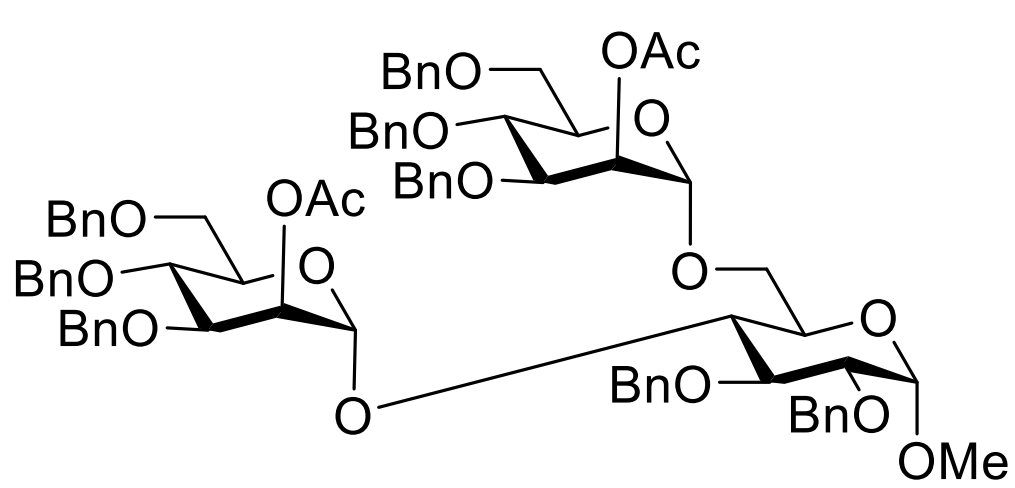

This compound was prepared by following the general experimental procedure C, using methyl-2,3-di-*O*-benzyl-4,6-*O*-di-ethylsilylene-α-D-mannopyranoside (**1a**) (50 mg, 109 µmol) or methyl-2,3-di-*O*-benzyl-4,6-*O*-di-isopropylsilylene-α-D-mannopyranoside (**1b**) (50 mg, 103 µmol), 2-*O*-acetyl-3,4,6-tri-O-benzyl-α-D-mannopyranosyl fluoride (**2**) (113 mg, 229 µmol for **1a** or 107 mg, 216 µmol for **1b**), and BF_3•OEt_2 (1.3 µL, 10.3 µmol). Purification by column chromatography eluting with ethyl acetate/hexane (1:19 to 1:5) to afford the product **3** (96 mg, 73 µmol, 67%, >97:3

dr from **1a** or 75 mg, 57 μmol, 55%, >97:3 dr from **1b**) as a clear residue. The byproduct **4** (27 mg, 32 μmol, 29%, >97:3 dr from **1a** or 32 mg, 38 μmol, 37%, >97:3 dr from **1b**) was isolated as a clear residue. Spectral data matches as previously reported.[1]

Methyl-2,4,6-tri-*O*-benzyl-3-*O*-(2-*O*-acetyl-3-*O*-benzyl-4,6-*O*-di-tert-butylsilylene-α-D-mannopyranosyl)-α-D-glucopyranoside (7)

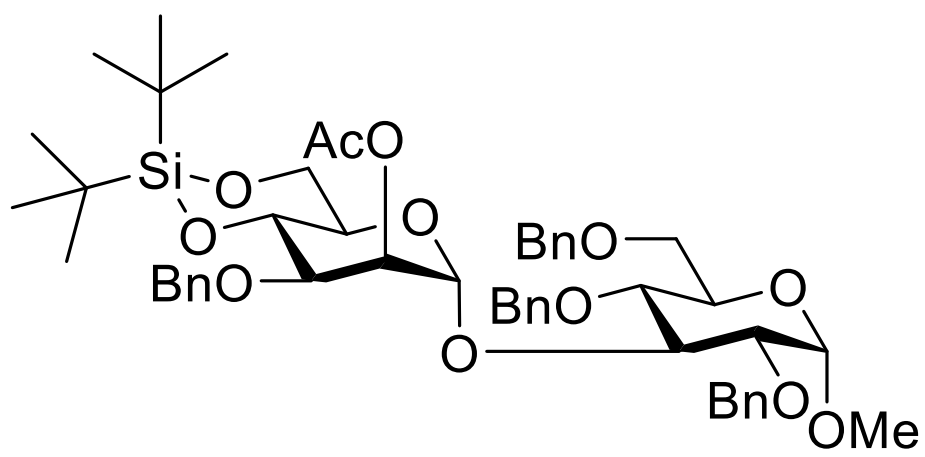

To a stirred mixture of 2-*O*-acetyl-3-*O*-benzyl-4,6-*O*-di-*tert*-butylsilylene-α-D-mannopyranosyl fluoride **5** (20 mg, 44 μmol) and methyl-2,4,6-tri-*O*-benzyl-3-*O*-trimethylsilyl-α-D-glucopyranoside **6** (23.6 mg, 44 μmol) in toluene (0.44 mL) was added $B(C_6F_5)_3$ (1.13 mg, 2.20 μmol) or BF_3•OEt_2 (0.62 mg, 4.40 μmol, 0.56 μL) at rt under N_2. After stirring for 45 min when using $B(C_6F_5)_3$ or 3 h when using BF_3•OEt_2, TLC indicated full consumption of the starting material and the reaction was quenched with a few droplets of triethylamine. The reaction was concentrated under reduced pressure and purified by silica gel column chromatography eluting with ethyl acetate/hexane (1:19 to 1:5) to afford the product **7** (25 mg, 27 μmol, 62% when using $B(C_6F_5)_3$ and 21 mg, 23 μmol, 53% when using BF_3•OEt_2) as a white foam. Spectral data matches as previously reported.[4]

2-*O*-acetyl-3-*O*-(2-*O*-acetyl-3,4,6-tri-*O*-benzyl-α-D-mannopyranosyl)-4,6-*O*-di-*tert*-butylsilylene-α-D-glucopyranosyl fluoride (13)

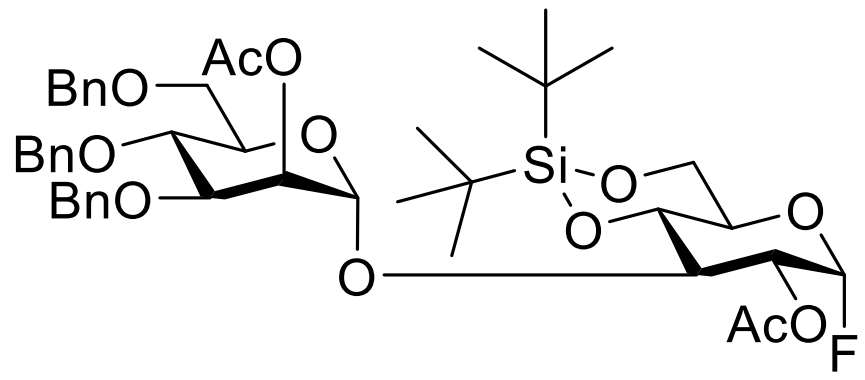

This compound was prepared by following general procedure B, with 2-*O*-acetyl-3,4,6-tri-*O*-benzyl-α-D-mannopyranosyl fluoride **2** (119 mg, 240 µmol) and 2-*O*-acetyl-3-*O*-trimethylsilyl-4,6-*O*-di-*tert*-butylsilylene-α-D-glucopyranosyl fluoride **23** (100 mg, 229 µmol) with BF_3•OEt_2 (3.25 mg, 2.90 µL, 22.9 µmol) in toluene (4.58 mL). Purification by silica gel column chromatography (5% to 25% EtOAc in hexanes) afforded the desired product **13** (151 mg, 180 µmol, 79%) as a colorless residue. ^{1}H NMR (500 MHz, $CDCl_3$) δ 7.36 (dd, *J* = 8.3, 1.6 Hz, 2H), 7.33 – 7.24 (m, 11H), 7.13 (dd, *J* = 7.4, 2.1 Hz, 2H), 5.66 (dd, *J* = 53.4, 2.8 Hz, 1H), 5.49 (t, *J* = 2.4 Hz, 1H), 5.37 (d, *J* = 2.0 Hz, 1H), 4.85 (d, *J* = 10.9 Hz, 1H), 4.79 – 4.68 (m, 3H), 4.53 (d, *J* = 12.0 Hz, 1H), 4.50 (d, *J* = 10.9 Hz, 1H), 4.48 (d, *J* = 10.9 Hz, 1H), 4.18 (dd, *J* = 9.3, 3.9 Hz, 1H), 4.12 (dd, *J* = 10.1, 8.6 Hz, 1H), 4.00 – 3.86 (m, 6H), 3.81 (dd, *J* = 10.8, 3.6 Hz, 1H), 3.75 (dd, *J* = 10.7, 1.8 Hz, 1H), 2.11 (s, 3H), 2.02 (s, 3H), 1.09 (s, 9H), 0.97 (s, 9H). ^{13}C NMR (126 MHz, $CDCl_3$) δ 170.4, 169.9, 138.7, 138.3, 138.0, 128.5, 128.4, 128.38, 128.33, 127.9, 127.8, 127.77, 127.74, 127.6, 104.2 (d, *J* = 228.1 Hz), 99.2, 78.3, 75.2, 75.0, 74.2, 73.7, 72.08, 72.03, 71.2 (d, *J* = 24.9 Hz), 69.0, 68.4 (d, *J* = 3.6 Hz), 68.3, 66.1, 27.6, 26.9, 22.8, 21.1, 20.9, 20.0. ^{19}F NMR (471 MHz, $CDCl_3$) δ -147.69 (dd, *J* = 53.4, 24.8 Hz). Anomeric configurations confirmed by the following ^{1}H spectral data: ^{1}H NMR (500 MHz, $CDCl_3$) δ 5.66 (dd, *J* = 53.4, 2.8 Hz, 1H, α-fluoroglucose), 5.37 (d, *J* = 2.0 Hz, 1H, α-mannose). ESI-HRMS m/z: calc'd for $C_{45}H_{59}FO_{12}Si$ $[M+Na]^+$: 861.3652, found: 861.3646.

2-*O*-benzoyl-3-*O*-(2-*O*-acetyl-3,4,6-tri-*O*-benzyl-α-D-mannopyranosyl)-4,6-*O*-di-*tert*-butylsilylene-α-D-glucopyranosyl fluoride (14)

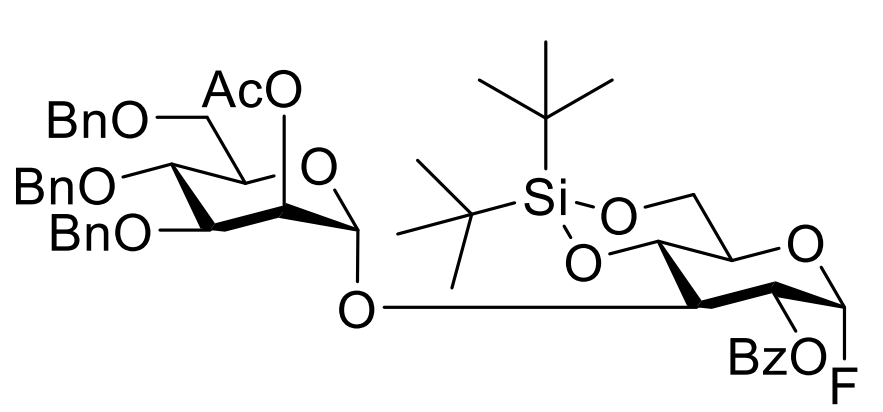

This compound was prepared by following general procedure B, with 2-*O*-acetyl-3,4,6-tri-*O*-benzyl-α-D-mannopyranosyl fluoride **2** (104 mg, 211 µmol) and 2-*O*-benzoyl-3-*O*-trimethylsilyl-4,6-*O*-di-*tert*-butylsilylene-α-D-glucopyranosyl fluoride (100 mg, 201 µmol) **S6** with BF_3•OEt_2 (2.85 mg, 2.54 µL, 20.1 µmol) in toluene (4.01 mL). Purification by silica gel column chromatography (5% to 25% EtOAc in hexanes) afforded the desired product **14** (155 mg, 172 µmol, 86%) as a colorless residue. ^{1}H NMR (500 MHz, $CDCl_3$) δ 8.02 (dd, *J* = 8.4, 1.4 Hz, 2H), 7.50 (tt, *J* = 7.3, 1.4 Hz, 1H), 7.31 (t, *J* = 7.9 Hz, 2H), 7.28 – 7.18 (m, 14H), 6.92 (dd, *J* = 6.5, 2.8 Hz, 2H), 5.71 (dd, *J* = 53.3, 2.9 Hz, 1H), 5.50 (dd, *J* = 3.1, 1.9 Hz, 1H), 5.37 (d, *J* = 2.0 Hz, 1H), 5.11 (ddd, *J* = 24.8, 9.9, 2.9 Hz, 1H), 4.68 (dd, *J* = 10.9, 9.7 Hz, 2H), 4.57 (d, *J* = 12.1 Hz, 1H), 4.45 (d, *J* = 10.9 Hz, 1H), 4.32 – 4.19 (m, 4H), 4.08 – 3.98 (m, 2H), 3.93 (t, *J* = 9.3 Hz, 1H), 3.89 – 3.80 (m, 2H), 3.62 (dt, *J* = 9.5, 2.5 Hz, 1H), 3.27 (dd, *J* = 10.9, 3.0 Hz, 1H), 3.22 (dd, *J* = 10.9, 2.0 Hz, 1H), 2.07 (s, 3H), 1.11 (s, 9H), 0.99 (s, 9H). ^{13}C NMR (126 MHz, $CDCl_3$) δ 168.7, 137.6, 137.1, 136.9, 127.9, 127.39, 127.30, 127.19, 127.10, 127.0, 126.7, 126.6, 126.4, 126.3, 126.1, 103.3 (d, *J* = 228.4 Hz), 98.6, 77.0, 75.2, 73.5, 72.6, 72.4, 70.8, 70.7, 69.9 (d, *J* = 24.6 Hz), 67.4, 67.3 (d, *J* = 3.5 Hz), 67.0, 64.9, 26.5, 25.7, 21.6, 19.9, 18.8. ^{19}F NMR (377 MHz, $CDCl_3$) δ -146.88 (dd, *J* = 53.4, 24.7 Hz). Anomeric configurations confirmed by the following ^{1}H spectral data: ^{1}H NMR (500 MHz, $CDCl_3$) δ 5.71 (dd, *J* = 53.3, 2.9 Hz, 1H, α-fluoroglucose), 5.37 (d, *J* = 2.0 Hz, 1H, α-mannose). ESI-HRMS m/z: calc'd for $C_{50}H_{61}FO_{12}Si$ $[M+NH_4]^+$: 918.4255, found: 918.4245.

2-*O*-(2-*O*-acetyl-3,4,6-tri-*O*-benzyl-α-D-mannopyranosyl)-3-*O*-acetyl-4,6-*O*-di-*tert*-butylsilylene-α-D-glucopyranosyl fluoride (15)

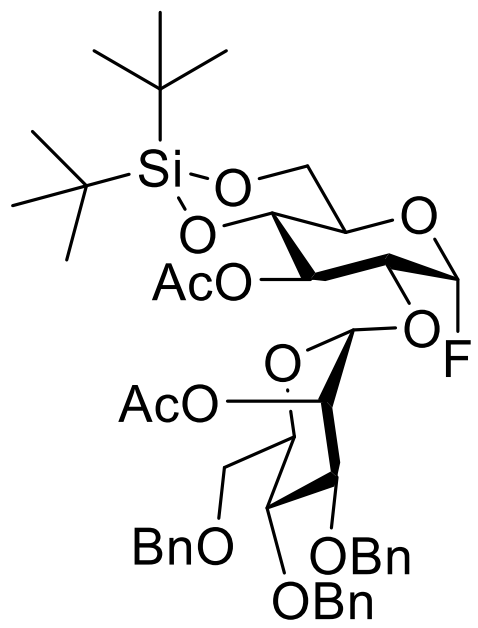

This compound was prepared by following general procedure B, with 2-*O*-acetyl-3,4,6-tri-*O*-benzyl-α-D-mannopyranosyl fluoride **2** (122 mg, 248 μmol) and 2-*O*-trimethylsilyl-3-*O*-acetyl-4,6-*O*-di-*tert*-butylsilylene-α-D-glucopyranosyl fluoride **S7** (103 mg, 236 μmol) with BF_3•OEt_2 (3.35 mg, 2.91 μL, 23.6 μmol) in toluene (4.72 mL). Purification by silica gel column chromatography (5% to 25% EtOAc in hexanes) afforded the desired product **15** (122 mg, 145 μmol, 62%) as a colorless residue. ^{1}H NMR (500 MHz, $CDCl_3$) δ 7.34 – 7.18 (m, 13H), 7.08 (dd, *J* = 7.3, 2.2 Hz, 2H), 5.61 (dd, *J* = 52.7, 2.9 Hz, 1H), 5.37 – 5.29 (m, 2H), 4.98 (d, *J* = 2.0 Hz, 1H), 4.82 (d, *J* = 11.1 Hz, 1H), 4.62 (dd, *J* = 11.8, 9.5 Hz, 2H), 4.47 (d, *J* = 3.5 Hz, 1H), 4.45 (d, *J* = 2.6 Hz, 1H), 4.42 (d, *J* = 11.3 Hz, 1H), 4.15 (dd, *J* = 9.9, 4.9 Hz, 1H), 3.95 (td, *J* = 10.1, 4.9 Hz, 1H), 3.87 – 3.70 (m, 7H), 3.66 (d, *J* = 10.9 Hz, 1H), 2.10 (s, 3H), 1.95 (s, 3H), 0.98 (s, 9H), 0.94 (s, 9H). ^{13}C NMR (126 MHz, $CDCl_3$) δ 170.4, 169.8, 138.6, 138.2, 137.8, 128.5, 128.48, 128.45, 128.3, 127.9, 127.79, 127.78, 127.59, 127.58, 103.5 (d, *J* = 231.3 Hz), 95.4, 77.7, 75.0, 74.6, 73.76, 73.5, 72.3 (d, *J* = 25.0 Hz), 72.1, 72.0, 71.6, 68.8, 68.6 (d, *J* = 3.6 Hz), 68.5, 66.1, 27.4, 26.9, 22.7, 21.2, 20.8, 20.1. ^{19}F NMR (471 MHz, $CDCl_3$) δ -147.56 (dd, *J* = 52.5, 24.4 Hz). Anomeric configurations confirmed by the following ^{1}H spectral data: ^{1}H NMR (500 MHz, $CDCl_3$) δ 5.61 (dd, *J* = 52.7, 2.9 Hz, 1H, α-fluoroglucose), 4.98 (d, *J* = 2.0 Hz, 1H, α-mannose). ESI-HRMS m/z: calc'd for $C_{45}H_{59}FO_{12}Si$ $[M+ Na]^+$: 861.3651, found: 861.3647

2-*O*-benzoyl-3-*O*-(2-*O*-acetyl-3,4,6-tri-*O*-benzyl-α-D-mannopyranosyl)-4,6-*O*-di-*tert*-butylsilylene-α-D-mannopyranosyl fluoride (16)

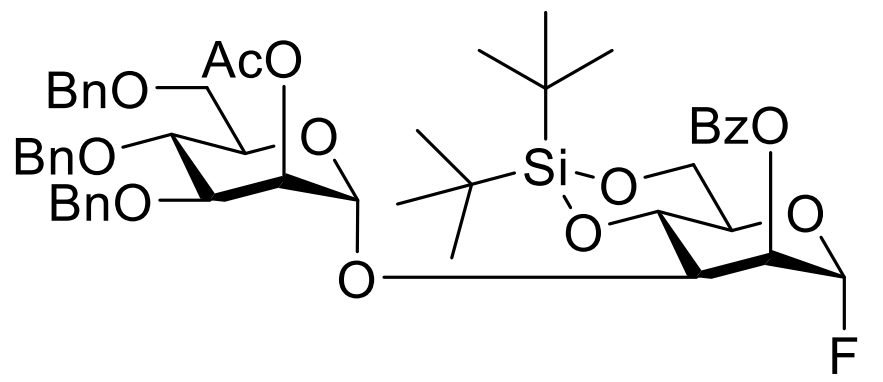

This compound was prepared by following general procedure B, with 2-*O*-acetyl-3,4,6-tri-*O*-benzyl-α-D-mannopyranosyl fluoride **2** (107 mg, 217 μmol) and 2-*O*-benzoyl-3-*O*-trimethylsilyl-4,6-*O*-di-*tert*-butylsilylene-α-D-mannopyranosyl fluoride **S9** (103 mg, 207 μmol) with BF_3•OEt_2 (2.93 mg, 2.62 μL, 20.7 μmol) in toluene (4.13 mL). Purification by silica gel column chromatography (5% to 25% EtOAc in hexanes) afforded the desired product **16** (120 mg, 133 μmol, 64%) as a colorless viscous residue. ^{1}H NMR (600 MHz, $CDCl_3$) δ 8.05 – 8.00 (m, 2H), 7.60 (t, *J* = 7.4 Hz, 1H), 7.49 – 7.44 (m, 2H), 7.38 – 7.34 (m, 2H), 7.33 – 7.23 (m, 7H), 7.21 – 7.13 (m, 7H), 5.67 – 5.54 (m, 2H), 5.35 – 5.31 (m, 1H), 5.30 (d, *J* = 1.9 Hz, 1H), 4.79 (d, *J* = 11.2 Hz, 1H), 4.71 (d, *J* = 12.0 Hz, 1H), 4.51 – 4.44 (m, 3H), 4.36 (t, *J* = 9.3 Hz, 1H), 4.27 (d, *J* = 11.1 Hz, 1H), 4.24 – 4.19 (m, 2H), 4.04 – 3.94 (m, 3H), 3.87 (t, *J* = 9.7 Hz, 1H), 3.80 (dd, *J* = 10.9, 4.1 Hz, 1H), 3.75 – 3.70 (m, 2H), 2.09 (s, 3H), 1.12 (s, 9H), 0.98 (s, 9H). ^{13}C NMR (151 MHz, $CDCl_3$) δ 169.9, 165.4, 138.8, 138.3, 137.9, 133.7, 130.0, 129.3, 128.7, 128.4, 128.38, 128.31, 128.1, 127.9, 127.77, 127.71, 127.6, 127.4, 105.5 (d, *J* = 222.2 Hz), 99.9, 78.0, 74.6, 74.3, 74.1, 73.6, 73.4, 72.1, 70.3 (d, *J* = 41.0 Hz), 69.5 (d, *J* = 2.0 Hz), 68.8, 68.7, 66.3, 27.6, 27.0, 22.9, 21.1, 20.0. ^{19}F NMR (564 MHz, $CDCl_3$) δ -136.54 (d, *J* = 48.4 Hz). Anomeric configurations confirmed by the following ^{1}H spectral data: ^{1}H NMR (600 MHz, $CDCl_3$) δ 5.60 (dd, *J* = 48.8, 1.7 Hz, 1H, α-fluoromannose), 5.30 (d, *J* = 1.9 Hz, 1H, α-mannose). ESI-HRMS m/z: calc'd for $C_{50}H_{61}FO_{12}Si$ $[M+NH_4]^+$: 918.4260, found: 918.4247.

2-*O*-(2-*O*-acetyl-3,4,6-tri-*O*-benzyl-α-D-mannopyranosyl)-3-*O*-benzyl-4,6-*O*-di-*tert*-butylsilylene-α-D-mannopyranosyl fluoride (17)

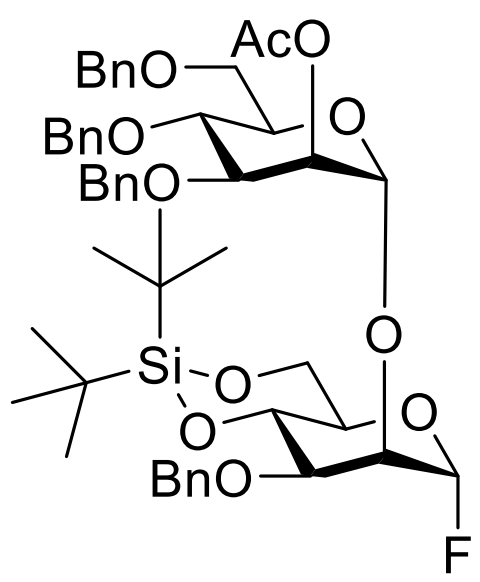

This compound was prepared by following general procedure B, with 2-*O*-acetyl-3,4,6-tri-*O*-benzyl-α-D-mannopyranosyl fluoride **2** (174 mg, 351 µmol) and 2-*O*-trimethylsilyl-3-*O*-benzyl-4,6-*O*-di-*tert*-butylsilylene-α-D-mannopyranosyl fluoride **S10** (162 mg, 334 µmol) with BF_3•OEt_2 (4.74 mg, 4.23 µL, 33.4 µmol) in toluene (6.68 mL). Purification by silica gel column chromatography (5% to 20% EtOAc in hexanes) afforded the desired product **17** (83 mg, 94 µmol, 28%) as a colorless residue. ^{1}H NMR (401 MHz, $CDCl_3$) δ 7.37 – 7.26 (m, 17H), 7.13 (dd, *J* = 7.1, 2.6 Hz, 3H), 5.58 (dd, *J* = 49.8, 1.9 Hz, 1H), 5.55 (dd, *J* = 3.2, 2.0 Hz, 1H), 5.16 (d, *J* = 2.0 Hz, 1H), 4.95 (d, *J* = 12.2 Hz, 1H), 4.82 (d, *J* = 10.6 Hz, 1H), 4.75 (s, 1H), 4.72 (d, *J* = 1.8 Hz, 1H), 4.65 (d, *J* = 12.0 Hz, 1H), 4.51 (d, *J* = 3.3 Hz, 1H), 4.48 (d, *J* = 4.4 Hz, 1H), 4.43 (d, *J* = 10.6 Hz, 1H), 4.25 (t, *J* = 9.5 Hz, 1H), 4.13 (dd, *J* = 9.9, 4.7 Hz, 1H), 4.07 (d, *J* = 3.0 Hz, 1H), 3.96 – 3.87 (m, 3H), 3.86 – 3.72 (m, 5H), 3.72 – 3.64 (m, 3H), 2.12 (s, 3H), 1.10 (s, 9H), 1.01 (s, 9H). ^{13}C NMR (126 MHz, $CDCl_3$) δ 170.0, 138.7, 138.2, 138.2, 138.0, 128.54, 128.52, 128.50, 128.4, 106.9 (d, *J* = 221.8 Hz), 100.2, 78.0, 75.4, 74.9 (d, *J* = 37.9 Hz), 74.6, 74.2, 73.69, 73.60, 72.2, 71.9, 69.6 (d, *J* = 2.2 Hz), 68.9, 68.3, 66.4, 27.6, 27.2, 22.8, 21.2, 20.1. ^{19}F NMR (377 MHz, $CDCl_3$) δ -136.46 (d, *J* = 50.0 Hz). Anomeric configurations confirmed by the following ^{1}H spectral data: ^{1}H NMR (401 MHz, $CDCl_3$) δ 5.58 (dd, *J* = 49.8, 1.9 Hz, 1H, α-fluoromannose), 5.16 (d, *J* = 2.0 Hz, 1H, α-mannose). ESI-HRMS m/z: calc'd for $C_{50}H_{63}FO_{11}Si$ $[M+NH_4]^+$: 904.4461, found: 904.4455.

2,3-*O*-di-(2-*O*-acetyl-3,4,6-tri-*O*-benzyl-α-D-mannopyranosyl)-4,6-*O*-di-*tert*-butylsilylene-α-D-mannopyranosyl fluoride (18)

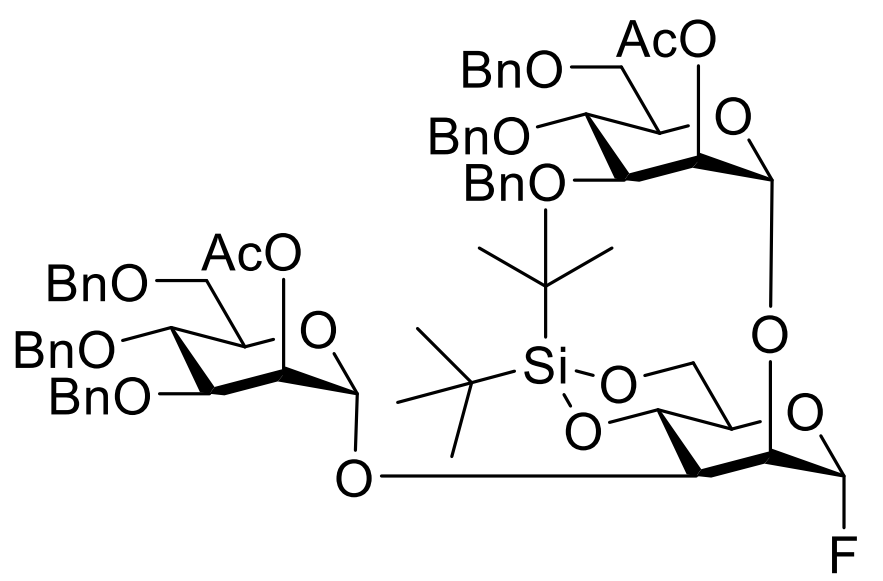

This compound was prepared by following general procedure C, with 2-*O*-acetyl-3,4,6-tri-*O*-benzyl-α-D-mannopyranosyl fluoride **2** (458 mg, 981µmol) and 2,3-*O*-di-trimethylsilyl-4,6-*O*-di-*tert*-butylsilylene-α-D-mannopyranosyl fluoride **S11** (218 mg, 467 µmol) with BF_3•OEt_2 (6.63 mg, 5.76 µL, 46.7 µmol) in toluene (9.34 mL). Purification by silica gel column chromatography (5% to 30% EtOAc in hexanes) afforded the desired product **18** (200 mg, 157 µmol, 34%) as a colorless residue. ^{1}H NMR (500 MHz, $CDCl_3$) δ 7.37 – 7.22 (m, 26H), 7.18 (dd, *J* = 7.7, 2.1 Hz, 2H), 7.14 (dd, *J* = 7.3, 2.4 Hz, 2H), 5.59 – 5.45 (m, 3H), 5.33 (d, *J* = 1.9 Hz, 1H), 5.07 (d, *J* = 1.9 Hz, 1H), 4.83 (dd, *J* = 10.8, 4.9 Hz, 2H), 4.76 (d, *J* = 10.6 Hz, 1H), 4.71 (d, *J* = 11.1 Hz, 1H), 4.67 (d, *J* = 12.0 Hz, 1H), 4.64 (d, *J* = 7.9 Hz, 1H), 4.62 (d, *J* = 9.4 Hz, 1H), 4.53 (d, *J* = 11.1 Hz, 1H), 4.49 (d, *J* = 5.9 Hz, 1H), 4.46 (dd, *J* = 13.2, 8.9 Hz, 3H), 4.21 (t, *J* = 9.5 Hz, 1H), 4.10 – 4.03 (m, 2H), 3.97 (dd, *J* = 9.1, 3.5 Hz, 1H), 3.94 – 3.64 (m, 12H), 2.09 (s, 3H), 1.93 (s, 3H), 1.12 (s, 9H), 0.96 (s, 9H). ^{13}C NMR (126 MHz, $CDCl_3$) δ 169.9, 138.8, 138.5, 138.3, 138.1, 137.8, 128.56, 128.54, 128.49, 128.43, 128.41, 128.3, 128.28, 128.26, 128.1, 128.0, 127.9, 127.89, 127.88, 127.86, 127.77, 127.72, 127.6, 127.5, 106.6 (d, *J* = 222.8 Hz), 100.4, 100.2, 78.8, 77.8, 76.3 (d, *J* = 38.0 Hz), 75.4, 75.0, 74.9, 74.2, 74.1, 73.6, 73.5, 72.4, 72.3, 72.2, 72.01, 69.7 (d, *J* = 1.8 Hz), 69.0, 68.8, 68.5, 68.3, 66.2, 27.6, 27.0, 22.8, 21.1, 20.9, 20.0. ^{19}F NMR (471 MHz, $CDCl_3$) δ -136.07 (d, *J* = 49.3 Hz). Anomeric configurations confirmed by one bond ^{1}H-^{13}C J value analysis in the ^{1}H coupled ^{13}C NMR spectrum: ^{13}C NMR (126 MHz, CDCl3) δ 106.54 (dd, *J* = 223.3, 183.9 Hz,

α-fluoromannose), 100.97 (d, J = 172.5 Hz, α-mannose), 100.79 (d, J = 172.9 Hz, α-mannose). ESI-HRMS m/z: calc'd for $C_{72}H_{87}FO_{17}Si$ $[M+Na]^+$: 1293.5588, found: 1293.5570.

2,3-*O*-carbonate-4-*O*-(2-*O*-acetyl-3,4,6-tri-*O*-benzyl-α-D-mannopyranosyl)-6-*O*-benzoyl-α-D-mannopyranosyl fluoride (19)

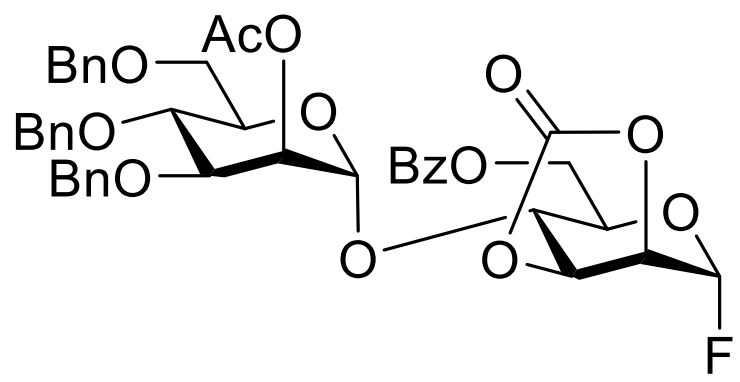

This compound was prepared by following general procedure B, with 2-*O*-acetyl-3,4,6-tri-*O*-benzyl-α-D-mannopyranosyl fluoride **2** (145 mg, 268 µmol) and 2,3-*O*-carbonate-4-*O*-trimethylsilyl-6-*O*-benzoyl-α-D-mannopyranosyl fluoride **S13** (98 mg, 255 µmol) with BF_3•OEt_2 (3.62 mg, 3.23 µL, 25.5 µmol) in toluene (5.10 mL). Purification by silica gel column chromatography (5% to 30% EtOAc in hexanes) afforded the desired product **19** (129 mg, 164 µmol, 64%) as a colorless residue. ^{1}H NMR (401 MHz, $CDCl_3$) δ 8.02 (dd, J = 8.4, 1.3 Hz, 2H), 7.56 (tt, J = 7.4, 1.4 Hz, 1H), 7.44 – 7.38 (m, 2H), 7.33 – 7.23 (m, 13H), 7.16 – 7.08 (m, 2H), 5.87 (d, J = 47.8 Hz, 1H), 5.34 (dd, J = 3.1, 2.0 Hz, 1H), 5.24 (d, J = 2.1 Hz, 1H), 5.00 – 4.94 (m, 1H), 4.83 – 4.77 (m, 2H), 4.73 – 4.65 (m, 2H), 4.55 (d, J = 3.1 Hz, 1H), 4.52 (d, J = 2.2 Hz, 1H), 4.49 – 4.42 (m, 2H), 4.31 (d, J = 12.1 Hz, 1H), 4.20 – 4.15 (m, 2H), 3.94 – 3.83 (m, 2H), 3.68 (ddd, J = 9.2, 4.0, 1.9 Hz, 1H), 3.58 (dd, J = 10.9, 4.1 Hz, 1H), 3.46 (dd, J = 10.9, 1.9 Hz, 1H), 2.15 (s, 3H). ^{13}C NMR (126 MHz, $CDCl_3$) δ 170.5, 166.0, 152.0, 138.3, 138.1, 137.8, 133.5, 129.9, 129.4, 128.6, 128.5, 128.4, 128.1, 128.0, 127.9, 127.8, 127.79, 127.70, 103.3 (d, J = 219.3 Hz), 97.3, 77.6, 77.5, 75.2, 74.0 (d, J = 49.9 Hz), 73.8, 73.5 (d, J = 3.9 Hz), 73.3, 72.2, 70.7, 68.9, 68.3, 68.2 (d, J = 3.9 Hz), 62.4, 21.1. ^{19}F NMR (377 MHz, $CDCl_3$) δ -135.92 (dd, J = 47.9, 4.4 Hz). Anomeric configurations confirmed by the following ^{1}H spectral data: ^{1}H NMR (401 MHz, $CDCl_3$) δ 5.87

(d, J = 47.8, 1H, α-fluoromannose), 5.24 (d, J = 2.1 Hz, 1H, α-mannose). ESI-HRMS m/z: calc'd for $C_{43}H_{43}FO_{13}$ $[M+NH_4]^+$: 804.3025, found: 804.3024.

2,3-*O*-carbonate-4-*O*-(2-*O*-acetyl-3,4,6-tri-*O*-benzyl-α-D-mannopyranosyl)-6-*O*-benzoyl-α-D-glucopyranosyl fluoride (20)

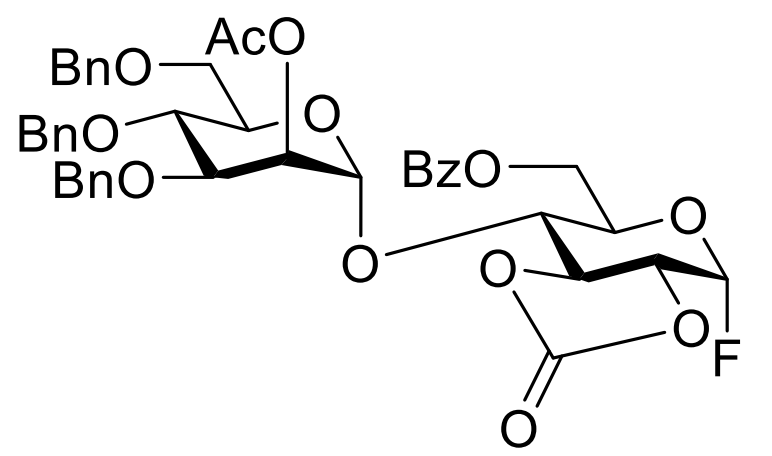

This compound was prepared by following general procedure B, with 2-*O*-acetyl-3,4,6-tri-*O*-benzyl-α-D-mannopyranosyl fluoride **2** (94.4 mg, 191 µmol) and 2,3-*O*-carbonate-4-*O*-trimethylsilyl-6-*O*-benzoyl-α-D-glucopyranosyl fluoride **S16** (69.9 mg, 182 µmol) with BF_3•OEt_2 (1.44 mg, 1.47 µL, 18.2 µmol) in toluene (3.64 mL). Purification by silica gel column chromatography (5% to 30% EtOAc in hexanes) afforded the desired product **20** (98 mg, 125 µmol, 69%) as a colorless residue. ^{1}H NMR (500 MHz, $CDCl_3$) δ 8.02 (dd, J = 8.4, 1.4 Hz, 2H), 7.61 – 7.55 (m, 1H), 7.45 – 7.39 (m, 2H), 7.34 – 7.21 (m, 13H), 7.12 (dd, J = 7.1, 2.6 Hz, 2H), 5.95 (dd, J = 56.0, 2.1 Hz, 1H), 5.43 (t, J = 2.4 Hz, 1H), 5.25 (d, J = 1.9 Hz, 1H), 4.83 – 4.74 (m, 2H), 4.74 – 4.68 (m, 2H), 4.54 (d, J = 3.1 Hz, 1H), 4.51 (d, J = 2.1 Hz, 1H), 4.49 – 4.42 (m, 3H), 4.34 (d, J = 12.1 Hz, 1H), 4.18 – 4.13 (m, 1H), 4.07 (ddd, J = 21.6, 11.6, 2.0 Hz, 1H), 3.87 – 3.83 (m, 2H), 3.70 – 3.63 (m, 1H), 3.57 (dd, J = 10.8, 4.5 Hz, 1H), 3.51 (dd, J = 10.8, 2.0 Hz, 1H), 2.16 (s, 3H). ^{13}C NMR (126 MHz, $CDCl_3$) δ 170.1, 165.8, 152.2, 138.28, 138.24, 137.7, 133.6, 129.8, 129.4, 128.6, 128.5, 128.45, 128.42, 128.2, 128.0, 127.9, 127.77, 127.70, 101.5 (d, J = 236.1 Hz), 97.2, 78.1 (d, J = 3.4 Hz), 77.7, 76.4 (d, J = 26.4 Hz), 75.1, 73.8, 73.5, 73.3, 72.1, 71.0, 68.5, 68.2, 61.7, 21.1. ^{19}F NMR (377 MHz, $CDCl_3$) δ -147.99 (dd, J = 56.2, 21.5 Hz). Anomeric configurations confirmed by the following ^{1}H spectral data: ^{1}H NMR (500 MHz, $CDCl_3$) δ 5.95

(dd, J = 56.0, 2.1 Hz, 1H, α-fluoroglucose), 5.25 (d, J = 1.9 Hz, 1H, α-mannose). ESI-HRMS m/z: calc'd for $C_{43}H_{43}FO_{13}$ $[M+NH_4]^+$: 804.3025, found: 804.3017.

2,3-*O*-carbonate-4,6-*O*-di-(2-*O*-acetyl-3,4,6-tri-*O*-benzyl-α-D-mannopyranosyl)-α-D-glucopyranosyl fluoride (21)

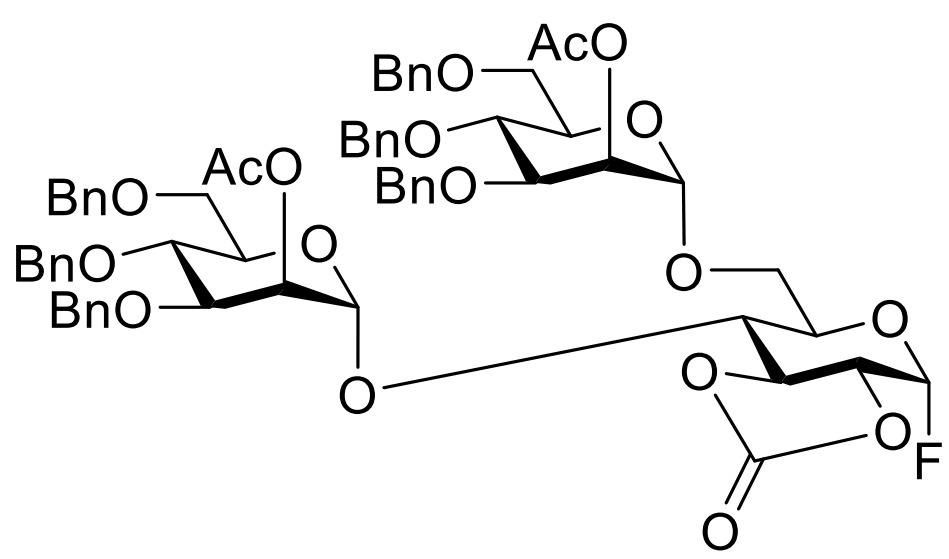

This compound was prepared by following general procedure C, with 2-*O*-acetyl-3,4,6-tri-*O*-benzyl-α-D-mannopyranosyl fluoride **2** (179 mg, 362 μmol) and 2,3-*O*-carbonate-4,6-*O*-di-trimethylsilyl-α-D-glucopyranosyl fluoride **25** (60.8 mg, 172 μmol) with BF_3•OEt_2 (2.45 mg, 2.19 μL, 17.2 μmol) in toluene (3.45 mL). Purification by silica gel column chromatography (5% to 30% EtOAc in hexanes) afforded the desired product **21** (152 mg, 131 μmol, 76%) as a colorless residue. ^{1}H NMR (500 MHz, $CDCl_3$) δ 7.34 – 7.21 (m, 26H), 7.18 (dd, J = 7.5, 2.3 Hz, 2H), 7.16 – 7.12 (m, 2H), 5.87 (dd, J = 56.6, 2.1 Hz, 1H), 5.40 (dd, J = 3.1, 2.0 Hz, 1H), 5.20 (d, J = 2.1 Hz, 1H), 5.15 (dd, J = 2.8, 1.8 Hz, 1H), 4.91 (d, J = 1.8 Hz, 1H), 4.86 (d, J = 10.8 Hz, 1H), 4.80 (d, J = 10.9 Hz, 1H), 4.73 – 4.66 (m, 2H), 4.66 – 4.56 (m, 4H), 4.51 (t, J = 11.3 Hz, 2H), 4.46 (d, J = 10.9 Hz, 1H), 4.43 (d, J = 12.1 Hz, 1H), 4.39 (d, J = 12.3 Hz, 1H), 4.23 (t, J = 9.6 Hz, 1H), 3.96 – 3.82 (m, 7H), 3.80 – 3.62 (m, 7H), 2.14 (s, 3H), 2.13 (s, 3H). ^{13}C NMR (126 MHz, $CDCl_3$) δ 170.5, 170.1, 152.3, 138.4, 138.29, 138.24, 138.0, 137.7, 128.5, 128.49, 128.41, 128.40, 128.3, 128.2, 128.1, 128.03, 128.02, 127.96, 127.92, 127.8, 127.77, 127.76, 127.70, 127.6, 127.5, 101.5 (d, J = 235.3 Hz), 99.0, 97.0, 78.1, 77.9 (d, J = 3.6 Hz), 77.7, 76.2 (d, J = 26.2 Hz), 75.3, 75.2, 75.1, 74.2, 73.7, 73.4, 73.3, 73.2, 72.5, 72.0, 71.9, 70.7, 69.3, 68.8, 68.7, 68.1, 65.3, 21.1, 21.0. ^{19}F NMR (471

MHz, $CDCl_3$) δ -147.73 (dd, *J* = 56.5, 21.7 Hz). Anomeric configurations confirmed by the following ^{1}H spectral data: ^{1}H NMR (500 MHz, $CDCl_3$) δ 5.87 (dd, *J* = 56.6, 2.1 Hz, 1H, α-fluoroglucose), 5.20 (d, *J* = 2.1 Hz, 1H, α-mannose), 4.91 (d, *J* = 1.8 Hz, 1H, α-mannose). ESI-HRMS m/z: calc'd for $C_{65}H_{69}FO_{18}$ $[M+Na]^+$: 1174.4806, found: 1174.4778.

2,3-*O*-carbonate-4-*O*-benzyl-6-*O*-(2-*O*-acetyl-3,4,6-tri-*O*-benzyl-α-D-mannopyranosyl)-α-D-glucopyranosyl fluoride (22)

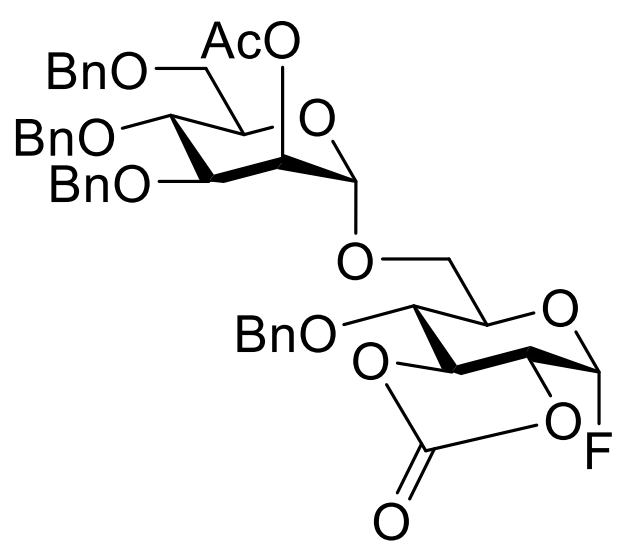

This compound was prepared by following general procedure B, with 2-*O*-acetyl-3,4,6-tri-*O*-benzyl-α-D-mannopyranosyl fluoride **2** (71 mg, 145 μmol) and 2,3-*O*-carbonate-4-*O*-benzyl-6-*O*-trimethylsilyl-α-D-glucopyranosyl fluoride **S20** (51 mg, 138 μmol) with BF_3•OEt_2 (1.95 mg, 1.74 μL, 13.8 μmol) in toluene (2.75 mL). Purification by silica gel column chromatography (5% to 30% EtOAc in hexanes) afforded the desired product **22** (84 mg, 110 μmol, 79%) as a colorless viscous residue. ^{1}H NMR (500 MHz, $CDCl_3$) δ 7.36 – 7.24 (m, 18H), 7.19 (dd, *J* = 7.5, 2.0 Hz, 2H), 5.90 (dd, *J* = 56.7, 1.8 Hz, 1H), 5.14 (dd, *J* = 3.1, 1.9 Hz, 1H), 4.89 (d, *J* = 10.9 Hz, 1H), 4.86 (d, *J* = 1.8 Hz, 1H), 4.83 (d, *J* = 11.4 Hz, 1H), 4.75 (dd, *J* = 11.9, 8.9 Hz, 1H), 4.66 (d, *J* = 11.6 Hz, 1H), 4.64 – 4.59 (m, 2H), 4.51 (d, *J* = 5.0 Hz, 1H), 4.48 (d, *J* = 4.5 Hz, 1H), 4.45 (d, *J* = 12.0 Hz, 1H), 4.01 (ddd, *J* = 21.8, 11.8, 2.0 Hz, 1H), 3.93 – 3.82 (m, 5H), 3.71 – 3.66 (m, 3H), 3.62 – 3.57 (m, 1H), 2.15 (s, 3H). ^{13}C NMR (126 MHz, $CDCl_3$) δ 170.6, 152.8, 138.4, 138.2, 138.0, 136.5, 128.7, 128.6, 128.5, 128.48, 128.43, 128.2, 128.09, 128.07, 128.02, 127.9, 127.86, 127.80, 101.7 (d, *J* = 234.9 Hz), 98.4, 79.1 (d, *J* = 3.2 Hz), 76.6 (d, *J* = 26.5 Hz), 75.4, 75.3, 74.3, 73.8, 73.5,

73.0, 72.1, 71.9, 69.1, 68.7, 64.9, 21.2. ^{19}F NMR (471 MHz, $CDCl_3$) δ -147.72 (dd, *J* = 56.6, 21.7 Hz). Anomeric configurations confirmed by the following ^{1}H spectral data: ^{1}H NMR (500 MHz, $CDCl_3$) δ 5.90 (dd, *J* = 56.7, 1.8 Hz, 1H, α-fluoroglucose), 4.86 (d, *J* = 1.8 Hz, 1H, α-mannose). ESI-HRMS m/z: calc'd for $C_{43}H_{45}FO_{12}$ $[M+Na]^+$: 795.2787, found: 795.2780.

Methyl-2,3-di-*O*-benzyl-4-*O*-[2-*O*-acetyl-3-*O*-(2-*O*-acetyl-3,4,6-tri-*O*-benzyl-α-D-mannopyranosyl)-4,6-*O*-di-*tert*-butylsilylene-α-D-glucopyranosyl]-6-*O*-benzoyl-α-D-glucopyranoside (24)

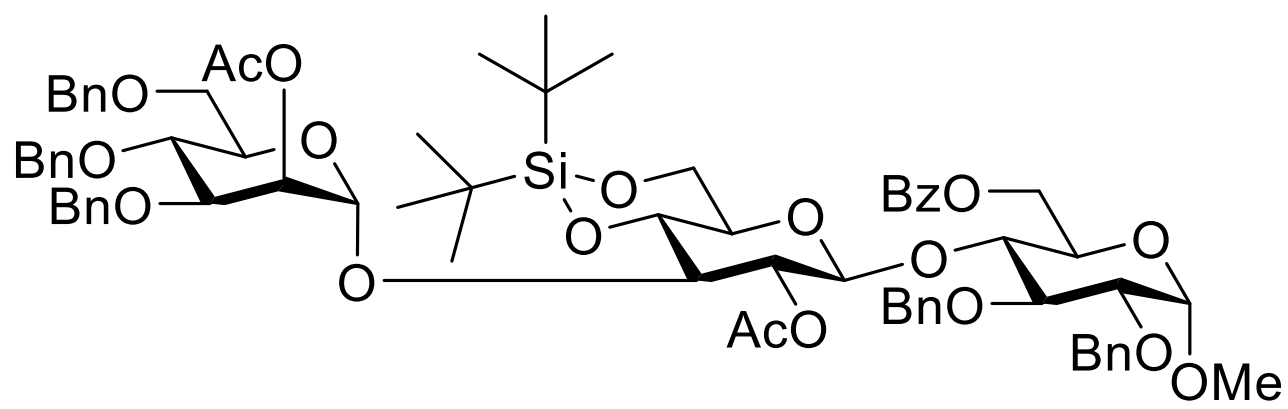

This compound was prepared by following general procedure D, with 2-*O*-acetyl-3,4,6-tri-*O*-benzyl-α-D-mannopyranosyl fluoride **2** (34 mg, 69 µmol), 2-*O*-acetyl-3-*O*-trimethylsilyl-4,6-*O*-di-*tert*-butylsilylene-α-D-glucopyranosyl fluoride **23** (30 mg, 69 µmol), and methyl-2,3-di-O-benzyl-4-O-trimethylsilyl-6-O-benzoyl-α-D-glucopyranoside (38 mg, 69 µmol) **9** with $B(C_6F_5)_3$ (3.5 mg, 6.9 µmol) in toluene (2.75 mL). Purification by silica gel column chromatography (5% to 30% EtOAc in hexanes) afforded the desired product **24** (52 mg, 40 µmol, 58%) as a colorless viscous residue. **24** (45 mg, 35 µmol, 50%) mixed with 7 mg of compound **9** repurification of **24** gave the following spectra ^{1}H NMR (500 MHz, $CDCl_3$) δ 8.05 – 7.99 (m, 3H), 7.57 (q, *J* = 7.4 Hz, 1H), 7.44 (t, *J* = 6.9 Hz, 3H), 7.39 – 7.21 (m, 32H), 7.11 (dd, *J* = 7.4, 2.1 Hz, 2H), 5.46 (d, *J* = 2.1 Hz, 1H), 5.32 (d, *J* = 2.0 Hz, 1H), 5.03 – 4.42 (m, 18H), 4.36 (dd, *J* = 11.9, 5.4 Hz, 1H), 3.95 – 3.59 (m, 14H), 3.52 (ddd, *J* = 13.0, 9.6, 3.6 Hz, 2H), 3.37 (s, 3H), 3.12 (td, *J* = 9.8, 5.0 Hz, 1H), 2.08 (s, 3H), 2.01 (s, 3H), 1.03 (s, 9H), 0.85 (s, 9H). ^{13}C NMR (126 MHz, $CDCl_3$) δ 170.0, 169.5, 166.1, 139.1, 138.8, 138.4, 138.2, 138.1, 133.4, 129.8, 129.6, 128.6, 128.5, 128.49, 128.42, 128.41,

128.3, 128.2, 128.1, 127.9, 127.5, 127.1, 101.1, 99.1, 98.0, 79.8, 79.6, 79.0, 78.3, 78.0, 77.7, 75.2, 74.9, 73.5, 72.2, 72.15, 72.10, 70.4, 68.8, 68.5, 68.4, 66.0, 63.2, 27.5, 26.9, 22.6, 21.15, 21.12, 19.8. Anomeric configurations confirmed by one bond ^{1}H-^{13}C J value analysis in the ^{1}H coupled ^{13}C NMR spectrum: ^{13}C NMR (126 MHz, $CDCl_3$) δ 101.1 (d, *J* = 162.2 Hz, β-glucose), 99.1 (d, *J* = 172.4 Hz, α-mannose), 98.06 (d, *J* = 168.5 Hz, α-glucose). ESI-HRMS m/z: calc'd for $C_{73}H_{88}O_{19}Si$ $[M+Na]^+$: 1314.6027, found: 1314.6005.

V. References

1. K. R. Harale, C. Sunil, G. C. Sati, A. Q. Ansel, T. M. Emmel, J. L. Martin, T. E. Spiller and J. Montgomery. Development, Scope, and Catalyst Selection in Boron-Catalyzed Iterative Glycosylation. *J. Am. Chem. Soc*. 2025, **147**, 46125-46135.
2. J. A. Nicasio, S. Steinberg, B. Inés, and M. Alcarazo. Tuning the Lewis Acidity of Boranes in Frustrated Lewis Pair Chemistry: Implications for the Hydrogenation of Electron-Poor Alkenes. *Chem. Eur. J.* 2013, **19**, 11016-11020.
3. G. C. Sati, J. L. Martin, Y. Xu, T. Malakar, P. M. Zimmerman, and J. Montgomery. Fluoride Migration Catalysis Enables Simple, Stereoselective, and Iterative Glycosylation. *J. Am. Chem. Soc*. 2020, **142**, 7235-7242.
4. J. L. Martin, G. C. Sati, T. Malakar, J. Hatt, P. M. Zimmerman, and J. Montgomery. Glycosyl Exchange of Unactivated Glycosidic Bonds: Suppressing or Embracing Side Reactivity in Catalytic Glycosylations. *J. Org. Chem*. 2022, **87**, 5817-5826.
5. J. Lv, J. J. Zhu, Y. Liu, and H. Dong. Regioselective Sulfonylation/Acylation of Carbohydrates Catalyzed by FeCl3 Combined with Benzoyltrifluoroacetone and Its Mechanism Study. *J. Org. Chem.* 2020, **85**, 3307-3319.
6. J. C. Lopez, P. Bernal-Albert, C. Uriel, S. Valverde, and A. M. Gomez. IPy_2BF_4/HF-Pyridine: A New Combination of Reagents for the Transformation of Partially Unprotected Thioglycosides and n-Pentenyl Glycosides to Glycosyl Fluorides. *J. Org. Chem.* 2007, **72**, 10268-10271.

VI. Copies of NMR Spectra

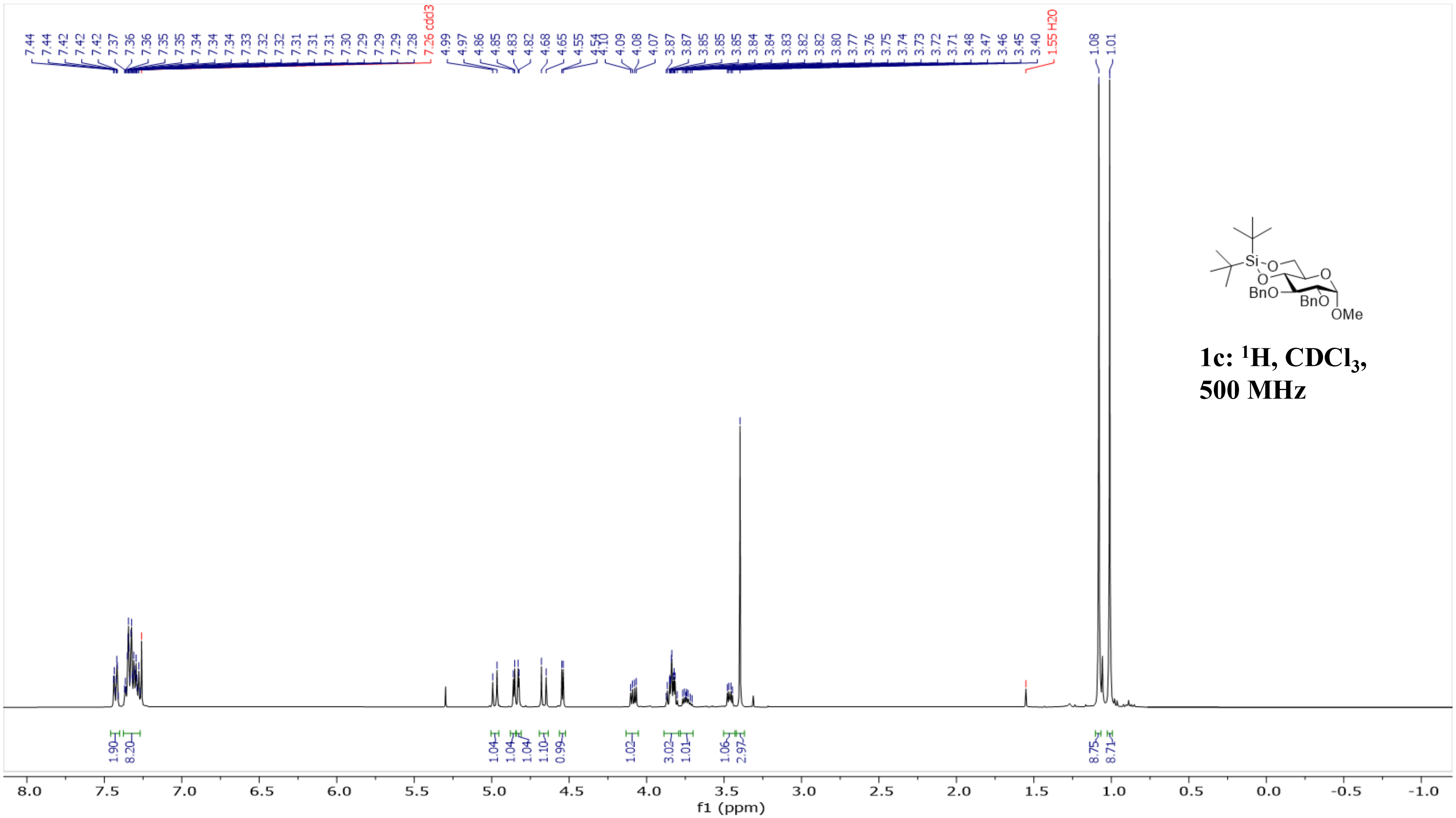

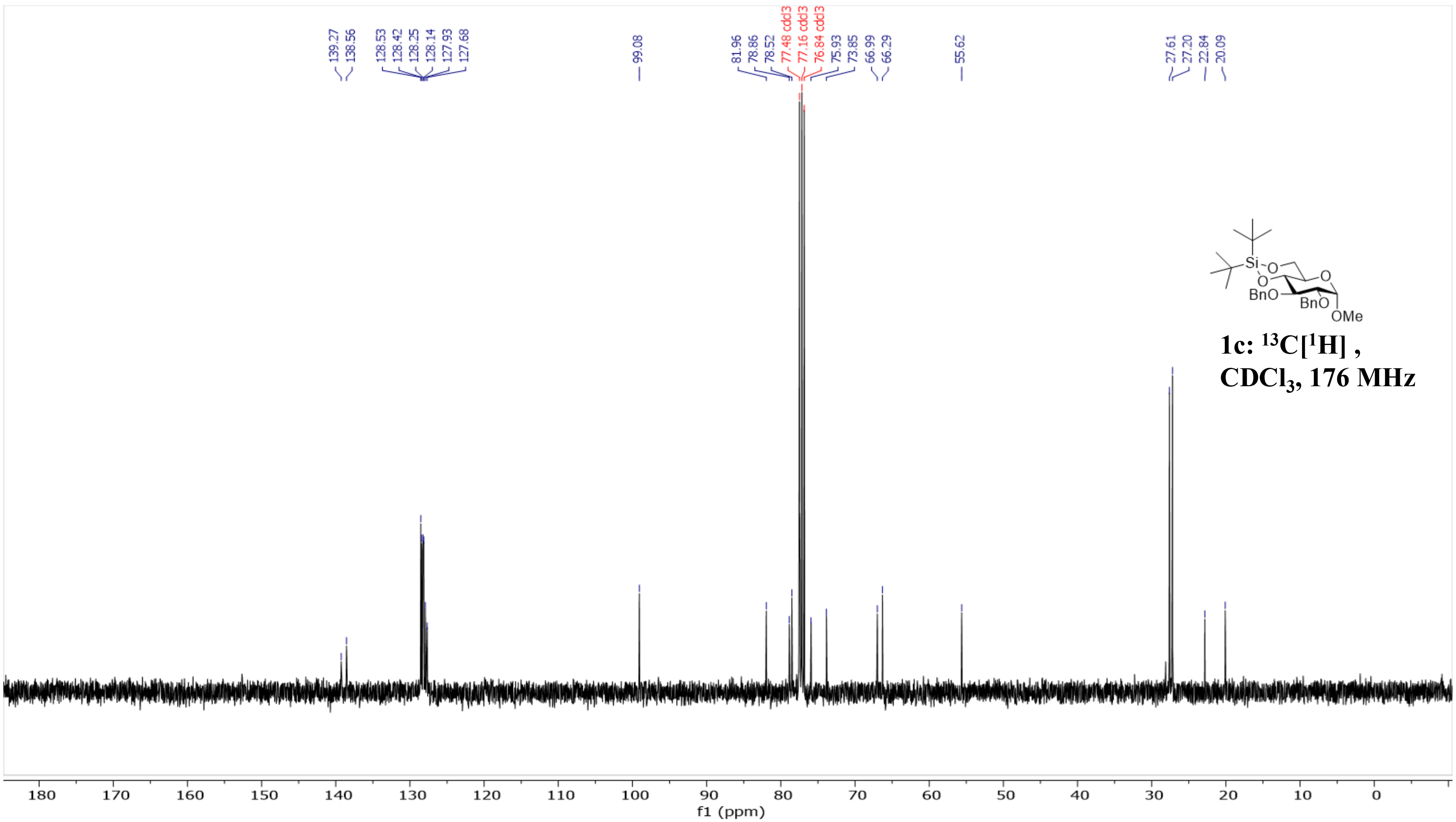
139.27
138.56
128.53
128.42
128.25
128.14
127.93
127.68
99.08
81.96
78.86
78.52
77.48 cdcl3
77.16 cdcl3
76.84 cdcl3
75.93
73.85
66.99
66.29
55.62
27.61
27.20
22.84
20.09
Si
O
O
O
BnO
BnO
OMe
1c: $^{13}C[^{1}H]$,
$CDCl_3$, 176 MHz
180 170 160 150 140 130 120 110 100 90 80 70 60 50 40 30 20 10 0
f1 (ppm)

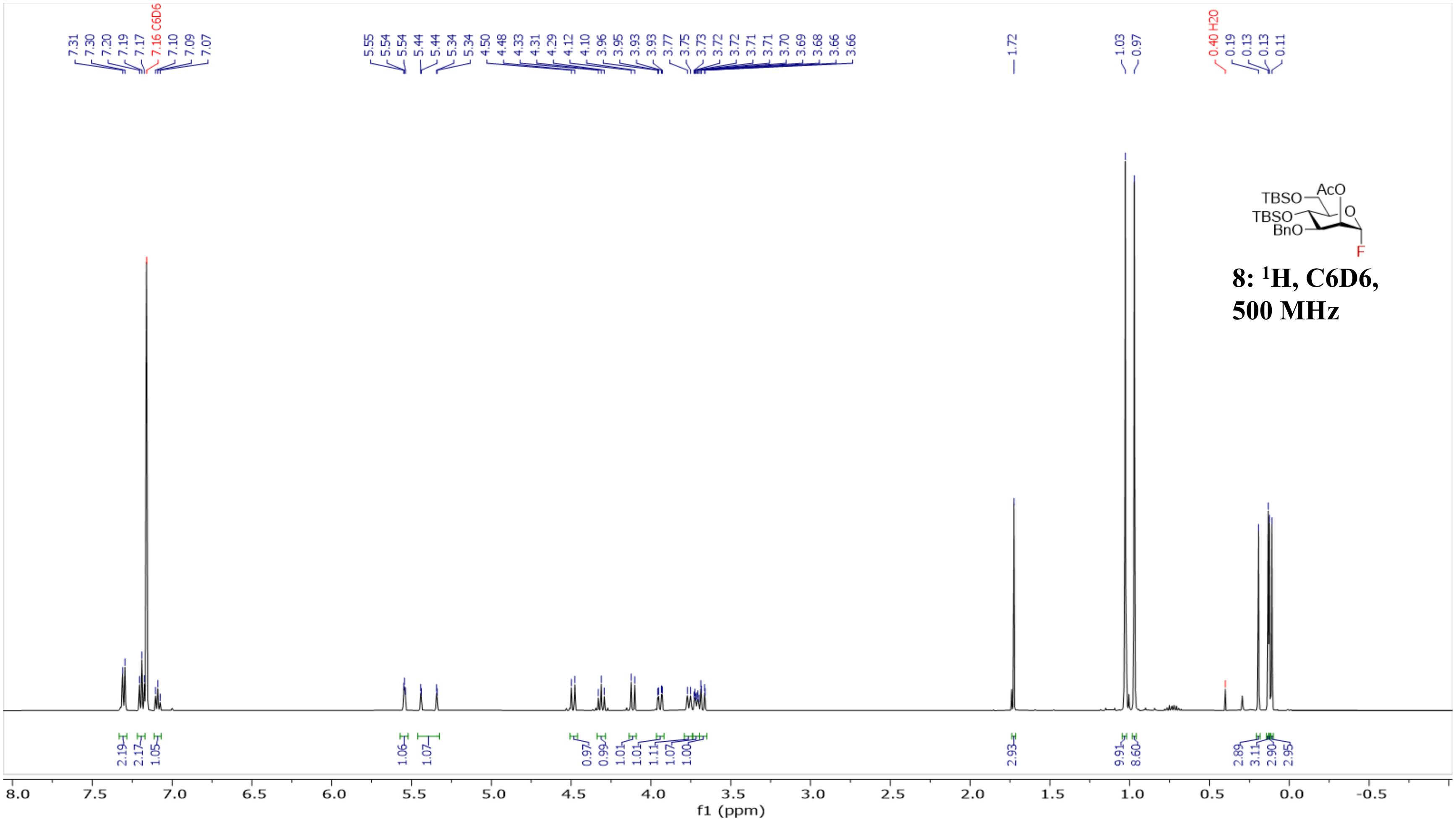
TBSO
AcO
O
TBSO
BnO
F
8: ¹H, C6D6,
500 MHz
7.31 7.30 7.20 7.19 7.17 7.16 C6D6 7.10 7.09 7.07
5.55 5.54 5.54 5.44 5.44 5.34 5.34 4.50 4.48 4.33 4.31 4.29 4.12 4.10 3.96 3.95 3.93 3.93 3.77 3.75 3.73 3.72 3.72 3.71 3.71 3.70 3.69 3.68 3.66 3.66
1.72
1.03 0.97
0.40 H2O 0.19 0.13 0.13 0.11
2.19 2.17 1.05 1.06 1.07 0.97 0.99 1.01 1.01 1.11 1.07 1.00 2.93 9.91 8.60 2.89 3.11 2.90 2.95
8.0 7.5 7.0 6.5 6.0 5.5 5.0 4.5 4.0 3.5 3.0 2.5 2.0 1.5 1.0 0.5 0.0 -0.5
f1 (ppm)

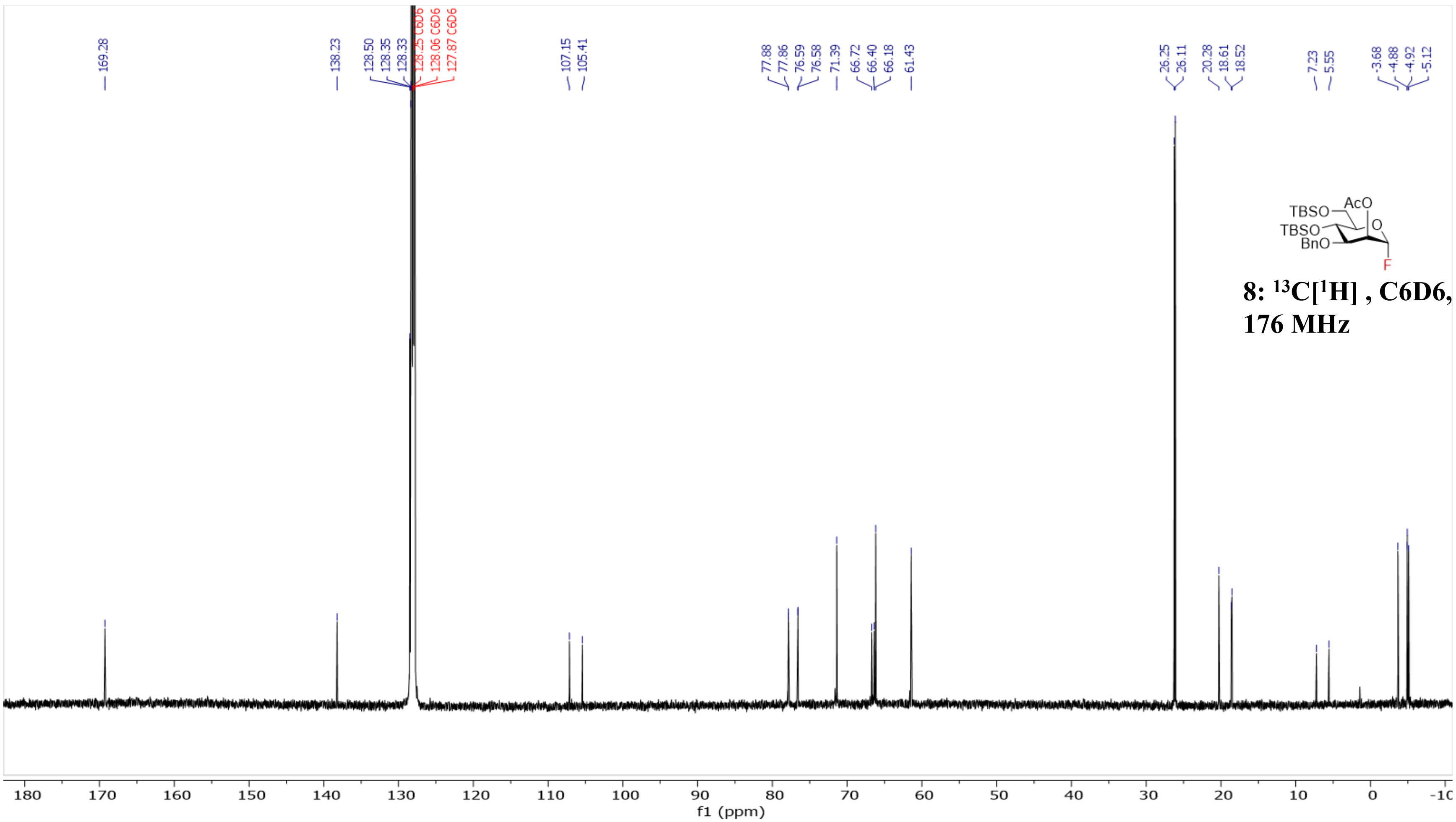

8: $^{13}C[^{1}H]$, C6D6, 176 MHz

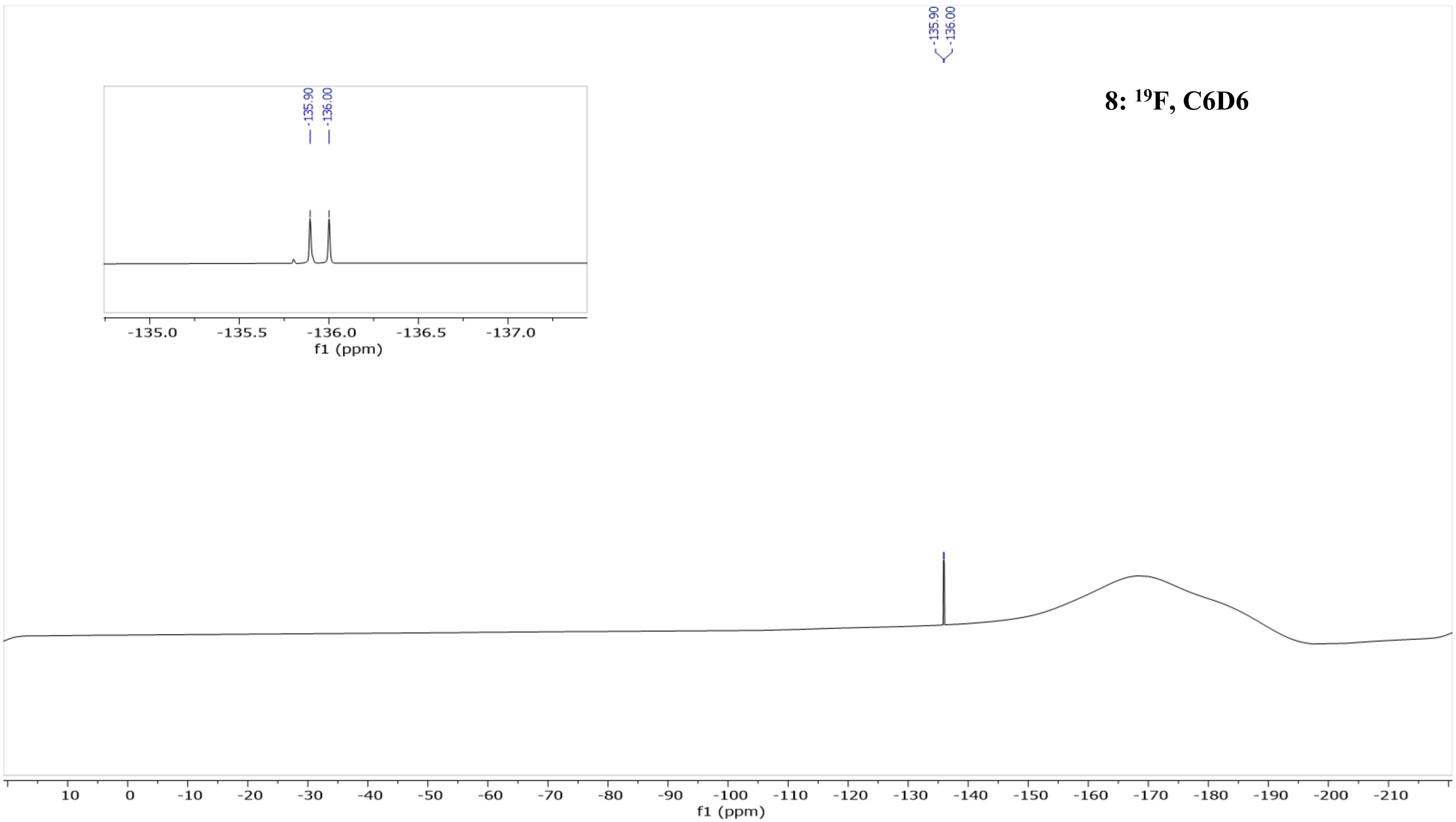

8: ¹⁹F, C6D6
-135.90
-136.00
-135.0
-135.5
-136.0
-136.5
-137.0
f1 (ppm)
10
0
-10
-20
-30
-40
-50
-60
-70
-80
-90
-100
-110
-120
-130
-140
-150
-160
-170
-180
-190
-200
-210
f1 (ppm)

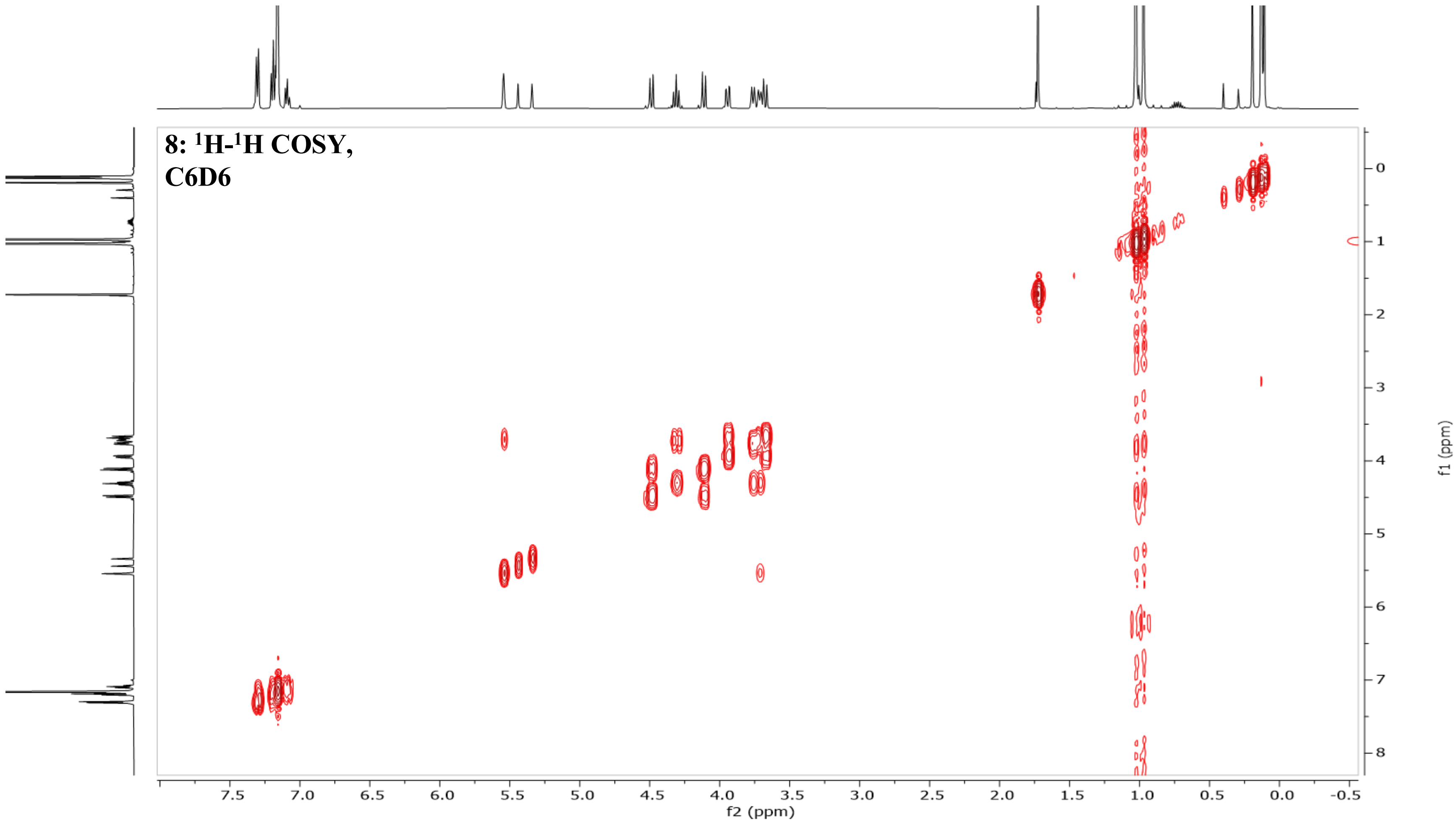
8: 1H-1H COSY,
C6D6
f2 (ppm)
f1 (ppm)

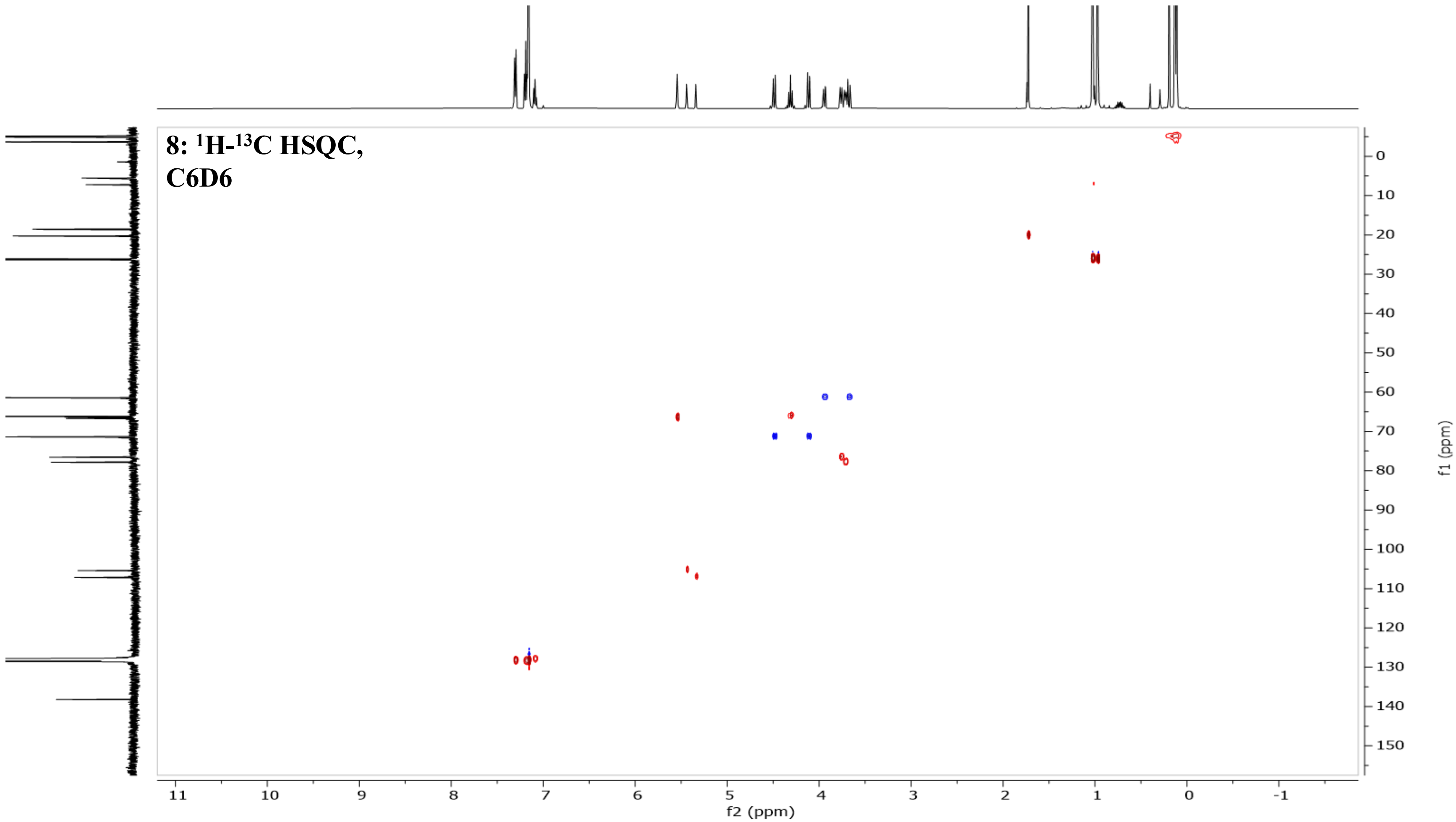
8: 1H-13C HSQC,
C6D6
f2 (ppm)
f1 (ppm)

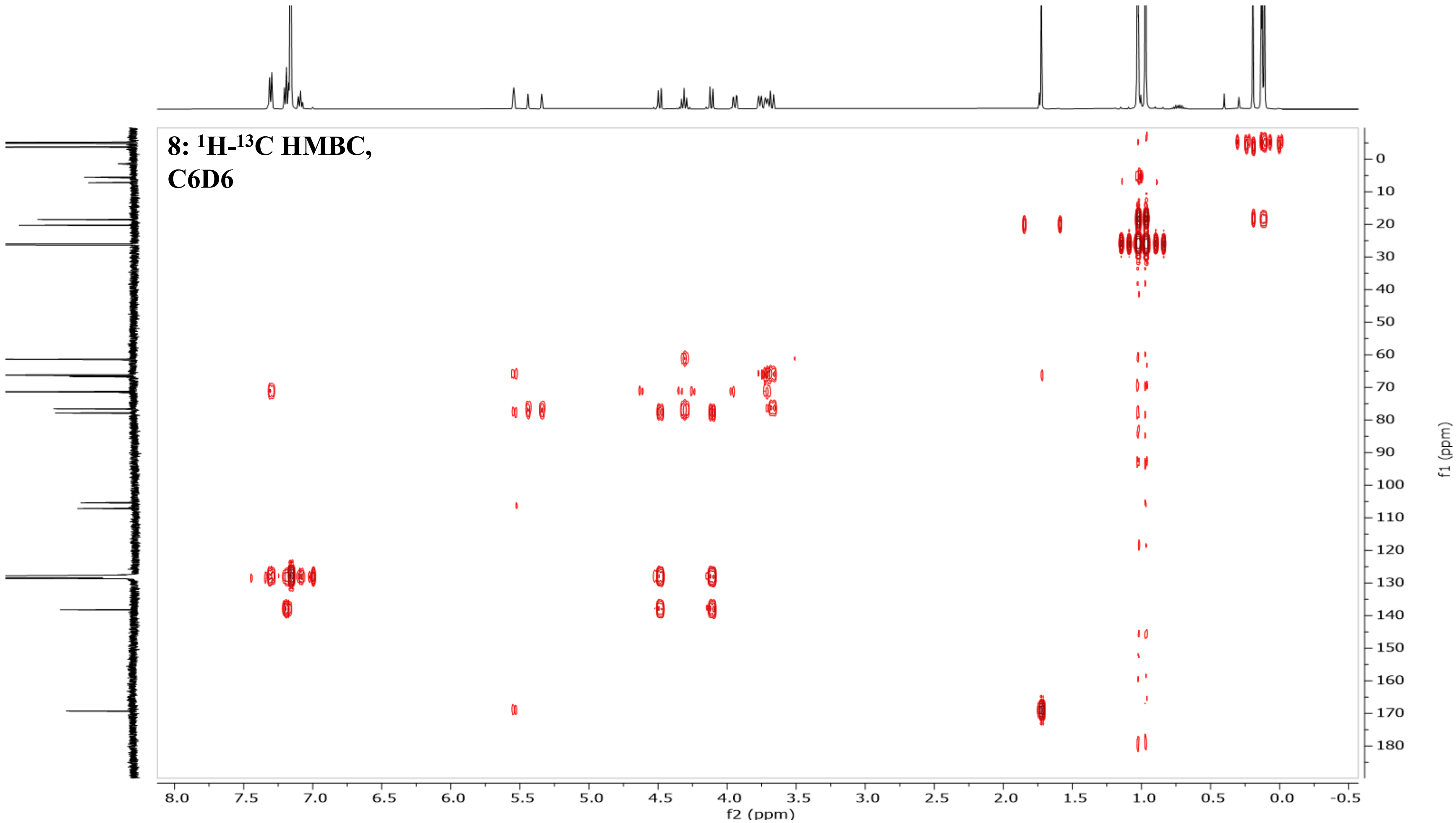
8: 1H-13C HMBC,
C6D6
8.0 7.5 7.0 6.5 6.0 5.5 5.0 4.5 4.0 3.5 3.0 2.5 2.0 1.5 1.0 0.5 0.0 -0.5
f2 (ppm)
0 10 20 30 40 50 60 70 80 90 100 110 120 130 140 150 160 170 180
f1 (ppm)

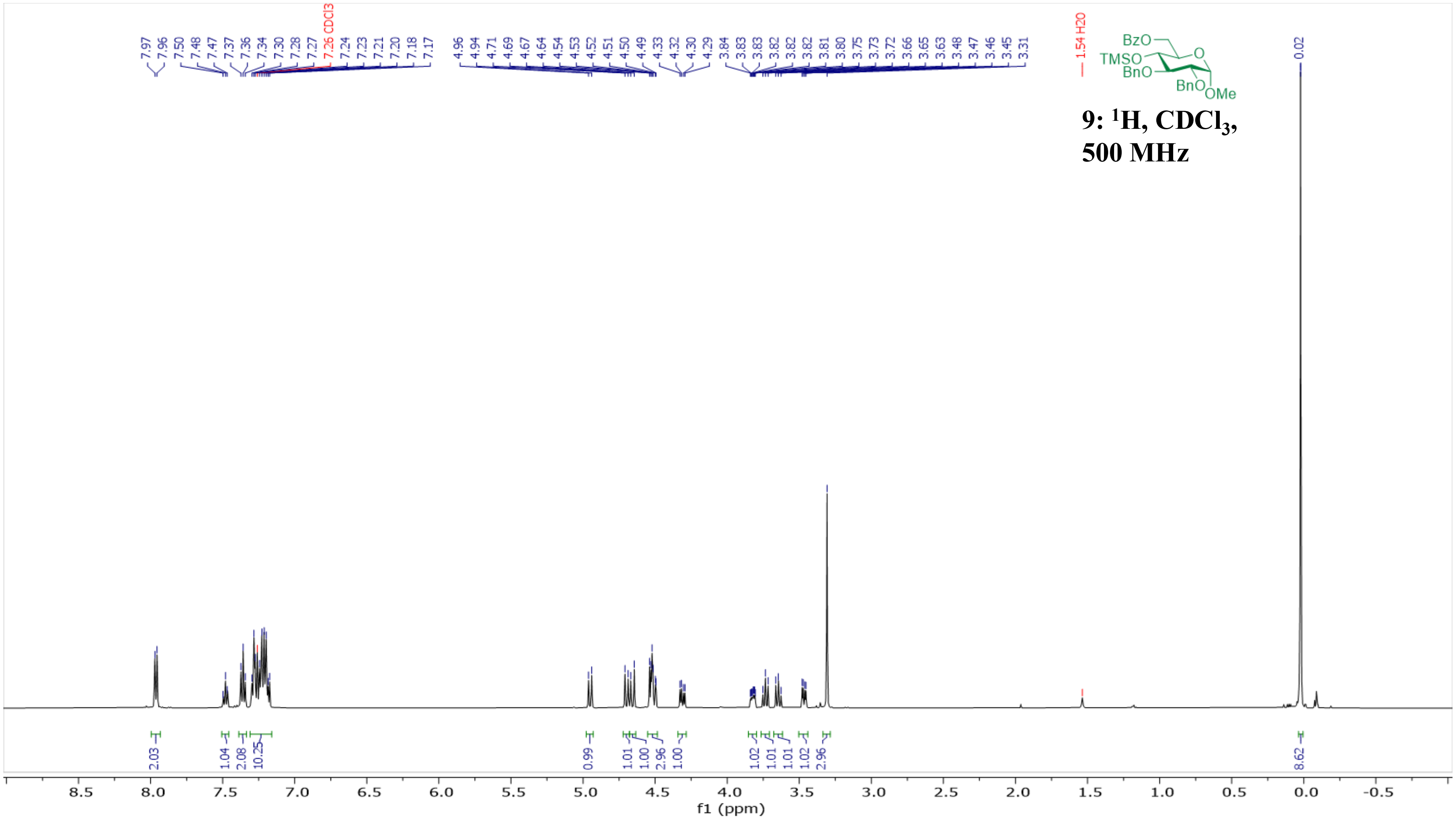

9: ¹H, CDCl3, 500 MHz
BzO
TMSO
BnO
BnO
OMe
f1 (ppm)

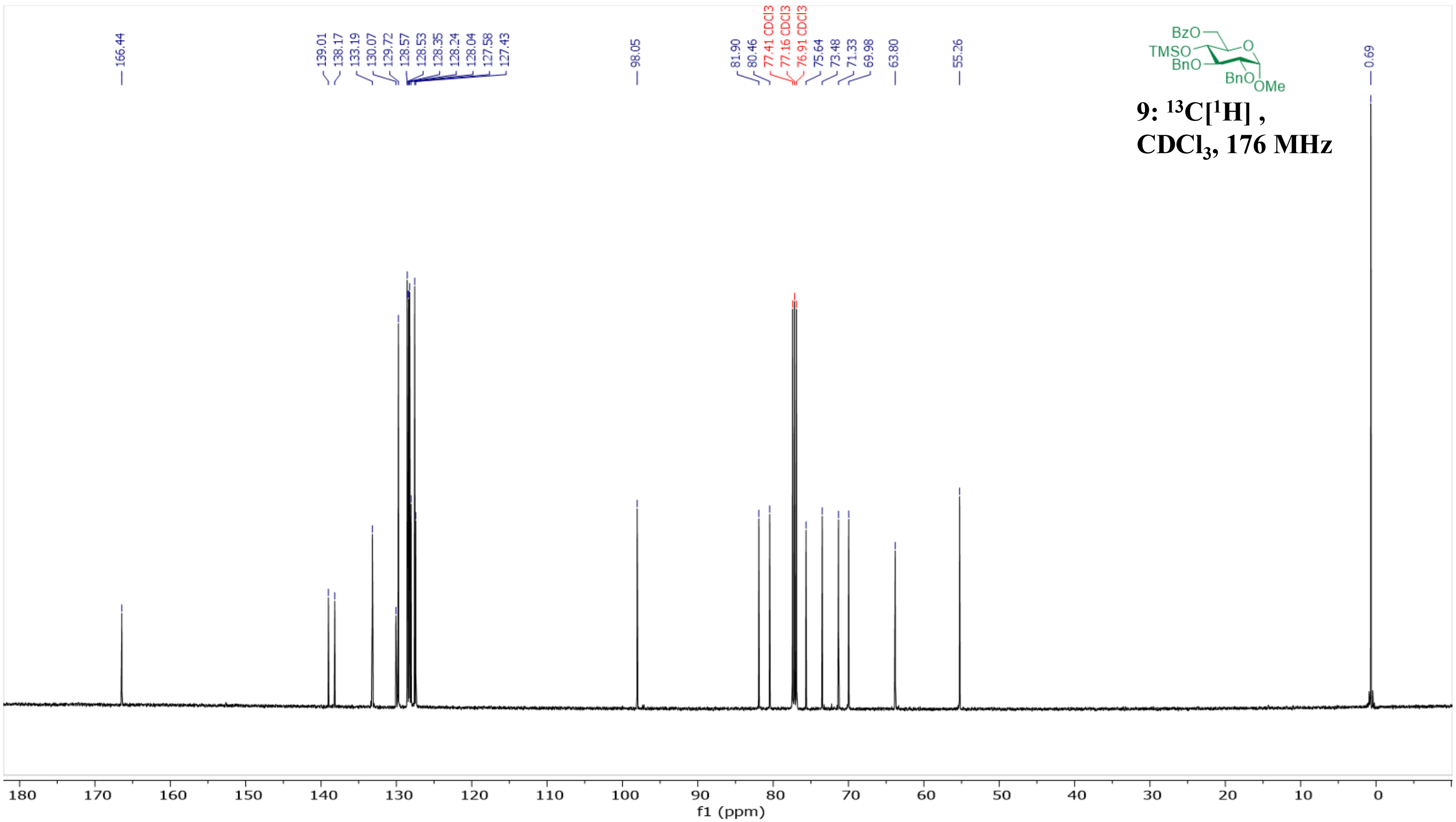

9: $^{13}C[^{1}H]$, $CDCl_3$, 176 MHz

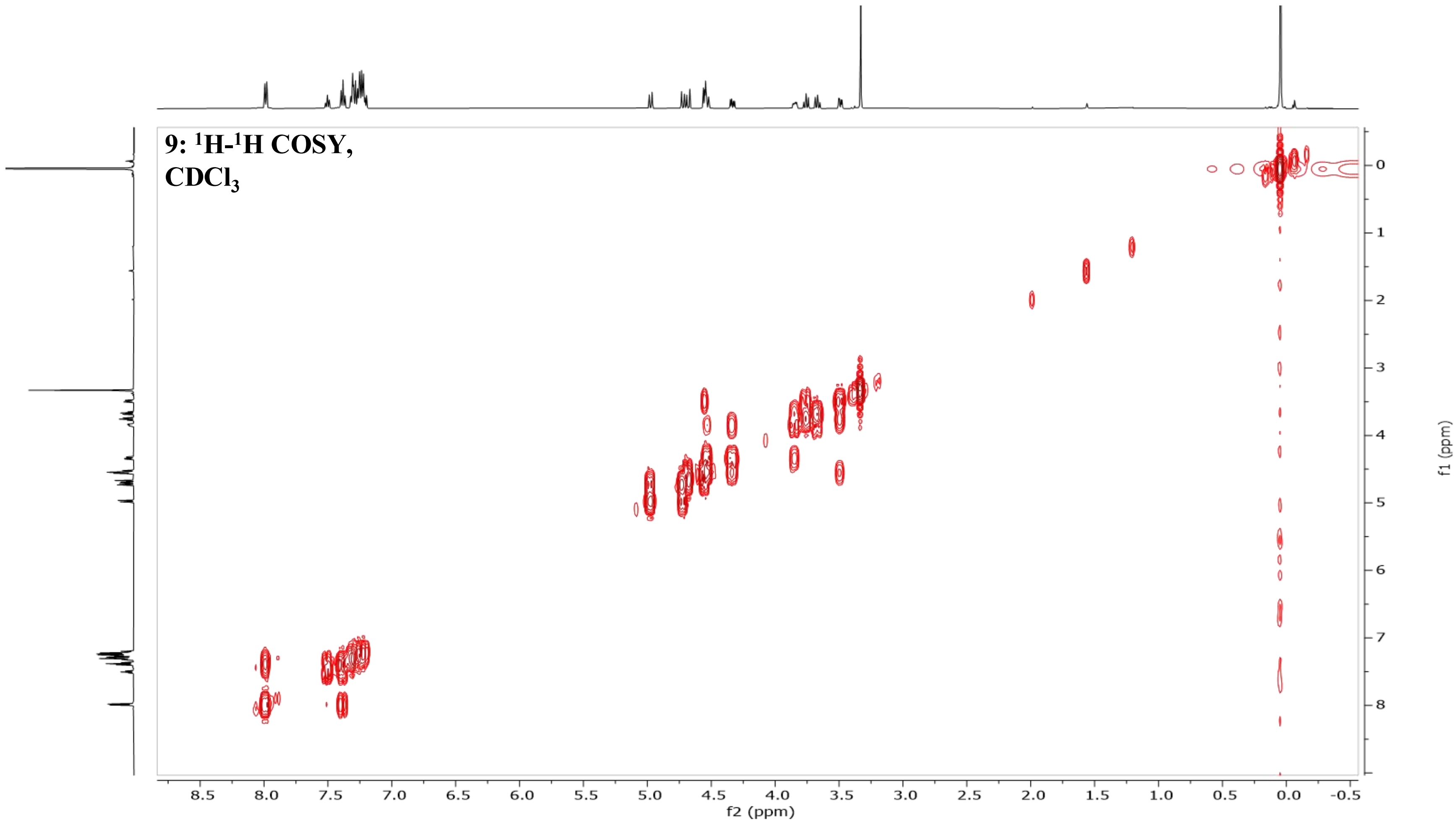
9: ¹H-¹H COSY,
CDCl₃
8.5 8.0 7.5 7.0 6.5 6.0 5.5 5.0 4.5 4.0 3.5 3.0 2.5 2.0 1.5 1.0 0.5 0.0 -0.5
f2 (ppm)
0 1 2 3 4 5 6 7 8
f1 (ppm)

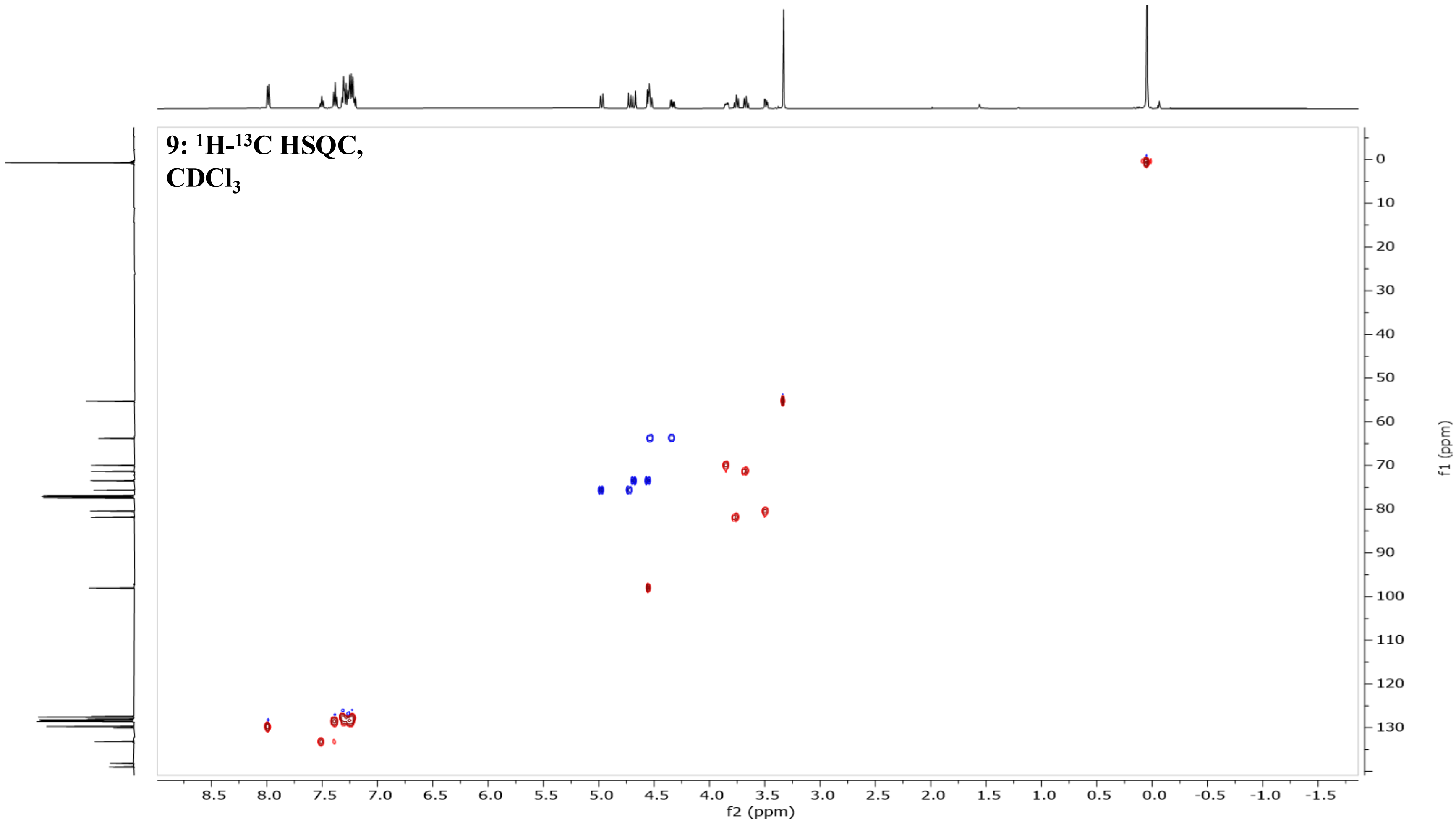
9: ^{1}H-^{13}C HSQC,
$CDCl_3$
f2 (ppm)
f1 (ppm)

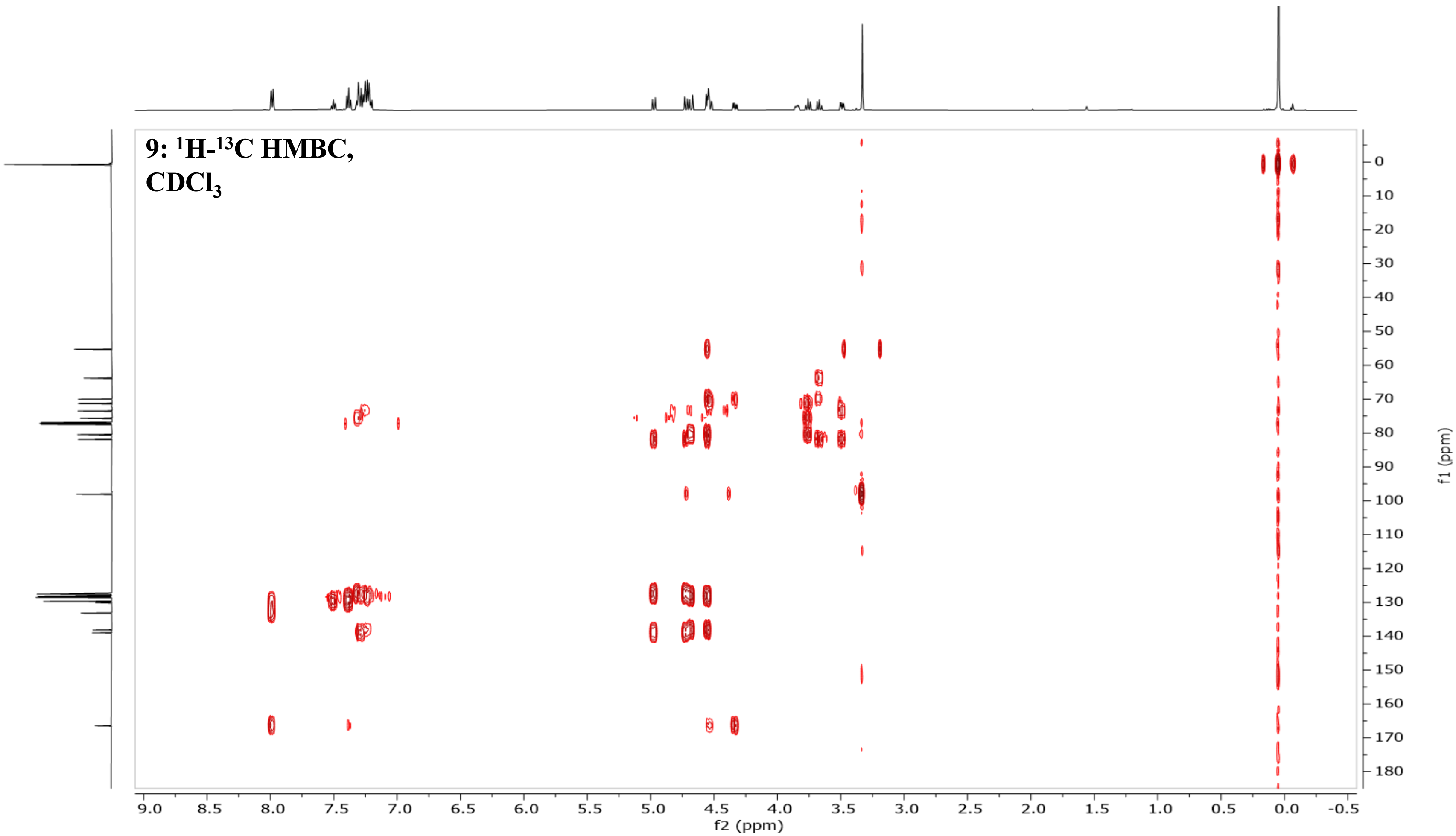
9: ¹H-¹³C HMBC,
CDCl3
f2 (ppm)
f1 (ppm)

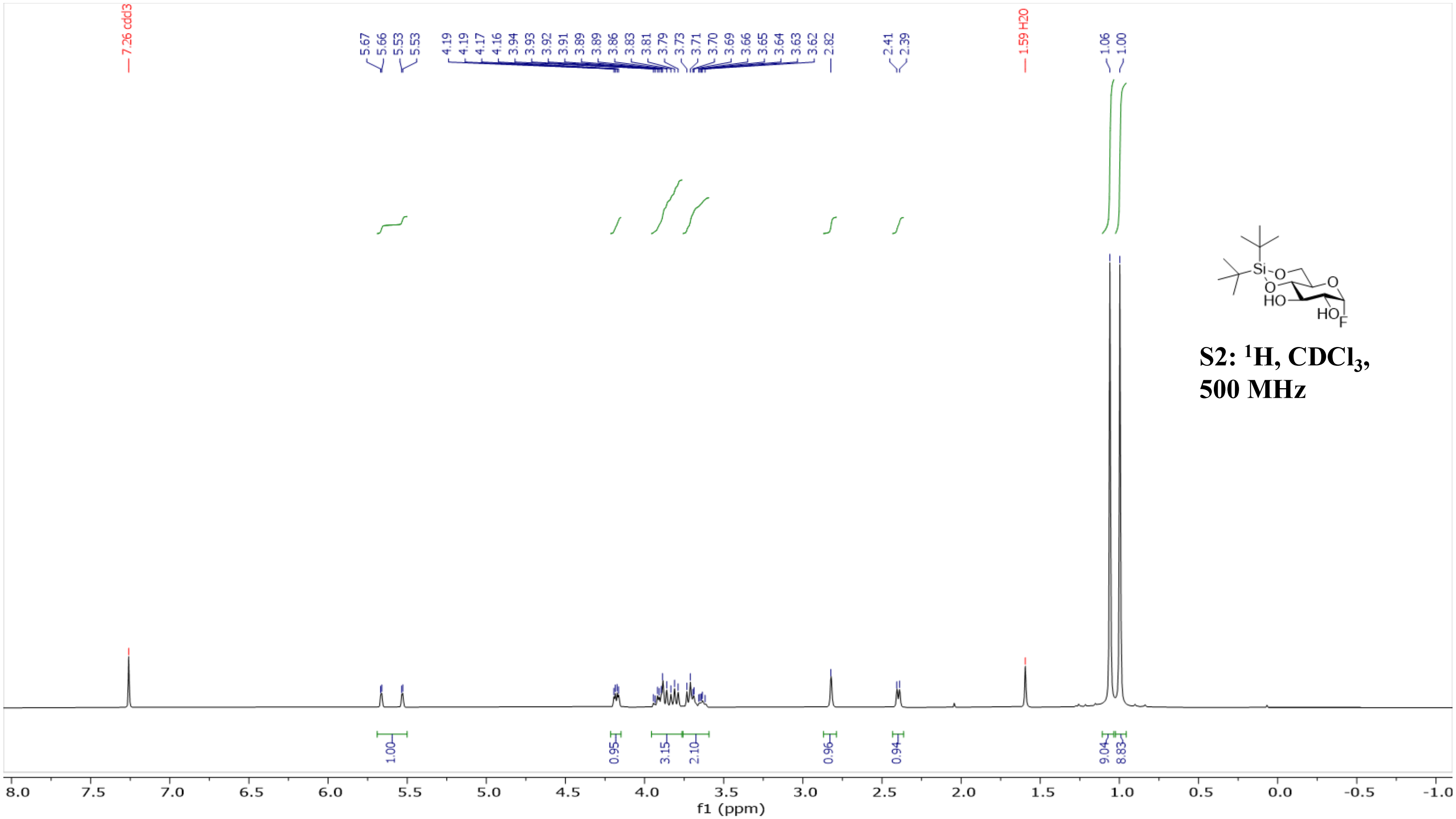

S2: ^{1}H, $CDCl_3$, 500 MHz

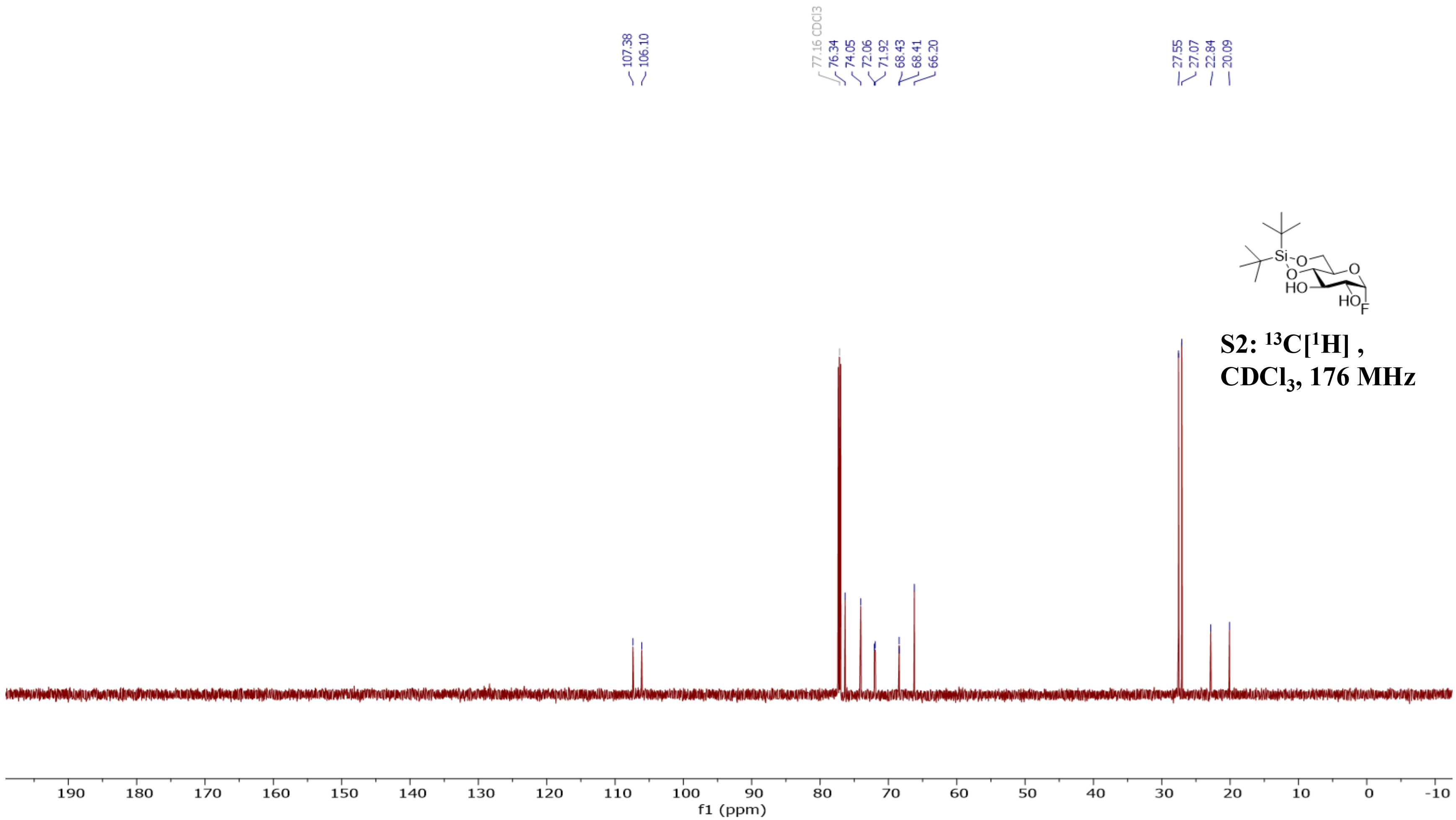

S2: ^{13}C[^{1}H] , CDCl$_3$, 176 MHz

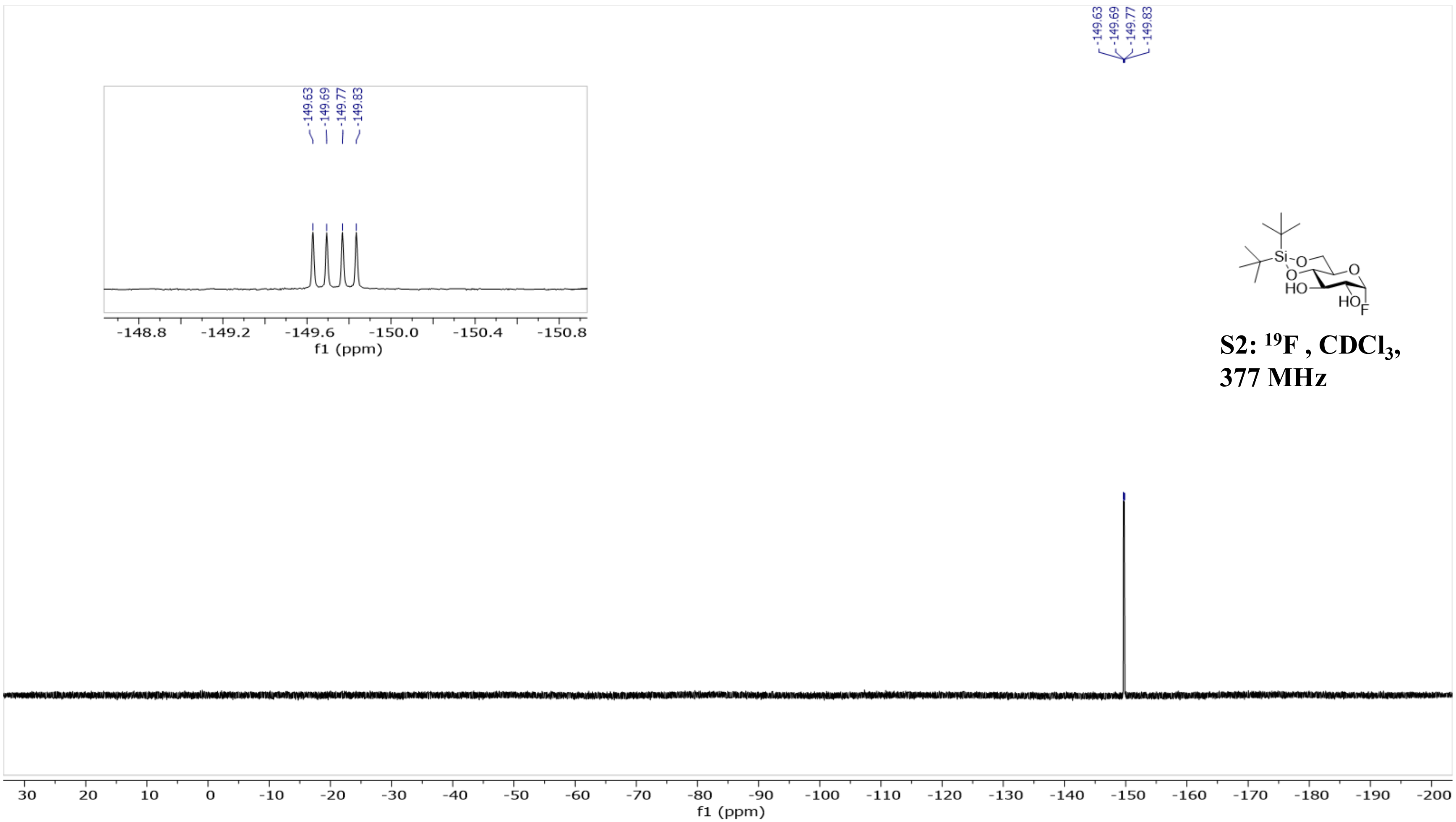

S2: ^{19}F , $CDCl_3$, 377 MHz

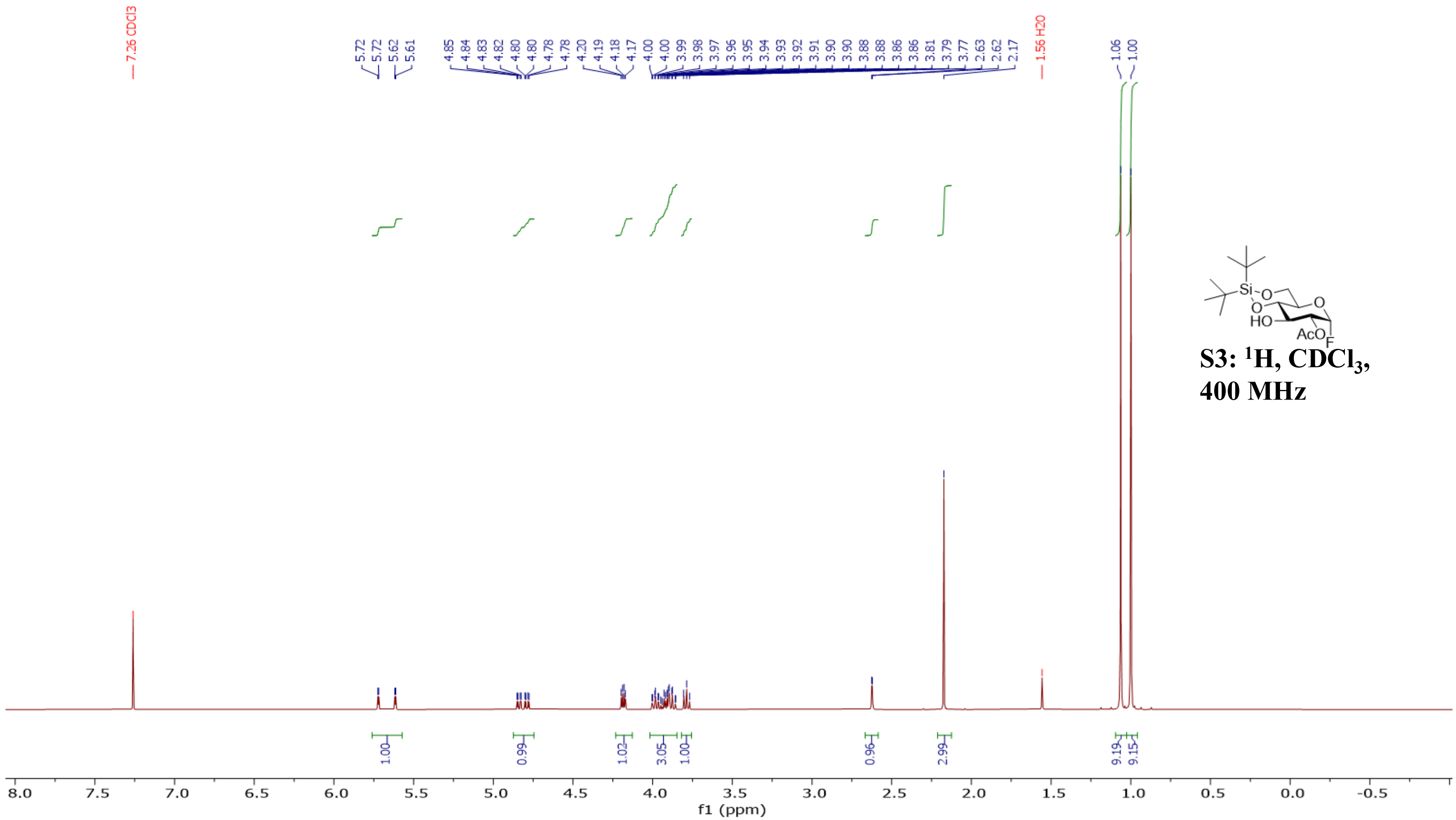

S3: ^{1}H, $CDCl_3$, 400 MHz

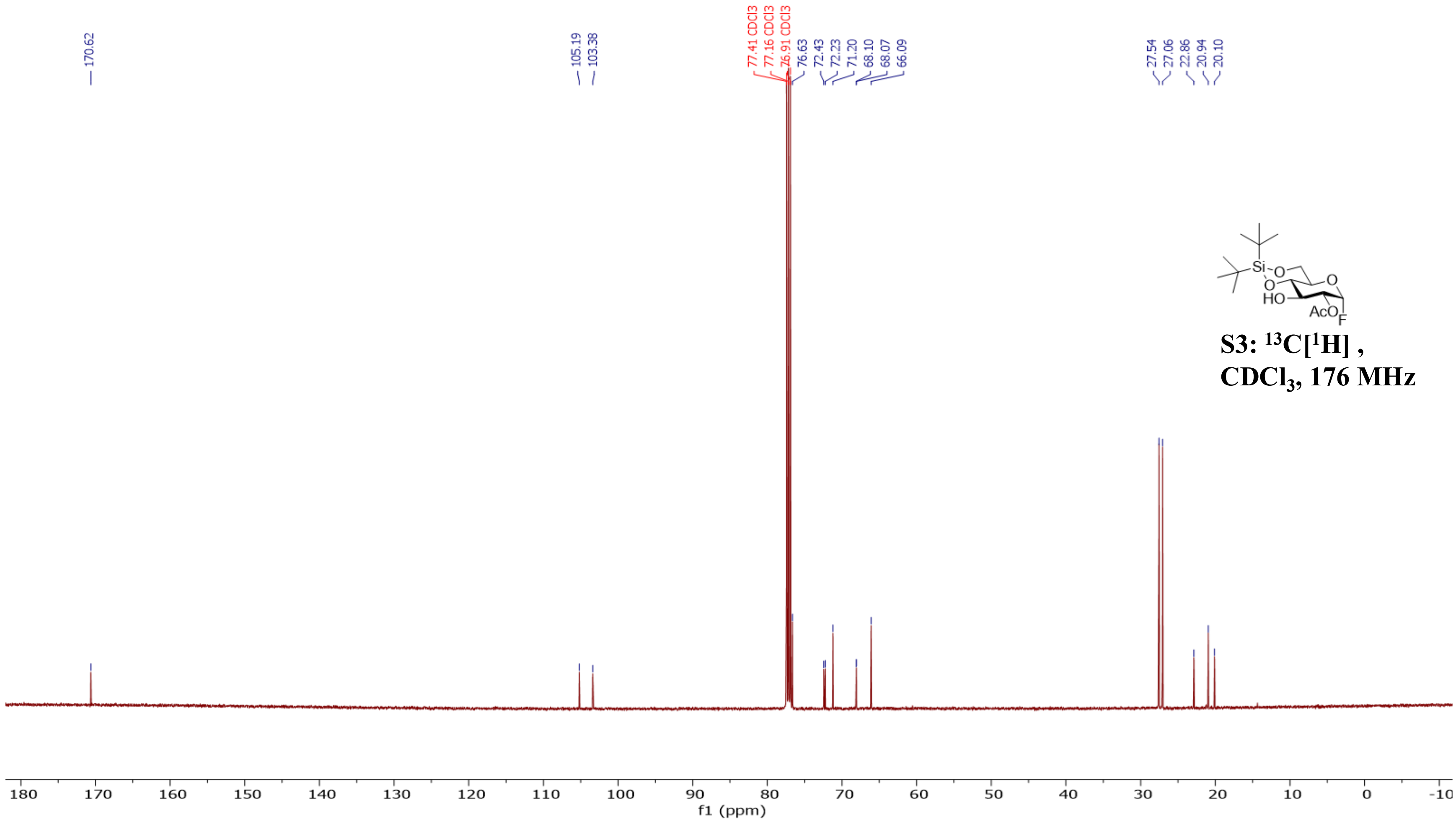

S3: $^{13}C[^{1}H]$, $CDCl_3$, 176 MHz

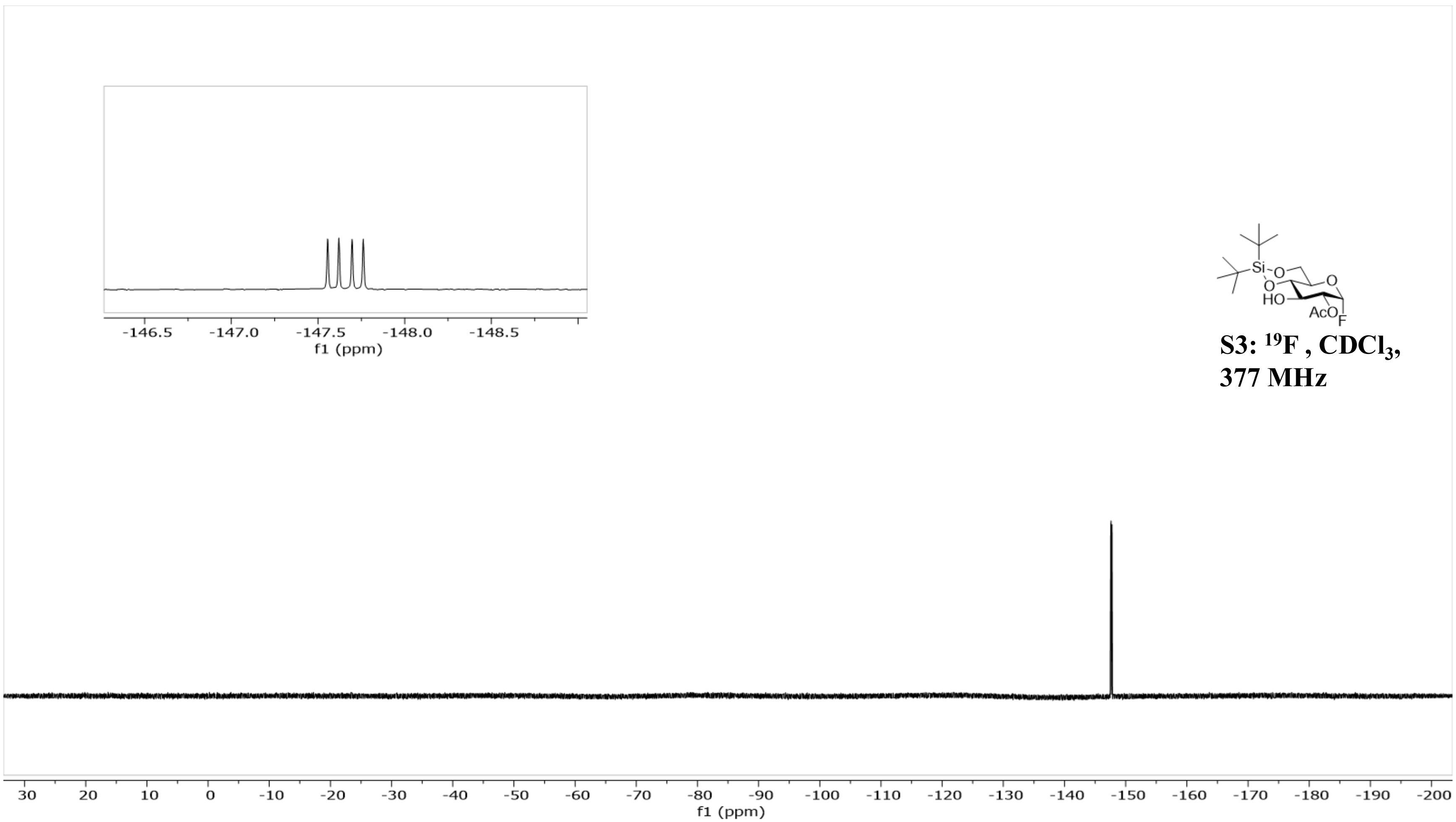

S3: ^{19}F , $CDCl_3$, 377 MHz

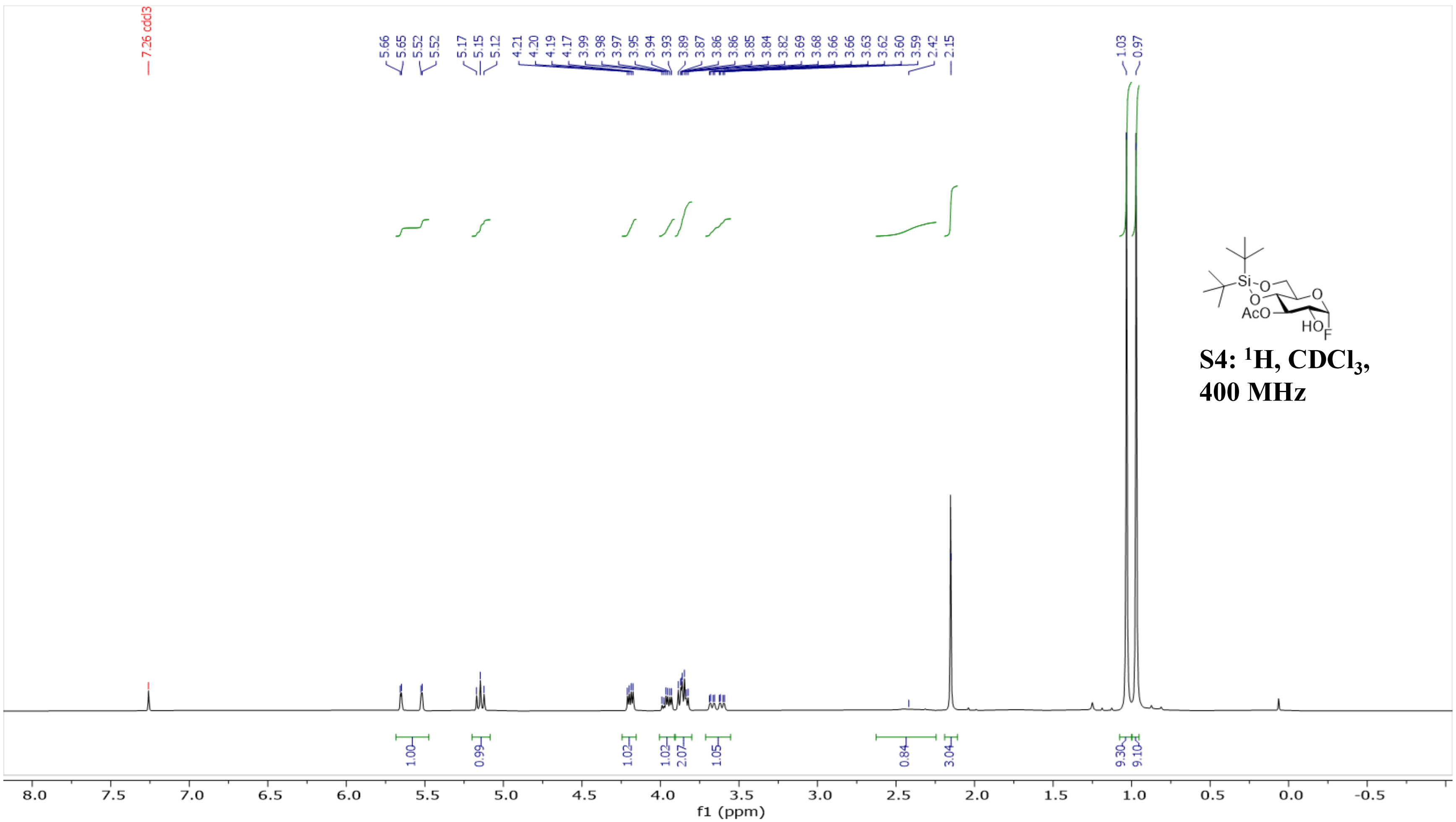

S4: ^{1}H, $CDCl_3$, 400 MHz

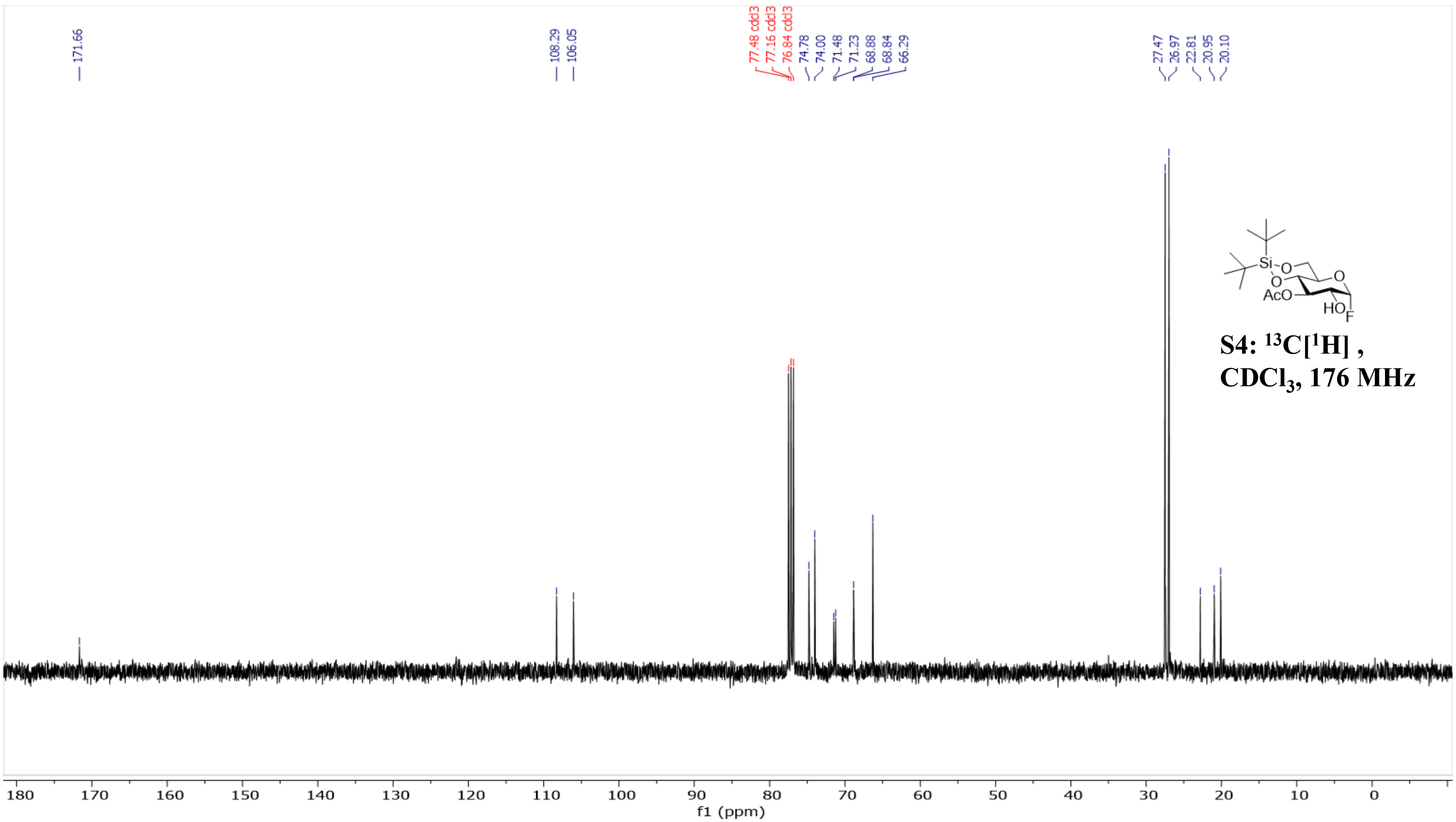
171.66
108.29
106.05
77.48 cdcl3
77.16 cdcl3
76.84 cdcl3
74.78
74.00
71.48
71.23
68.88
68.84
66.29
27.47
26.97
22.81
20.95
20.10
Si
O
O
O
AcO
HO
F
S4: 13C[1H] ,
CDCl3, 176 MHz
180 170 160 150 140 130 120 110 100 90 80 70 60 50 40 30 20 10 0
f1 (ppm)

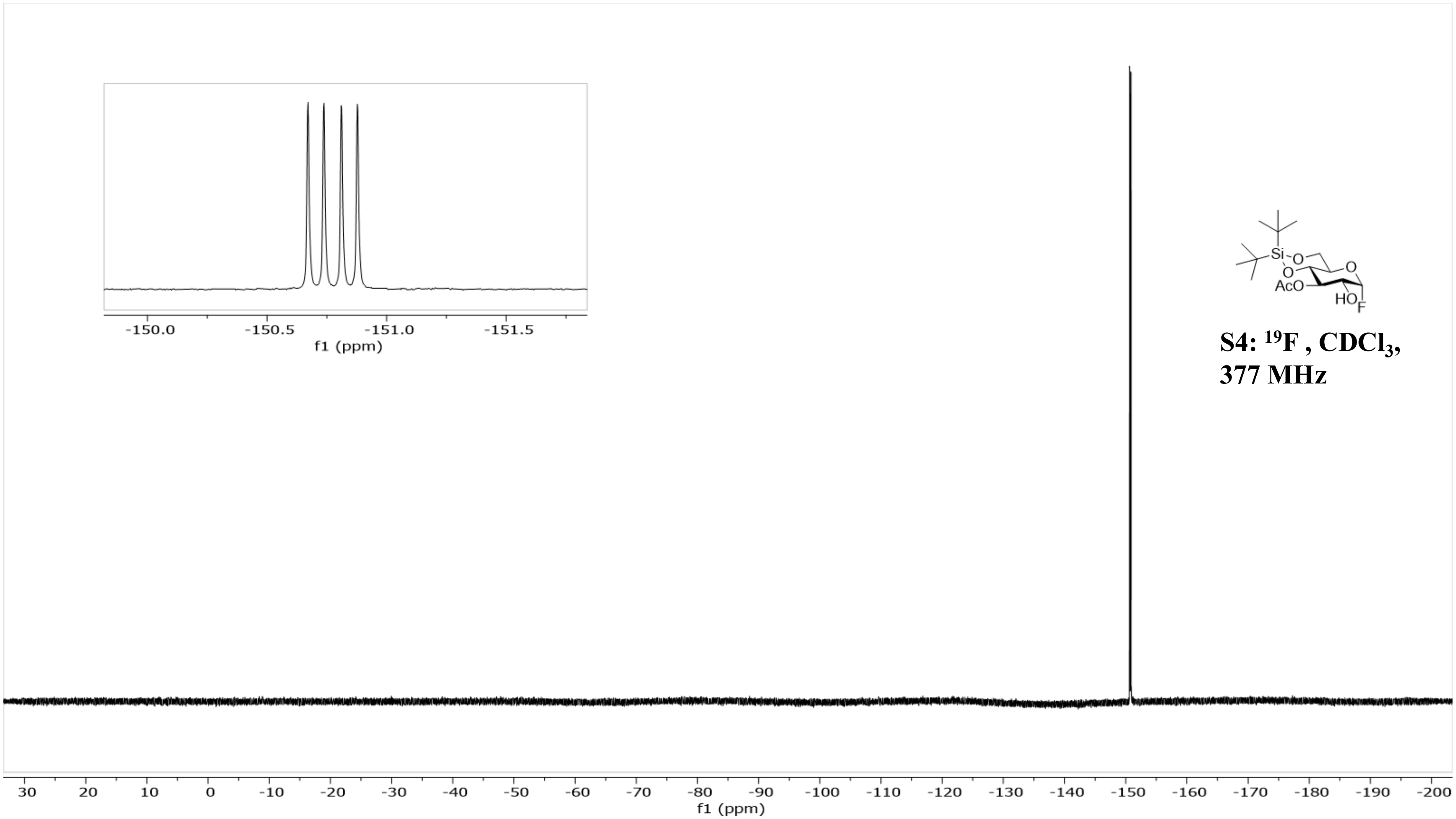
-150.0
-150.5
-151.0
-151.5
f1 (ppm)
Si
O
O
O
AcO
HO
F
S4: ^{19}F , $CDCl_3$,
377 MHz
30
20
10
0
-10
-20
-30
-40
-50
-60
-70
-80
-90
-100
-110
-120
-130
-140
-150
-160
-170
-180
-190
-200
f1 (ppm)

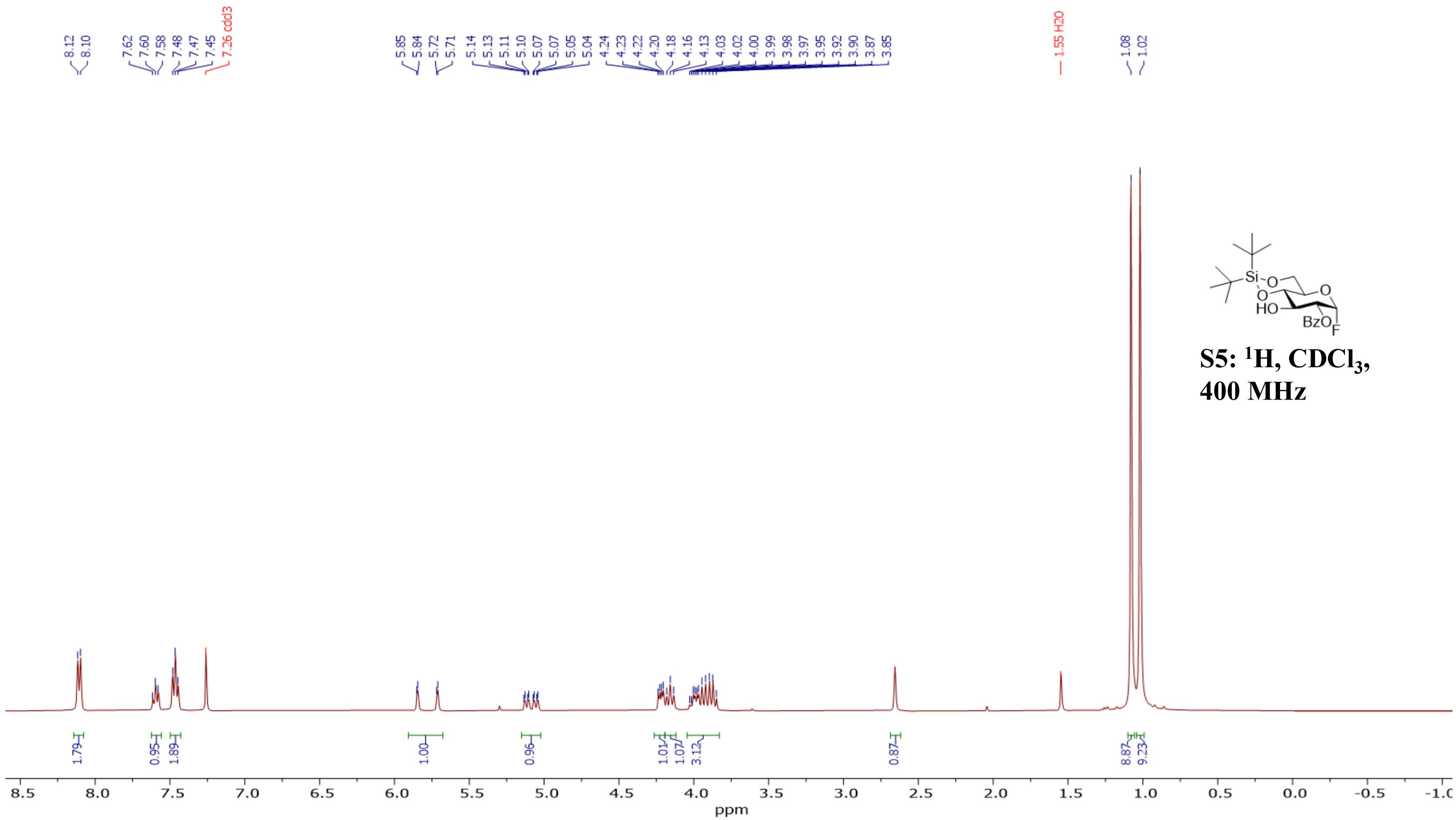
S5: ^{1}H, $CDCl_3$,
400 MHz
ppm
HO
BzO
F
Si
O

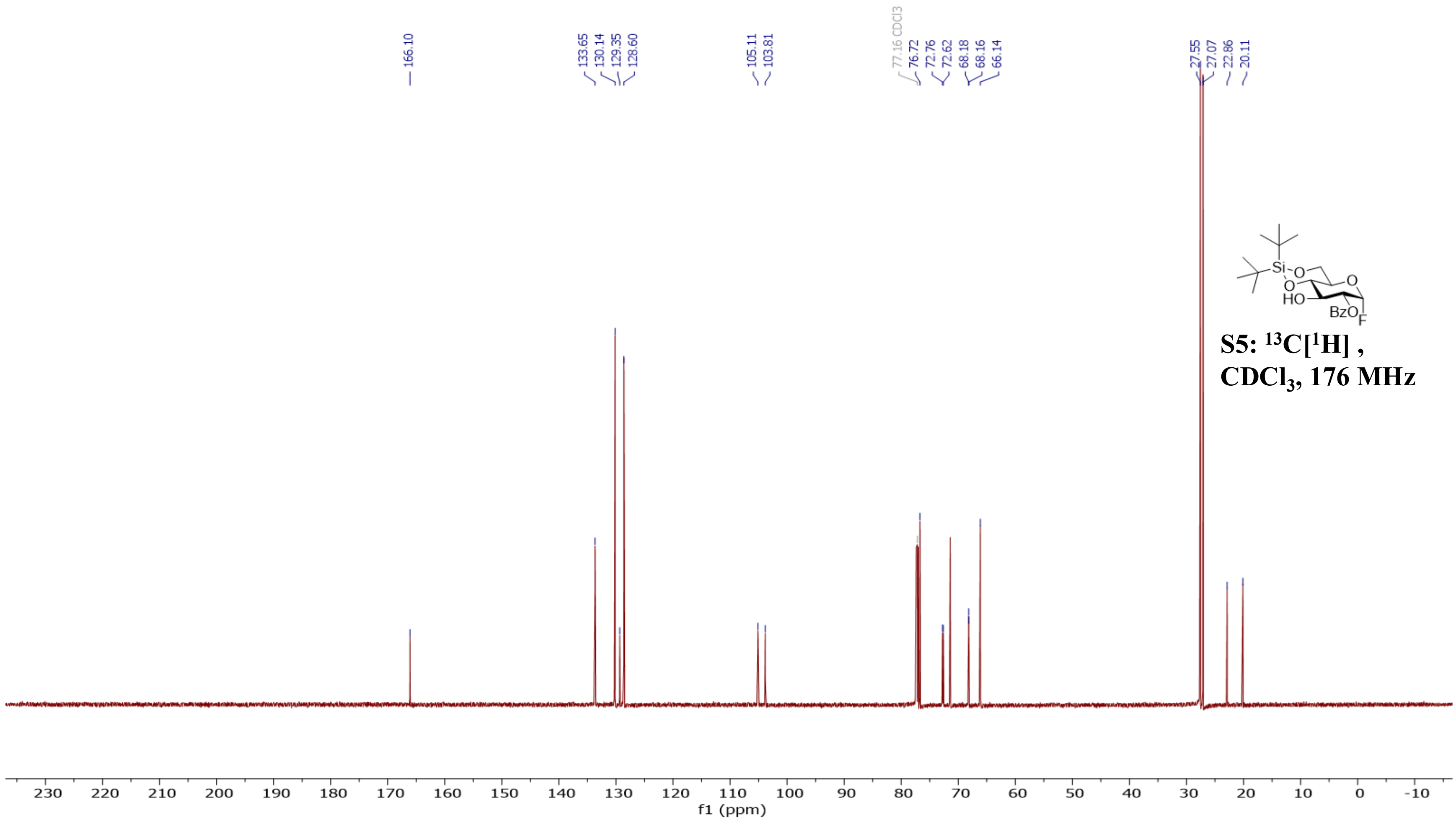

S5: $^{13}C[^{1}H]$, $CDCl_3$, 176 MHz

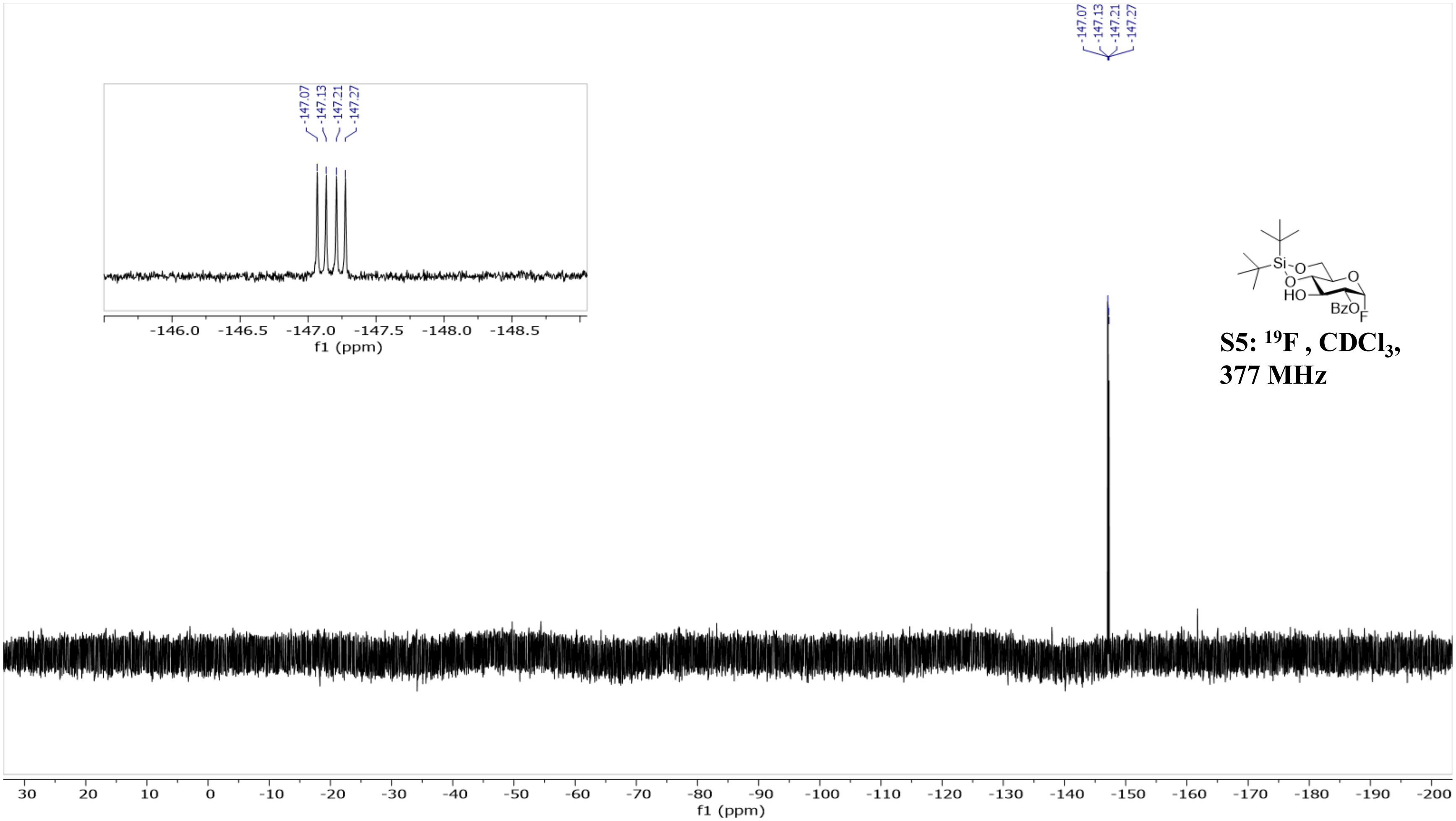

S5: ^{19}F , $CDCl_3$, 377 MHz

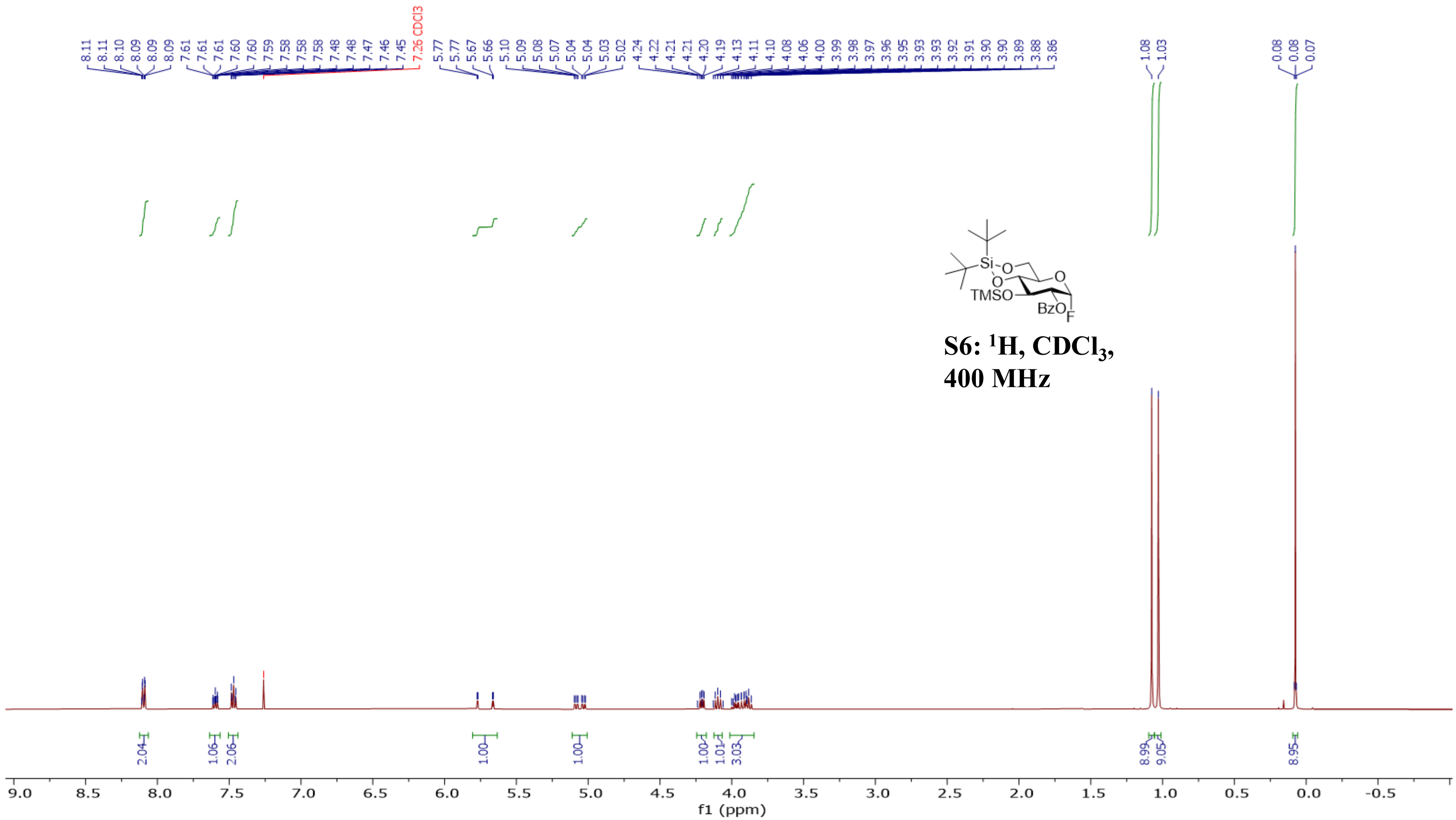

S6: ¹H, CDCl3, 400 MHz
TMSO
BzO
F
Si
O
7.26 CDCl3
f1 (ppm)

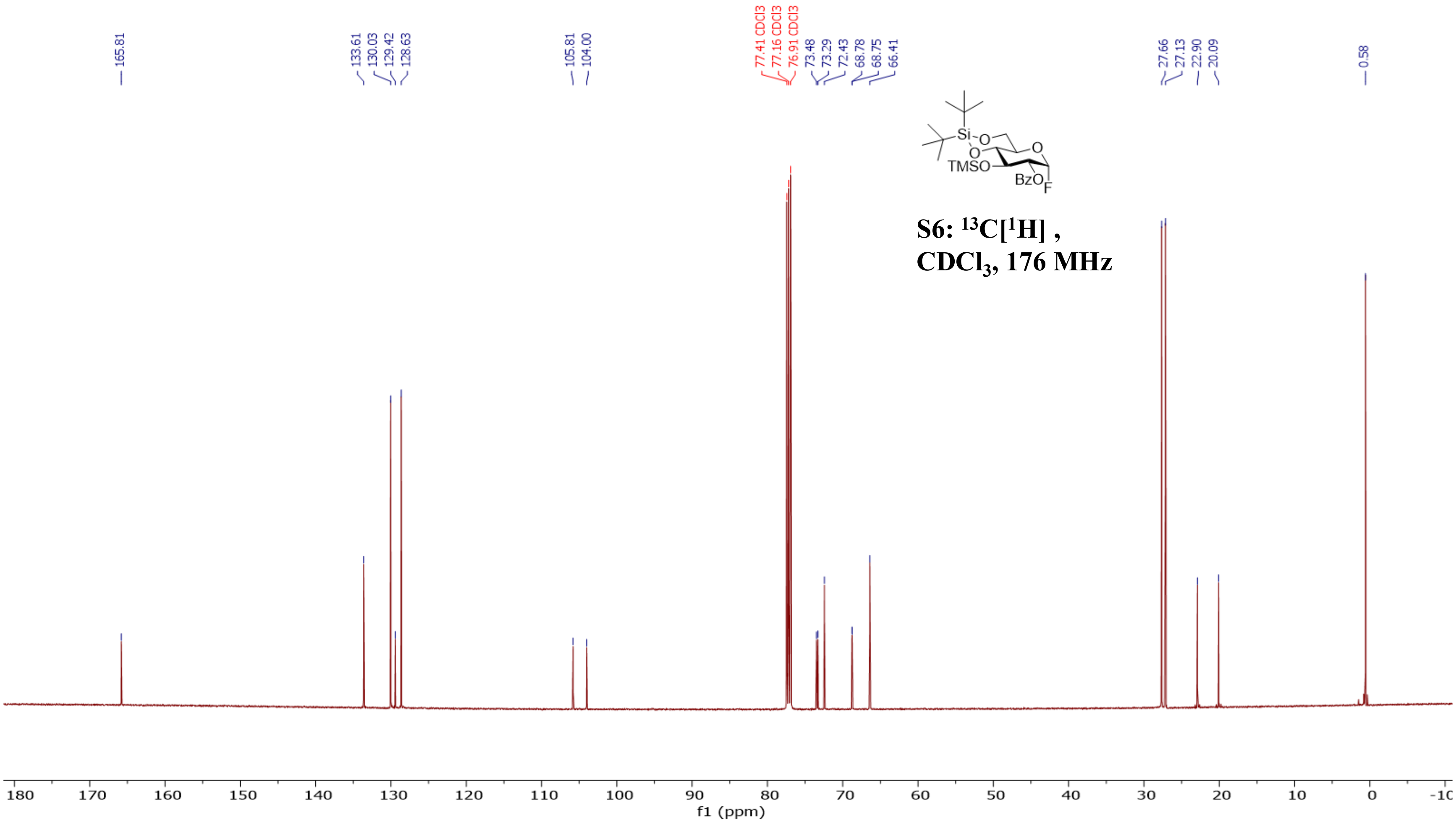

S6: $^{13}C[^{1}H]$, $CDCl_3$, 176 MHz

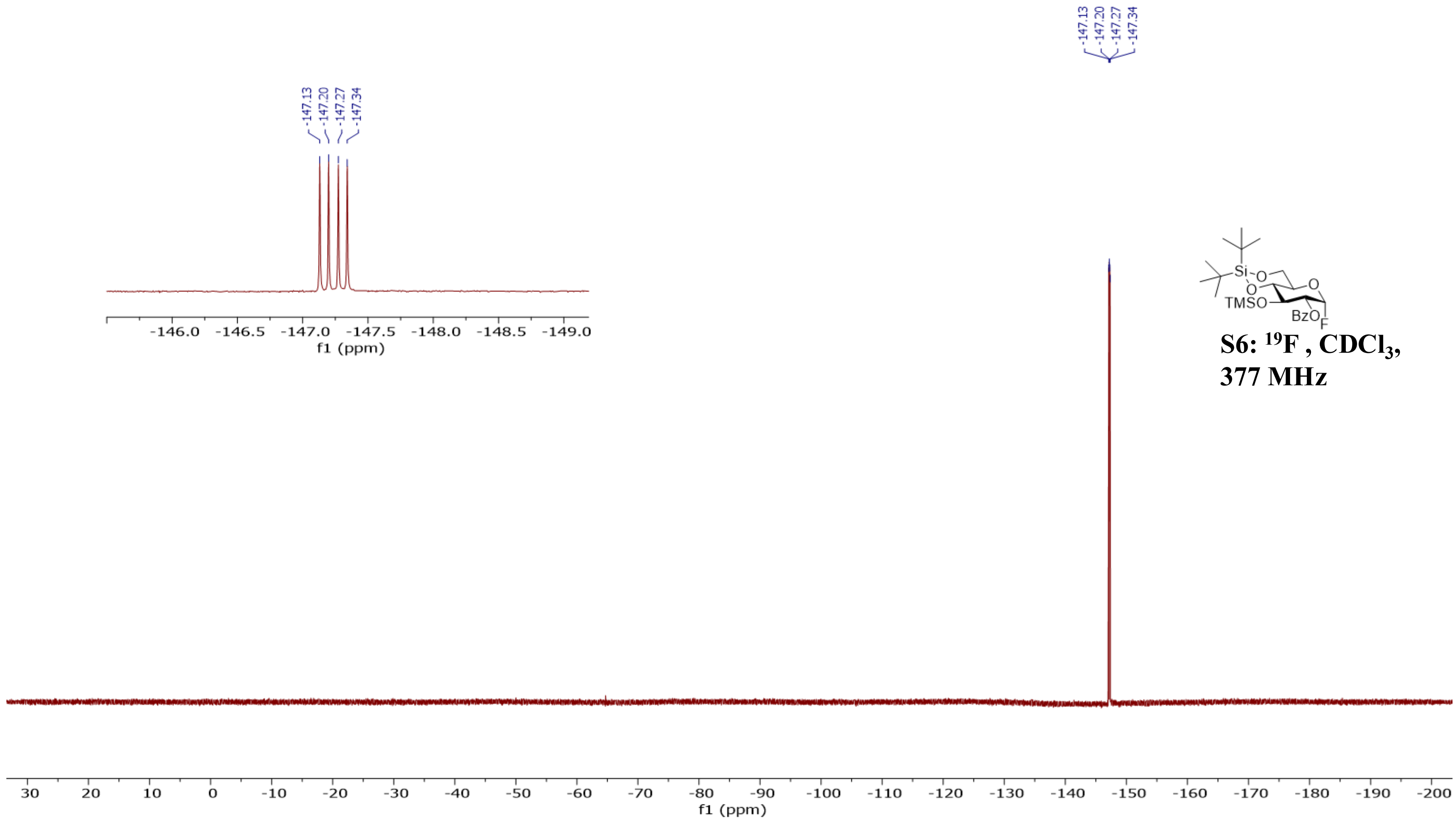

S6: ^{19}F , CDCl$_3$, 377 MHz

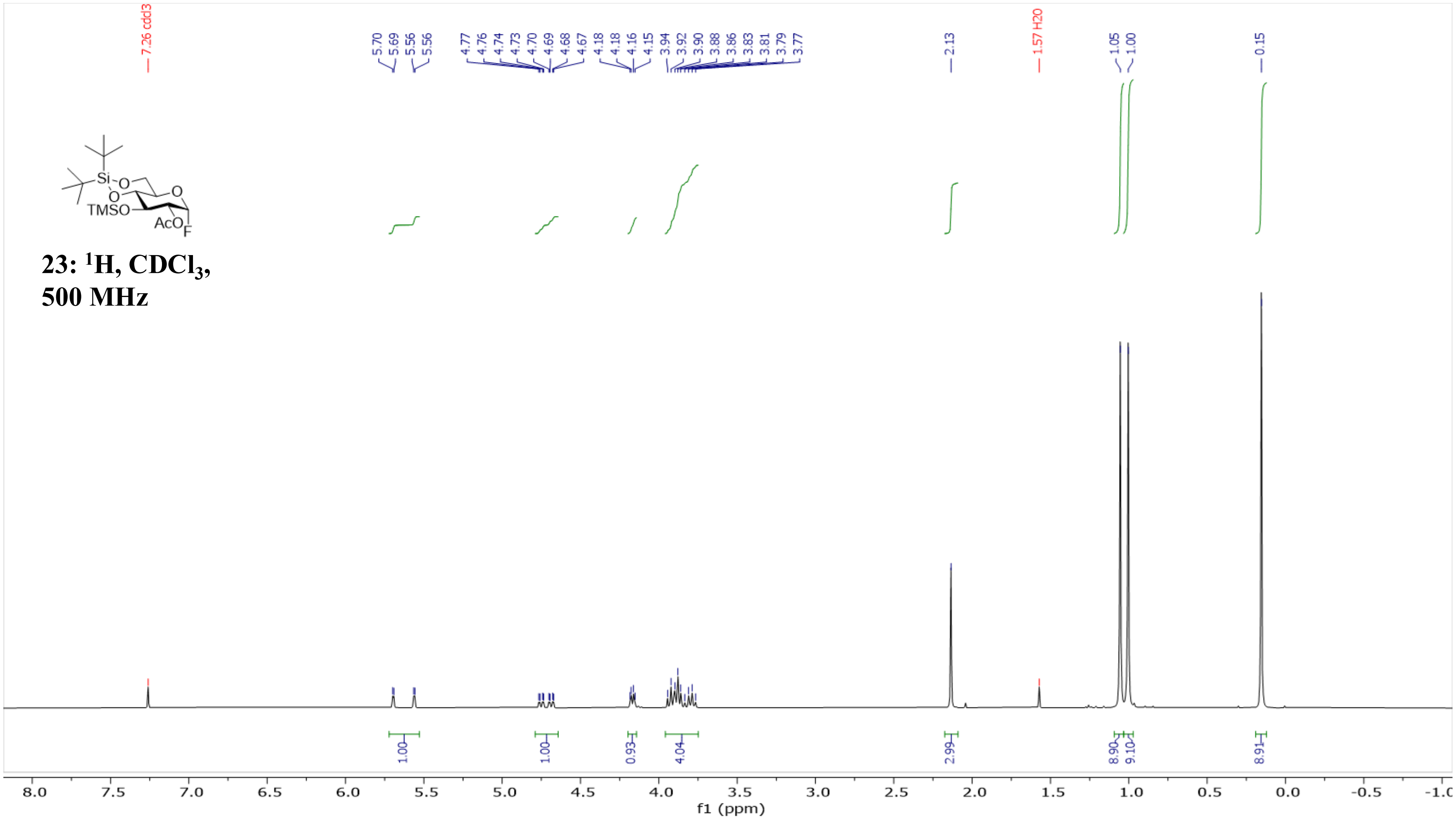

23: ^{1}H, $CDCl_3$, 500 MHz

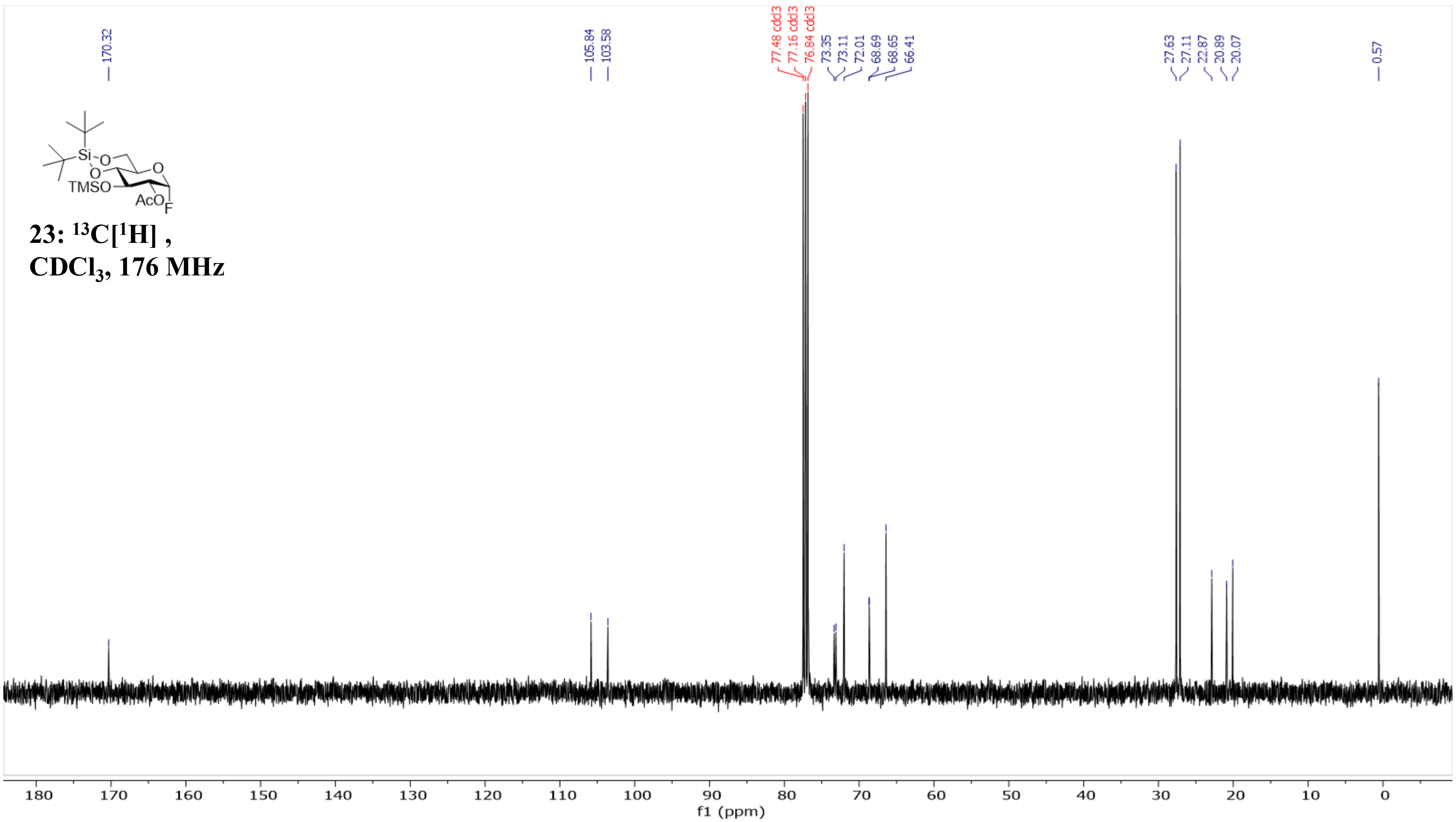

23: ^{13}C[^{1}H] , CDCl$_3$, 176 MHz

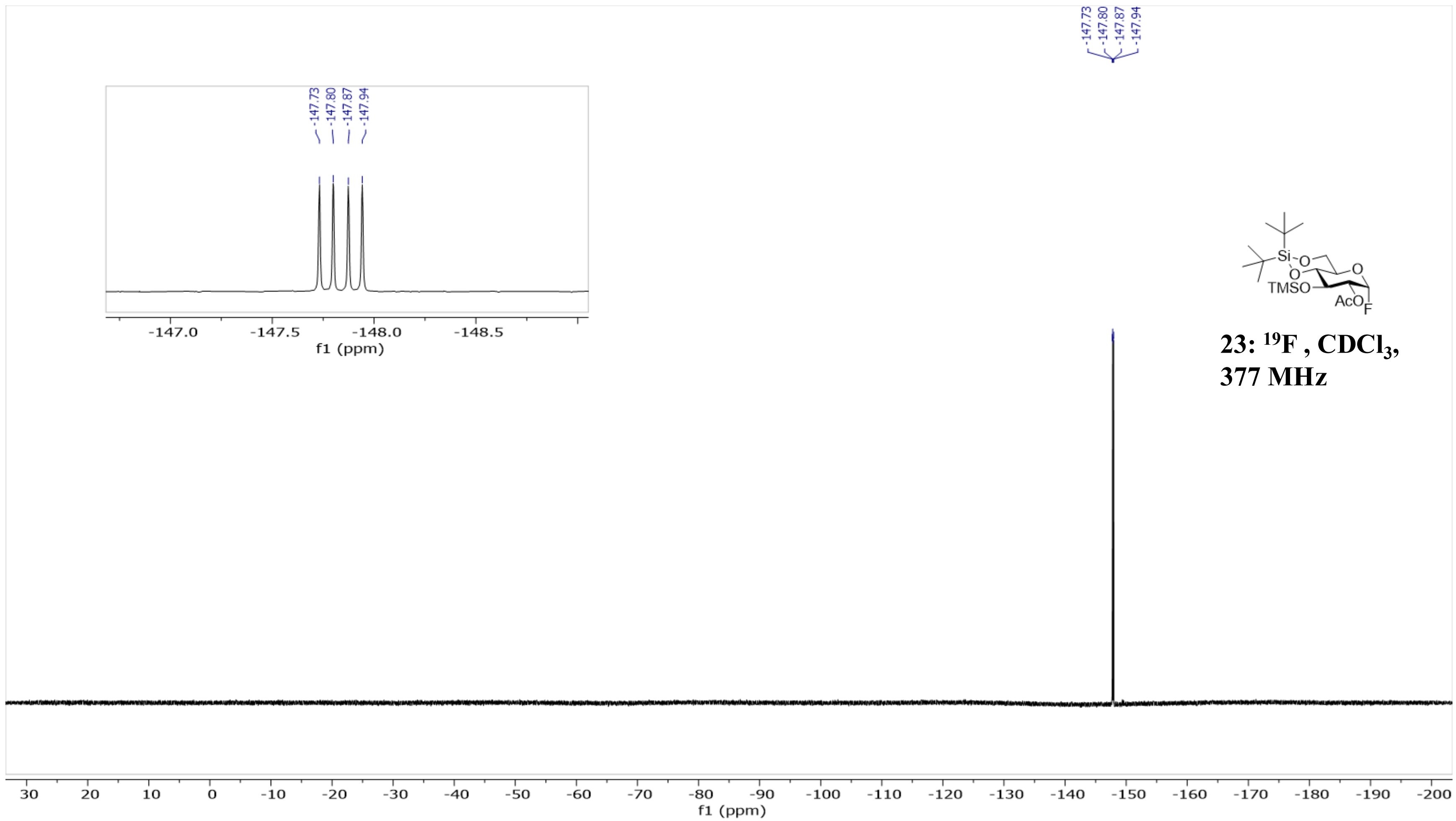

23: ^{19}F , $CDCl_3$, 377 MHz

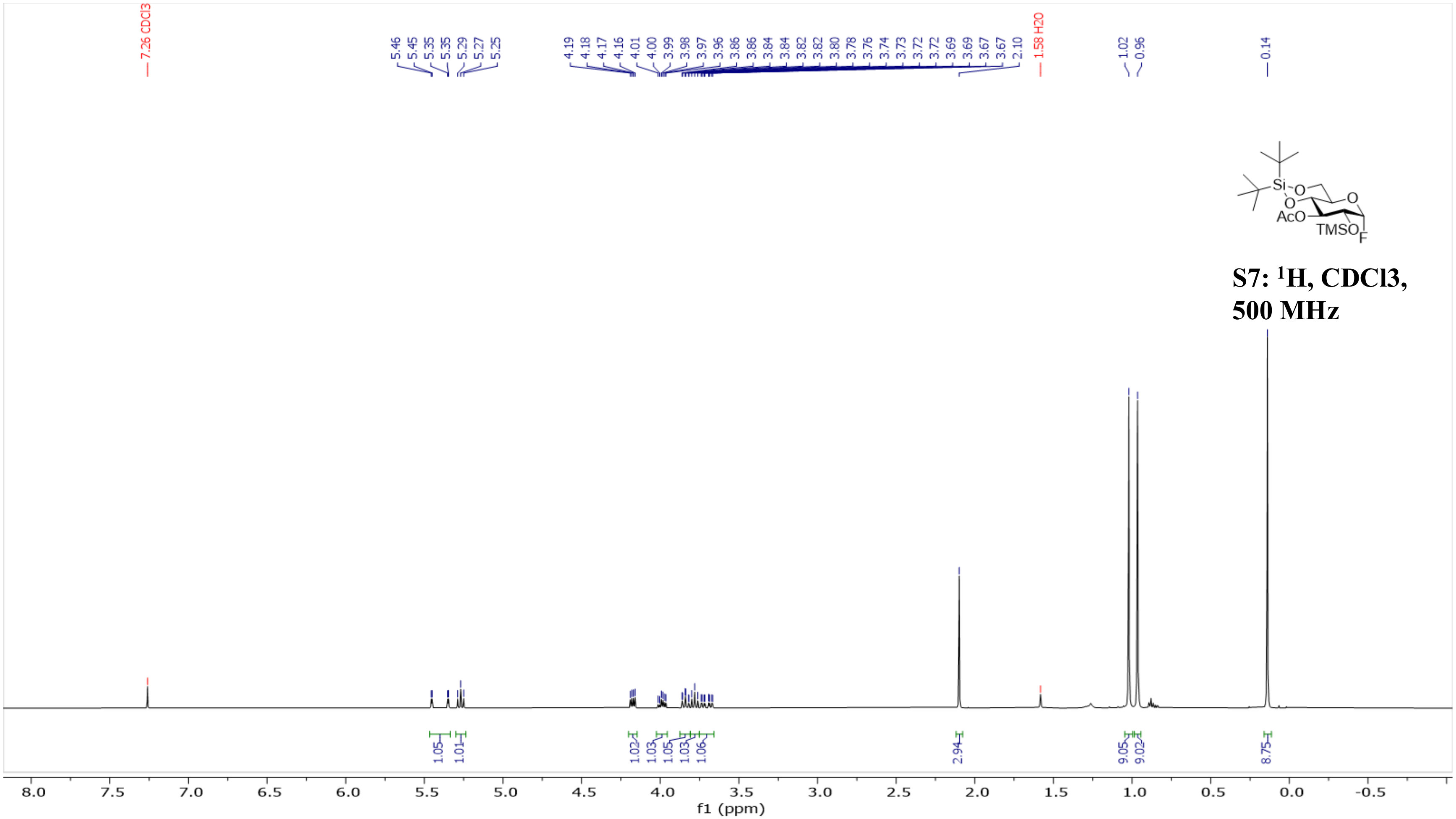

S7: ^{1}H, CDCl3, 500 MHz

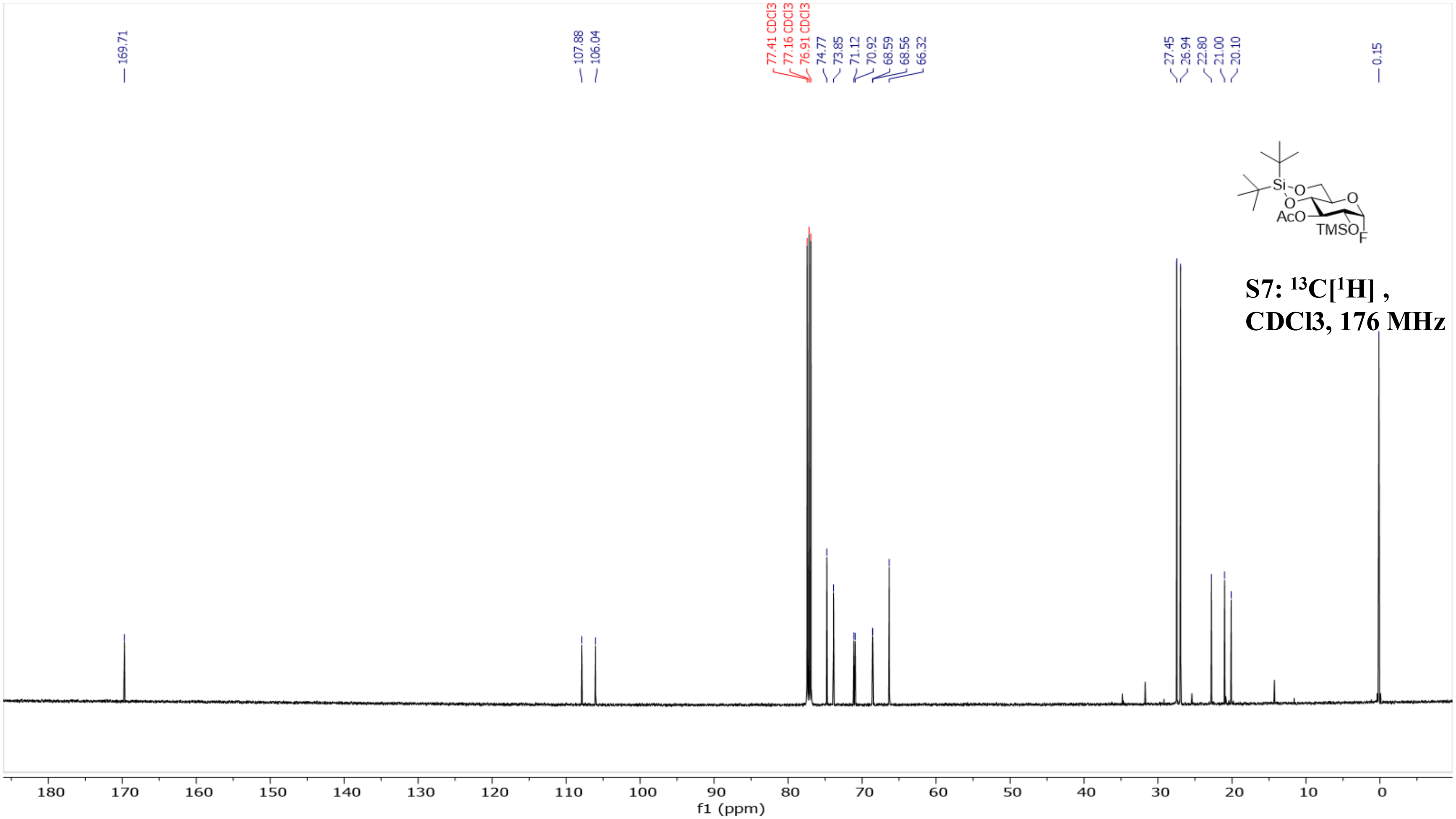
169.71
107.88
106.04
77.41 CDCl3
77.16 CDCl3
76.91 CDCl3
74.77
73.85
71.12
70.92
68.59
68.56
66.32
27.45
26.94
22.80
21.00
20.10
0.15
Si
O
O
O
AcO
TMSO
F
S7: 13C[1H] ,
CDCl3, 176 MHz
f1 (ppm)

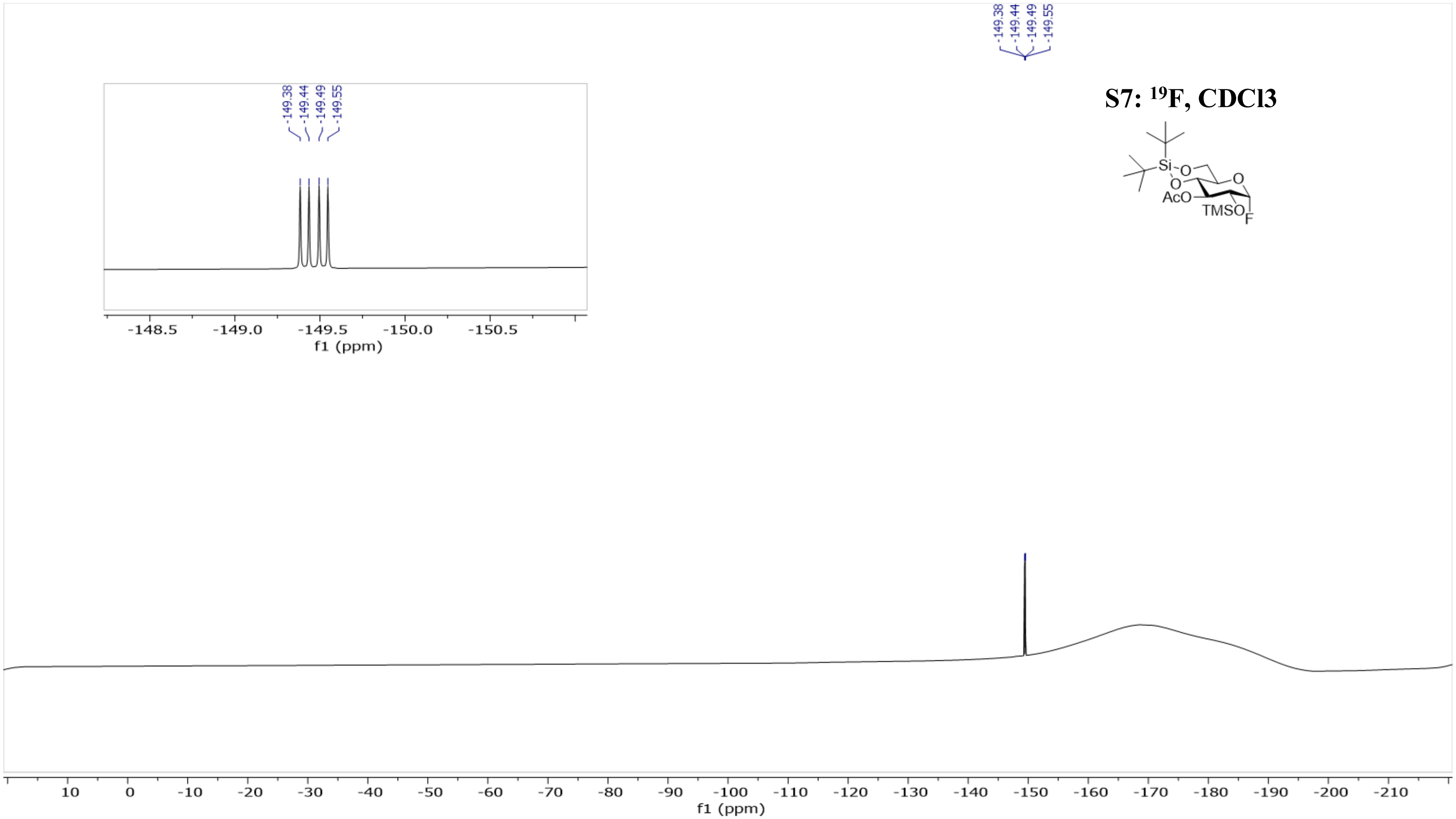

-149.38
-149.44
-149.49
-149.55
S7: 19F, CDCl3
Si
O
O
O
AcO
TMSO
F
-148.5
-149.0
-149.5
-150.0
-150.5
f1 (ppm)
10
0
-10
-20
-30
-40
-50
-60
-70
-80
-90
-100
-110
-120
-130
-140
-150
-160
-170
-180
-190
-200
-210
f1 (ppm)

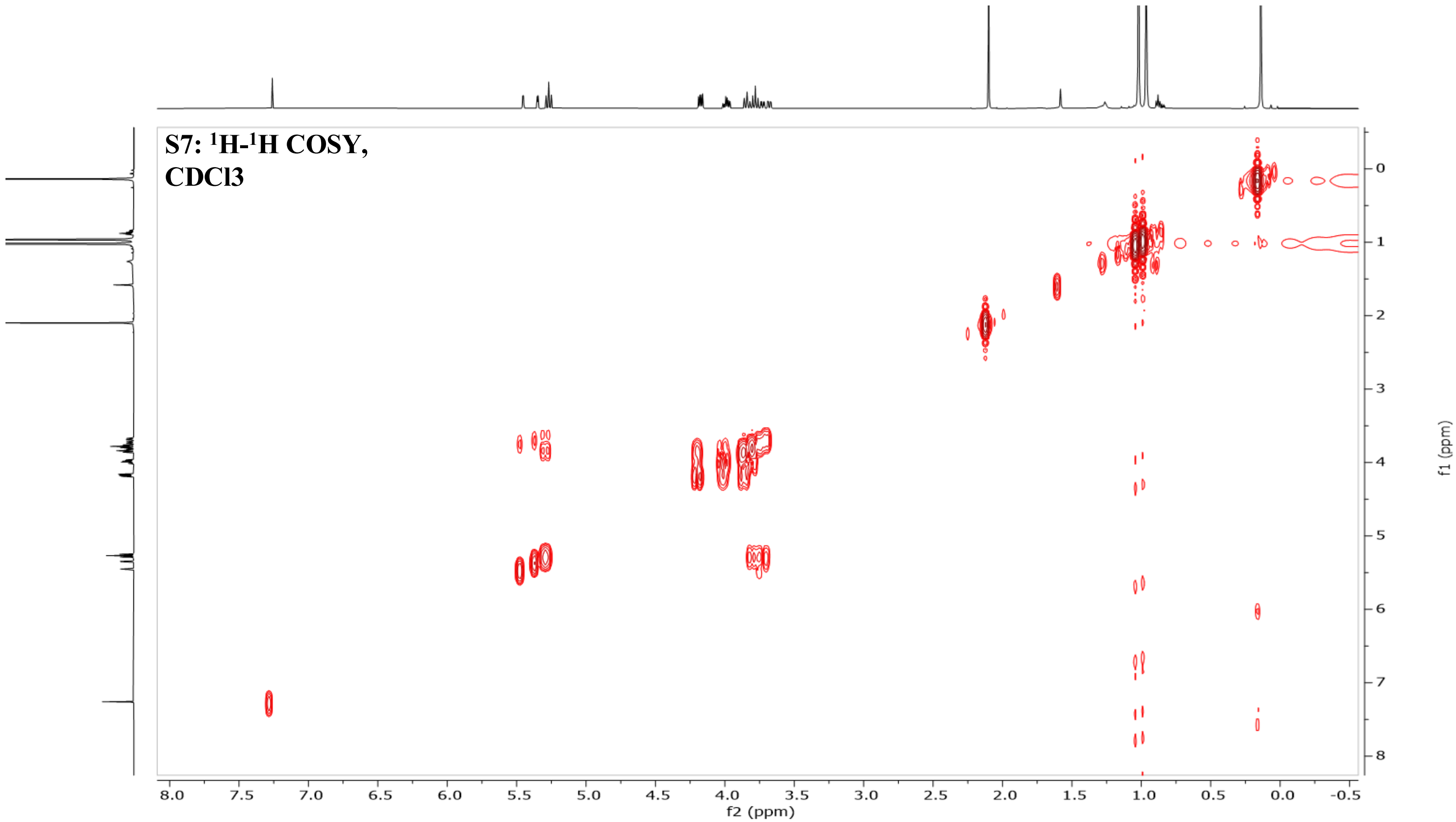

S7: 1H-1H COSY,
CDCl3
8.0 7.5 7.0 6.5 6.0 5.5 5.0 4.5 4.0 3.5 3.0 2.5 2.0 1.5 1.0 0.5 0.0 -0.5
f2 (ppm)
0 1 2 3 4 5 6 7 8
f1 (ppm)

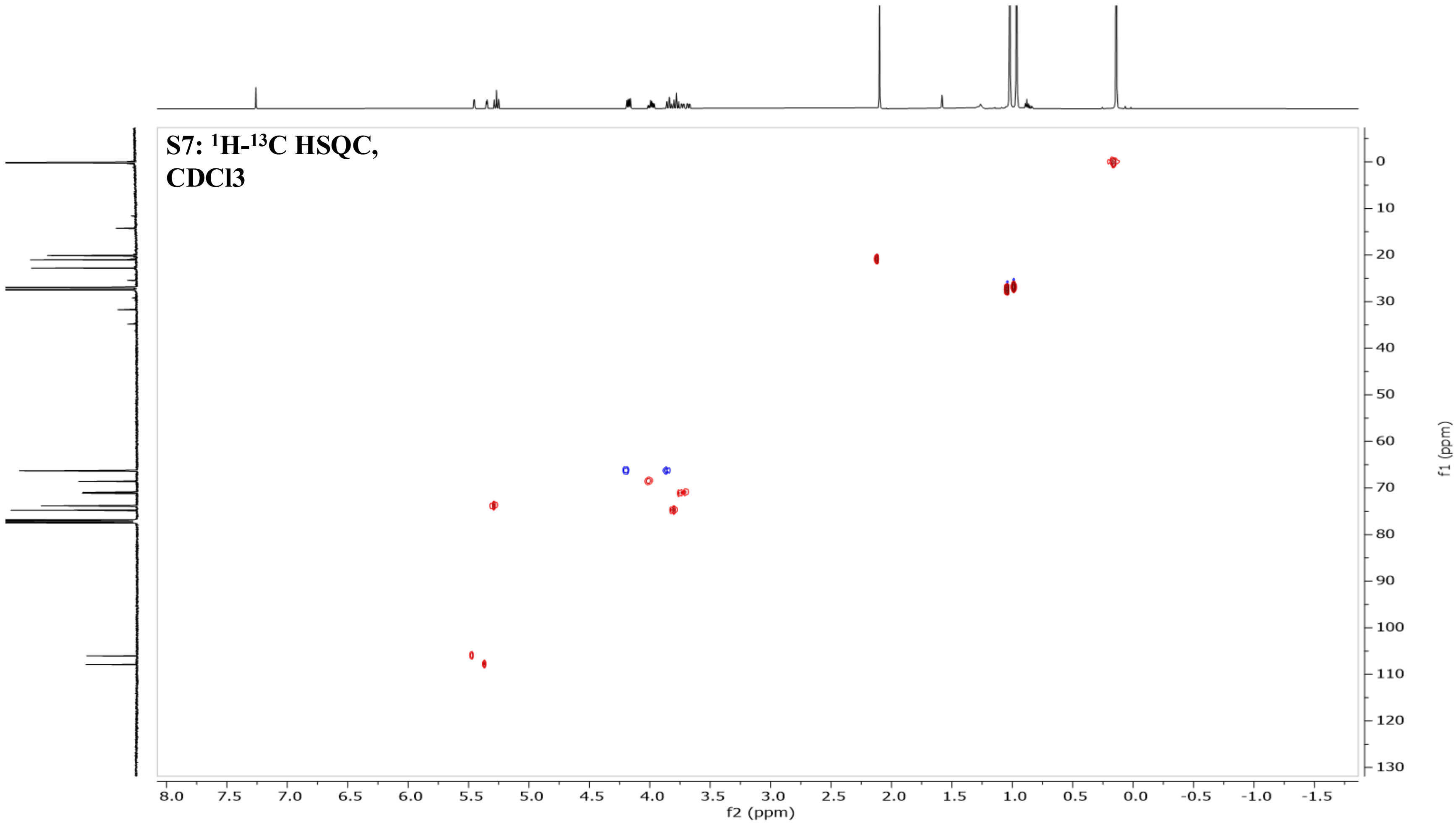
S7: 1H-13C HSQC,
CDCl3
f2 (ppm)
f1 (ppm)
8.0 7.5 7.0 6.5 6.0 5.5 5.0 4.5 4.0 3.5 3.0 2.5 2.0 1.5 1.0 0.5 0.0 -0.5 -1.0 -1.5
0 10 20 30 40 50 60 70 80 90 100 110 120 130

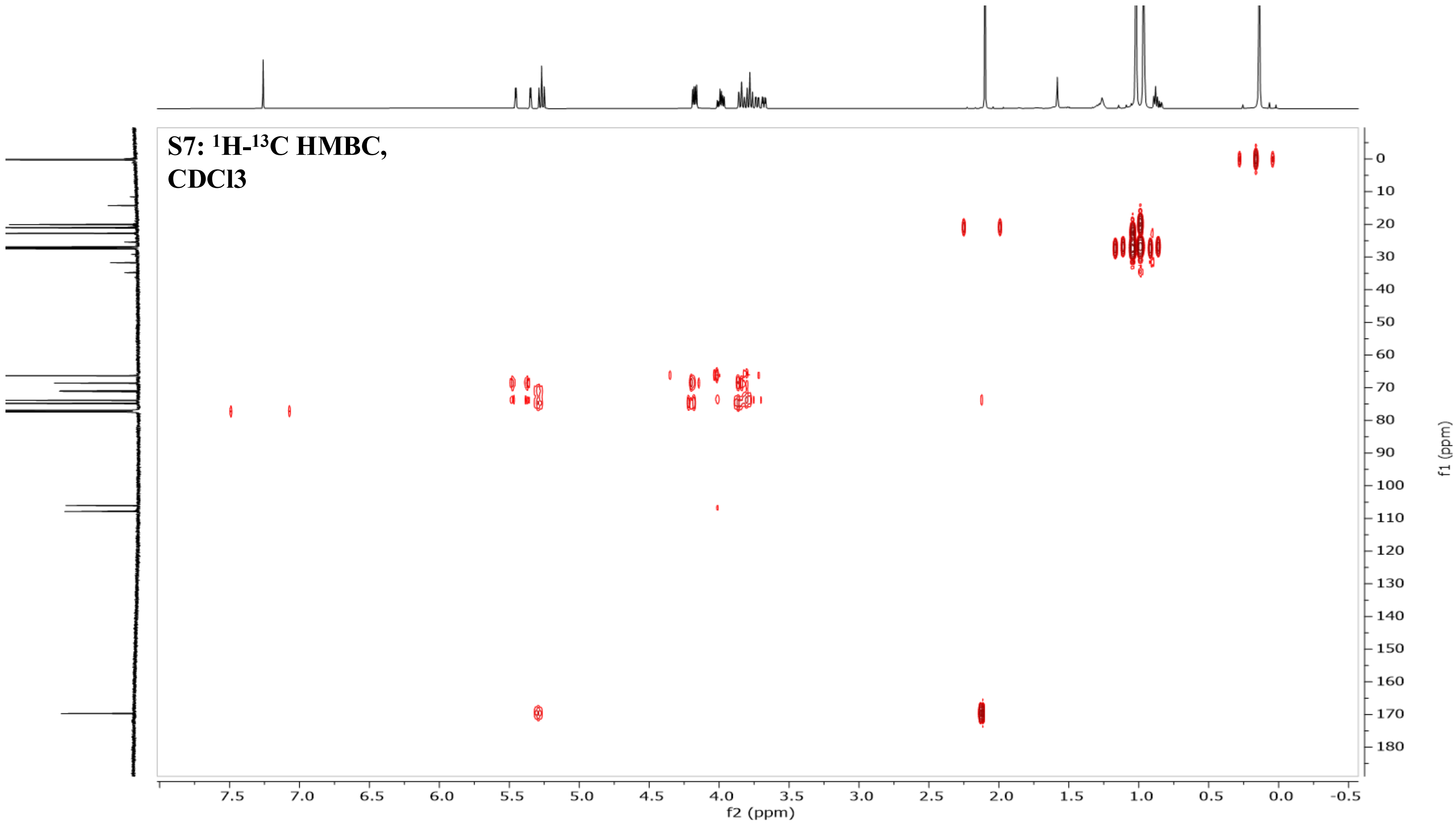
S7: ^{1}H-^{13}C HMBC,
CDCl3
f2 (ppm)
f1 (ppm)

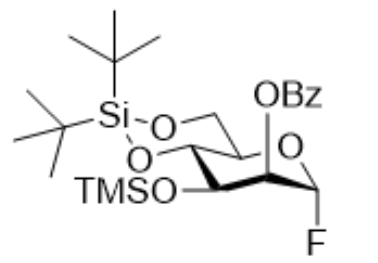

S9: ^{1}H, $CDCl_3$, 400 MHz

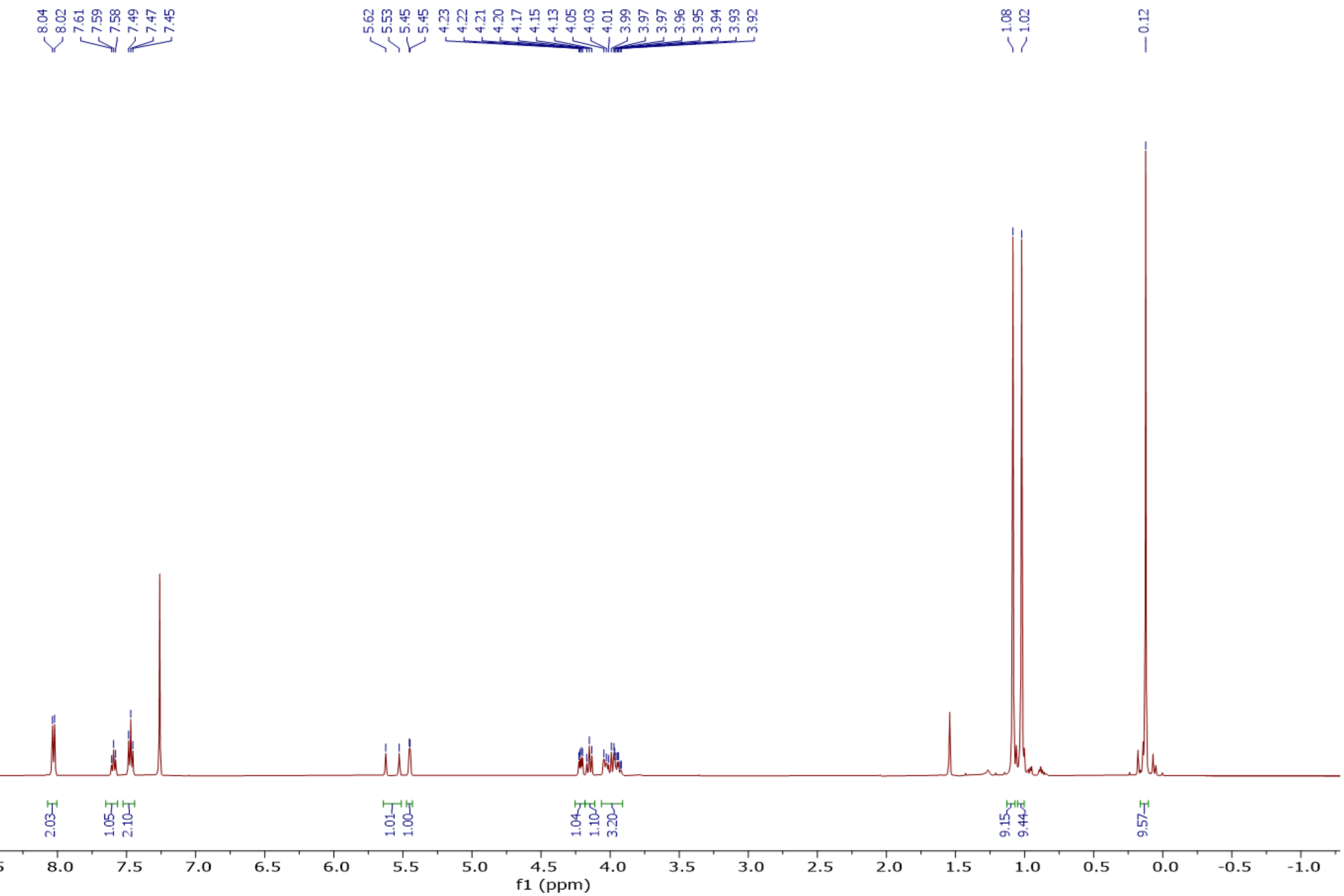

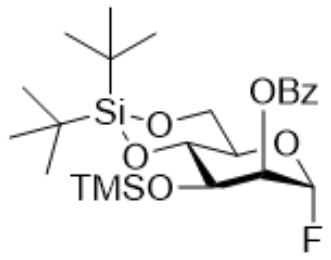

S9: $^{13}C[^{1}H]$,
$CDCl_3$, 176 MHz

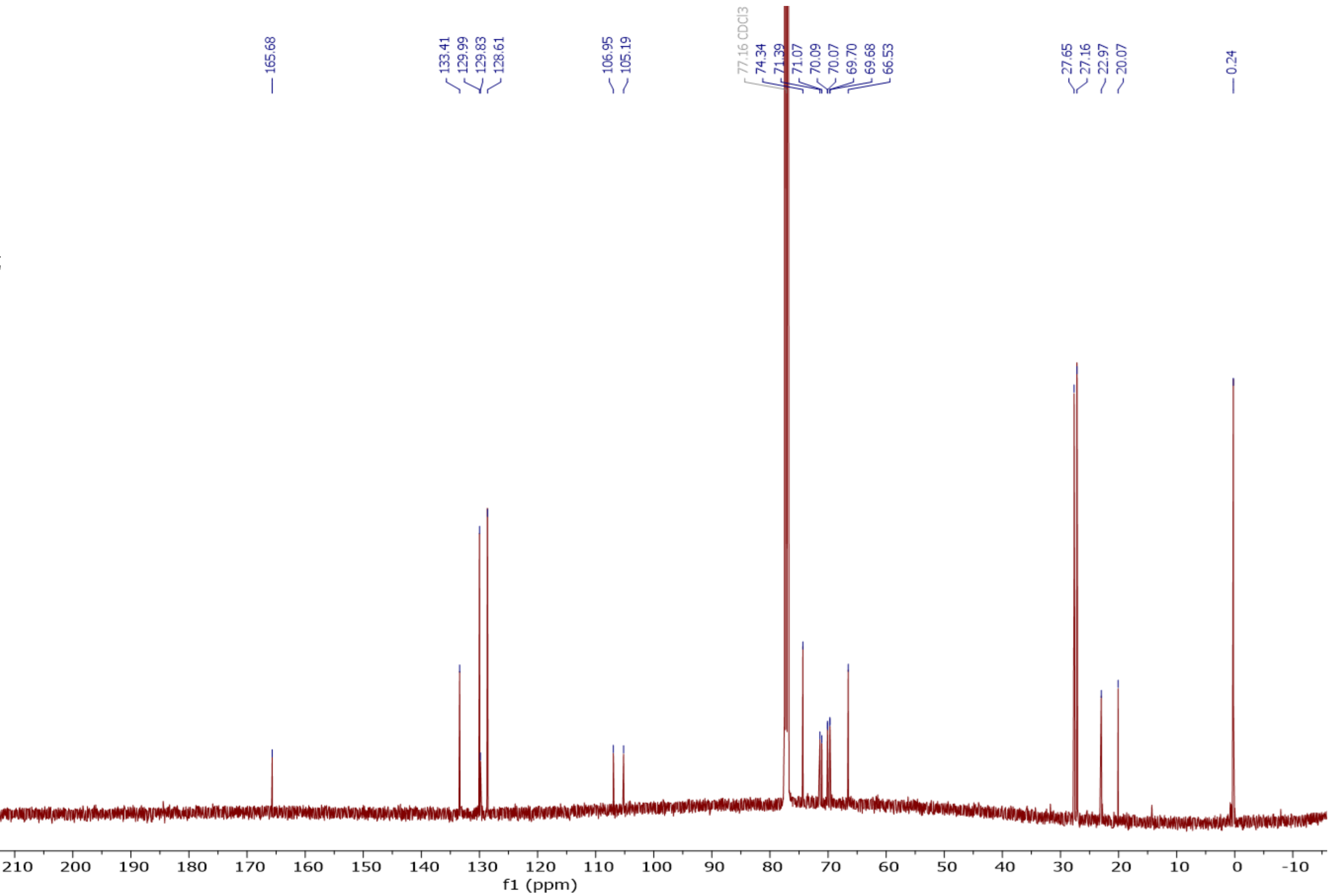

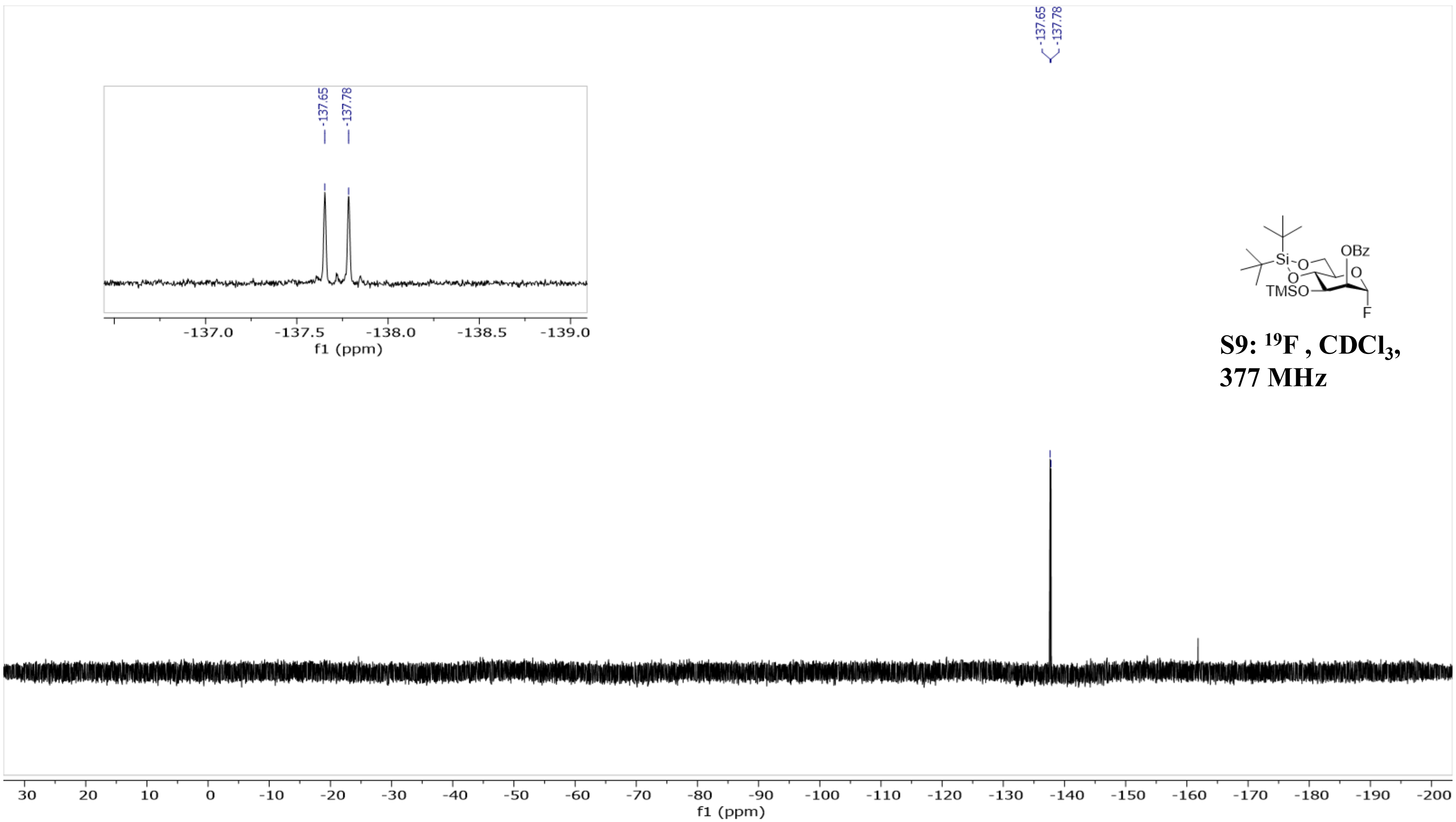

S9: ^{19}F , CDCl$_3$, 377 MHz

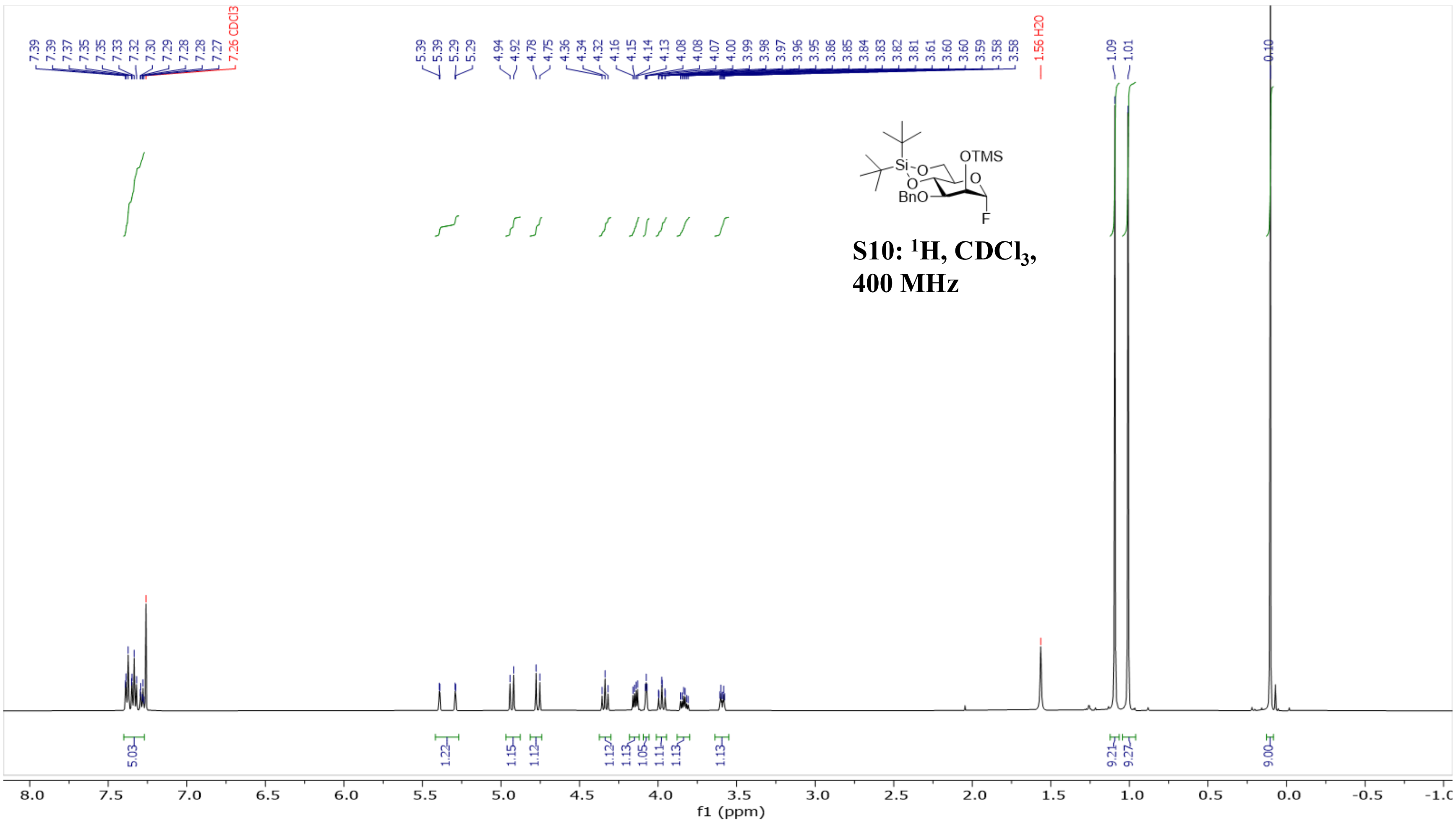

S10: ^{1}H, $CDCl_3$, 400 MHz

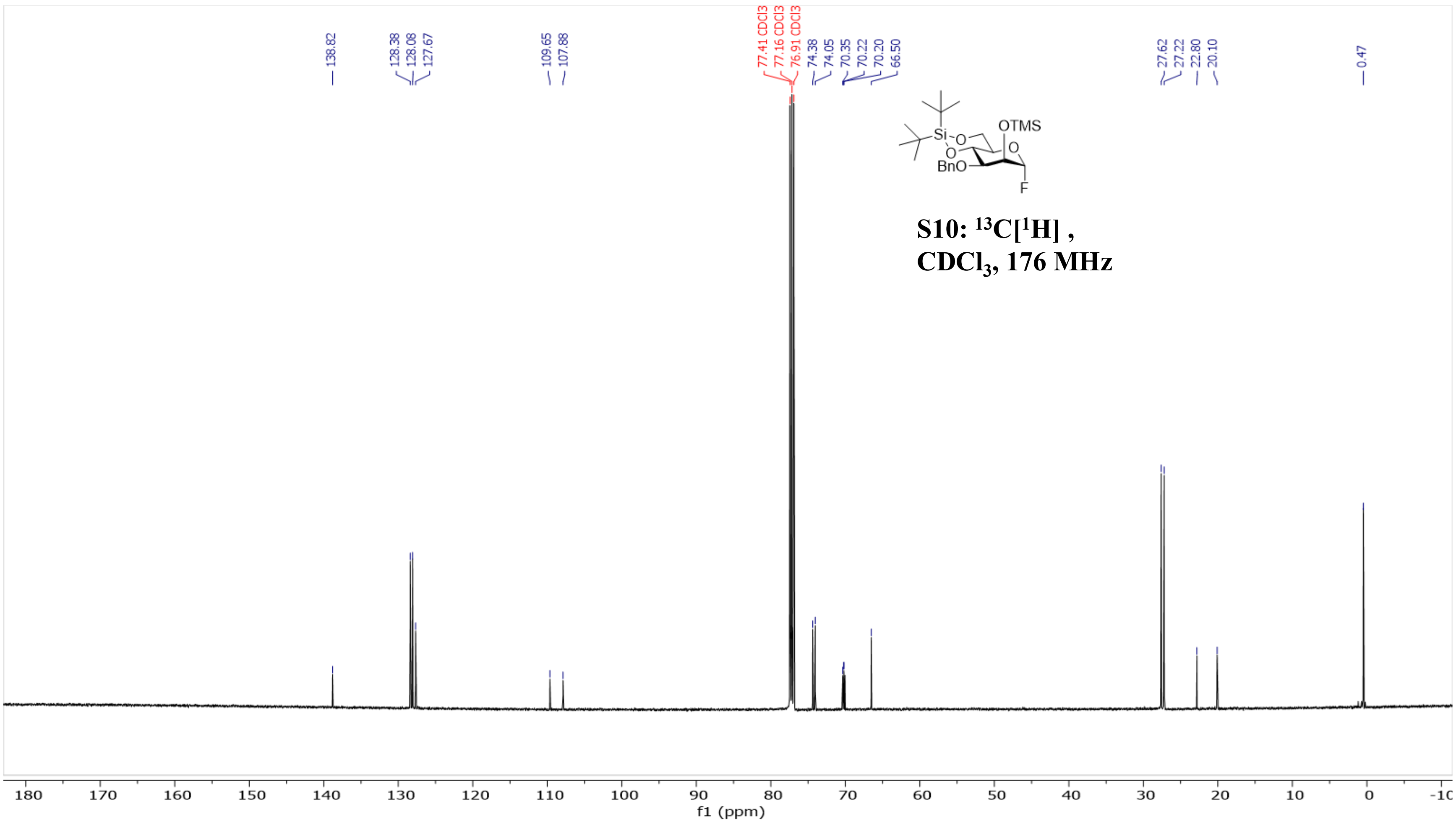
138.82
128.38
128.08
127.67
109.65
107.88
77.41 CDCl3
77.16 CDCl3
76.91 CDCl3
74.38
74.05
70.35
70.22
70.20
66.50
27.62
27.22
22.80
20.10
0.47
OTMS
Si
O
O
O
BnO
F
S10: $^{13}C[^{1}H]$,
$CDCl_3$, 176 MHz
180
170
160
150
140
130
120
110
100
90
80
70
60
50
40
30
20
10
0
-10
f1 (ppm)

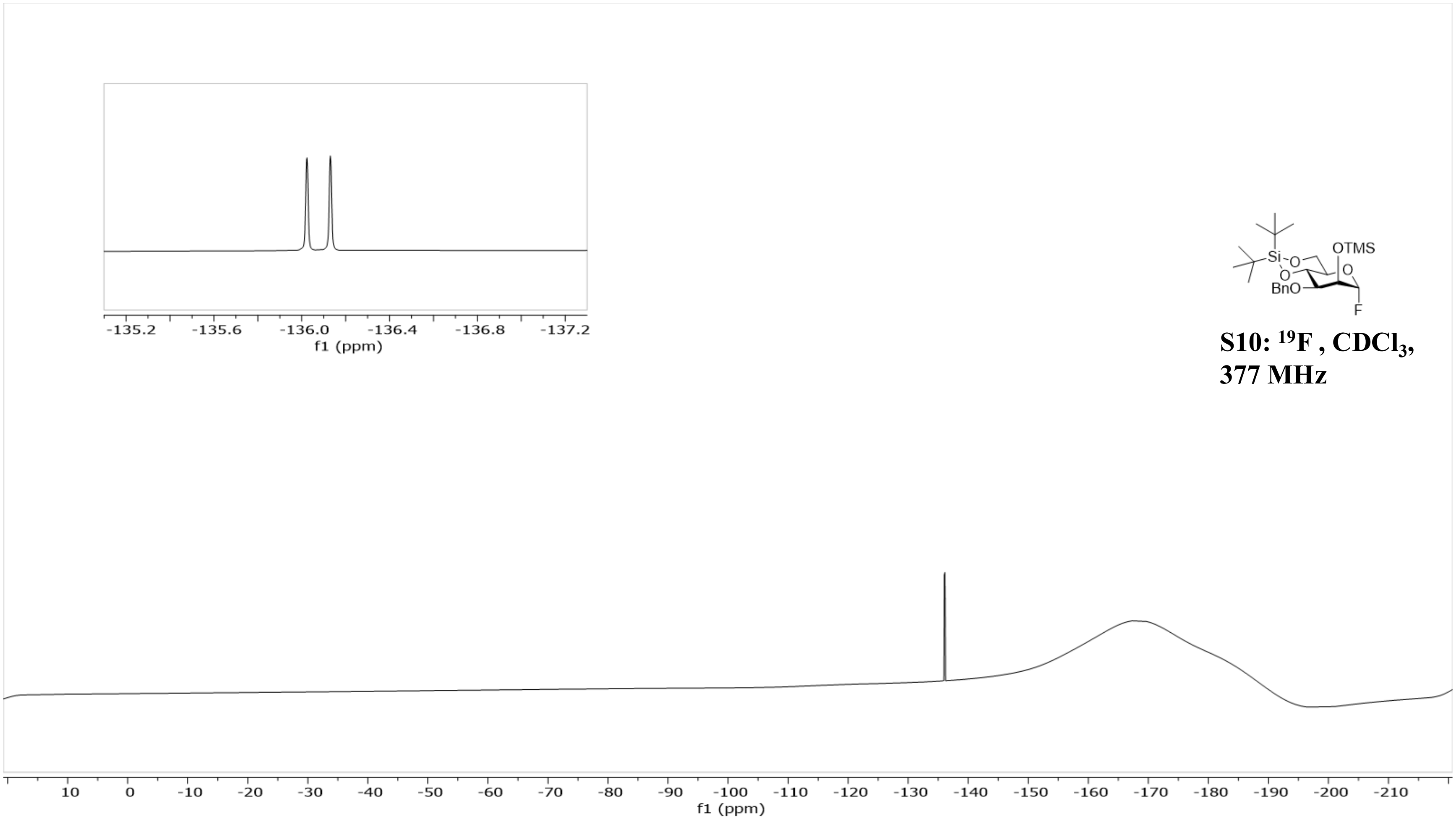

S10: ^{19}F , $CDCl_3$, 377 MHz

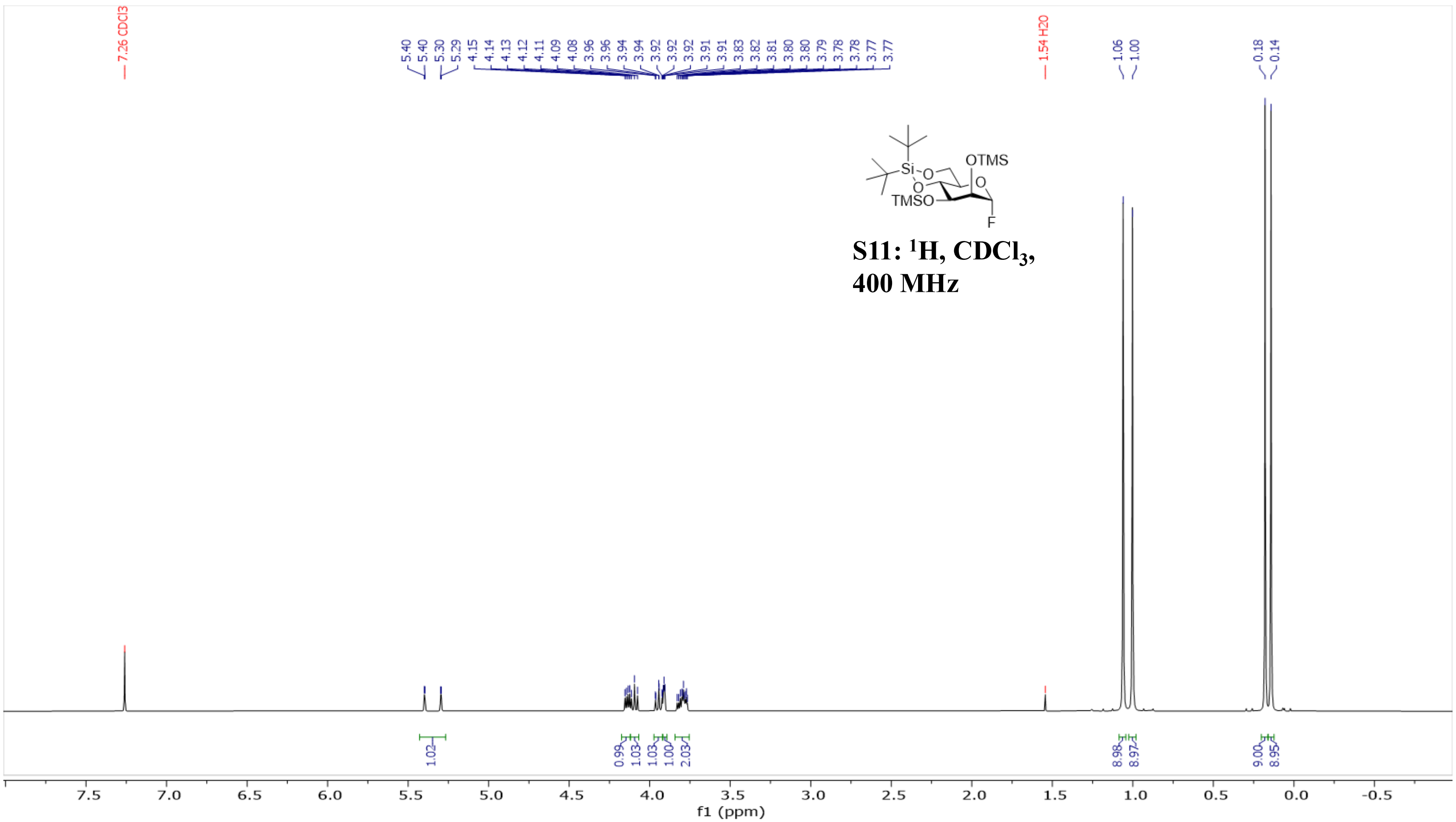
7.26 CDCl3
5.40
5.40
5.30
5.29
4.15
4.14
4.13
4.12
4.11
4.09
4.08
3.96
3.96
3.94
3.94
3.92
3.92
3.92
3.91
3.91
3.83
3.82
3.81
3.80
3.80
3.79
3.78
3.78
3.77
3.77
1.54 H2O
1.06
1.00
0.18
0.14
OTMS
Si
O
O
O
TMSO
F
S11: 1H, CDCl3, 400 MHz
1.02
0.99
1.03
1.03
1.00
2.03
8.98
8.97
9.00
8.95
7.5
7.0
6.5
6.0
5.5
5.0
4.5
4.0
3.5
3.0
2.5
2.0
1.5
1.0
0.5
0.0
-0.5
f1 (ppm)

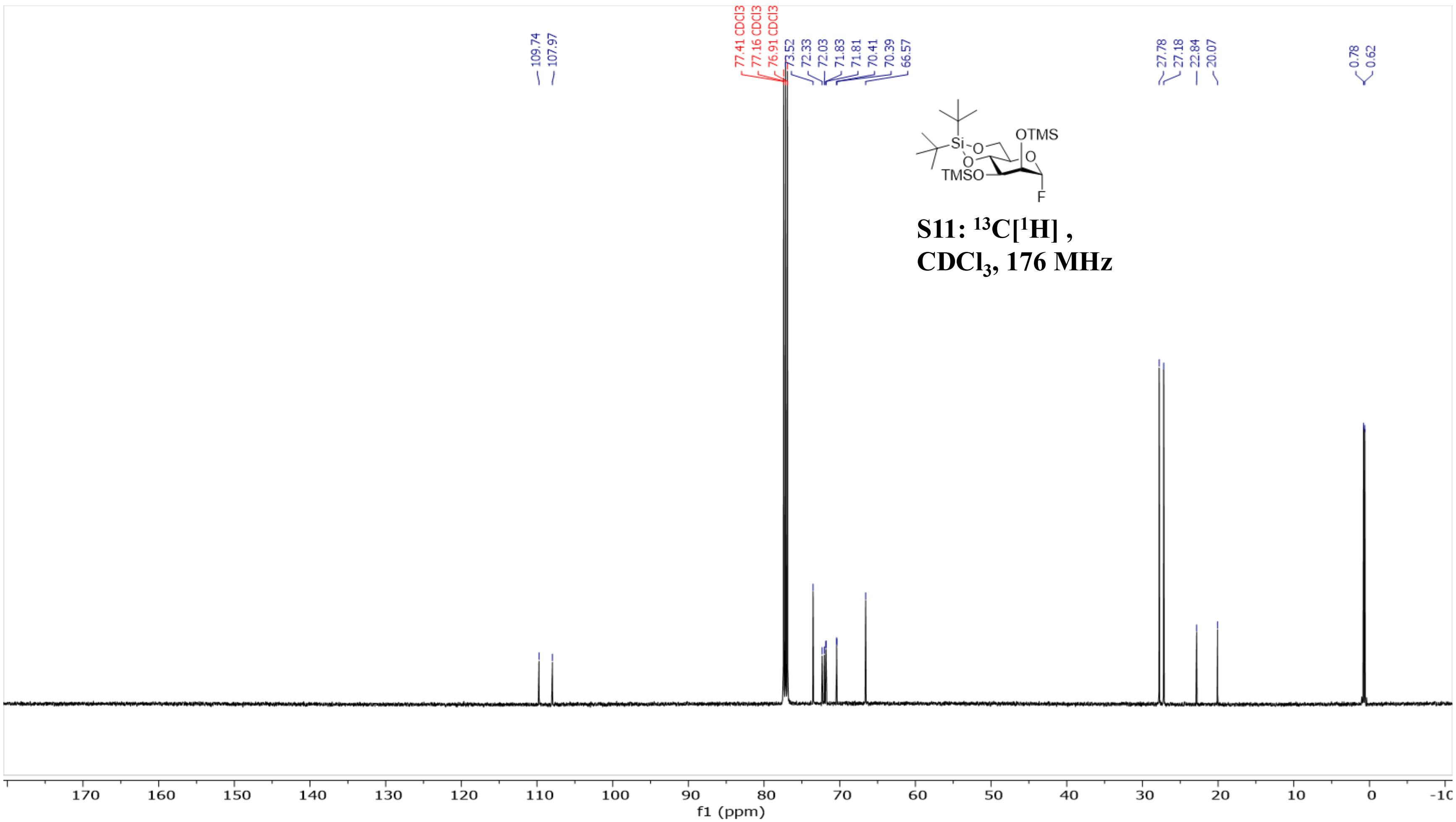
109.74
107.97
77.41 CDCl3
77.16 CDCl3
76.91 CDCl3
73.52
72.33
72.03
71.83
71.81
70.41
70.39
66.57
27.78
27.18
22.84
20.07
0.78
0.62
OTMS
Si
O
O
TMSO
F
S11: $^{13}C[^{1}H]$,
$CDCl_3$, 176 MHz
170 160 150 140 130 120 110 100 90 80 70 60 50 40 30 20 10 0 -10
f1 (ppm)

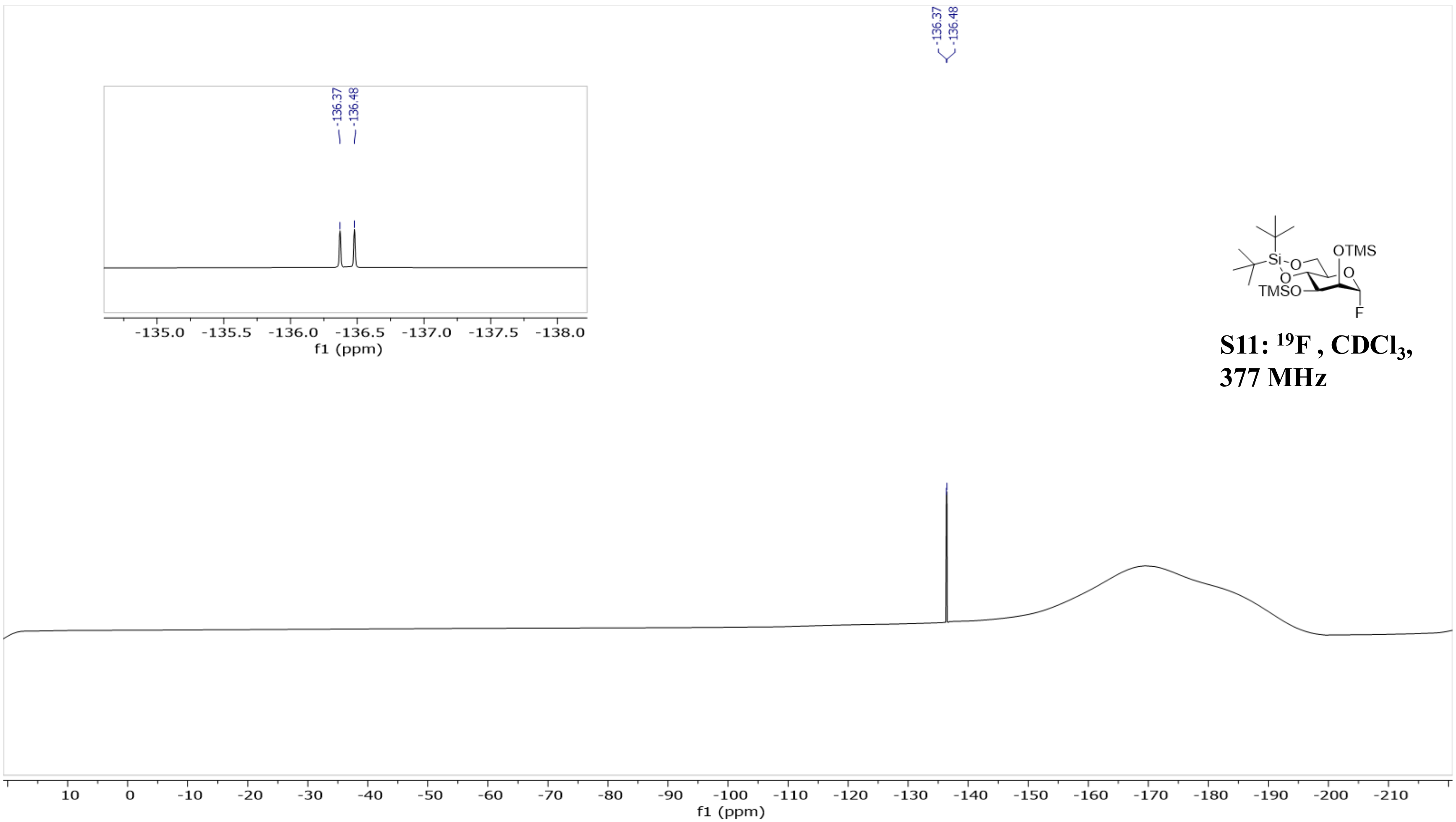

S11: ^{19}F , $CDCl_3$, 377 MHz

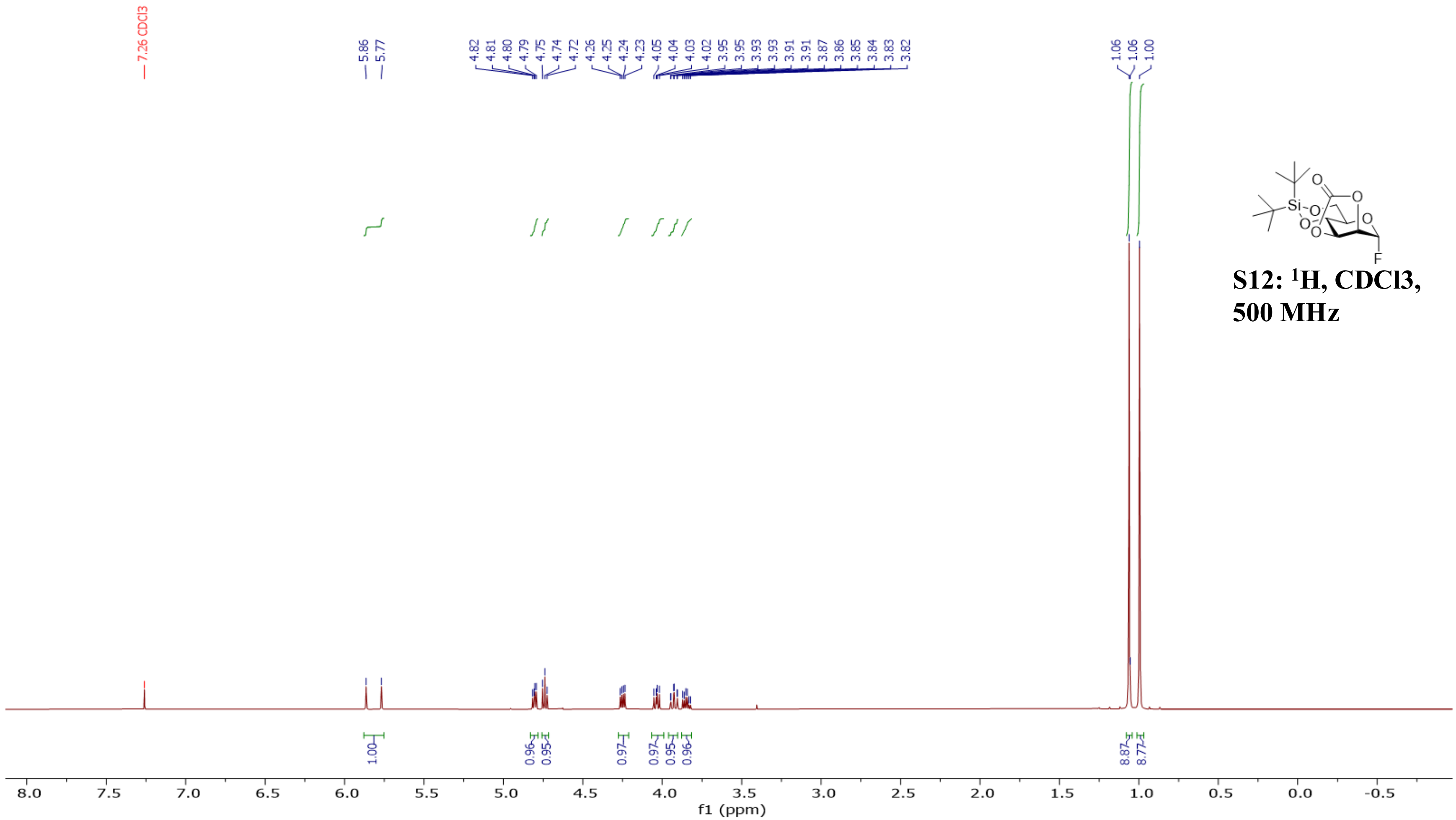

S12: ^{1}H, CDCl3, 500 MHz

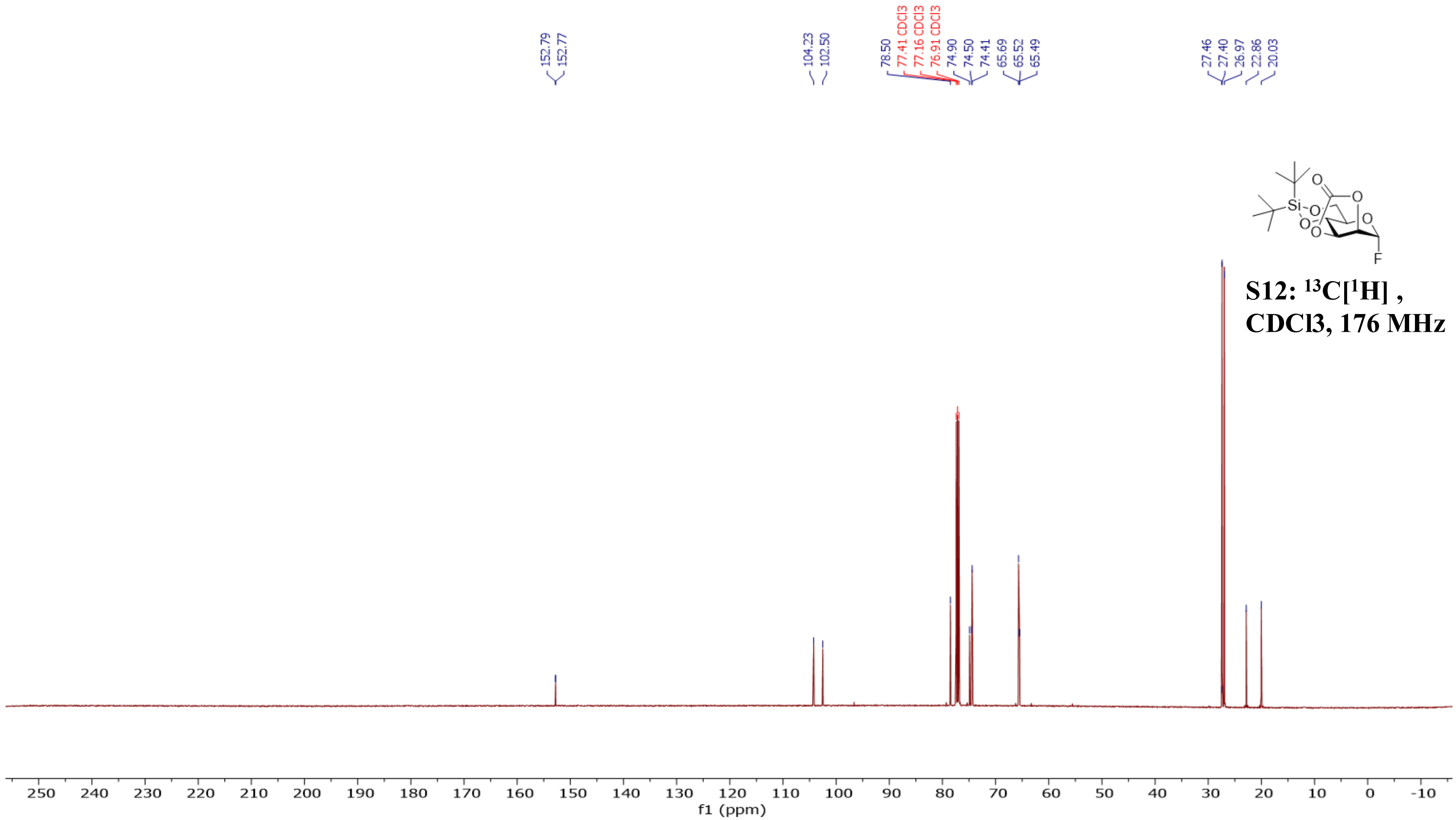

S12: $^{13}C[^{1}H]$, CDCl3, 176 MHz

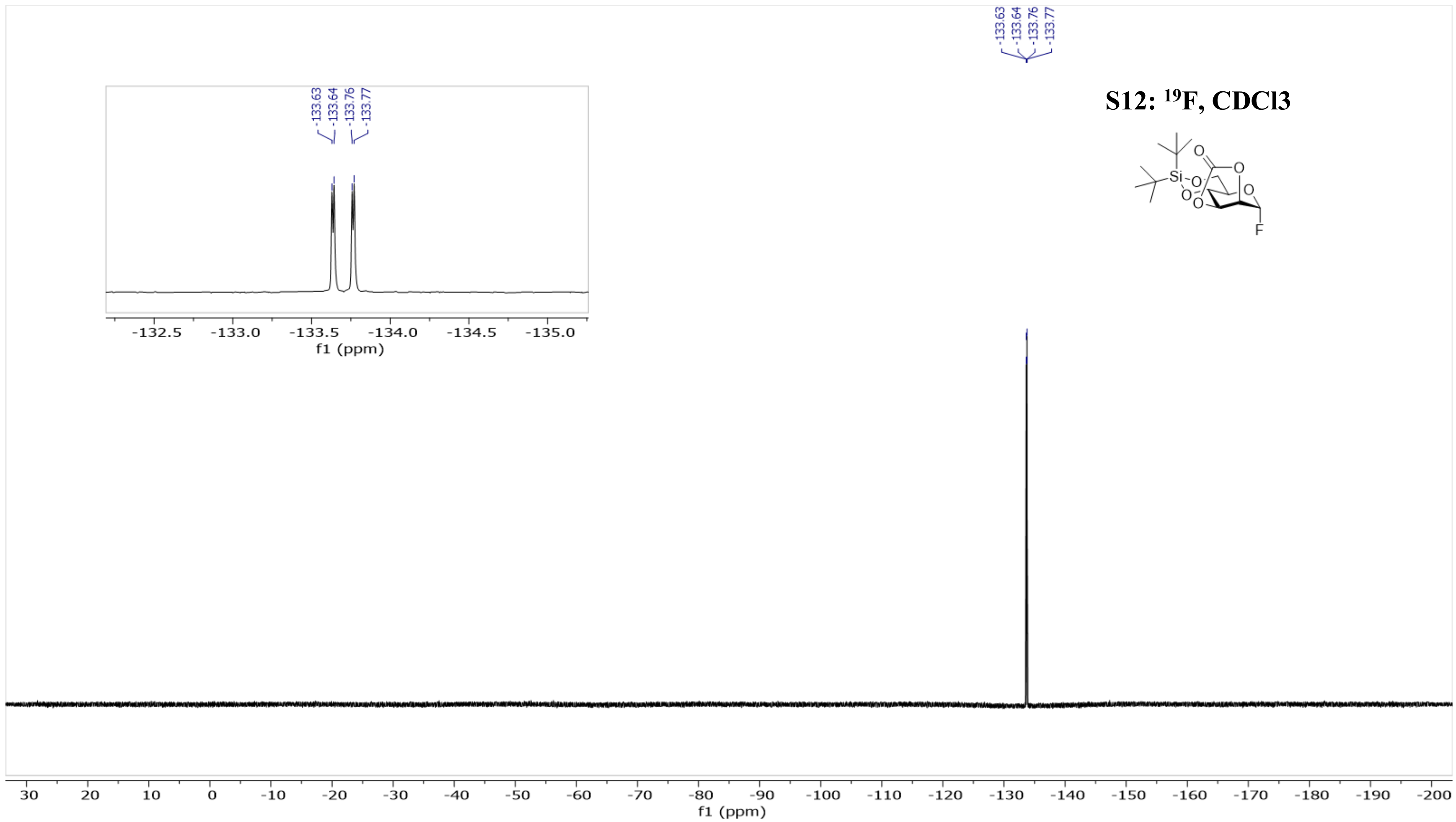
S12: ¹⁹F, CDCl3
-133.63
-133.64
-133.76
-133.77
-132.5
-133.0
-133.5
-134.0
-134.5
-135.0
f1 (ppm)
30
20
10
0
-10
-20
-30
-40
-50
-60
-70
-80
-90
-100
-110
-120
-130
-140
-150
-160
-170
-180
-190
-200
f1 (ppm)
Si
O
F

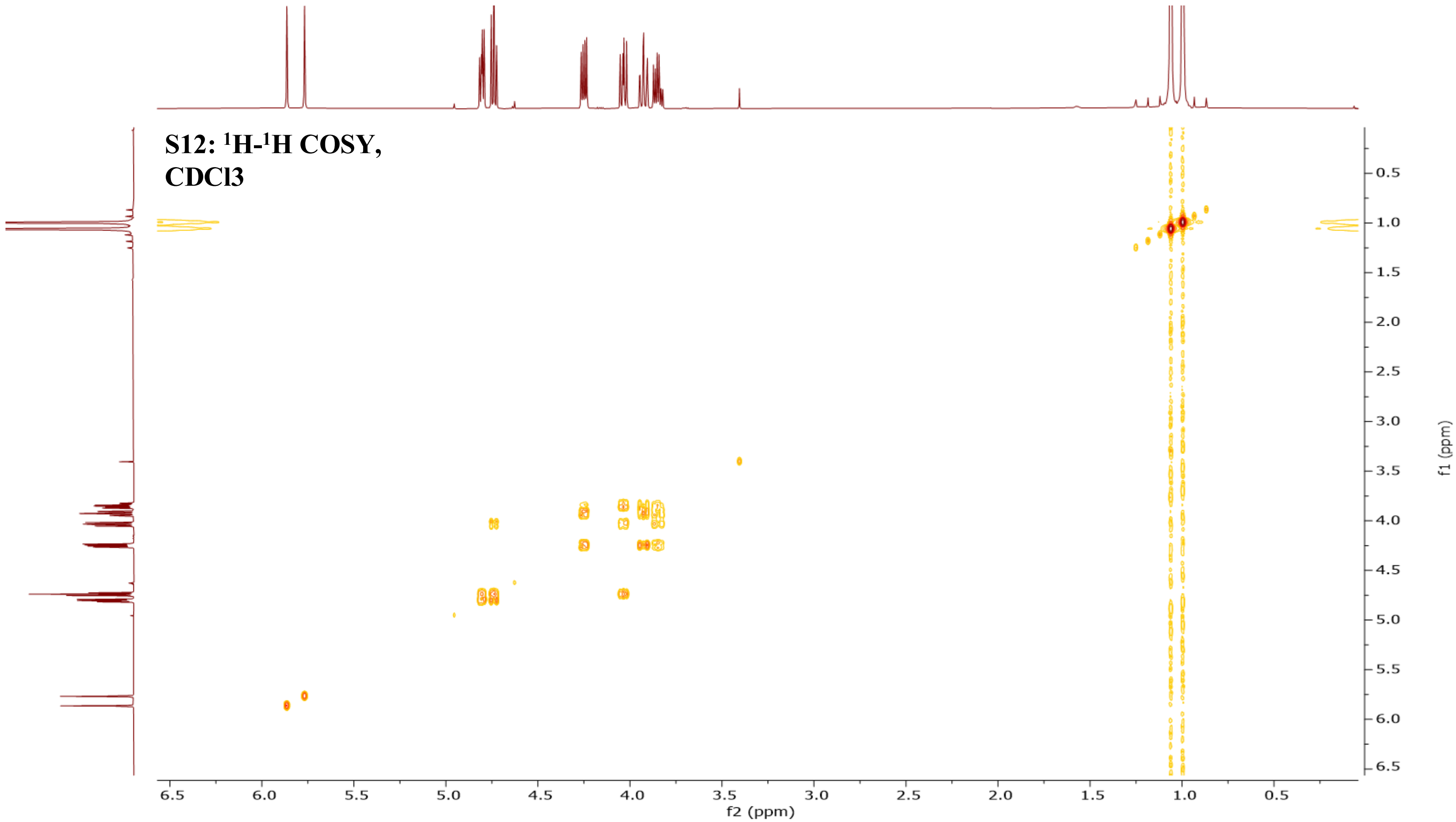
S12: 1H-1H COSY,
CDCl3
f2 (ppm)
f1 (ppm)
6.5
6.0
5.5
5.0
4.5
4.0
3.5
3.0
2.5
2.0
1.5
1.0
0.5

S12: ^{1}H-^{13}C HSQC, CDCl3

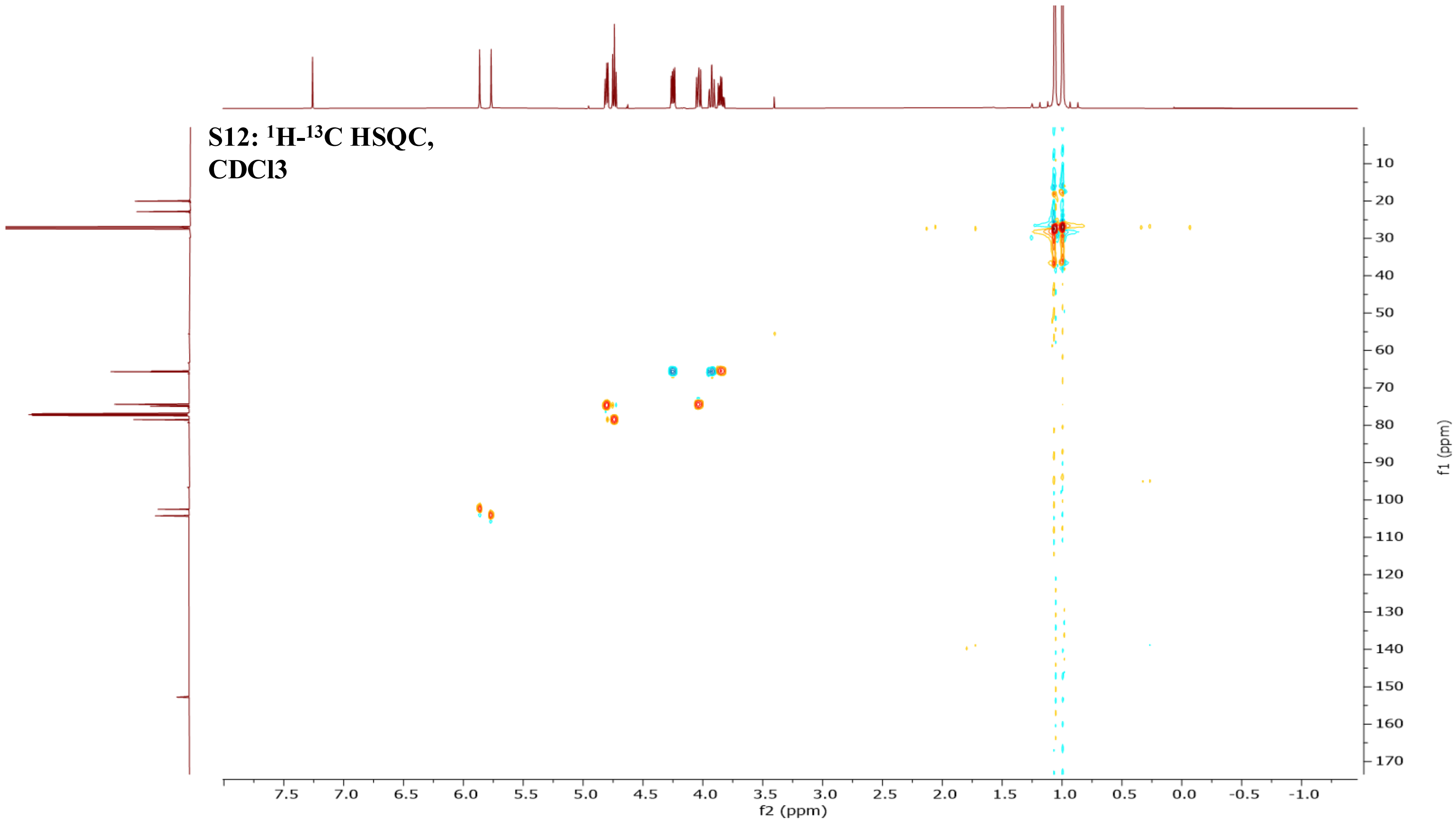

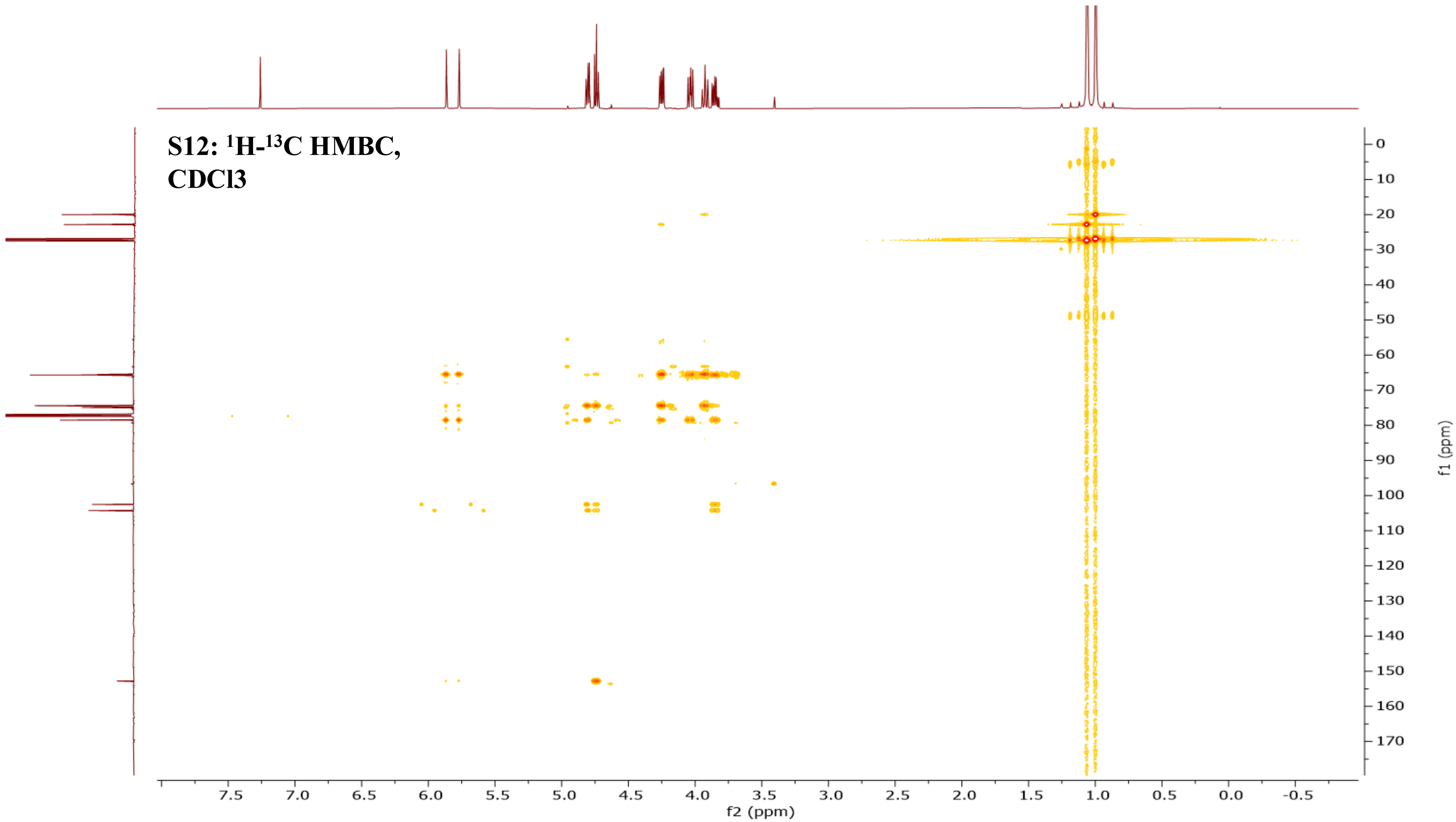

S12: 1H-13C HMBC,
CDCl3
f2 (ppm)
f1 (ppm)

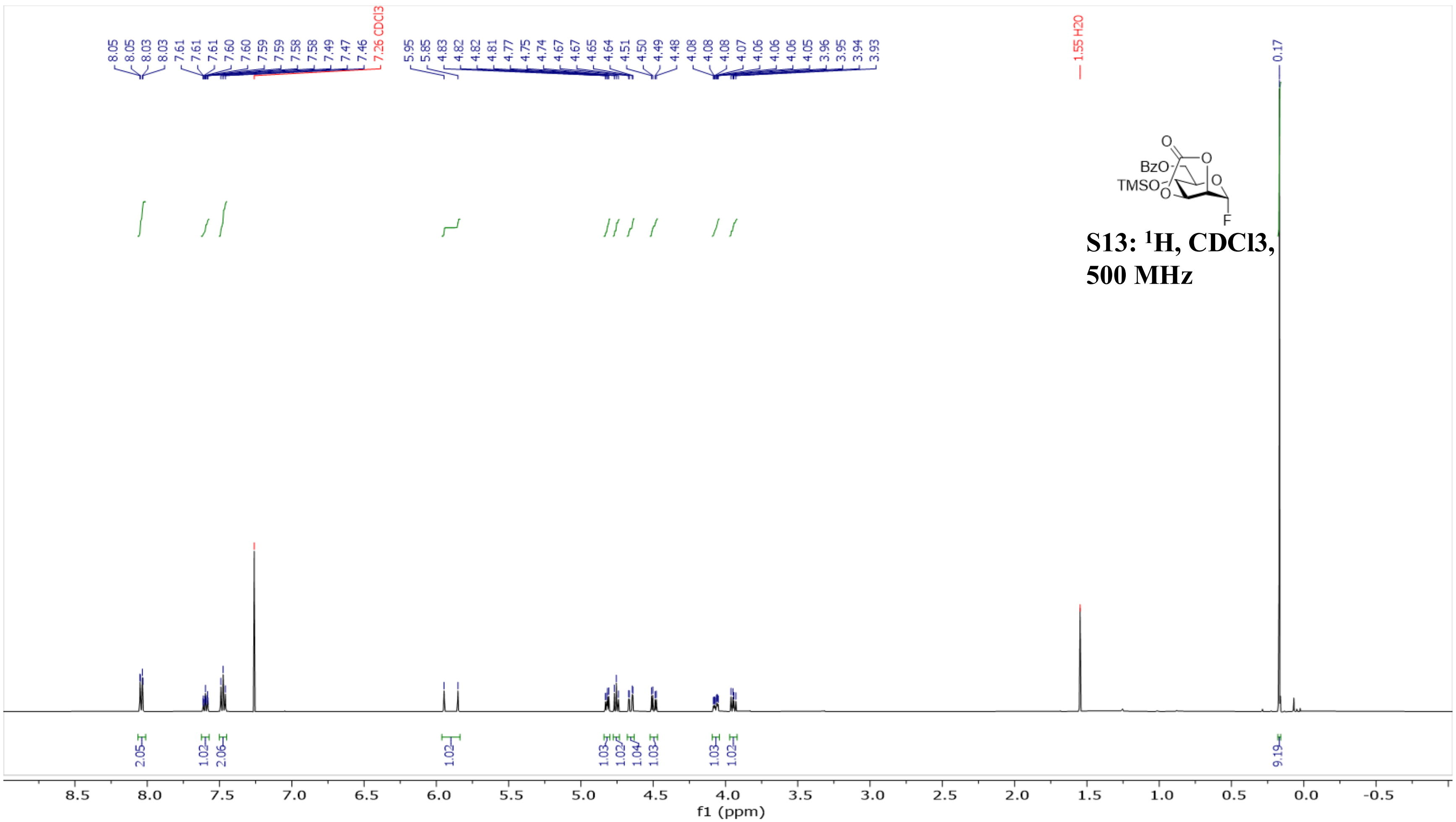

S13: ^{1}H, CDCl3, 500 MHz

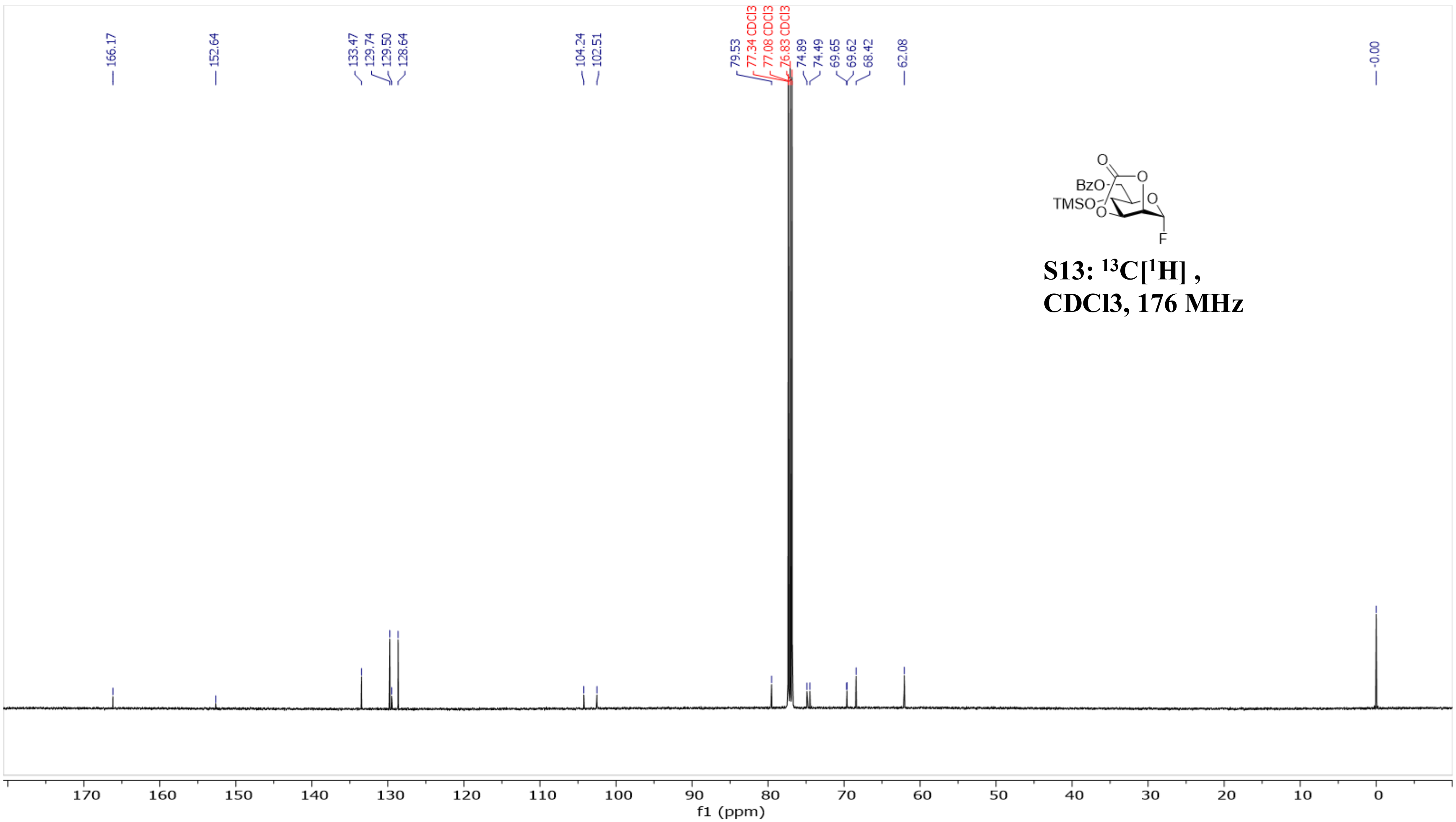

**S13: $^{13}C[^{1}H]$,
CDCl3, 176 MHz**

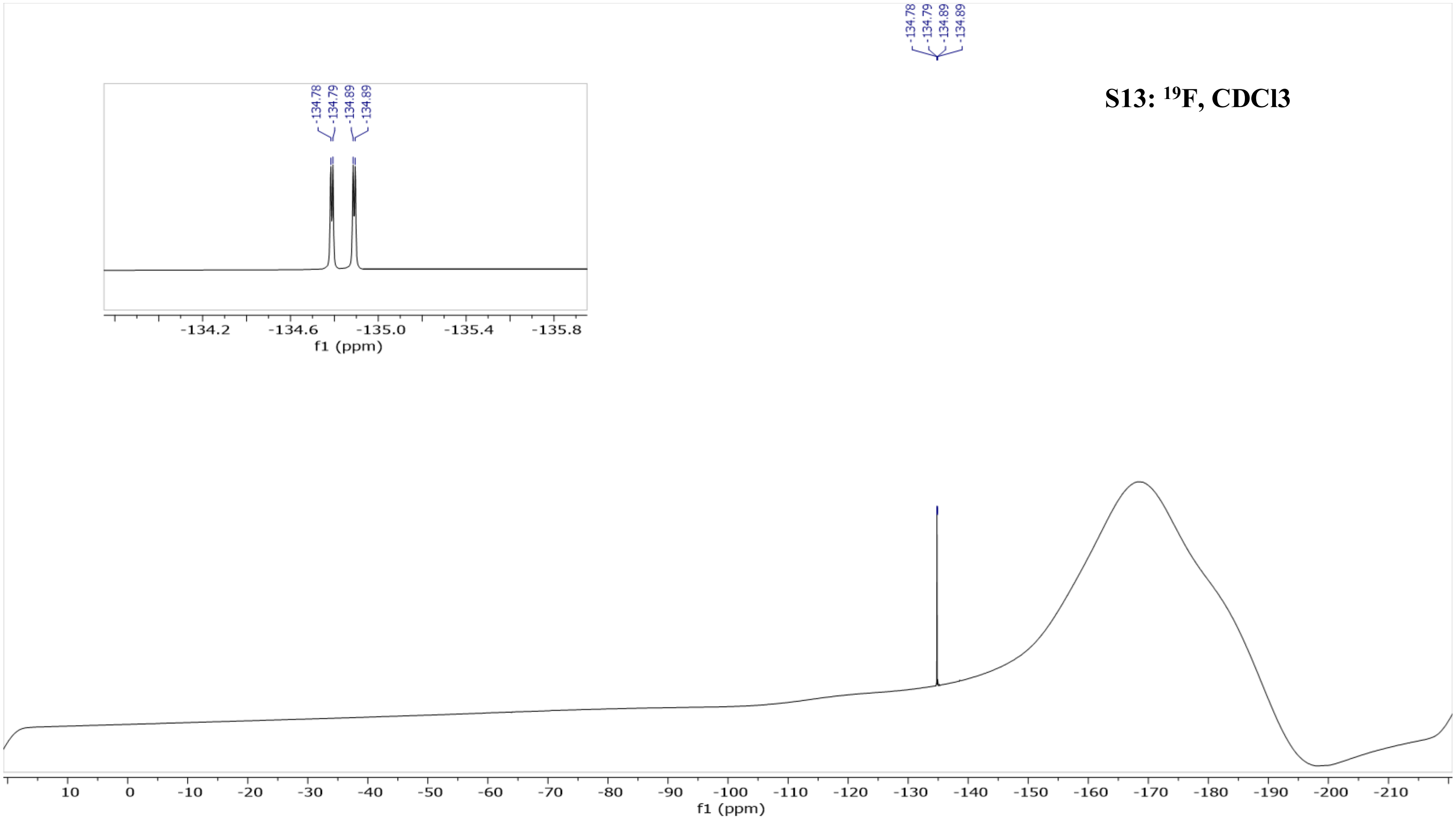
S13: ^{19}F, CDCl3
-134.78
-134.79
-134.89
-134.89
-134.2
-134.6
-135.0
-135.4
-135.8
f1 (ppm)
10 0 -10 -20 -30 -40 -50 -60 -70 -80 -90 -100 -110 -120 -130 -140 -150 -160 -170 -180 -190 -200 -210
f1 (ppm)

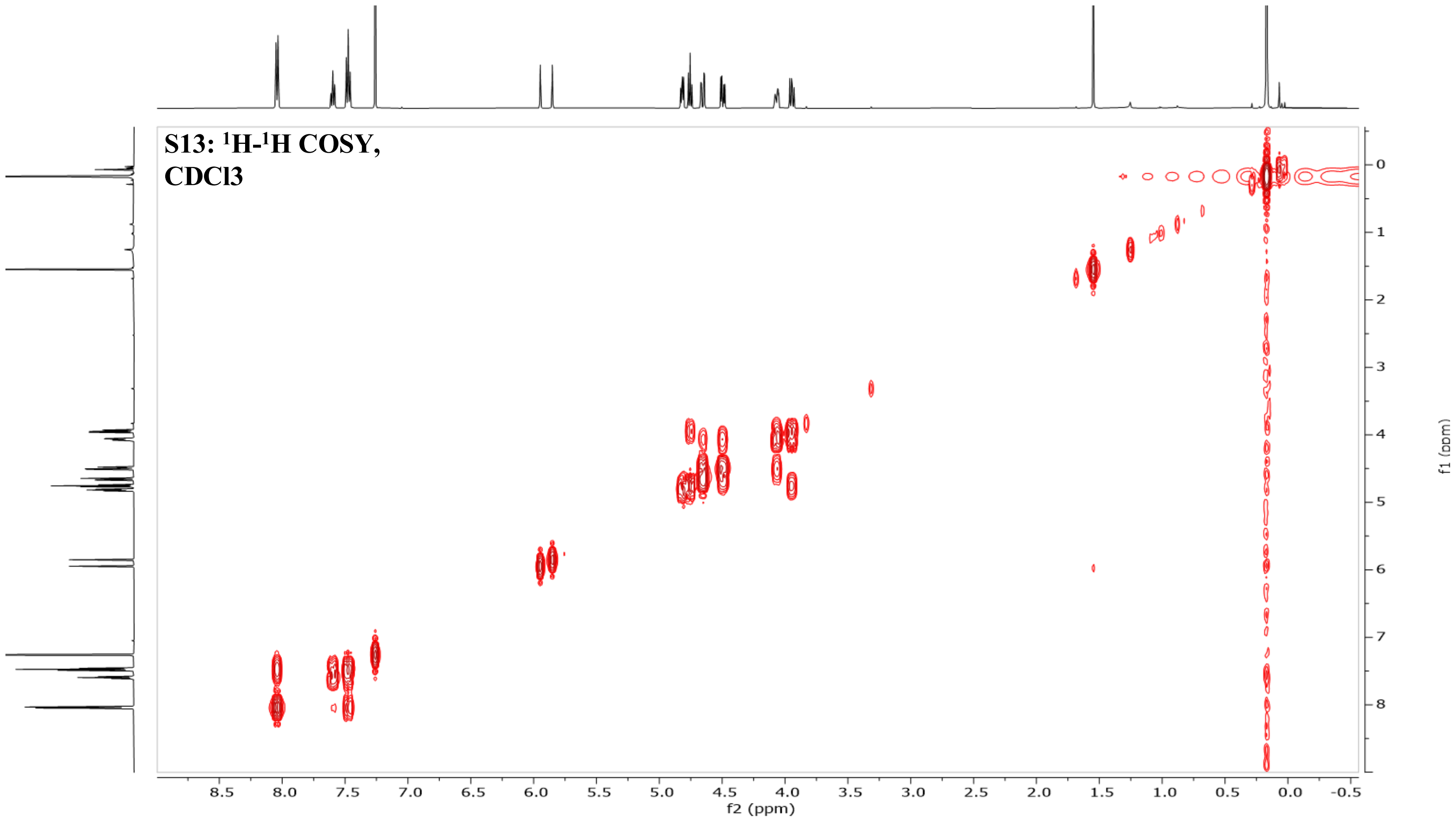
S13: ¹H-¹H COSY,
CDCl3
f2 (ppm)
f1 (ppm)

S13: ^{1}H-^{13}C HSQC, CDCl3

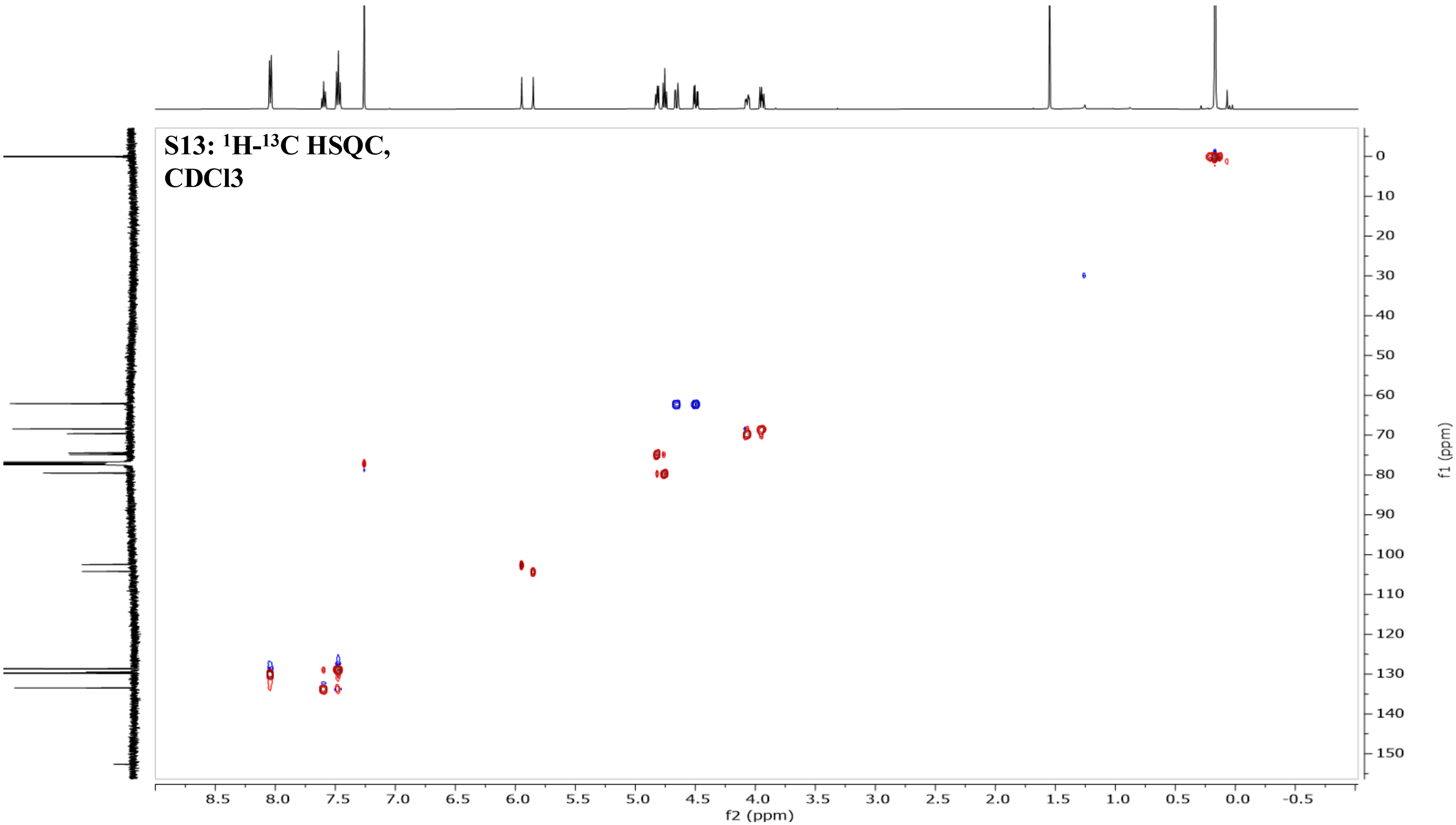

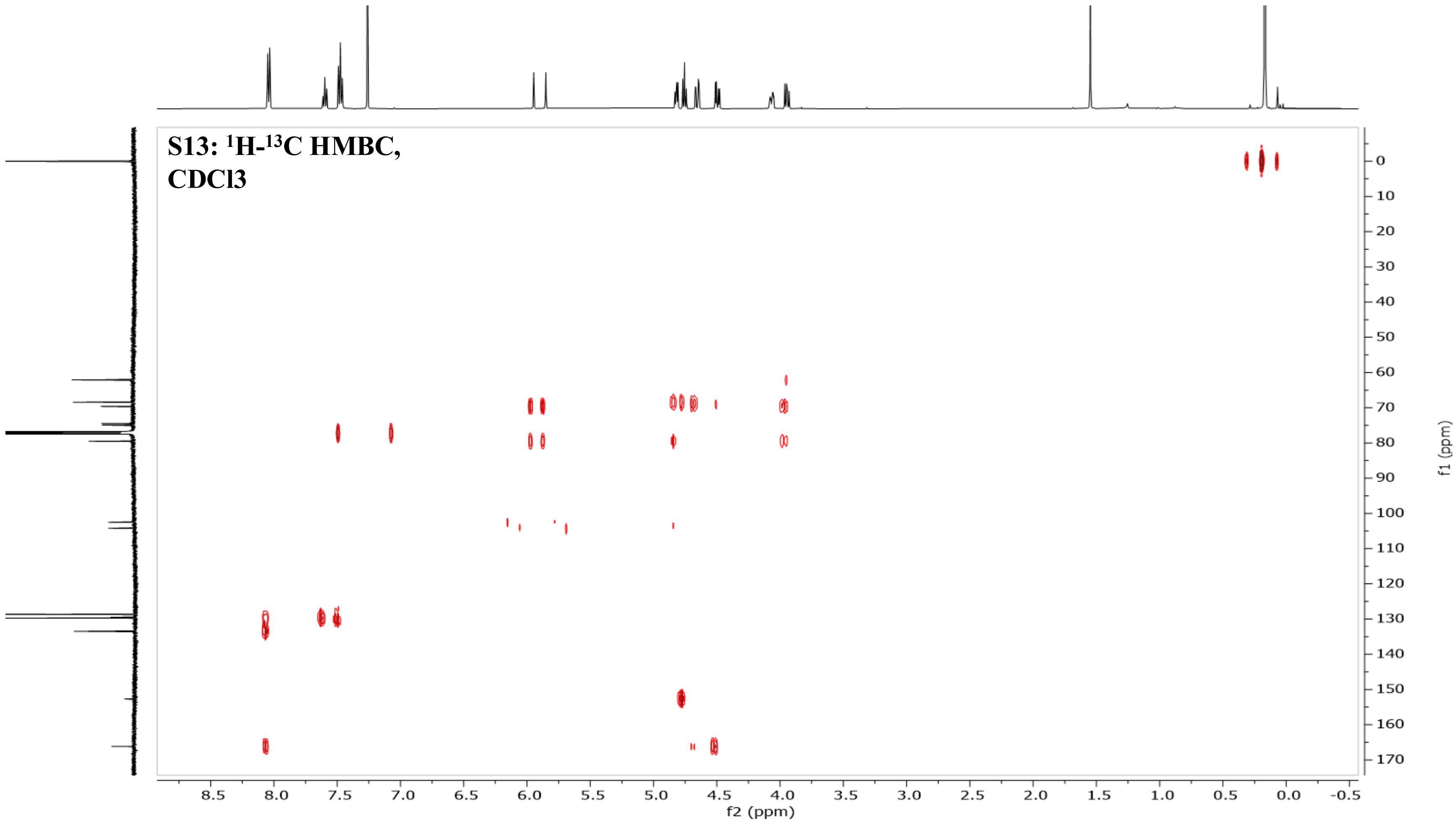
S13: ^{1}H-^{13}C HMBC,
CDCl3
f2 (ppm)
f1 (ppm)

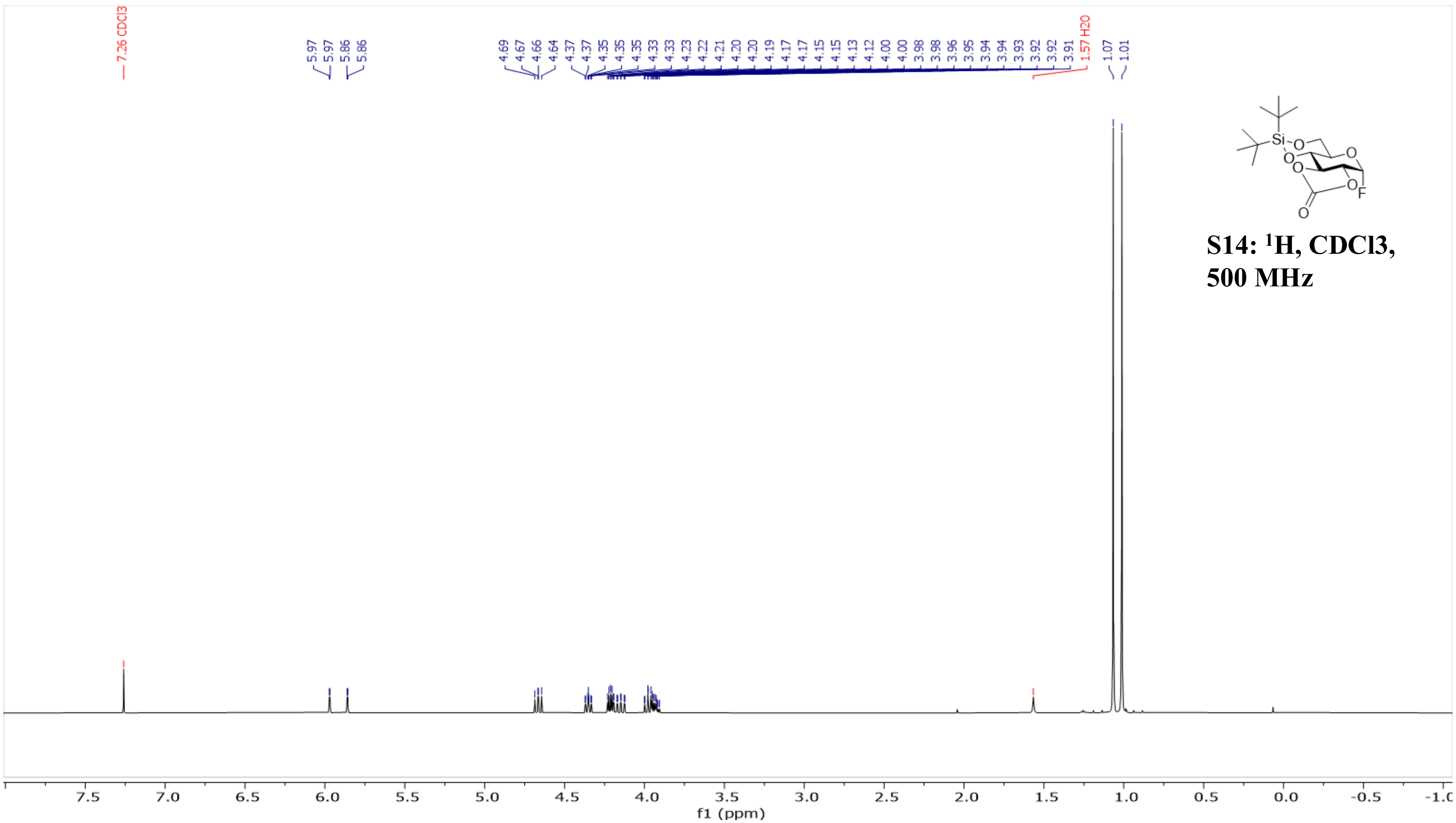

S14: ^{1}H, CDCl3, 500 MHz

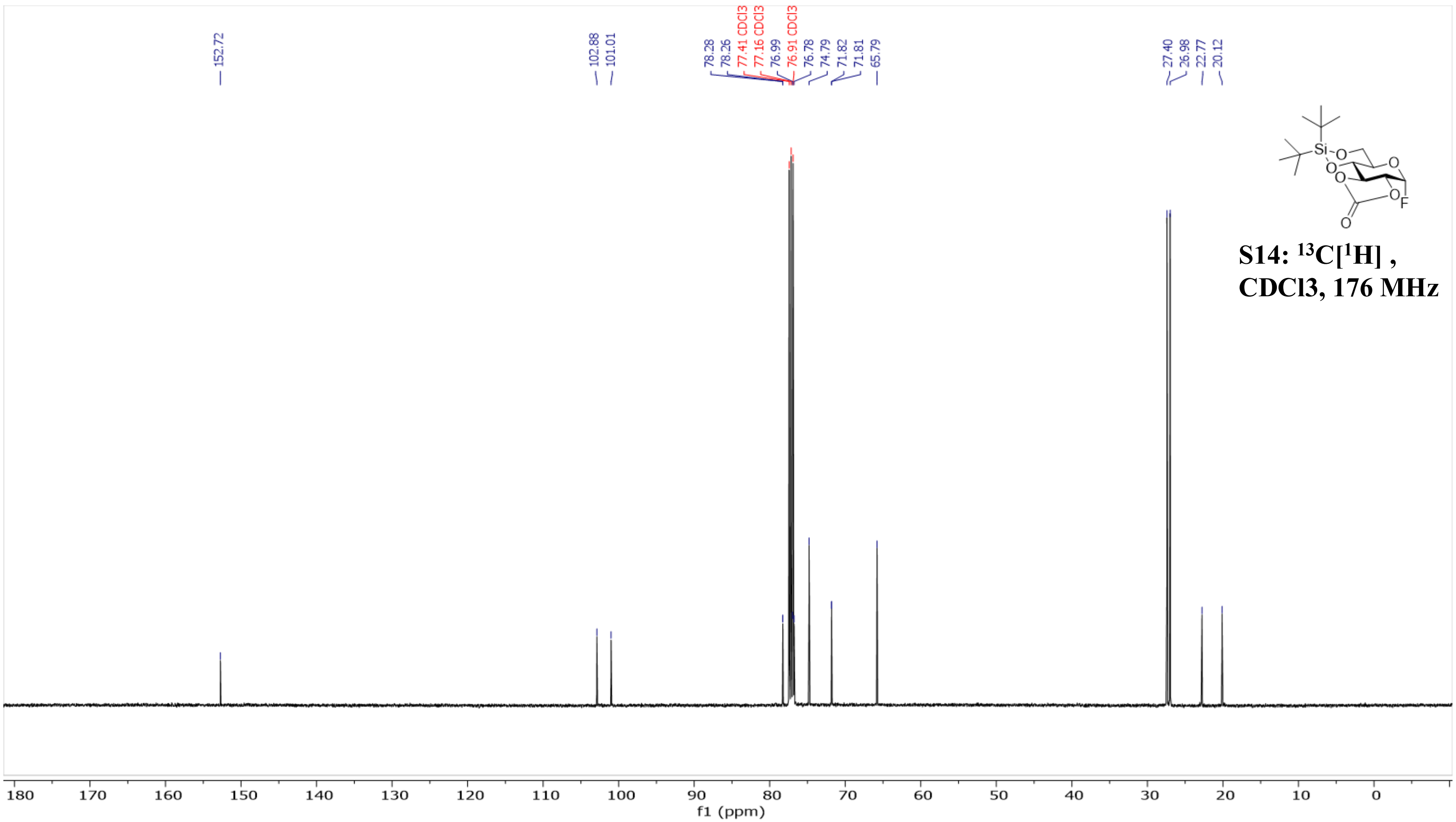

S14: $^{13}C[^{1}H]$, CDCl3, 176 MHz

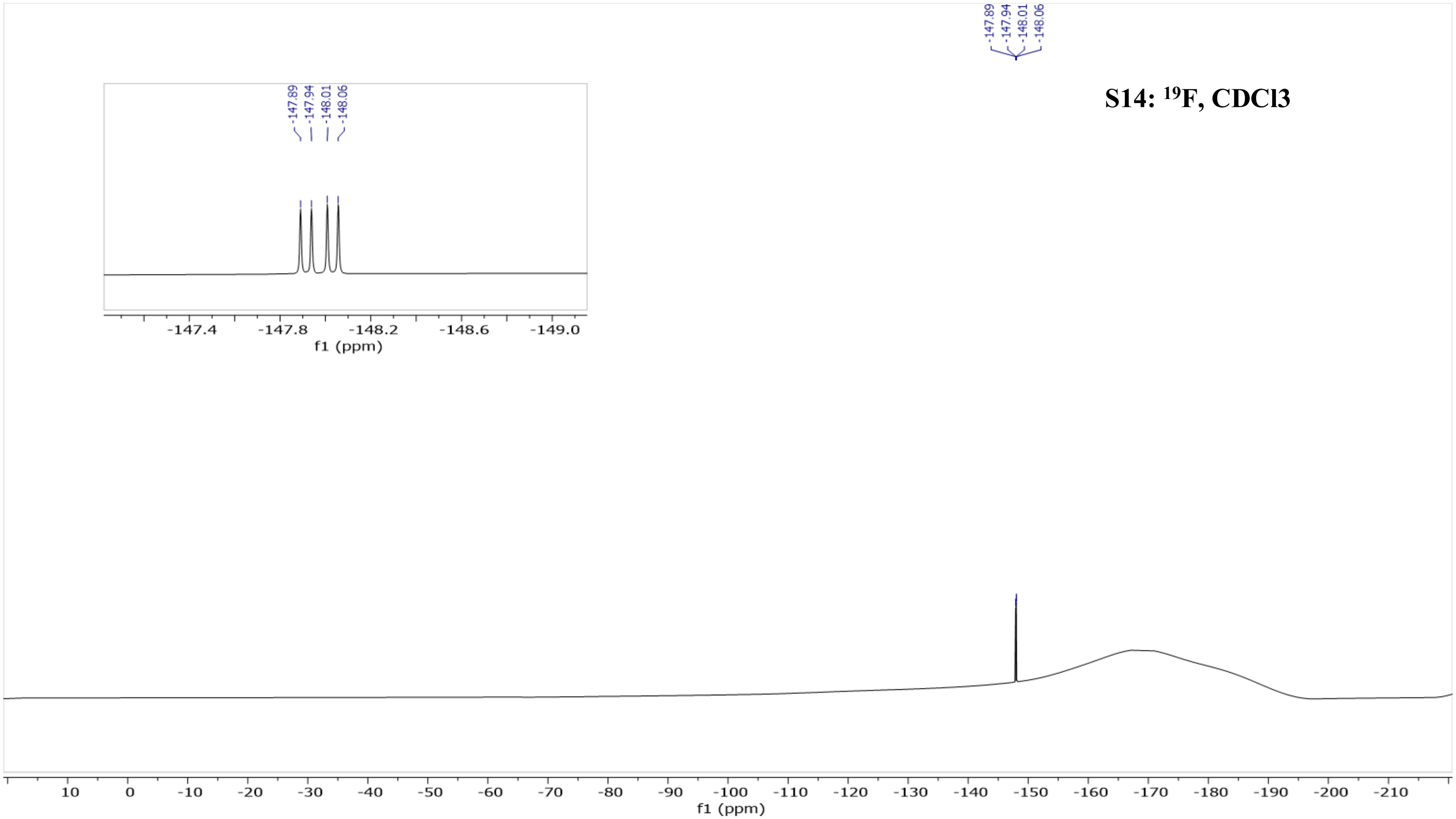
S14: ^{19}F, CDCl3
-147.89
-147.94
-148.01
-148.06
-147.4
-147.8
-148.2
-148.6
-149.0
f1 (ppm)
10
0
-10
-20
-30
-40
-50
-60
-70
-80
-90
-100
-110
-120
-130
-140
-150
-160
-170
-180
-190
-200
-210
f1 (ppm)

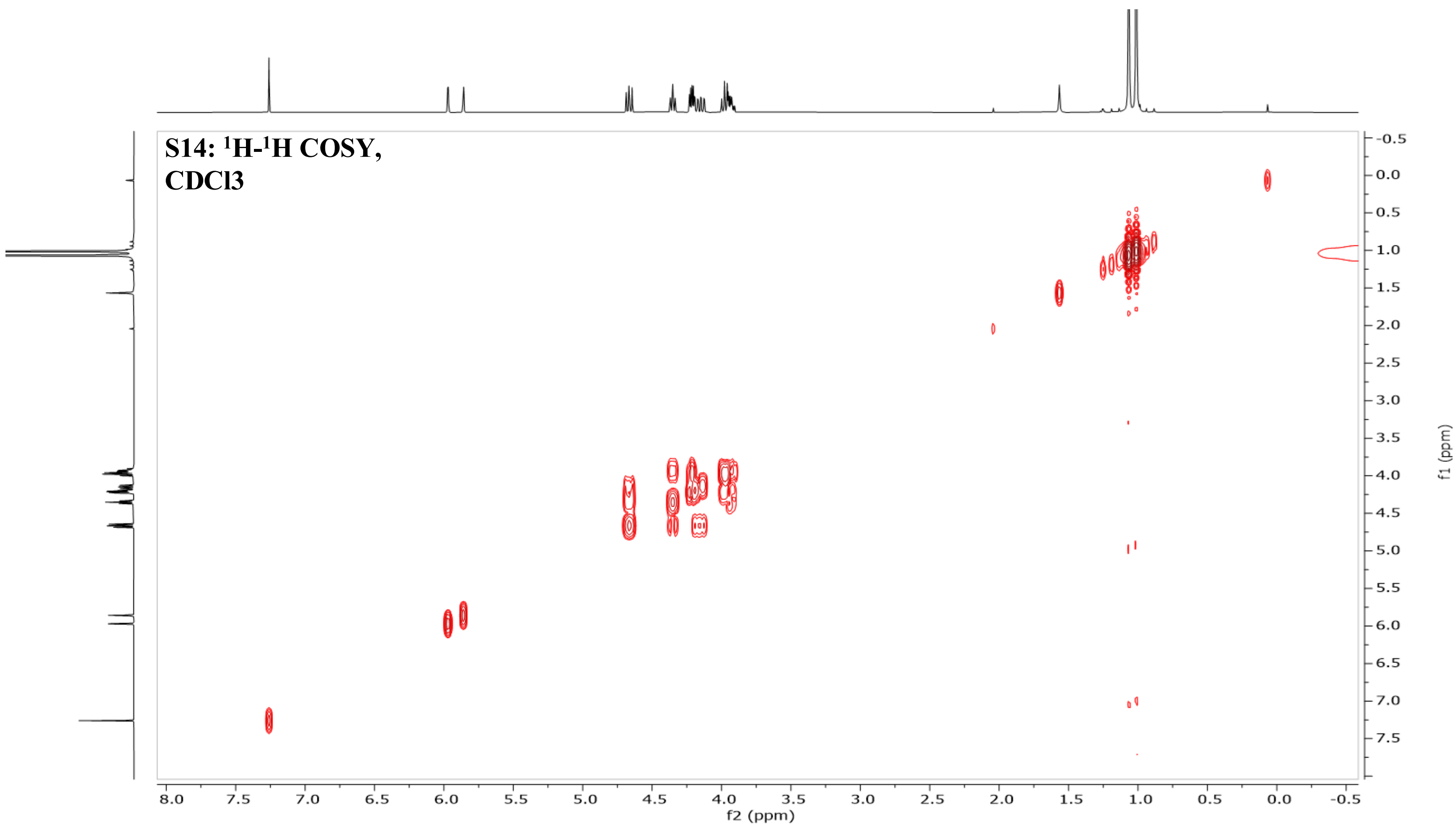

S14: 1H-1H COSY,
CDCl3
f2 (ppm)
f1 (ppm)

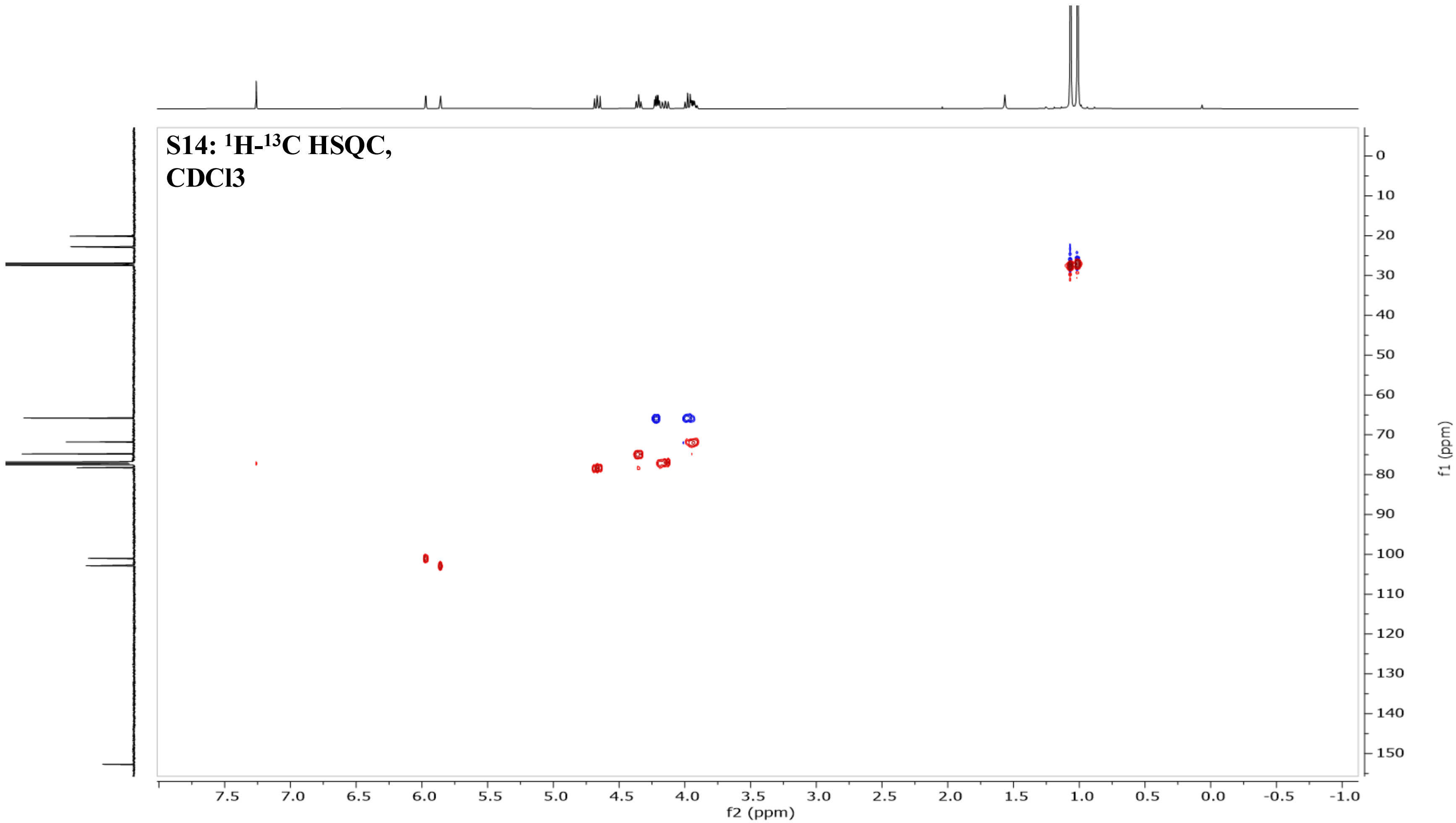
S14: ^1H-^{13}C HSQC,
CDCl3
f2 (ppm)
f1 (ppm)

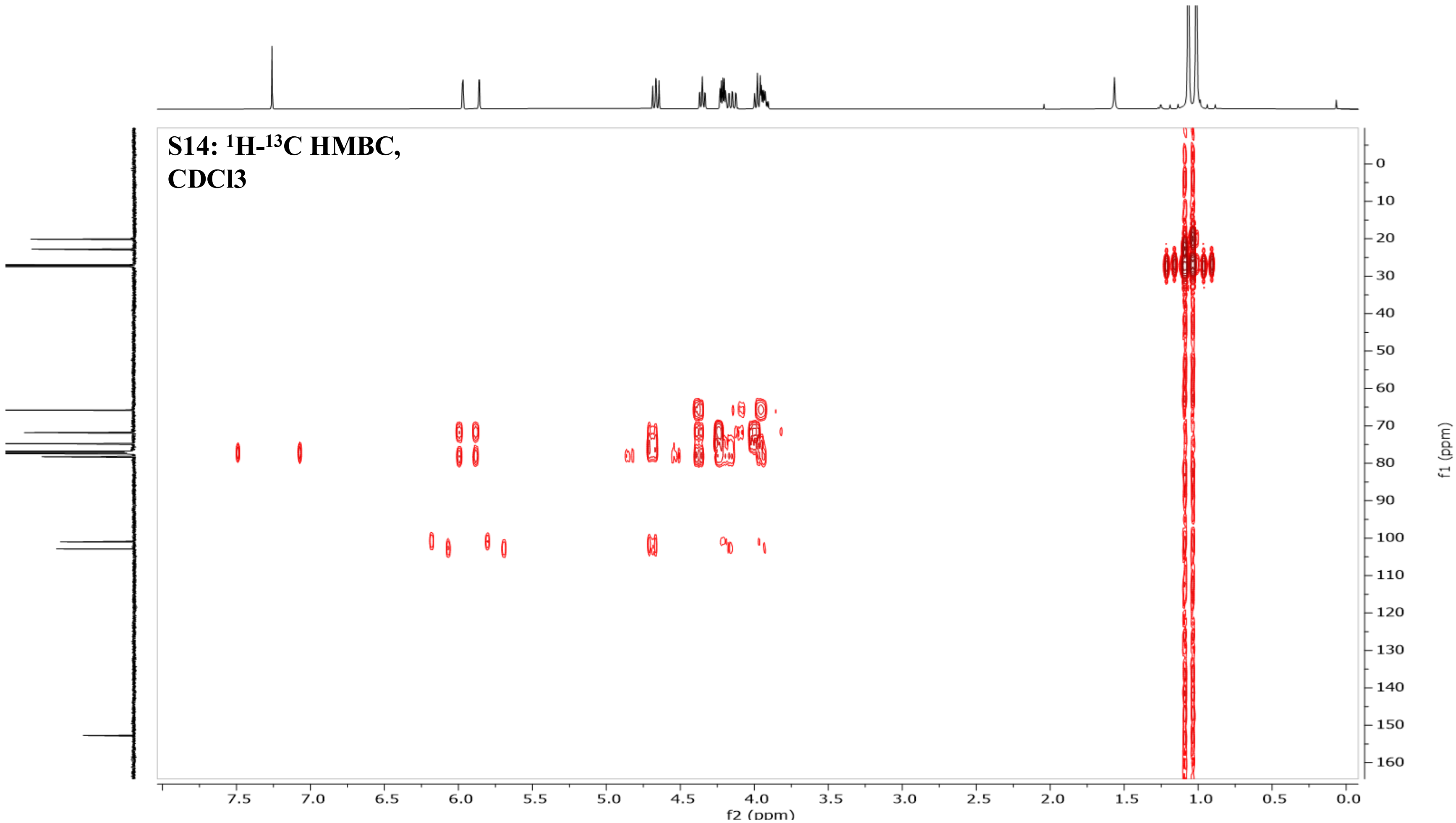
S14: 1H-13C HMBC,
CDCl3
f2 (ppm)
f1 (ppm)

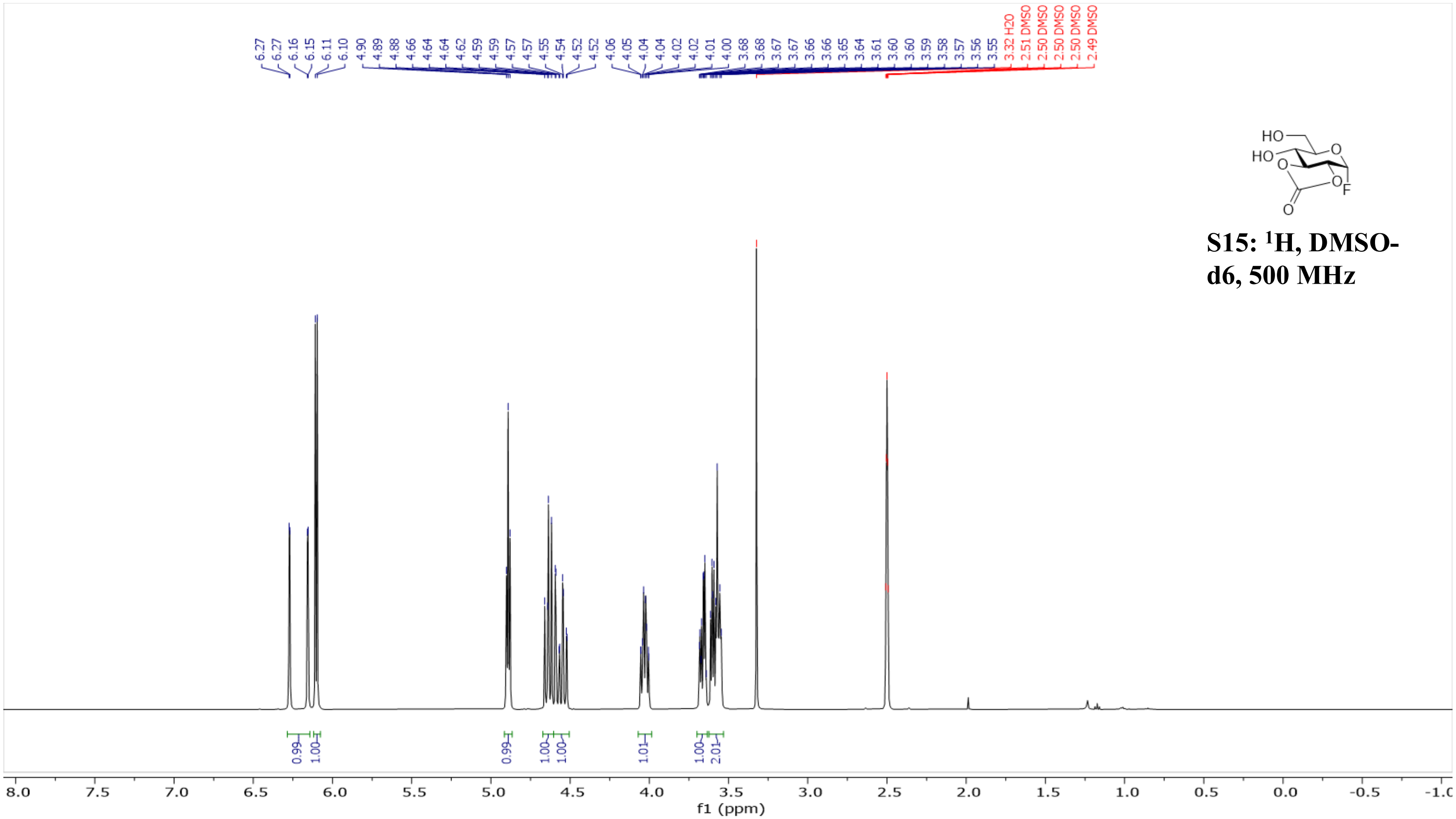

S15: ^1H, DMSO-d6, 500 MHz

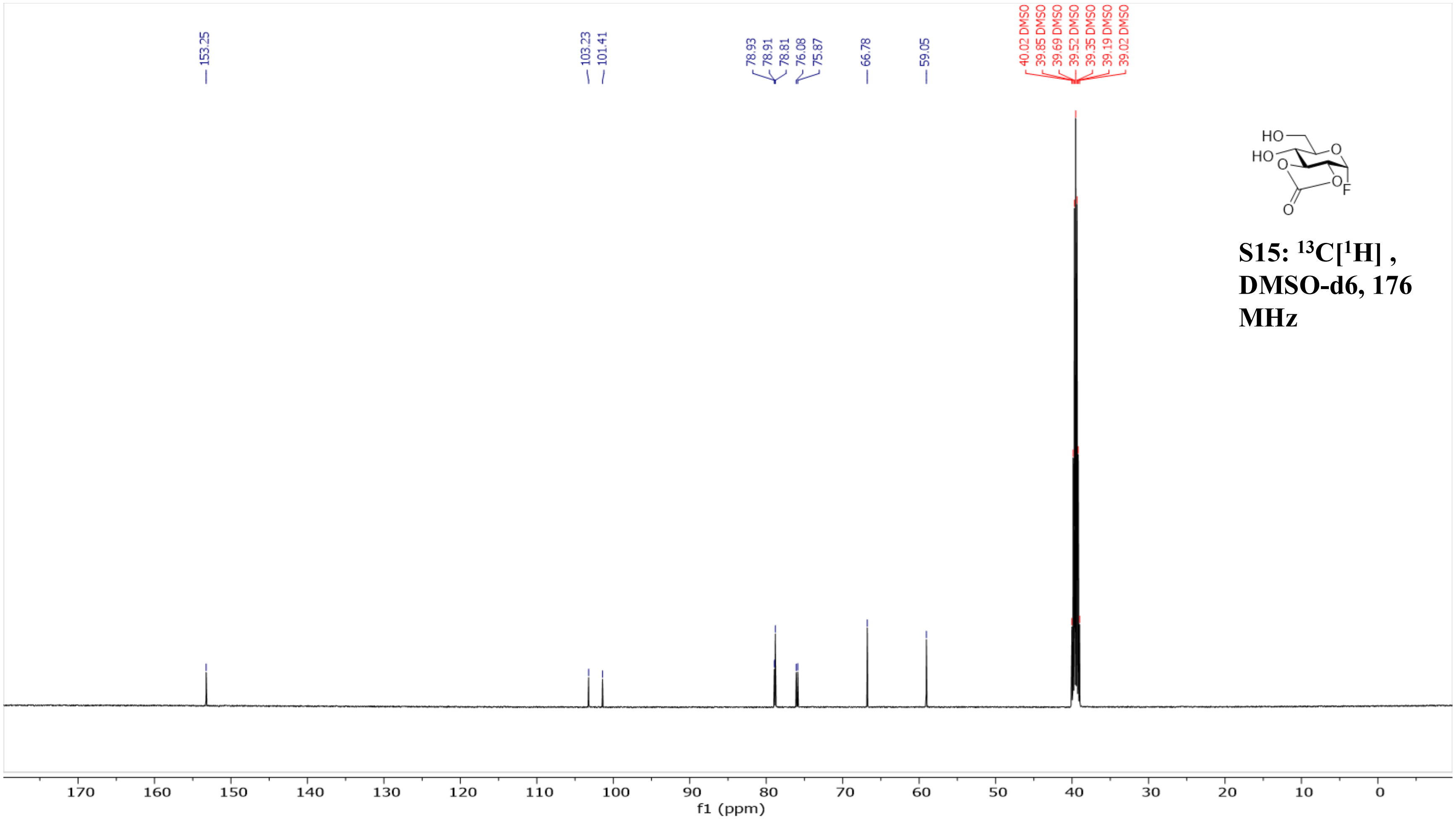

S15: $^{13}C[^{1}H]$, DMSO-d6, 176 MHz

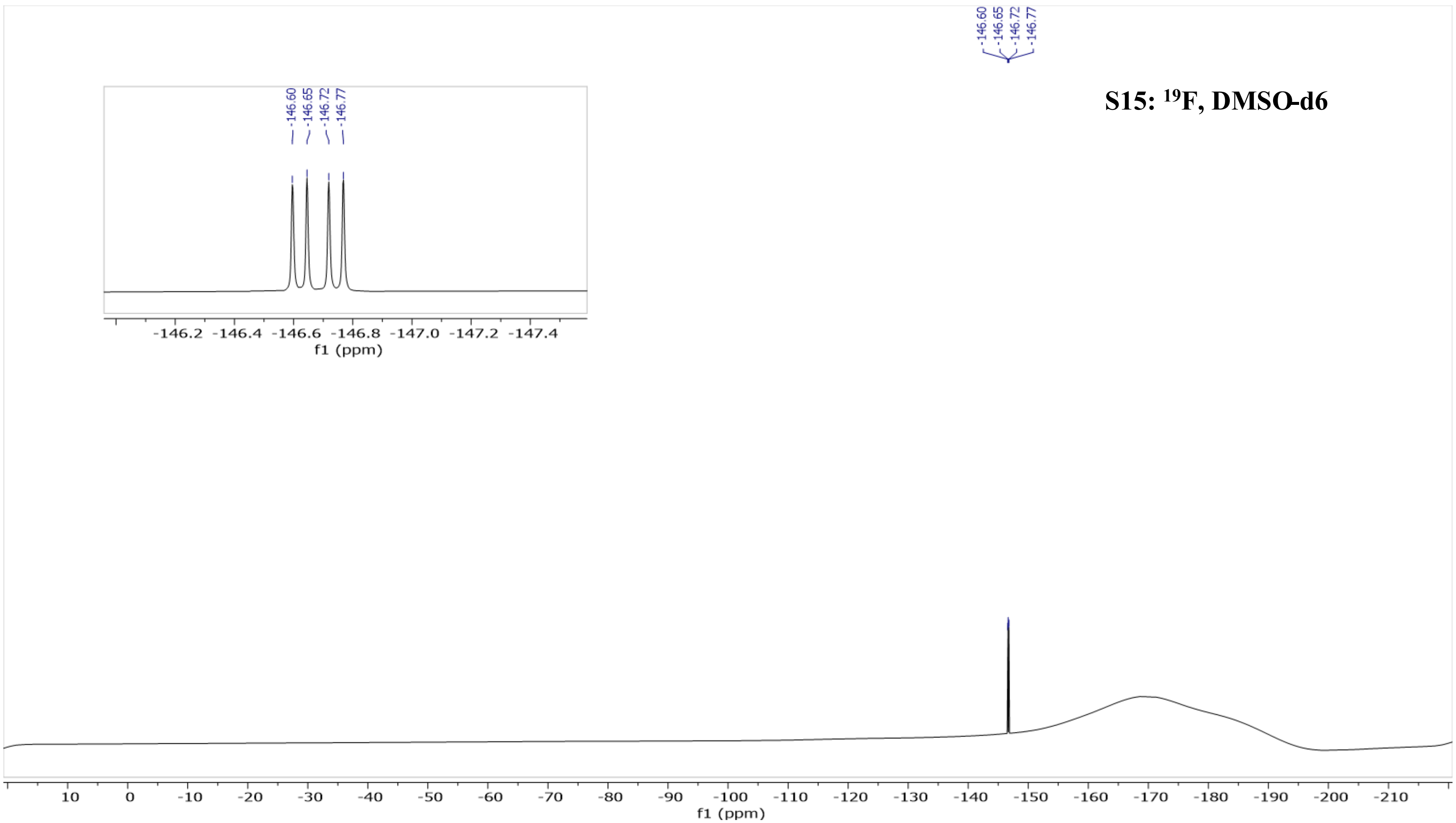

S15: 19F, DMSO-d6
-146.60
-146.65
-146.72
-146.77
-146.2 -146.4 -146.6 -146.8 -147.0 -147.2 -147.4
f1 (ppm)
10 0 -10 -20 -30 -40 -50 -60 -70 -80 -90 -100 -110 -120 -130 -140 -150 -160 -170 -180 -190 -200 -210
f1 (ppm)

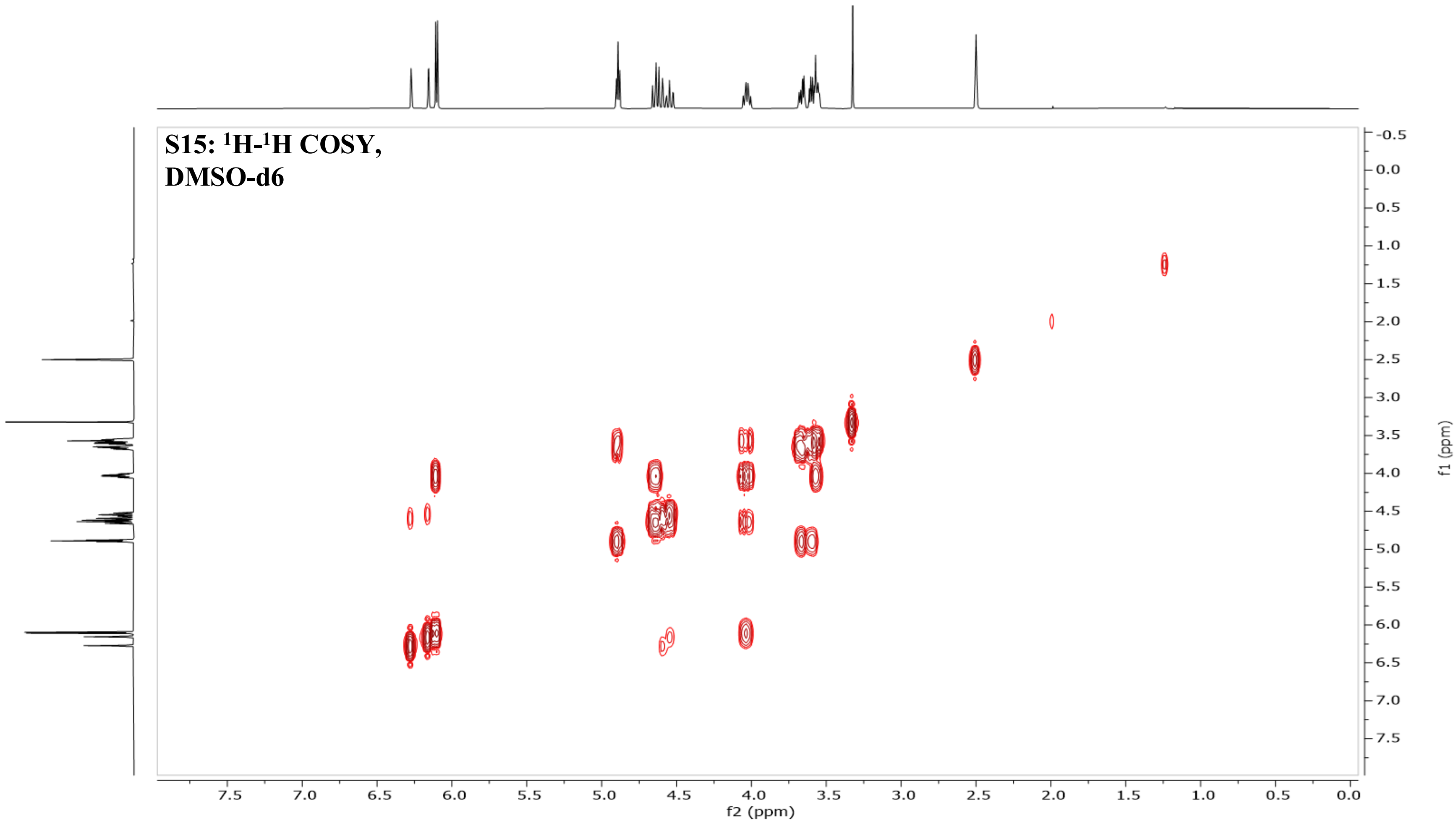
S15: ^1H-^1H COSY,
DMSO-d6
f2 (ppm)
f1 (ppm)
7.5
7.0
6.5
6.0
5.5
5.0
4.5
4.0
3.5
3.0
2.5
2.0
1.5
1.0
0.5
0.0
-0.5

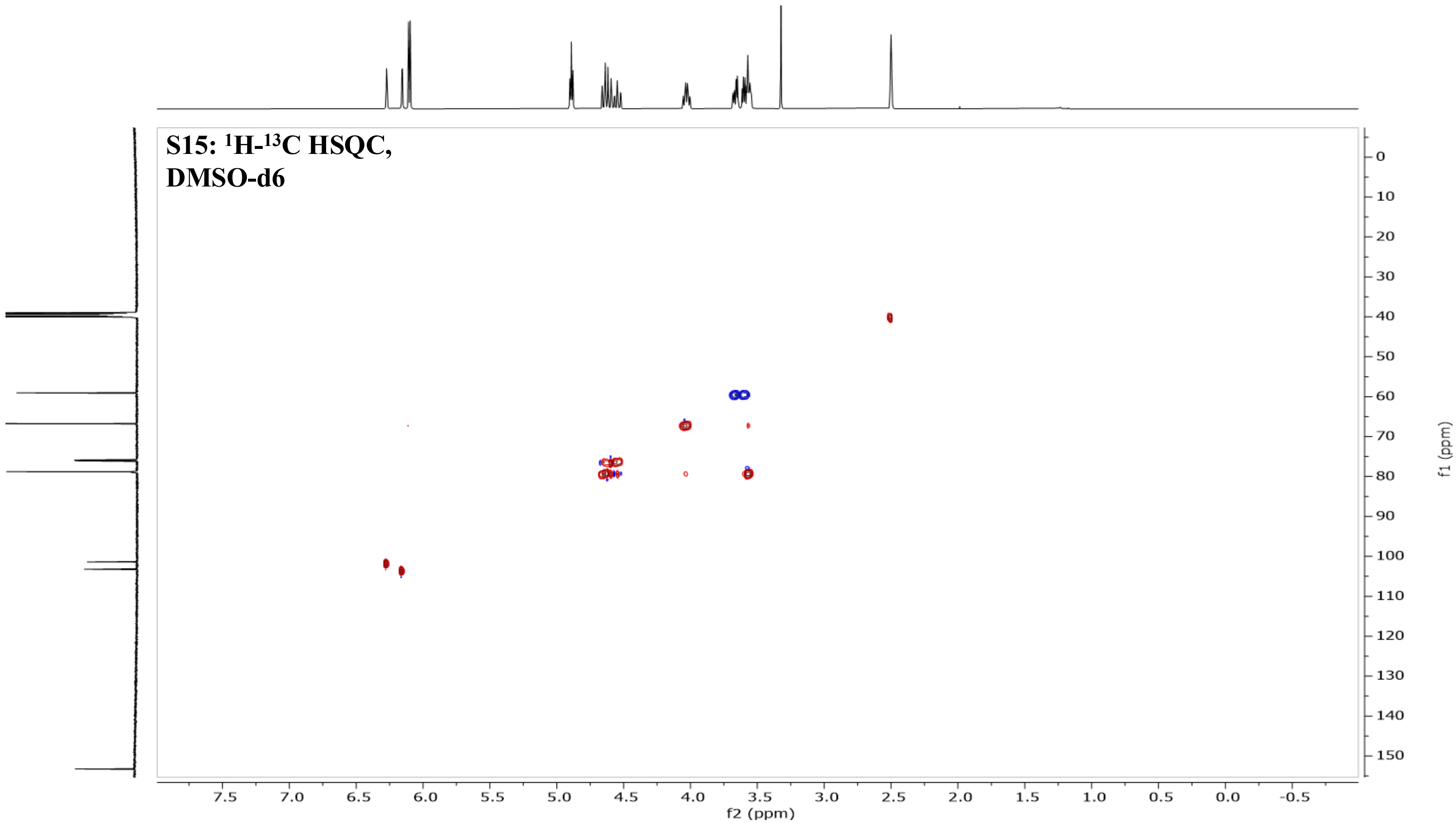
S15: 1H-13C HSQC,
DMSO-d6
f2 (ppm)
f1 (ppm)

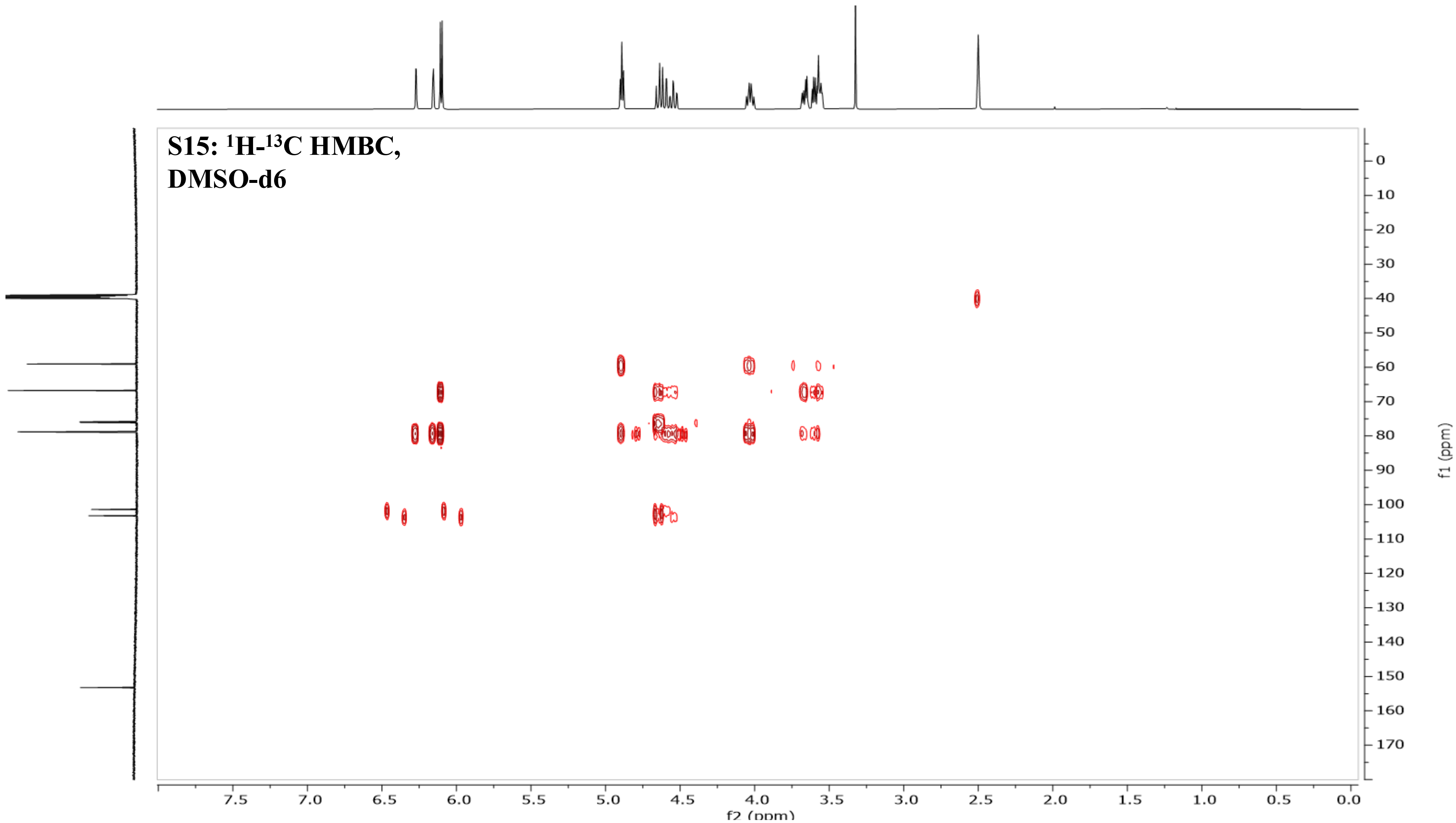
S15: 1H-13C HMBC,
DMSO-d6
f2 (ppm)
f1 (ppm)
7.5
7.0
6.5
6.0
5.5
5.0
4.5
4.0
3.5
3.0
2.5
2.0
1.5
1.0
0.5
0.0
0
10
20
30
40
50
60
70
80
90
100
110
120
130
140
150
160
170

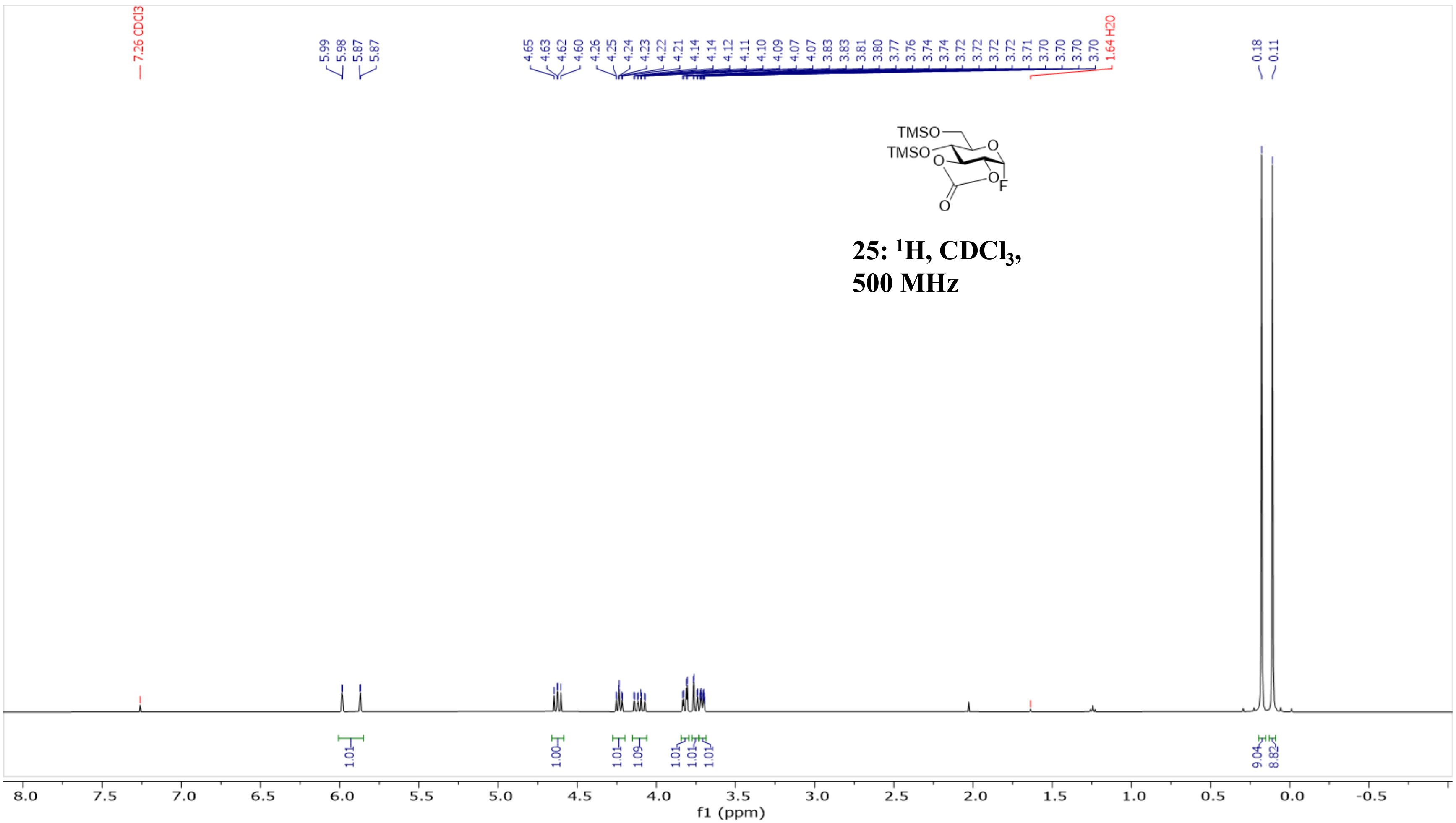
7.26 CDCl3
5.99
5.98
5.87
5.87
4.65
4.63
4.62
4.60
4.26
4.25
4.24
4.23
4.22
4.21
4.14
4.14
4.12
4.11
4.10
4.09
4.07
4.07
3.83
3.83
3.81
3.80
3.77
3.76
3.74
3.74
3.72
3.72
3.72
3.72
3.71
3.70
3.70
3.70
3.70
1.64 H2O
0.18
0.11
TMSO
TMSO
O
O
O
O
F
25: 1H, CDCl3,
500 MHz
1.01
1.00
1.01
1.09
1.01
1.01
1.01
9.04
8.82
8.0
7.5
7.0
6.5
6.0
5.5
5.0
4.5
4.0
3.5
3.0
2.5
2.0
1.5
1.0
0.5
0.0
-0.5
f1 (ppm)

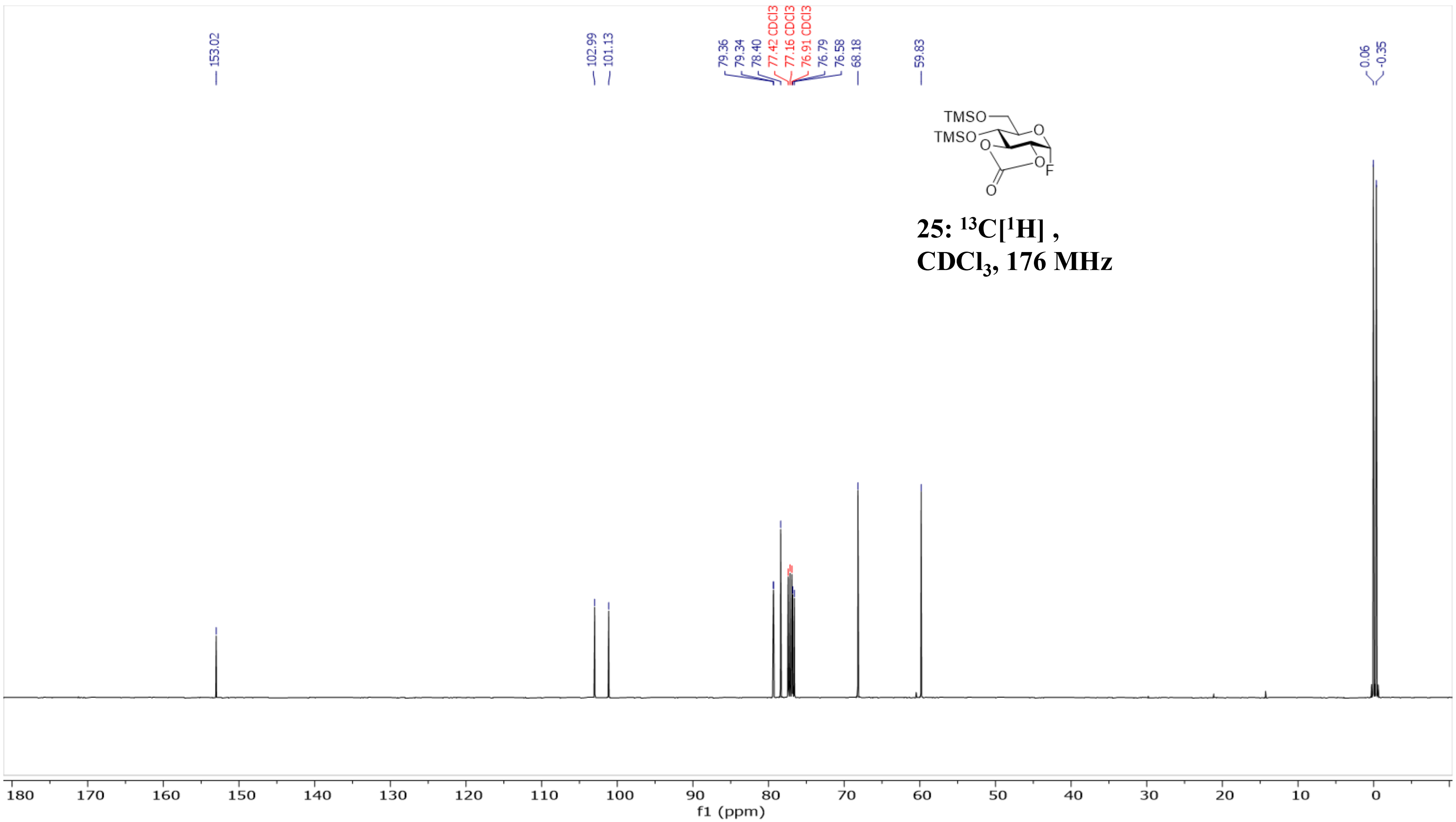

25: $^{13}C[^{1}H]$, $CDCl_3$, 176 MHz

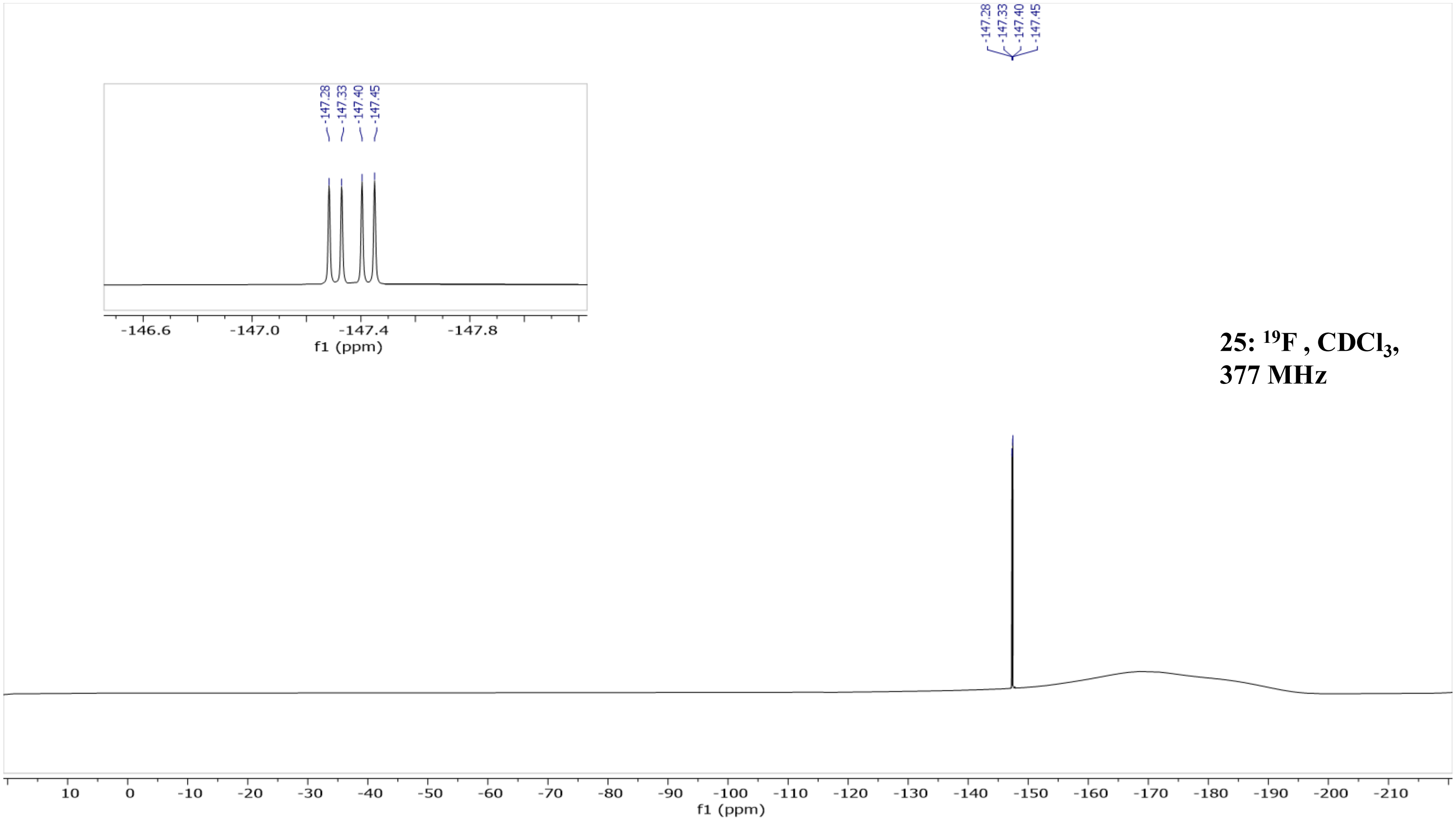

25: ^{19}F , $CDCl_3$, 377 MHz

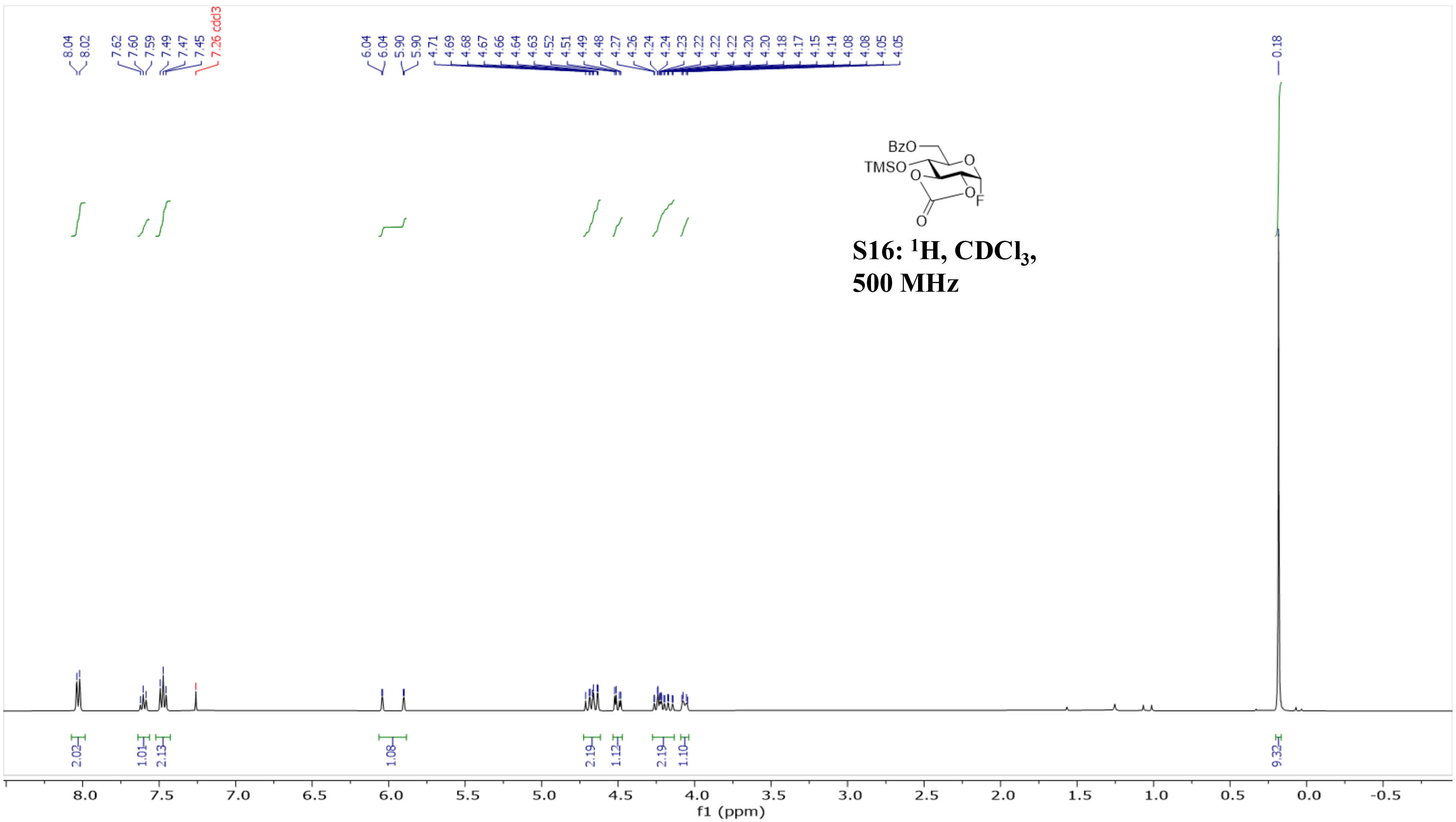

BzO
TMSO
O
O
O
F
O
S16: ^{1}H, CDCl$_3$, 500 MHz
f1 (ppm)

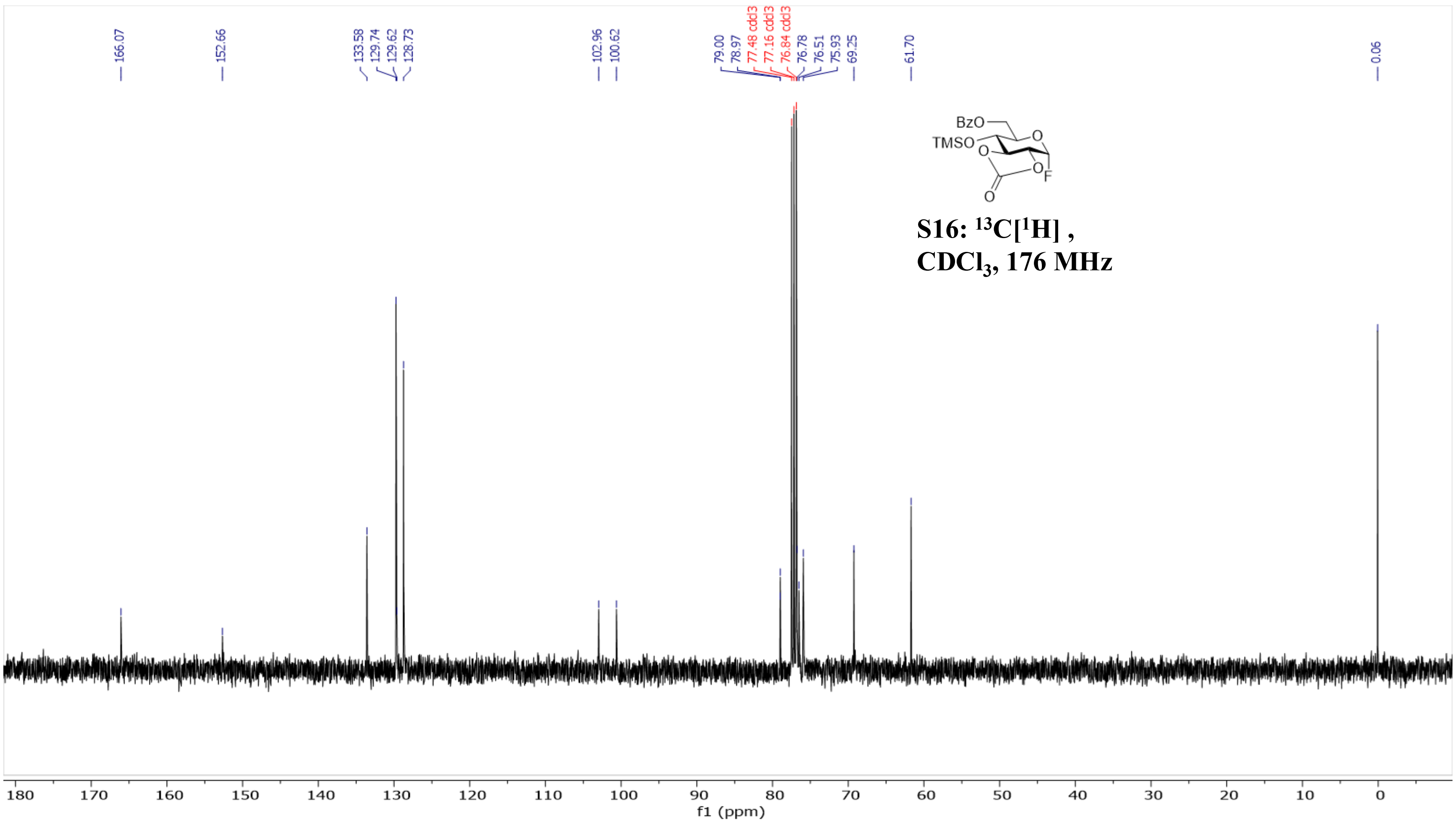
166.07
152.66
133.58
129.74
129.62
128.73
102.96
100.62
79.00
78.97
77.48 cdcl3
77.16 cdcl3
76.84 cdcl3
76.78
76.51
75.93
69.25
61.70
0.06
BzO
TMSO
O
O
O
O
F
S16: $^{13}C[^{1}H]$,
$CDCl_3$, 176 MHz
180
170
160
150
140
130
120
110
100
90
80
70
60
50
40
30
20
10
0
f1 (ppm)

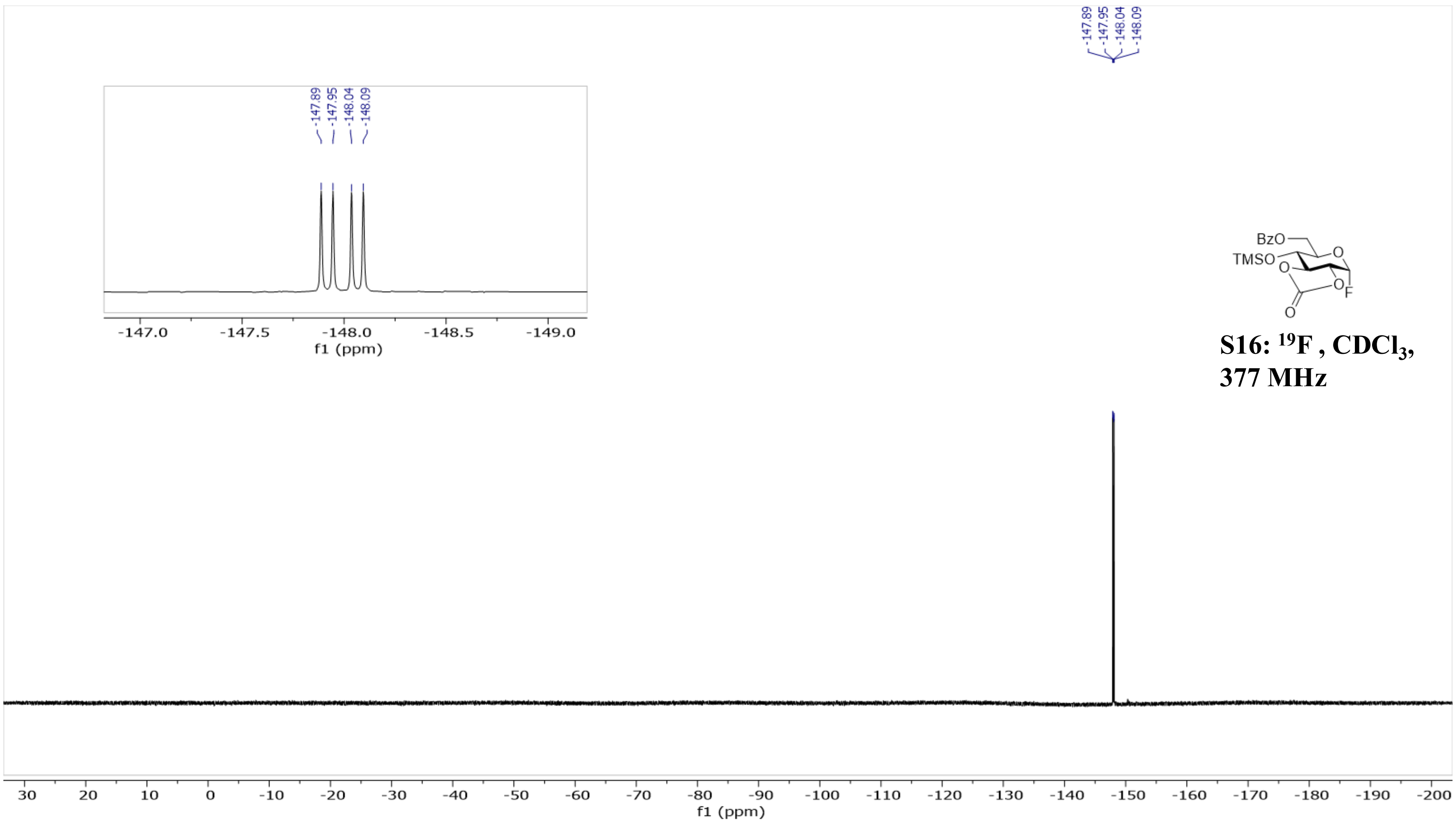

S16: ^{19}F , $CDCl_3$, 377 MHz

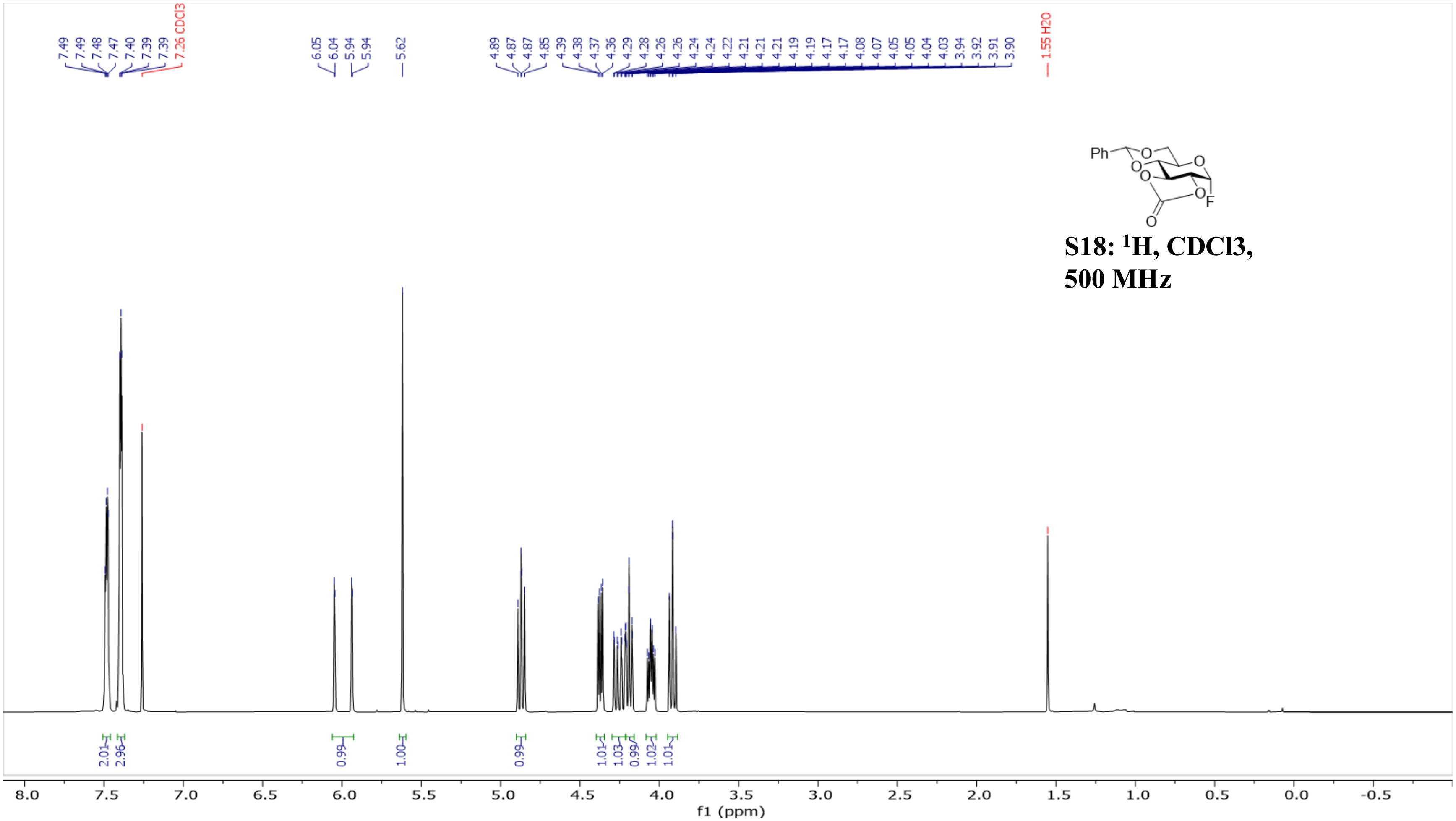

S18: ^{1}H, CDCl3, 500 MHz

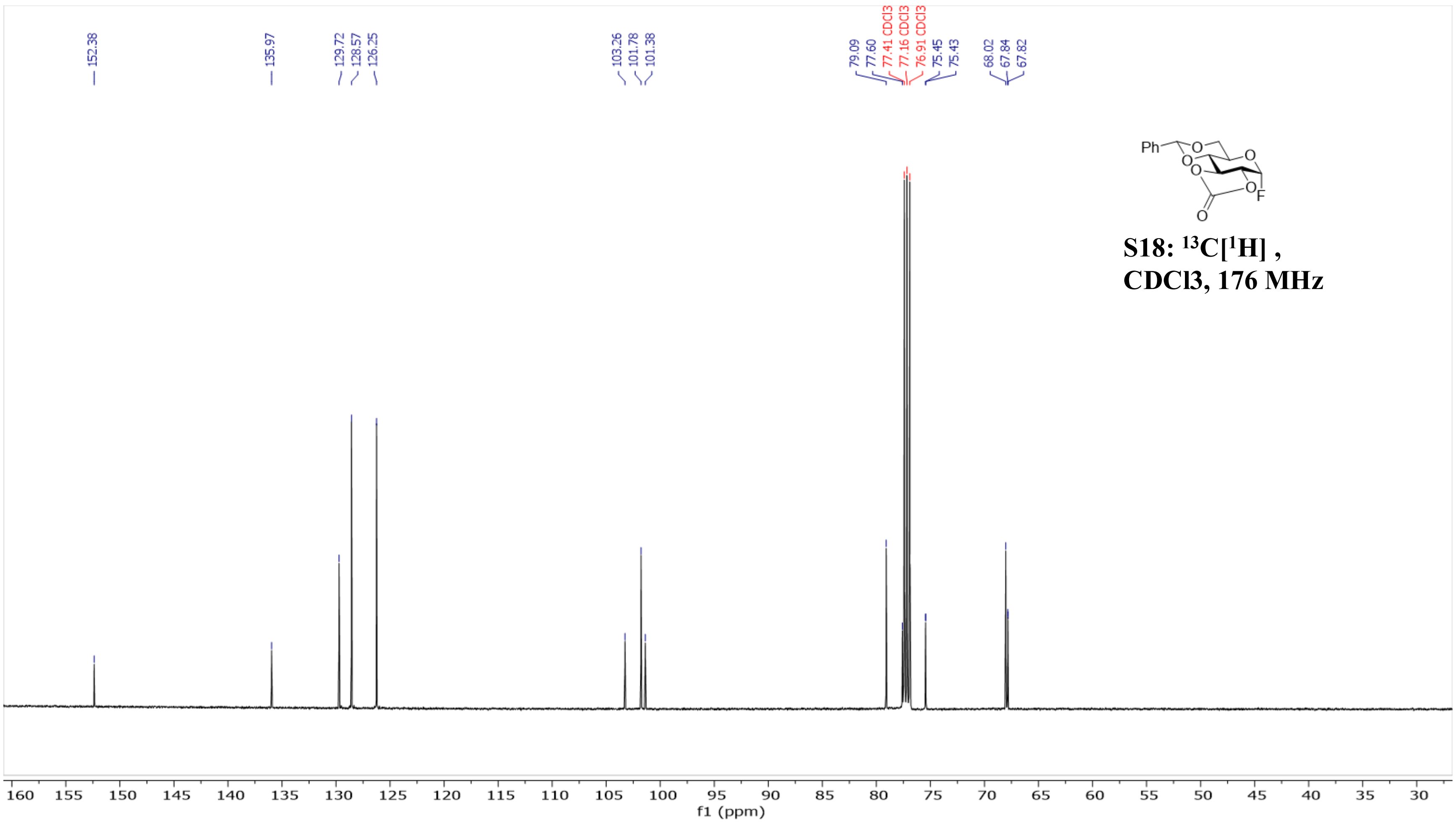

S18: ^{13}C[^{1}H] , CDCl3, 176 MHz

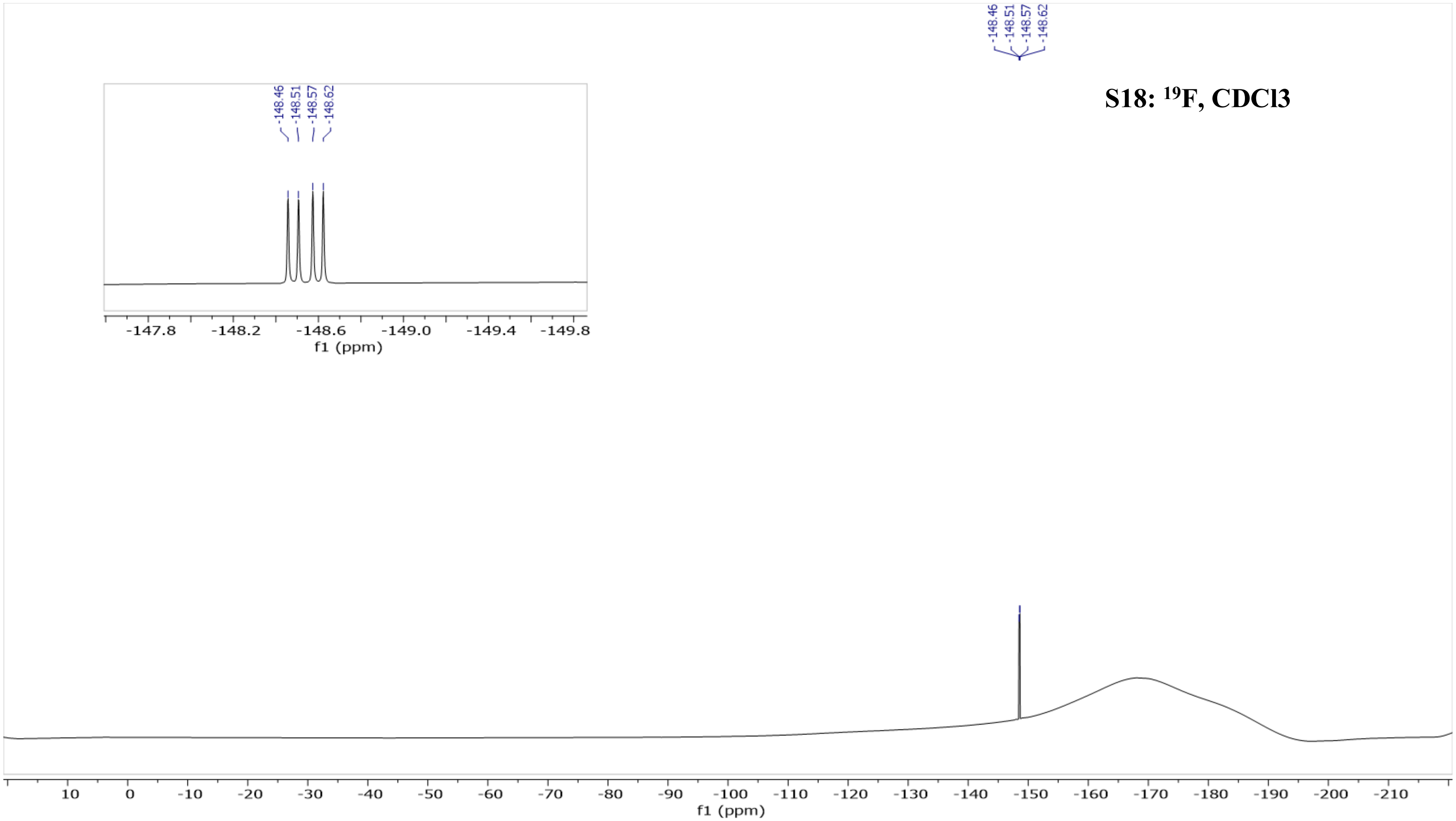
-148.46
-148.51
-148.57
-148.62
S18: 19F, CDCl3
-147.8
-148.2
-148.6
-149.0
-149.4
-149.8
f1 (ppm)
10
0
-10
-20
-30
-40
-50
-60
-70
-80
-90
-100
-110
-120
-130
-140
-150
-160
-170
-180
-190
-200
-210
f1 (ppm)

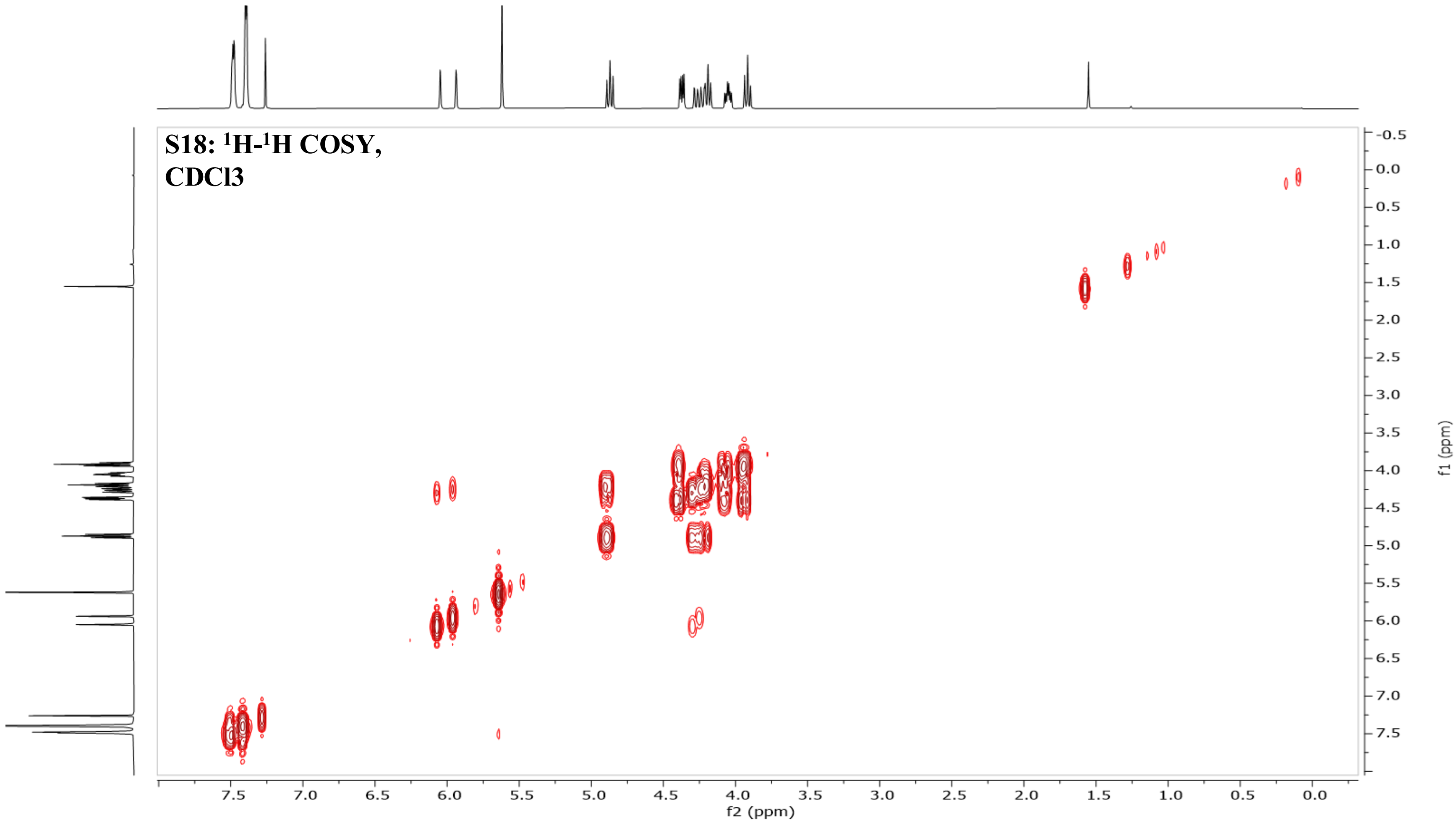

S18: 1H-1H COSY,
CDCl3
f2 (ppm)
f1 (ppm)

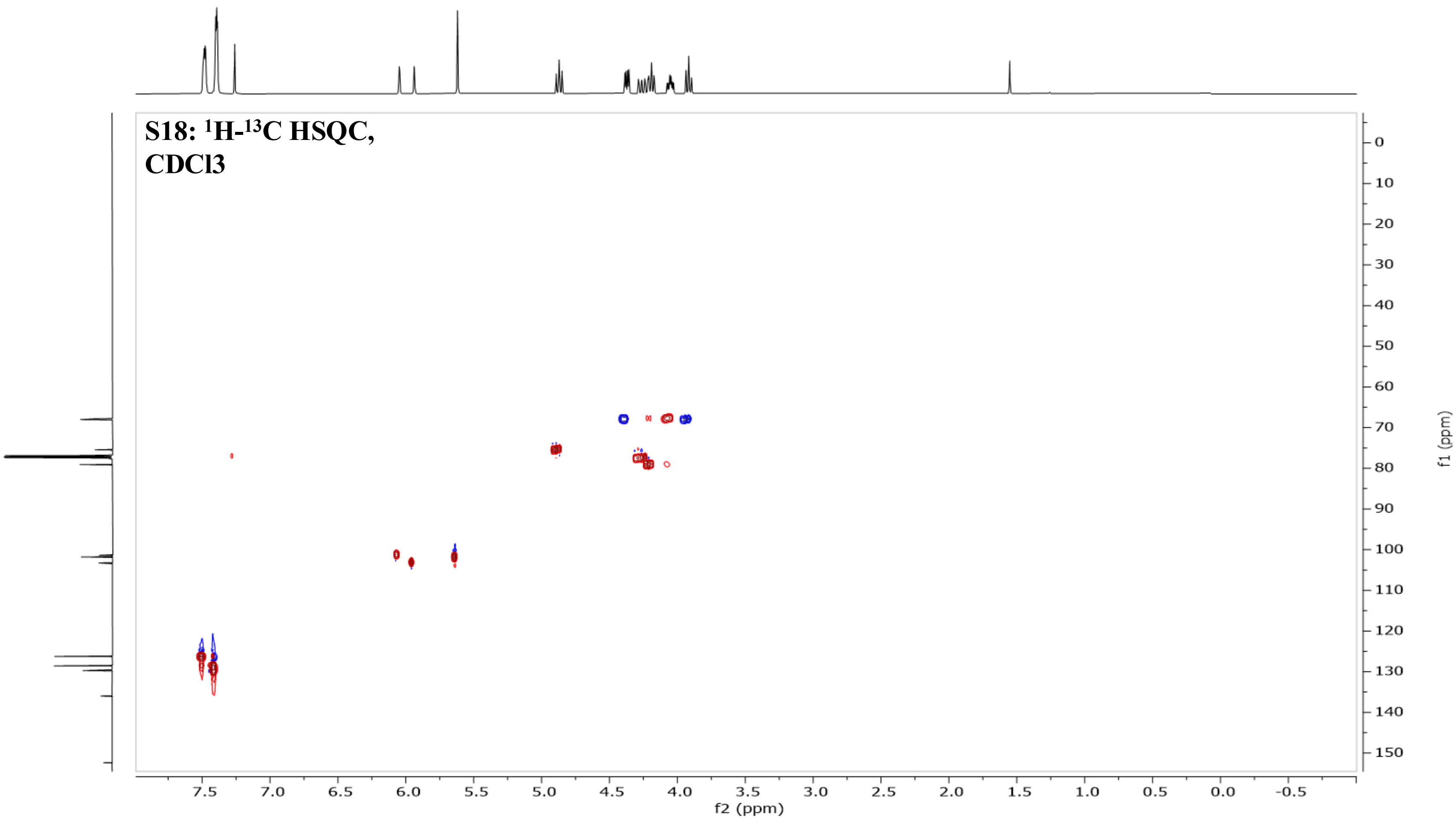
S18: ¹H-¹³C HSQC,
CDCl3
f2 (ppm)
f1 (ppm)

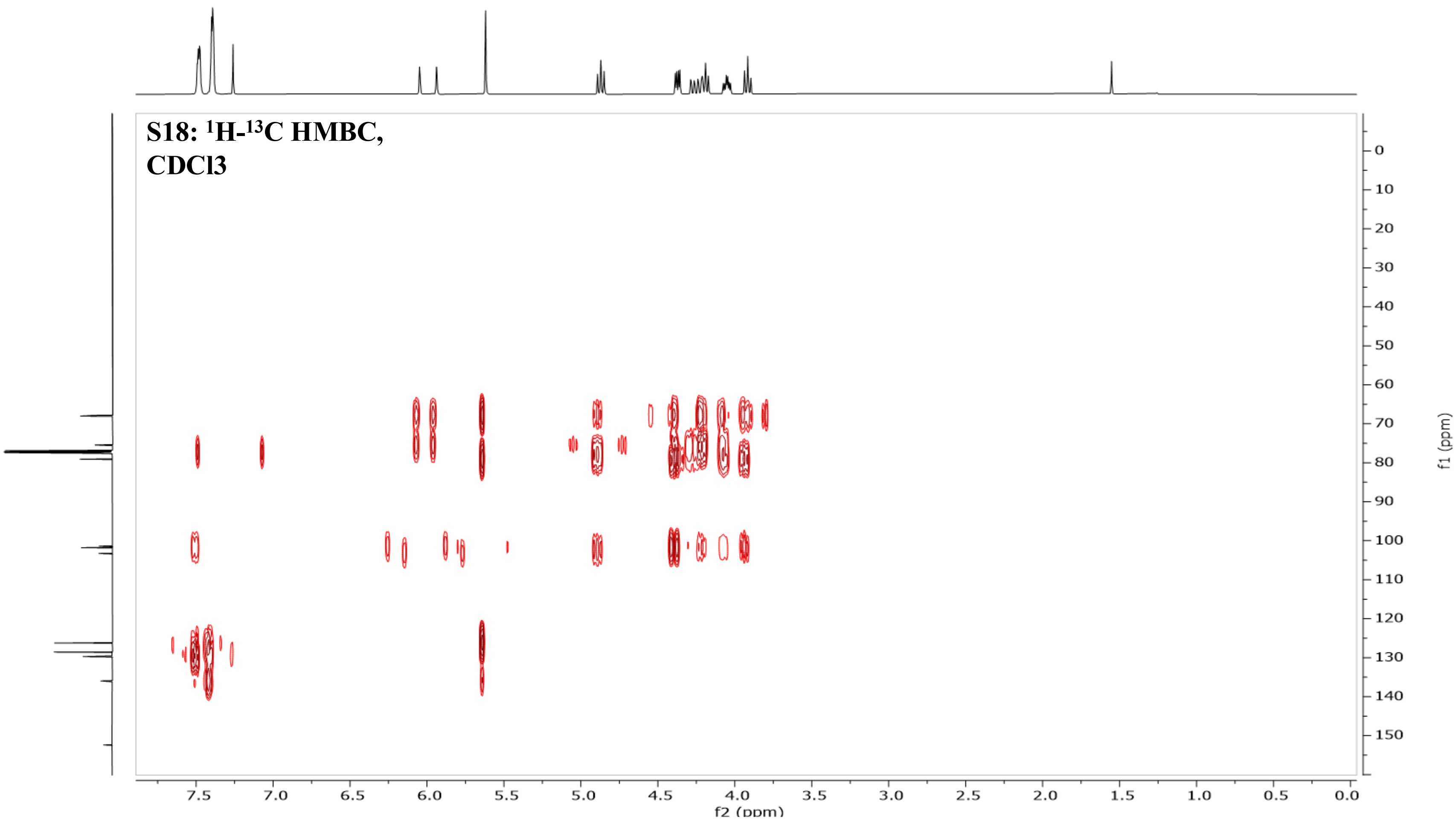
S18: 1H-13C HMBC,
CDCl3
f2 (ppm)
f1 (ppm)
7.5 7.0 6.5 6.0 5.5 5.0 4.5 4.0 3.5 3.0 2.5 2.0 1.5 1.0 0.5 0.0
0 10 20 30 40 50 60 70 80 90 100 110 120 130 140 150

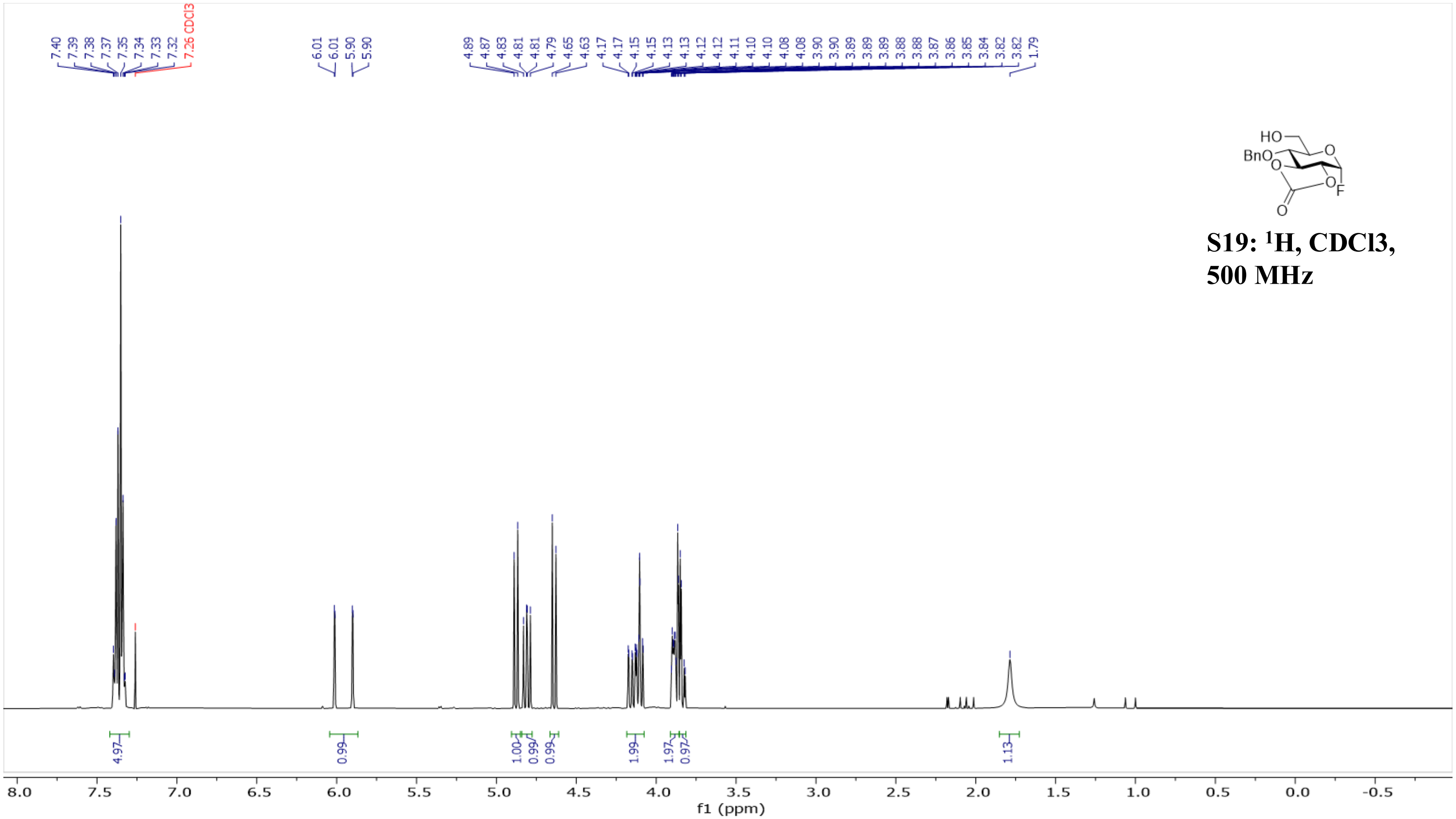

S19: ^{1}H, CDCl3, 500 MHz

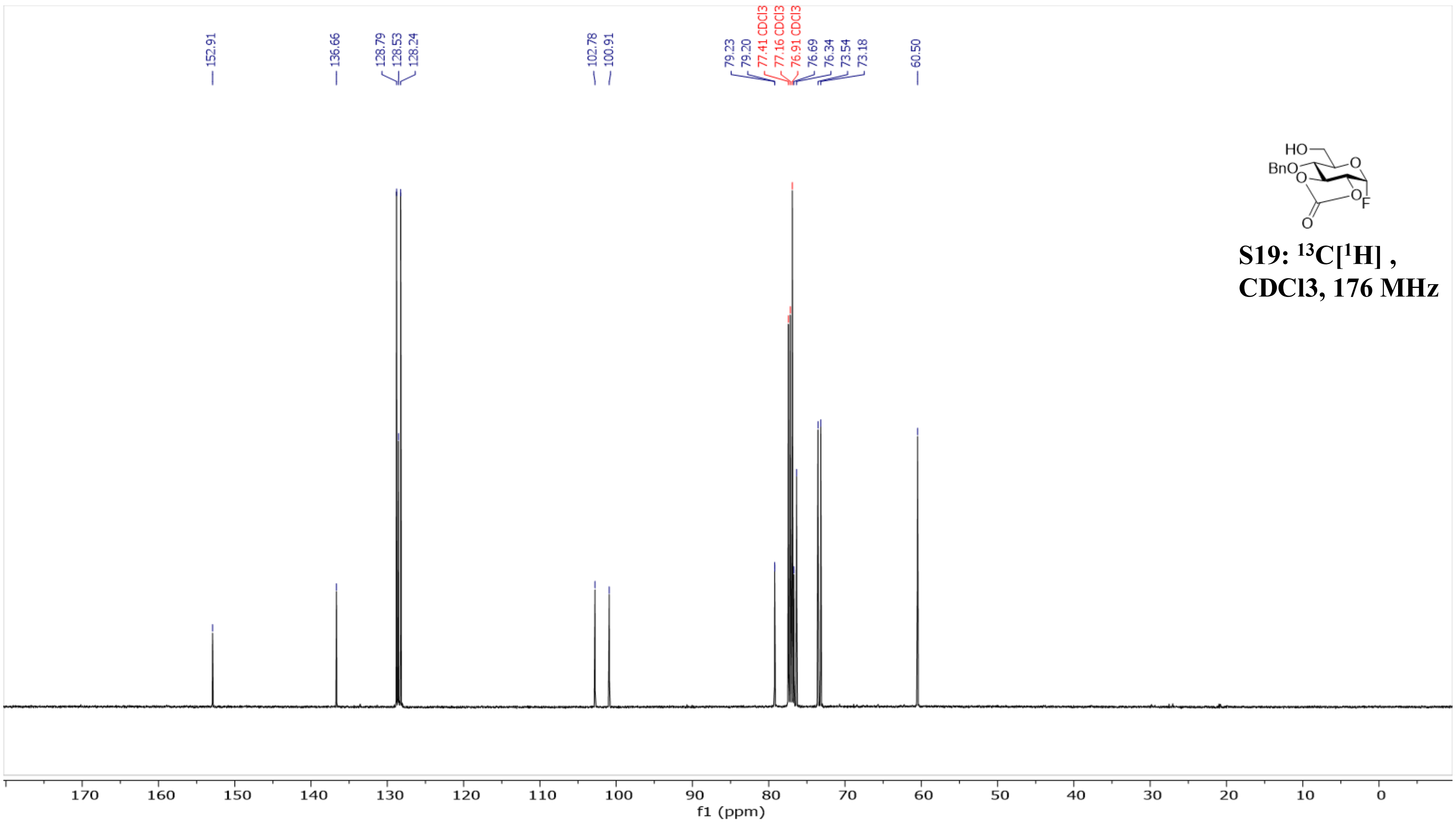

S19: ^{13}C[^{1}H] , CDCl3, 176 MHz

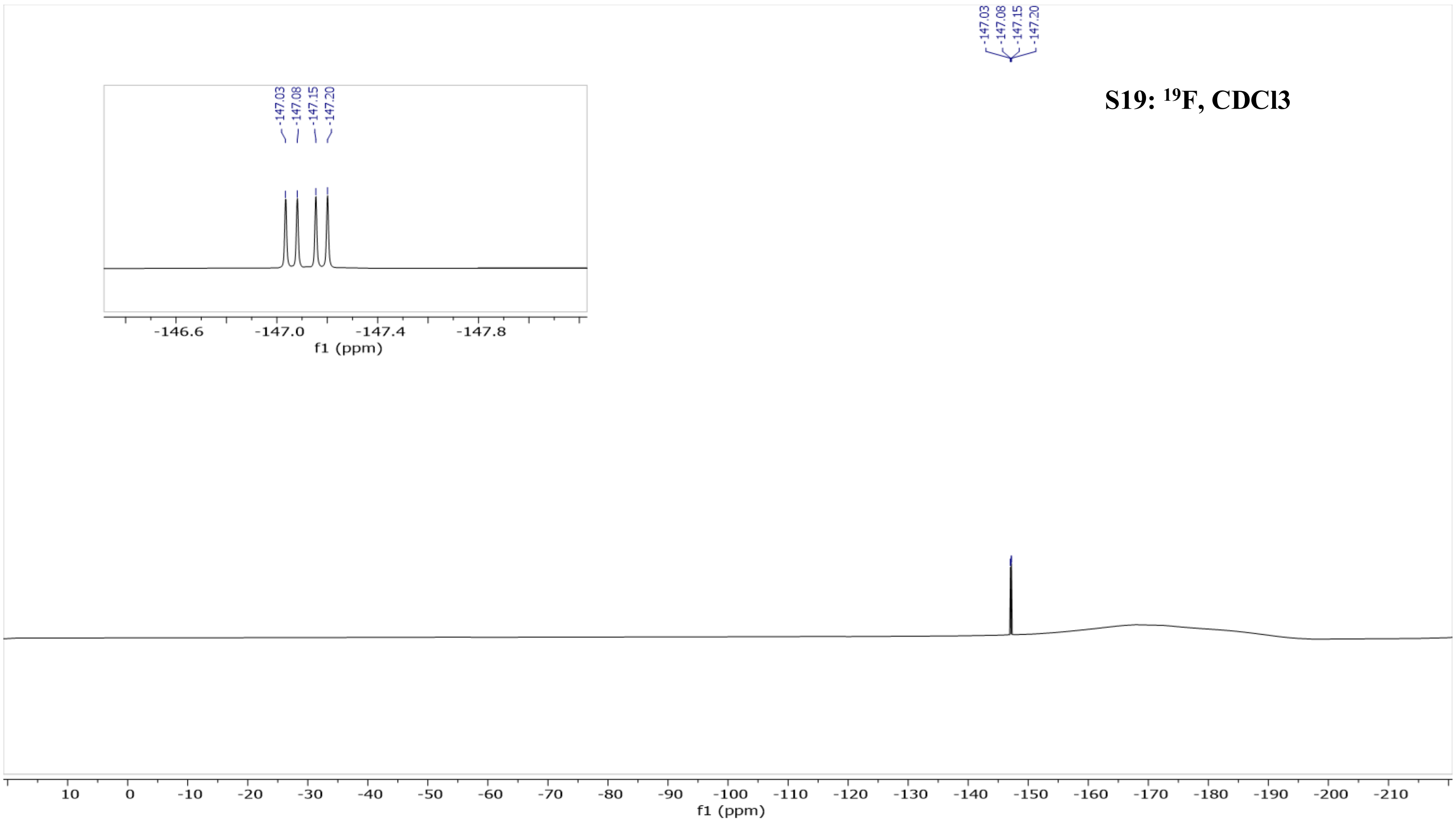
-147.03
-147.08
-147.15
-147.20
S19: ¹⁹F, CDCl3
-146.6
-147.0
-147.4
-147.8
f1 (ppm)
10
0
-10
-20
-30
-40
-50
-60
-70
-80
-90
-100
-110
-120
-130
-140
-150
-160
-170
-180
-190
-200
-210
f1 (ppm)

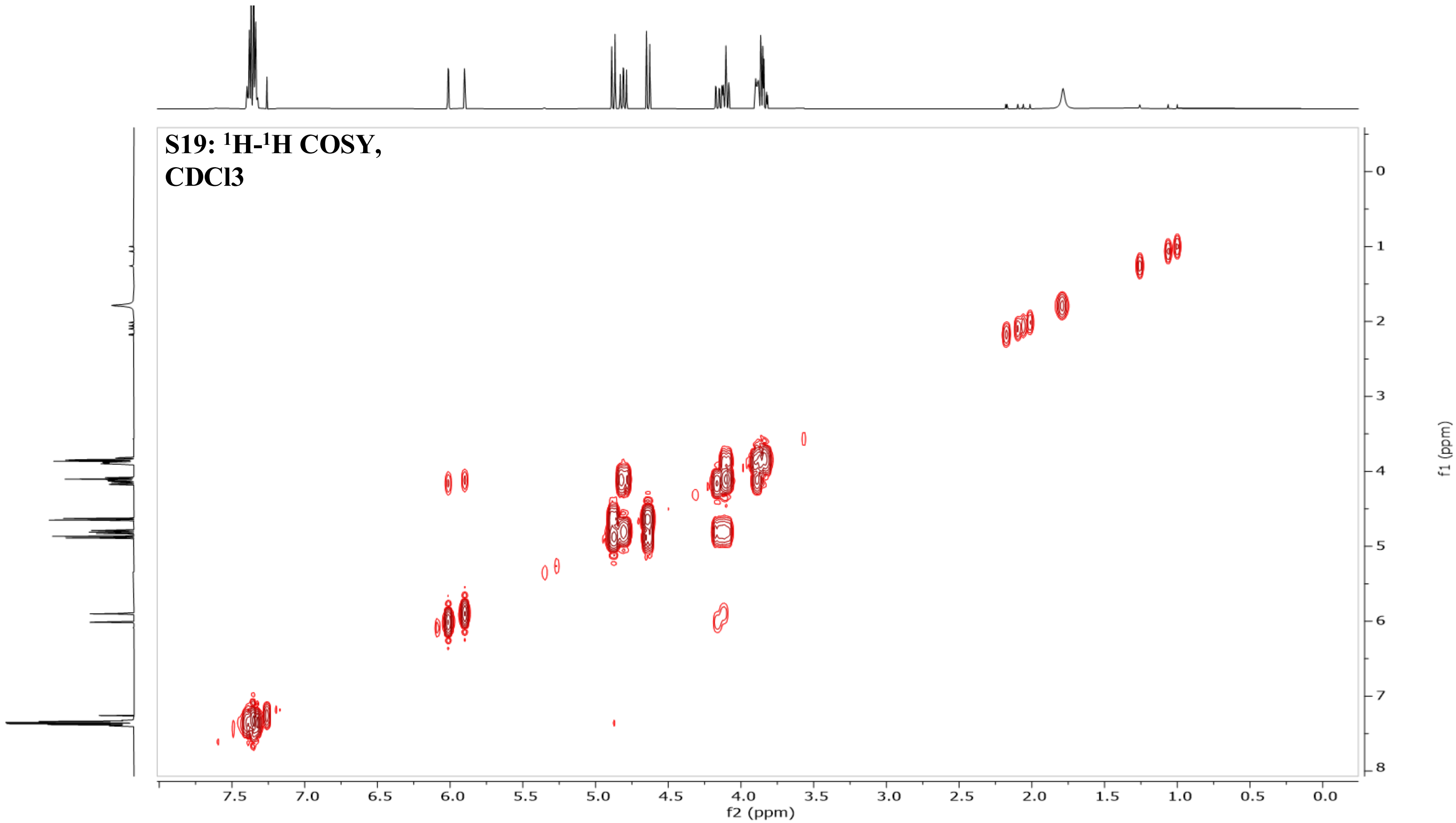
S19: ^{1}H-^{1}H COSY,
CDCl3
f2 (ppm)
f1 (ppm)
7.5
7.0
6.5
6.0
5.5
5.0
4.5
4.0
3.5
3.0
2.5
2.0
1.5
1.0
0.5
0.0
0
1
2
3
4
5
6
7
8

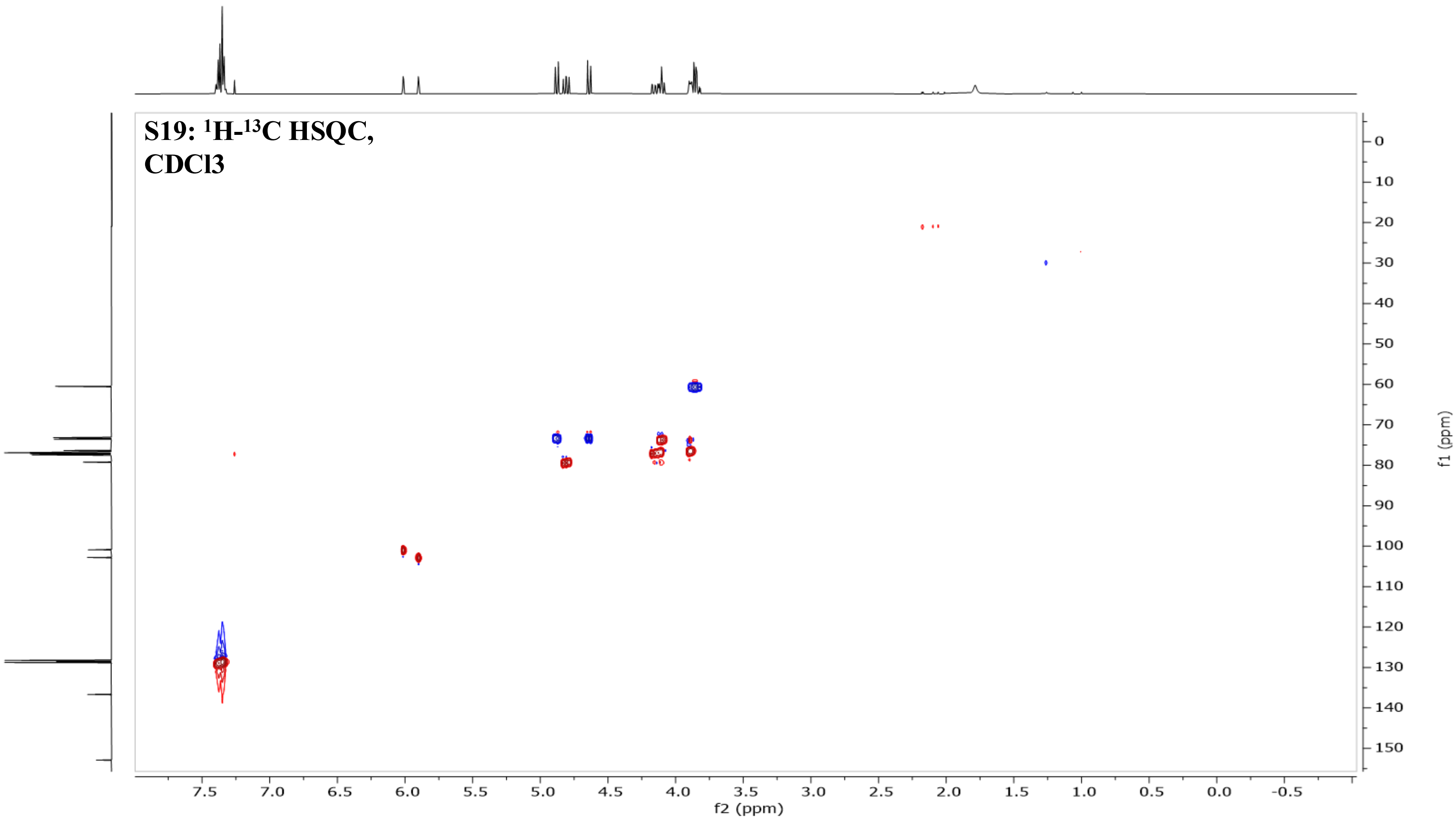
S19: 1H-13C HSQC,
CDCl3
f2 (ppm)
f1 (ppm)

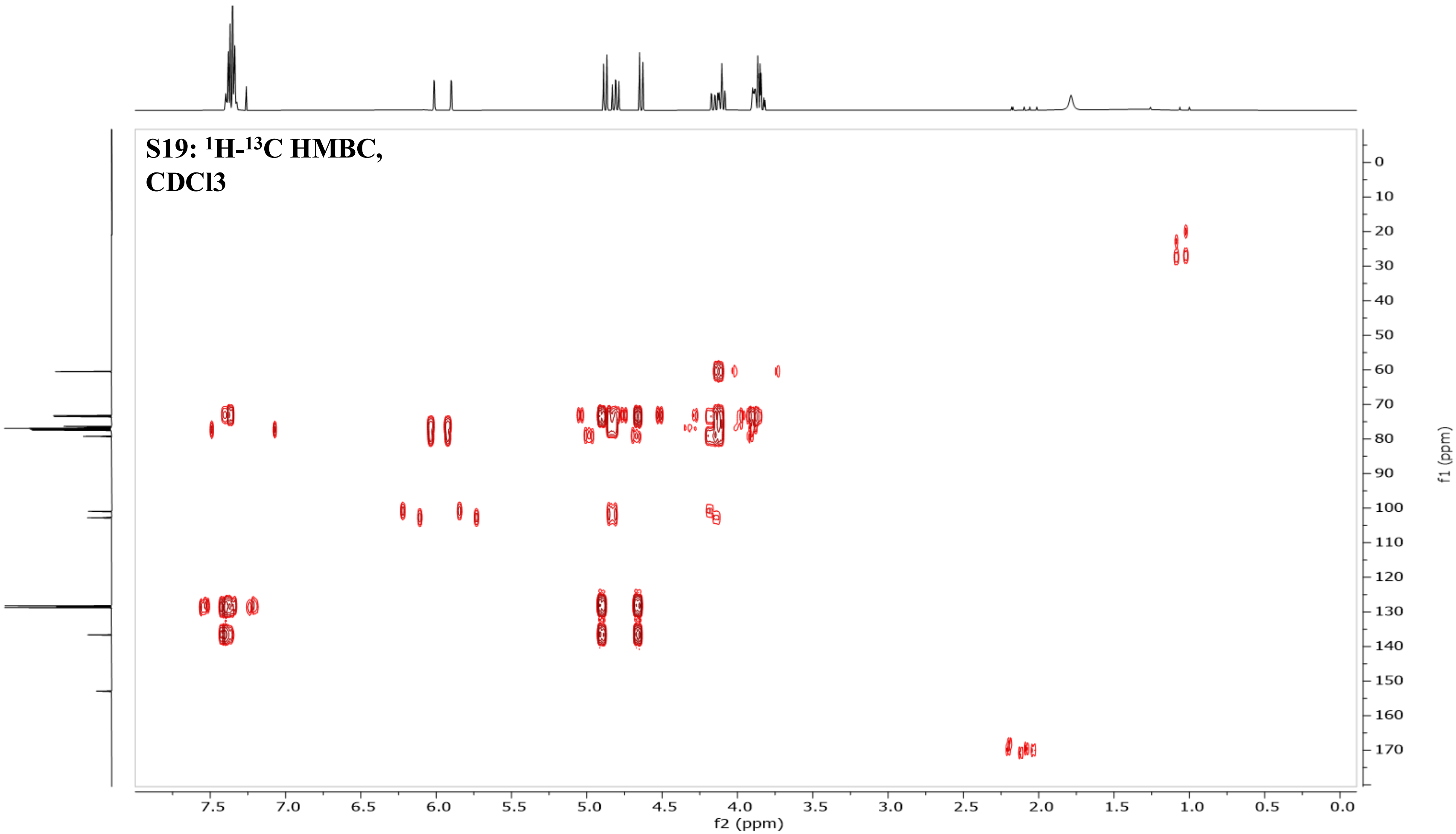
S19: 1H-13C HMBC,
CDCl3
f2 (ppm)
f1 (ppm)

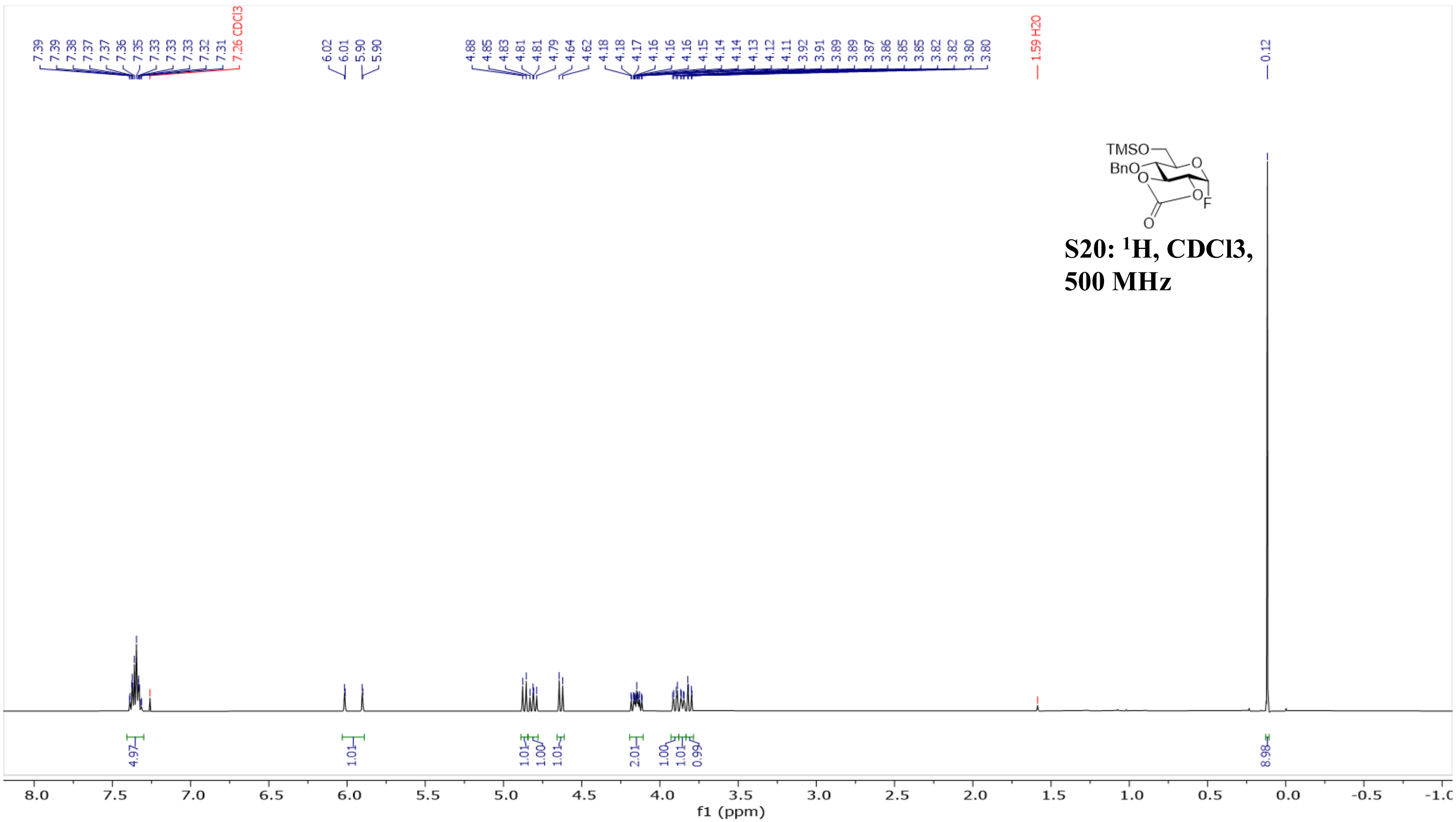
S20: [1]H, CDCl3,
500 MHz
f1 (ppm)

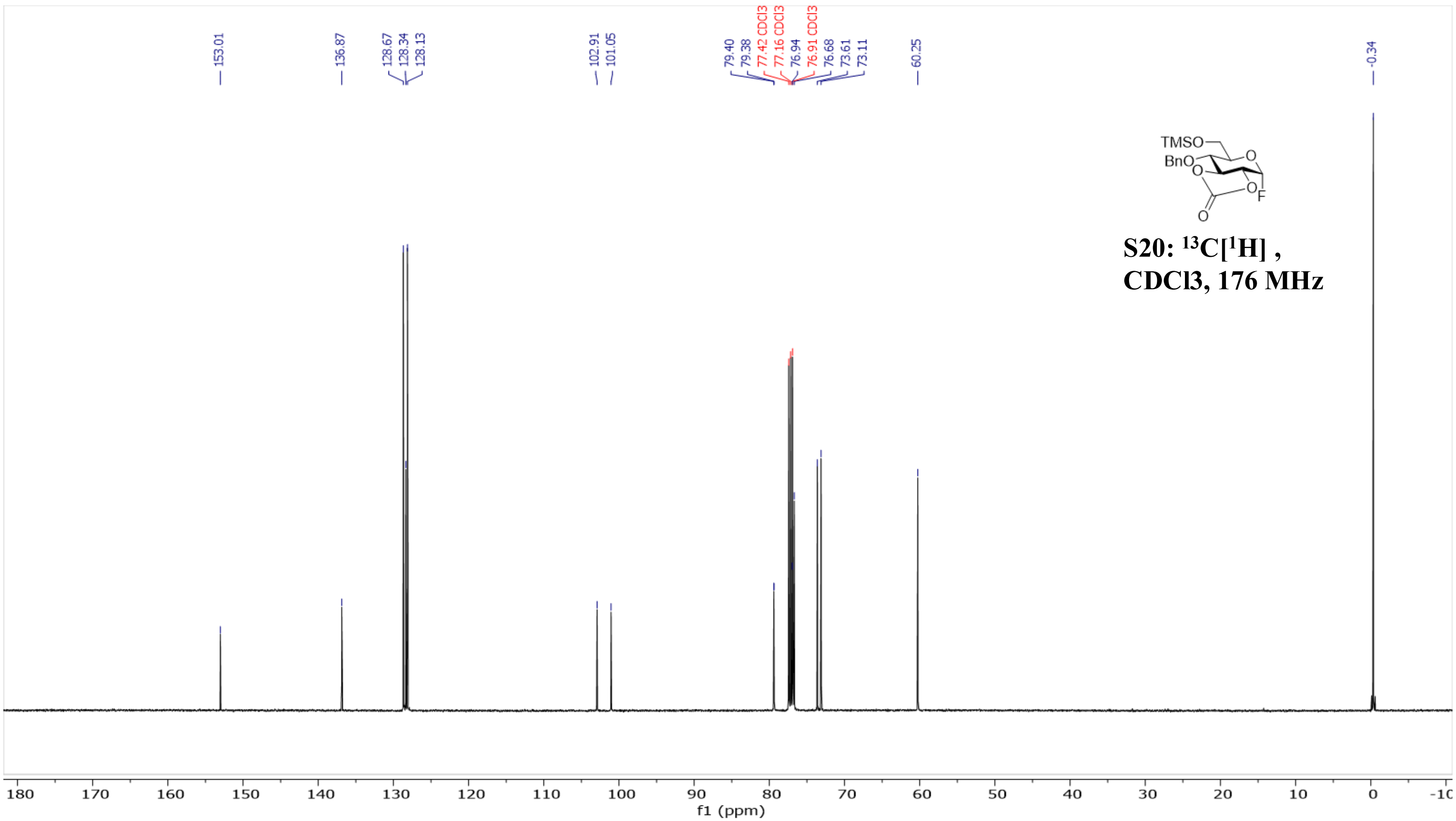
153.01
136.87
128.67
128.34
128.13
102.91
101.05
79.40
79.38
77.42 CDCl3
77.16 CDCl3
76.94
76.91 CDCl3
76.68
73.61
73.11
60.25
-0.34
TMSO
BnO
O
O
O
F
O
S20: 13C[1H] ,
CDCl3, 176 MHz
180 170 160 150 140 130 120 110 100 90 80 70 60 50 40 30 20 10 0 -10
f1 (ppm)

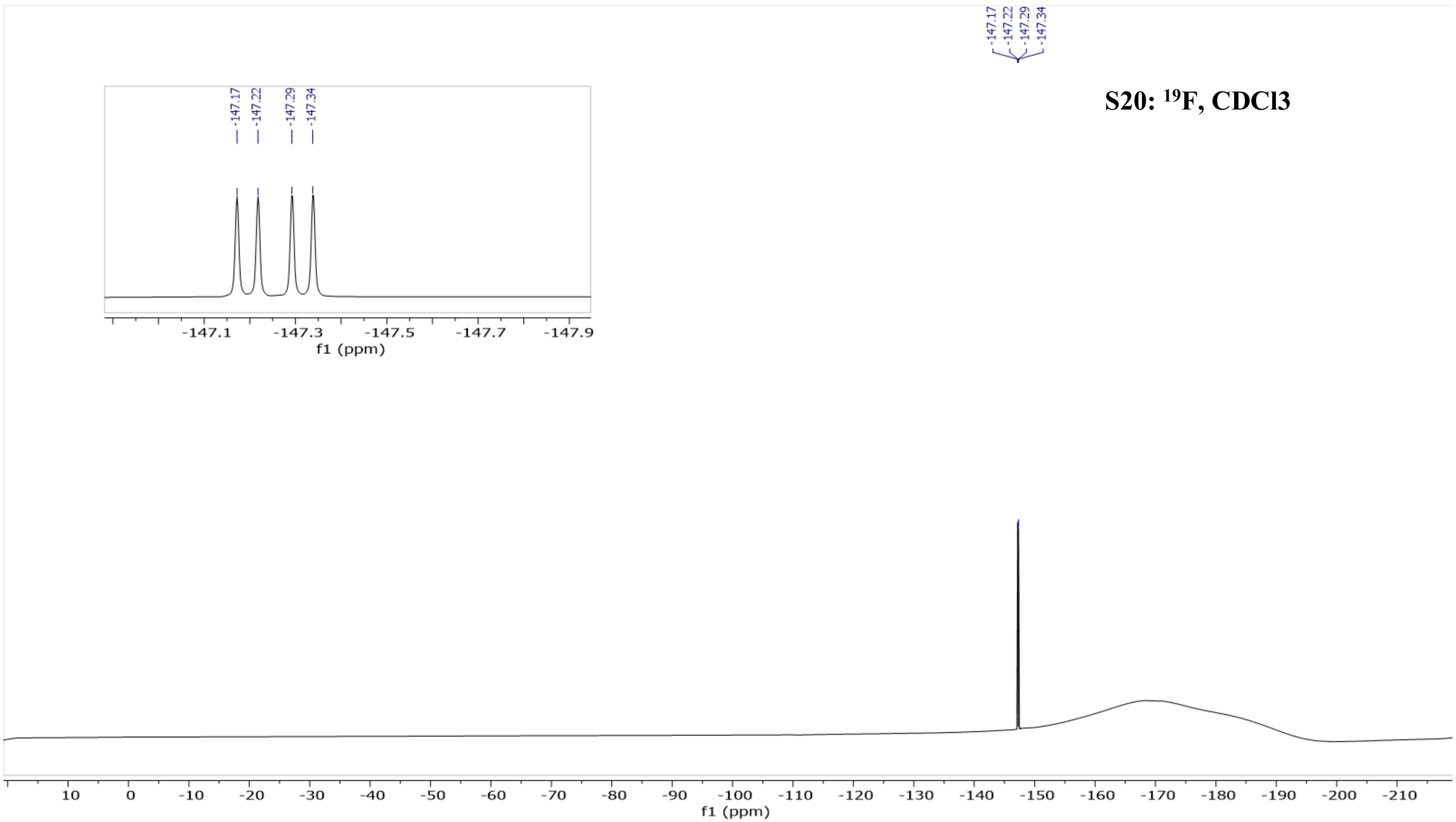
S20: ^{19}F, CDCl3
-147.17
-147.22
-147.29
-147.34
-147.1
-147.3
-147.5
-147.7
-147.9
f1 (ppm)
10 0 -10 -20 -30 -40 -50 -60 -70 -80 -90 -100 -110 -120 -130 -140 -150 -160 -170 -180 -190 -200 -210
f1 (ppm)

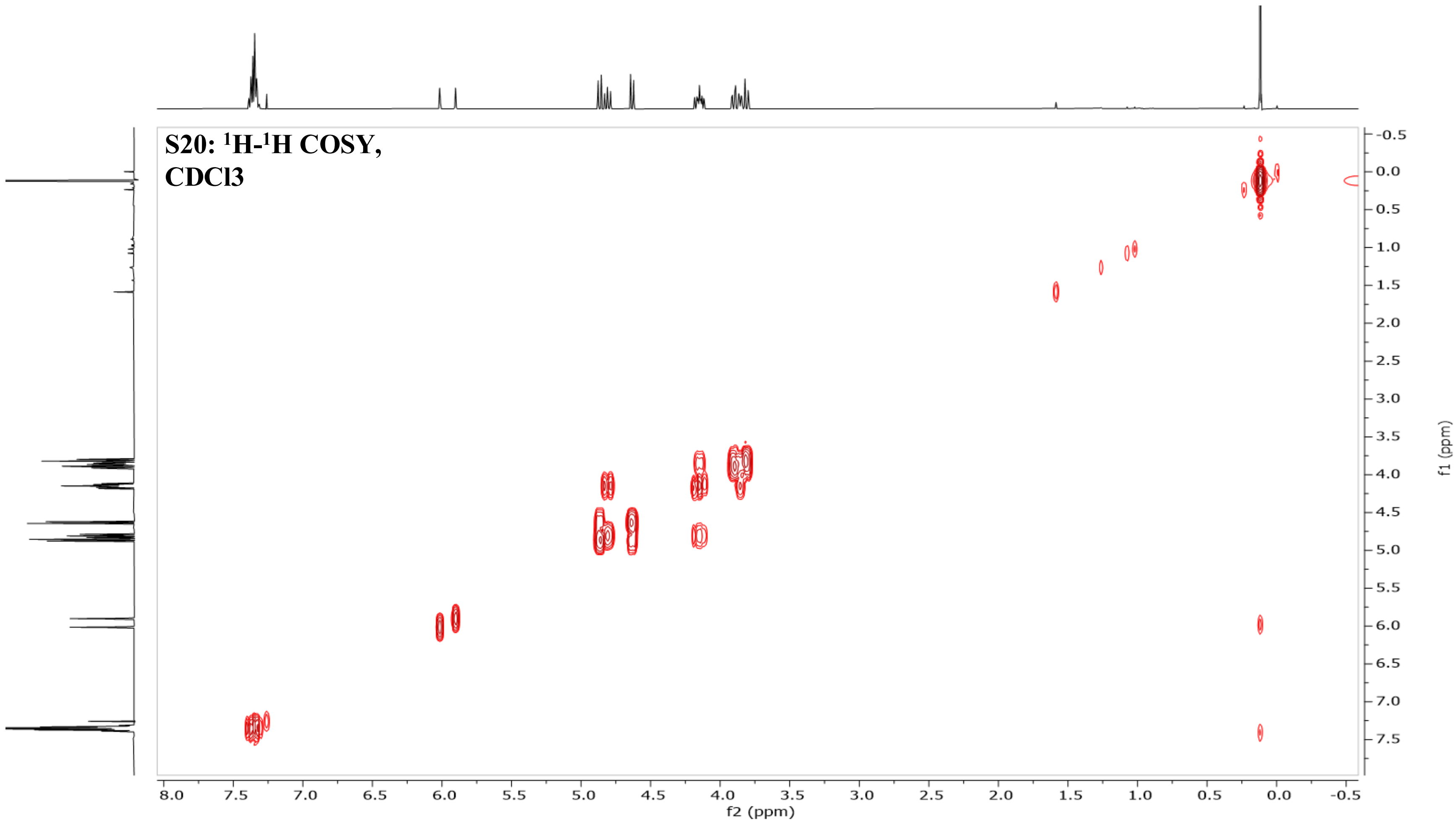
S20: ^{1}H-^{1}H COSY,
CDCl3
f2 (ppm)
f1 (ppm)

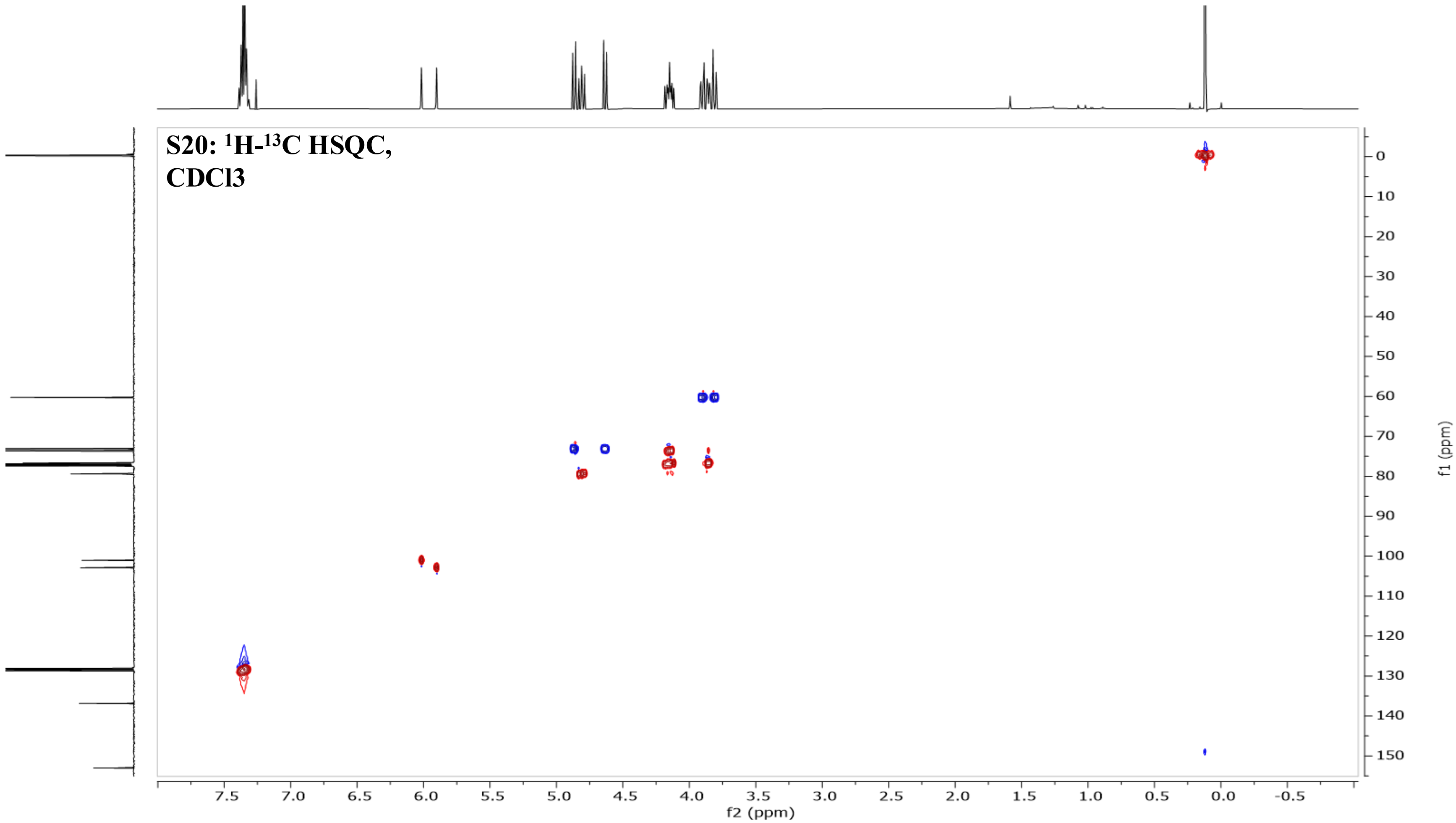
S20: ^{1}H-^{13}C HSQC,
CDCl3
f2 (ppm)
f1 (ppm)

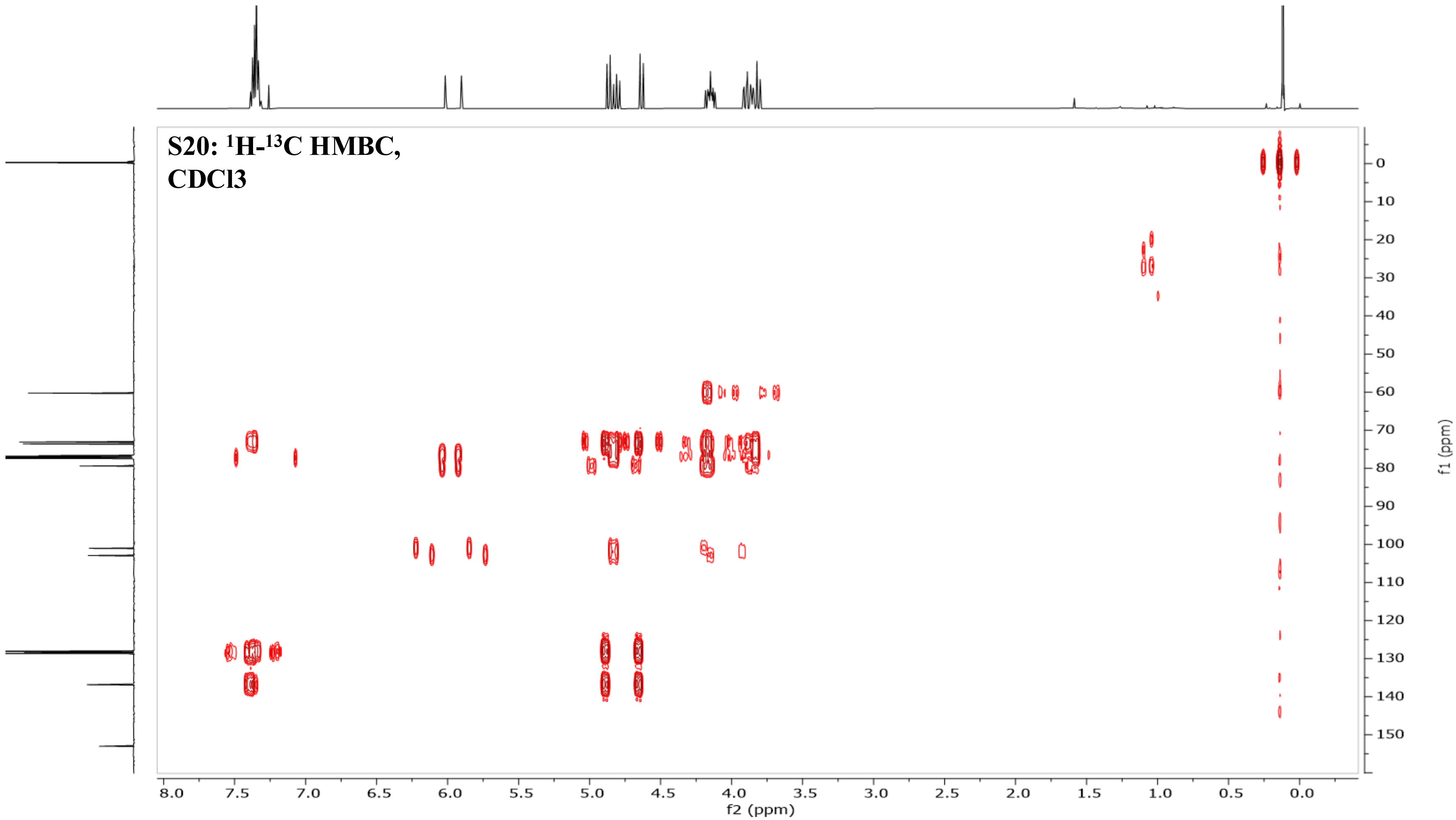
S20: ^{1}H-^{13}C HMBC,
CDCl3
f2 (ppm)
f1 (ppm)

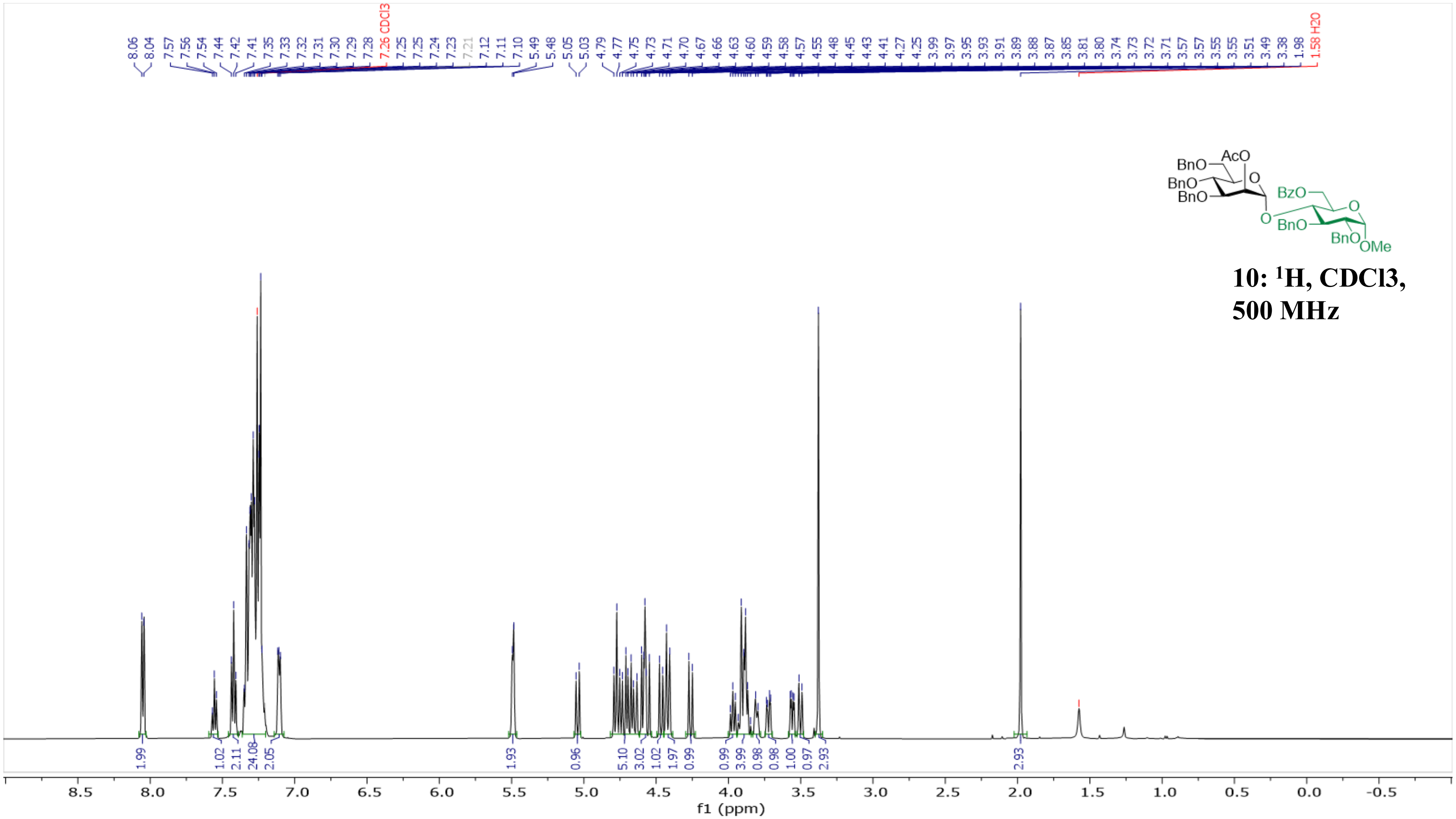

10: ¹H, CDCl3, 500 MHz

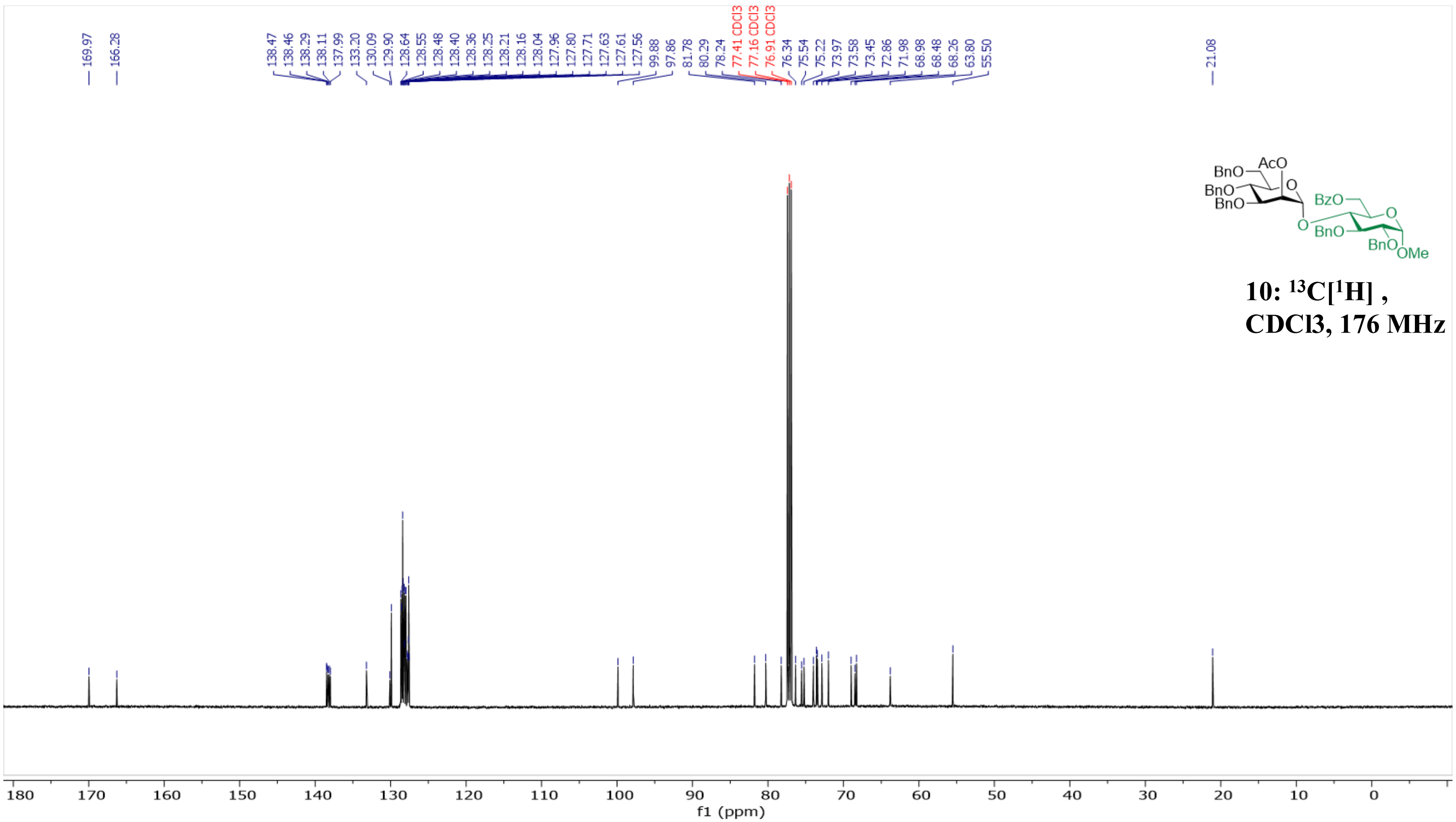
169.97
166.28
138.47
138.46
138.29
138.11
137.99
133.20
130.09
129.90
128.64
128.55
128.48
128.40
128.36
128.25
128.21
128.16
128.04
127.96
127.80
127.71
127.63
127.61
127.56
99.88
97.86
81.78
80.29
78.24
77.41 CDCl3
77.16 CDCl3
76.91 CDCl3
76.34
75.54
75.22
73.97
73.58
73.45
72.86
71.98
68.98
68.48
68.26
63.80
55.50
21.08
BnO
AcO
BnO
BnO
O
O
BzO
O
BnO
BnO
OMe
10: 13C[1H] ,
CDCl3, 176 MHz
180
170
160
150
140
130
120
110
100
90
80
70
60
50
40
30
20
10
0
f1 (ppm)

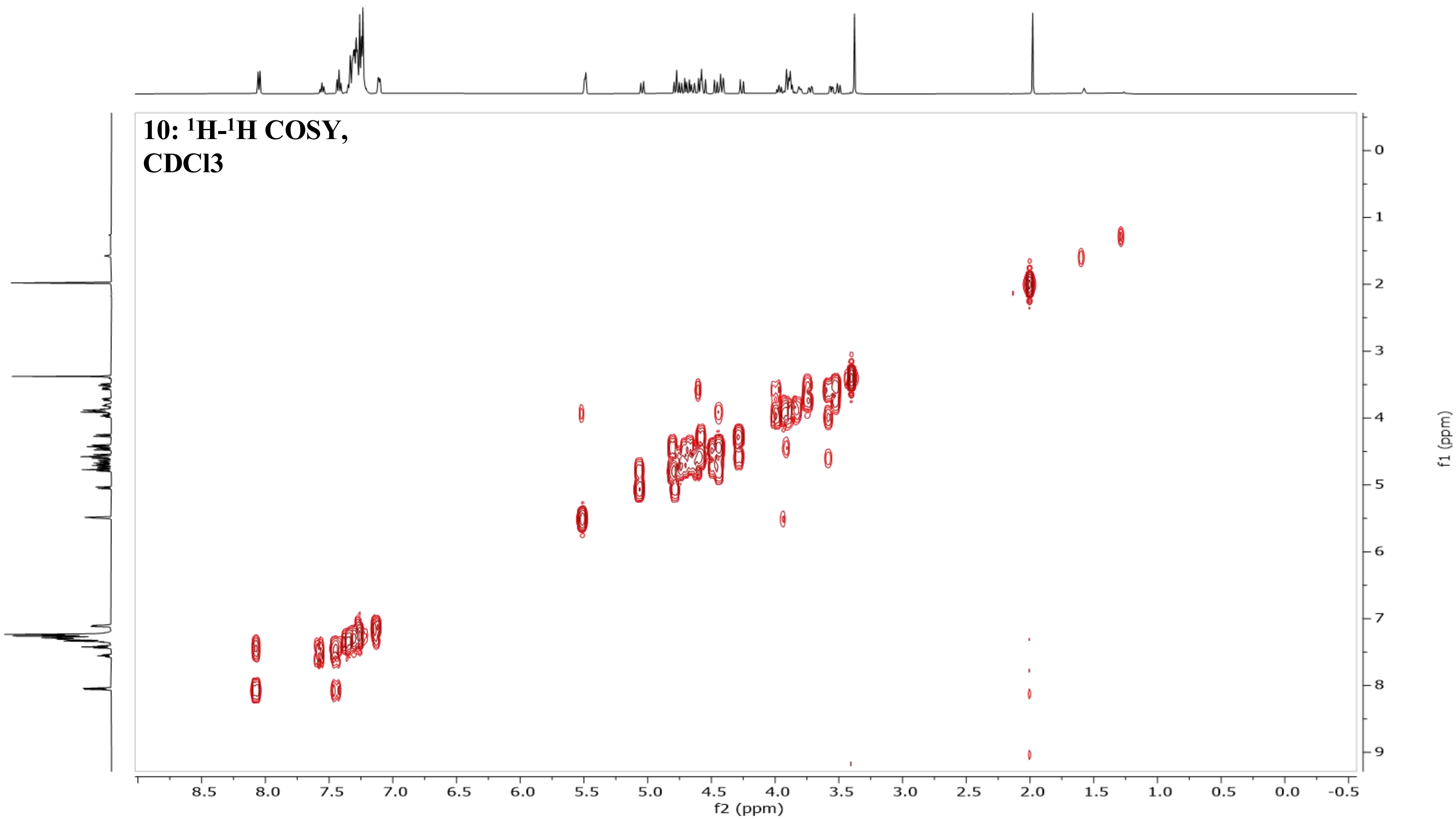
10: ¹H-¹H COSY,
CDCl3
f2 (ppm)
f1 (ppm)

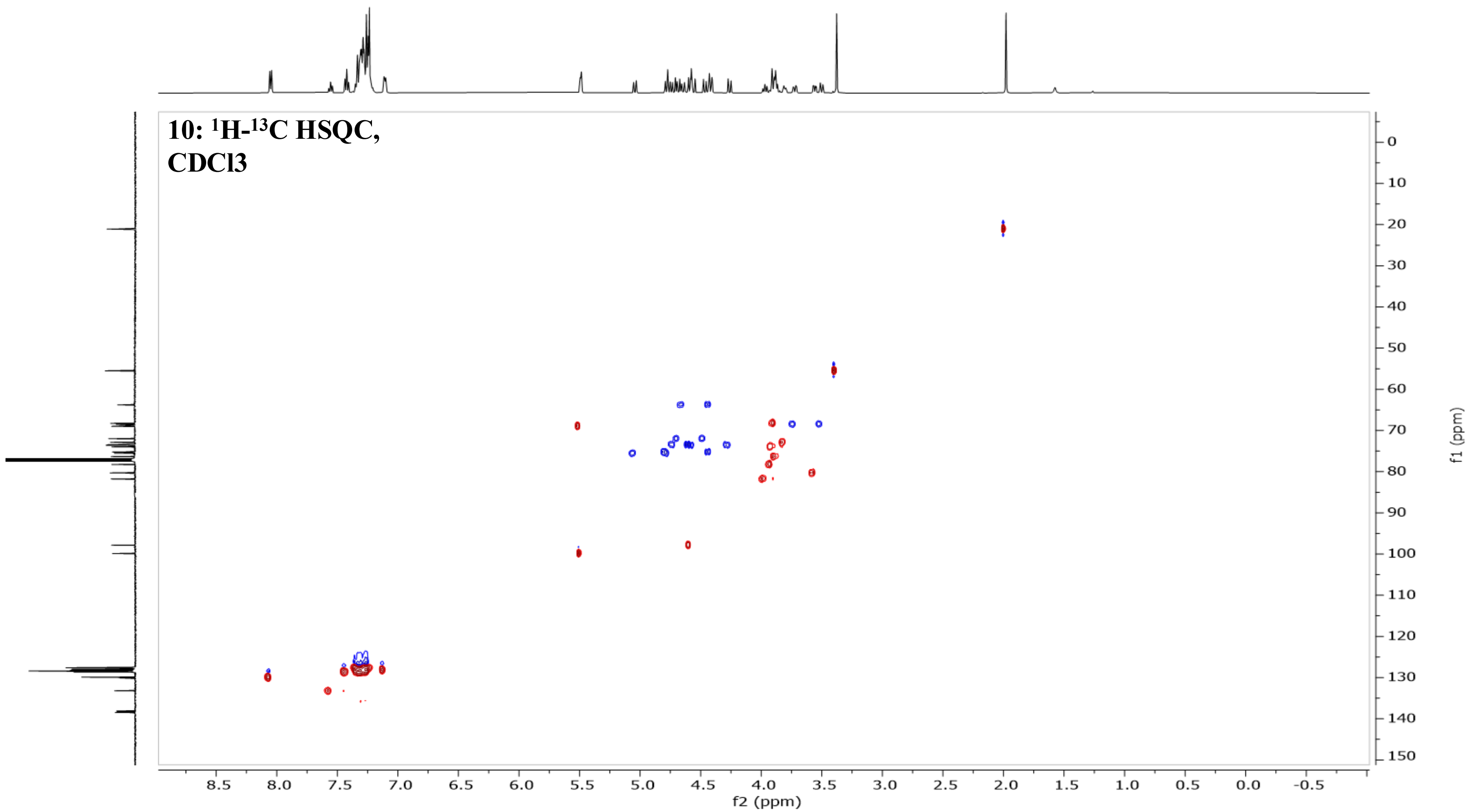

10: ¹H-¹³C HSQC,
CDCl3
f2 (ppm)
f1 (ppm)

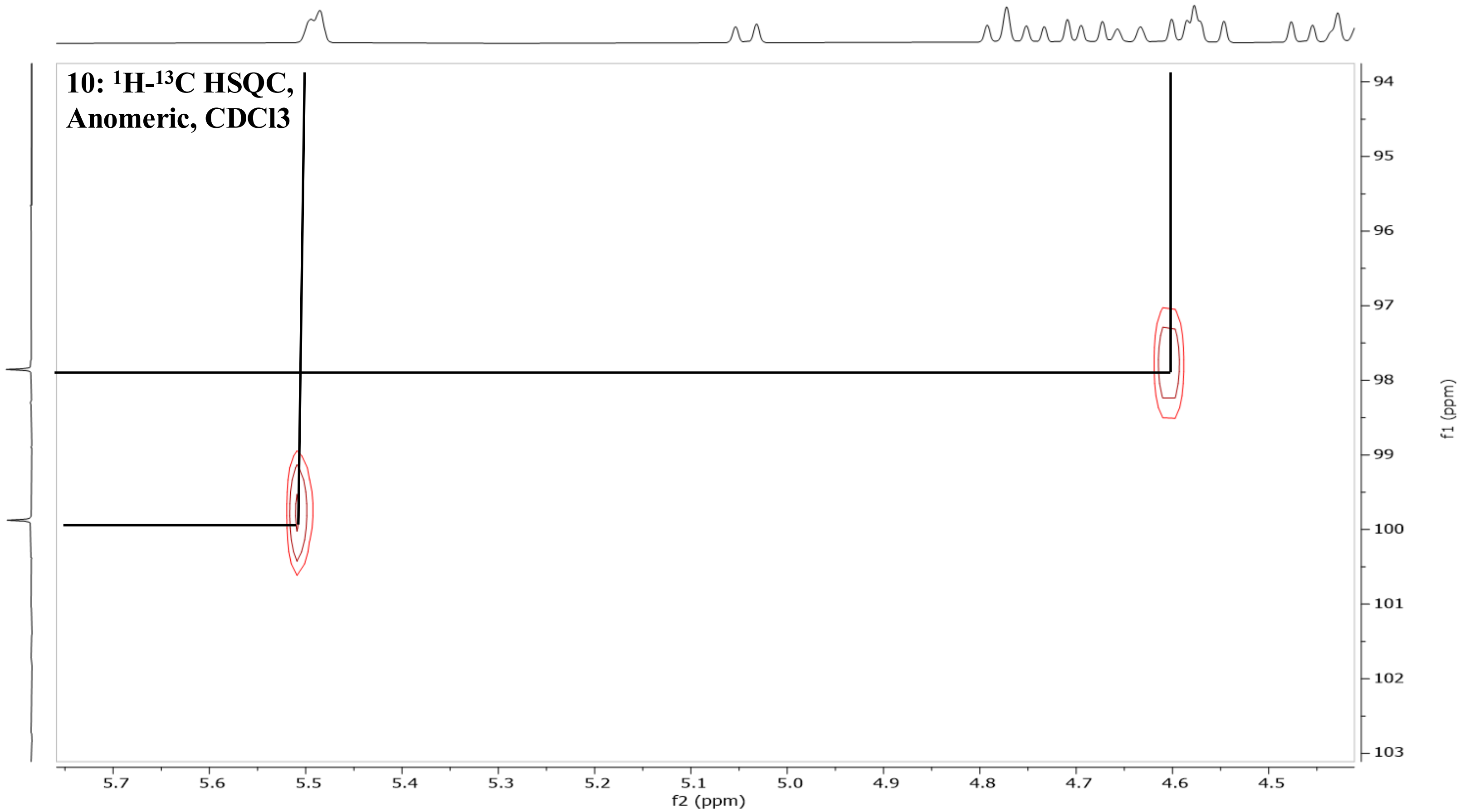
10: 1H-13C HSQC,
Anomeric, CDCl3
5.7
5.6
5.5
5.4
5.3
5.2
5.1
5.0
4.9
4.8
4.7
4.6
4.5
f2 (ppm)
94
95
96
97
98
99
100
101
102
103
f1 (ppm)

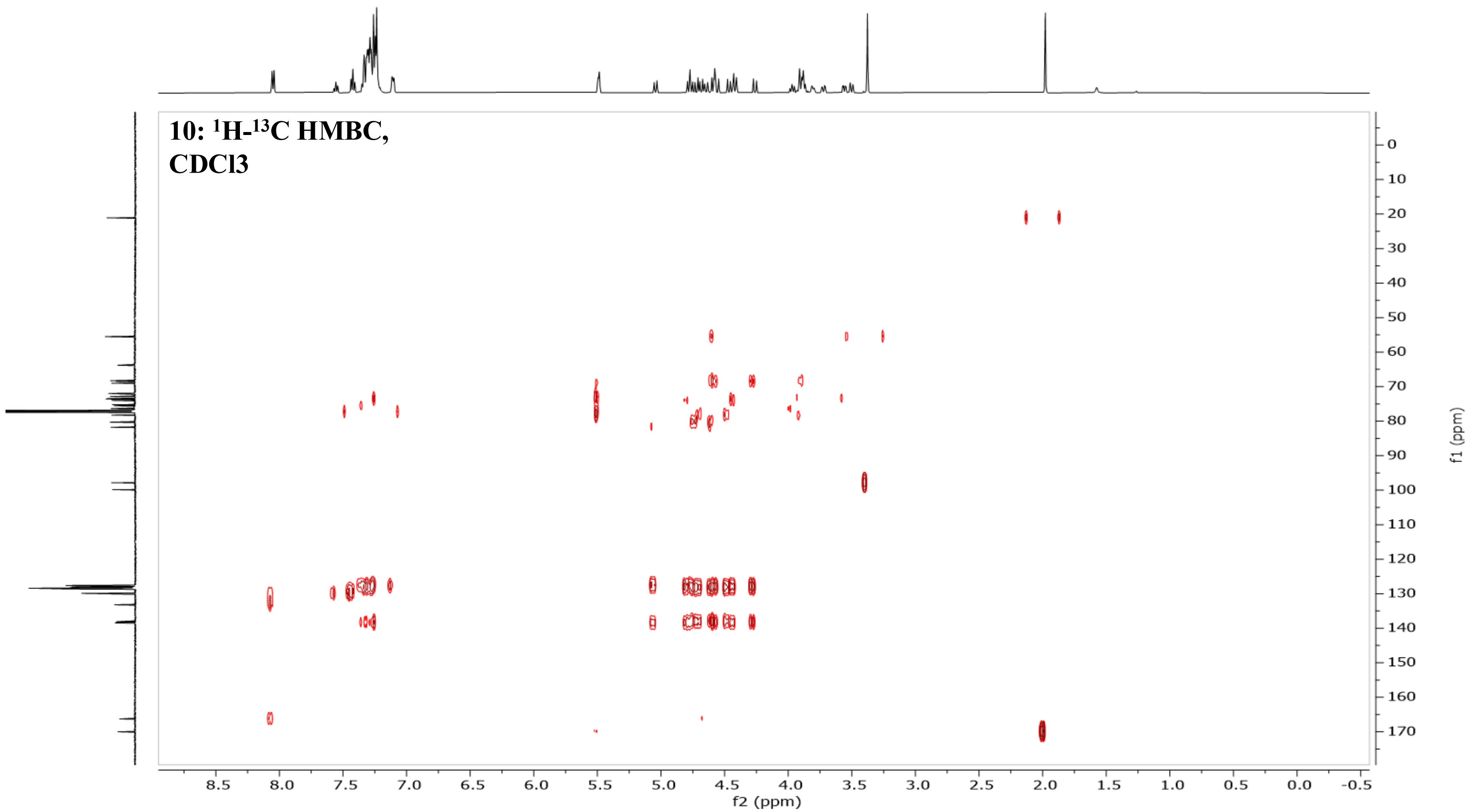
10: ¹H-¹³C HMBC,
CDCl3
f2 (ppm)
f1 (ppm)

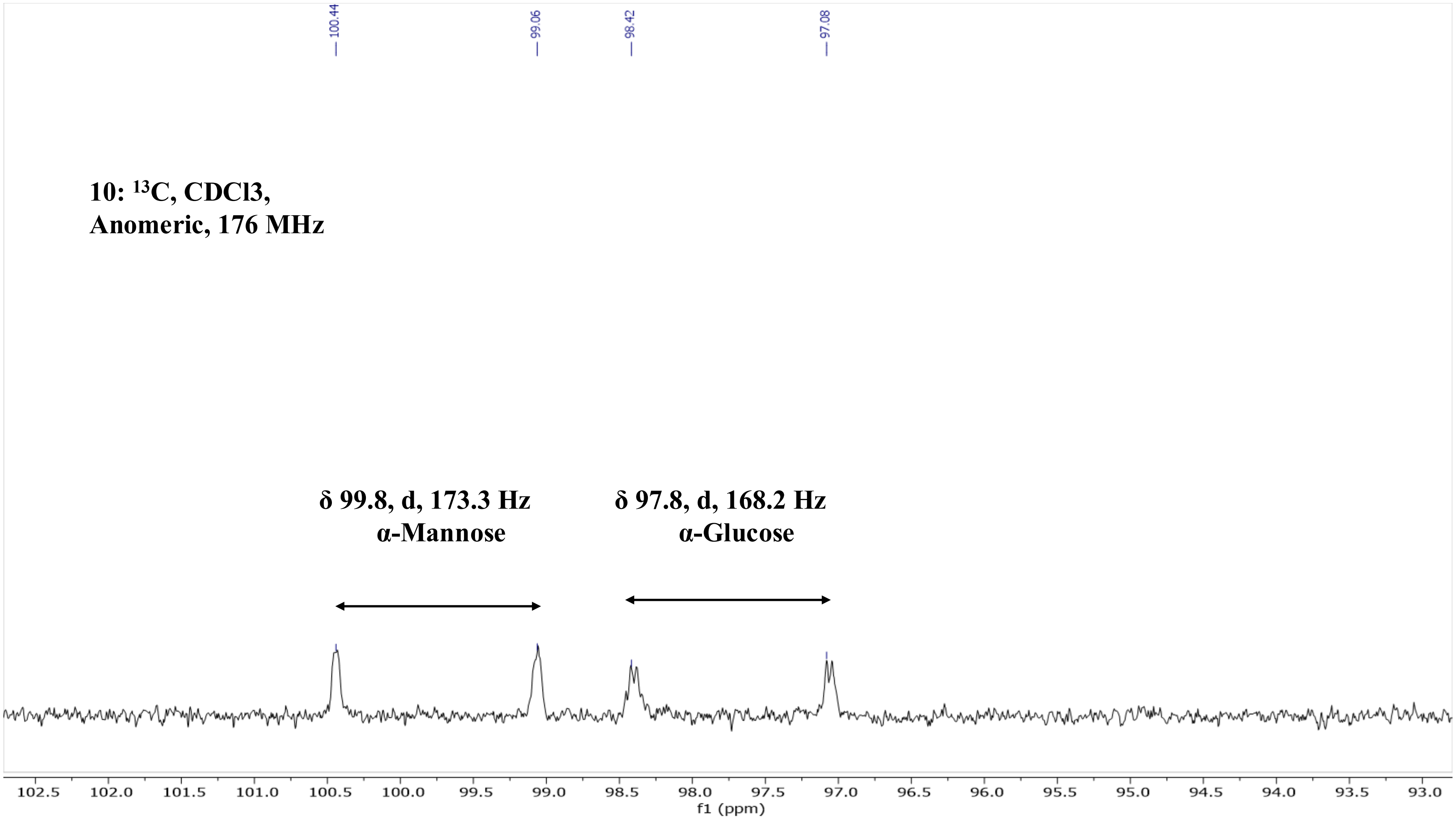

100.44
99.06
98.42
97.08
10: ¹³C, CDCl3,
Anomeric, 176 MHz
δ 99.8, d, 173.3 Hz
α-Mannose
δ 97.8, d, 168.2 Hz
α-Glucose
102.5
102.0
101.5
101.0
100.5
100.0
99.5
99.0
98.5
98.0
97.5
97.0
96.5
96.0
95.5
95.0
94.5
94.0
93.5
93.0
f1 (ppm)

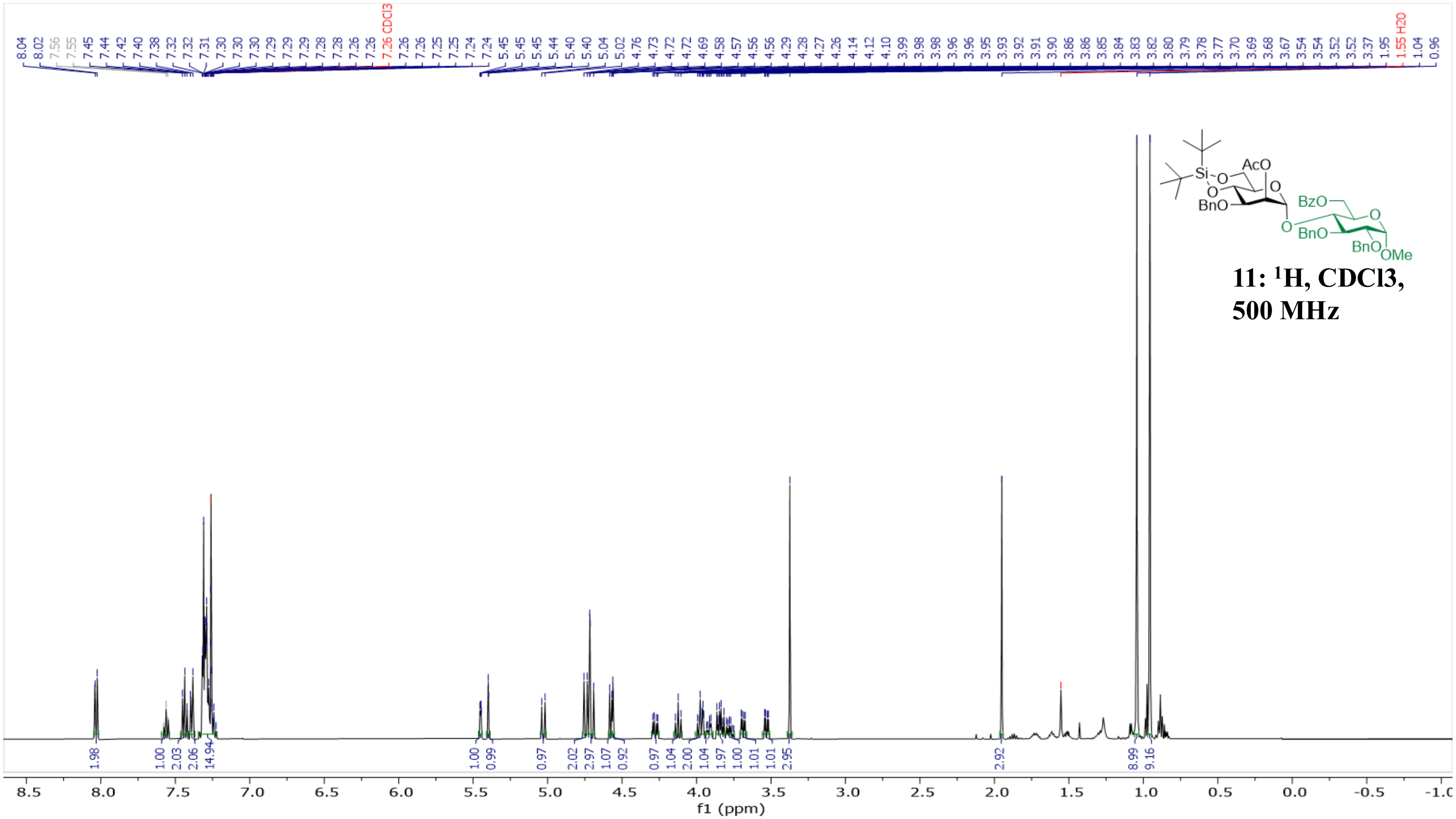

11: ^{1}H, $CDCl_3$, 500 MHz

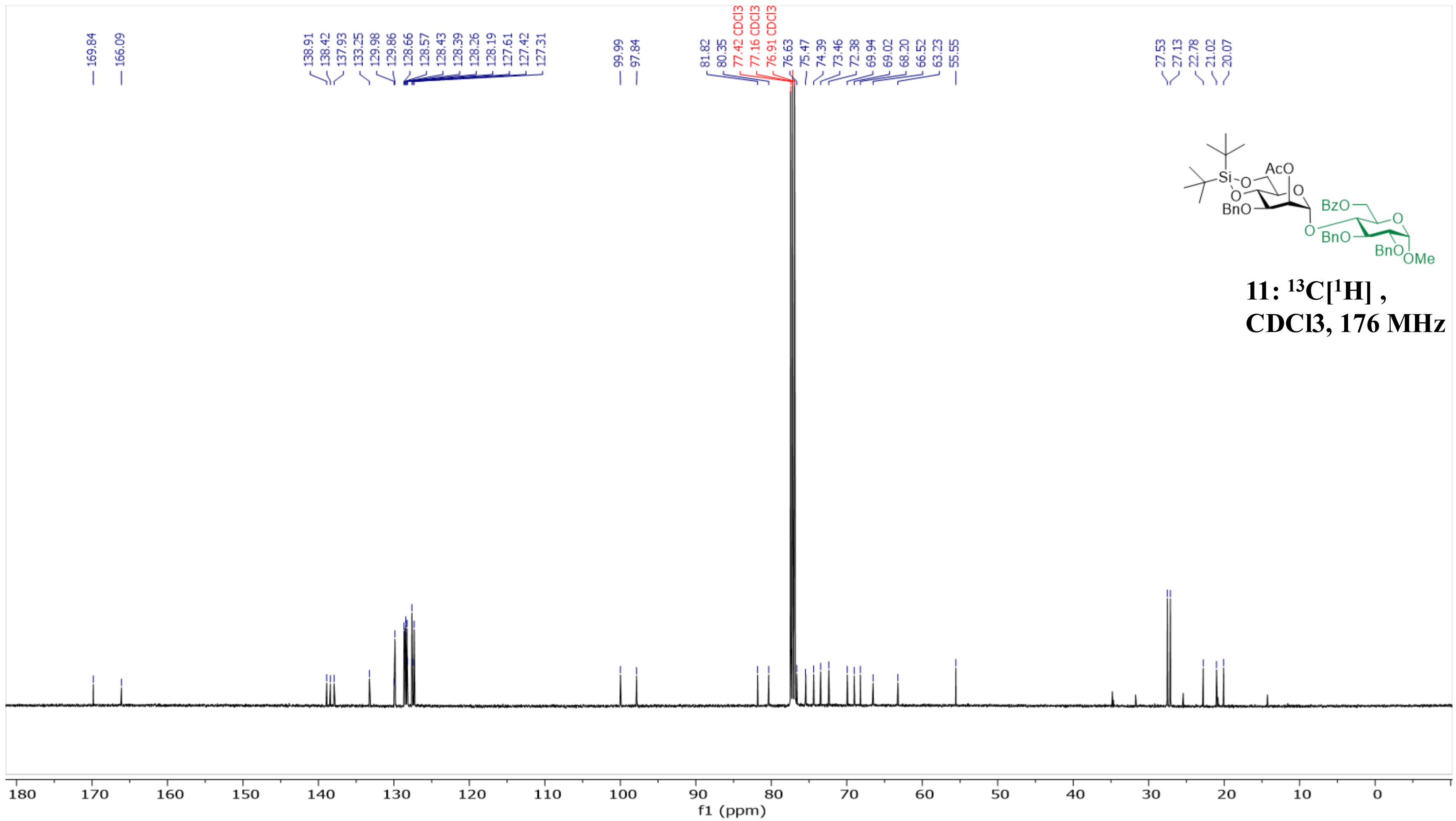
169.84
166.09
138.91
138.42
137.93
133.25
129.98
129.86
128.66
128.57
128.43
128.39
128.26
128.19
127.61
127.42
127.31
99.99
97.84
81.82
80.35
77.42 CDCl3
77.16 CDCl3
76.91 CDCl3
76.63
75.47
74.39
73.46
72.38
69.94
69.02
68.20
66.52
63.23
55.55
27.53
27.13
22.78
21.02
20.07
Si
O
AcO
BnO
BzO
OMe
11: 13C[1H] ,
CDCl3, 176 MHz
180 170 160 150 140 130 120 110 100 90 80 70 60 50 40 30 20 10 0
f1 (ppm)

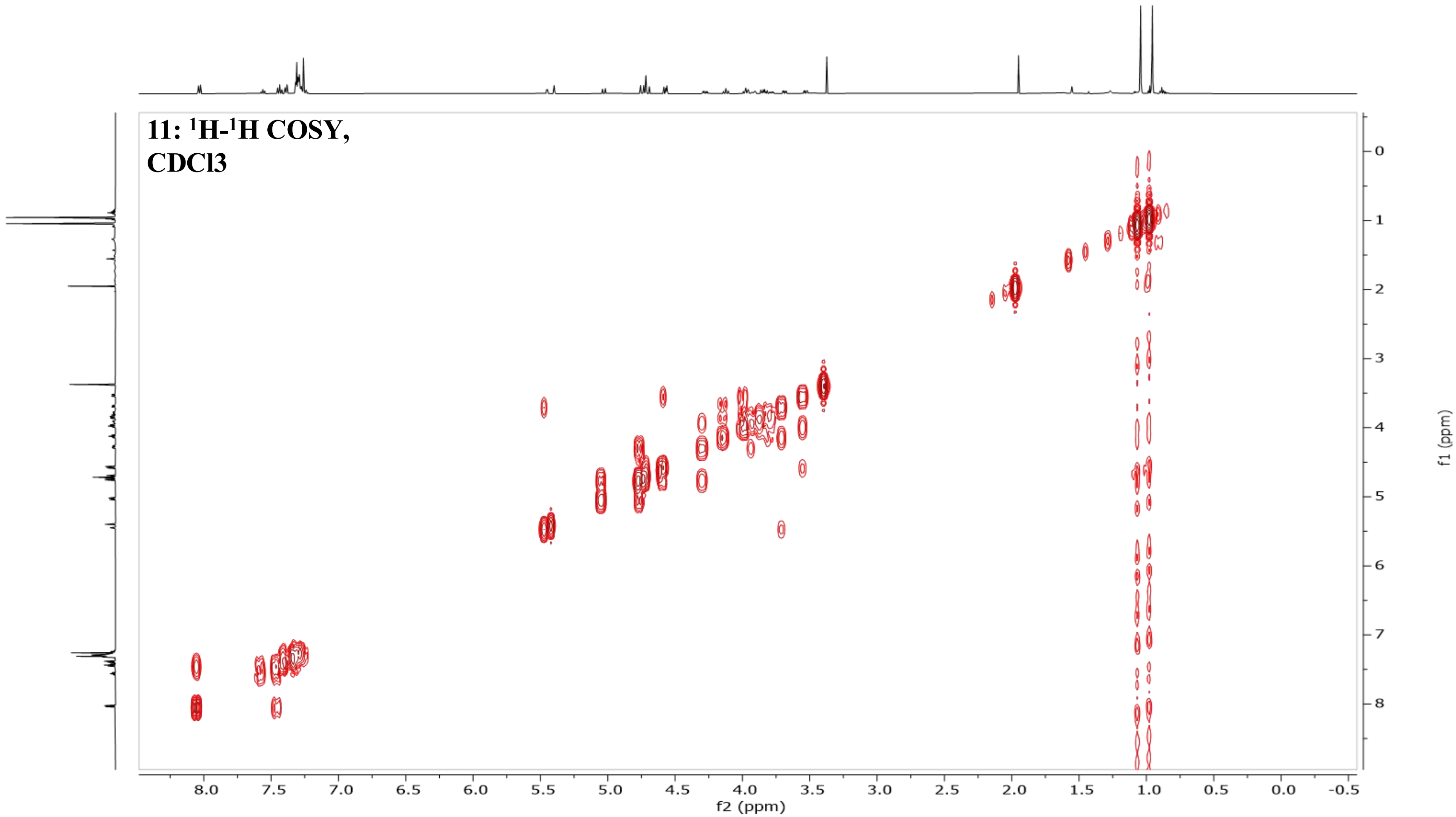
11: 1H-1H COSY,
CDCl3
f2 (ppm)
f1 (ppm)

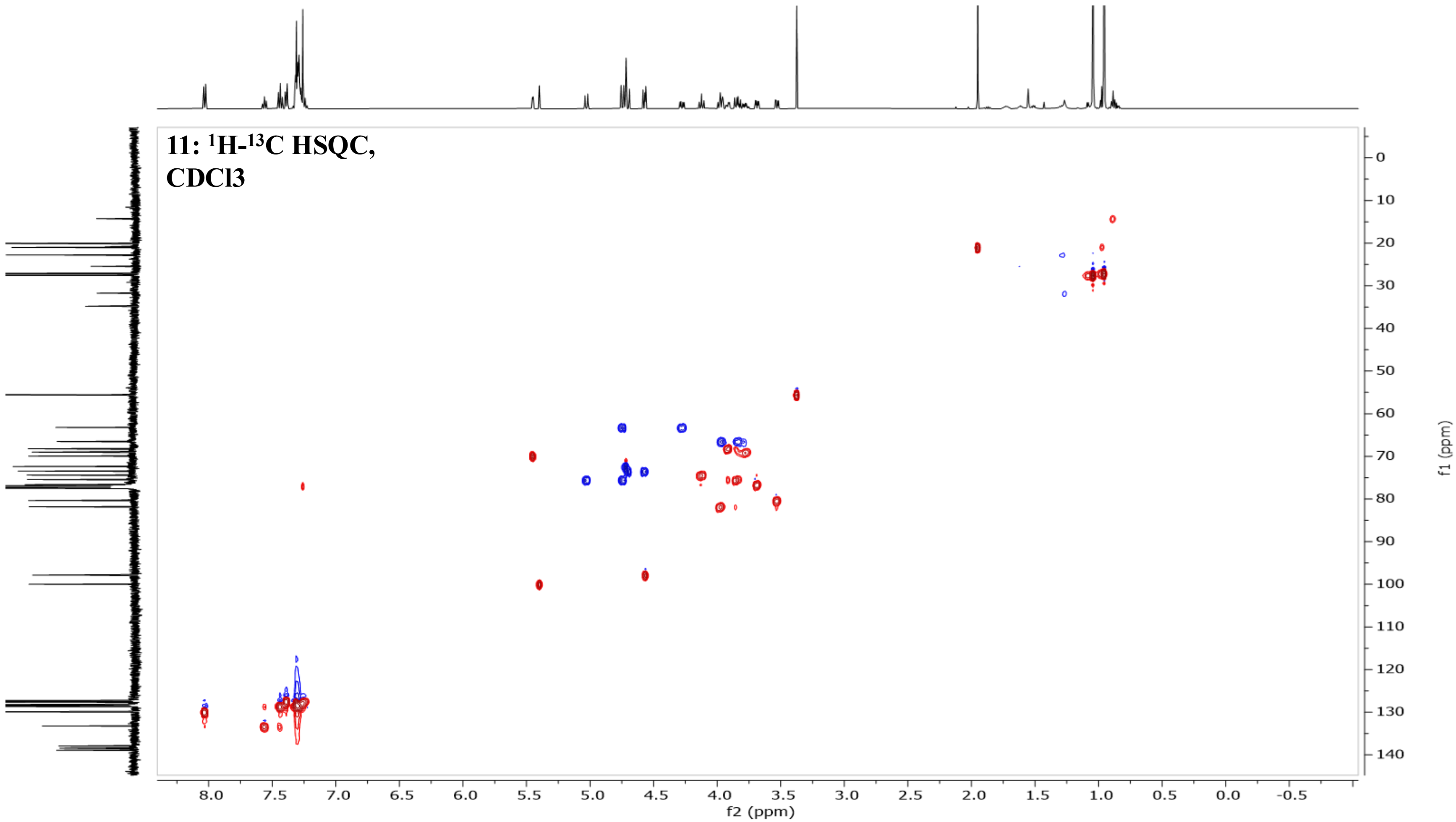
11: ^{1}H-^{13}C HSQC,
CDCl3
f2 (ppm)
f1 (ppm)

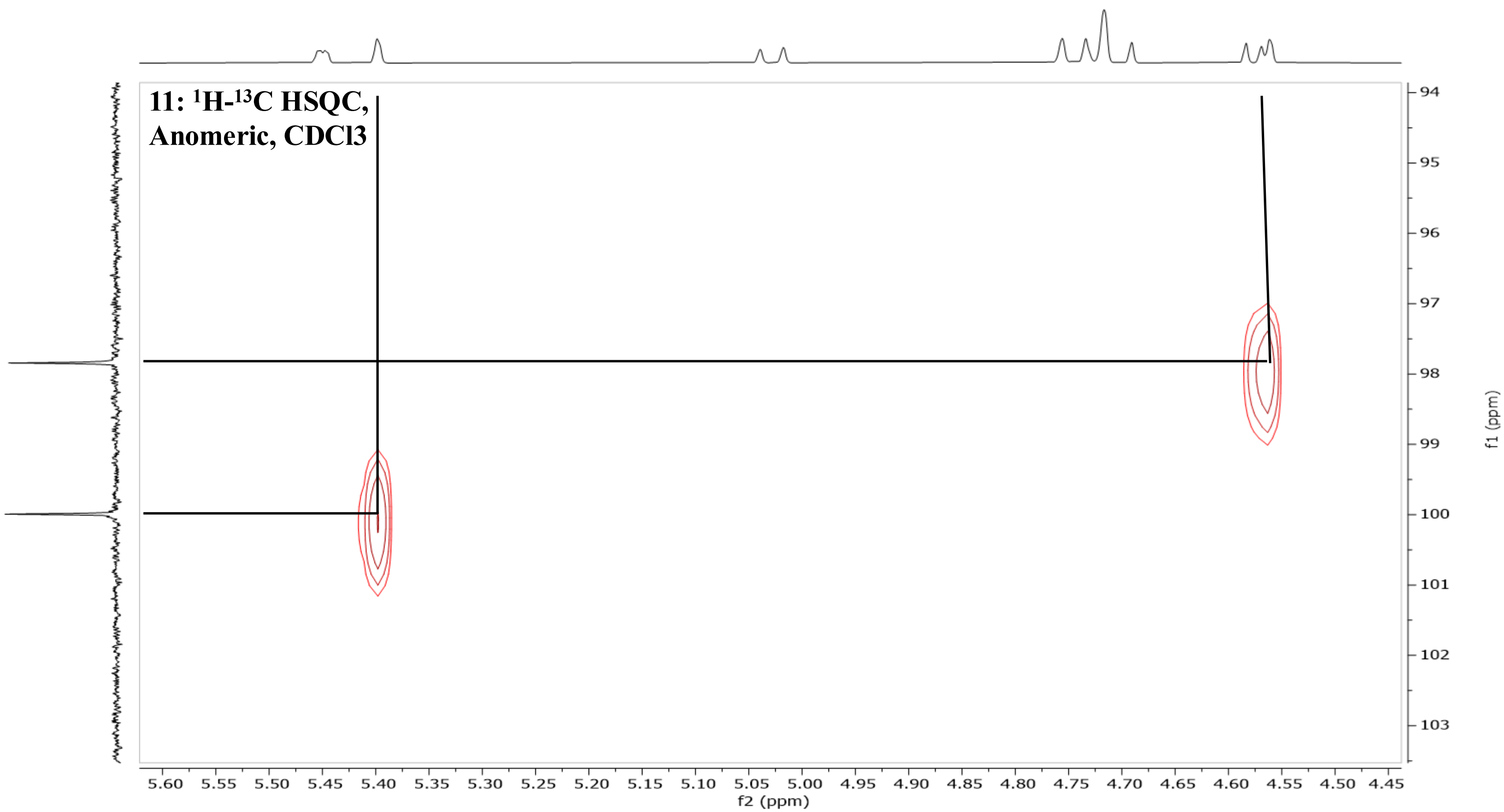
11: ¹H-¹³C HSQC,
Anomeric, CDCl3
f2 (ppm)
f1 (ppm)

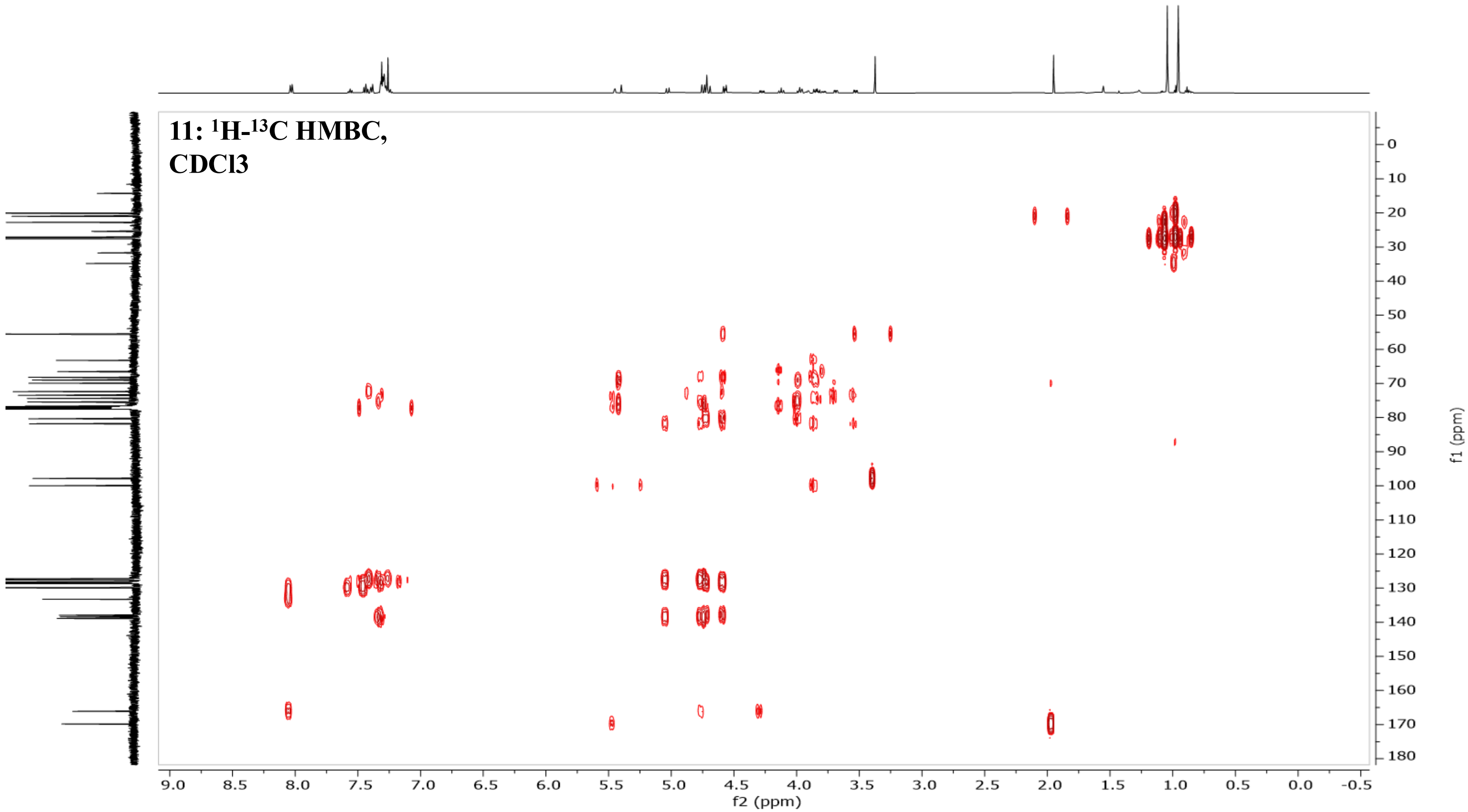

11: ^{1}H-^{13}C HMBC,
CDCl3
f2 (ppm)
f1 (ppm)

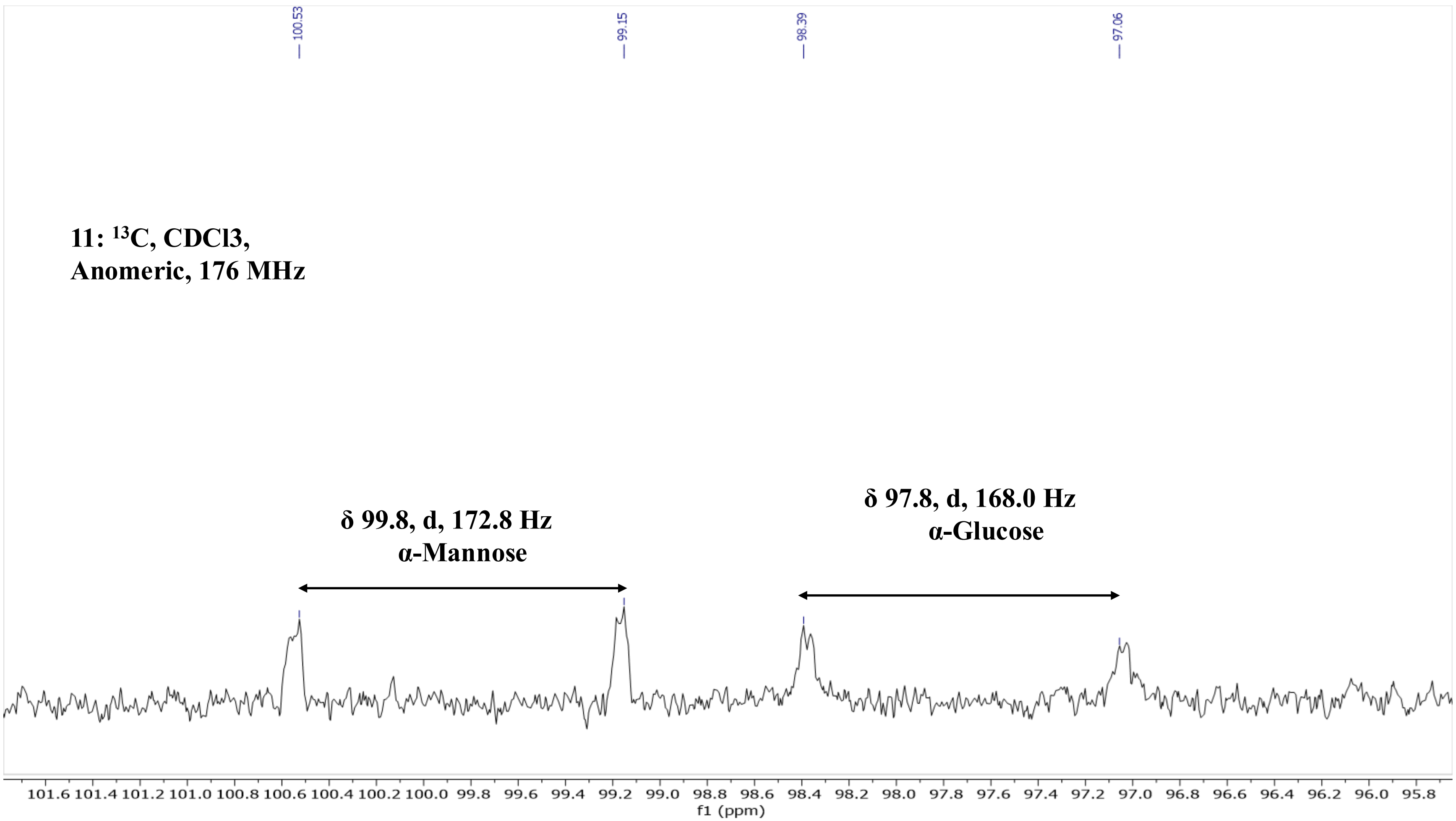

100.53
99.15
98.39
97.06
11: ¹³C, CDCl3,
Anomeric, 176 MHz
δ 99.8, d, 172.8 Hz
α-Mannose
δ 97.8, d, 168.0 Hz
α-Glucose
101.6 101.4 101.2 101.0 100.8 100.6 100.4 100.2 100.0 99.8 99.6 99.4 99.2 99.0 98.8 98.6 98.4 98.2 98.0 97.8 97.6 97.4 97.2 97.0 96.8 96.6 96.4 96.2 96.0 95.8
f1 (ppm)

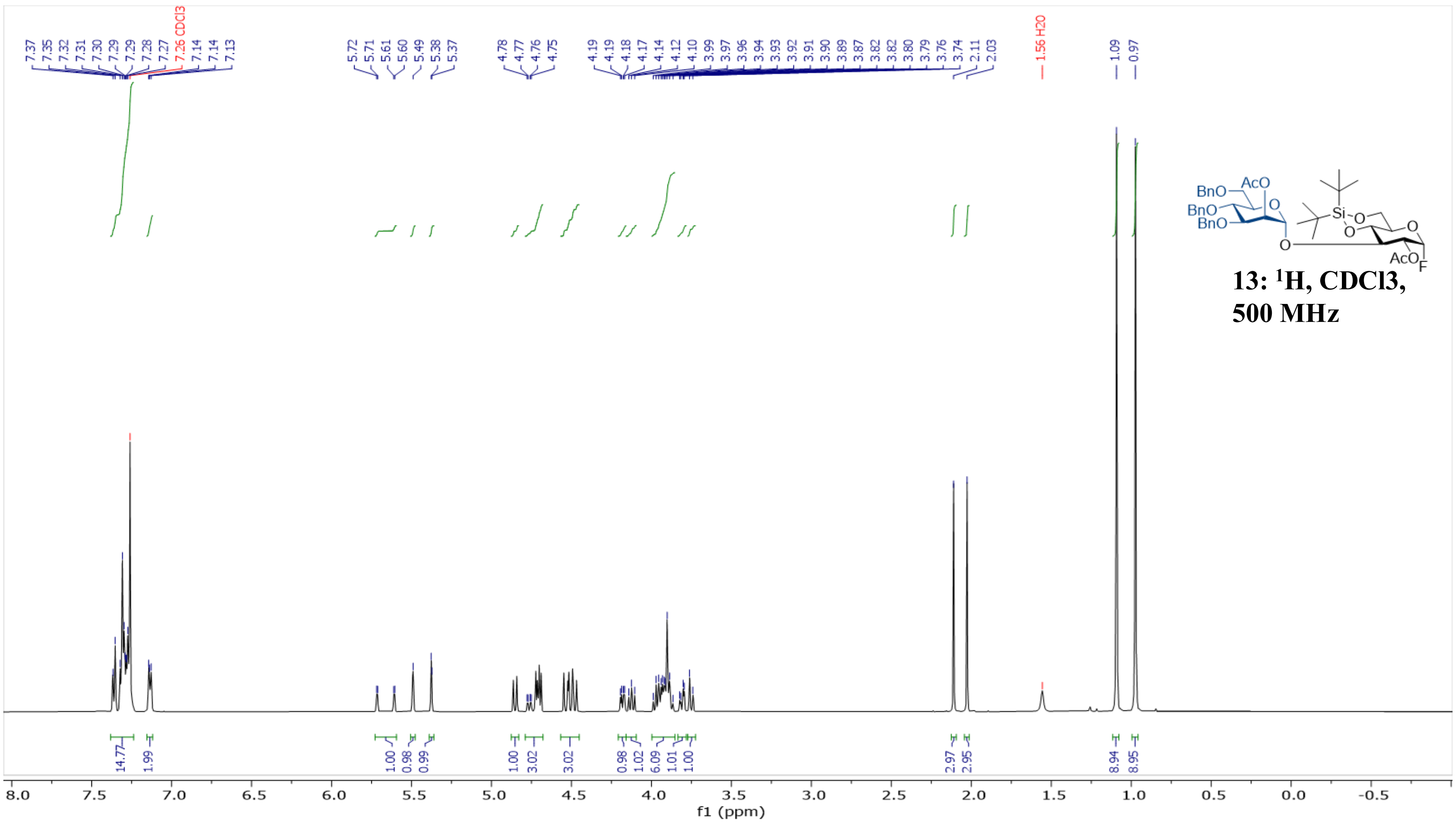
13: 1H, CDCl3,
500 MHz
BnO
AcO
BnO
BnO
Si
AcO
F
f1 (ppm)

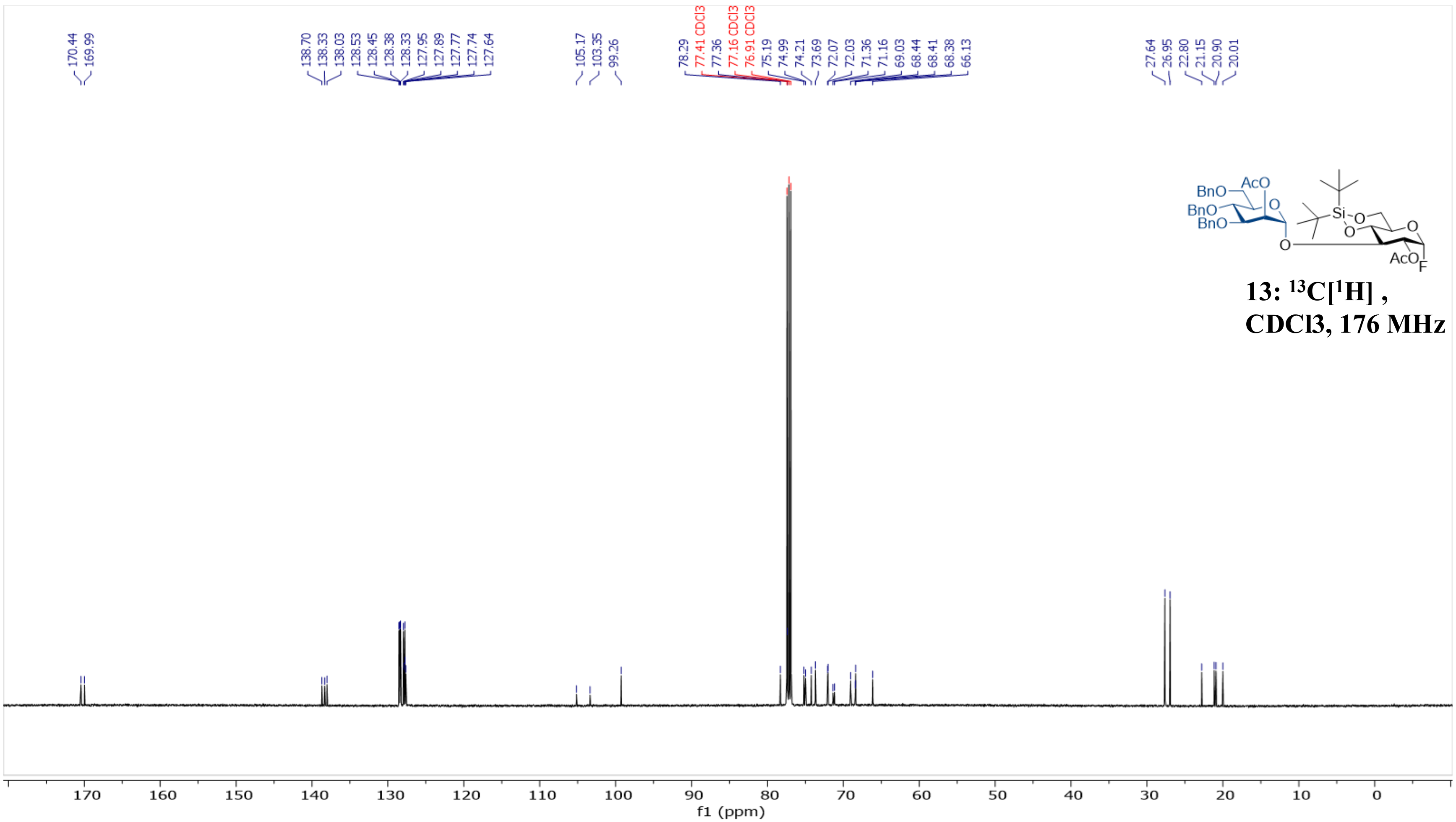

170.44
169.99
138.70
138.33
138.03
128.53
128.45
128.38
128.33
127.95
127.89
127.77
127.74
127.64
105.17
103.35
99.26
78.29
77.41 CDCl3
77.36
77.16 CDCl3
76.91 CDCl3
75.19
74.99
74.21
73.69
72.07
72.03
71.36
71.16
69.03
68.44
68.41
68.38
66.13
27.64
26.95
22.80
21.15
20.90
20.01
BnO
AcO
BnO
BnO
O
Si
O
O
O
O
AcO
F
13: 13C[1H] ,
CDCl3, 176 MHz
170
160
150
140
130
120
110
100
90
80
70
60
50
40
30
20
10
0
f1 (ppm)

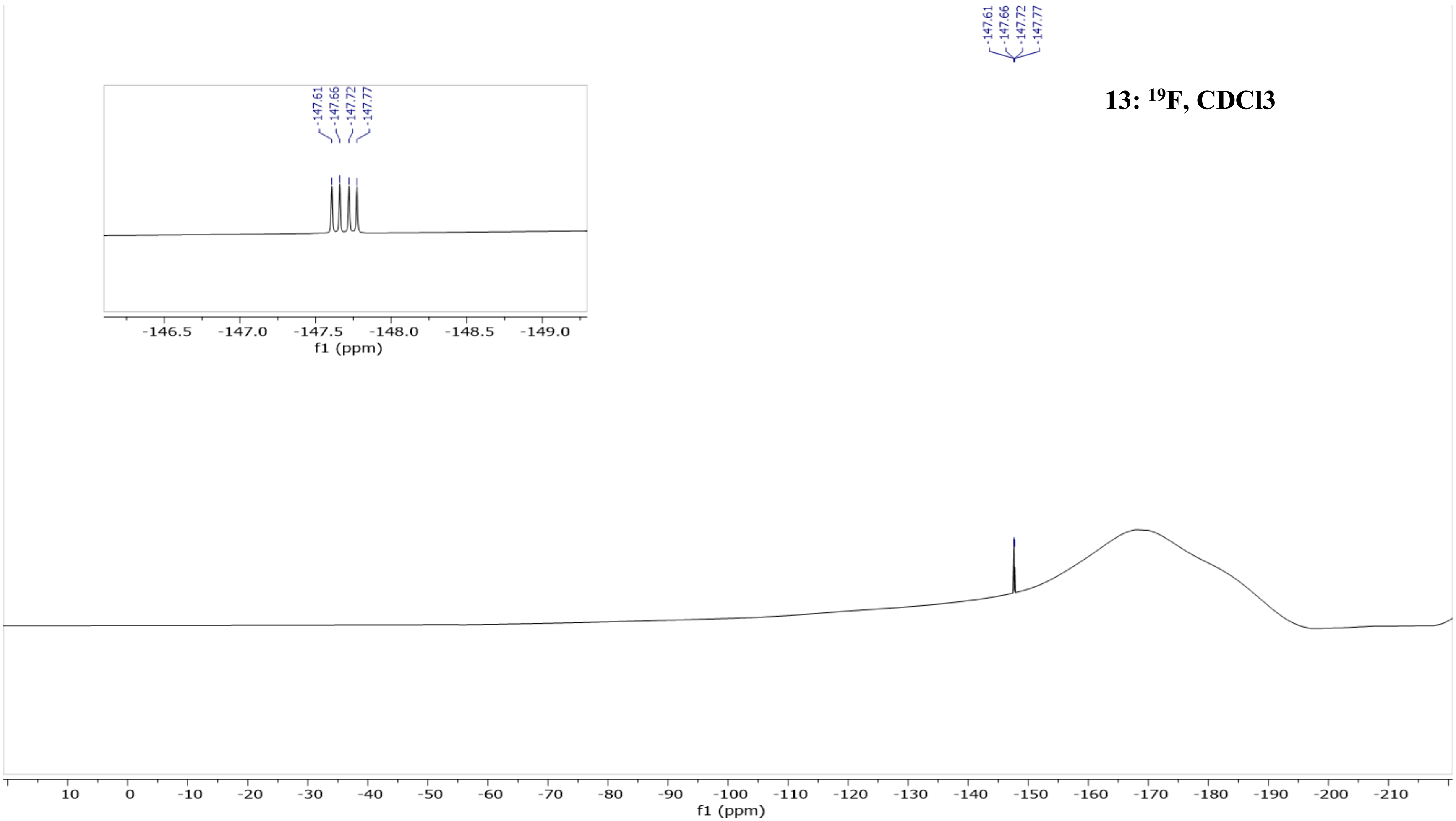

-147.61
-147.66
-147.72
-147.77
13: ^{19}F, CDCl3
-146.5 -147.0 -147.5 -148.0 -148.5 -149.0
f1 (ppm)
10 0 -10 -20 -30 -40 -50 -60 -70 -80 -90 -100 -110 -120 -130 -140 -150 -160 -170 -180 -190 -200 -210
f1 (ppm)

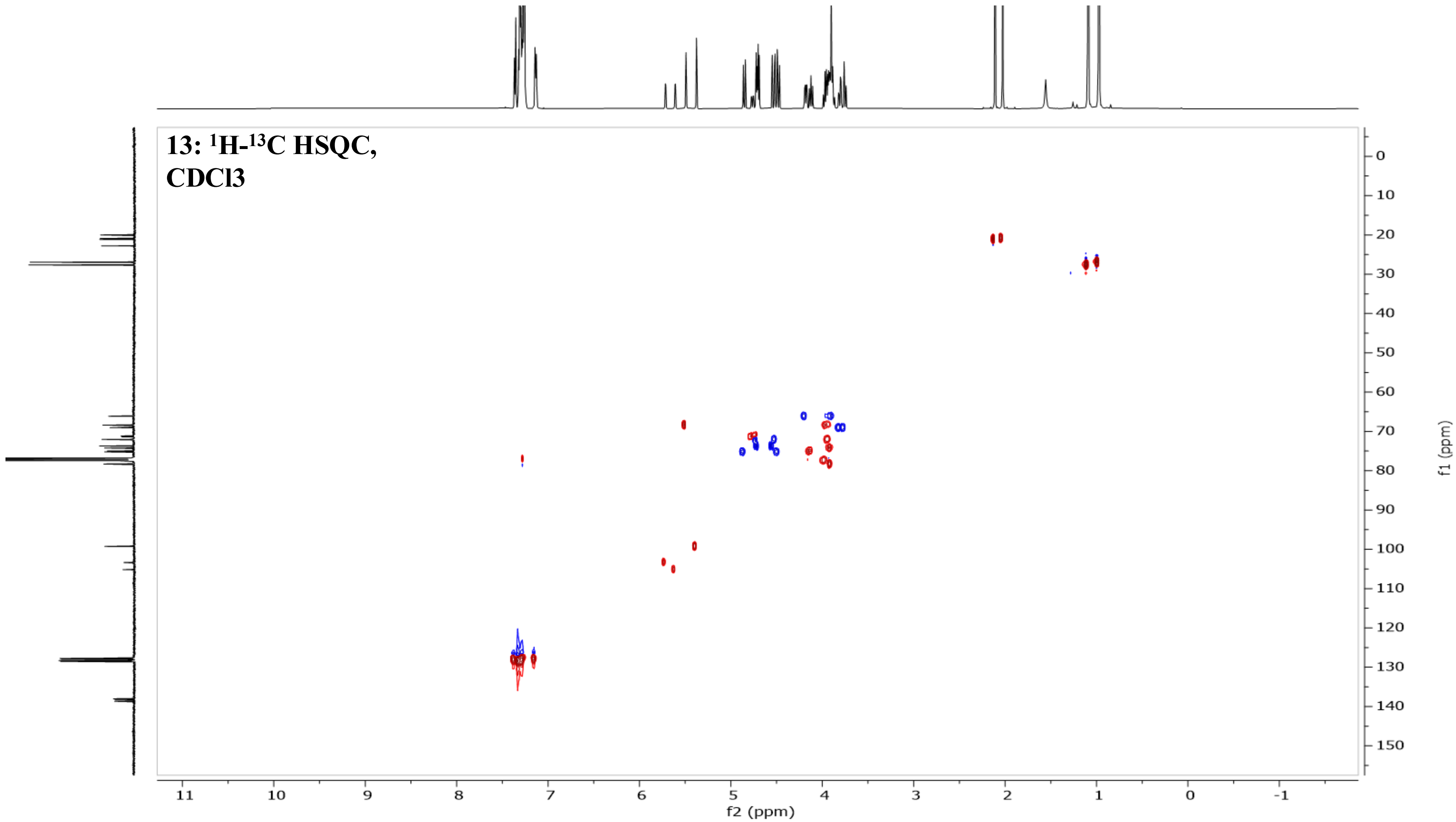

13: ¹H-¹³C HSQC,
CDCl3
f2 (ppm)
f1 (ppm)

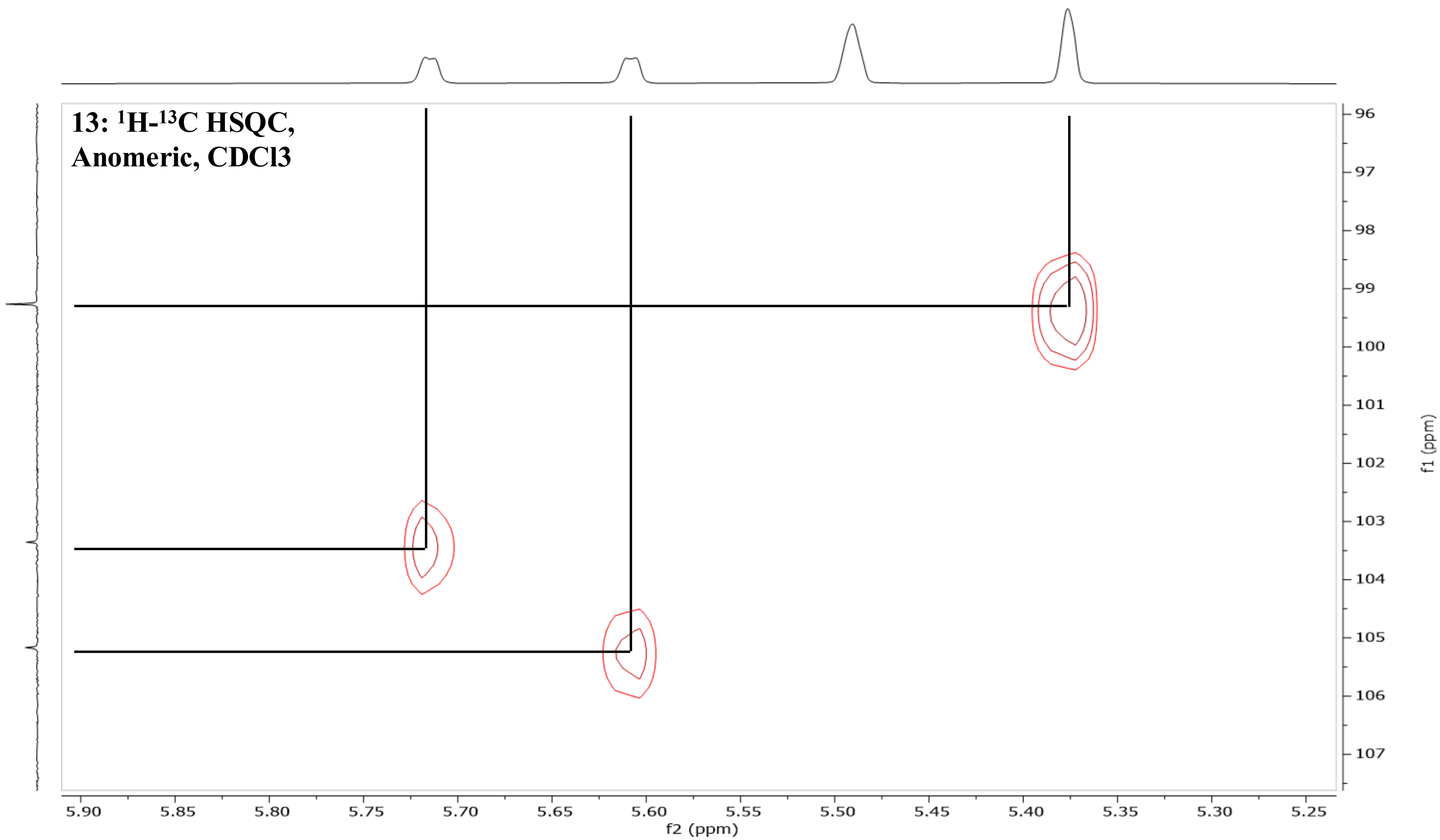
13: 1H-13C HSQC,
Anomeric, CDCl3
f2 (ppm)
f1 (ppm)
5.90
5.85
5.80
5.75
5.70
5.65
5.60
5.55
5.50
5.45
5.40
5.35
5.30
5.25
96
97
98
99
100
101
102
103
104
105
106
107

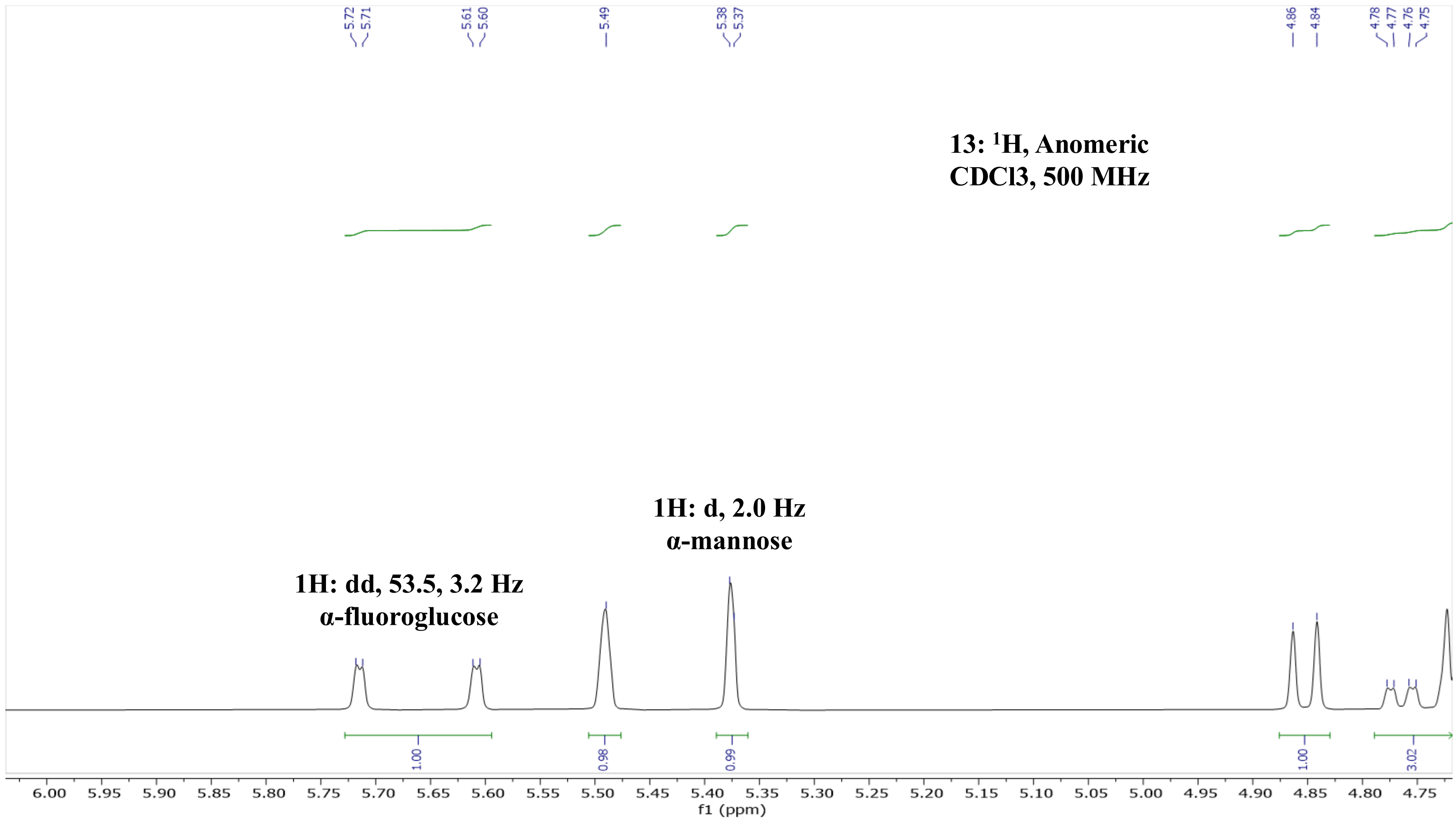
5.72
5.71
5.61
5.60
5.49
5.38
5.37
4.86
4.84
4.78
4.77
4.76
4.75
13: ¹H, Anomeric
CDCl3, 500 MHz
1H: d, 2.0 Hz
α-mannose
1H: dd, 53.5, 3.2 Hz
α-fluoroglucose
1.00
0.98
0.99
1.00
3.02
6.00 5.95 5.90 5.85 5.80 5.75 5.70 5.65 5.60 5.55 5.50 5.45 5.40 5.35 5.30 5.25 5.20 5.15 5.10 5.05 5.00 4.95 4.90 4.85 4.80 4.75
f1 (ppm)

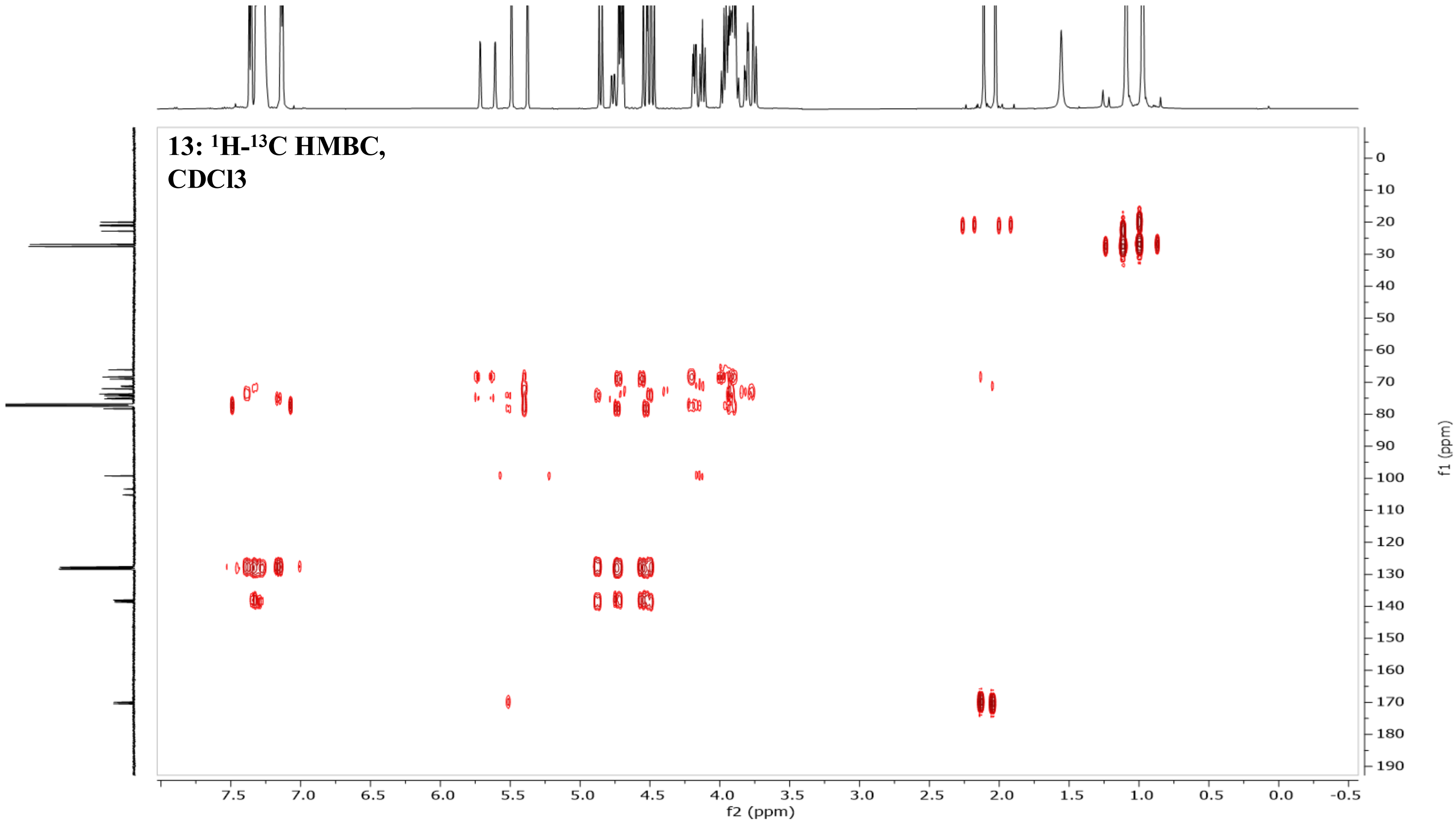
13: 1H-13C HMBC,
CDCl3
f2 (ppm)
f1 (ppm)

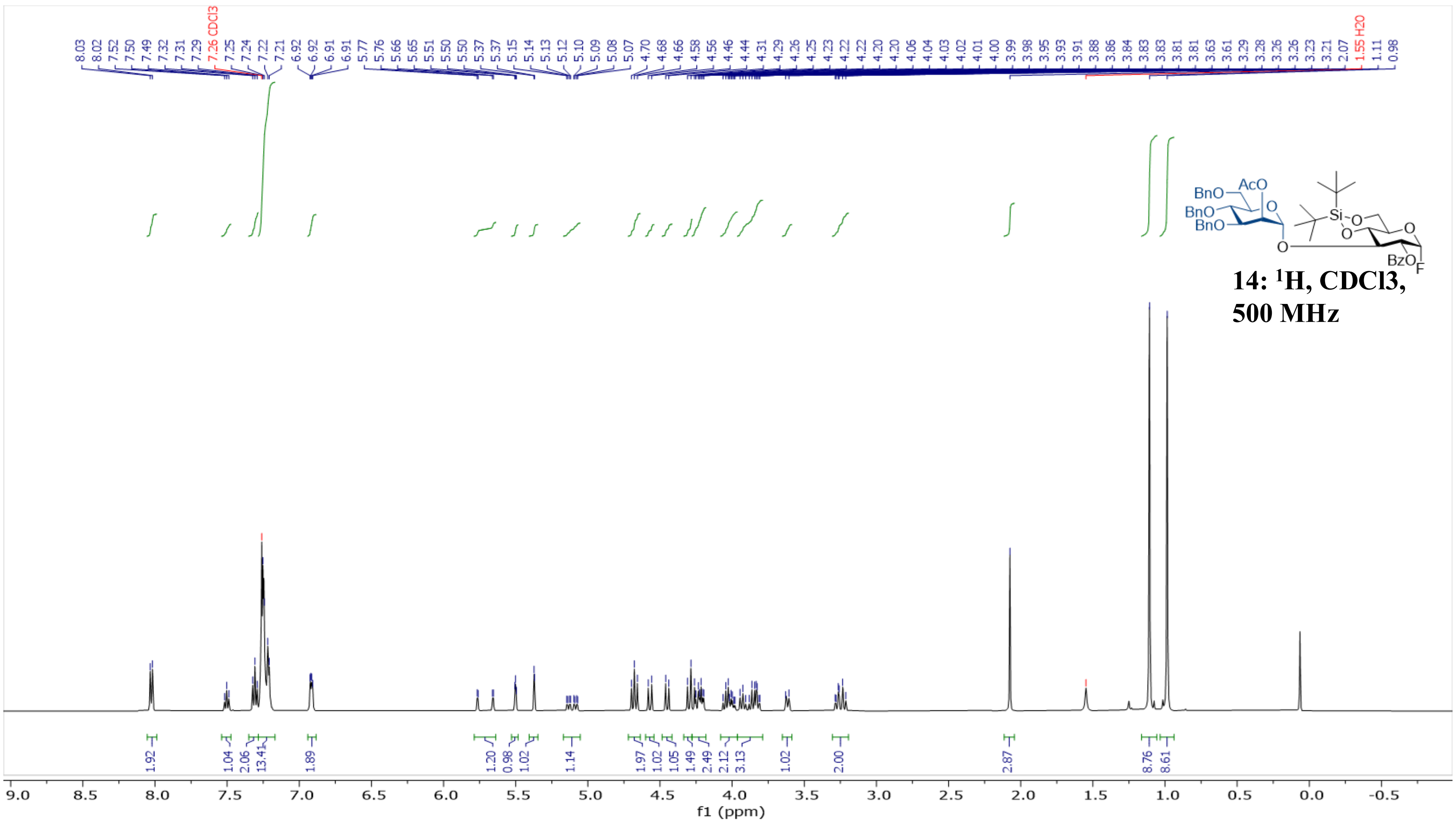
14: ¹H, CDCl3,
500 MHz
f1 (ppm)

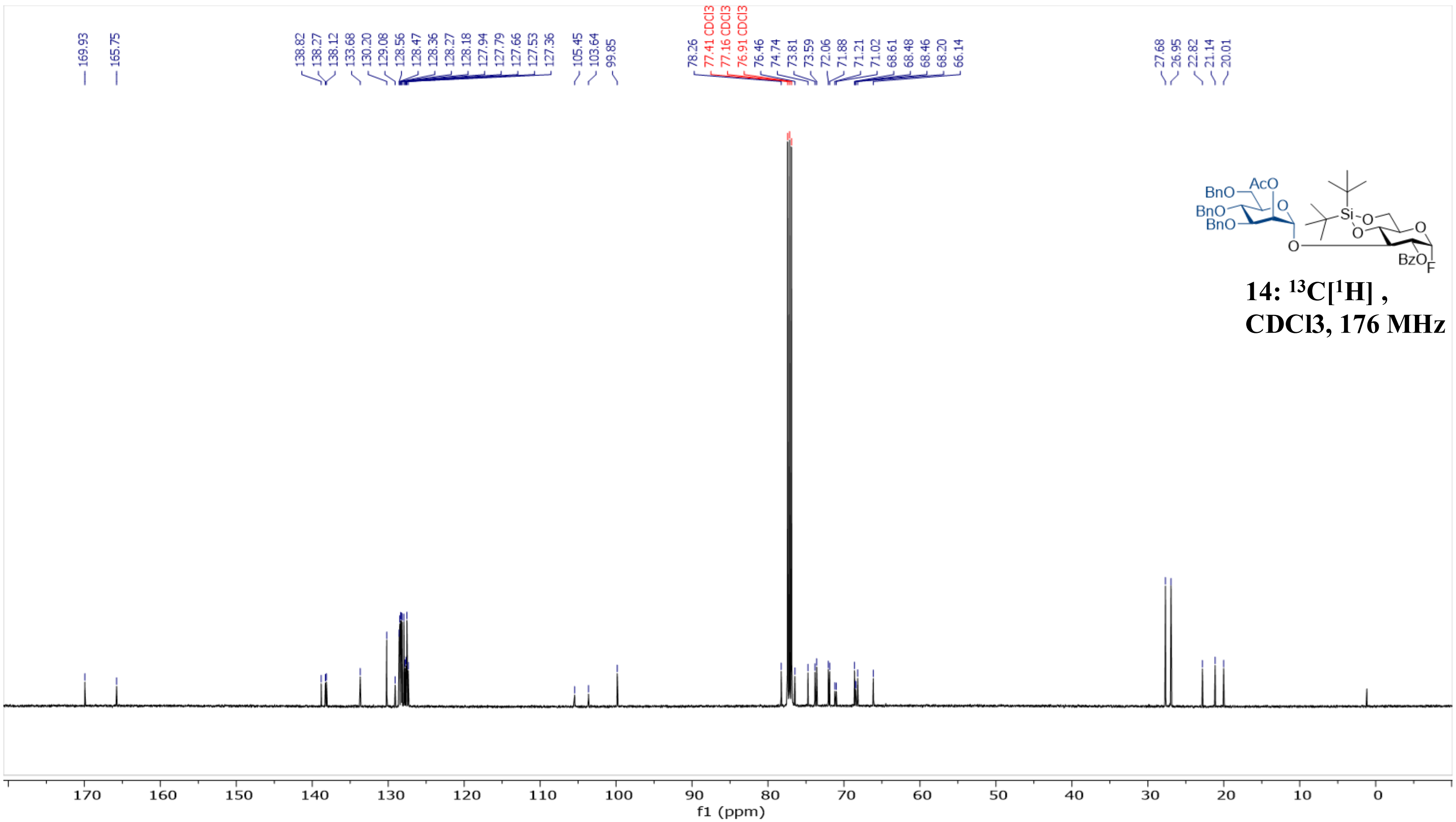
169.93
165.75
138.82
138.27
138.12
133.68
130.20
129.08
128.56
128.47
128.36
128.27
128.18
127.94
127.79
127.66
127.53
127.36
105.45
103.64
99.85
78.26
77.41 CDCl3
77.16 CDCl3
76.91 CDCl3
76.46
74.74
73.81
73.59
72.06
71.88
71.21
71.02
68.61
68.48
68.46
68.20
66.14
27.68
26.95
22.82
21.14
20.01
BnO
AcO
O
BnO
BnO
Si
O
O
O
O
BzO
F
14: $^{13}C[^{1}H]$,
CDCl3, 176 MHz
170 160 150 140 130 120 110 100 90 80 70 60 50 40 30 20 10 0
f1 (ppm)

14: ^{19}F, CDCl3

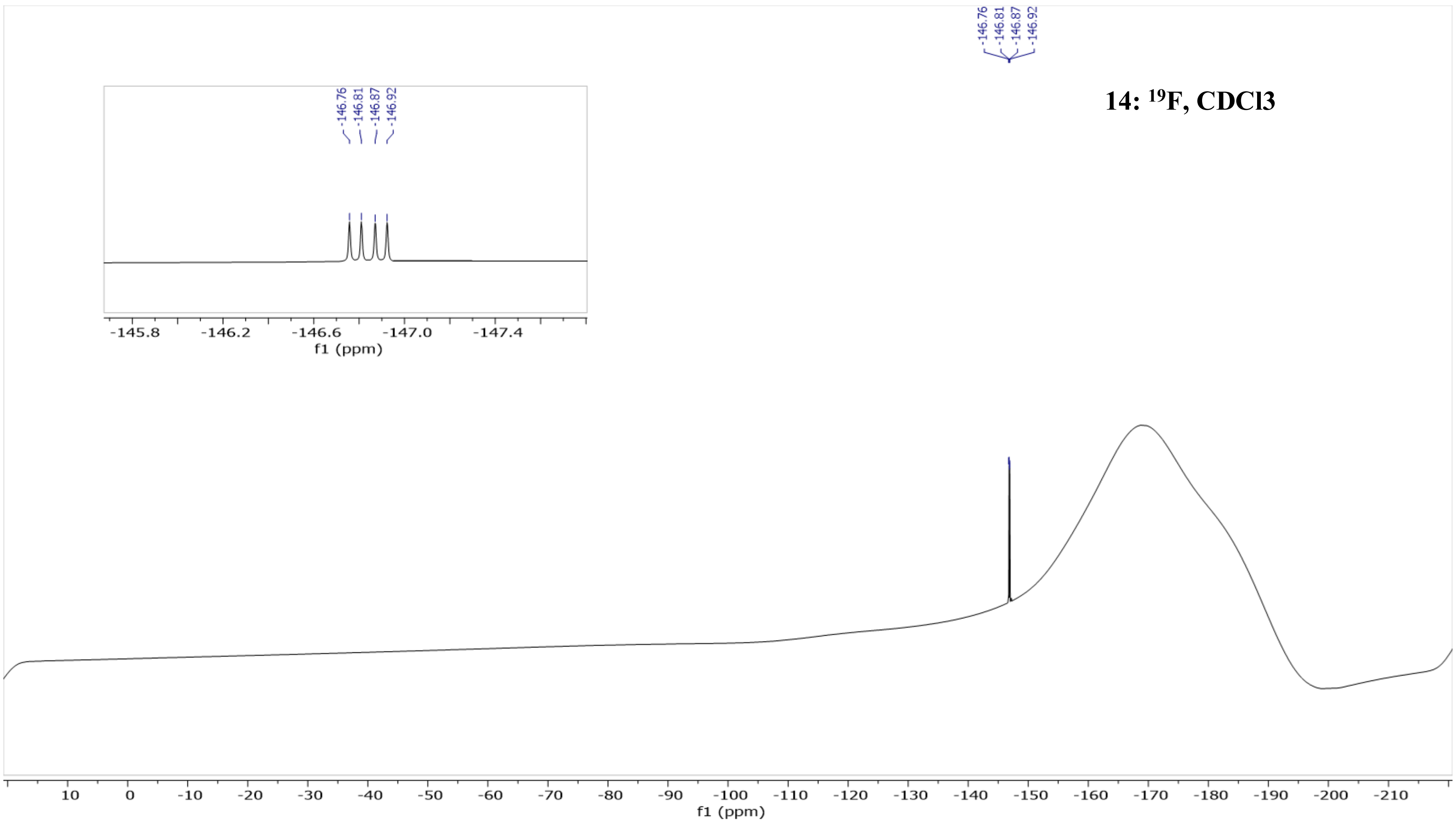
-146.76
-146.81
-146.87
-146.92
-145.8
-146.2
-146.6
-147.0
-147.4
f1 (ppm)
10
0
-10
-20
-30
-40
-50
-60
-70
-80
-90
-100
-110
-120
-130
-140
-150
-160
-170
-180
-190
-200
-210
f1 (ppm)

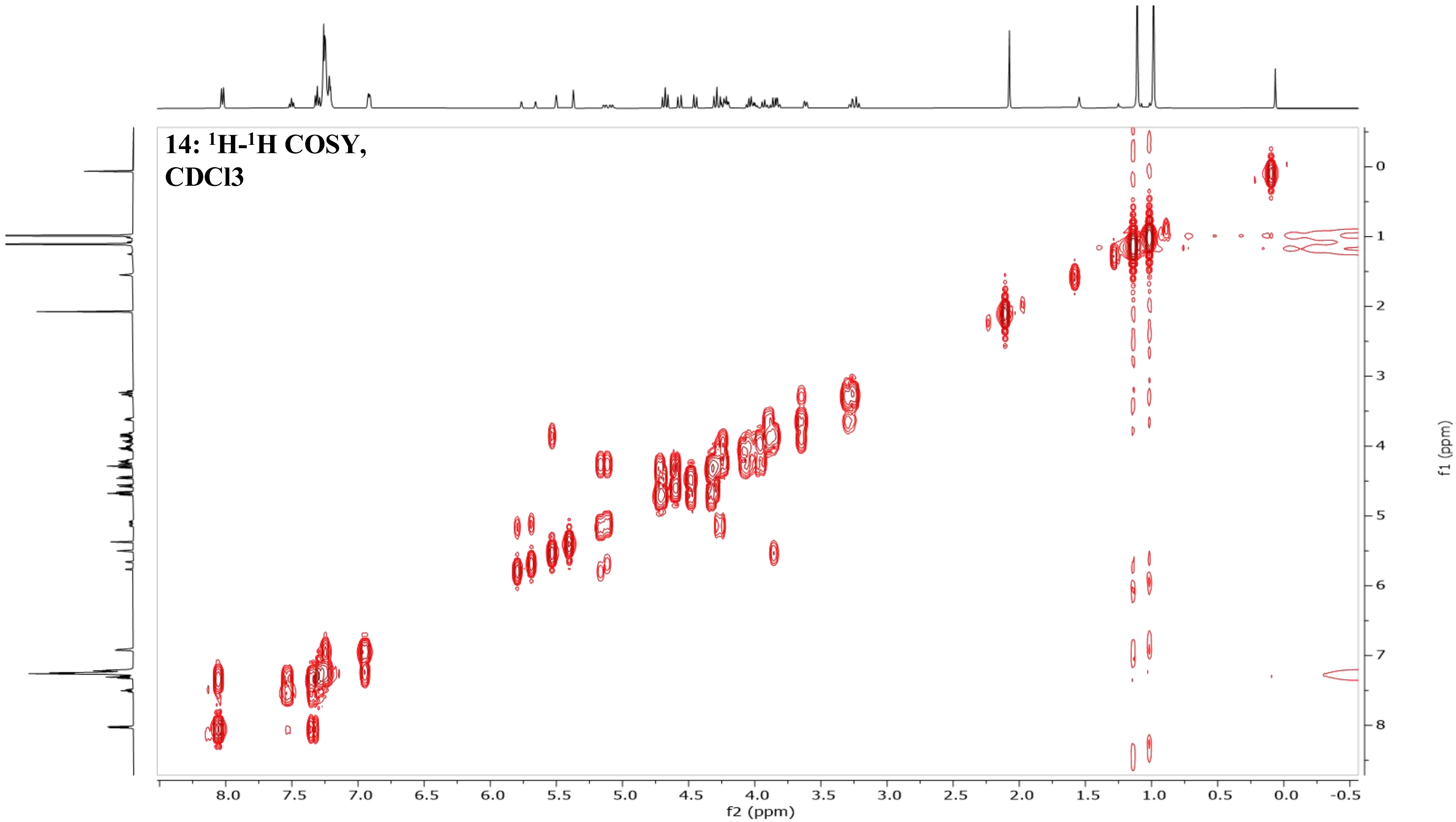
14: 1H-1H COSY,
CDCl3
f2 (ppm)
f1 (ppm)

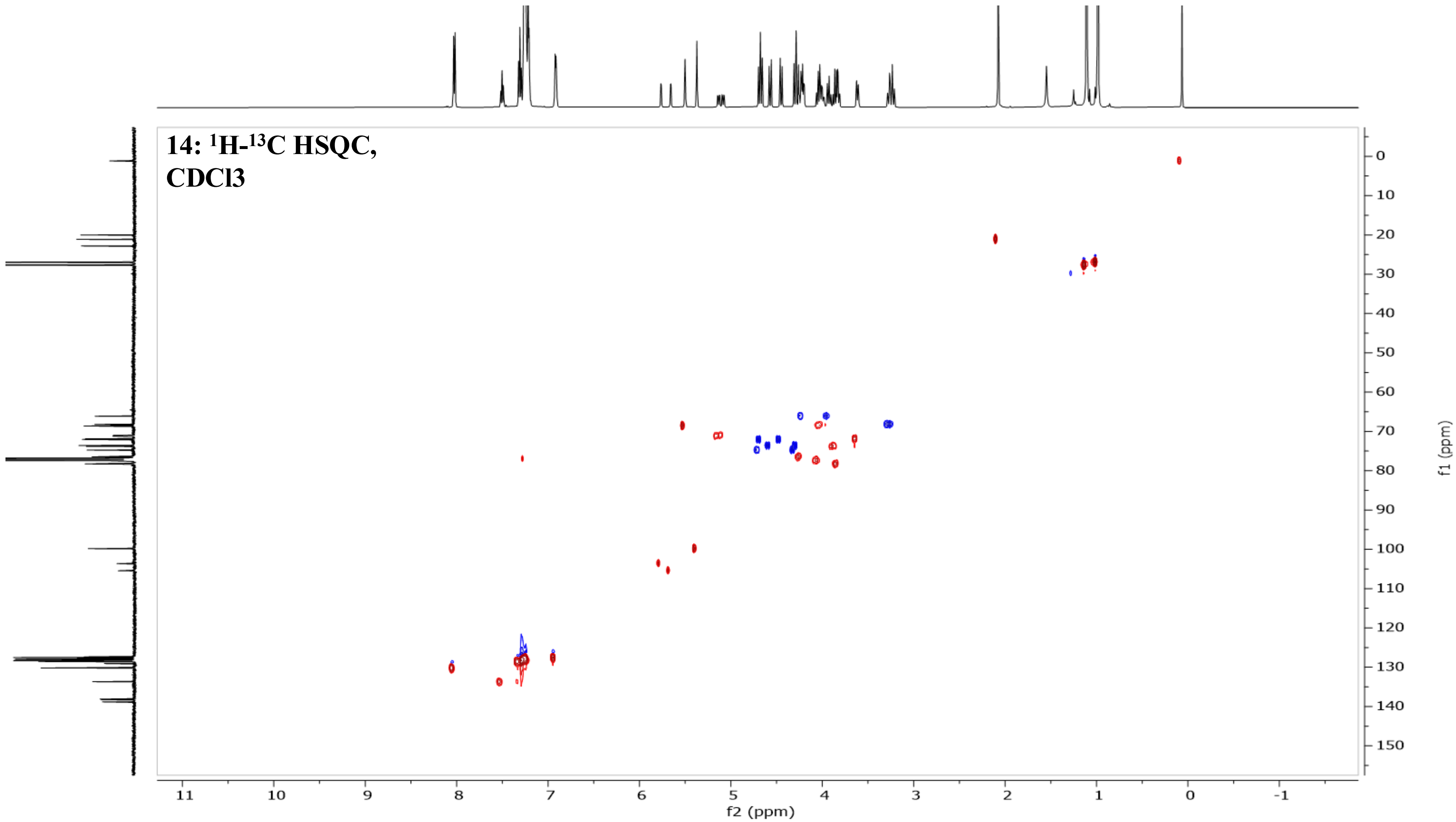
14: ¹H-¹³C HSQC,
CDCl3
f2 (ppm)
f1 (ppm)

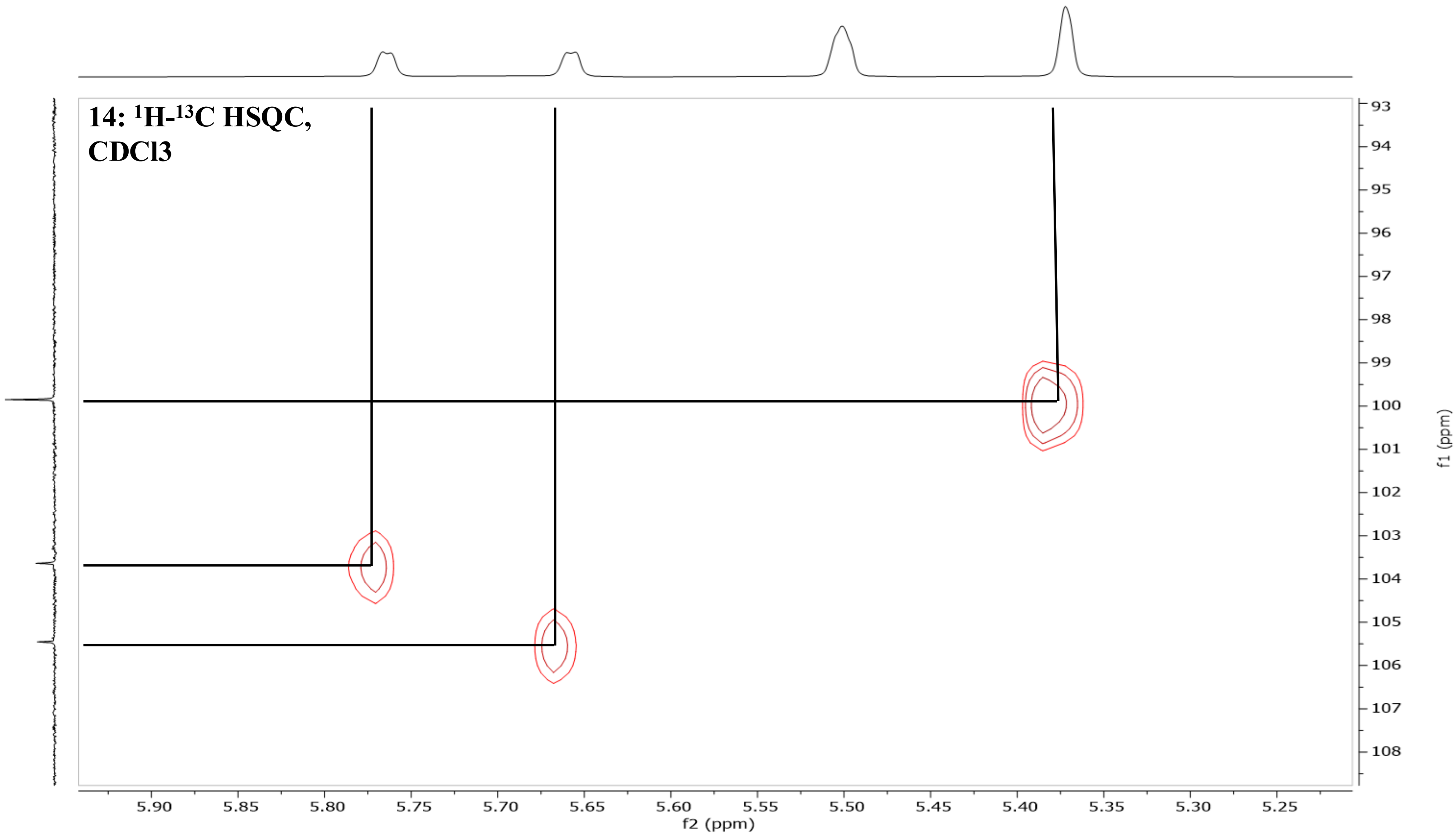
14: 1H-13C HSQC, CDCl3
f2 (ppm)
f1 (ppm)
5.90
5.85
5.80
5.75
5.70
5.65
5.60
5.55
5.50
5.45
5.40
5.35
5.30
5.25
93
94
95
96
97
98
99
100
101
102
103
104
105
106
107
108

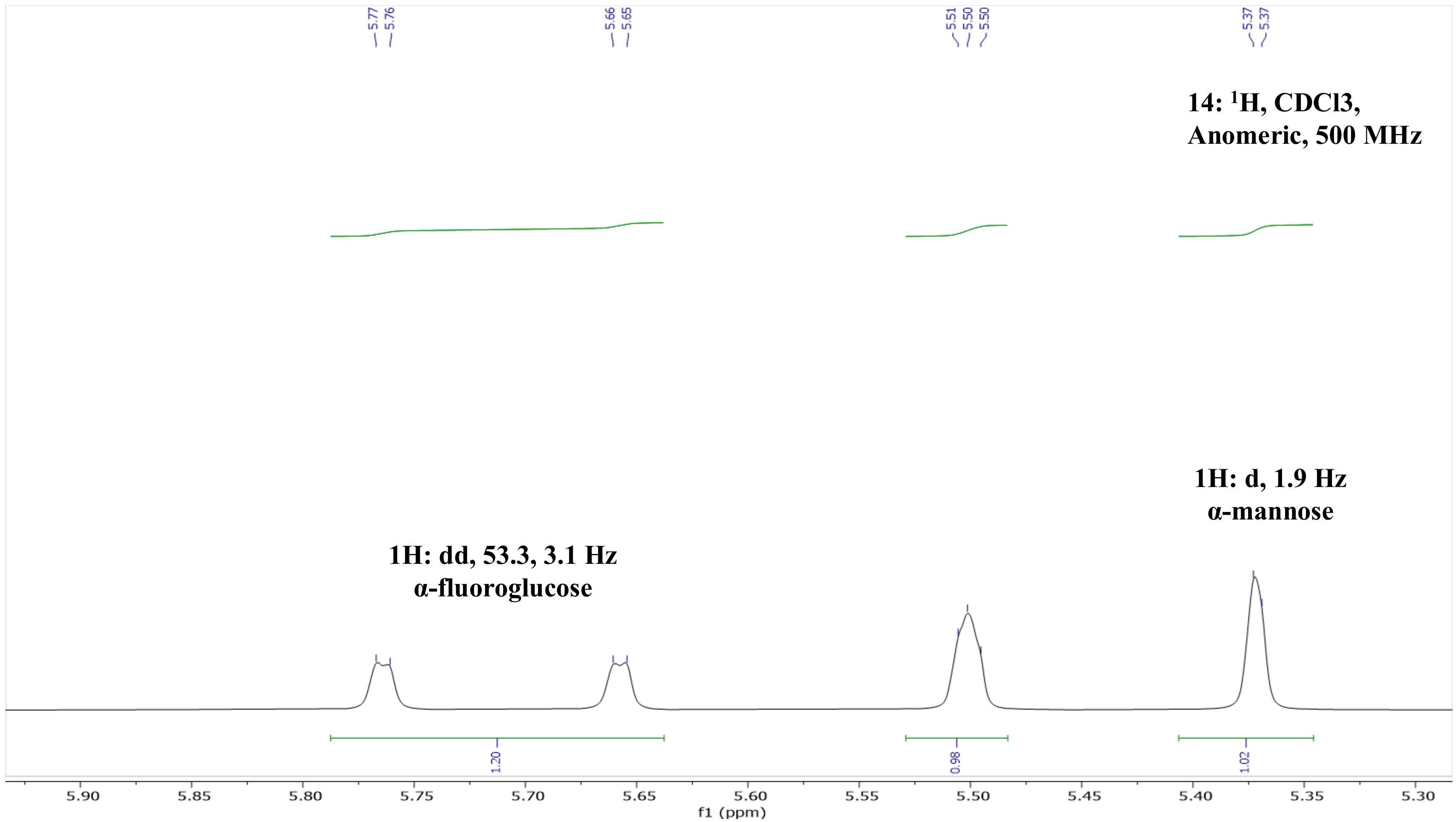

5.77
5.76
5.66
5.65
5.51
5.50
5.50
5.37
5.37
14: ¹H, CDCl3,
Anomeric, 500 MHz
1H: d, 1.9 Hz
α-mannose
1H: dd, 53.3, 3.1 Hz
α-fluoroglucose
1.20
0.98
1.02
5.90
5.85
5.80
5.75
5.70
5.65
5.60
5.55
5.50
5.45
5.40
5.35
5.30
f1 (ppm)

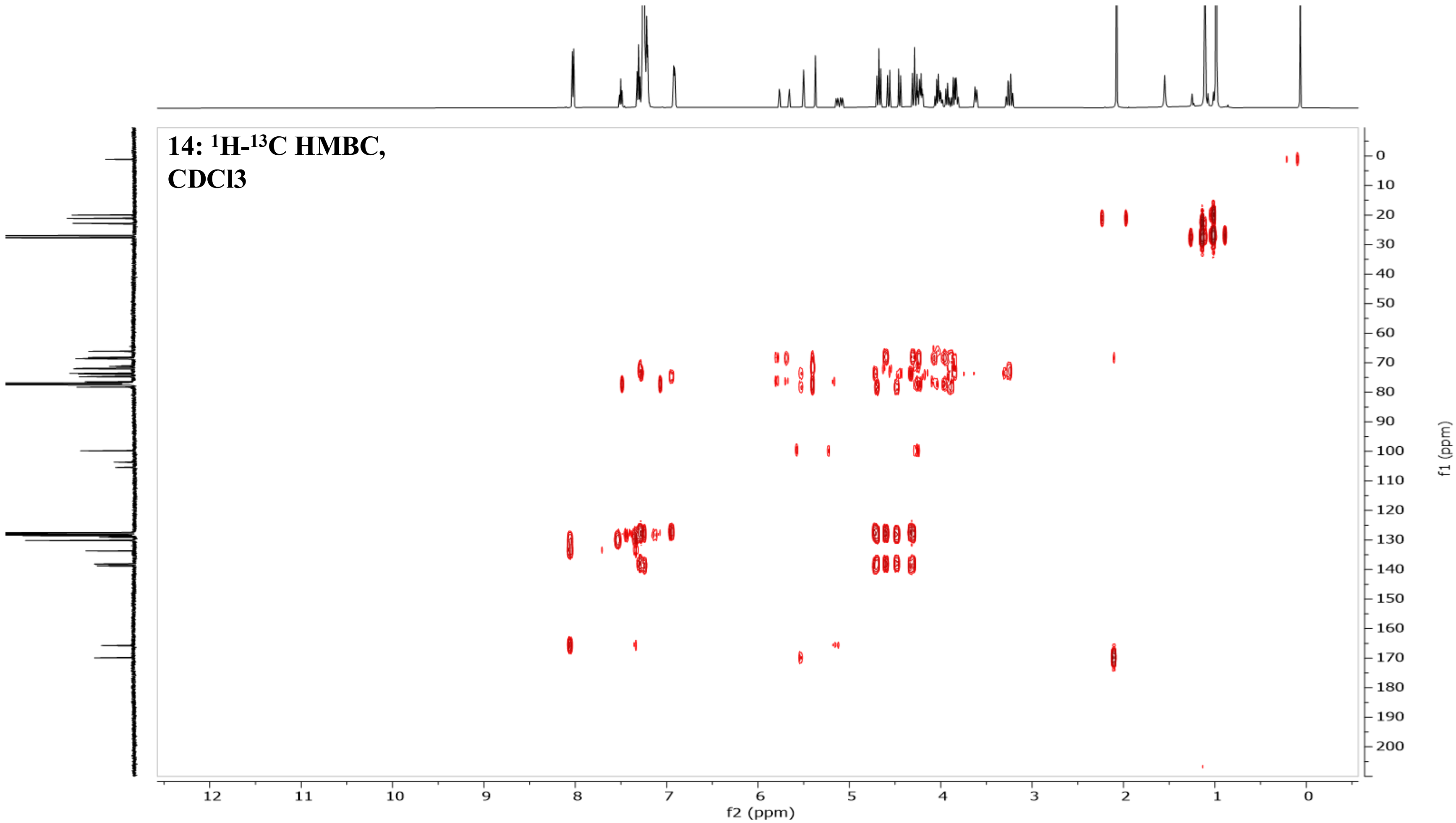
14: ¹H-¹³C HMBC,
CDCl3
f2 (ppm)
f1 (ppm)

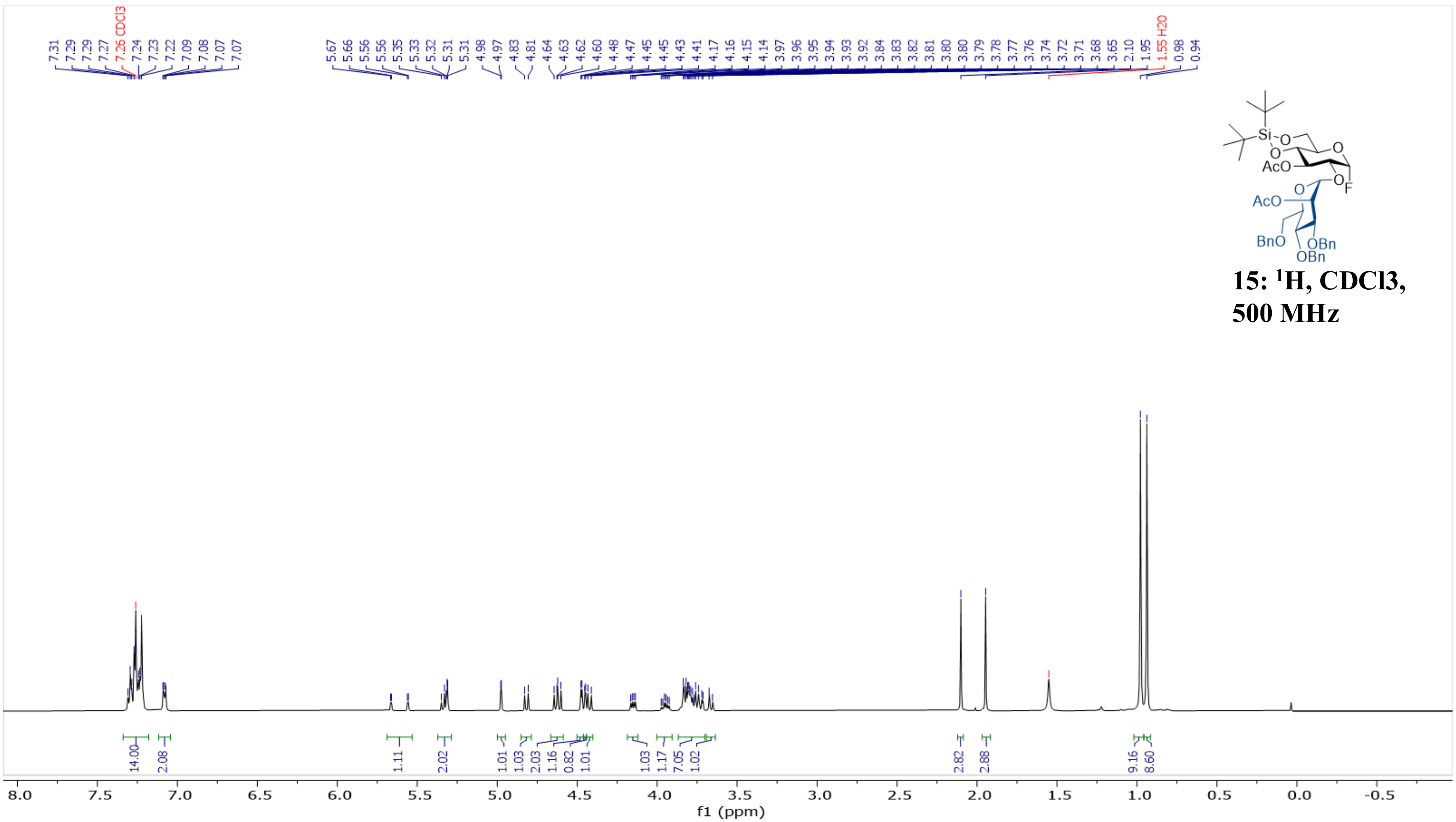

15: ¹H, CDCl3, 500 MHz
f1 (ppm)

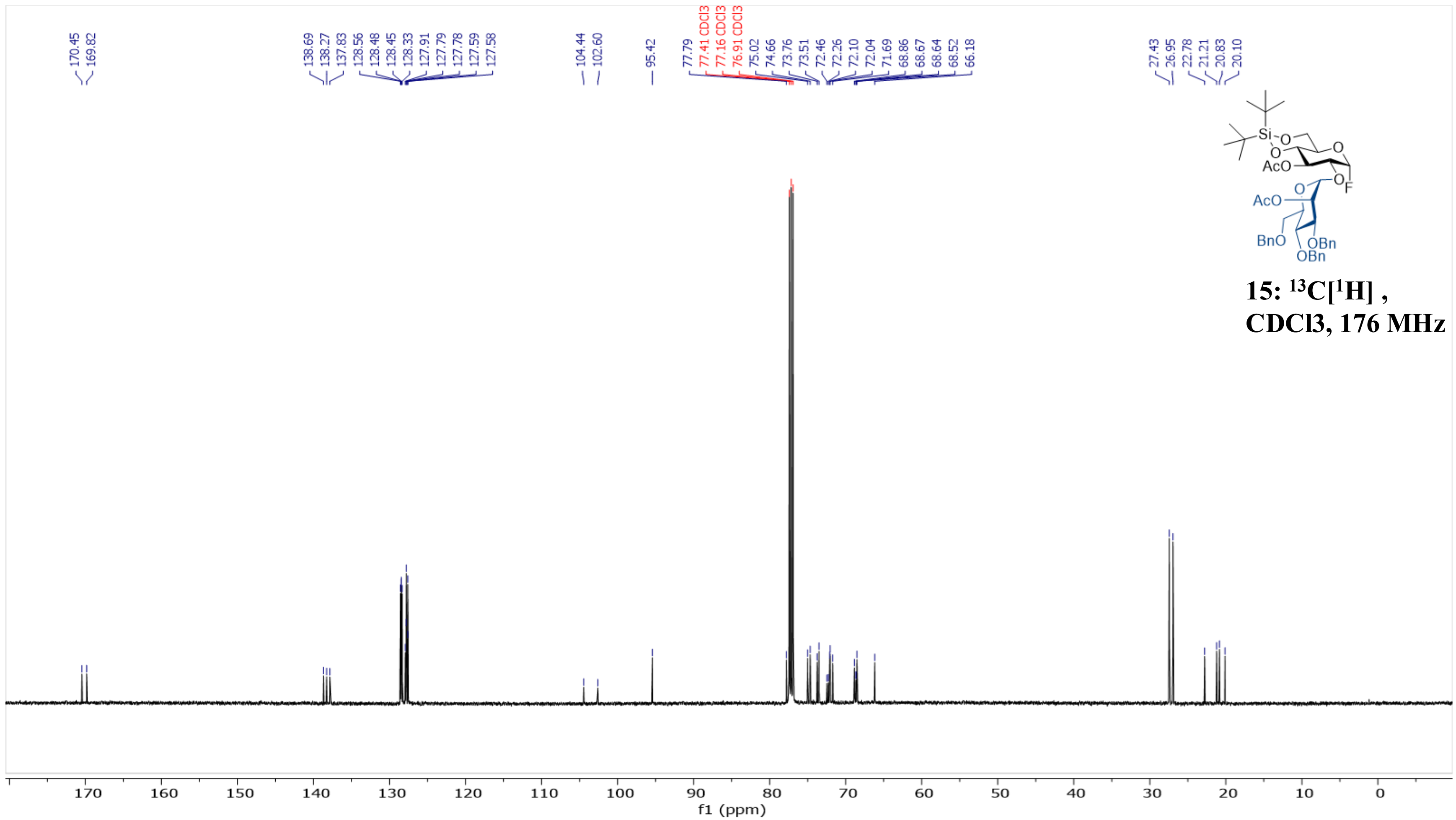

15: $^{13}C[^{1}H]$, CDCl3, 176 MHz

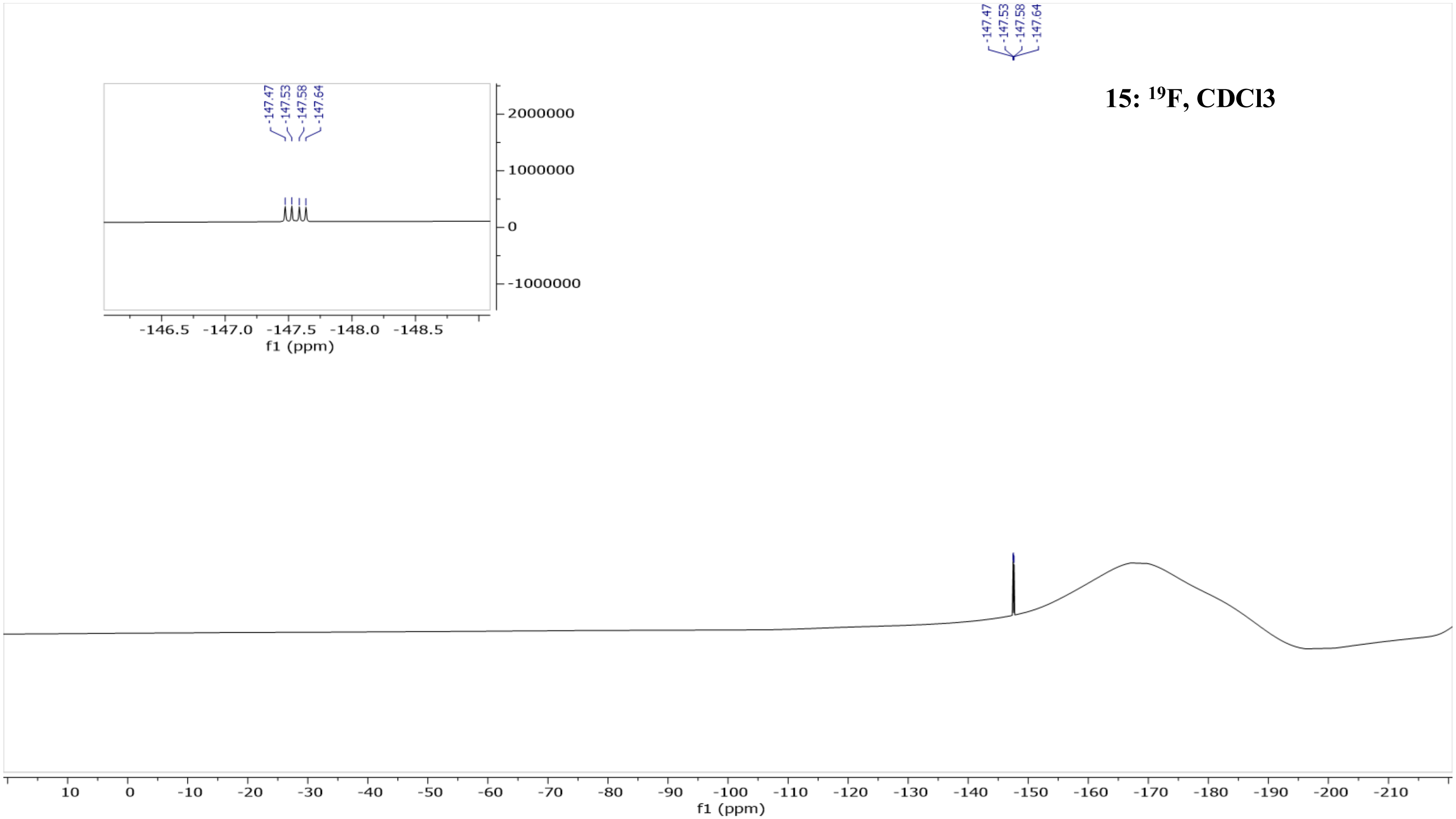
-147.47
-147.53
-147.58
-147.64
15: ¹⁹F, CDCl3
2000000
1000000
0
-1000000
-146.5 -147.0 -147.5 -148.0 -148.5
f1 (ppm)
10 0 -10 -20 -30 -40 -50 -60 -70 -80 -90 -100 -110 -120 -130 -140 -150 -160 -170 -180 -190 -200 -210
f1 (ppm)

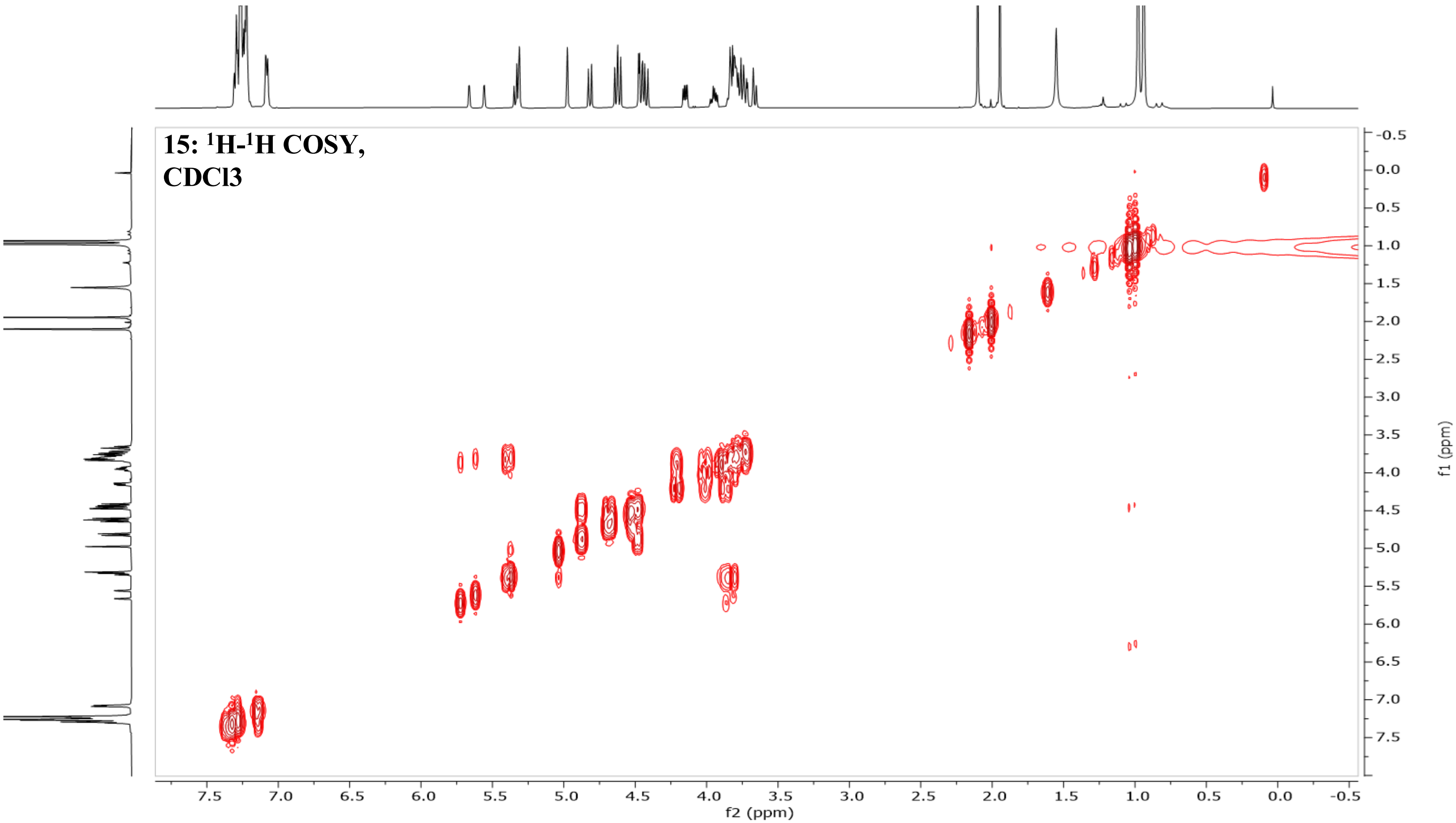
15: ¹H-¹H COSY,
CDCl3
f2 (ppm)
f1 (ppm)

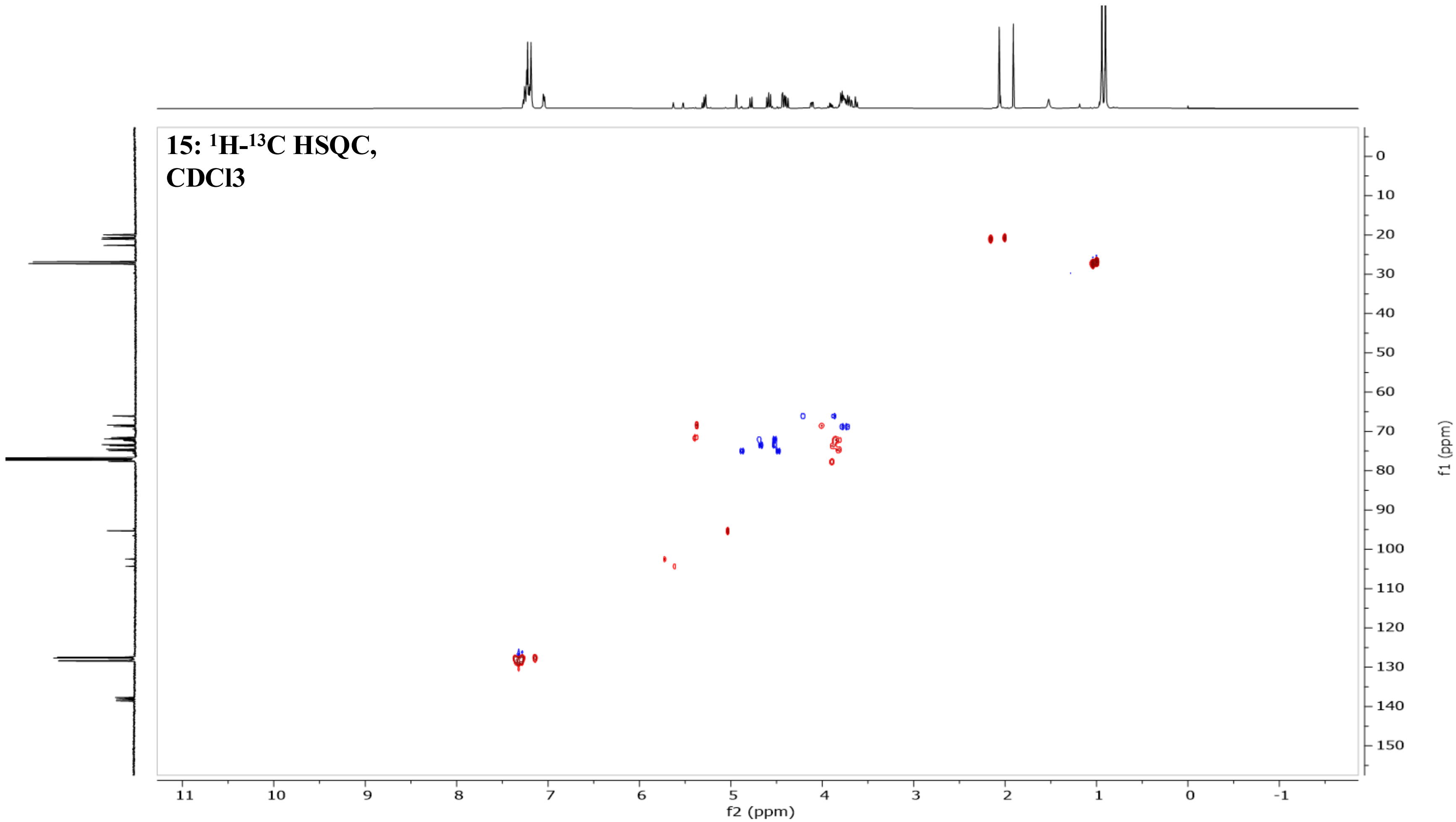
15: 1H-13C HSQC,
CDCl3
f2 (ppm)
f1 (ppm)
11 10 9 8 7 6 5 4 3 2 1 0 -1
0 10 20 30 40 50 60 70 80 90 100 110 120 130 140 150

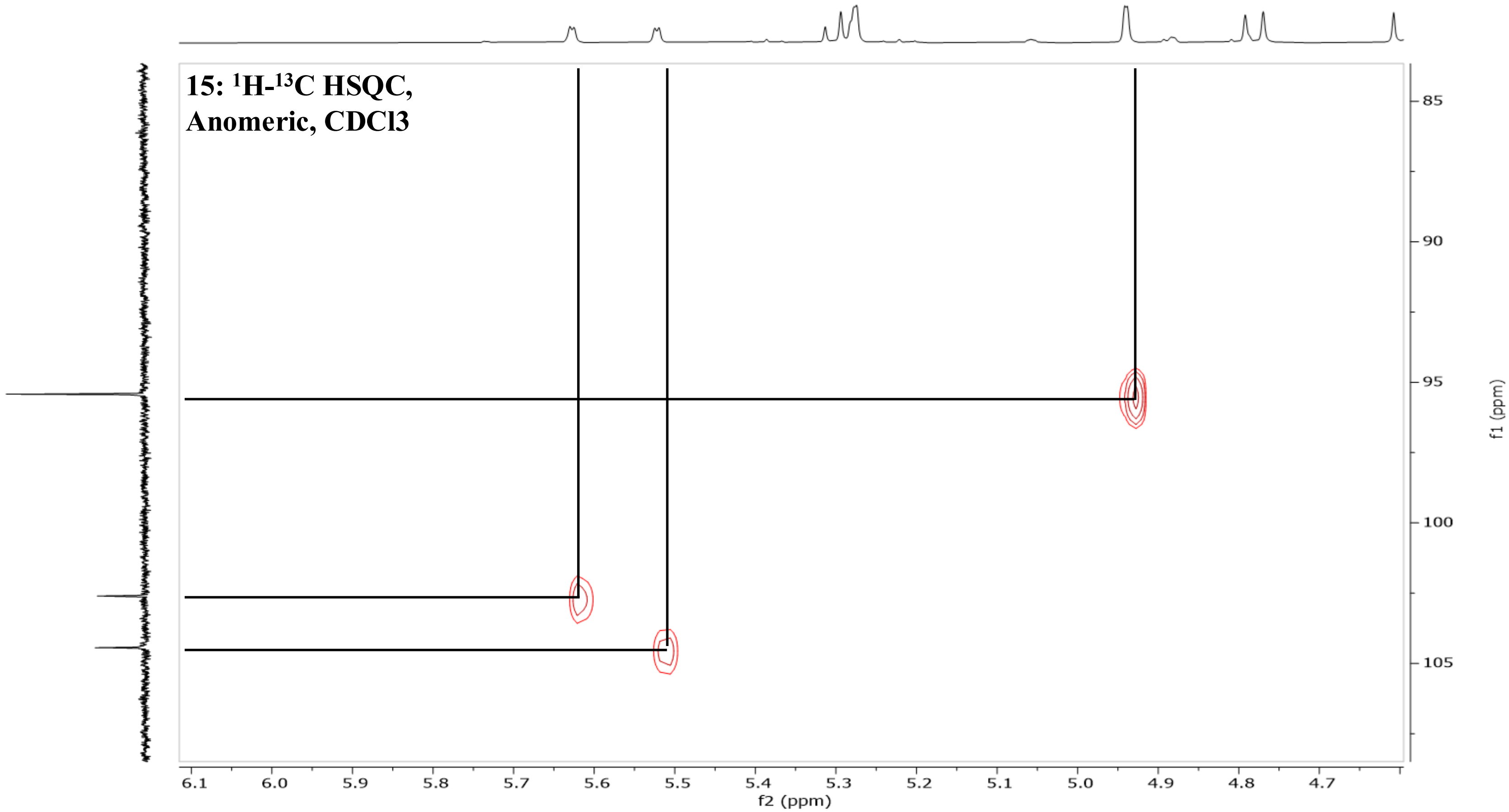
15: ¹H-¹³C HSQC,
Anomeric, CDCl3
85
90
95
100
105
f1 (ppm)
6.1
6.0
5.9
5.8
5.7
5.6
5.5
5.4
5.3
5.2
5.1
5.0
4.9
4.8
4.7
f2 (ppm)

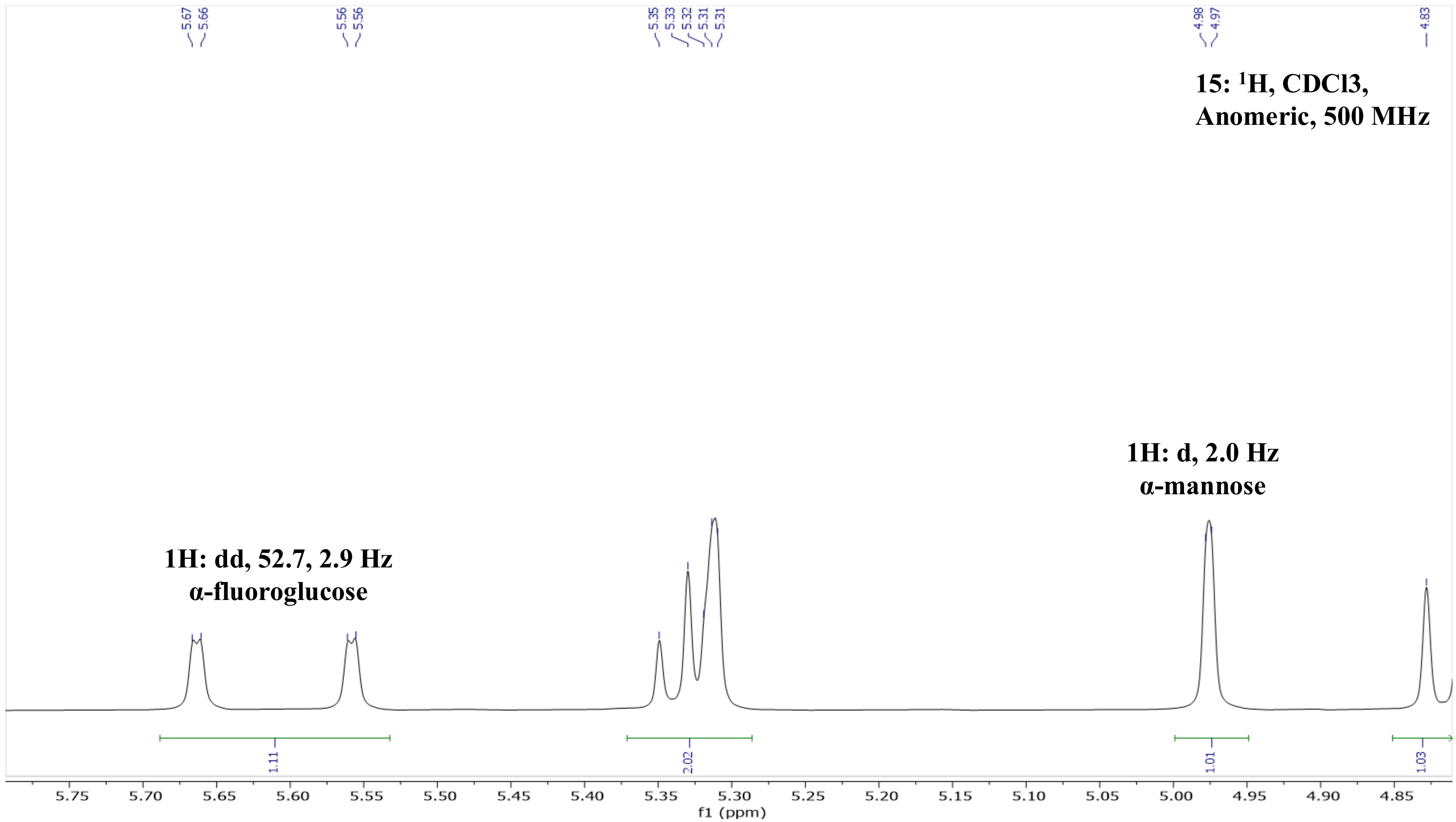
5.67
5.66
5.56
5.56
5.35
5.33
5.32
5.31
5.31
4.98
4.97
4.83
15: ¹H, CDCl3,
Anomeric, 500 MHz
1H: dd, 52.7, 2.9 Hz
α-fluoroglucose
1H: d, 2.0 Hz
α-mannose
1.11
2.02
1.01
1.03
5.75
5.70
5.65
5.60
5.55
5.50
5.45
5.40
5.35
5.30
5.25
5.20
5.15
5.10
5.05
5.00
4.95
4.90
4.85
f1 (ppm)

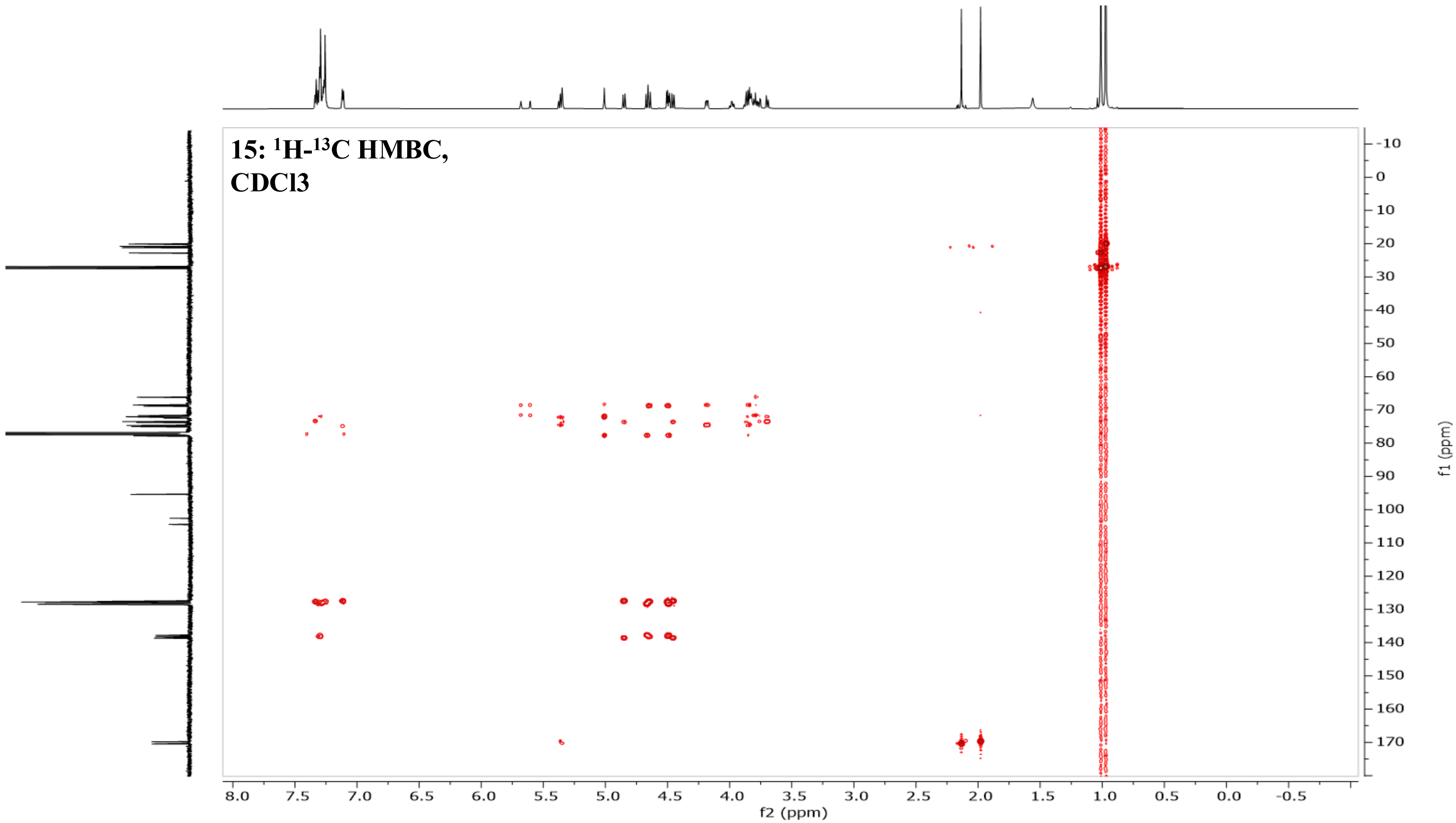
15: ¹H-¹³C HMBC,
CDCl3
f2 (ppm)
f1 (ppm)

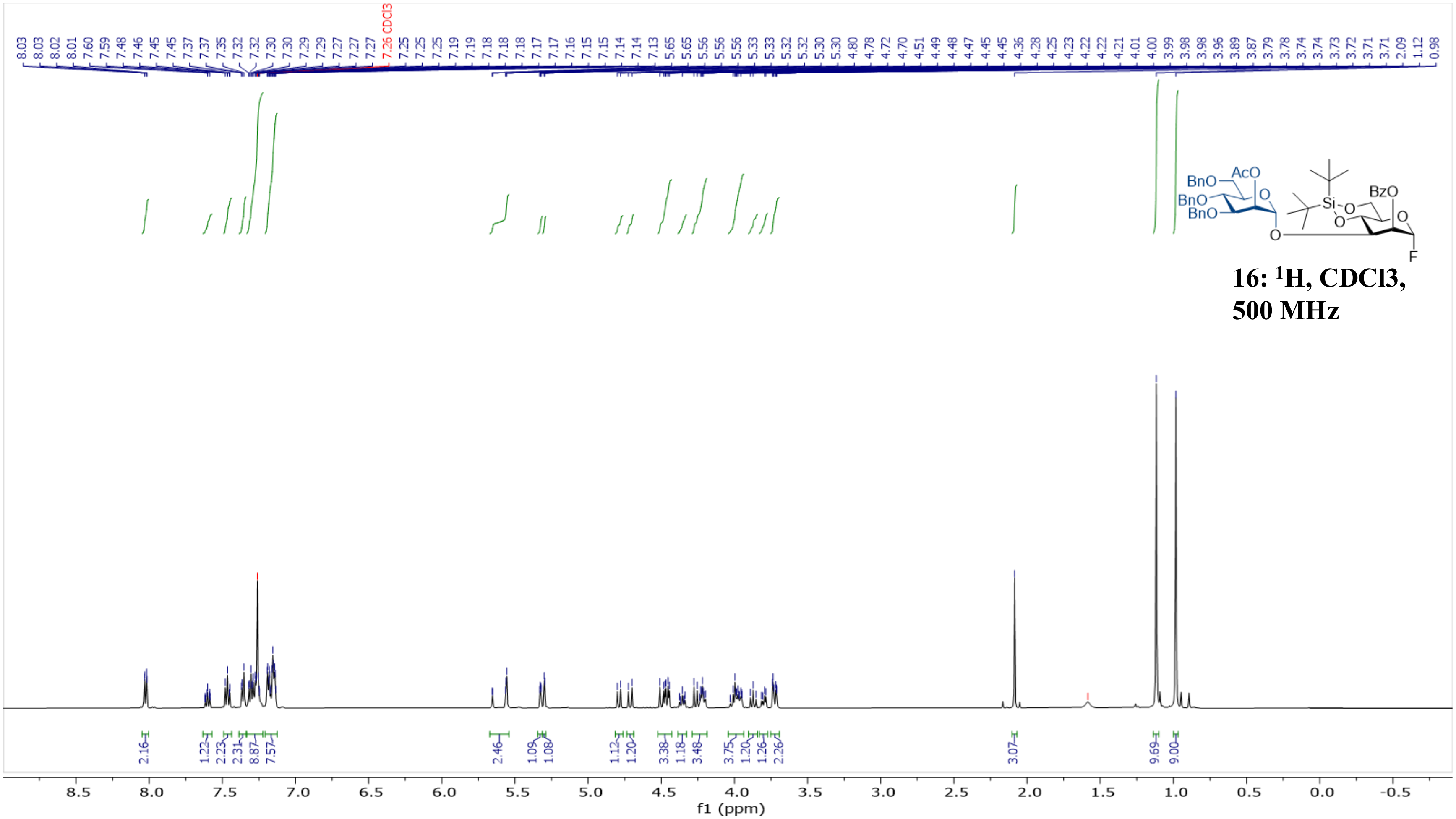
16: ^{1}H, CDCl3, 500 MHz
f1 (ppm)

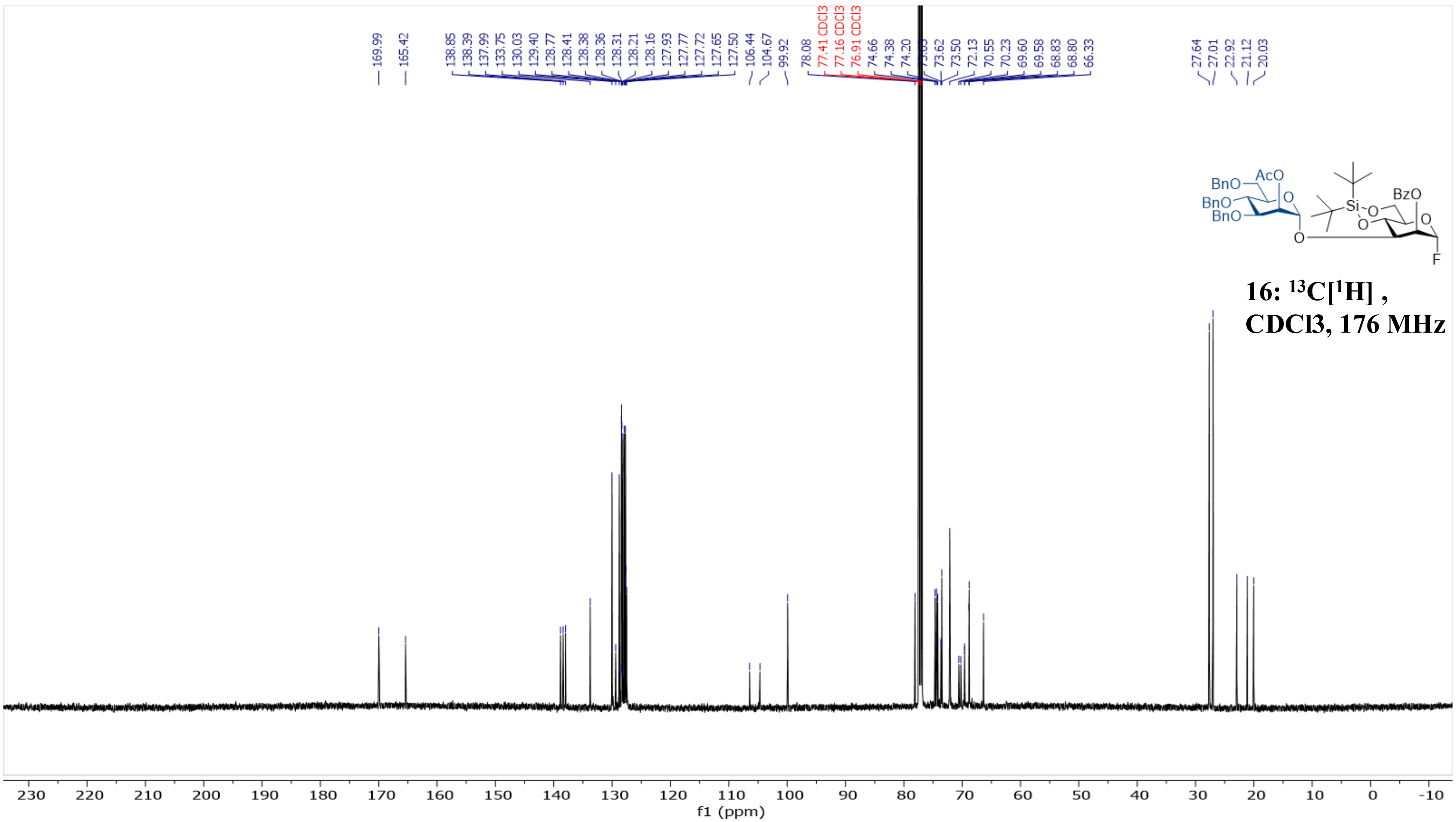
169.99
165.42
138.85
138.39
137.99
133.75
130.03
129.40
128.77
128.41
128.38
128.36
128.31
128.21
128.16
127.93
127.77
127.72
127.65
127.50
106.44
104.67
99.92
78.08
77.41 CDCl3
77.16 CDCl3
76.91 CDCl3
74.66
74.38
74.20
73.62
73.50
72.13
70.55
70.23
69.60
69.58
68.83
68.80
66.33
27.64
27.01
22.92
21.12
20.03
BnO
AcO
BnO
BnO
O
O
Si
O
O
BzO
O
F
16: 13C[1H] ,
CDCl3, 176 MHz
230 220 210 200 190 180 170 160 150 140 130 120 110 100 90 80 70 60 50 40 30 20 10 0 -10
f1 (ppm)

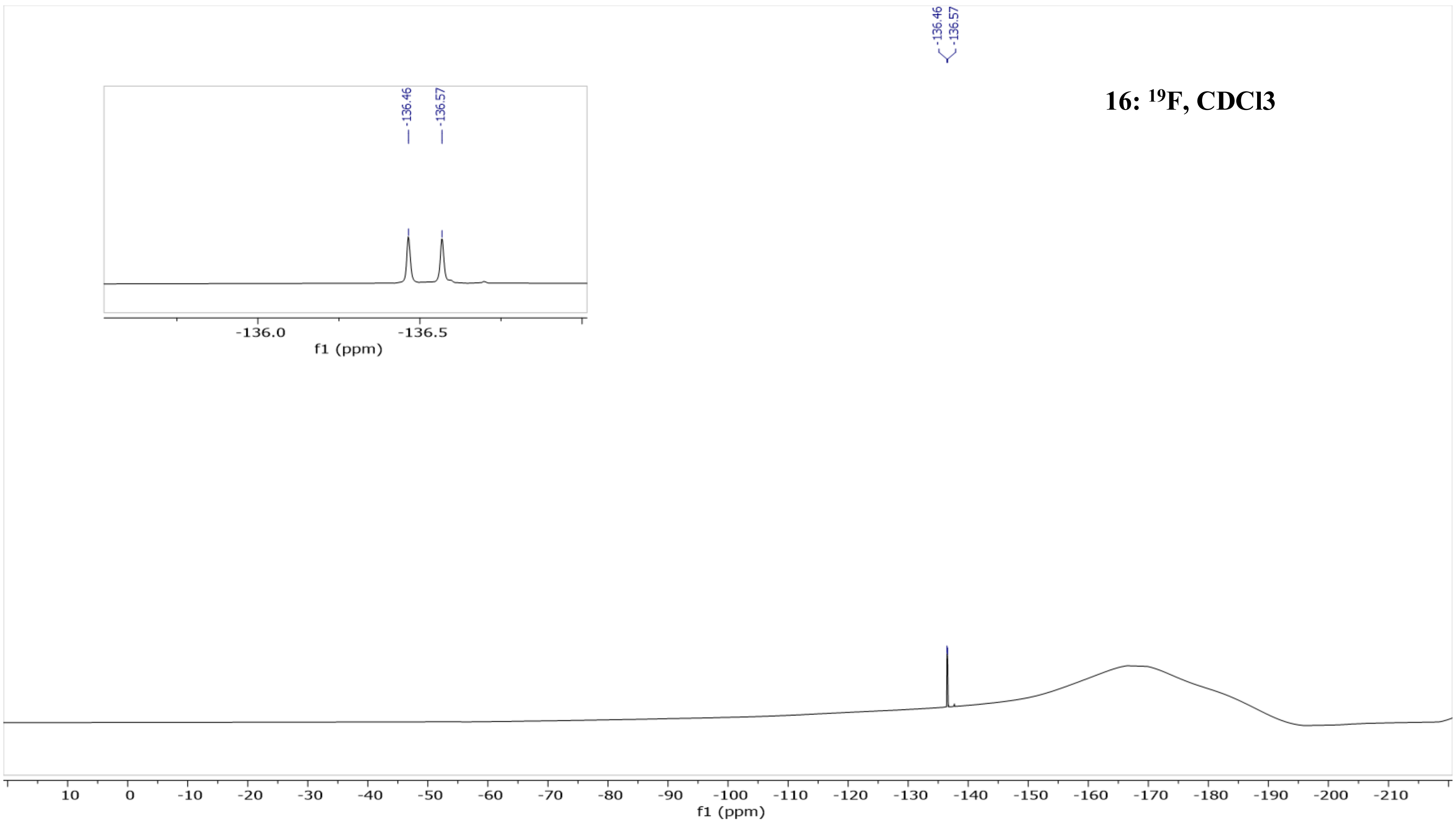
-136.46
-136.57
16: 19F, CDCl3
-136.0
-136.5
f1 (ppm)
10 0 -10 -20 -30 -40 -50 -60 -70 -80 -90 -100 -110 -120 -130 -140 -150 -160 -170 -180 -190 -200 -210
f1 (ppm)

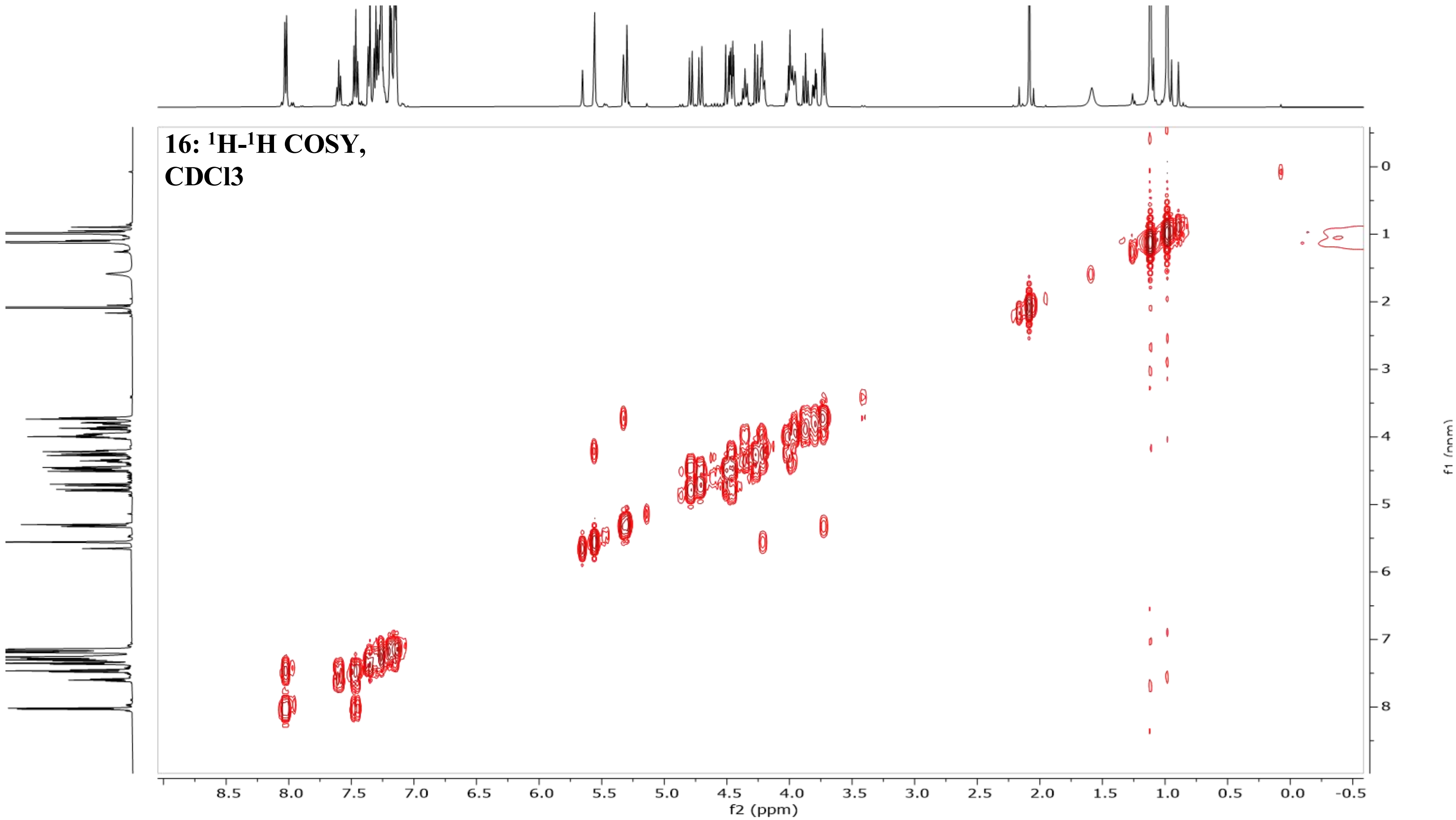
16: 1H-1H COSY,
CDCl3
f2 (ppm)
f1 (ppm)

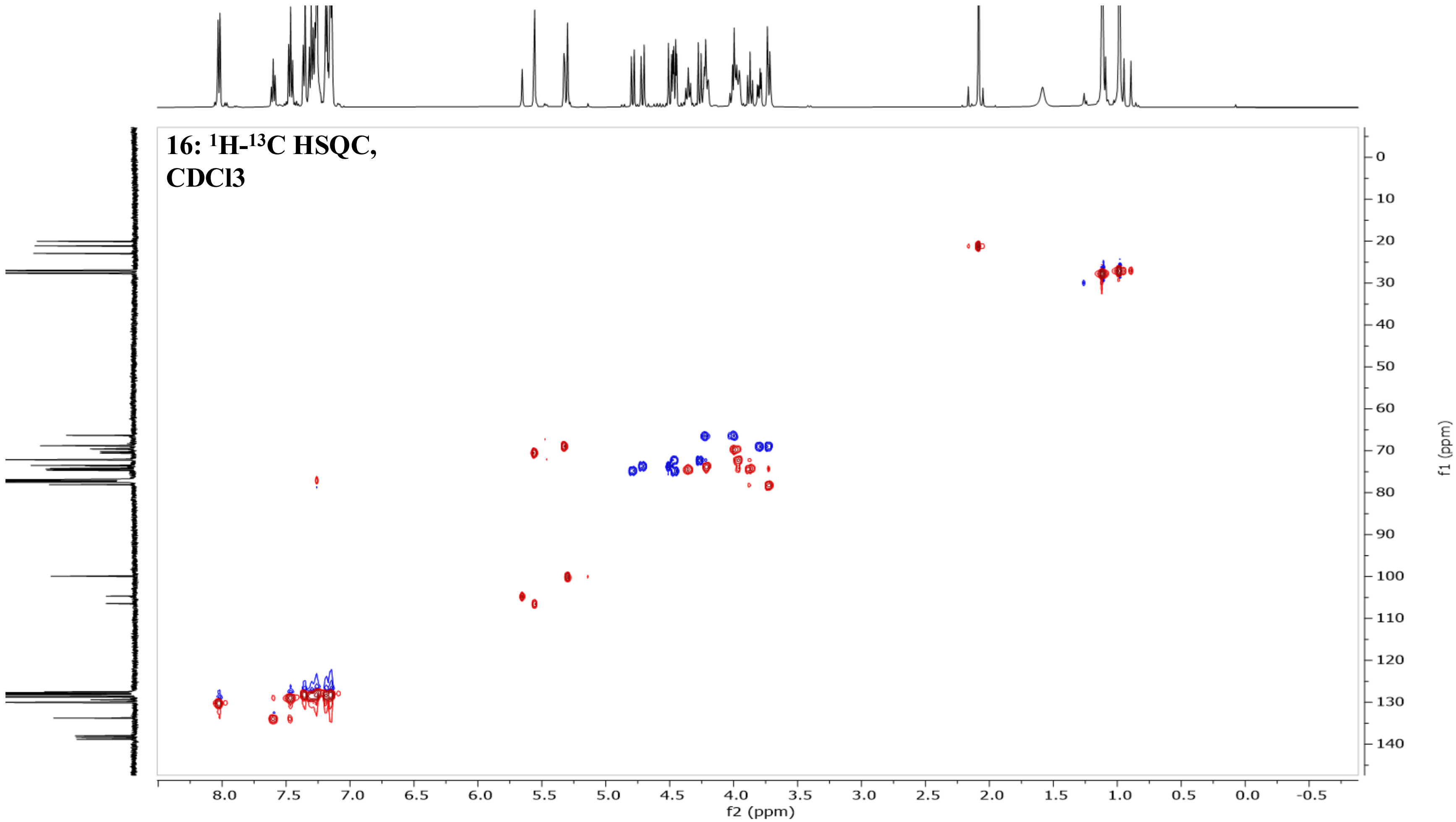
16: 1H-13C HSQC,
CDCl3
f2 (ppm)
f1 (ppm)

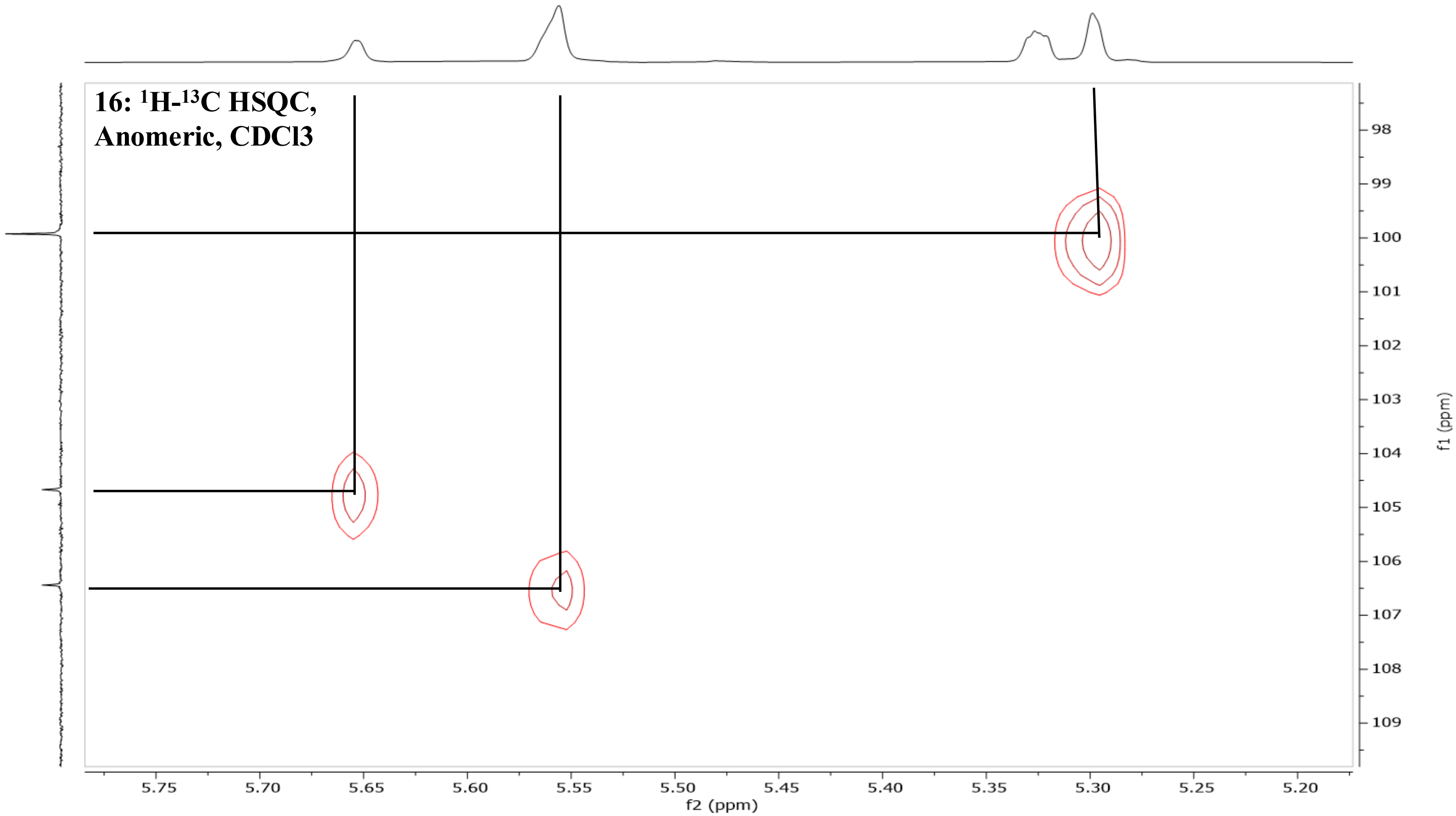

16: ^{1}H-^{13}C HSQC,
Anomeric, CDCl3
5.75
5.70
5.65
5.60
5.55
5.50
5.45
5.40
5.35
5.30
5.25
5.20
f2 (ppm)
98
99
100
101
102
103
104
105
106
107
108
109
f1 (ppm)

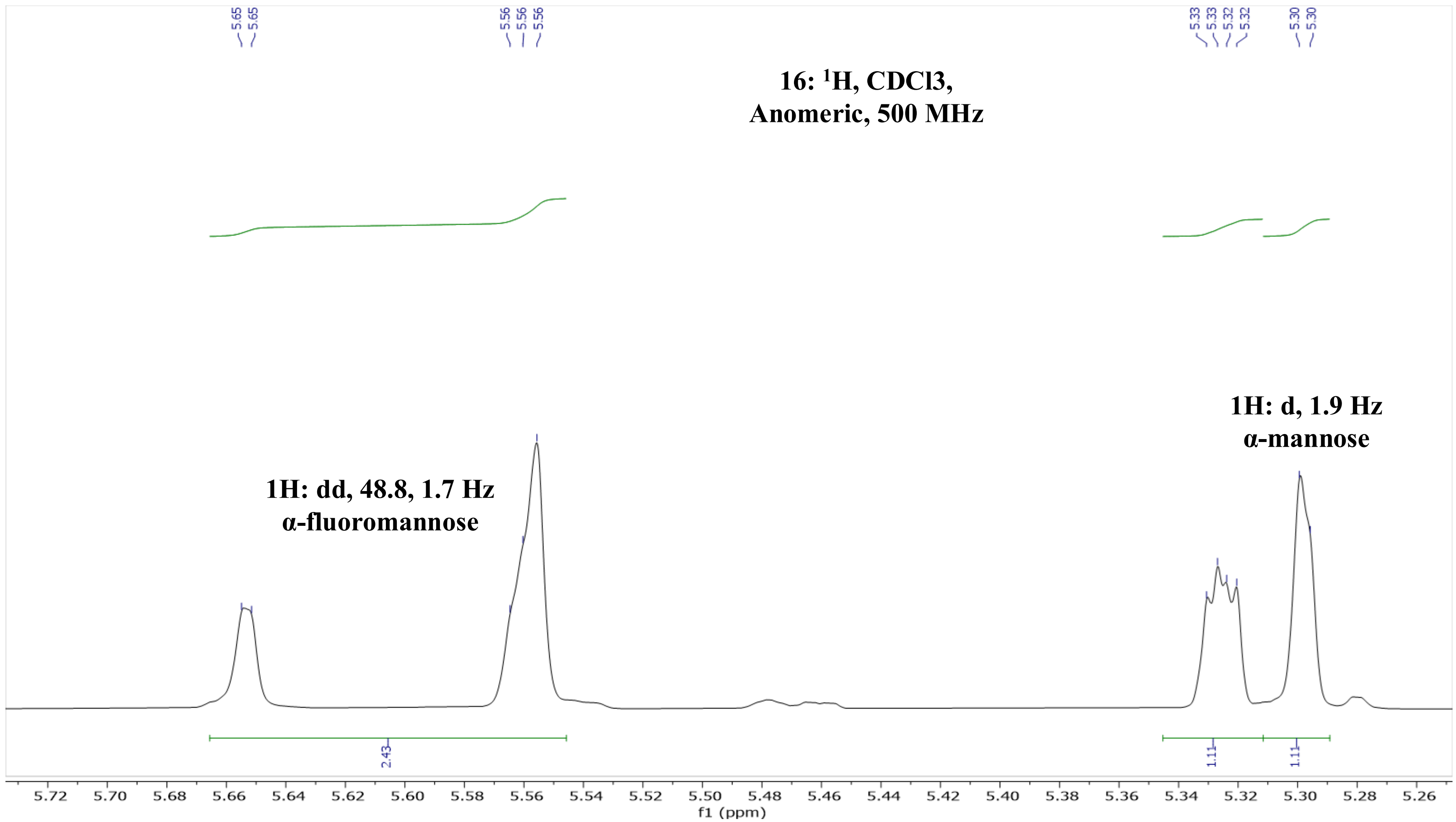
16: 1H, CDCl3,
Anomeric, 500 MHz
1H: dd, 48.8, 1.7 Hz
α-fluoromannose
1H: d, 1.9 Hz
α-mannose
5.65
5.65
5.56
5.56
5.56
5.33
5.33
5.32
5.32
5.30
5.30
2.43
1.11
1.11
5.72
5.70
5.68
5.66
5.64
5.62
5.60
5.58
5.56
5.54
5.52
5.50
5.48
5.46
5.44
5.42
5.40
5.38
5.36
5.34
5.32
5.30
5.28
5.26
f1 (ppm)

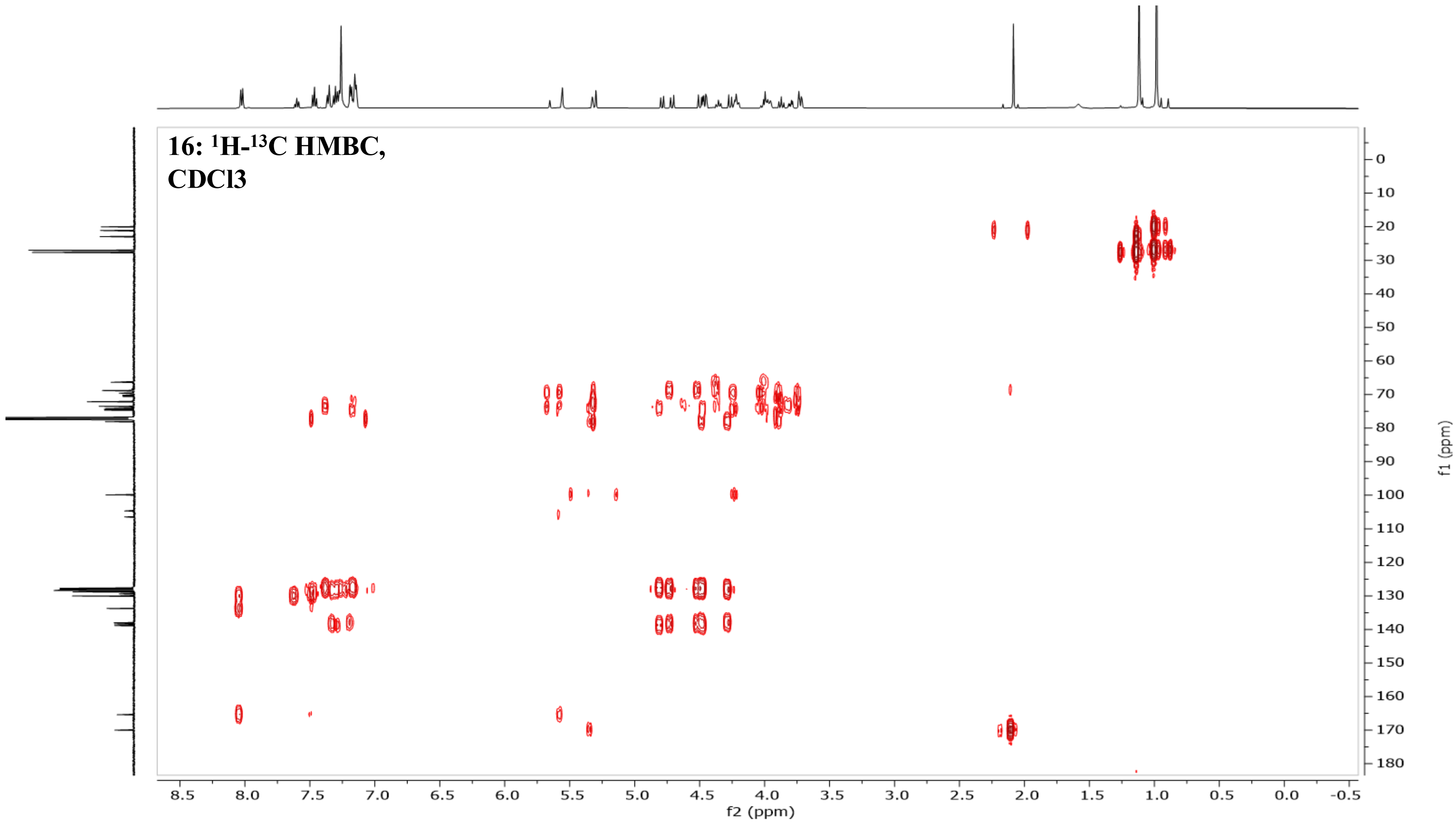

16: ¹H-¹³C HMBC,
CDCl3
f2 (ppm)
f1 (ppm)

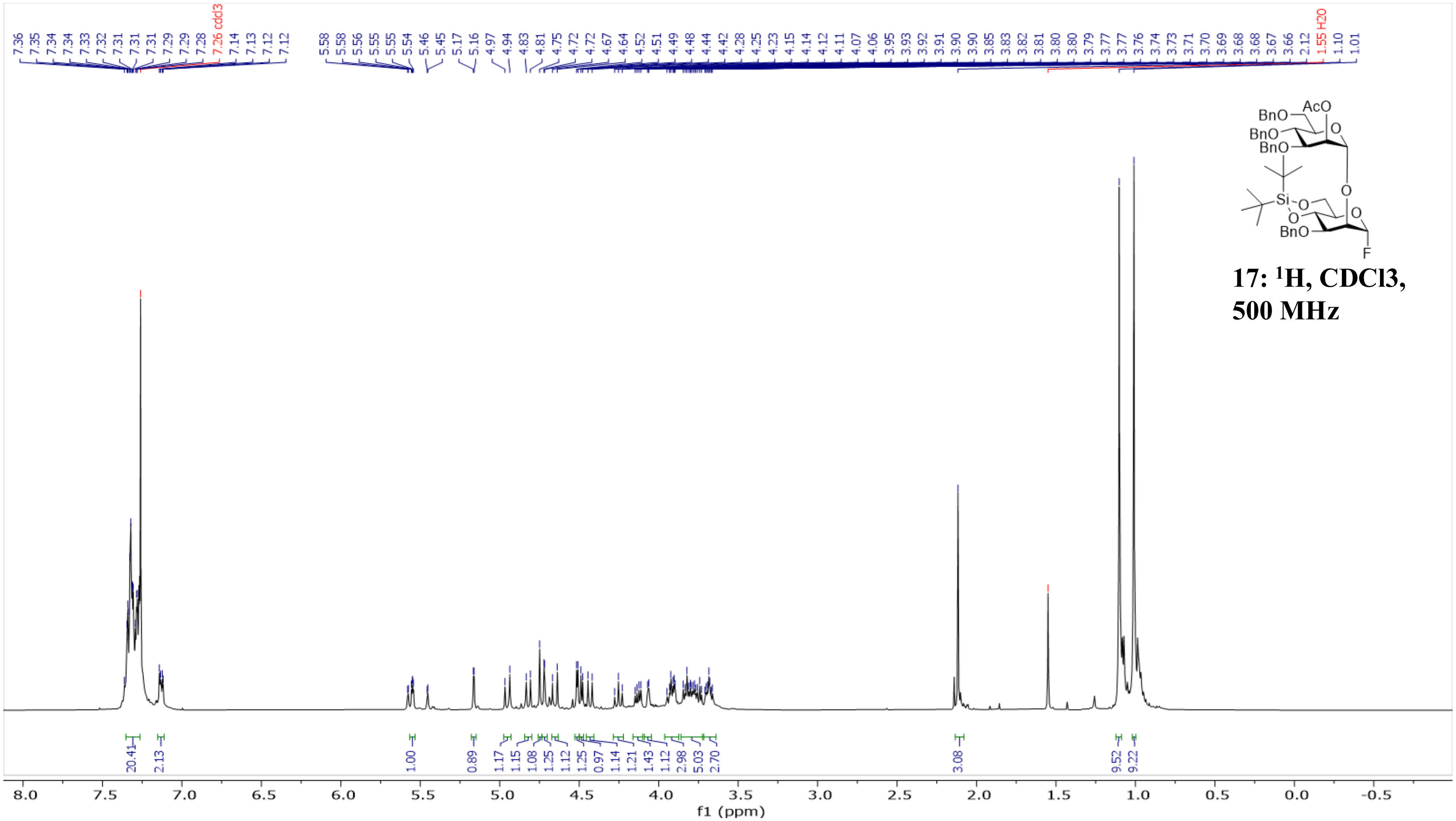

17: ¹H, CDCl3, 500 MHz
f1 (ppm)

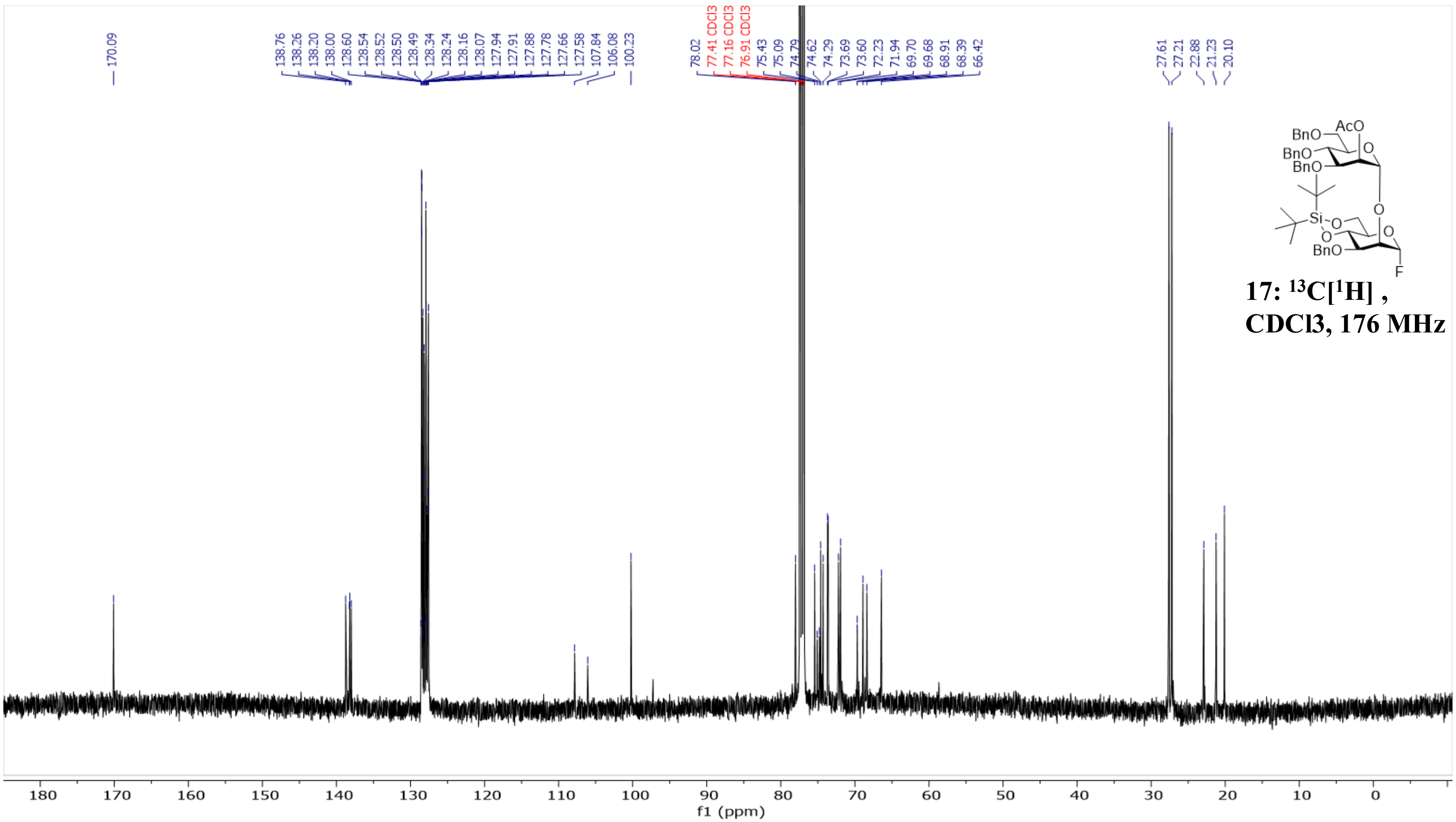

17: $^{13}C[^{1}H]$, CDCl3, 176 MHz

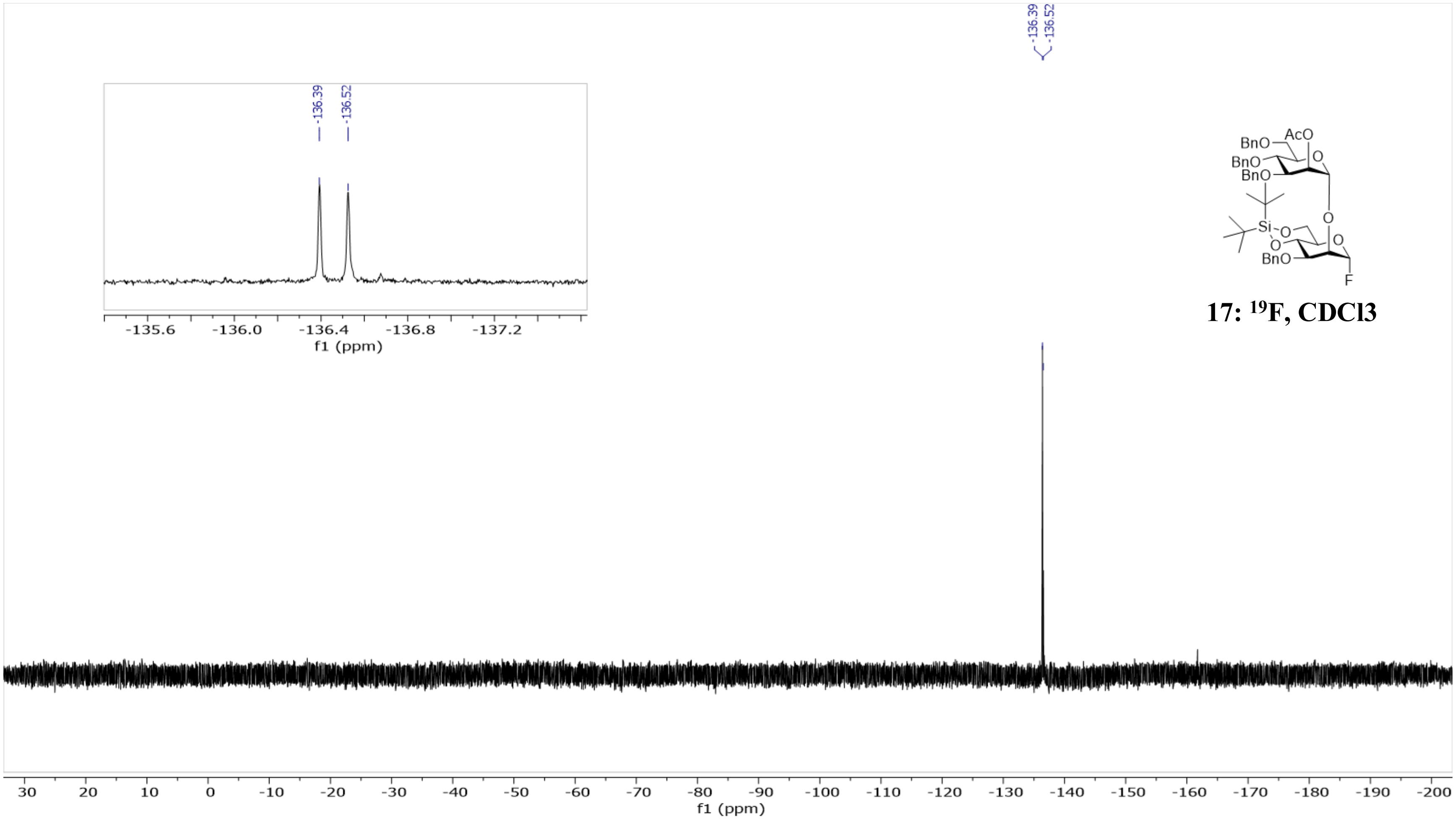
-136.39
-136.52
BnO
AcO
O
BnO
BnO
Si
O
O
O
O
BnO
F
-135.6
-136.0
-136.4
-136.8
-137.2
f1 (ppm)
30
20
10
0
-10
-20
-30
-40
-50
-60
-70
-80
-90
-100
-110
-120
-130
-140
-150
-160
-170
-180
-190
-200
f1 (ppm)

17: ^{19}F, CDCl3

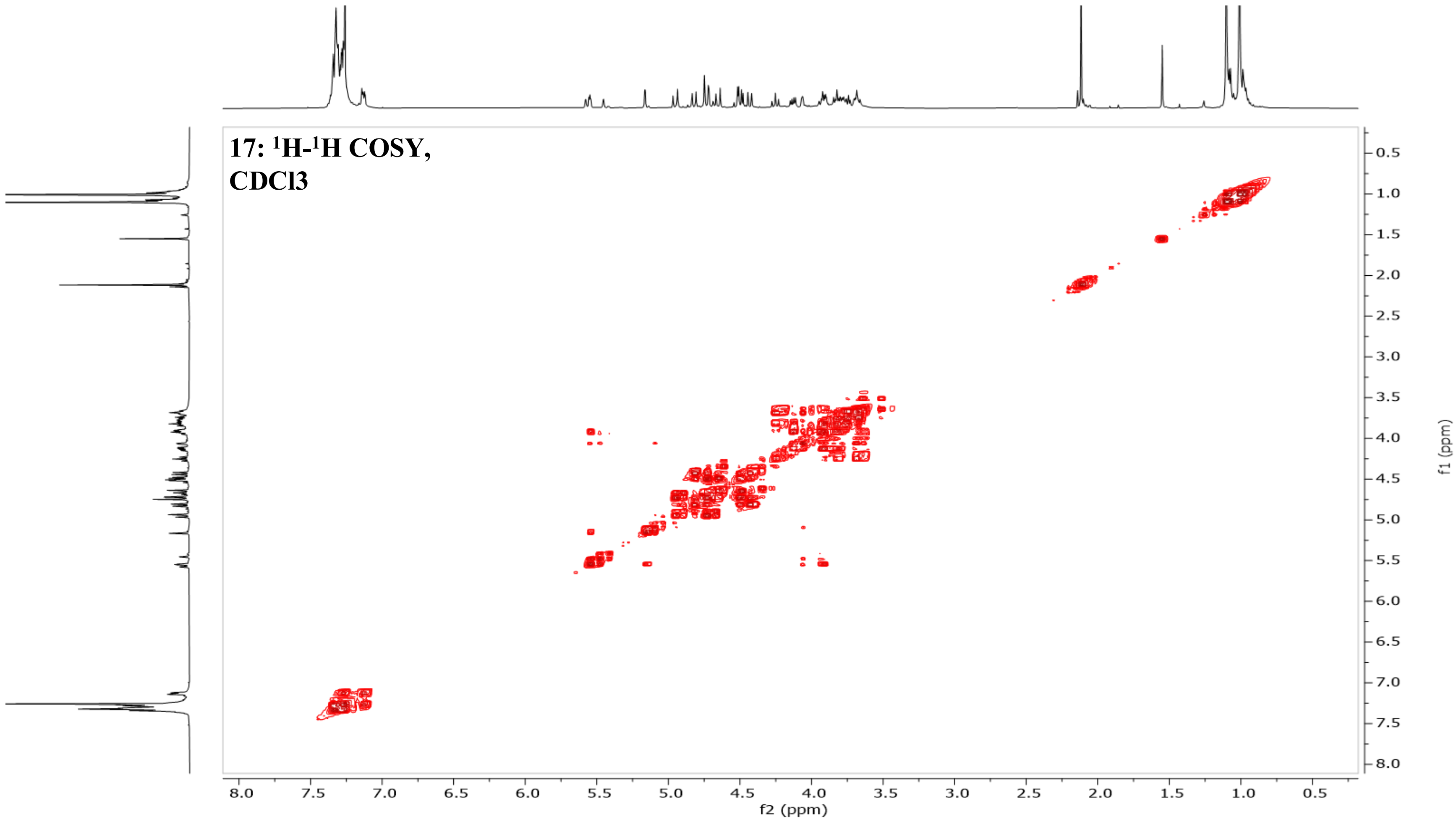

17: 1H-1H COSY,
CDCl3
f2 (ppm)
f1 (ppm)
8.0
7.5
7.0
6.5
6.0
5.5
5.0
4.5
4.0
3.5
3.0
2.5
2.0
1.5
1.0
0.5

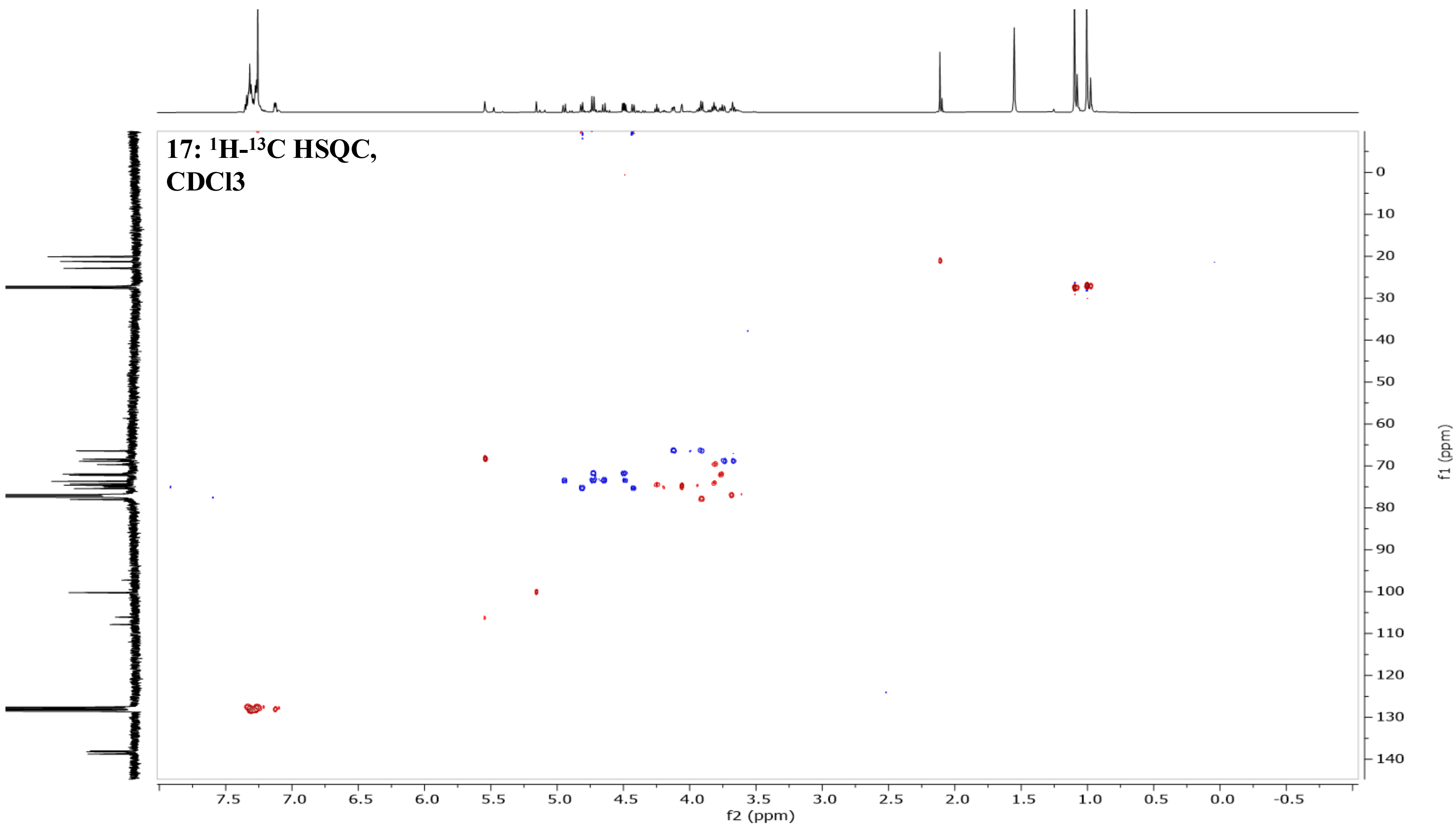

17: ^{1}H-^{13}C HSQC,
CDCl3
f2 (ppm)
f1 (ppm)

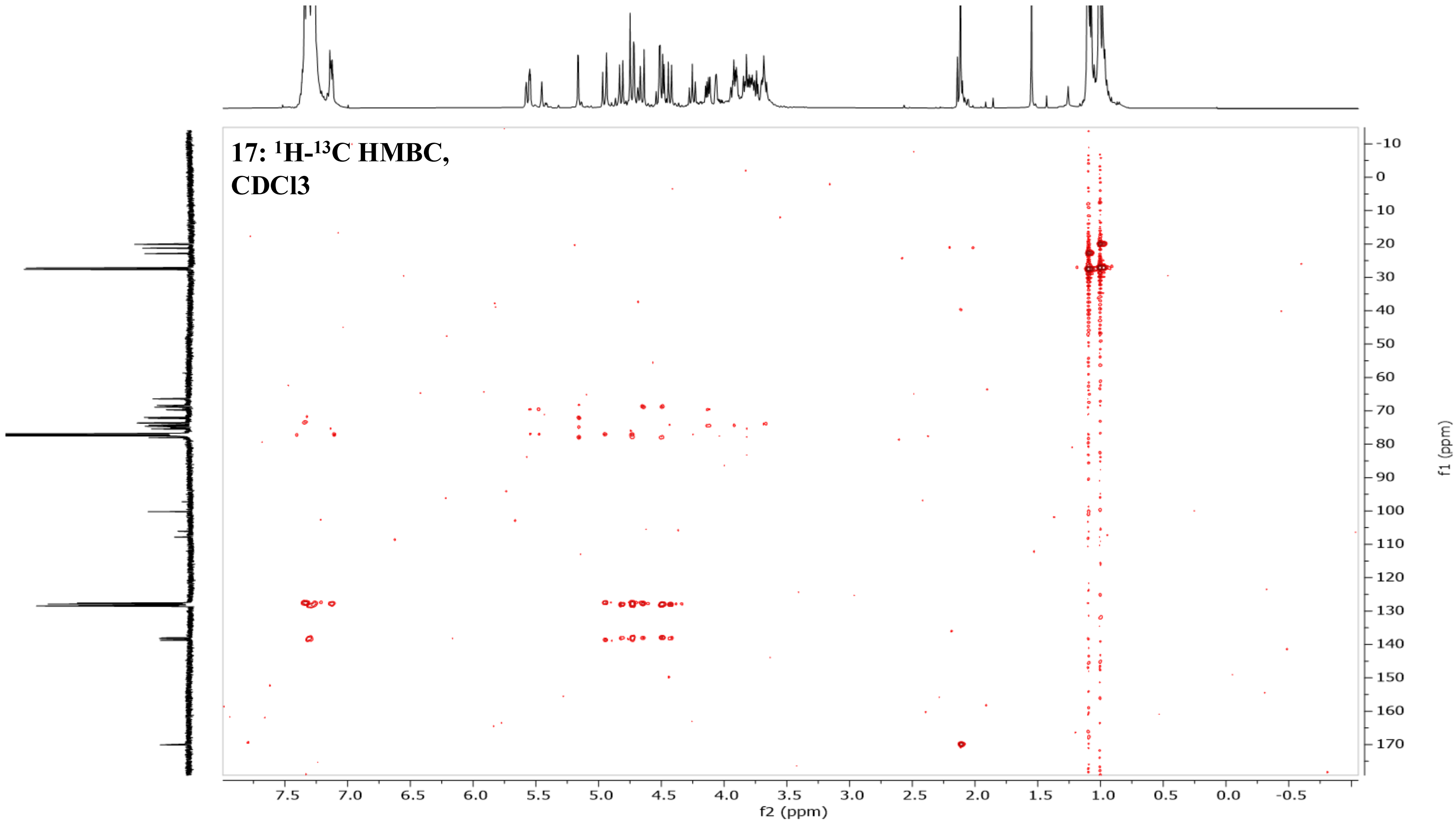

17: ¹H-¹³C HMBC,
CDCl3
f2 (ppm)
f1 (ppm)

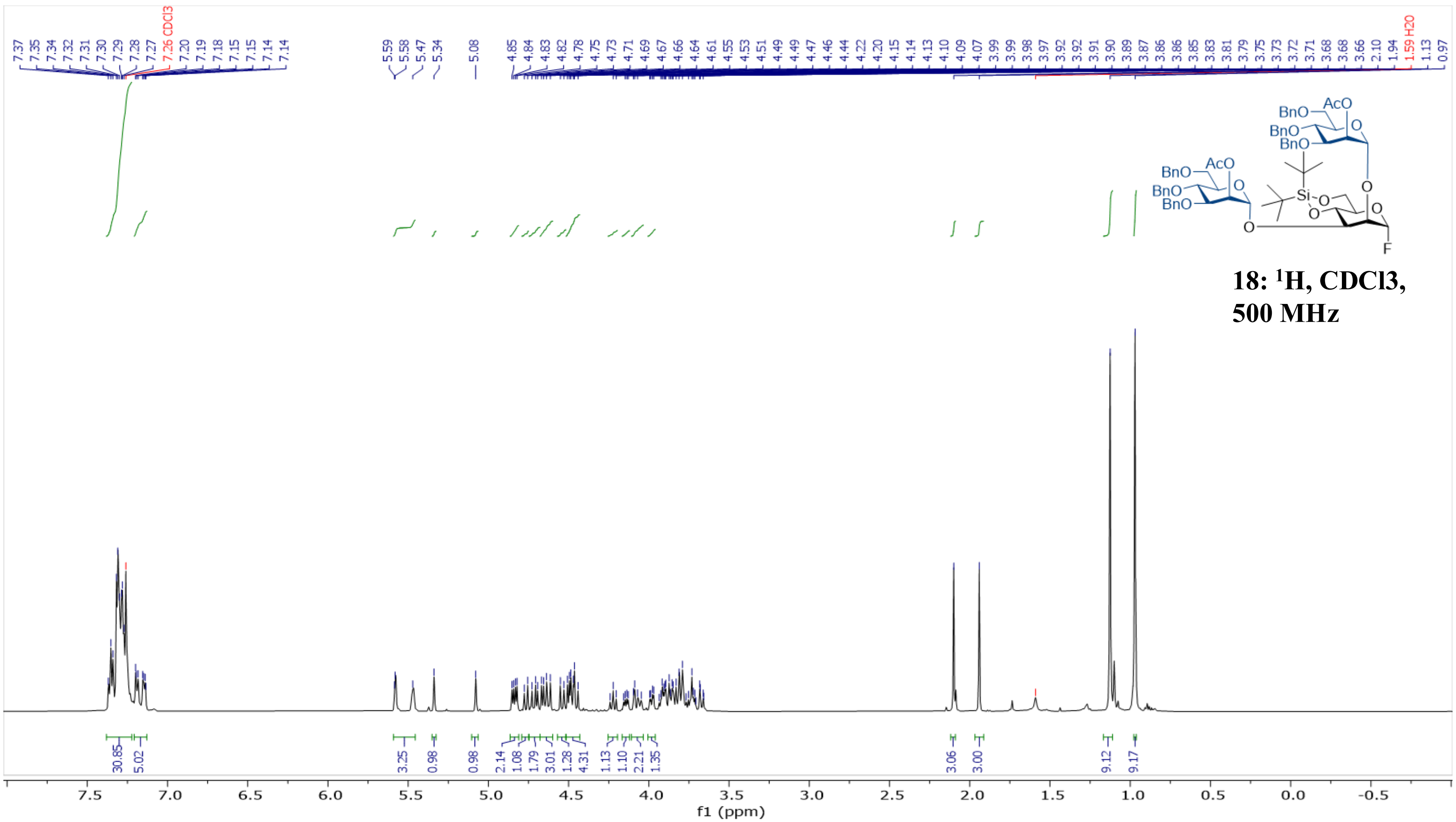
18: ¹H, CDCl3, 500 MHz
f1 (ppm)

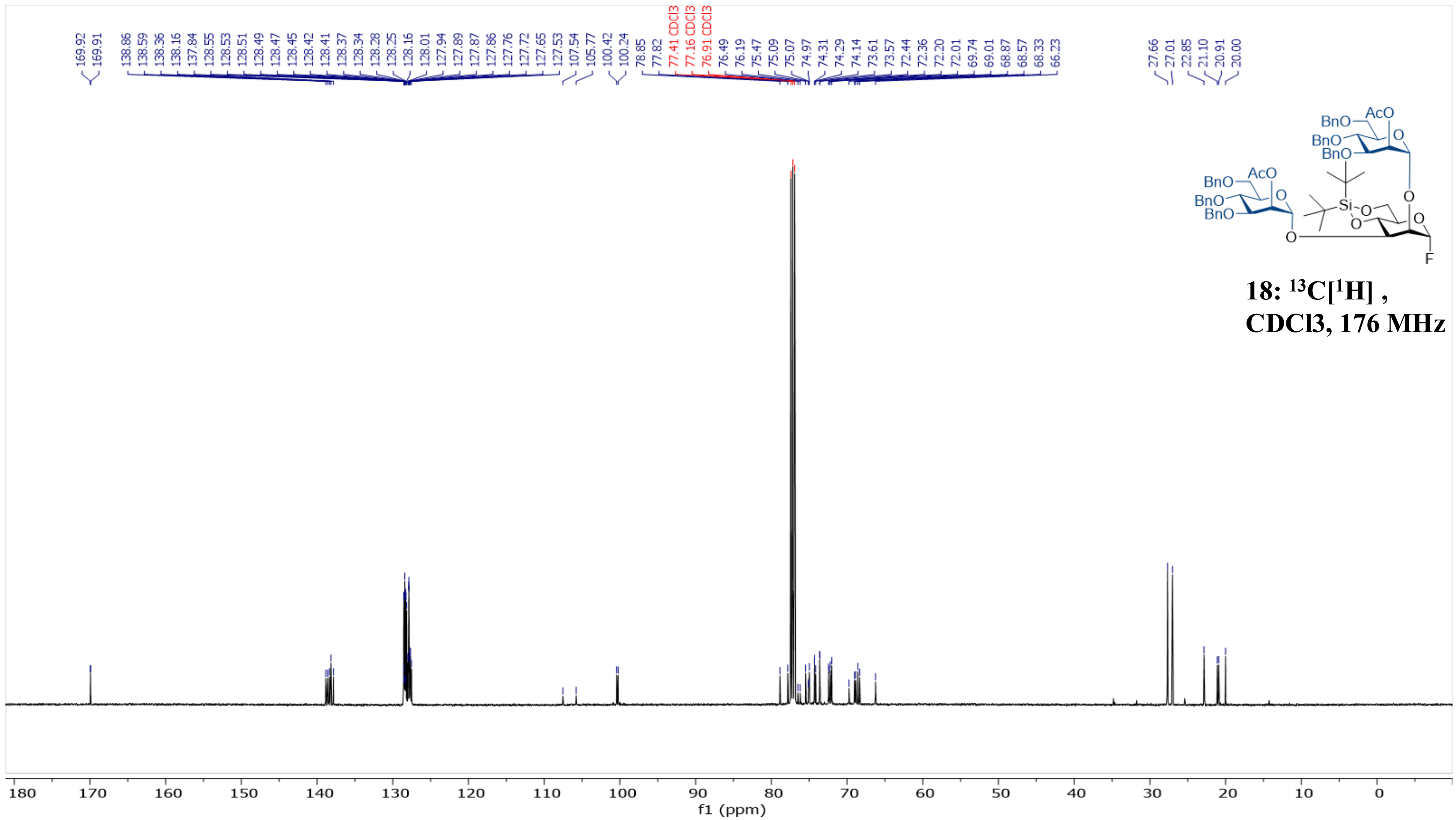

18: 13C[1H] ,
CDCl3, 176 MHz
f1 (ppm)

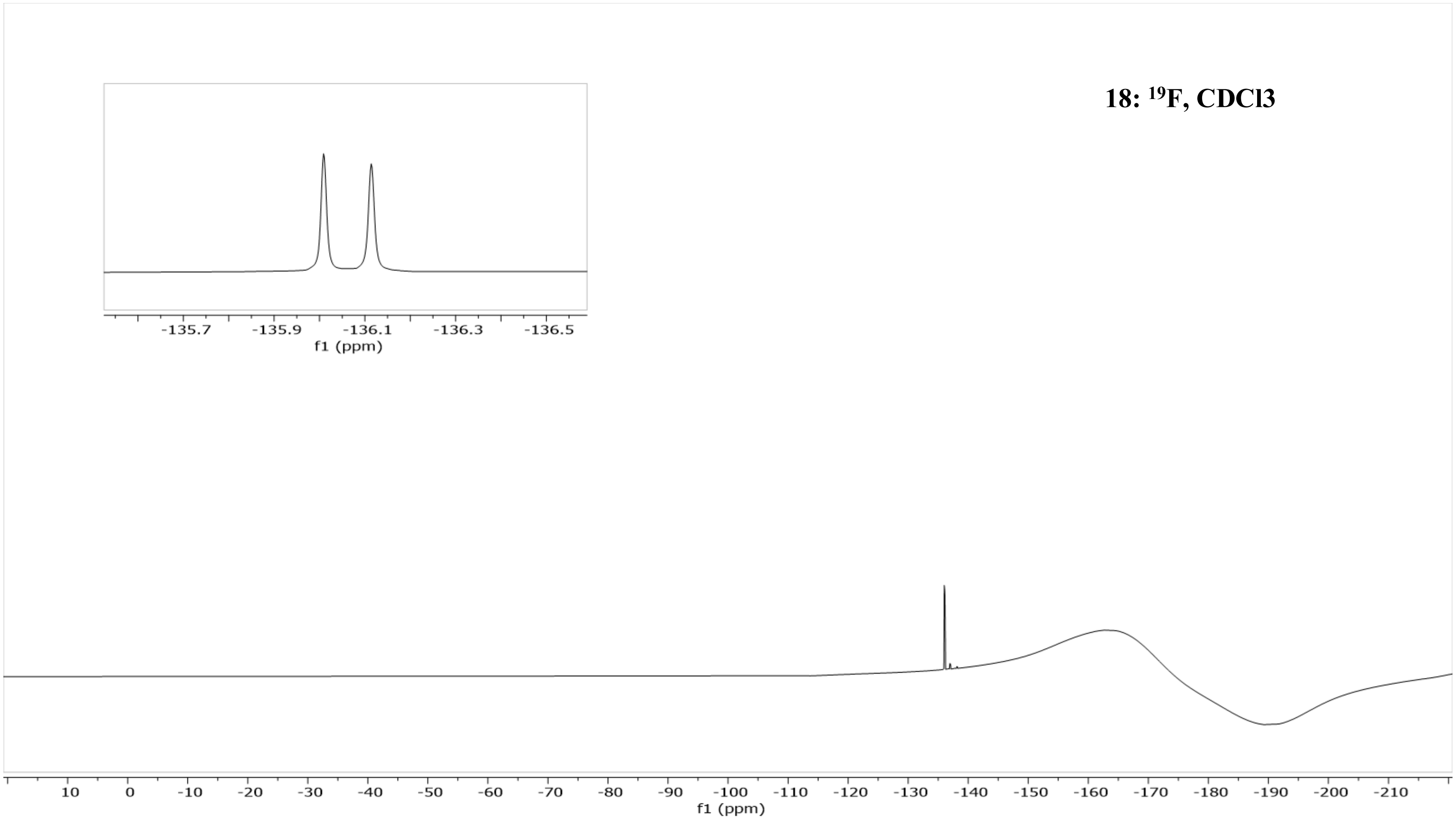

18: ¹⁹F, CDCl3
-135.7
-135.9
-136.1
-136.3
-136.5
f1 (ppm)
10
0
-10
-20
-30
-40
-50
-60
-70
-80
-90
-100
-110
-120
-130
-140
-150
-160
-170
-180
-190
-200
-210
f1 (ppm)

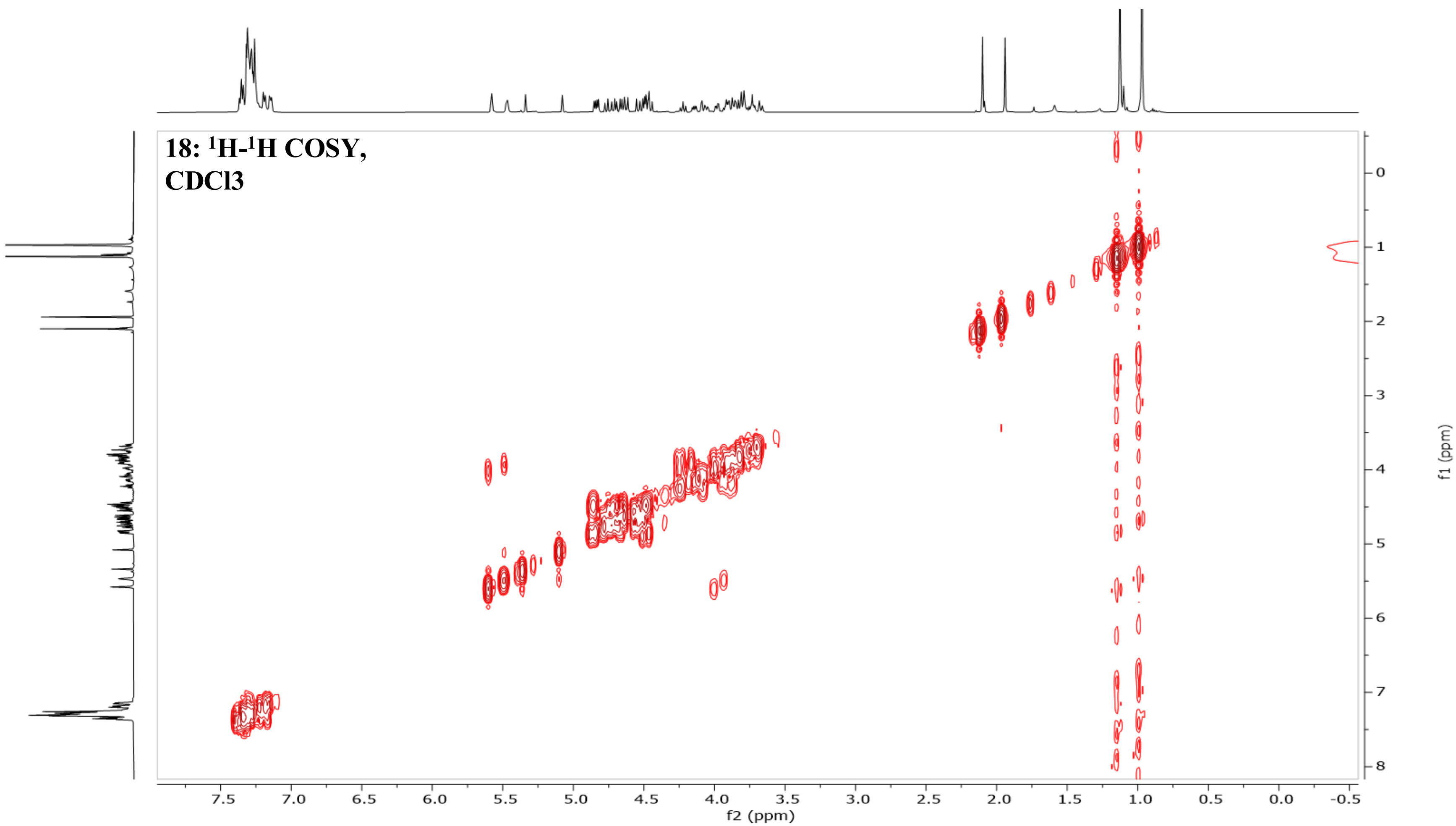
18: ¹H-¹H COSY,
CDCl3
f2 (ppm)
f1 (ppm)

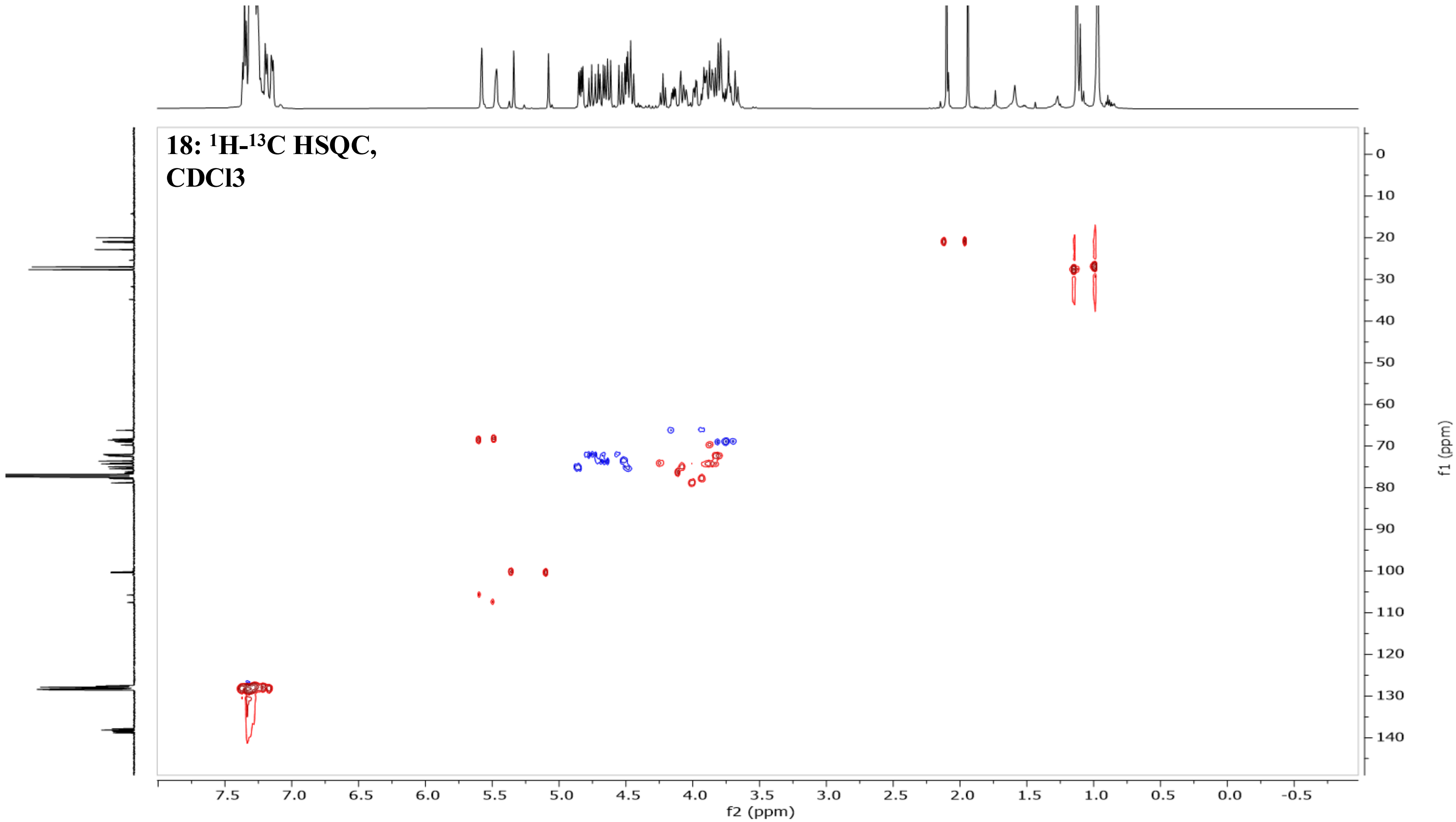
18: 1H-13C HSQC,
CDCl3
f2 (ppm)
f1 (ppm)

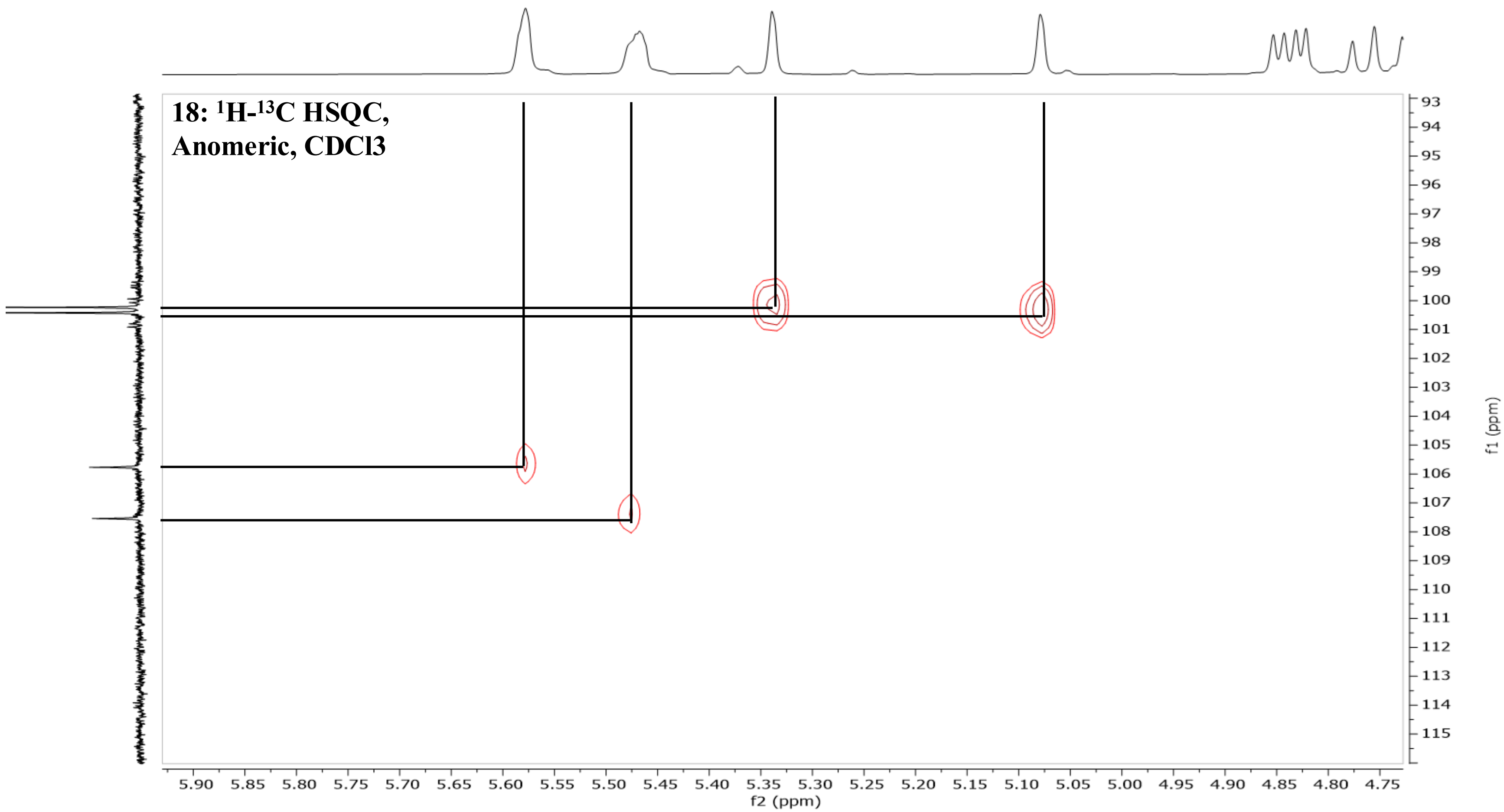
18: 1H-13C HSQC,
Anomeric, CDCl3
f2 (ppm)
f1 (ppm)

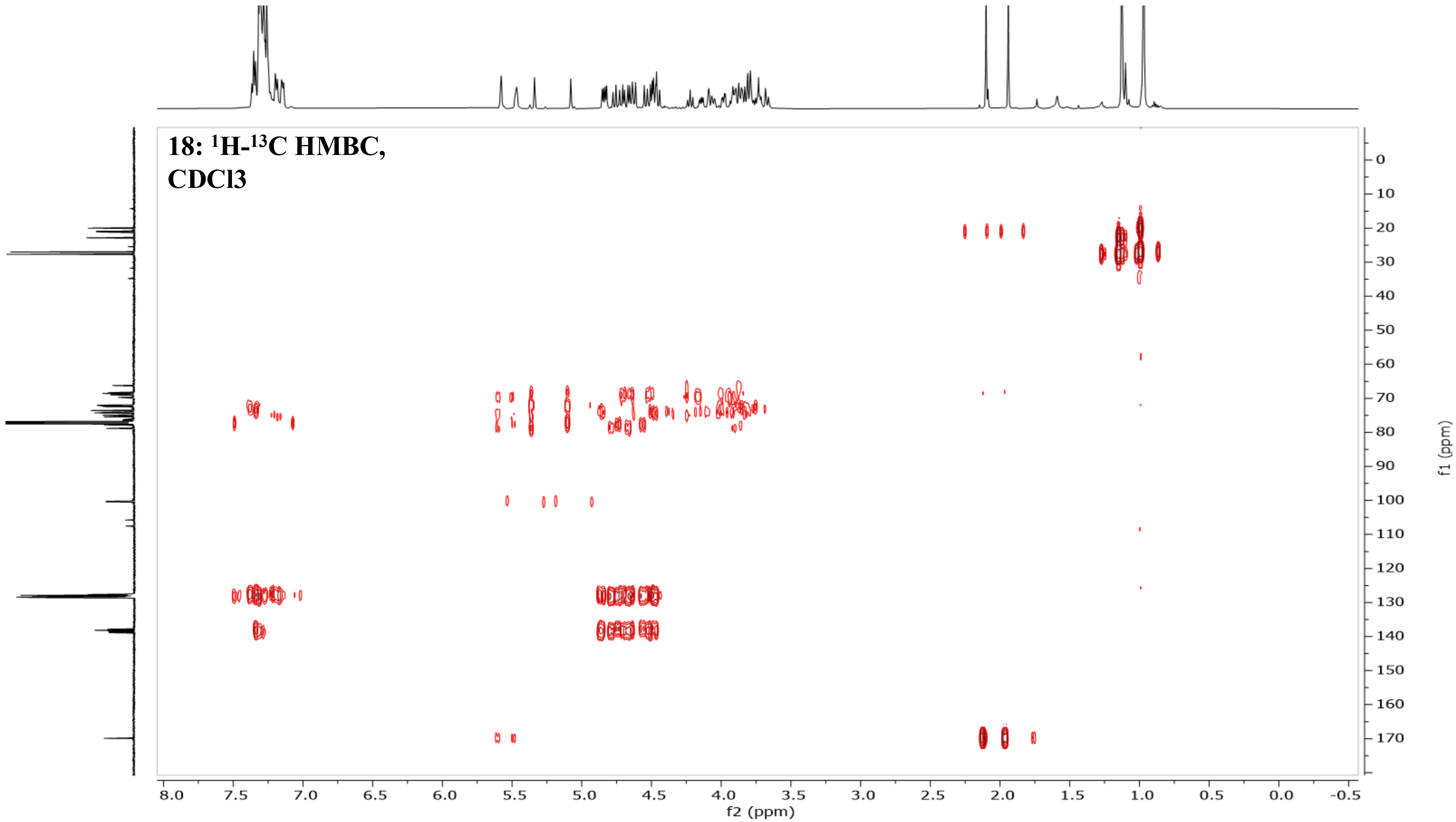

18: ¹H-¹³C HMBC,
CDCl3
f2 (ppm)
f1 (ppm)

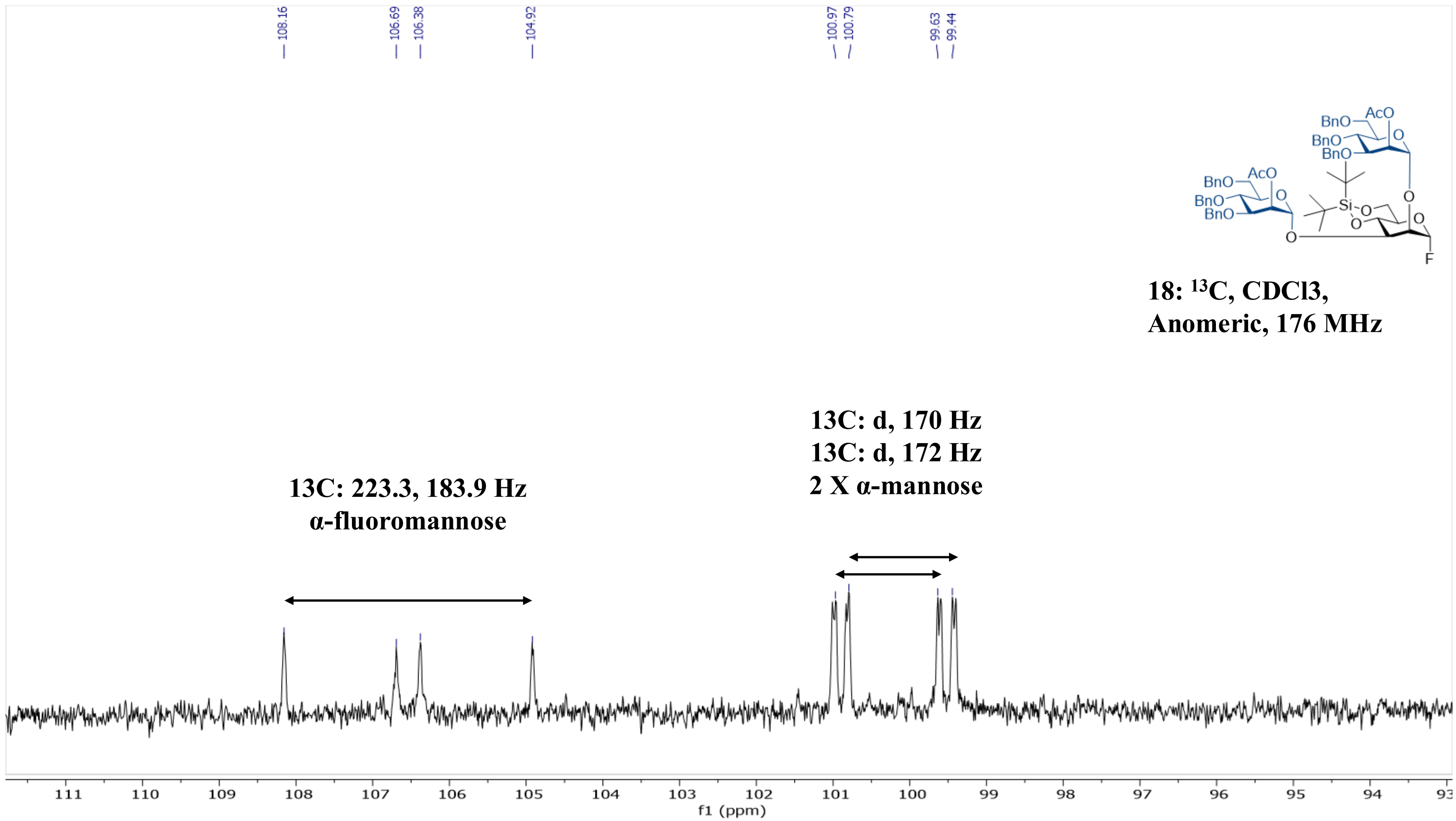
108.16
106.69
106.38
104.92
100.97
100.79
99.63
99.44
BnO
AcO
BnO
BnO
O
Si
O
O
O
F
18: ^{13}C, CDCl3,
Anomeric, 176 MHz
13C: d, 170 Hz
13C: d, 172 Hz
2 X α-mannose
13C: 223.3, 183.9 Hz
α-fluoromannose
111 110 109 108 107 106 105 104 103 102 101 100 99 98 97 96 95 94 93
f1 (ppm)

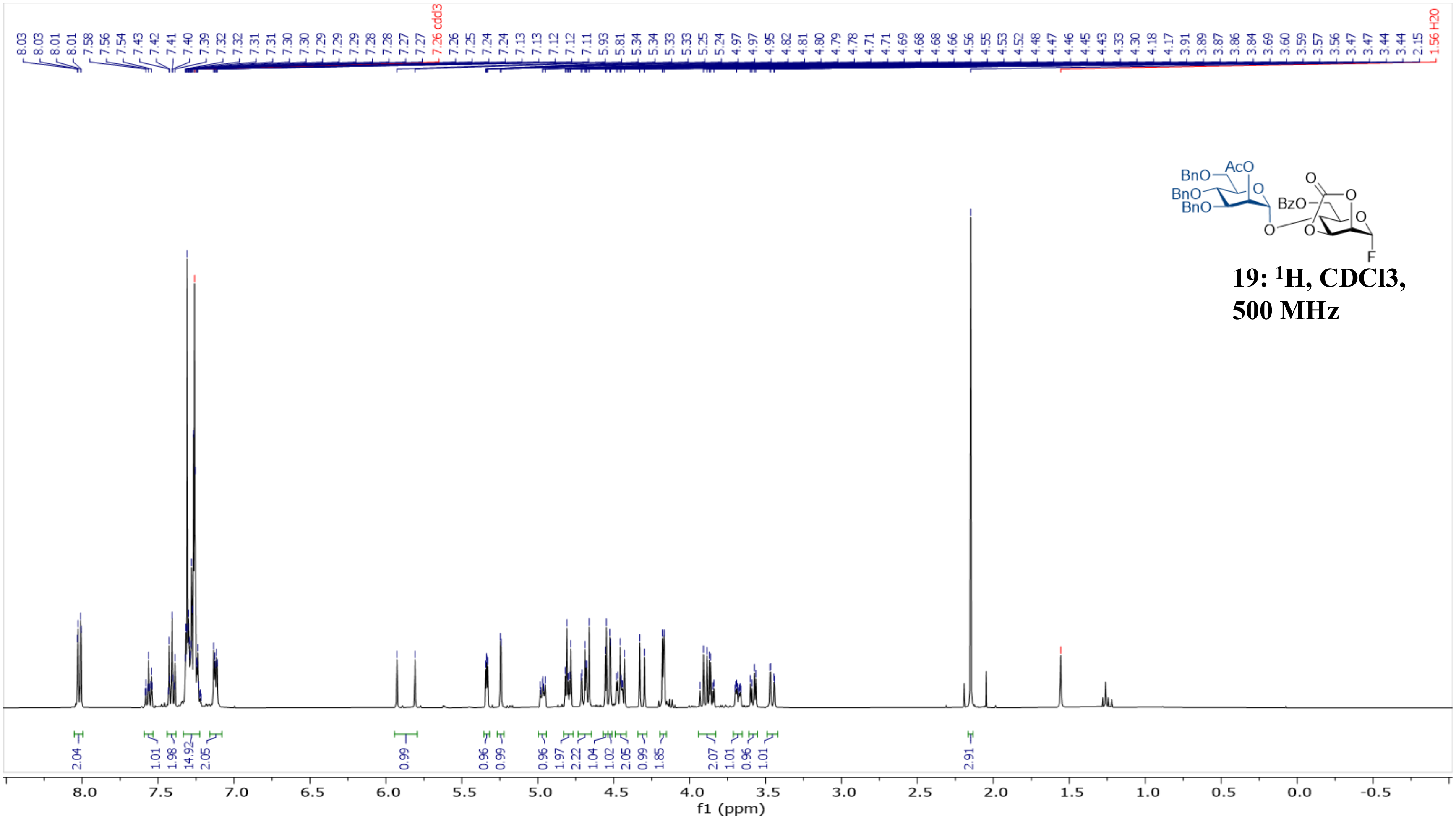
19: ¹H, CDCl3, 500 MHz
f1 (ppm)

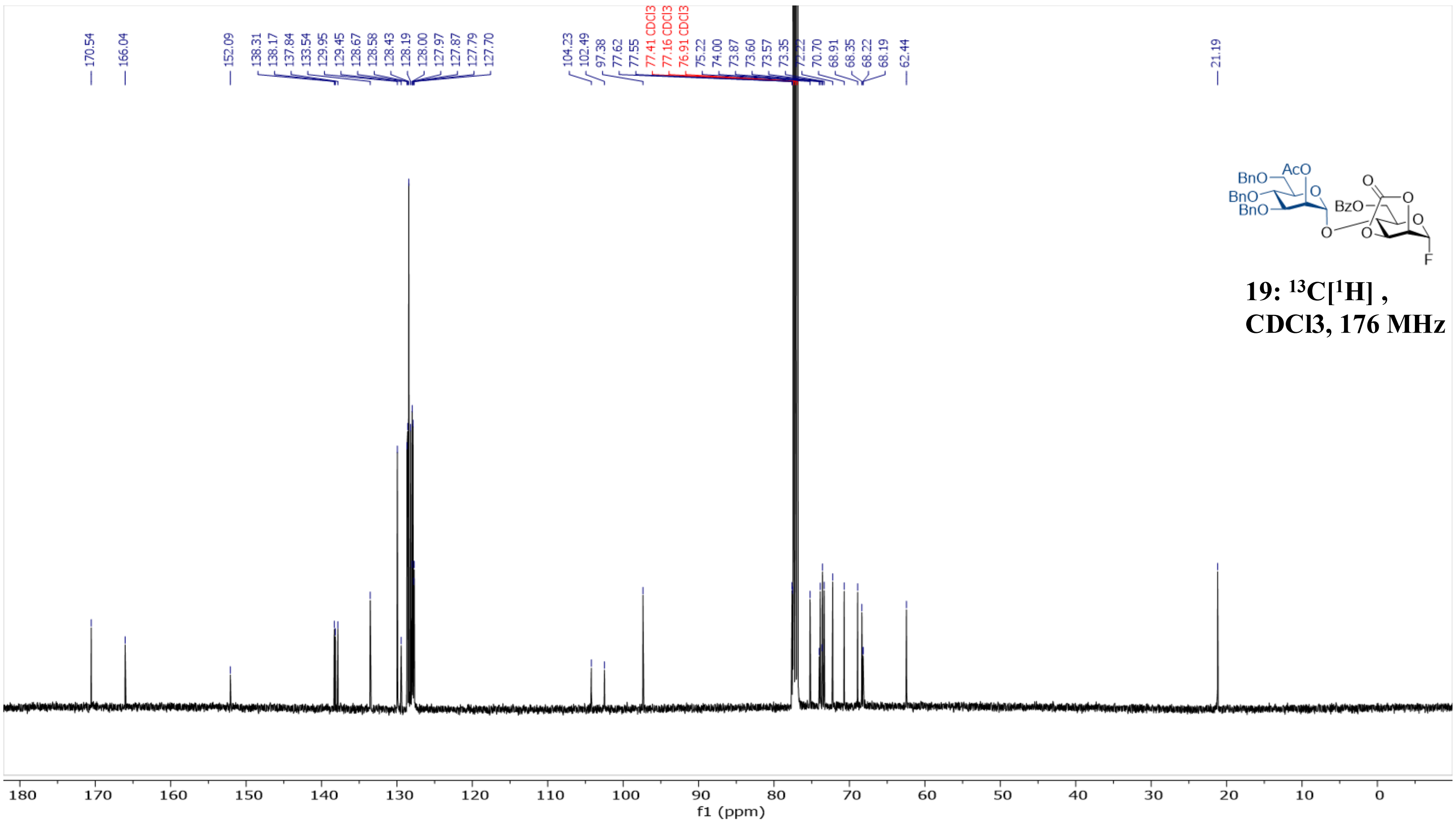
170.54
166.04
152.09
138.31
138.17
137.84
133.54
129.95
129.45
128.67
128.58
128.43
128.19
128.00
127.97
127.87
127.79
127.70
104.23
102.49
97.38
77.62
77.55
77.41 CDCl3
77.16 CDCl3
76.91 CDCl3
75.22
74.00
73.87
73.60
73.57
73.35
72.22
70.70
68.91
68.35
68.22
68.19
62.44
21.19
BnO
AcO
BnO
BnO
O
O
BzO
O
O
O
O
F
19: 13C[1H] ,
CDCl3, 176 MHz
180 170 160 150 140 130 120 110 100 90 80 70 60 50 40 30 20 10 0
f1 (ppm)

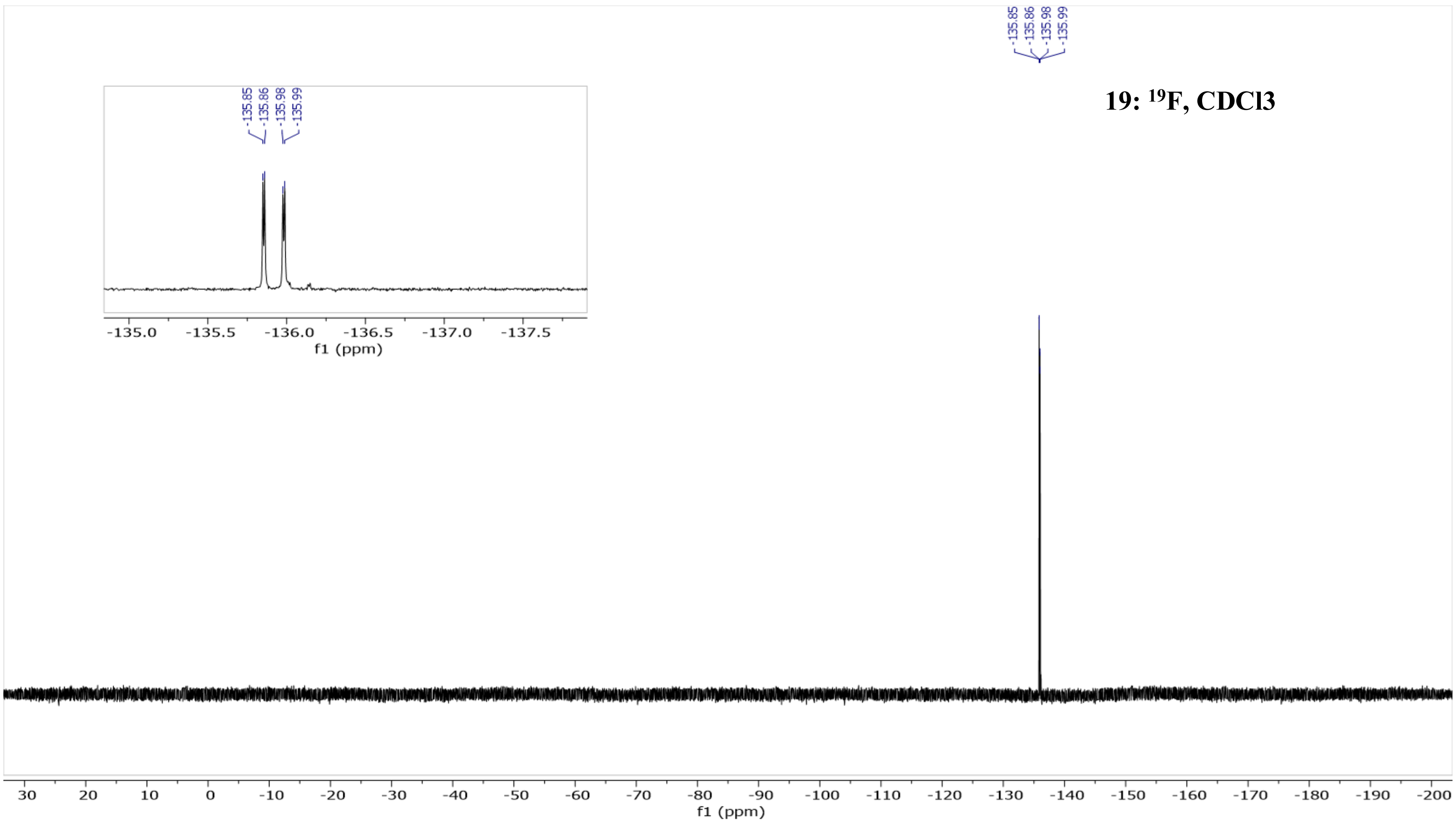
-135.85
-135.86
-135.98
-135.99
-135.0
-135.5
-136.0
-136.5
-137.0
-137.5
f1 (ppm)
30
20
10
0
-10
-20
-30
-40
-50
-60
-70
-80
-90
-100
-110
-120
-130
-140
-150
-160
-170
-180
-190
-200
f1 (ppm)

19: ^{19}F, CDCl3

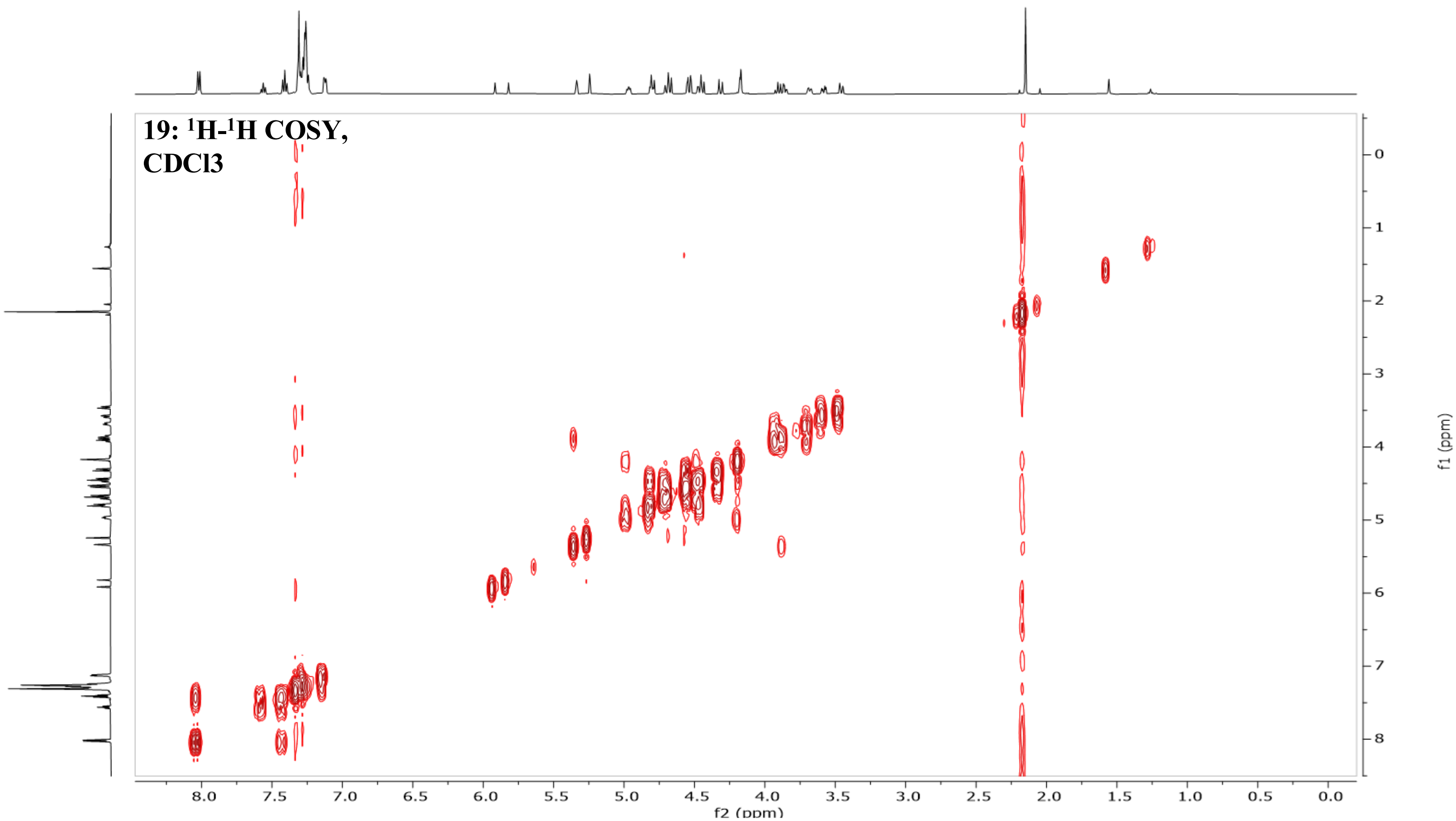
19: 1H-1H COSY,
CDCl3
f2 (ppm)
f1 (ppm)
8.0 7.5 7.0 6.5 6.0 5.5 5.0 4.5 4.0 3.5 3.0 2.5 2.0 1.5 1.0 0.5 0.0
0 1 2 3 4 5 6 7 8

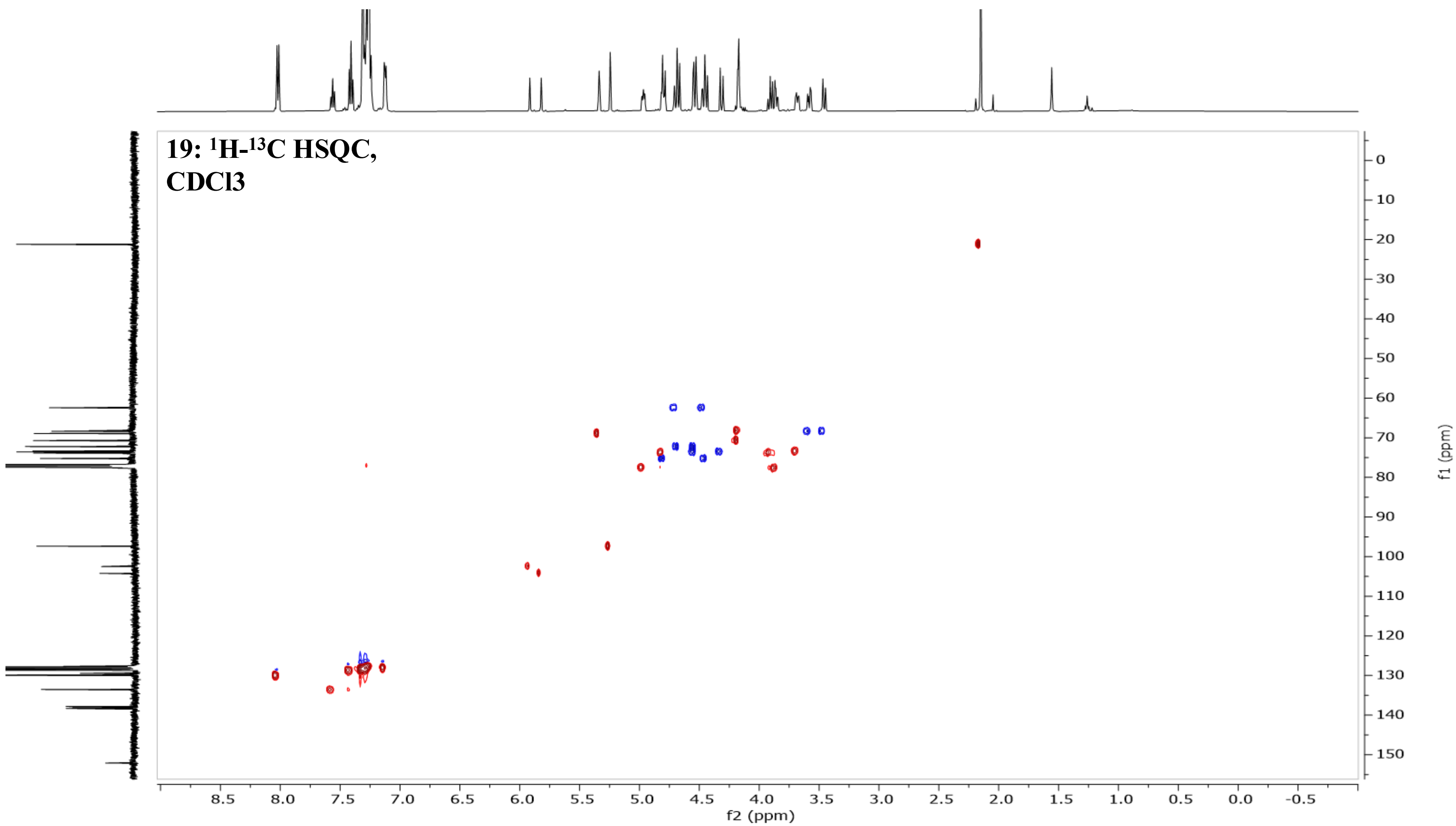
19: ^{1}H-^{13}C HSQC,
CDCl3
f1 (ppm)
f2 (ppm)
8.5 8.0 7.5 7.0 6.5 6.0 5.5 5.0 4.5 4.0 3.5 3.0 2.5 2.0 1.5 1.0 0.5 0.0 -0.5
0 10 20 30 40 50 60 70 80 90 100 110 120 130 140 150

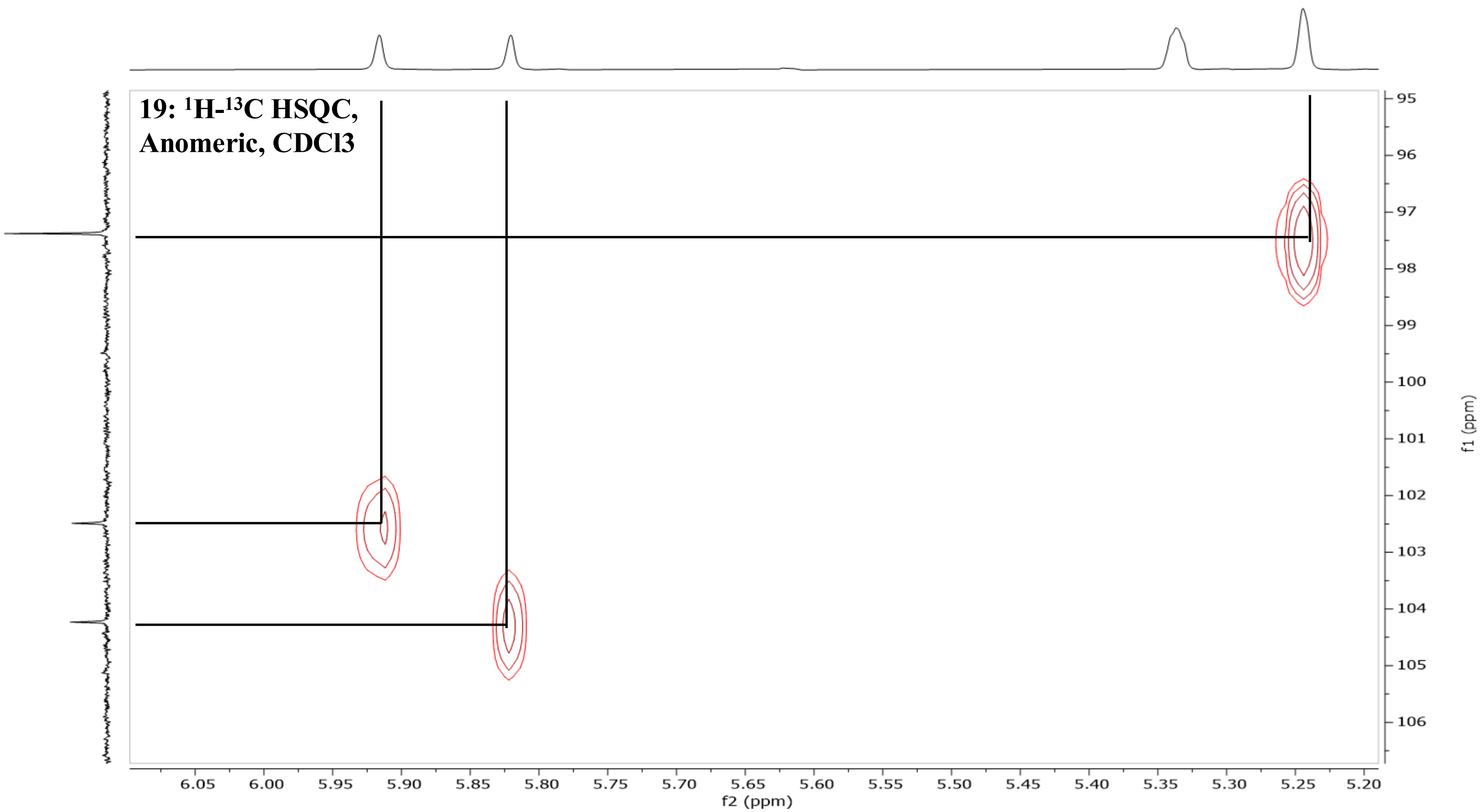
19: ¹H-¹³C HSQC,
Anomeric, CDCl3
6.05
6.00
5.95
5.90
5.85
5.80
5.75
5.70
5.65
5.60
5.55
5.50
5.45
5.40
5.35
5.30
5.25
5.20
f2 (ppm)
95
96
97
98
99
100
101
102
103
104
105
106
f1 (ppm)

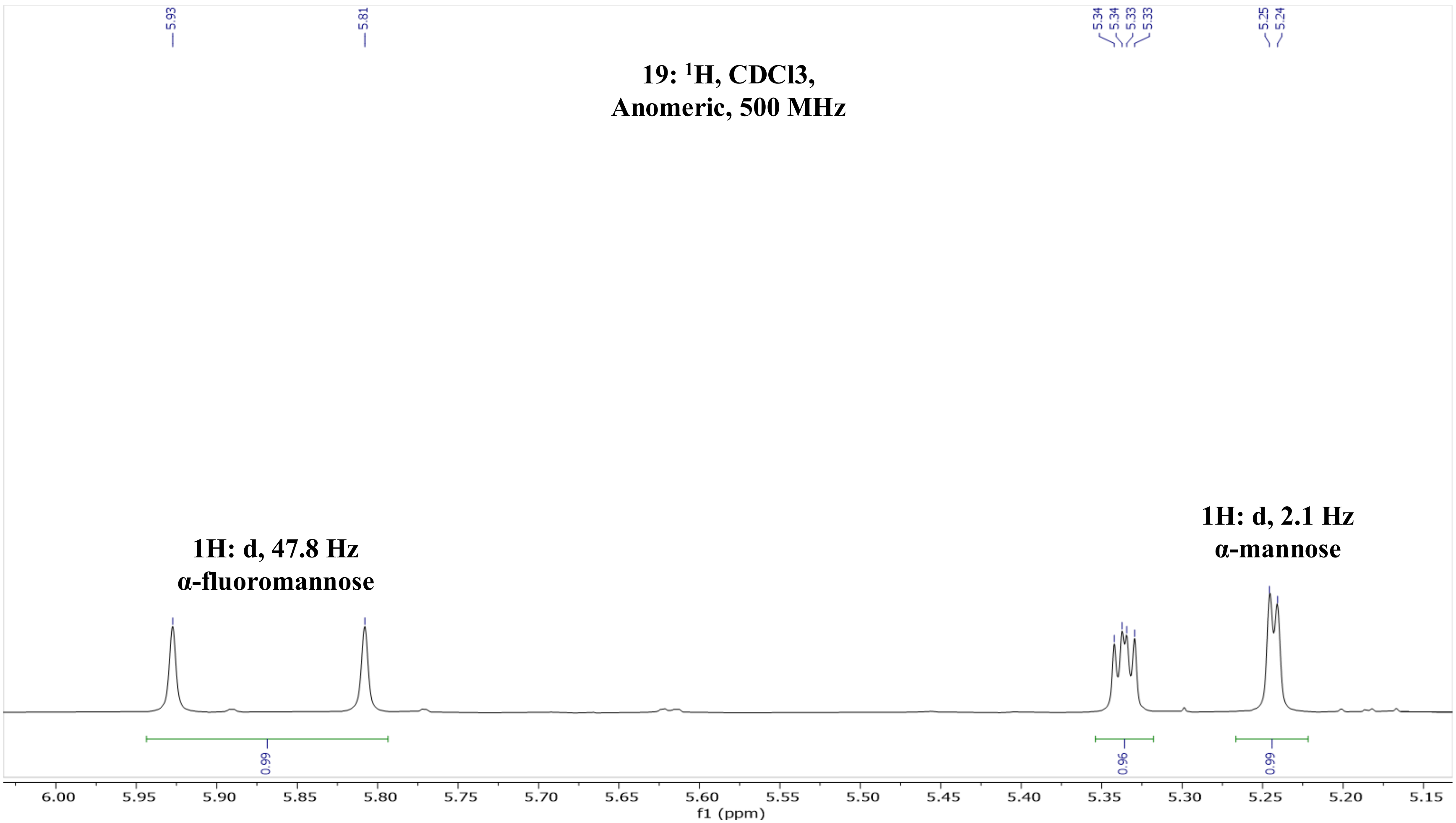

19: 1H, CDCl3,
Anomeric, 500 MHz
1H: d, 47.8 Hz
α-fluoromannose
1H: d, 2.1 Hz
α-mannose
5.93
5.81
5.34
5.34
5.33
5.33
5.25
5.24
0.99
0.96
0.99
6.00
5.95
5.90
5.85
5.80
5.75
5.70
5.65
5.60
5.55
5.50
5.45
5.40
5.35
5.30
5.25
5.20
5.15
f1 (ppm)

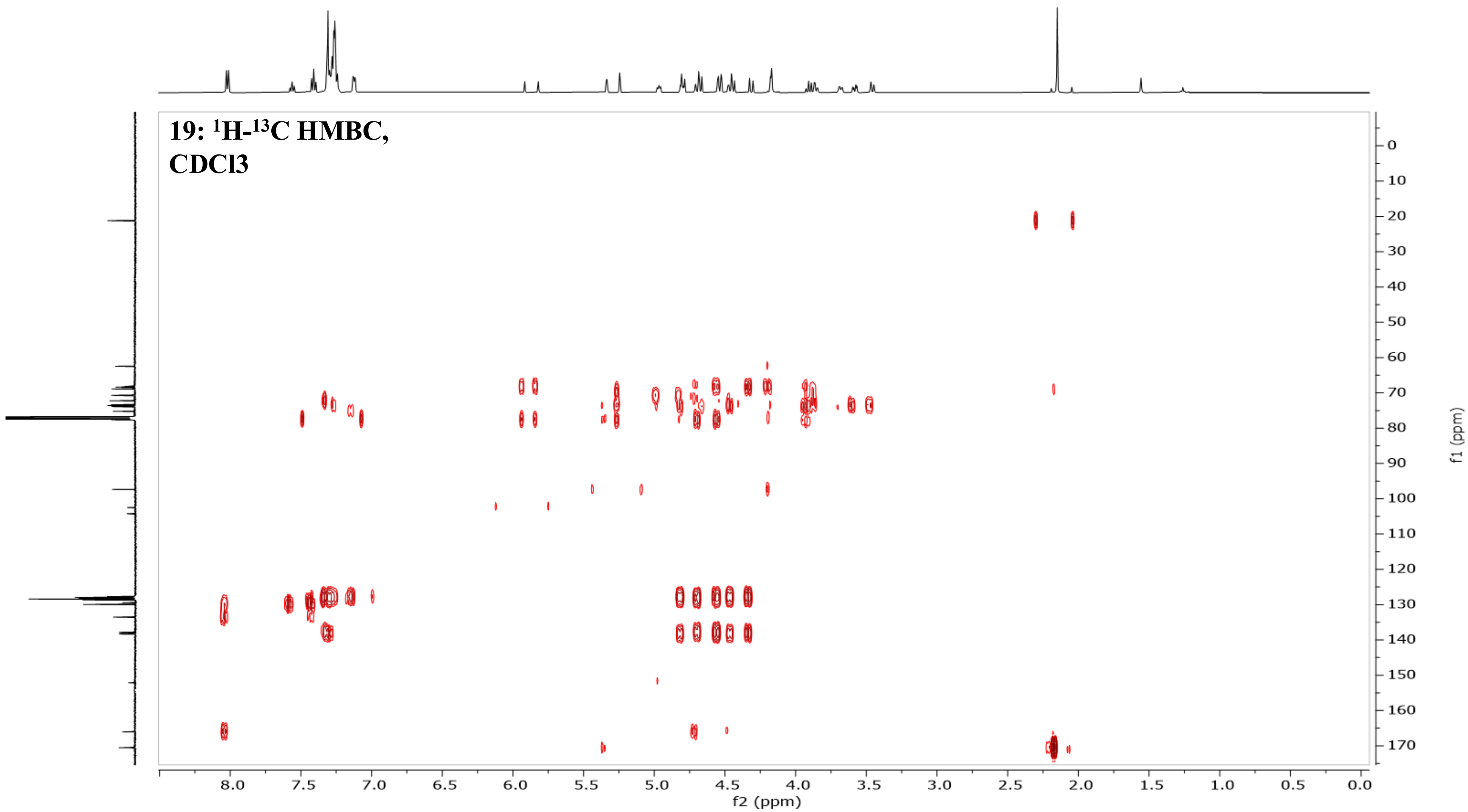
19: ¹H-¹³C HMBC, CDCl3
f2 (ppm)
f1 (ppm)
8.0 7.5 7.0 6.5 6.0 5.5 5.0 4.5 4.0 3.5 3.0 2.5 2.0 1.5 1.0 0.5 0.0
0 10 20 30 40 50 60 70 80 90 100 110 120 130 140 150 160 170

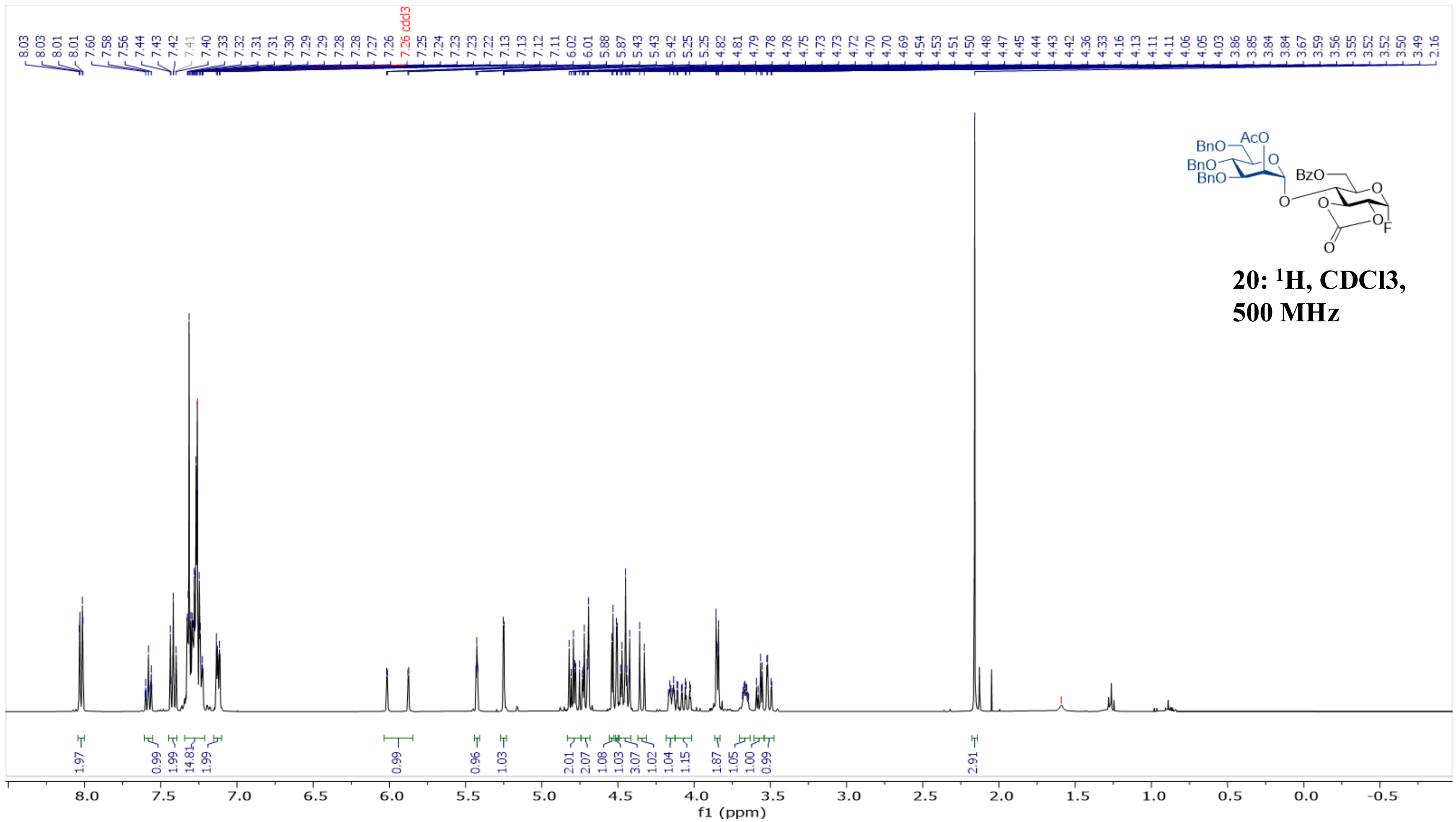
20: ¹H, CDCl3, 500 MHz
f1 (ppm)

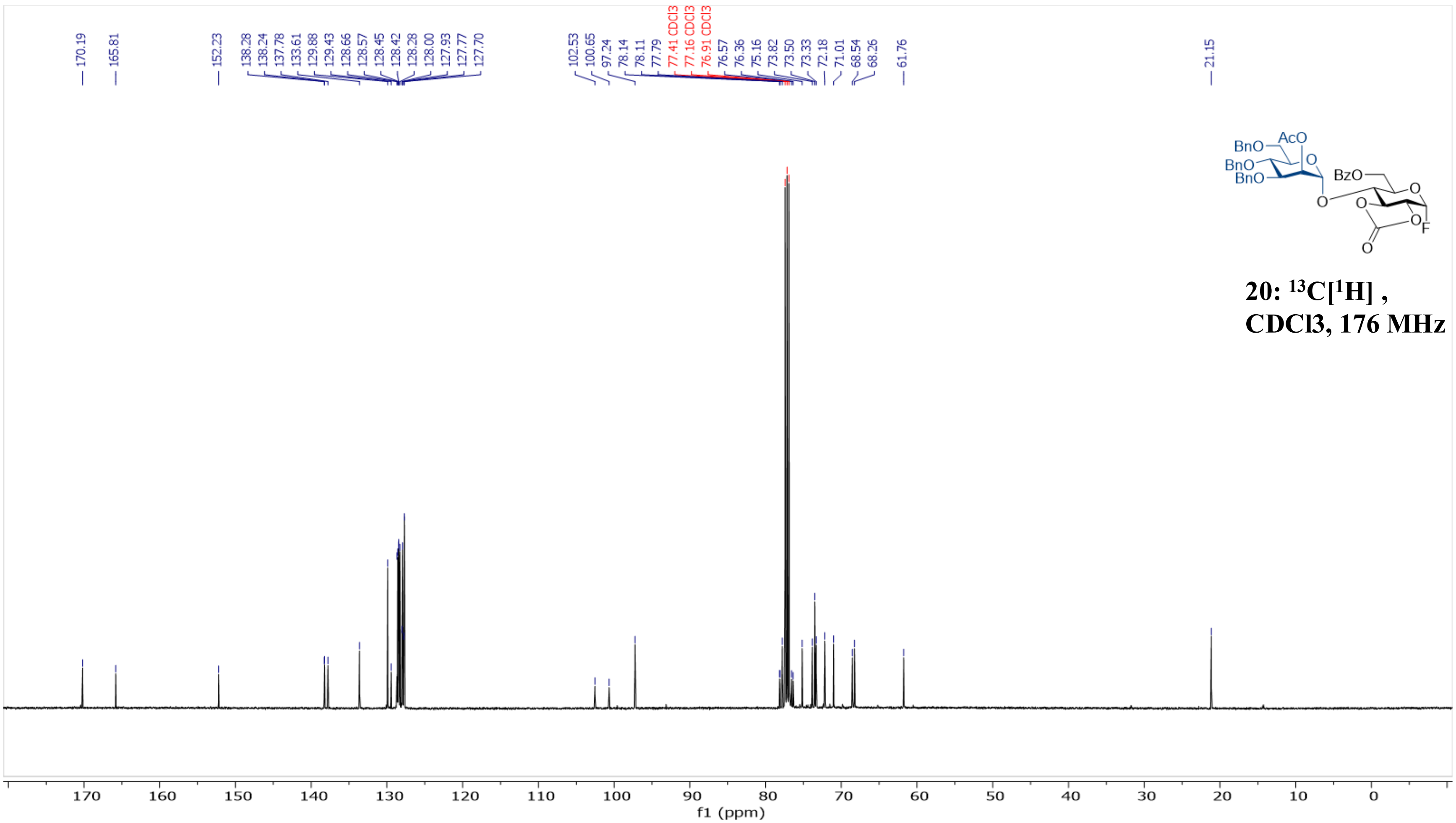

20: $^{13}C[^{1}H]$, CDCl3, 176 MHz

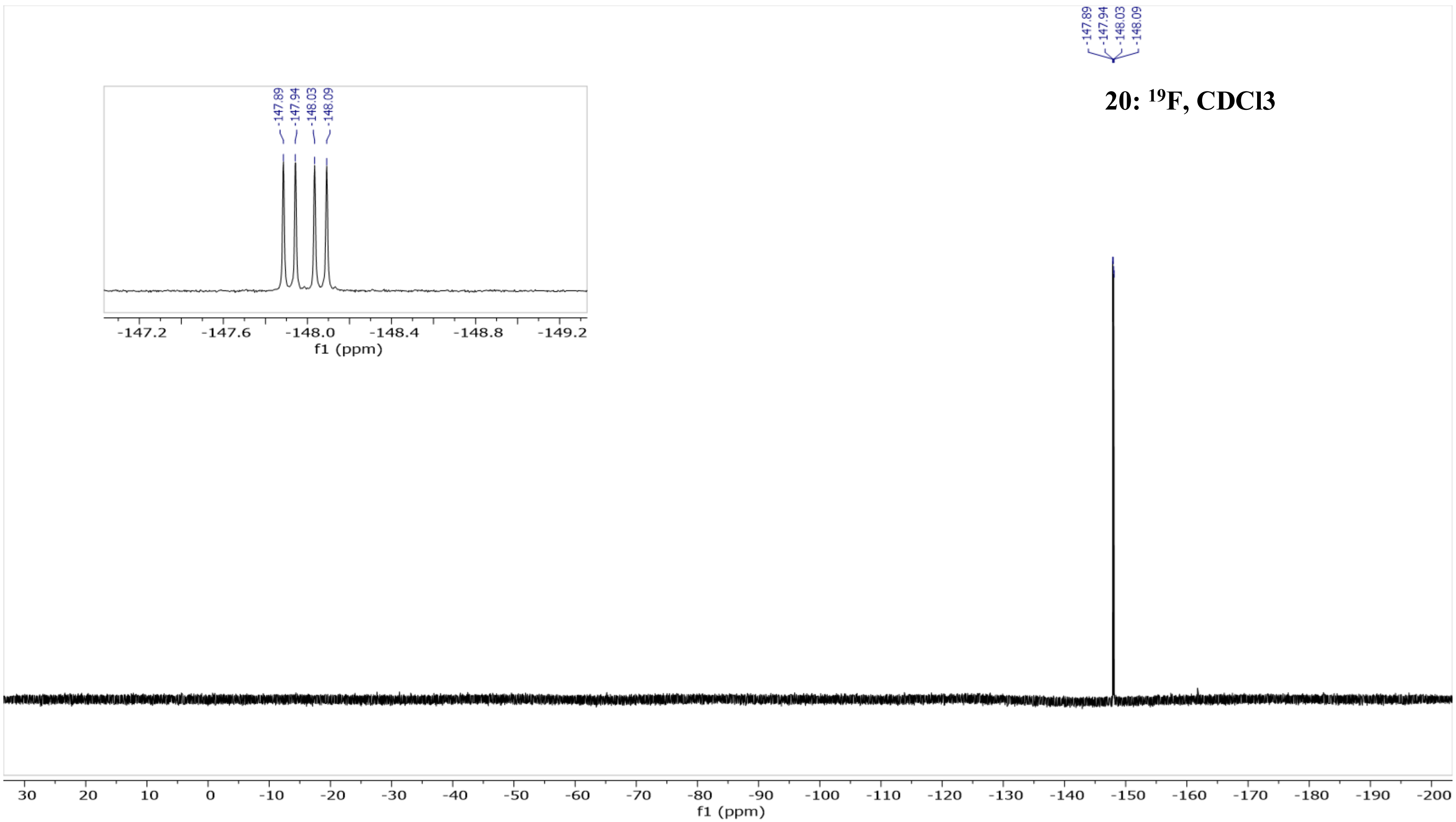
-147.89
-147.94
-148.03
-148.09
20: 19F, CDCl3
-147.2
-147.6
-148.0
-148.4
-148.8
-149.2
f1 (ppm)
30 20 10 0 -10 -20 -30 -40 -50 -60 -70 -80 -90 -100 -110 -120 -130 -140 -150 -160 -170 -180 -190 -200
f1 (ppm)

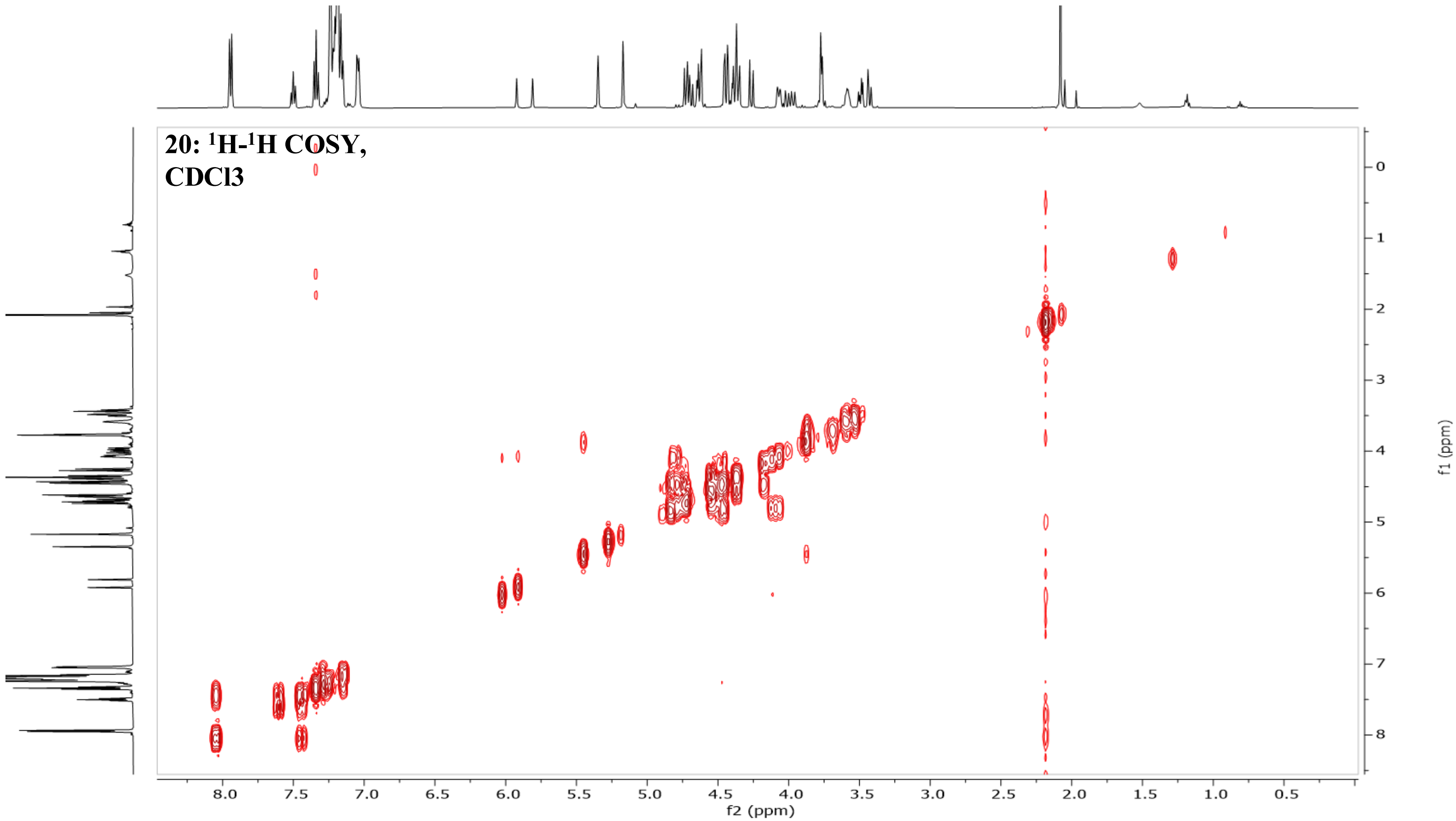

20: ¹H-¹H COSY,
CDCl3
f2 (ppm)
f1 (ppm)

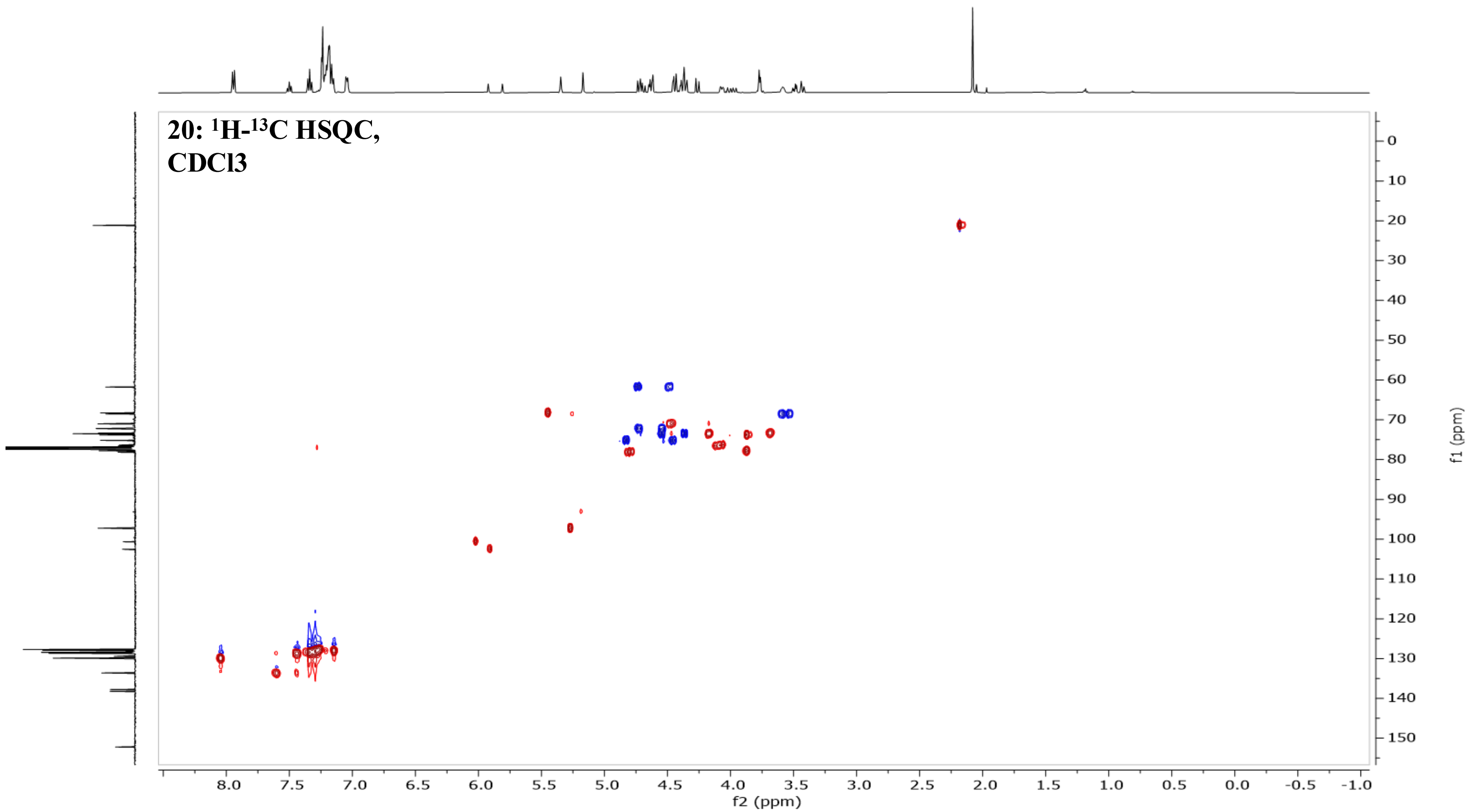
20: ¹H-¹³C HSQC,
CDCl3
f2 (ppm)
f1 (ppm)

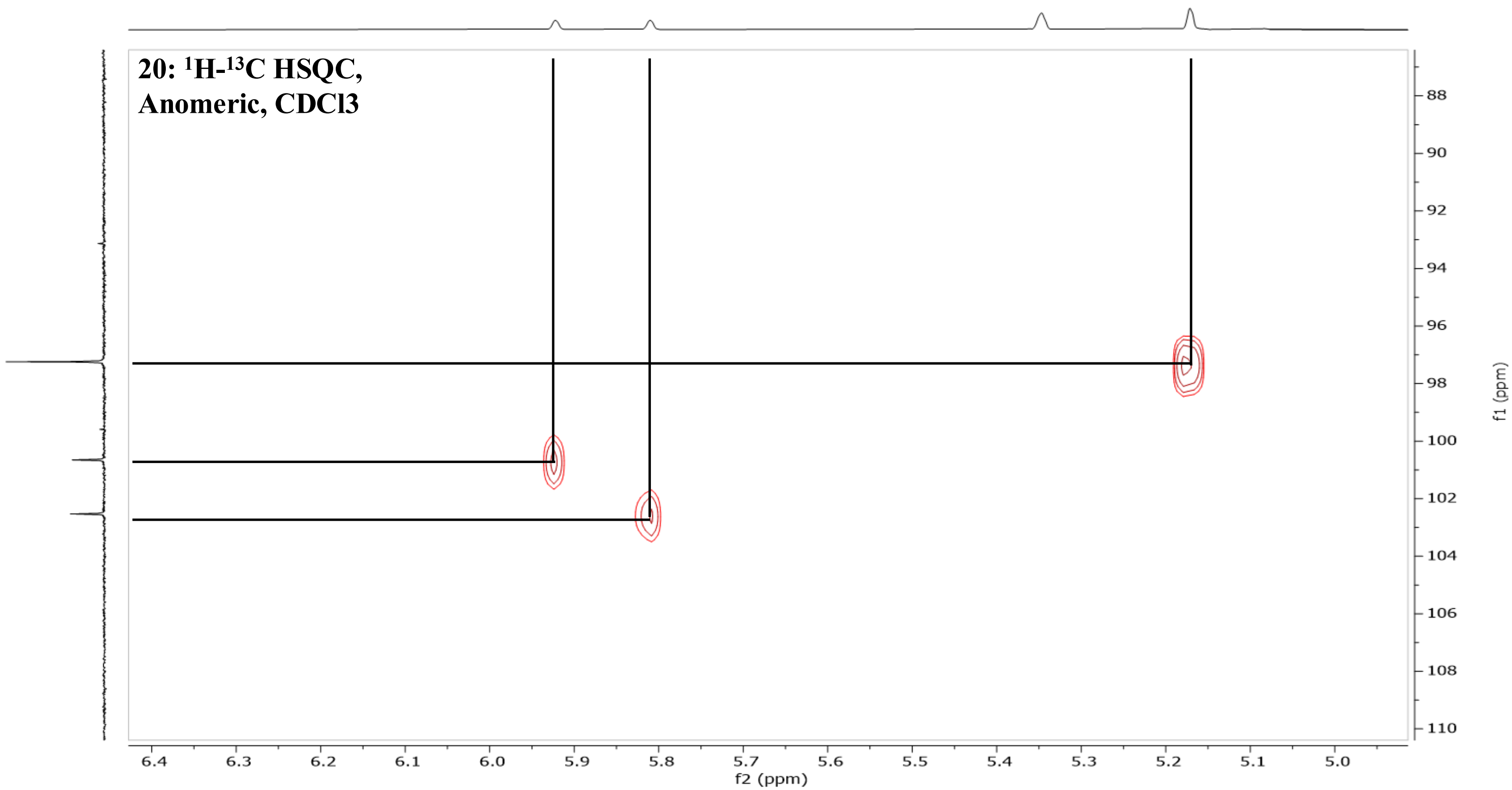
20: 1H-13C HSQC,
Anomeric, CDCl3
f2 (ppm)
f1 (ppm)
6.4
6.3
6.2
6.1
6.0
5.9
5.8
5.7
5.6
5.5
5.4
5.3
5.2
5.1
5.0
88
90
92
94
96
98
100
102
104
106
108
110

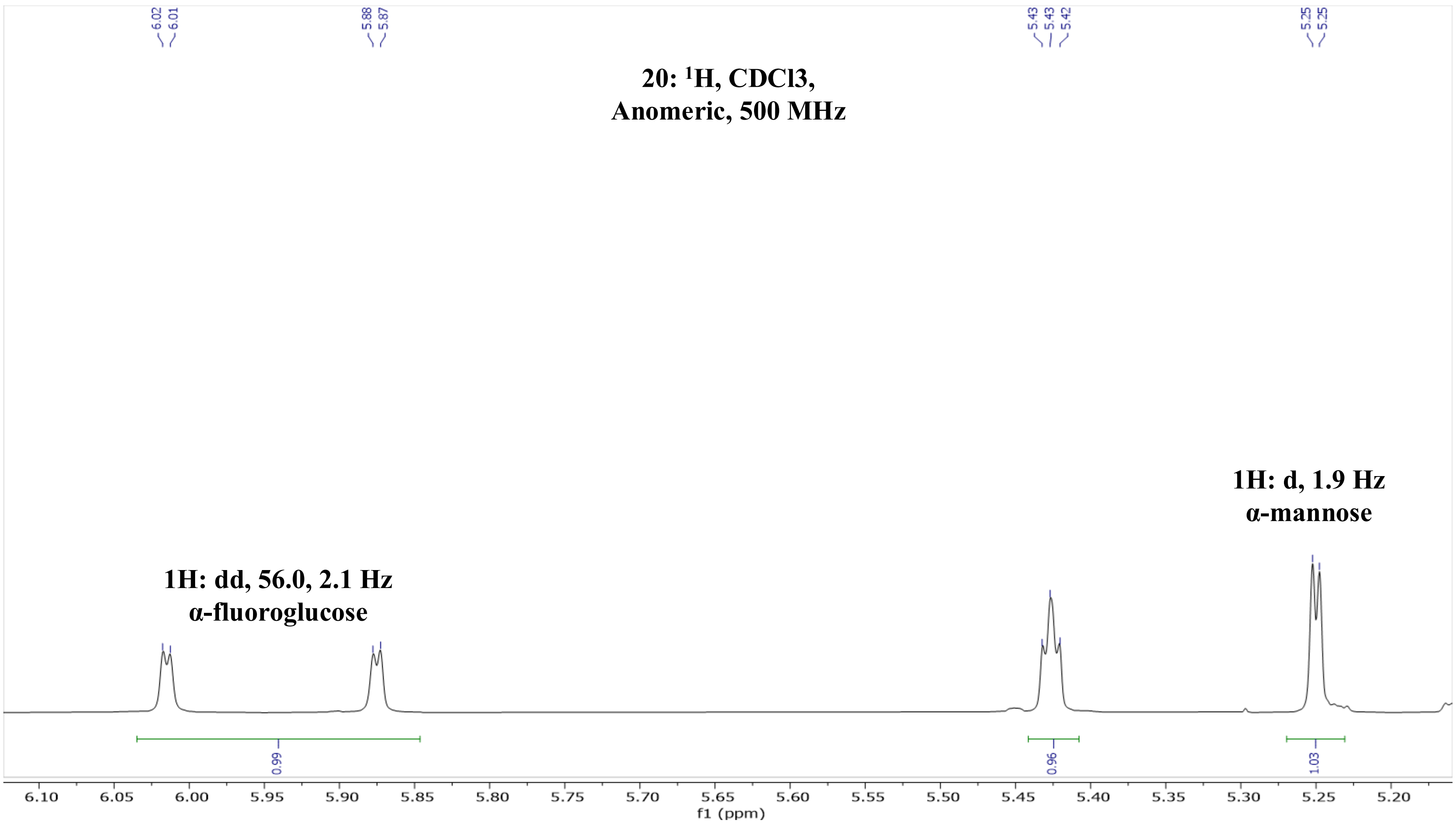

6.02
6.01
5.88
5.87
5.43
5.43
5.42
5.25
5.25
20: ¹H, CDCl3,
Anomeric, 500 MHz
1H: d, 1.9 Hz
α-mannose
1H: dd, 56.0, 2.1 Hz
α-fluoroglucose
0.99
0.96
1.03
6.10
6.05
6.00
5.95
5.90
5.85
5.80
5.75
5.70
5.65
5.60
5.55
5.50
5.45
5.40
5.35
5.30
5.25
5.20
f1 (ppm)

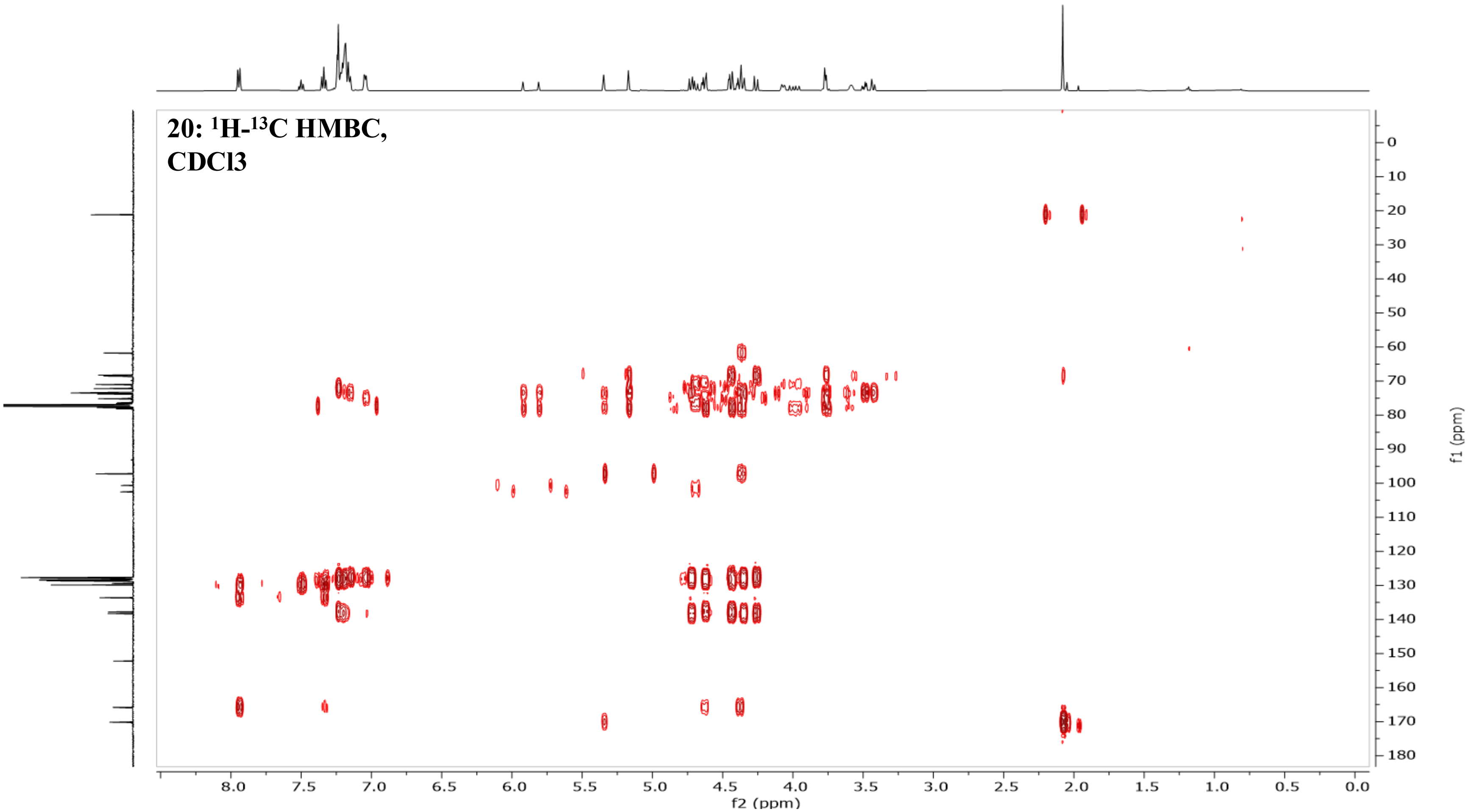

20: ¹H-¹³C HMBC,
CDCl3
0
10
20
30
40
50
60
70
80
90
100
110
120
130
140
150
160
170
180
f1 (ppm)
8.0
7.5
7.0
6.5
6.0
5.5
5.0
4.5
4.0
3.5
3.0
2.5
2.0
1.5
1.0
0.5
0.0
f2 (ppm)

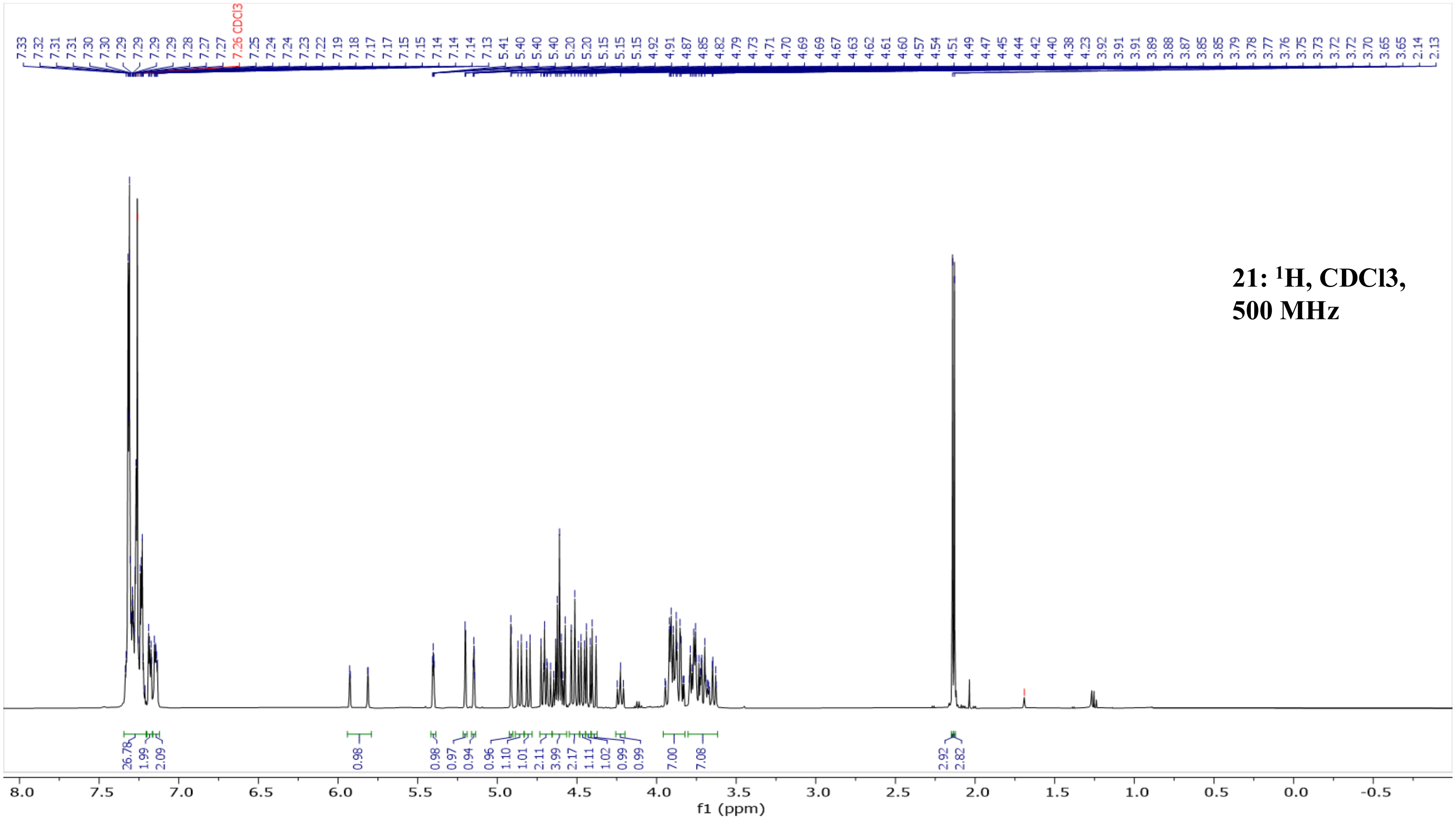
21: ¹H, CDCl3,
500 MHz
f1 (ppm)

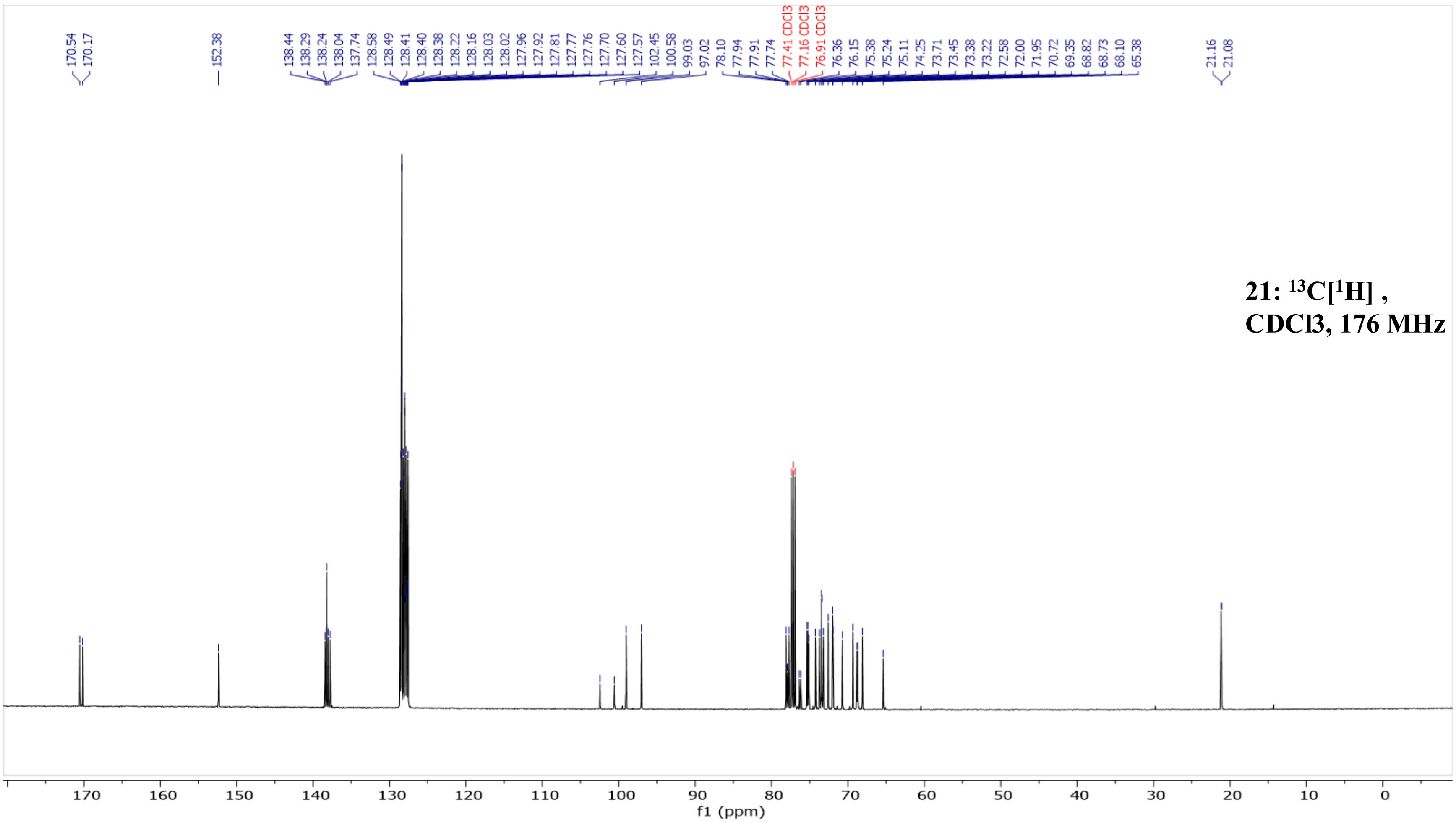

21: $^{13}C[^{1}H]$, CDCl3, 176 MHz

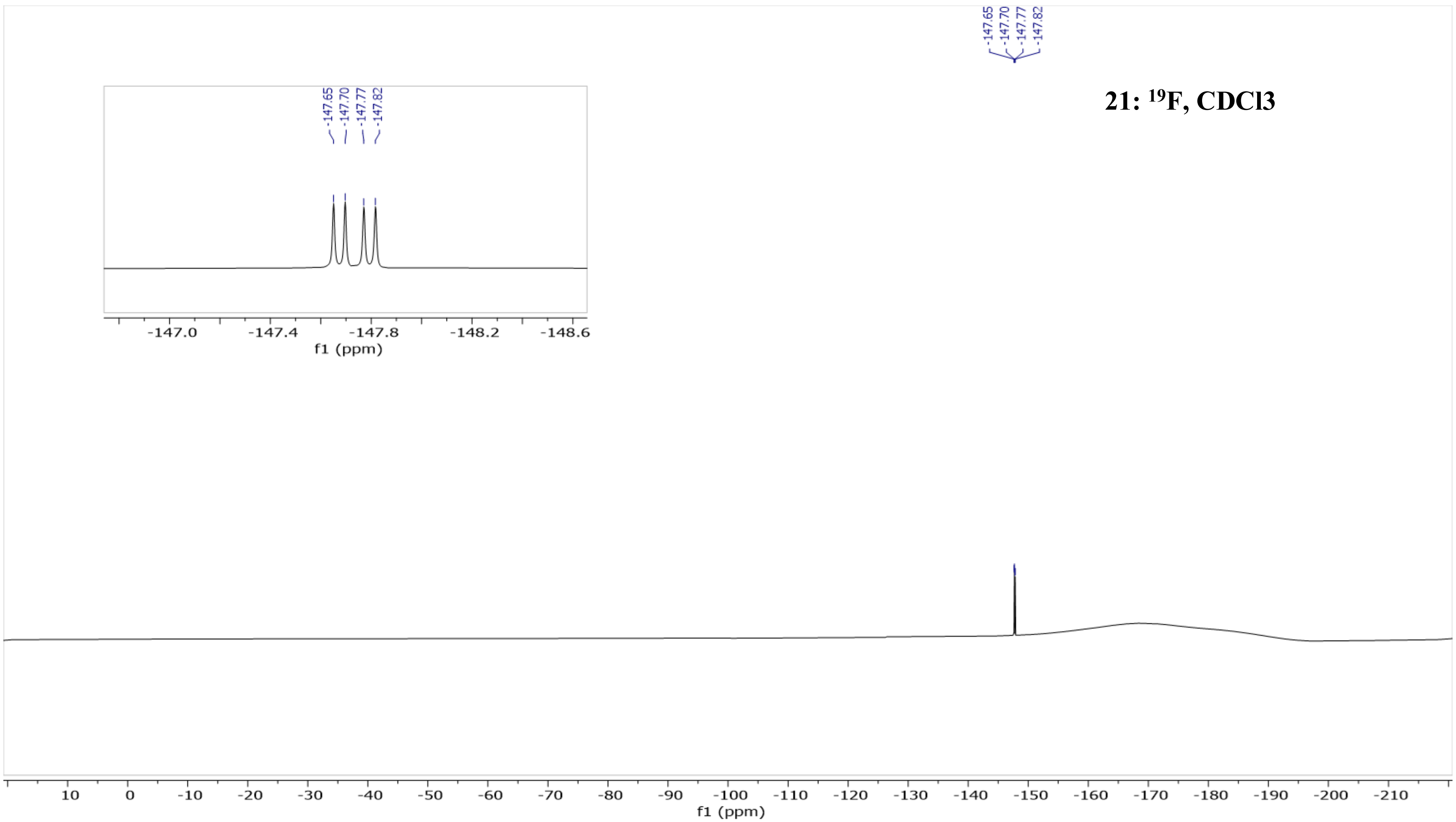
-147.65
-147.70
-147.77
-147.82
21: ^{19}F, CDCl3
-147.0
-147.4
-147.8
-148.2
-148.6
f1 (ppm)
10 0 -10 -20 -30 -40 -50 -60 -70 -80 -90 -100 -110 -120 -130 -140 -150 -160 -170 -180 -190 -200 -210
f1 (ppm)

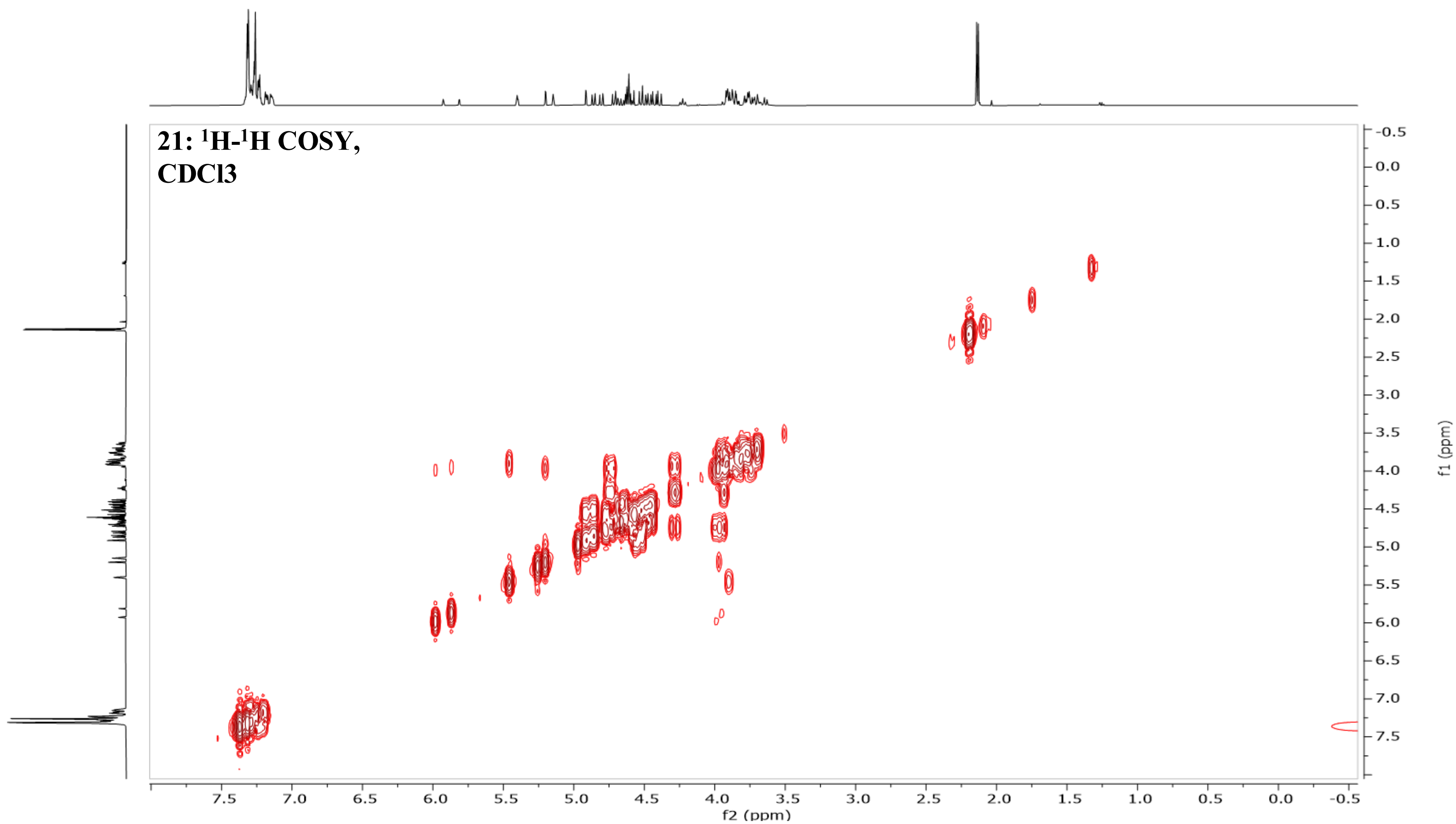
21: ¹H-¹H COSY,
CDCl3
f2 (ppm)
f1 (ppm)

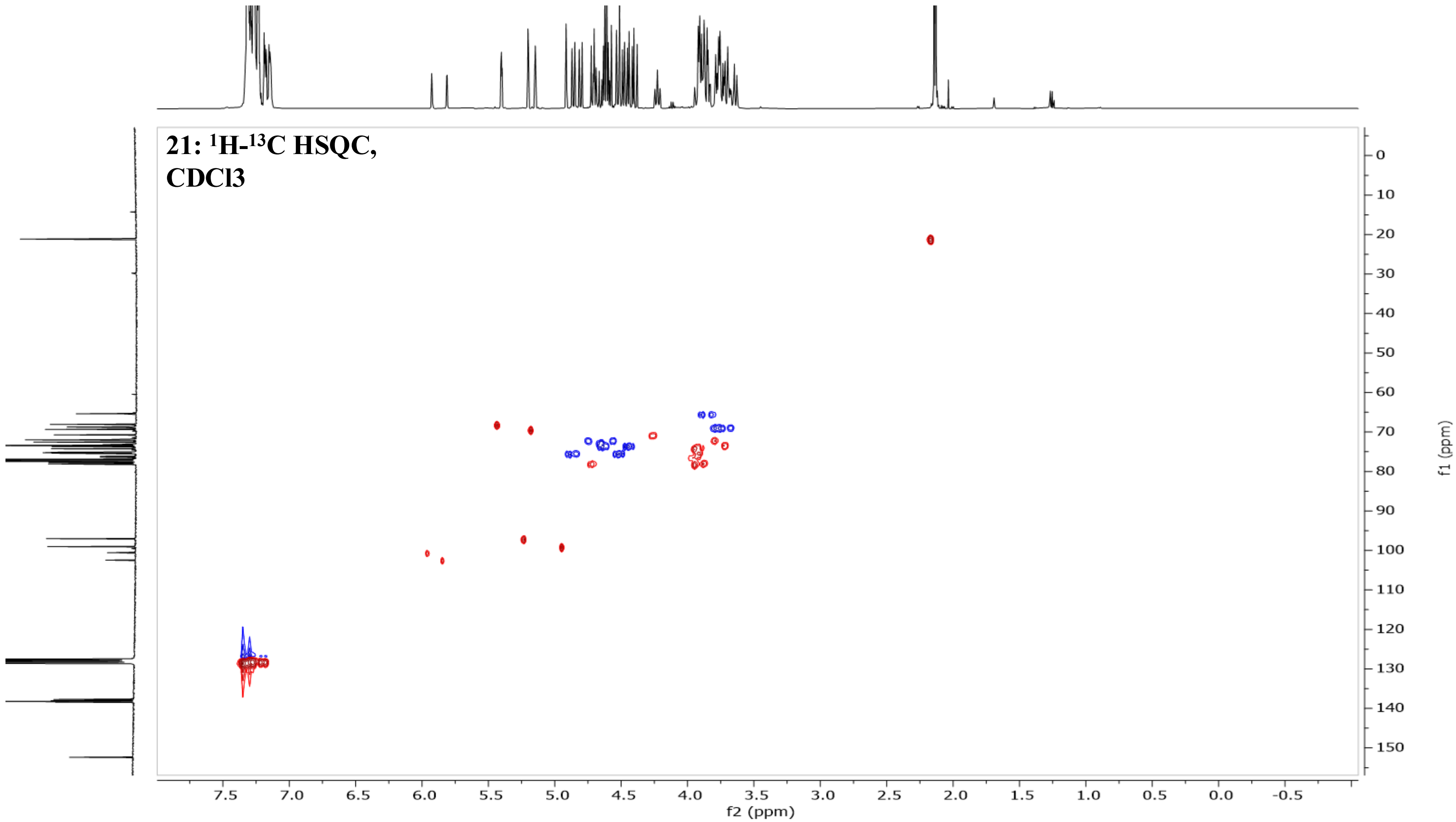

21: ¹H-¹³C HSQC,
CDCl3
f2 (ppm)
f1 (ppm)

21: ^{1}H-^{13}C HSQC, Anomeric, CDCl3

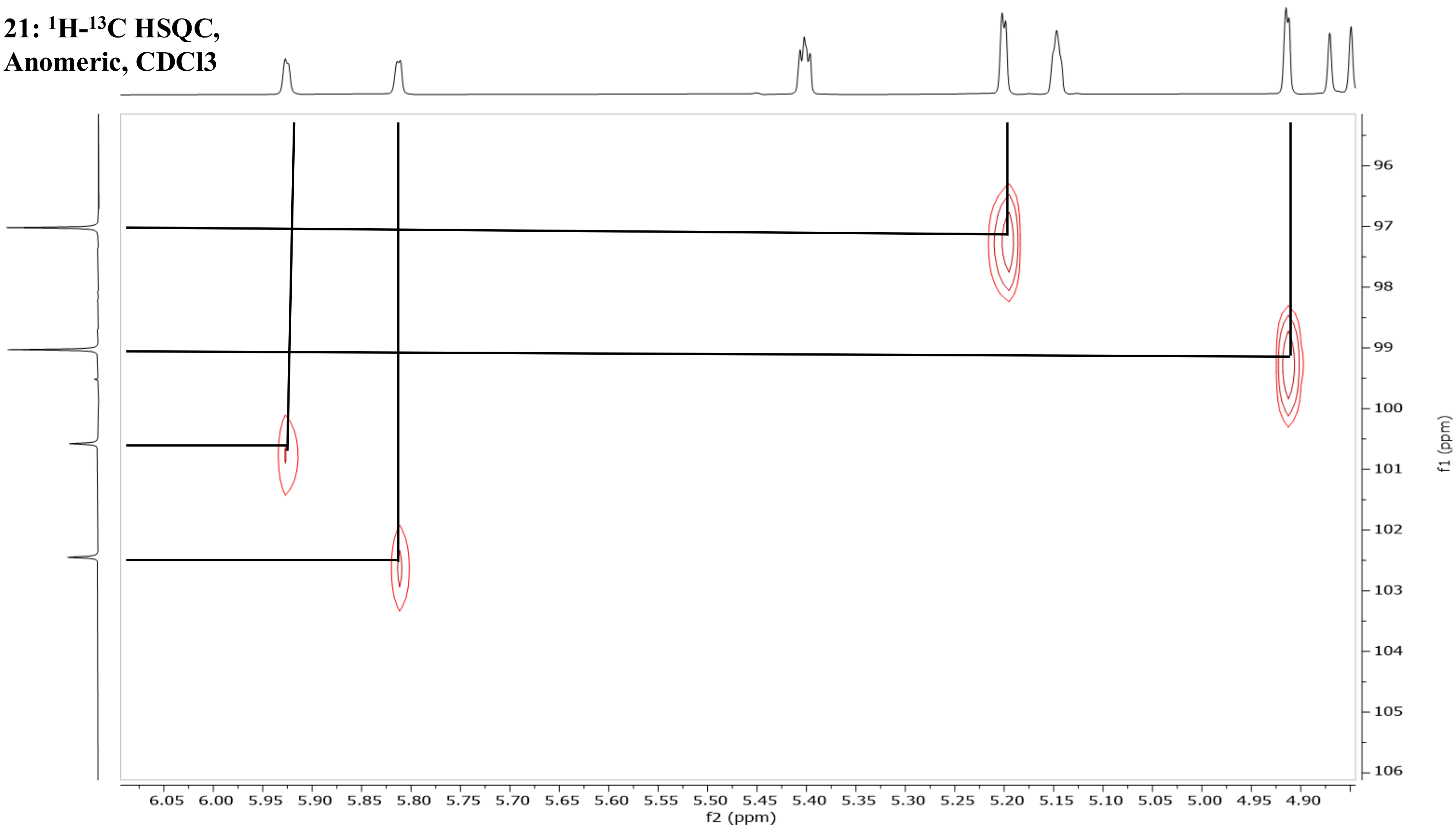

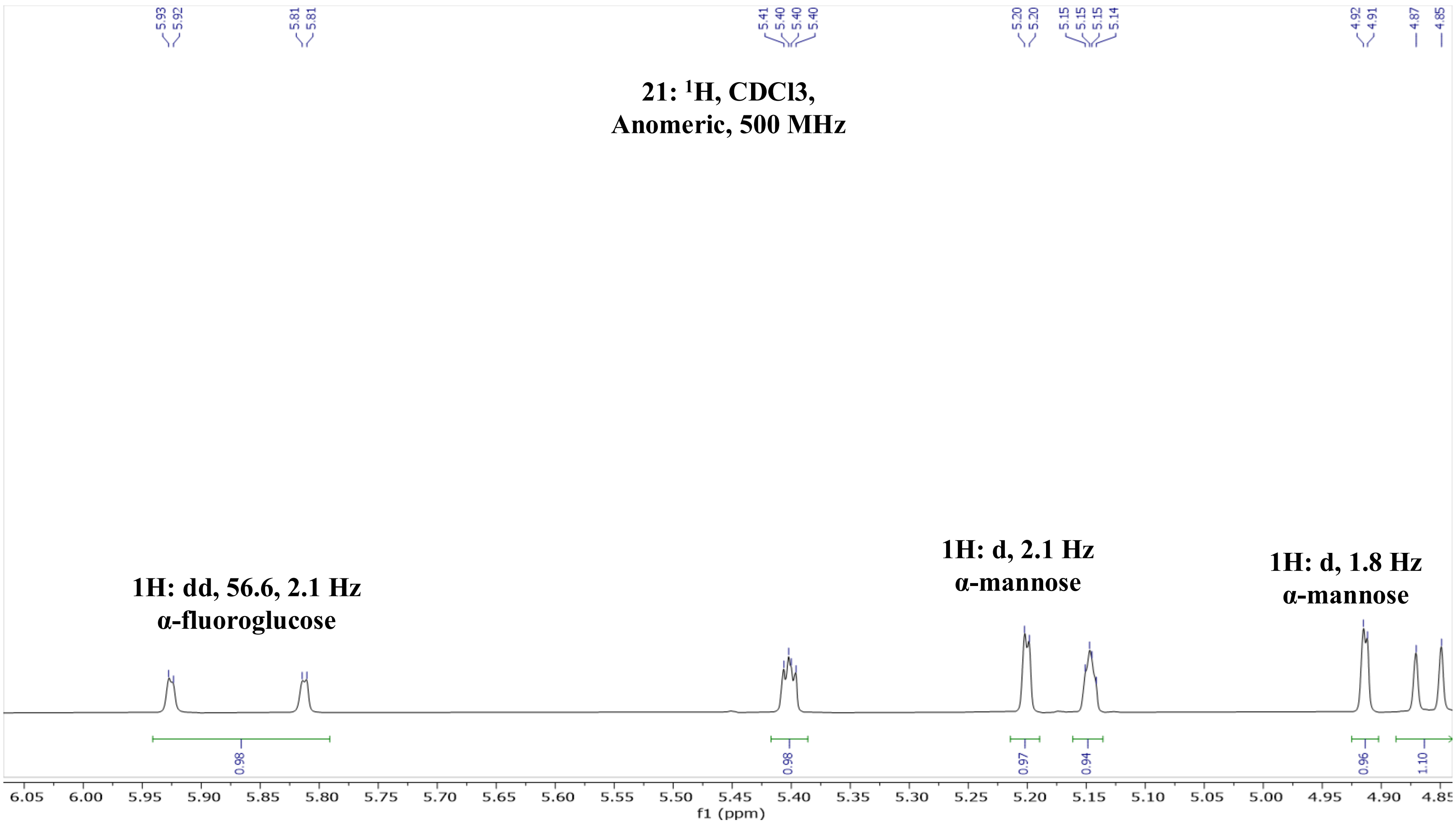
21: ¹H, CDCl3,
Anomeric, 500 MHz
1H: dd, 56.6, 2.1 Hz
α-fluoroglucose
1H: d, 2.1 Hz
α-mannose
1H: d, 1.8 Hz
α-mannose
f1 (ppm)

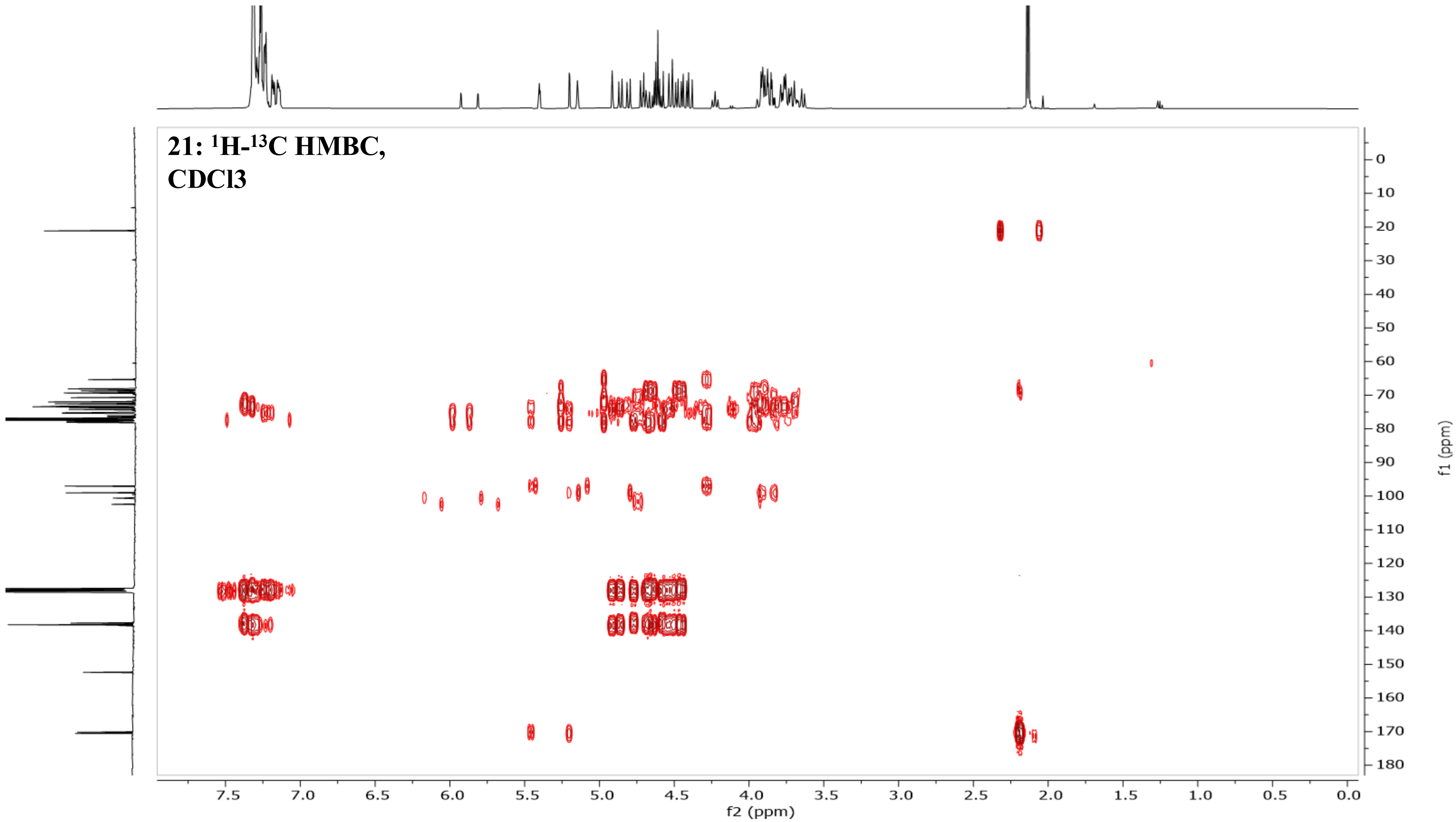

21: ¹H-¹³C HMBC,
CDCl3
f2 (ppm)
f1 (ppm)

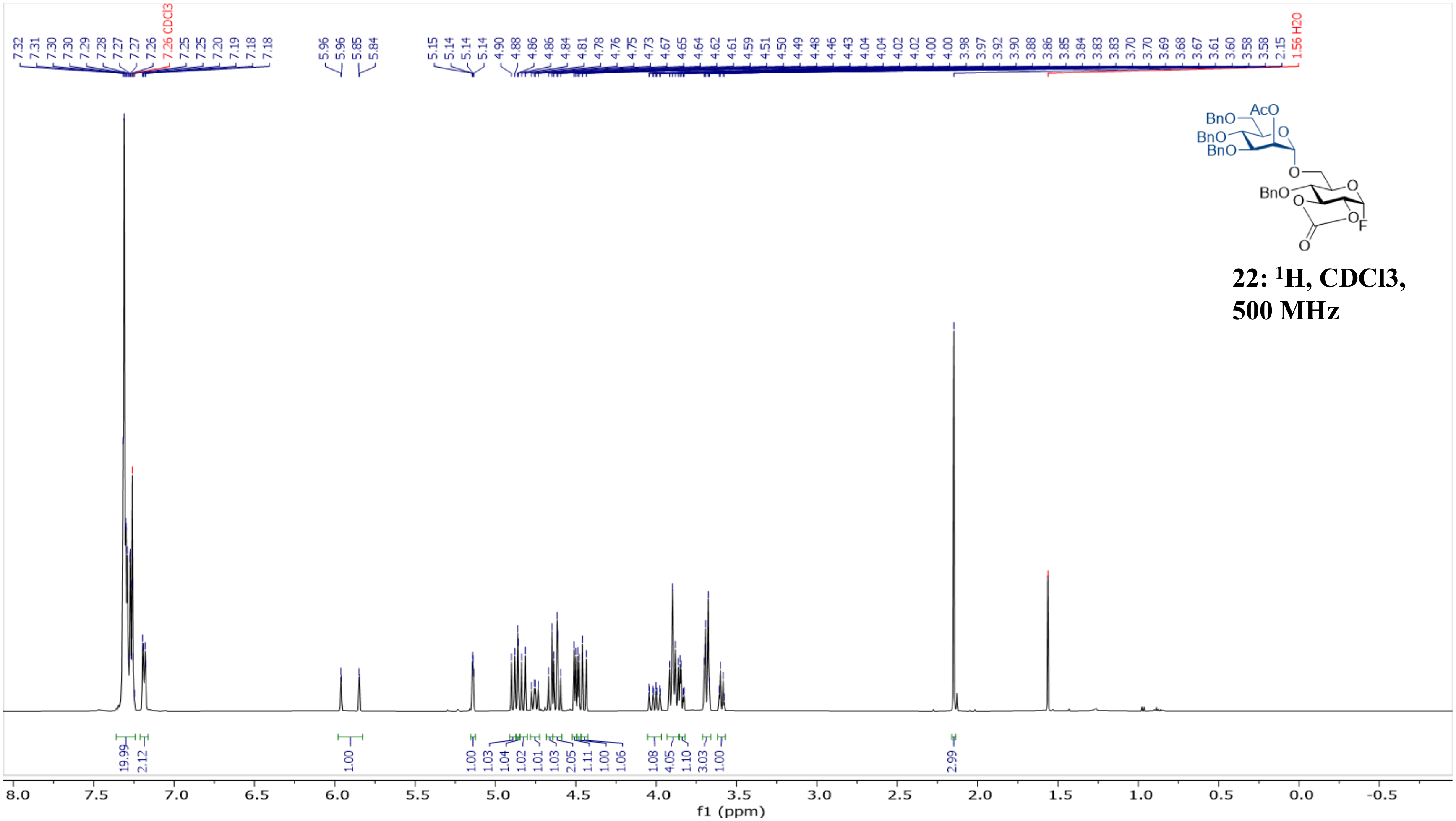

22: ^{1}H, CDCl3, 500 MHz

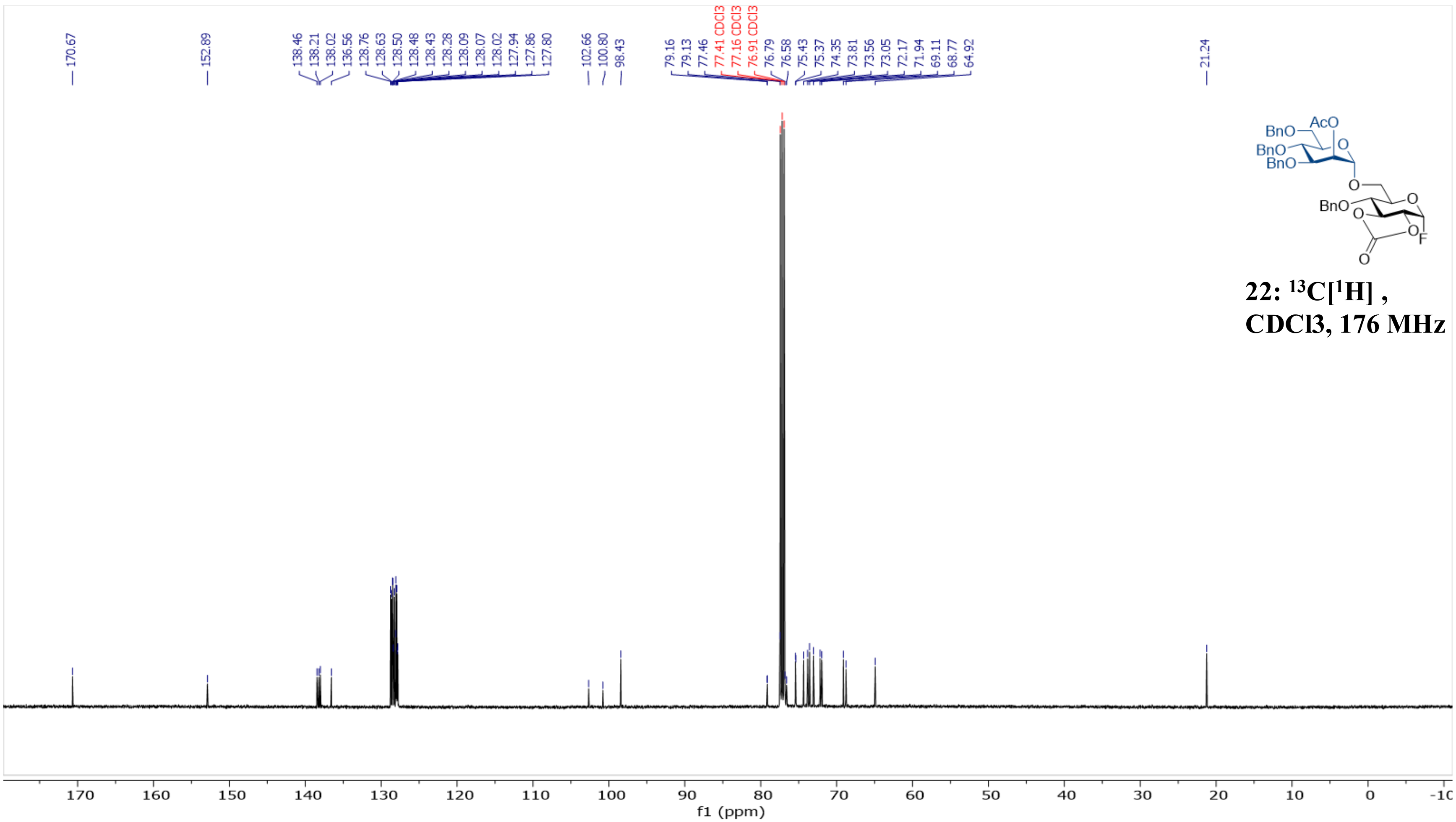

22: $^{13}C[^{1}H]$, CDCl3, 176 MHz

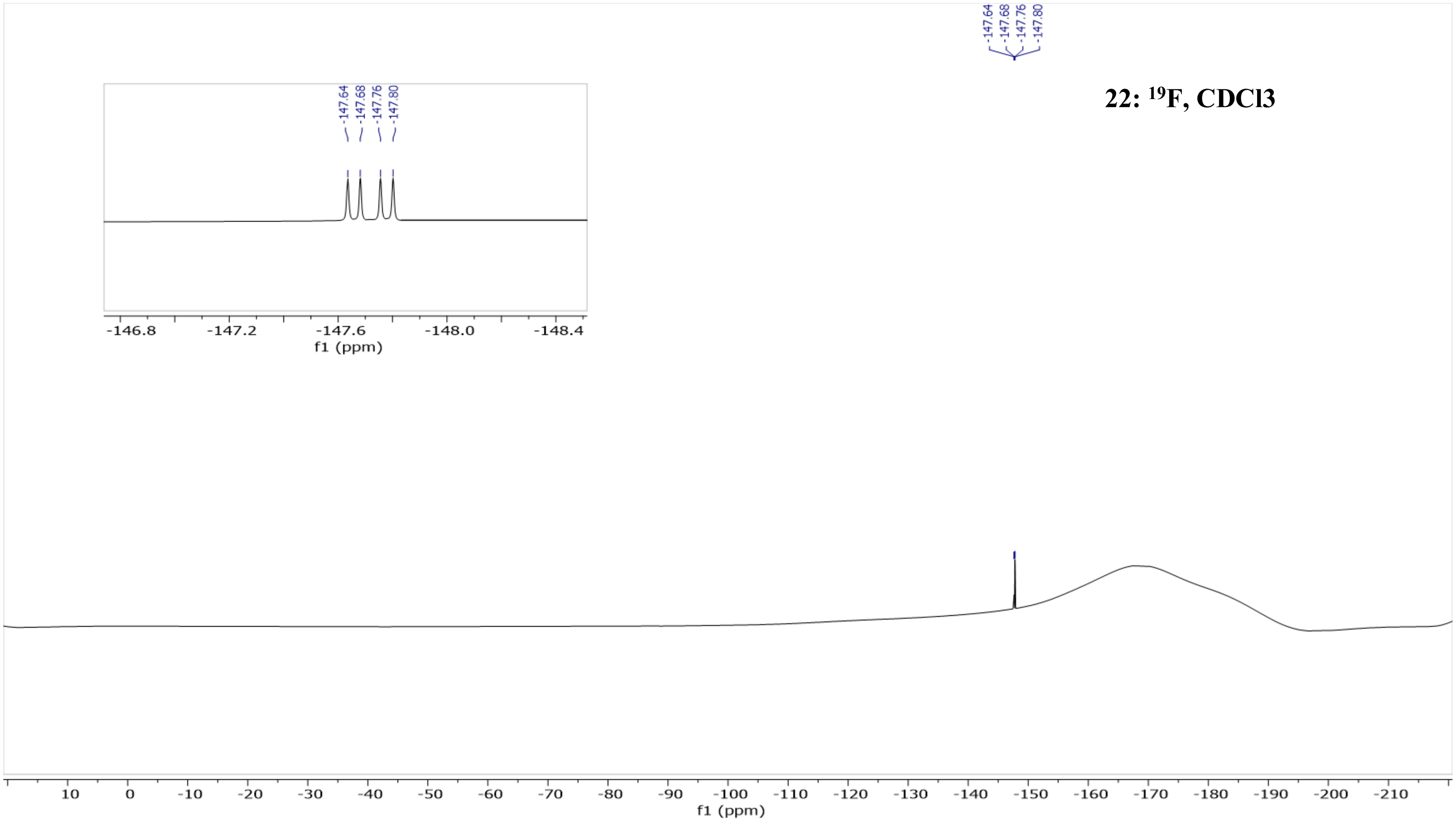

-147.64
-147.68
-147.76
-147.80
22: 19F, CDCl3
-146.8
-147.2
-147.6
-148.0
-148.4
f1 (ppm)
10
0
-10
-20
-30
-40
-50
-60
-70
-80
-90
-100
-110
-120
-130
-140
-150
-160
-170
-180
-190
-200
-210
f1 (ppm)

22: ^1H-^1H COSY, CDCl3

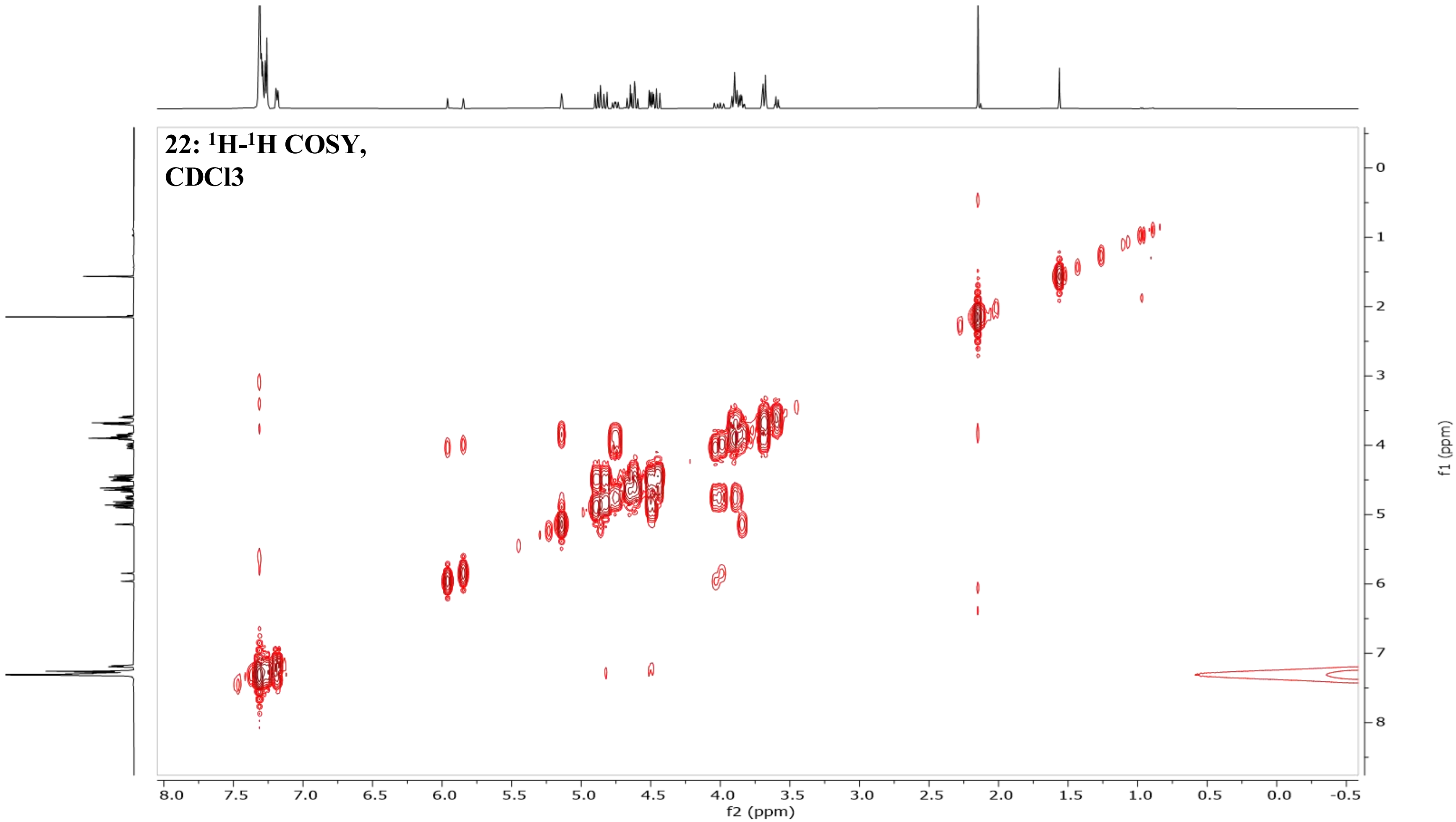

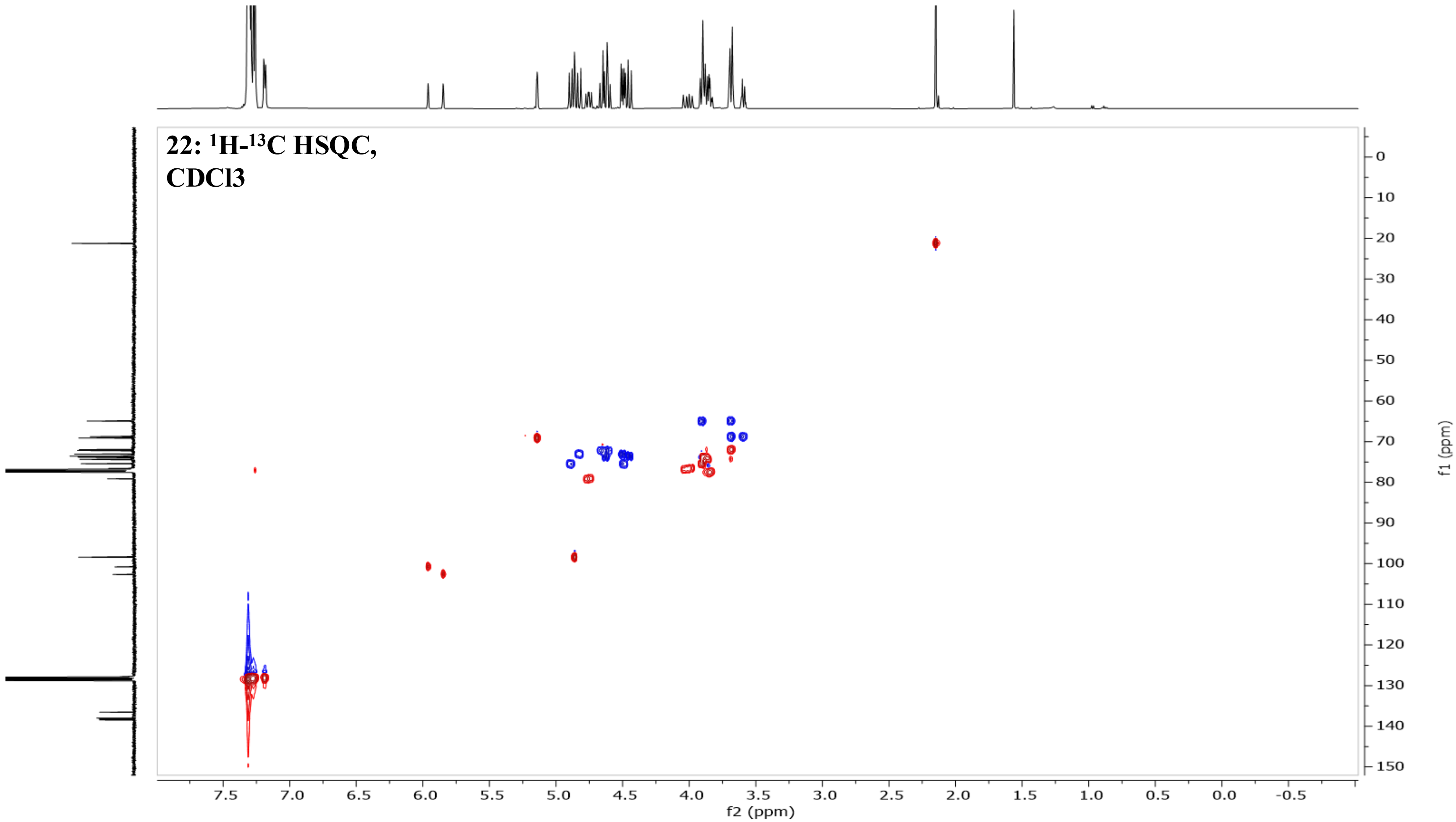

22: 1H-13C HSQC,
CDCl3
f2 (ppm)
f1 (ppm)

22: ^1H-^{13}C HSQC, Anomeric, CDCl3

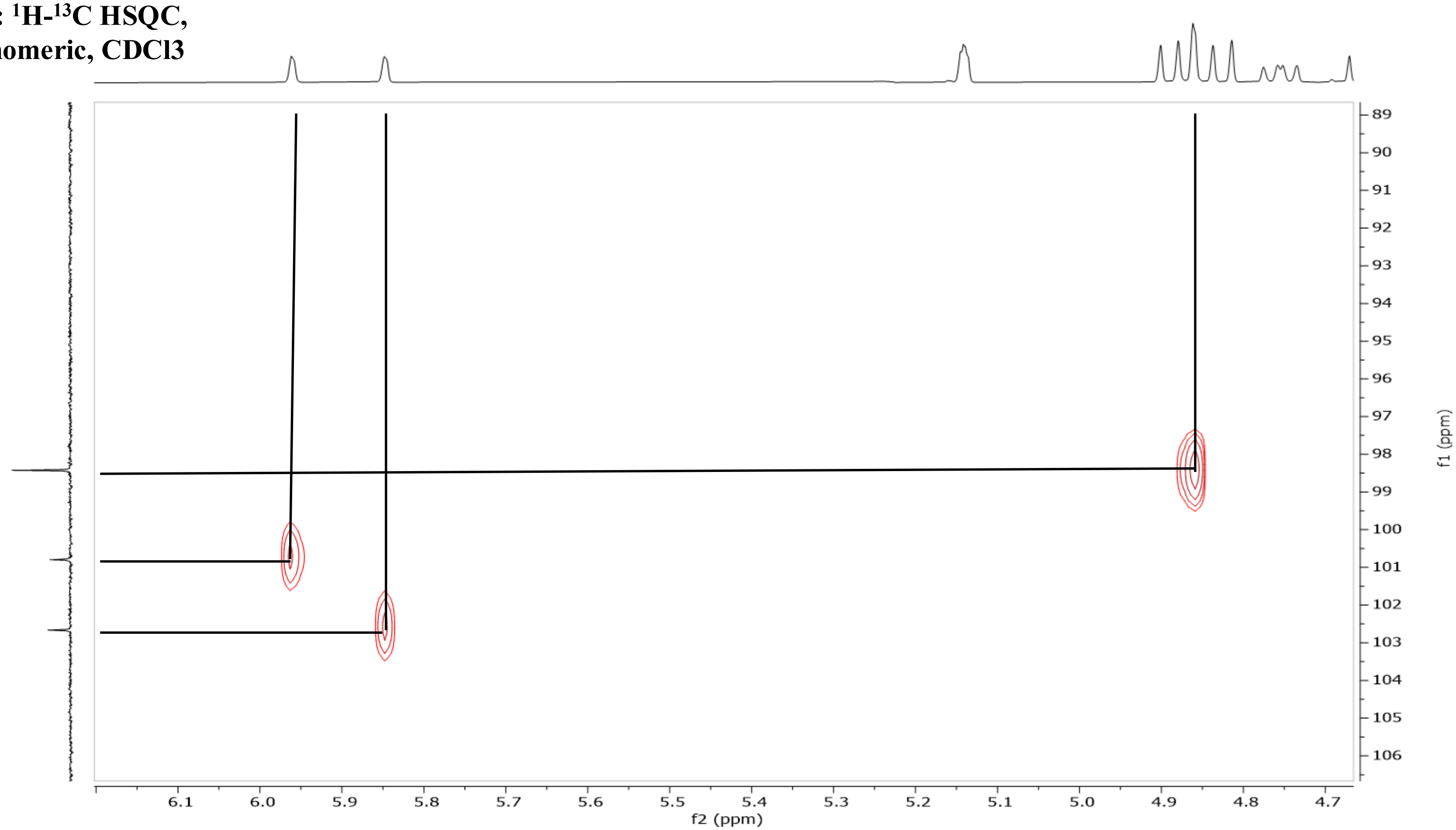

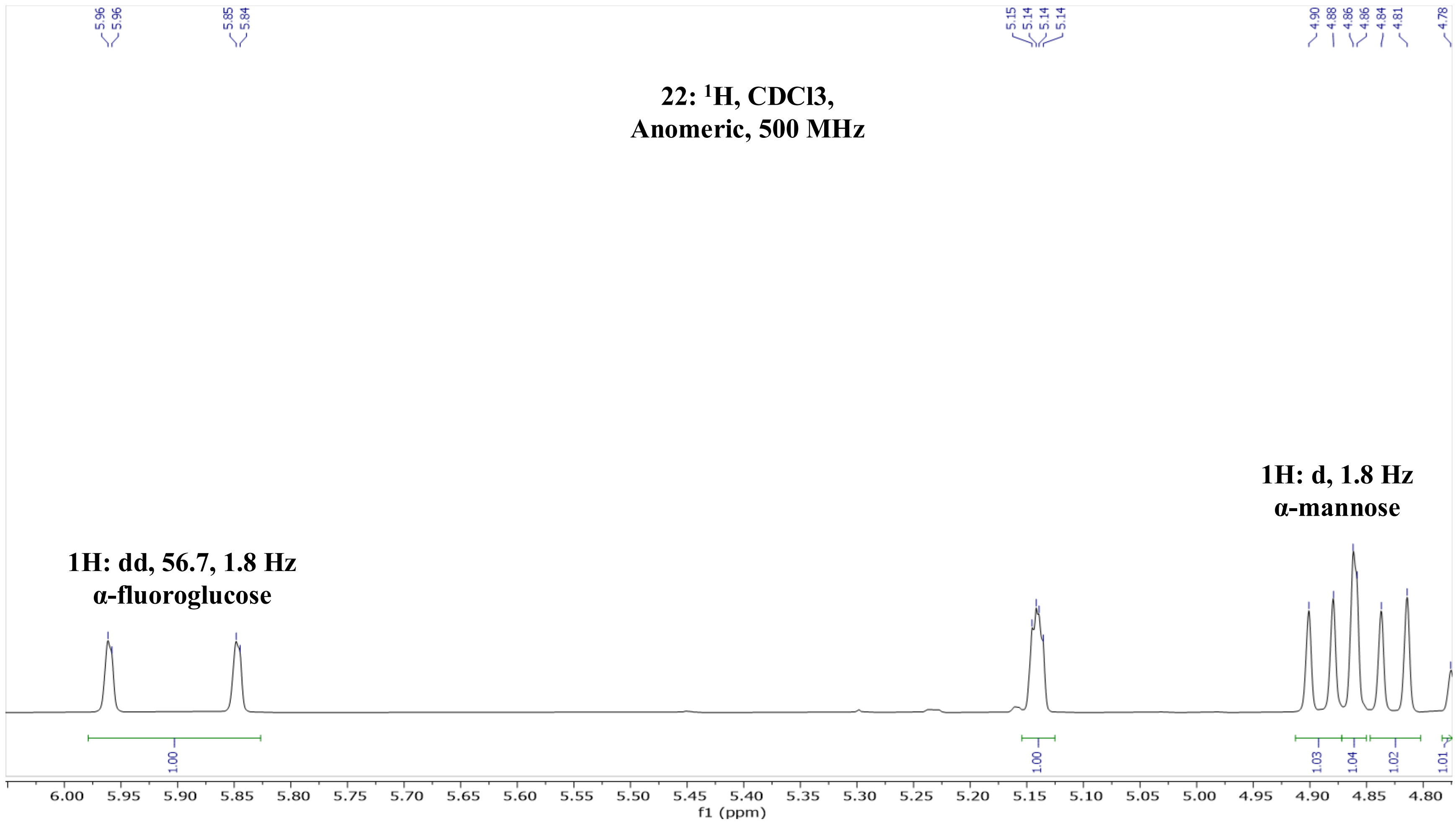
22: ¹H, CDCl3,
Anomeric, 500 MHz
1H: dd, 56.7, 1.8 Hz
α-fluoroglucose
1H: d, 1.8 Hz
α-mannose
f1 (ppm)

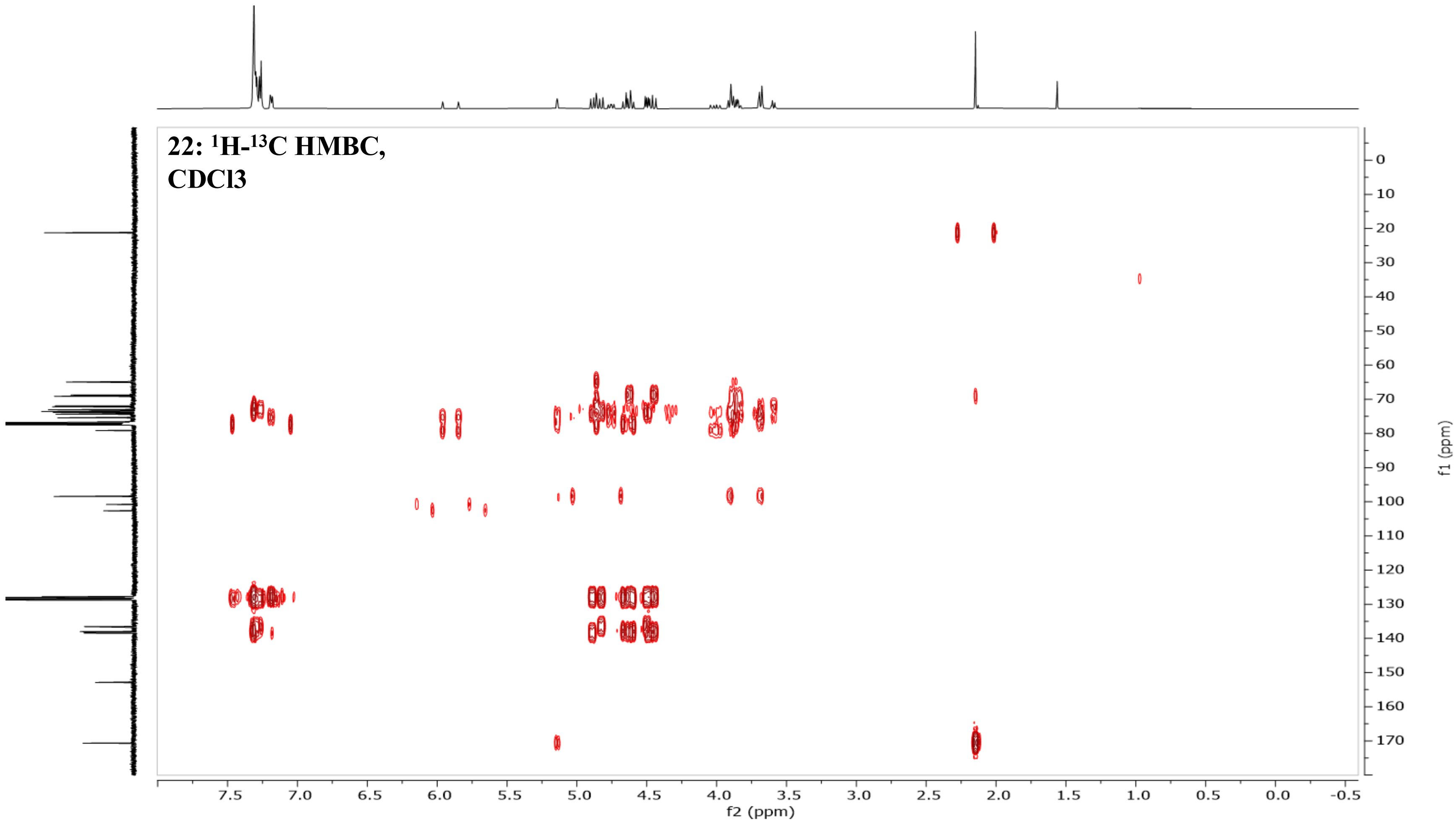
22: ¹H-¹³C HMBC,
CDCl3
f2 (ppm)
f1 (ppm)
7.5 7.0 6.5 6.0 5.5 5.0 4.5 4.0 3.5 3.0 2.5 2.0 1.5 1.0 0.5 0.0 -0.5
0 10 20 30 40 50 60 70 80 90 100 110 120 130 140 150 160 170

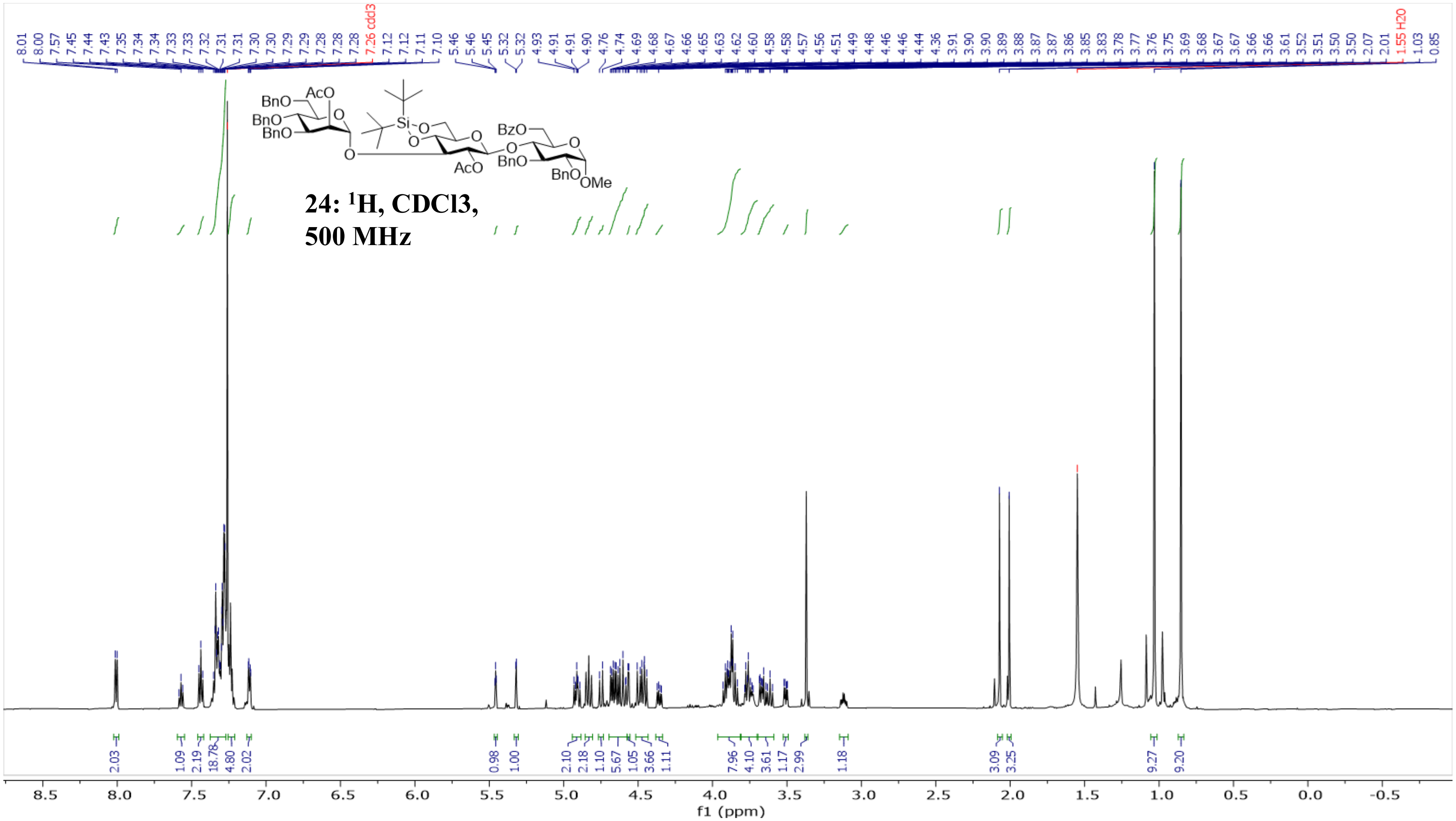
24: ¹H, CDCl3, 500 MHz
BnO
AcO
BnO
BnO
O
Si
O
O
BzO
AcO
BnO
BnO
OMe
f1 (ppm)
7.26 cdcl3
1.55 H2O

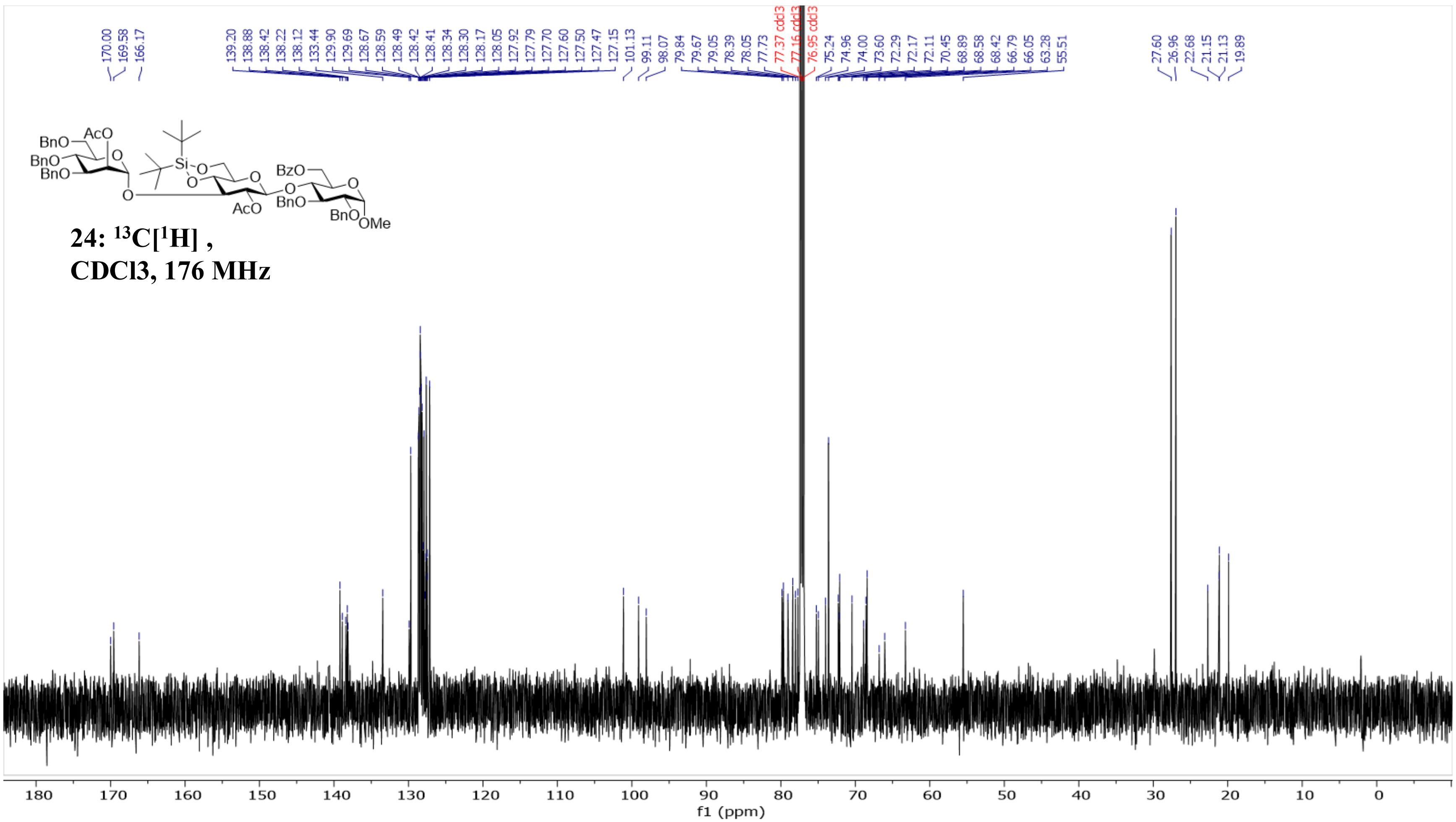

24: $^{13}C[^{1}H]$,
CDCl3, 176 MHz
170.00
169.58
166.17
139.20
138.88
138.42
138.22
138.12
133.44
129.90
129.69
128.67
128.59
128.49
128.42
128.41
128.34
128.30
128.17
128.05
127.92
127.79
127.70
127.60
127.50
127.47
127.15
101.13
99.11
98.07
79.84
79.67
79.05
78.39
78.05
77.73
77.37 cdcl3
77.16 cdcl3
76.95 cdcl3
75.24
74.96
74.00
73.60
72.29
72.17
72.11
70.45
68.89
68.58
68.42
66.79
66.05
63.28
55.51
27.60
26.96
22.68
21.15
21.13
19.89
f1 (ppm)

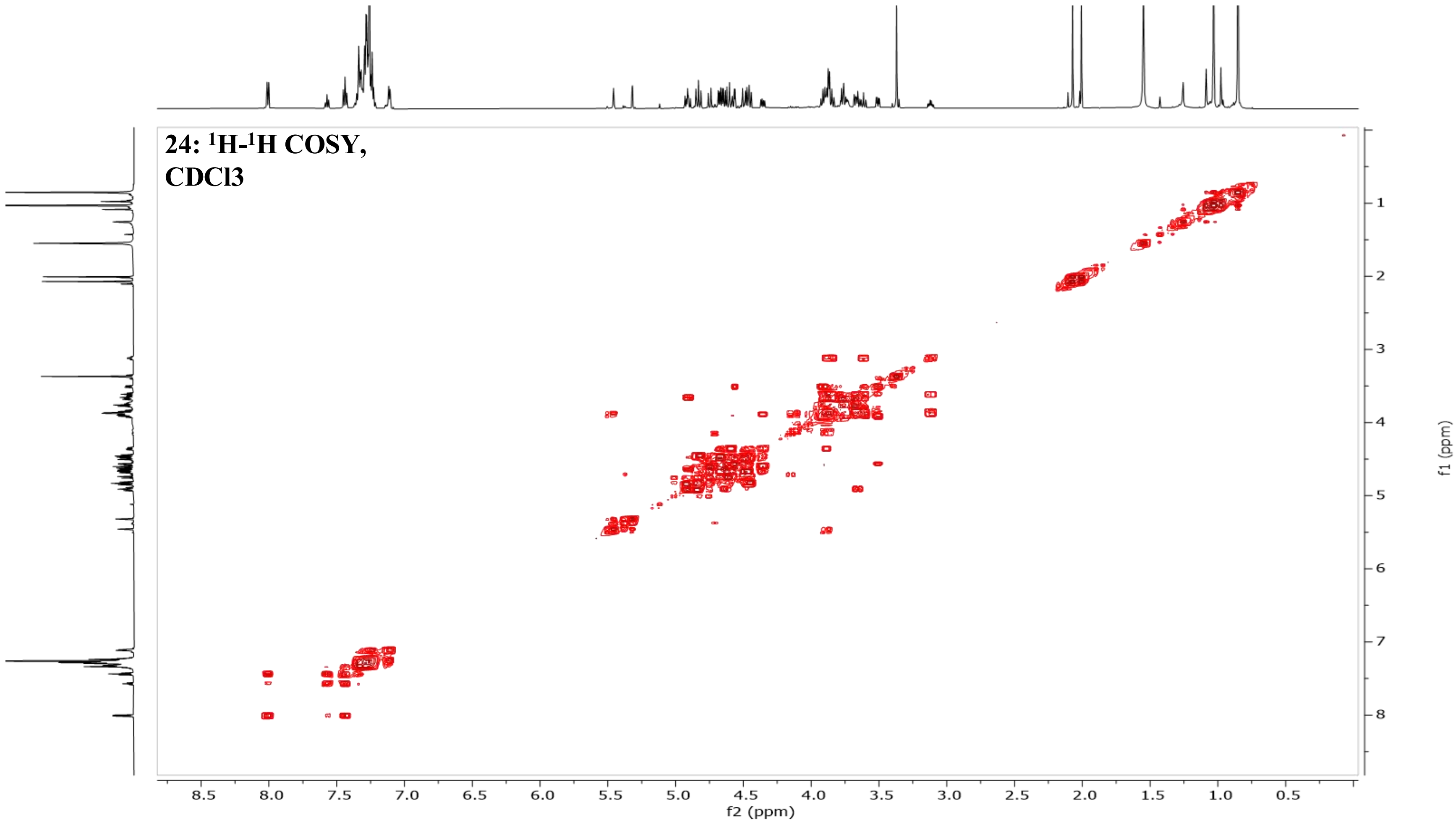

24: ¹H-¹H COSY,
CDCl3
f2 (ppm)
f1 (ppm)

24: ^{1}H-^{13}C HSQC, CDCl3

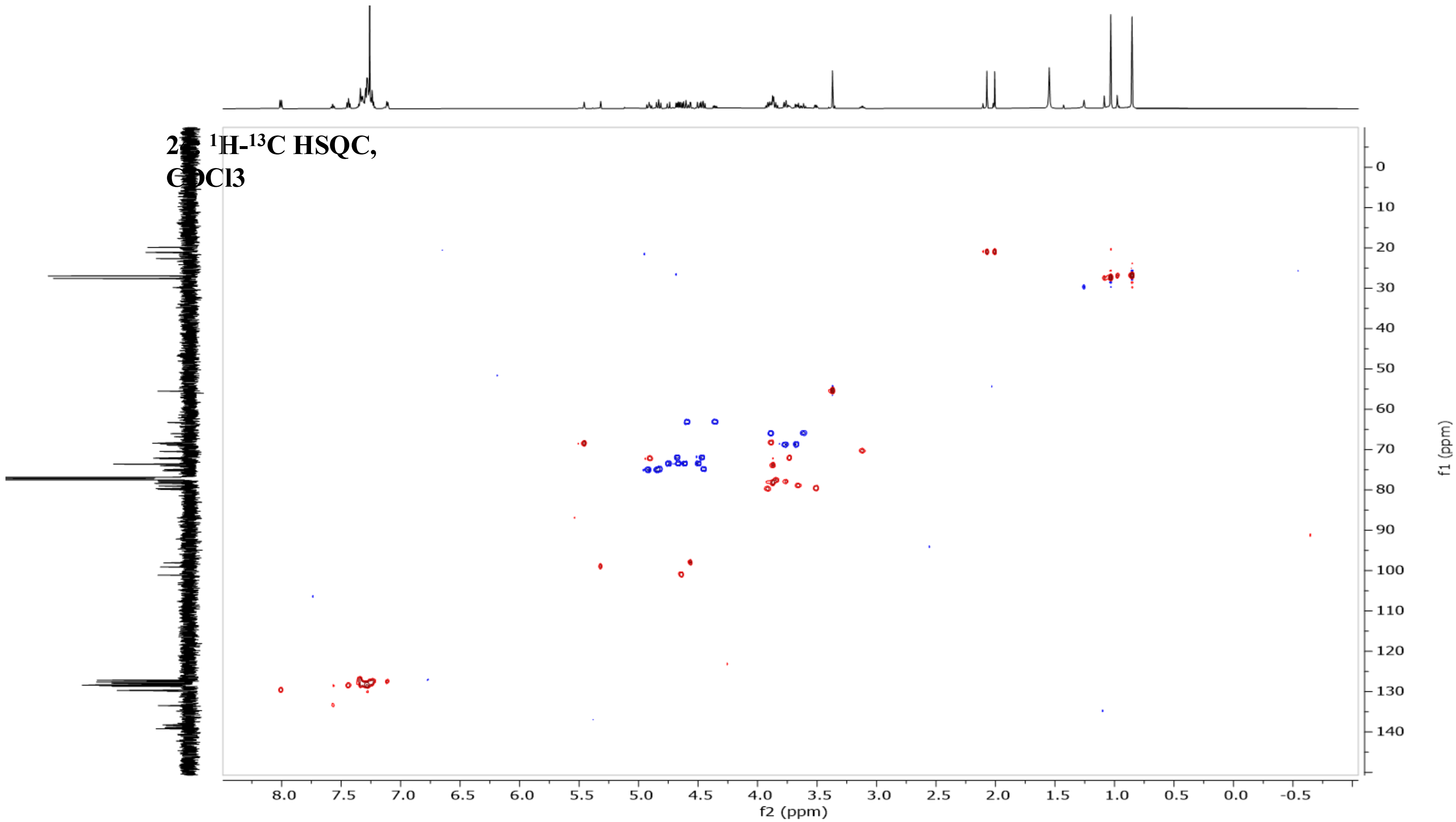

24: ^{1}H-^{13}C HSQC, Anomeric, CDCl3

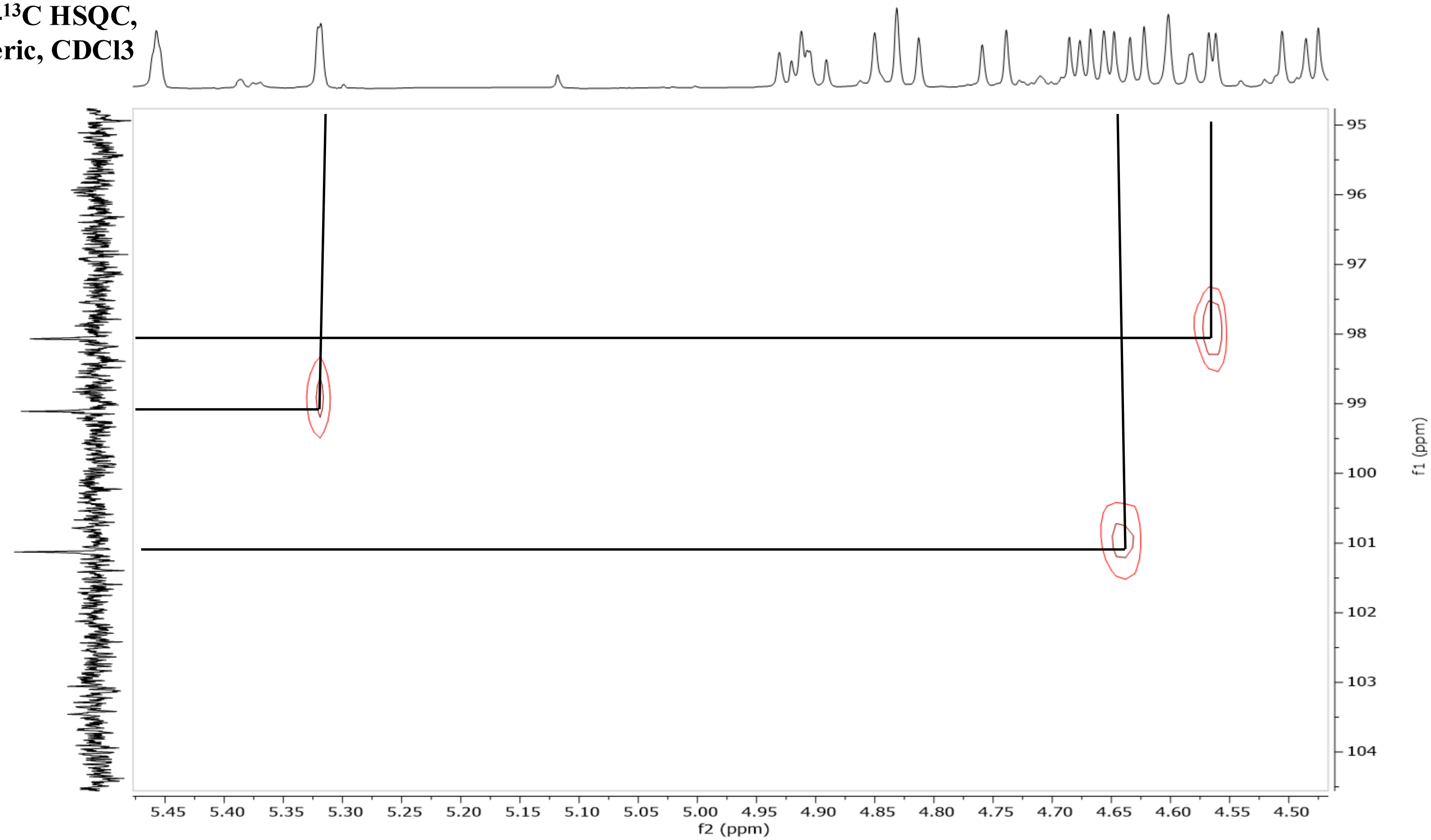

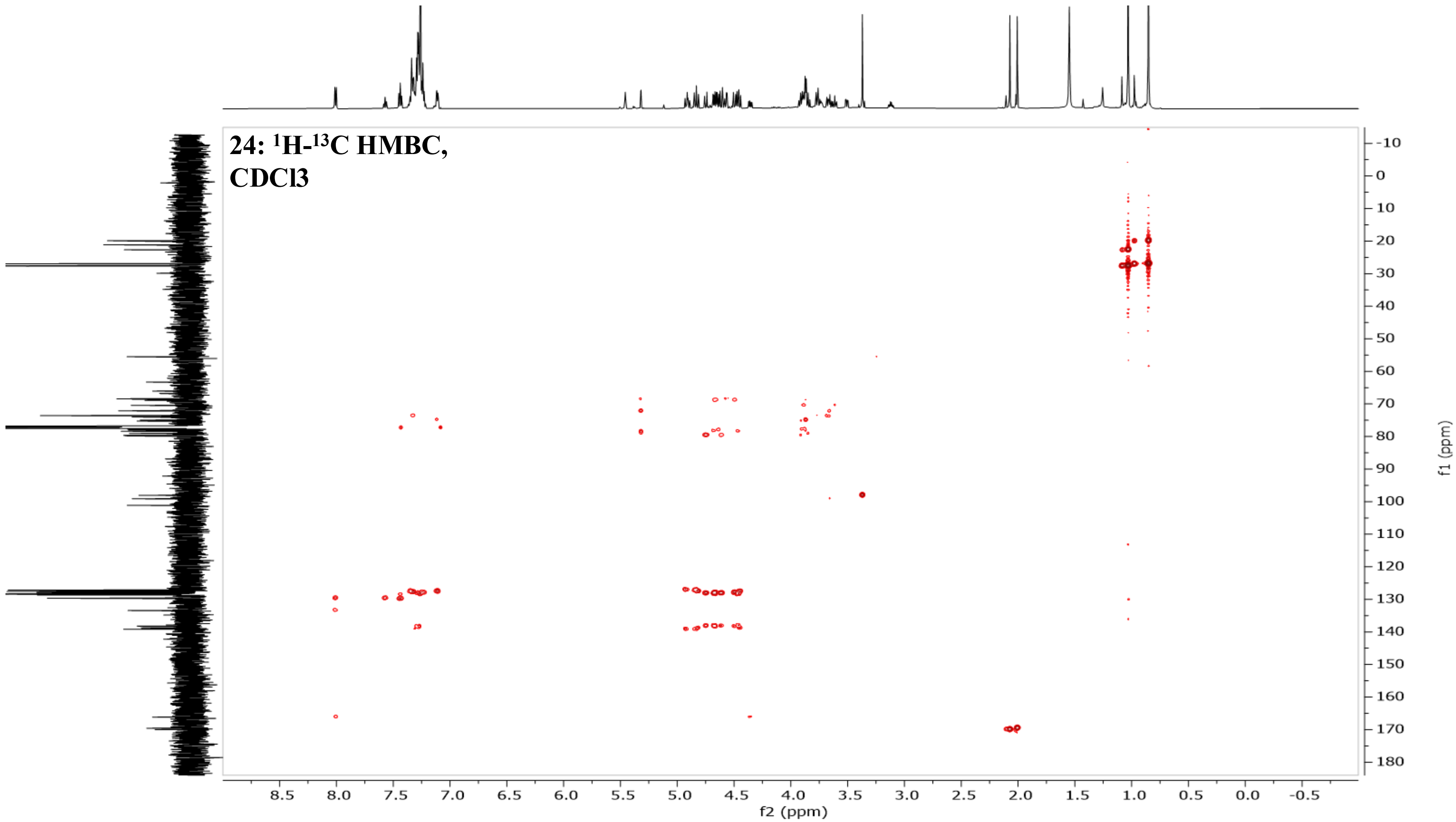
24: 1H-13C HMBC,
CDCl3
f2 (ppm)
f1 (ppm)

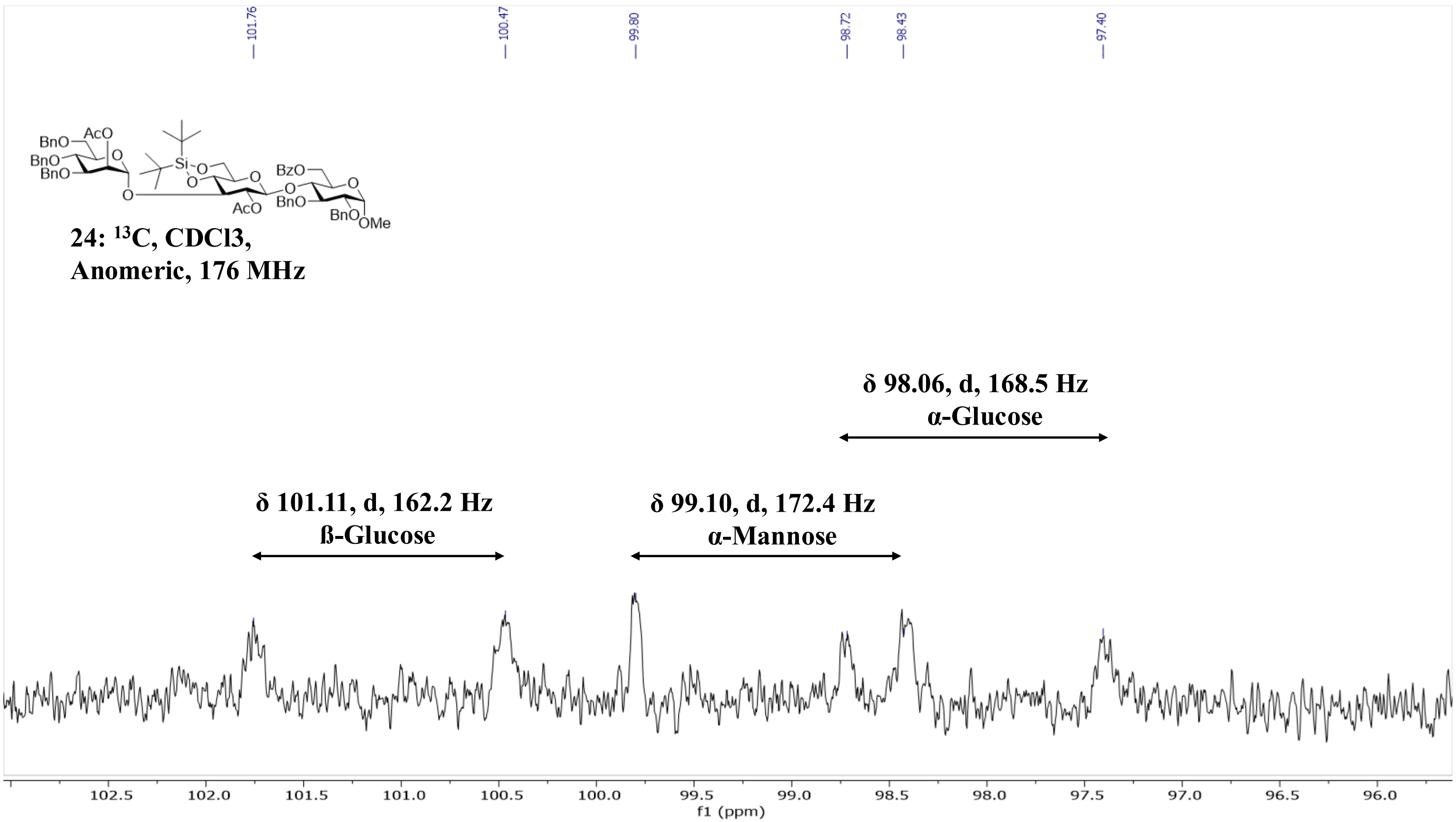
101.76
100.47
99.80
98.72
98.43
97.40
BnO
AcO
BnO
BnO
Si
O
AcO
BzO
BnO
BnO
OMe
24: 13C, CDCl3,
Anomeric, 176 MHz
δ 98.06, d, 168.5 Hz
α-Glucose
δ 101.11, d, 162.2 Hz
ß-Glucose
δ 99.10, d, 172.4 Hz
α-Mannose
102.5
102.0
101.5
101.0
100.5
100.0
99.5
99.0
98.5
98.0
97.5
97.0
96.5
96.0
f1 (ppm)
